# Effect of dairy consumption on cognition in older adults: A population-based cohort study

**DOI:** 10.1016/j.jnha.2023.100031

**Published:** 2024-01-01

**Authors:** Natalia Ortega, Cristian Carmeli, Orestis Efthimiou, Jürg-Hans Beer, Armin von Gunten, Martin Preisig, Leonardo Zullo, Julien Vaucher, Peter Vollenweider, Pedro Marques-Vidal, Nicolas Rodondi, Arnaud Chiolero, Patricia O. Chocano-Bedoya

**Affiliations:** a Institute for Primary Health Care (BIHAM), University of Bern, Bern, Switzerland; b Population Health Laboratory (#PopHealthLab), University of Fribourg, Fribourg, Switzerland; c Graduate School for Health Sciences, University of Bern, Bern, Switzerland; d Institute of Social and Preventive Medicine (ISPM), University of Bern, Bern, Switzerland; e University of Zurich and Kantonsspital Baden, Baden, Switzerland; f Service of Old Age Psychiatry, Department of Psychiatry, Lausanne University Hospital, University of Lausanne, Prilly, Switzerland; g Department of Psychiatry, Lausanne University Hospital and University of Lausanne, Lausanne, Switzerland; h Department of Medicine, Internal Medicine, Lausanne University Hospital and University of Lausanne, Lausanne, Switzerland; i Department of Medicine and Specialties, Internal Medicine, Fribourg Hospital and University of Fribourg, Fribourg, Switzerland; j Department of General Internal Medicine, Inselspital University Hospital Bern, University of Bern, Bern, Switzerland; k School of Population and Global Health, McGill University, Montreal, Canada

**Keywords:** Dairy products, Cognitive function, Diet, Aging, Cohort study

## Abstract

**Objective:**

We aimed to assess the effect on cognitive function of adding dairy (total, fermented, non-fermented, full fat, low fat, and sugary) to the diet and of substituting some food groups for dairy.

**Design:**

Secondary analysis of a prospective population-based cohort study.

**Participants:**

We analyzed data from 1334 cognitively healthy participants (median age 67 years at baseline) with a mean follow-up of 5.6 years from the CoLaus|PsyColaus cohort in Lausanne, Switzerland.

**Measurements:**

The participants completed a food frequency questionnaire at baseline and cognitive tests at baseline and at follow-up. Clinical dementia rating was the primary outcome. Subjective cognitive decline, memory, verbal fluency, executive and motor functions were secondary outcomes.

**Methods:**

Our exposure was the consumption of total and 5 sub-types of dairy products (g/d). We used marginal structural models to compute average causal effects of 1) increasing dairy consumption by 100 g/d and 2) substituting 100 g/d of meat, fish, eggs, fruits and vegetables with dairy on the outcomes. We used inverse probability of the treatment and lost to follow-up weighting to account for measured confounding and non-random loss to follow-up.

**Results:**

Overall, the effects of adding dairy products to the diet on cognition were negligible and imprecise. No substitution had a substantial and consistent effect on clinical dementia rating. The substitution of fish [11.7% (−3% to 26.5%)] and eggs [18% (2.3%–33.7%)] for dairy products could negatively impact verbal memory and neurolinguistic processes.

**Conclusion:**

We found no effect of adding dairy to the diet or substituting meat, vegetables or fruit for dairy on cognitive function in this cohort of older adults. The substitution of fish and eggs for dairy could have a negative effect on some secondary outcomes, but more studies modeling food substitutions are needed to confirm these results.

## 1 Introduction

As the global population ages, there is a growing number of people living with dementia [1]. Earlier stages of dementia include subjective cognitive decline (SCD), very mild (VMCI), and mild cognitive impairment (MCI) [2]. For instance, after a MCI diagnosis, the risk of conversion to dementia rises by 10% compared to healthy cognition [3]. Since treatment strategies have failed to reverse dementia or ameliorate cognitive function in these earlier stages [4], prevention strategies at the population level, if proven to be efficient, could be key to addressing the burden of cognitive decline and ultimately dementia. These strategies should target causal risk factors, including nutrition [5,6]. Previous studies have shown that vegetables and fruits [[7], [8], [9]], nuts [10,11] and fish [12,13] intake could prevent cognitive impairment in older adults. This effect was attributed to a high content of antioxidants and mono- and polysaturated fatty acids [14]. The relationship between cognitive impairment and the intake of other foods, such as dairy products, remains unclear.

The effect of dairy products on cognition remains controversial because various biological mechanisms could lead to effects in opposite directions. Beneficial effects are expected from cheese containing poly-unsaturated fatty acids lowering inflammation, and from a wider range of fermented products rich in anti-inflammatory components like oleamide, dehydrogesterol, peptides and living organisms that nurture the intestinal microbiota and short- and medium- chain fatty acids [[15], [16], [17], [18], [19]]. However, dairy products can also be highly caloric, rich in saturated fats [20], added salt and sugar or other sweeteners, leading to cognitive decline.

Several population-based studies have examined the relationship between dairy intake and cognitive function and have shown inconclusive results that are challenging to compare [[21], [22], [23]]. First, they included diverse outcomes (Alzheimer’s disease, vascular dementia, cognitive decline or impairment and dementia) and used different cognitive tests or case-reporting methods to measure them. Second, while studies had a causal goal, neither the targeted parameters nor the causal models and assumptions were explicitly stated. Consequently, they relied on very different sets of confounders without clear selection criteria and performed different calorie adjustment strategies that made their estimates incomparable. Besides this lack of comparability, no study included a comprehensive set of measurements of cognitive function or had baseline cognitive function assessments. Additionally, assessing food substitutions is relevant to account for the homeostatic energy content of diets and to validate established recommendations based on implicit replacements by making them explicit food substitutions. Furthermore, little research has been conducted assessing the effect of subtypes of dairy products consumption (fermented, non-fermented, full-fat, low-fat, high in sugar), usually exclusively focusing on the role of total dairy or milk consumption.

In the present study, we aimed at evaluating the long-term effects of total and subtypes of dairy intake on a comprehensive set of cognitive function measures using a causal framework and explicitly targeting two different population parameters. We hypothesized that the addition of dairy products to the diet leads to improved cognitive function, and that the substitution of other food groups (e.g., meat, fish) for dairy products could be beneficial.

## 2 Methods

### 2.1 Study design and population

**Fig. 1:**
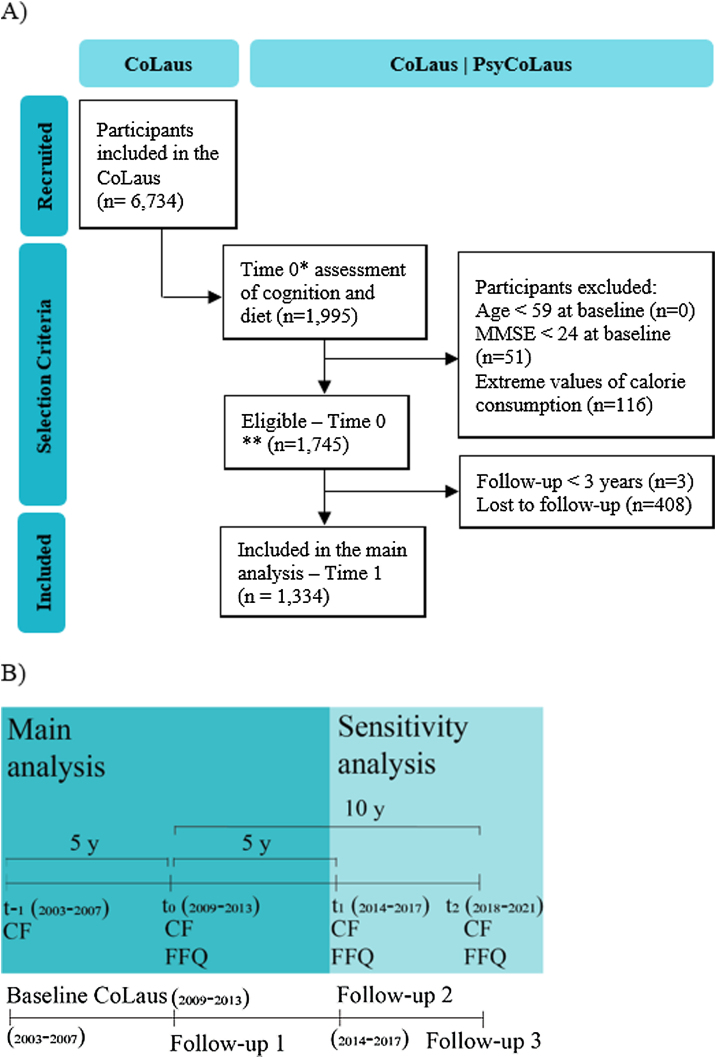
A) Participant flowchart. *Time 0 in our study corresponds to Follow-up 1 in CoLaus study. **The entire eligible population was used to calculate the weights. B) Timeline of our analysis, CF: cognitive function assessment, FFQ: food frequency questionnaires, t_-1_: baseline assessment in CoLaus cohort, t_0_: Time 0 assessment of our main analysis and Follow-up 1 in CoLaus, t_1_: Time 1 of our main analysis and Follow-up 2 in CoLaus, t_2_: Time 2 for the sensitivity analysis and Follow-up 3 in CoLaus.

Our target population were older adults living in Switzerland. Thus, we used CoLaus|PsyCoLaus as our source population to address our research question. CoLaus|PsyCoLaus initially included a random sample of 6734 people (age range: 35–75 years) selected from the residents of the city of Lausanne (Switzerland) between 2003 and 2007. Participants were reassessed approximately five (Follow-up 1, 2009–2013), nine (Follow-up 2, 2014–2017) and 13 years (Follow-up 3, 2018–2021). Dietary data were systematically collected from Follow-up 1 on and cognitive assessments in participants aged 65 years and older were performed from the same follow-up on. The data of the present analyses stemmed from Follow-ups 1–3 [24,25]. Consequently, CoLaus|PsyCoLaus Follow-up 1, 2 and 3 became Time 0, 1 and 2 in the present study and are referred like this throughout the manuscript (Fig. 1). We included participants over 59 years old, cognitively healthy at baseline (Mini-Mental State Examination - MMSE > 23), and who completed the first dietary assessment. We excluded participants with extreme total calorie intake (female <500 kcal and >3500 kcal and male <800 kcal and >4000 kcal) [26]. We considered that a participant was followed-up if they had a cognitive function re-assessment at least 3 years after the first cognitive function assessment at Time 0. If participants did not have an assessment at Time 1 or it was shorter than 3 years, we considered Time 2 (if available) as Time 1. Participant flowchart is available in Fig. 1. An informed consent for further data use was obtained from all participants and the CoLaus|PsyCoLaus study was approved by the Ethics Commission of Canton Vaud (reference PB_2018-00038, 239/09, decision of 21 June 2021).

### 2.2 Exposure: total and subtypes of dairy consumption

The dietary assessment was performed in the physical evaluation at baseline through validated semi-quantitative food frequency questionnaires (FFQ) including 97 items. Details on the procedure and coding were provided elsewhere [27]. Exposure to total dairy products was calculated as the sum of the number of grams of milk, cheese, yogurt, cream, butter, sugary desserts (e.g., ice-creams) and their variants (e.g., low fat, non-sugar). Analogously, we calculated exposure to subtypes of dairy: fermented dairy (yogurt, cheese) and non-fermented products (milk, cream, desserts), full-fat dairy (full-fat milk, yogurt, regular cheese, cream, butter and desserts, low-fat dairy (skimmed milk, yogurt, 1–2% fat cheese and low-fat desserts), sugary dairy products (cream, desserts, flavored yogurts).

### 2.3 Outcomes: cognitive assessment

Comprehensive cognitive testing was performed in psychiatric evaluations between baseline and Time 2. The primary outcome of the present analysis was clinical dementia rating (CDR), a widely used scale to assess cognitive and functional status for the clinical staging of cognitive impairment, encompassing data in six domains: memory, orientation, judgment and problem solving, community affairs, home and hobbies, and personal care [28]. In CoLaus|PsyCoLaus, a CDR of 0 was considered healthy, 0.5 corresponded to MCI and of 1 to dementia. In our sample, we coded any value above 0 as cognitive impairment.

Secondary outcomes included SCD and specific cognitive functions including praxis and episodic memory, verbal fluency, selective attention, processing speed and neurolinguistic processes. SCD was measured with the Cognitive Complaint Inventory, a validated French questionnaire [29,30]. SCD is defined as a self-perceived decline in cognitive functioning compared to a previous normal status and precedes any test that can detect an impaired cognitive function. A participant was considered a case of SCD when the subject answers “yes” to 3 or more items; and/or to item 5, and/or to items A, 4, 5, 7, 8 [31]. Episodic verbal memory was assessed through the Buschke and Groeber test [32]. It is an enhanced cued recall that is useful for memory assessment because induces semantic processing and coordinates encoding and retrieval for maximum recall. We selected the animal naming task to assess verbal semantic fluency (naming animals in 2 min). To assess selective attention skills and processing speed, the Stroop color test [33] was performed to evaluate the capacity to inhibit cognitive interference when there is more than one feature to process in a stimulus. We used the interference condition of the Stroop color test, which is the results of comparing the time it takes to name the color of a word when the ink color and the word are incongruent to when they are congruent. The Dénonimation Orale d’Images (DO40) test [34] is the oral image naming test that allowed the evaluation of neurolinguistic processes in the semantic, visual perception and the lexical aspects. Finally, CERAD (Consortium to Establish a Registry for Alzheimer’s Disease) praxis items consists of 4 tests that require drawing, copying, or articulating spatial patterns or designs and was used to evaluate the link between cognitive and motor functions.

### 2.4 Covariates

**Fig. 2:**
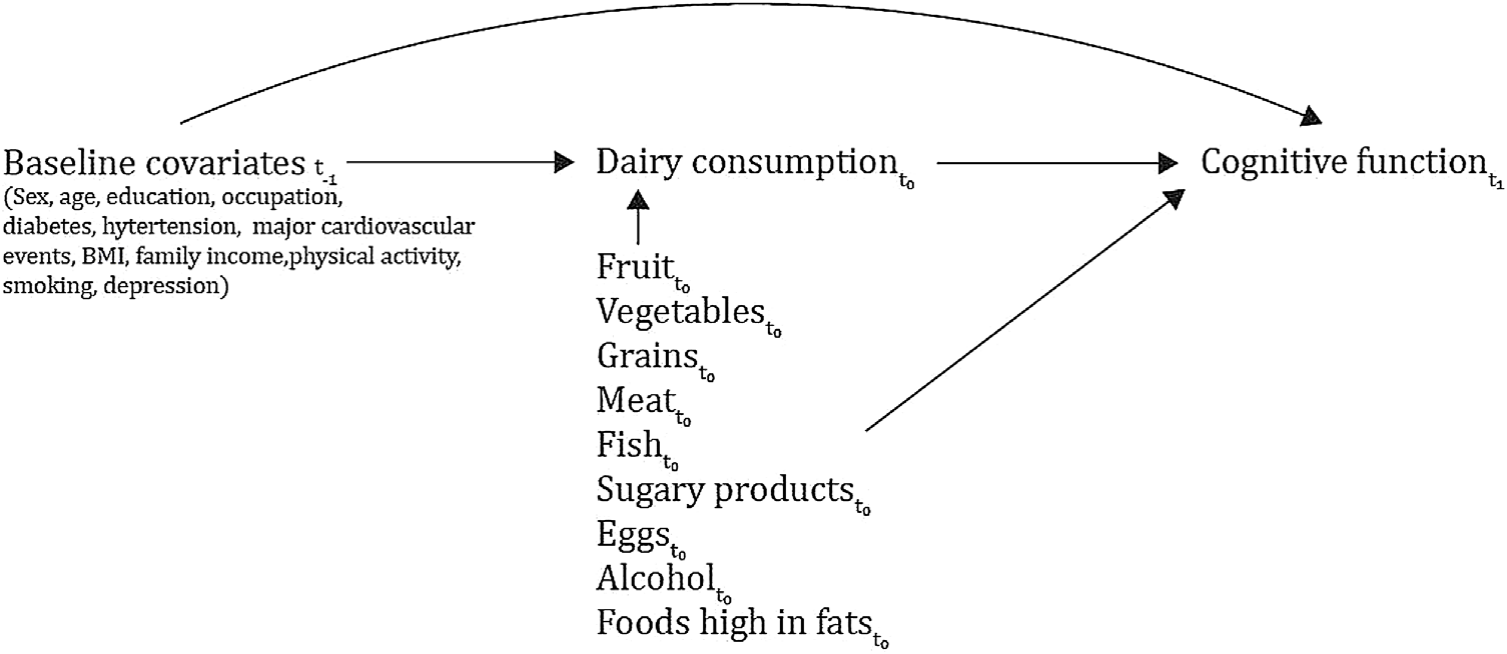
Directed acyclic graph (DAG) for causal model identification of the addition and substitution effects.t_-1_ refers to the baseline in CoLaus study, before our study Time 0, that corresponds to t_0_. t_1_ refers to Time 1 for our study and Follow-up 2 of the CoLaus study.

Participants completed questionnaires at each follow-up including their demographic and socioeconomic characteristics, lifestyle and comorbidity information. In the confounding adjustment set, we included covariates assessed prior to the exposure that we expected to be associated with cognitive function at follow-up. We first identified the covariates confounding the relationship detailed in a Directed Acyclic Graph (DAG) presented in Fig. 2 (the detailed DAG is provided in Supplementary Fig. 1). We used the following set to block all the backdoor paths in our causal model: age (<70, 70–74 and >74), sex, smoking status (current, former, never), past major cardiovascular events (self-reported - cardiomyopathy, congenital heart disease, valvular heart disease, heart failure, coronary artery disease, angina, myocardial infarction, stroke, percutaneous coronary intervention, coronary artery bypass graft or pacing) (yes/no), diabetes diagnosis or treatment (yes/no), education (elementary, high school or superior), body mass index (normal, overweight, obese), physical activity (tertiles), history of any major depressive disorder diagnosis (yes/no), family income (tertiles), occupation type and history of diagnosis of hypertension or self-reported use of hypertensive drugs (yes/no). Second, and given our goal of identifying the effect of adding dairy to the diet and substituting dairy for different food groups, we chose all-component models [35] to calculate both. Thus, we included all other food groups (vegetables, fruits, fish and seafood, meat, eggs, grains, alcohol, sugary processed products and high-in-fats foods) conforming the full diet to account for remaining dietary intake (g/d) to compute addition effects and leaving one food group out to model the substitution effects for the excluded food.

### 2.5 Causal model

The causal motivation of the study using observational data did not come without identification assumptions. To interpret our results causally we need to make four assumptions: 1) consistencyis met when we set the exposure to the observed one and the potential outcome would take the same value of the observed outcome. Consistency is sometimes referred as proposing a well-defined intervention because knowing who received which level of the “intervention” in your data is essential to meet this assumption. By specifying our parameters of interest as the addition effects - average causal effects of adding 100 g/d of dairy products to the baseline diet vs. observed consumption - and the substitution effects - average causal effect of substituting 100 g/d of a food group (e.g., meat, eggs, fruit) for 100 g/d of dairy - we had well-defined interventions with a clear interpretation and public health message, 2) conditional exchangeability is met when the exposure is independent of the potential outcomes conditional on the measured confounders. In Fig. 2 we depicted in a DAG the dependencies of the data to justify our exchangeability assumption (extended in Supplementary Fig. 1 with all the interdependencies), 3) positivity, referring to a the positive probability of being assigned to the exposure for all levels and combinations of the selected covariates and 4) no interference, meaning that the potential outcome of each participant is just affected by their own exposure, unaffected by the others.

### 2.6 Statistical analysis

We estimated Average Causal Effects (ACE) of the addition of 100 g of dairy/d to the diet compared as per usual and of the substitution effects as the difference in ACEs of different foods and dairy using stabilized inverse probability weighting (IPW) of marginal structural models (MSMs) as our estimator. MSMs are better indicated than logistic regression (or other multivariable regression approaches) in observational studies for high-multidimensional datasets and it does not assume no effect modification by the confounders as we are estimating marginal effects (not conditional) [36]. We calculated IPW of the treatment to balance the confounders at baseline using Generalized Linear Models in the eligible population (n = 1745). We truncated weights over the 99.5th percentile. To relax that missing data on the outcome was missing completely at random, we applied IPW of loss to follow-up that assumes missing at random pattern, meaning that participants were lost-to-follow-up only conditional on observed covariates at Time 0. Namely, we included age, sex, occupation, BMI, smoking, past cardiovascular events, hypertension and diabetes. Our final weight was the product between both and again truncated at 99.5th percentile. We fitted MSMs specifying binomial distributions for the counterfactual outcomes and estimated average marginal predicted probabilities of the seven outcomes. For continuous outcomes, we used the 75th percentile for all the measures because we had too little variability to use them as continuous variables and the errors of the residuals were severely skewed (memory, DO40, Stroop color test and CERAD praxis items). To compute the ACE of adding dairy to the diet (also known as total causal effects), we reported the slope of the MSMs in the units of 100 g total or subtype of dairy/d addition to the baseline diet consisting of the same amount (in g/d) of other food groups. To compute the ACE of substituting different food groups for dairy (also known as relative causal effects), we subtracted the ACE of dairy to the ACE of the food for which it was substituted (100 g/d). We excluded participants with missing data on the outcomes and therefore, we had slightly different numbers of participants for each outcome (Table 1) in the MSMs. We included all other food groups in the model. We calculated parametric 95% confidence intervals (CIs) of the estimates.

**Table 1:** Baseline characteristics of the study sample stratified by servings per day (/d) of total dairy consumption. SMD: Standardized mean difference, IQR: Interquartile range, CDR: Clinical dementia rating, SCD: Subjective cognitive decline, DO40: Denomination Orale test, CERAD: Consortium to Establish a Registry for Alzheimer's Disease.

	Once or less than once/d	More than once but less than 3 times/d	3 or more times/d	SMD
n	145	788	813	
Sex (%)				0.115
Male	54 (37.2)	360 (45.7)	326 (40.1)	
Female	91 (62.8)	428 (54.3)	487 (59.9)	
Age (%)				0.249
Below 70	101 (69.7)	492 (62.4)	472 (58.1)	
Between 70 and 74	33 (22.8)	174 (22.1)	178 (21.9)	
Over 74	11 (7.6)	122 (15.5)	163 (20.0)	
Education (%)				0.109
Elementary	104 (71.7)	518 (65.7)	568 (69.9)	
High school	18 (12.4)	134 (17.0)	126 (15.5)	
Superior	23 (15.9)	136 (17.3)	119 (14.6)	
Occupation (%)				0.162
High	4 (2.8)	39 (5.1)	42 (5.2)	
Middle	16 (11.3)	95 (12.4)	81 (10.1)	
Low	25 (17.6)	85 (11.1)	98 (12.2)	
Not working	97 (68.3)	546 (71.4)	583 (72.5)	
Missing	30			
BMI (%)				0.075
Normal	60 (41.7)	291 (37.4)	313 (38.9)	
Overweight	54 (37.5)	333 (42.8)	334 (41.5)	
Obese	30 (20.8)	154 (19.8)	158 (19.6)	
Smoking (%)				0.124
Never	61 (42.1)	310 (39.5)	359 (44.3)	
Former	57 (39.3)	362 (46.1)	336 (41.4)	
Current	27 (18.6)	113 (14.4)	116 (14.3)	
Past cardiovascular event - Yes (%)	28 (19.4)	159 (20.3)	166 (20.4)	0.016
Hypertension - Yes (%)	94 (64.8)	530 (67.3)	543 (66.9)	0.035
Alcohol consumption (%)				0.15
Non-drinkers	33 (24.6)	163 (22.7)	214 (29.0)	
Light drinkers	58 (43.3)	348 (48.5)	348 (47.2)	
Moderate and heavy drinkers	43 (32.1)	207 (28.8)	176 (23.8)	
Depression - Yes (%)	21 (16.9)	71 (9.9)	98 (13.2)	0.139
Missing	122			
Total calories (kcal/day) (median, IQR)	1236 (421)	1596 (506)	1855 (578)	0.826
Family income (%) Tertiles				0.148
Lowest tertile	39 (44.8)	194 (37.5)	211 (40.5)	
Middle tertile	40 (46.0)	242 (46.7)	241 (46.3)	
Highest tertile	8 (9.2)	82 (15.8)	69 (13.2)	
Missing	297			
Diabetes - Yes (%)	19 (13.1)	80 (10.2)	107 (13.2)	0.063
Physical activity (%)				0.079
High	25 (20.5)	139 (20.1)	129 (18.0)	
Low	58 (47.5)	302 (43.8)	336 (47.0)	
Medium	39 (32.0)	249 (36.1)	250 (35.0)	
Missing	224			

Cognitive measure	Time 0	Time 1	Time 2
	Proportion (%)	Proportion (%)	Proportion (%)
CDR (Impaired vs. healthy)	466/975 (47.8)	429/918 (46.7)	181/428 (42.3)
SCD (Impaired vs. healthy)	177/966 (18.3)	178/974 (18.3)	76/459 (16.6)
Verbal (lower than 36 vs. 36–58)	213/921 (23.1)	474/955 (49.6)	80/451 (17.7)
Memory (lower than 48 vs. 48)	483/921 (52.4)	174/953 (18.3)	326/450 (72.4)
DO40 (lower than 40 vs. 40)	130/933 (13.9)	182/956 (19.0)	128/459 (27.9)
Stroop (lower than 24 vs. 24)	295/965 (30.6)	312/967 (32.3)	70/452 (15.5)
CERAD praxis items (lower than 11 vs. 11)	305/974 (31.3)	524/982 (53.4	233/463 (50.3)

We performed sensitivity analyses to test the assumptions made in our analysis. First, we calculated the cumulative average of the reported dairy and the other food groups consumption in two FFQ (at baseline and after five years, corresponding to Time 1). We used data from the Time 0 assessment and Time 1 for the exposure to dairy products and the cognitive tests at Time 2 after 10 years (instead of after 5 years in the main analysis) (Fig. 1). We used the same inclusion criteria and covariates that at baseline. Thereby, we intended to capture the variability of the diet in the longer term to reduce measurement bias, and to explore how the shorter follow-up in the main analysis could have influenced the estimates. Second, we fitted linear mixed effects models with random intercepts for individuals with repeated measurements, to estimate cognitive decline over time. We used binary outcomes for CDR and SCD and kept the variables continuous for the other cognitive outcomes. We specified binomial and gaussian distribution for the errors, respectively. Models were standardized for the same baseline covariates using IPW. We ran this sensitivity analysis for addition effects only. Third, we also considered death as a competing event for a sensitivity analysis.

We did not adjust for multiple comparisons because we interpreted confidence intervals cautiously and assessed consistent trends across different cognitive tests for different cognitive domains rather than single tests’ estimates. We reported standardized mean differences (SMD) to quantify the differences in baseline characteristics and the covariate balance after IPW. The IPW models excluded incomplete cases, but MSMs were run in the full sample. We considered SMDs <0.1 to be well-balanced across units of dairy consumption. We performed all the analyses in R version 4.3.1 (2023-06-10) using the tidyverse [37], MASS [38], broom [39], survey [40], ggpubr [41], and geepack [42] packages. The code and outputs were developed and using Jupyter Notebook and can be found in Supplementary File 2.

## 3 Results

We included 1334 adults (61.7% females) with a median follow-up of 5.2 years (range: 3.0–7.4 years) and a median age of 67 (range 59–82) years. Descriptive characteristics stratified by total dairy consumption level of our sample are shown in Table 1. Total dairy consumption was lower in younger and female participants, who were more likely to consume one or less than one dairy serving/d, whereas males usually consumed between 2 and 3 servings/d. Standard mean deviation (SMD) for the baseline characteristics were balanced (SMD < 0.1) after IPW and are presented in Supplementary Table 1. The range of dairy consumption is described in Supplementary Table 2. At follow-up, 364 (46%) had a CDR score of 0.5. Related to the secondary outcomes, 142 (18%) of the participants reported SCD. Forty-nine percent of the participants scored lower than 36 (maximum 58) in the verbal assessment, 18.3%, 19%, 32%, 53% had a lower score than the maximum possible for memory, DO40, Stroop and CERAD praxis items, respectively (Table 1).

The differences between participants in the full CoLaus cohort versus the subset included in CoLaus|PsyCoLaus have been described previously [25]. However, we also found some differences between participants excluded from the analysis because they had no information on cognition at follow-up (n = 411) were most frequently male, older, more hypertension, higher BMI, higher depression prevalence and diabetes (Supplementary Table 3).

### 3.1 Average causal effects of adding dairy to the diet

The estimated ACEs of adding total or any subtype of dairy to the diet were small increases in the probability of scoring under 75th percentile in cognitive function tests (Table 2). The risk of CDR, our primary outcome, for the increase of total dairy consumption was 1.5% (−0.5% to 3.5%). Similar magnitude and precision applied to the addition of all other dairy subtypes and CDR, except for full-fat and sugary dairy that the effect estimates were 2.4% (0.1%–4.6%) and 4.4% (0.8%–8.0%) increase in the risk difference, respectively. Effect estimates for total dairy were also imprecise for all the secondary outcome measures. All the estimates were non-informative or had negligible effect sized across secondary cognitive outcomes.

**Table 2:** Average Causal Effect estimates of adding 100 g of dairy per day to the baseline diet. CDR: Clinical dementia rating, SCD: Subjective cognitive decline, DO40: Dénonimation Orale d’Images. In parenthesis are 95% confidence intervals.

	CDR	SCD	Memory	Verbal fluency	Stroop	DO40	CERAD
Total dairy	1.52% (−0.49% to 3.52%)	0.88% (−1.05% to 2.81%)	1.97% (−0.2% to 4.14%)	−0.31% (−2.16% to 1.54%)	1% (−1.27% to 3.27%)	−0.29% (−2.62% to 2.05%)	0.87% (−1.33% to 3.07%)
Fermented dairy	1.1% (−1.79% to 3.99%)	0.13% (−2.43% to 2.7%)	2.62% (−0.55% to 5.79%)	−0.68% (−3.17% to 1.8%)	2.12% (−1.08% to 5.33%)	−2.18% (−4.95% to 0.59%)	1.13% (−1.88% to 4.14%)
Non fermented dairy	0.1% (−3.57% to 3.78%)	1.87% (−0.75% to 4.48%)	1.92% (−0.68% to 4.51%)	−0.62% (−3.83% to 2.59%)	−0.84% (−4.09% to 2.4%)	2.9% (−0.47% to 6.27%)	2.14% (−1.4% to 5.68%)
Full fat dairy	2.35% (0.08% to 4.63%)	1.1% (−0.93% to 3.13%)	2.09% (−0.04% to 4.23%)	−0.54% (−2.61% to 1.53%)	1.43% (−1.07% to 3.93%)	−1.92% (−4.73% to 0.89%)	0.1% (−2.42% to 2.62%)
Low fat dairy	−7.85% −16.09% to 0.4%)	4.6% (−2.44% to 11.65%)	5.91% (−4.16% to 15.98%)	−8.1% (−17.34% to 1.14%)	−3.45% (−9.97% to 3.06%)	1.01% (−8.24% to 10.26%)	−4.48% (−12.88% to 3.91%)
Sugary dairy	4.4% (0.76% to 8.03%)	2.39% (−1.95% to 6.73%)	1.23% (−3.02% to 5.47%)	1.91% (−1.13% to 4.95%)	2.98% (−1.55% to 7.5%)	−0.15% (−4.76% to 4.47%)	0.2% (−3.98% to 4.39%)

### 3.2 Average causal effects of substituting other food groups for dairy

No food substitution for total dairy product led to a relevant risk difference for our primary outcome. The substitution estimates were of small magnitude and had inconsistent directions for total and subtypes of dairy. The substitution of vegetables for full fat and sugary dairy increased the risk of CDR by 4.4% (0.4%–8.4%) and 6.9% (2.1%–11.8%), respectively, at follow-up. These results were confirmed in the sensitivity analysis.

For the secondary outcomes, we observed some noteworthy trends for the substitution of fish and eggs for dairy. The substitution for fish led to a 11.7% (−3% to 26.5%) increased risk for DO40, and of similar magnitude and precision across dairy subtypes. The substitution for eggs led to a 15% (−1.9% to 31.9%) and 18% (2.3%–33.7%) higher probability of scoring below the 75th percentile for memory and DO40 score, respectively, also consistent for all dairy subtypes. Finally, the substitutions of meat and fruit for dairy were of very small magnitude and again, inconsistent in their directions for all secondary cognitive measures, for total dairy and subtypes (Table 3).

**Table 3:** Average Causal Effect of substitution estimates for cognition outcomes of substituting 100 g of dairy for a different food group. CDR: Clinical dementia rating, SCD: Subjective cognitive decline, DO40: Dénonimation Orale d’Images.

	Total dairy	Fermented dairy	Non fermented dairy	Full fat dairy	Low fat dairy	Sugary dairy
CDR						
Meat	−1.3% (−9.9% to 7.3%)	−1.6% (−10.4% to 7.2%)	−1.7% (−10.7% to 7.3%)	−0.3% (−8.9% to 8.4%)	−8.7% (−19.5% to 2.1%)	2.2% (−6.8% to 11.3%)
Fish	0.5% (−16% to 17.1%)	0.2% (−16.4% to 16.9%)	0.1% (−16.7% to 16.9%)	1.6% (−15% to 18.2%)	−6.8% (−24.6% to 11%)	4.1% (−12.7% to 20.9%)
Eggs	10.3% (−17.9% to 38.5%)	10% (−18.2% to 38.3%)	9.9% (−18.4% to 38.2%)	11.4% (−16.8% to 39.6%)	3% (−26% to 31.9%)	13.9% (−14.4% to 42.2%)
Vegetables	3.4% (−0.5% to 7.3%)	3.1% (−1.3% to 7.4%)	3% (−1.8% to 7.7%)	4.4% (0.4% to 8.4%)	−4% (−11.6% to 3.6%)	6.9% (2.1% to 11.8%)
Fruits	0.4% (−2.4% to 3.3%)	0.2% (−3.3% to 3.6%)	0% (−3.9% to 4%)	1.5% (−1.5% to 4.5%)	−6.9% (−14% to 0.2%)	4% (0% to 8%)
SCD						
Meat	−0.8% (−10.3% to 8.6%)	−2% (−11.7% to 7.7%)	0.1% (−9.6% to 9.8%)	−0.6% (−10.1% to 8.9%)	−2.6% (−13.2% to 8%)	0.2% (−9.8% to 10.2%)
Fish	4.5% (−7.3% to 16.2%)	3.3% (−8.7% to 15.3%)	5.4% (−6.6% to 17.4%)	4.7% (−7.2% to 16.5%)	2.7% (−10.1% to 15.4%)	5.5% (−6.8% to 17.7%)
Eggs	−1.7% (−28.2% to 24.8%)	−2.9% (−29.5% to 23.7%)	−0.8% (−27.4% to 25.8%)	−1.5% (−28% to 25%)	−3.5% (−30.4% to 23.4%)	−0.7% (−27.4% to 26%)
Vegetables	1% (−1.9% to 3.8%)	−0.2% (−3.9% to 3.5%)	1.9% (−1.7% to 5.5%)	1.2% (−1.9% to 4.3%)	−0.8% (−6.4% to 4.8%)	2% (−2.4% to 6.4%)
Fruits	2.5% (0.5% to 4.5%)	1.4% (−1.7% to 4.4%)	3.4% (0.5% to 6.4%)	2.7% (0.4% to 5.1%)	0.7% (−4.5% to 6%)	3.5% (−0.4% to 7.4%)
Memory						
Meat	−1.1% (−9.9% to 7.7%)	0.3% (−9.2% to 9.8%)	−0.5% (−9.6% to 8.5%)	−0.3% (−9.2% to 8.7%)	−1.4% (−11.3% to 8.5%)	−1.7% (−11.1% to 7.7%)
Fish	−3.3% (−17.6% to 11%)	−2% (−16.7% to 12.7%)	−2.8% (−17.3% to 11.7%)	−2.5% (−16.9% to 11.9%)	−3.6% (−18.7% to 11.4%)	−4% (−18.6% to 10.7%)
Eggs	15% (−1.9% to 31.9%)	16.4% (−0.9% to 33.6%)	15.6% (−1.5% to 32.6%)	15.8% (−1.1% to 32.8%)	14.7% (−2.8% to 32.2%)	14.4% (−2.8% to 31.6%)
Vegetables	−0.4% (−5.1% to 4.3%)	0.9% (−4.9% to 6.8%)	0.1% (−5.1% to 5.3%)	0.4% (−4.5% to 5.3%)	−0.7% (−7.3% to 5.8%)	−1.1% (−6.8% to 4.6%)
Fruits	−0.4% (−3% to 2.3%)	1% (−3.4% to 5.4%)	0.2% (−3.3% to 3.6%)	0.5% (−2.6% to 3.5%)	−0.7% (−5.9% to 4.6%)	−1% (−5.2% to 3.2%)
Verbal fluency						
Meat	5.3% (−3% to 13.6%)	4.3% (−4.3% to 12.8%)	4.5% (−4.3% to 13.2%)	4.5% (−3.9% to 12.9%)	7.3% (−2.4% to 17.1%)	7.1% (−1.6% to 15.8%)
Fish	10.2% (−3.7% to 24%)	9.1% (−4.8% to 23.1%)	9.3% (−4.8% to 23.4%)	9.4% (−4.5% to 23.3%)	12.2% (−2.5% to 26.9%)	12% (−2.1% to 26.1%)
Eggs	−5% (−27% to 17.1%)	−6% (−28.2% to 16.1%)	−5.8% (−28% to 16.4%)	−5.8% (−27.8% to 16.3%)	−2.9% (−25.6% to 19.7%)	−3.2% (−25.4% to 19.1%)
Vegetables	−0.9% (−4.1% to 2.3%)	−1.9% (−5.7% to 1.9%)	−1.7% (−6% to 2.6%)	−1.6% (−5.1% to 1.8%)	1.2% (−4.9% to 7.2%)	1% (−3.2% to 5.1%)
Fruits	0.1% (−2.2% to 2.5%)	−0.9% (−3.9% to 2.2%)	−0.7% (−4.4% to 3%)	−0.6% (−3.3% to 2.1%)	2.2% (−3.5% to 7.8%)	2% (−1.5% to 5.5%)
Stroop						
Meat	6.8% (−7.9% to 21.5%)	10% (−5% to 25%)	5.5% (−9.6% to 20.5%)	8.4% (−6.5% to 23.2%)	4.7% (−11.7% to 21.1%)	9.1% (−6.1% to 24.3%)
Fish	−9.1% (−25.2% to 7%)	−5.9% (−22.2% to 10.4%)	−10.4% (−26.8% to 6%)	−7.5% (−23.7% to 8.7%)	−11.2% (−28.8% to 6.5%)	−6.8% (−23.3% to 9.8%)
Eggs	2.8% (−26.1% to 31.7%)	6% (−23% to 35%)	1.5% (−27.6% to 30.6%)	4.4% (−24.6% to 33.4%)	0.8% (−29% to 30.5%)	5.2% (−24% to 34.3%)
Vegetables	−1.4% (−5.9% to 3.1%)	1.8% (−3.5% to 7.1%)	−2.7% (−8.2% to 2.8%)	0.2% (−4.7% to 5.1%)	−3.5% (−12% to 5%)	0.9% (−4.9% to 6.8%)
Fruits	−0.5% (−2.8% to 1.9%)	2.7% (−1% to 6.4%)	−1.8% (−5.8% to 2.1%)	1.1% (−2% to 4.2%)	−2.6% (−10.2% to 5%)	1.8% (−2.6% to 6.3%)
DO40						
Meat	0% (−9.1% to 9%)	−1.7% (−11% to 7.5%)	2.4% (−7.3% to 12.1%)	−1.5% (−10.8% to 7.8%)	2.5% (−9.5% to 14.4%)	−0.6% (−10.3% to 9%)
Fish	11.7% (−3% to 26.5%)	10% (−4.8% to 24.9%)	14.2% (−1% to 29.3%)	10.3% (−4.6% to 25.2%)	14.3% (−2.4% to 30.9%)	11.1% (−4% to 26.3%)
Eggs	18% (2.3% to 33.7%)	16.3% (0.5% to 32.1%)	20.4% (4.4% to 36.5%)	16.5% (0.7% to 32.3%)	20.5% (3% to 38%)	17.4% (1.3% to 33.4%)
Vegetables	0% (−3.7% to 3.7%)	−1.7% (−5.8% to 2.5%)	2.5% (−2.6% to 7.5%)	−1.4% (−5.6% to 2.7%)	2.5% (−6.1% to 11.2%)	−0.6% (−5.6% to 4.4%)
Fruits	−1.7% (−4.6% to 1.1%)	−0.03% (−6.9% to 0%)	0% (−3.8% to 5.2%)	−3.2% (−6.6% to 0.3%)	0.8% (−7.5% to 9.1%)	−2.3% (−6.8% to 2.1%)
CERAD praxis items						
Meat	4.9% (−7.2% to 17%)	4.8% (−7.7% to 17.2%)	4.9% (−7.7% to 17.4%)	3.2% (−9.1% to 15.5%)	−1.7% (−15.7% to 12.3%)	3.5% (−9.1% to 16.1%)
Fish	0.7% (−16.1% to 17.5%)	0.5% (−16.5% to 17.6%)	0.6% (−16.5% to 17.8%)	−1% (−18% to 15.9%)	−5.9% (−24.1% to 12.3%)	−0.7% (−17.9% to 16.4%)
Eggs	−0.7% (−33.7% to 32.3%)	−0.9% (−34% to 32.3%)	−0.8% (−34% to 32.4%)	−2.4% (−35.5% to 30.7%)	−7.3% (−41.1% to 26.4%)	−2.1% (−35.3% to 31.1%)
Vegetables	−1.5% (−5.2% to 2.2%)	−1.7% (−6.4% to 3%)	−1.6% (−6.5% to 3.4%)	−3.2% (−7.5% to 1.1%)	−8.1% (−16% to −0.3%)	−2.9% (−8% to 2.2%)
Fruits	0.5% (−2.2% to 3.1%)	0.3% (−3.6% to 4.3%)	0.4% (−3.7% to 4.6%)	−1.2% (−4.6% to 2.2%)	−6.2% (−13.6% to 1.3%)	−0.9% (−5.3% to 3.4%)

### 3.3 Sensitivity analysis

We included 838 participants (64% women) in the sensitivity analysis. The mean age was 67.3 years old at baseline and they were followed-up for a mean of 9.7 years. The effects of adding 100 g dairy/d to the diet followed the same trends as in the main analysis. The sensitivity analysis confirmed the results of the addition effects on CDR and showed trends in the same direction for total dairy and the subtypes. We found a 1.5% (−0.5% to 3.5%) risk increase of CDR after adding any type of dairy to the diet, and equal trends for subtypes as in the main analysis. For the secondary outcomes, the risk differences were of small magnitude and non-informative across outcomes and subtypes of dairy product (Supplementary Table 4). Estimates obtained from the linear mixed effects models were equally of small magnitude (Supplementary Table 6).

The substitution effects were consistent with the main analysis. For our primary outcome, there was an increased probability of 9.1% (3.2%–14.9%) of cognitive impairment related to the substitution of vegetables with total dairy and subtypes, as observed in the main analysis. Similar trends were found across secondary outcomes for the substitution of fish and eggs with dairy. The substitution for fish with total dairy was detrimental for the CERAD praxis items [26.6% (−5.2% to 58.4%)], as in the main analysis but beneficial for the DO40 test [−17.3% (−34.2% to 0.3%)]. Similarly, the substitution for eggs with total dairy was beneficial for the Stroop test [−50.9% (−100.9% to −1%)], as in the main analysis. The other effect estimates for these foods were too imprecise to be informative. The effect estimates related to the substitution of meat, vegetables and fruits with dairy had similar non-informative and very small magnitudes (Supplementary Table 5). Conducting an analysis adjusting for competing events due to death was not relevant because there were 15 deaths (<1%) in our analysis sample.

## 4 Discussion

Our study suggested that adding 100 g/d dairy to the diet had no effect on cognitive function among older adults. There was a negligible and imprecise effect of increasing total and any subtype of dairy consumption in the main analysis that we could confirm in the longer-term follow-up sensitivity analysis. Estimates were overall in the same direction pointing towards a negative effect of increasing dairy consumption. However, the effects were too imprecise to conclude anything. The substitution effects followed the same trends for meat, fruit and vegetables, for which we observed small and imprecise substitution effects across outcomes for all subtypes of dairy products. The substitution of 100 g of fish or eggs/d for 100 g of any subtype of dairy products/d led to a higher risk of having a lower score in some cognitive function measures, but not for CDR. The effects in the main analysis were consistent with the sensitivity analysis, with a few effect estimates that increased in magnitude but also in imprecision. Therefore, this study has some clinical implications. Since the magnitude of the effect of adding dairy was clinically irrelevant, we may not recommend increasing the amount of dairy consumed by participants. The effect of substitution dairy were imprecise and confidence intervals were non-informative. Thus, we cannot rule out the presence of a clinically relevant effect. Comparing results across studies is challenging due to the different outcomes assessed and the diverse statistical approaches and interpretations. First, the effects of adding dairy on top of the baseline diet were targeted implicitly by all the studies, none included remaining energy adjustment. Most studies reported associations between total dairy and the MMSE or clinical diagnosis codes (e.g., DSM5). Consequently, we focused our comparison on the direction of the estimates given that we used very different adjustment sets and energy adjustment approach. Among the 13 studies that explored the association between total dairy and cognitive function, seven reported protective effects [13,[43], [44], [45], [46], [47], [48]] of which four presented imprecise estimates [13,[44], [45], [46]], three harmful but imprecise effects [[49], [50], [51]] and three null effects [[52], [53], [54]]. The studies reporting positive effects of dairy consumption on the incidence of MCI or dementia interpreted coefficients from a model built-in for multiple exposures. Thus, these studies missed important confounders [43,48]. Studies without energy adjustment also found more often protective effects [43,45,48], possibly because they estimated the effect of the increase in dairy consumption together with the increase in total calorie intake and residual confounding. This might lead to the conclusion that those with higher consumption and higher calorie intake have better cognition, as well described in studies in the older adult subpopulation. However, among studies that selected a similar set of covariates as ours, we found similar results even if they had different cognitive outcomes or analytic approaches [13,44,46,50,52,53], reporting null or imprecise associations.

No studies examined explicit addition effects, but we are in line with studies finding null effects even though they adjusted for total calories. Second, no previous study targeted the effect of substituting foods and therefore, we could not compare our estimates to previous work and compare the potentially harmful effect of substituting fish and eggs for dairy. There were some studies reporting a positive effect of fish on cognition [12,13], thus supporting the no substitution of this food group. However, we found no consistent positive effects of eggs supporting non substituting them for dairy products. These results could be relevant and against recommending dairy as an alternative protein source to fish. While the literature found positive effects of vegetables and fruits [[7], [8], [9]], our study showed that possibly they did not differ significantly from the effect of dairy, given that the effect of the substitution is very small. This could be due to the elevated content of water in fruits and vegetables that plays a role when we report our findings in g/day. Surprisingly, we found no differences between different dairy subtypes even if they are a very heterogenous food group.

Our study addressed some of the limitations of previous studies. We included a comprehensive set of cognitive function measures evaluating not only memory but also verbal fluency, motor capacity and executive function. Similarly, we incorporated also dairy subtypes in our assessment because there was scarce literature and dairy is a very diverse group. This overcame a main weakness of past studies using only the MMSE or clinical diagnosis codes (e.g., ICD8, 9, 10 or DSM-III). To our knowledge, this was the first study reporting the substitution effects of other foods groups (e.g., meat, fish, vegetables) for dairy on cognitive function. We did not observe any relevant nor consistent differences in cognitive function for any substitution for total dairy or subtypes. We highlight the commitment to a causal framework that is explicit in the targeted parameters estimands and in the assumptions they entailed.

To confirm our results, we would need to address five main limitations. First, the generalizability of our study to our target population is limited. From the initial CoLaus sample, 4605 participants (68%) answered the FFQ at the first follow-up, and 3719 participants accepted (67%) to have a baseline cognitive assessment in CoLaus|PsyCoLaus. Consequently, our study sample might not represent the initial target sample from CoLaus|PsyColaus given that the intersection of both selections resulted in 1436 eligible participants (20% of the initial sample). Second, nutrition exposure assessment through FFQ could be affected by measurement bias and considering that we used continuous measures for dietary intake when they are suited to assess levels of intake rather than continuous quantities, hindering consistency. Our sample was restricted to healthy participants at baseline when the FFQs were completed, so we argue that the measurement bias in the exposure is non-differential, thus biasing the estimate towards the null. Third, we could not exclude violation of exchangeability assumption for residual confounding, even though we included all the relevant variables based on past studies and expert knowledge. Fourth, IPTW estimator might be misspecified. It considered linear relationships and a gaussian distribution as the probability density function, while there might be non-linear interactions, for example between dairy consumption and the cognitive function outcomes. It also assumes no measurement error on the exposure (outcome of the model to estimate the weights). Lastly, the addition and substitution effects estimands may not be not fully consistent with our observed data [55] because an individual will likely change other food groups intake if we were to intervene adding dairy products, while we assumed they remained constant. The same would apply to substitution effects assuming that participants would only exchange of the two food groups after the substitution without involving any switch in other food groups.

Overall, we cannot transport our results outside the exposure and outcome ranges observed in our data and to non-Swiss populations. Even though our covered a wide exposure range for total dairy intake, low-fat and sugary dairy intake ranges were narrow. For the outcome, there were few cognitive impaired participants, so our results only apply to a relatively healthy cognitive population of older adults, and consequently, we selected the 75th percentile to dichotomize the outcomes instead of clinical cut-offs or the medians. Transportability to non-Swiss populations is also limited because diets are highly dependent on geographical variation, where different combinations of dairy with other food groups may have different effects on cognition. We believe that further studies making explicit food substitutions would be valuable to confirm our results. Because different populations may substitute dairy products with different foods and this leads to opposing results in the different studies, we could mitigate this by modeling explicit substitutions. In addition, the compositional nature of the diet requires more studies examining dietary patterns instead of specific food groups.

## 5 Conclusion

We found that neither adding dairy to the diet nor substituting different food groups for dairy had a consistent effect on cognitive function. The substitution of fish and eggs for dairy products could negatively impact some cognitive functions but not overall cognitive functioning. Further studies need to evaluate substitution effects in populations with a wider range of sub-types of dairy intake, more variability in the cognitive function and with a larger sample size to increase the power to detect smaller effects and make less strong causal identification assumptions.

## Funding

This study and NO are funded by the SNF-project grant n° 204967 “Prospective international study of dairy and inflammation on cognitive decline” (PI: Patricia Chocano-Bedoya). The CoLaus|PsyCoLaus study was supported by unrestricted research grants from 10.13039/100004330GlaxoSmithKline, the Faculty of Biology and Medicine of Lausanne, the 10.13039/501100001711Swiss National Science Foundation (grants 3200B0–105993, 3200B0-118308, 33CSCO-122661, 33CS30-139468, 33CS30-148401, 33CS30_177535 and 3247730_204523) and the Swiss Personalized Health Network (grant 2018DRI01).

## Conflict of interest statement

The authors declared no conflict of interest to disclose.

## Data availability statement

The data of CoLaus|PsyCoLaus study used in this article cannot be fully shared as they contain potentially sensitive personal information on participants. According to the Ethics Committee for Research of the Canton of Vaud, sharing these data would be a violation of the Swiss legislation with respect to privacy protection. However, coded individual-level data that do not allow researchers to identify participants are available upon request to researchers who meet the criteria for data sharing of the CoLaus|PsyCoLaus Datacenter (CHUV, Lausanne, Switzerland). Any researcher affiliated to a public or private research institution who complies with the CoLaus|PsyCoLaus standards can submit a research application to research.colaus@chuv.ch or research.psycolaus@chuv.ch. Proposals requiring baseline data only, will be evaluated by the baseline (local) Scientific Committee (SC) of the CoLaus and PsyCoLaus studies. Proposals requiring follow-up data will be evaluated by the follow-up (multicentric) SC of the CoLaus|PsyCoLaus cohort study. Detailed instructions for gaining access to the CoLaus|PsyCoLaus data used in this study are available at www.colaus-psycolaus.ch/professionals/how-to-collaborate/. We provide detailed code and pooled results in Supplementary File 2.

## Author contributions

Conception and design: PCB, NO; Data analysis: NO; Interpretation of the results: NO, PCB, AC, CC, PMV, OE, AvG, MP, NR, JHB; Drafting of the article: NO; Statistical methods advice: OE, CC; Final approval of the article: AC, MV, CC, PMV, OE, AvG, MP, PCB, NR, JHB, LZ, JV, PV ; Provision of study data: PMV, AVG, MP, LZ, JV, PV.
