# Supplementary material for: Effect of dairy consumption on cognition in older adults: A population-based cohort study
Source: J Nutr Health Aging. 2024 Jan 1;28(2):100031. doi: 10.1016/j.jnha.2023.100031 (PMC12877266; doi:10.1016/j.jnha.2023.100031)
Supplement: Supplementary file 1 [file mmc1.docx]

**Supplementary Table 1**. Baseline characteristics table with balanced covariates after inverse probability weighting for the continuous total dairy exposure. SMD: Standardized mean difference, IPW: Inverse Probability Weights, BMI: Body mass index.

|  | **SMD baseline** | **SMD after IPW** |
| --- | --- | --- |
| Sex | 0.061 | 0.055 |
| Age | 0.026 | 0.039 |
| Education | 0.035 | 0.058 |
| Occupation | 0.156 | 0.070 |
| BMI | 0.060 | 0.055 |
| Smoking | 0.096 | 0.051 |
| Past cardiovascular event | 0.084 | 0.049 |
| Hypertension | 0.056 | 0.010 |
| Alcohol consumption | 0.0003 | 0.0001 |
| Depression | 0.166 | 0.022 |
| Family income | 0.069 | 0.012 |
| Diabetes | 0.012 | 0.078 |
| Physical activity | 0.080 | 0.075 |

**Supplementary Table 2**. Range (quartiles) of dairy consumption (g/d) among PsyCoLaus|CoLaus participants.

|  | Q1 | Q2 | Q3 | Q4 |
| --- | --- | --- | --- | --- |
| Total dairy | [0, 119) | [199, 208) | [208, 307) | [307, 1698) |
| Fermented dairy | [0, 67) | [67, 128) | [128, 214) | [214, 1652) |
| Non-fermented dairy | [0, 16) | [16, 45) | [45, 104) | [104, 965) |
| Full fat dairy | [0, 94) | [94, 172) | [172, 268) | [268, 1419) |
| Low fat dairy | [0-0) | [0-0) | [0, 39) | [39, 932) |
| Sugary dairy | [0, 8) | [8, 32) | [32, 90) | [90, 712) |

**Supplementary Table 3**. Differences in baseline characteristics between the initial CoLaus|PsyColaus subset (n=1,745) and the participants who were included in the analyses (not lost-to-follow-up) (n=1,347). SMD: Standardized Mean Difference.

|  | **No** | **Yes** | **SMD** |
| --- | --- | --- | --- |
| n | 412 | 1334 |  |
| Sex (Male) (%) | 233 (56.6) | 507 (38.0) | 0.378 |
| Age |  |  | 0.32 |
| Less than 70 years old | 215 (52.2) | 850 (63.7) |  |
| 70 to 75 years old | 88 (21.4) | 297 (22.3) |  |
| Older than 75 | 109 (26.5) | 187 (14.0) |  |
| Education (%) |  |  | 0.1 |
| Elementary | 295 (71.6) | 895 (67.1) |  |
| High school | 60 (14.6) | 218 (16.3) |  |
| Superior | 57 (13.8) | 221 (16.6) |  |
| Occupational position (%) |  |  | 0.03 |
| High | 21 ( 5.2) | 64 ( 4.9) |  |
| Middle | 48 (11.8) | 144 (11.0) |  |
| Low | 48 (11.8) | 160 (12.3) |  |
| Not working | 289 (71.2) | 937 (71.8) |  |
| BMI (%) |  |  | 0.199 |
| Normal | 135 (33.3) | 529 (40.0) |  |
| Overweight | 167 (41.1) | 554 (41.9) |  |
| Obese | 104 (25.6) | 238 (18.0) |  |
| Smoking habits (%) |  |  | 0.214 |
| Never | 140 (34.2) | 590 (44.3) |  |
| Former | 195 (47.7) | 560 (42.0) |  |
| Current | 74 (18.1) | 182 (13.7) |  |
| CV event - Yes (%) | 108 (26.3) | 245 (18.4) | 0.19 |
| Hypertension - Yes (%) | 310 (75.2) | 857 (64.3) | 0.239 |
| Alcohol consumption (%) |  |  | 0.011 |
| Non-drinkers | 94 (25.5) | 316 (25.9) |  |
| Light drinkers | 176 (47.8) | 578 (47.3) |  |
| Moderate and  heavy drinkers | 98 (26.6) | 328 (26.8) |  |
| Depression - Yes (%) | 53 (14.3) | 137 (11.3) | 0.09 |
| Total calories (mean (SD)) | 1683 (584) | 1687 (559) | 0.007 |
| Family income (%) |  |  | 0.211 |
| Lowest tertile | 38 (43.7) | 406 (39.1) |  |
| Middle tertile | 42 (48.3) | 481 (46.3) |  |
| Highest tertile | 7 ( 8.0) | 152 (14.6) |  |
| Diabetes - Yes (%) | 74 (18.0) | 132 ( 9.9) | 0.235 |
| Physical activity (%) |  |  | 0.094 |
| High | 77 (22.0) | 216 (18.4) |  |
| Low | 151 (43.1) | 545 (46.3) |  |
| Medium | 122 (34.9) | 416 (35.3) |  |

**Supplementary Figure 1.** Detailed Directed Acyclic Graph (DAG) of all the hypothesized interdependencies between our measured covariates. CV: cardiovascular


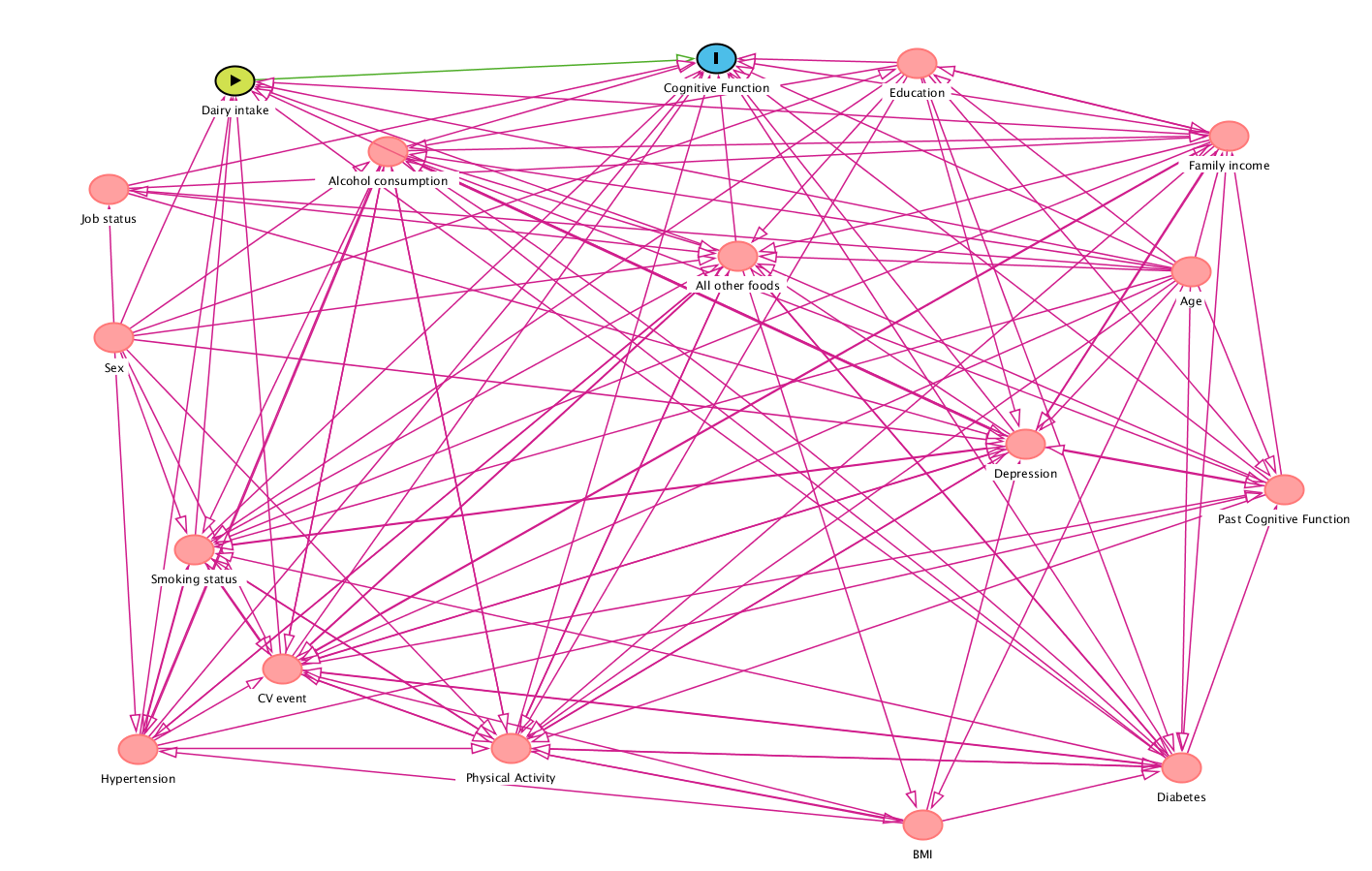


**Supplementary Table 4**. Sensitivity analysis. Average causal effect estimates of adding 100 g of dairy per day to the baseline diet. CDR: Clinical dementia rating, SCD: Subjective cognitive decline.

|  | **CDR** | **SCD** | **Memory** | **Verbal fluency** | **Stroop** | **DO40** | **CERAD** |
| --- | --- | --- | --- | --- | --- | --- | --- |
| **Total dairy** | 1.52% (-0.49% to 3.52%) | 1.27% (-0.82% to 3.36%) | 1.97% (-0.2% to 4.14%) | 0.57% (-2.37% to 3.52%) | 0.62% (-2.73% to 3.97%) | 0.69% (-1.6% to 2.98%) | -0.81% (-4.4% to 2.78%) |
| **Fermented dairy** | 1.1% (-1.79% to 3.99%) | 1.81% (-1.52% to 5.14%) | 2.62% (-0.55% to 5.79%) | -1.38% (-5.84% to 3.08%) | -0.23% (-4.68% to 4.23%) | 0.12% (-3.34% to 3.58%) | -1.23% (-6.35% to 3.89%) |
| **Non fermented dairy** | 0.1% (-3.57% to 3.78%) | 0.38% (-3.82% to 4.59%) | 1.92% (-0.68% to 4.51%) | 1.2% (-3.81% to 6.21%) | -2.04% (-6.93% to 2.85%) | 1.06% (-2.7% to 4.83%) | -3.05% (-8.43% to 2.33%) |
| **Full fat dairy** | 2.35% (0.08% to 4.63%) | 1.96% (-0.54% to 4.47%) | 2.09% (-0.04% to 4.23%) | -0.83% (-4.28% to 2.62%) | 0.52% (-3.2% to 4.23%) | -0.29% (-3.81% to 3.22%) | -2.46% (-6.95% to 2.04%) |
| **Low fat dairy** | -7.85% (-16.09% to 0.4%) | 0.38% (-3.82% to 4.59%) | 5.91% (-4.16% to 15.98%) | 1.2% (-3.81% to 6.21%) | -2.04% (-6.93% to 2.85%) | 0.01% (-4.57% to 4.59%) | -3.05% (-8.43% to 2.33%) |
| **Sugary dairy** | 4.4% (0.76% to 8.03%) | 3.37% (-2.15% to 8.88%) | 1.23% (-3.02% to 5.47%) | -6.71% (-16.08% to 2.65%) | -0.96% (-13.04% to 11.12%) | -14.06% (-33.9% to 5.77%) | 9.39% (-4.09% to 22.86%) |

**Supplementary Table 5**. Sensitivity analysis. Average Causal Effect of substitution estimates for cognition outcomes of substituting 100 g of dairy for a different food group. CDR: Clinical dementia rating, SCD: Subjective cognitive decline.

|  | **CDR** | | | | | |
| --- | --- | --- | --- | --- | --- | --- |
| **Meat** | 0.5% (-14.8% to 15.8%) | -0.3% (-15.9% to 15.4%) | -0.01% (-16.7% to 15.3%) | -1.7% (-17.2% to 13.8%) | 7.3% (-9.1% to 23.7%) | -1% (-18.2% to 16.2%) |
| **Fish** | 11.4% (-20% to 42.7%) | 10.6% (-20.9% to 42.2%) | 10.2% (-21.5% to 41.9%) | 9.3% (-22.2% to 40.7%) | 18.2% (-13.7% to 50.2%) | 9.9% (-22.4% to 42.2%) |
| **Eggs** | -9.2% (-63.7% to 45.3%) | -10% (-64.6% to 44.6%) | -10.4% (-65.1% to 44.3%) | -11.4% (-65.9% to 43.2%) | -2.4% (-57.2% to 52.5%) | -10.7% (-65.8% to 44.4%) |
| **Vegetables** | 9.1% ( 3.2% to 14.9%) | 8.3% ( 1.6% to 15%) | 7.9% ( 0.4% to 15.4%) | 6.9% ( 0.6% to 13.3%) | 15.9% ( 7.6% to 24.3%) | 7.6% (-2.3% to 17.4%) |
| **Fruits** | 1.2% (-4.2% to 6.5%) | 0.4% (-5.9% to 6.7%) | 0% (-7.1% to 7.1%) | -0.9% (-6.9% to 5%) | 8% ( 0% to 16%) | -0.3% (-9.8% to 9.3%) |
|  | **SCD** | | | | | |
| **Meat** | -0.7% (-11.4% to 10.1%) | -0.2% (-11.2% to 10.8%) | -1.8% (-13.2% to 9.6%) | -0.2% (-11% to 10.6%) | -1.9% (-13.9% to 10.2%) | -0.6% (-12.1% to 11%) |
| **Fish** | -8.8% (-29.1% to 11.4%) | -8.4% (-28.8% to 12%) | -9.9% (-30.5% to 10.6%) | -8.3% (-28.6% to 11.9%) | -10% (-31% to 10.9%) | -8.7% (-29.4% to 11.9%) |
| **Eggs** | -8% (-41.3% to 25.4%) | -7.5% (-41% to 25.9%) | -9.1% (-42.7% to 24.5%) | -7.5% (-40.9% to 25.9%) | -9.2% (-43% to 24.6%) | -7.9% (-41.5% to 25.8%) |
| **Vegetables** | 1% (-3.9% to 5.9%) | 1.4% (-4% to 6.9%) | -0.1% (-6.3% to 6%) | 1.4% (-3.6% to 6.5%) | -0.2% (-7.6% to 7.1%) | 1.1% (-5.4% to 7.5%) |
| **Fruits** | 3.4% ( 1% to 5.8%) | 3.8% ( 0.5% to 7.2%) | 2.3% (-2.2% to 6.7%) | 3.9% ( 1.2% to 6.5%) | 2.2% (-3.8% to 8.2%) | 3.5% (-1.3% to 8.3%) |
|  | **Memory** | | | | | |
| **Meat** | -2.5% (-12.2% to 7.2%) | -1.7% (-11.6% to 8.2%) | -5.9% (-16.1% to 4.4%) | -3% (-12.9% to 6.9%) | -6.6% (-17.3% to 4.1%) | 1.7% (-9.7% to 13.2%) |
| **Fish** | 3% (-14.4% to 20.4%) | 3.8% (-13.7% to 21.4%) | -0.3% (-18.1% to 17.4%) | 2.5% (-15% to 20.1%) | -1.1% (-19% to 16.9%) | 7.2% (-11.2% to 25.7%) |
| **Eggs** | 18.4% (-18.1% to 54.8%) | 19.2% (-17.3% to 55.7%) | 15% (-21.6% to 51.6%) | 17.9% (-18.6% to 54.4%) | 14.3% (-22.4% to 51%) | 22.6% (-14.4% to 59.5%) |
| **Vegetables** | 4% (-0.3% to 8.3%) | 4.8% ( 0.1% to 9.5%) | 0.6% (-4.8% to 6.1%) | 3.5% (-1.2% to 8.2%) | -0.1% (-6.2% to 6.1%) | 8.2% (0.8% to 15.6%) |
| **Fruits** | 3.1% ( 0.4% to 5.8%) | 3.9% ( 0.6% to 7.3%) | -0.2% (-4.5% to 4%) | 2.6% (-0.7% to 5.9%) | -1% (-6.2% to 4.2%) | 7.3% (0.7% to 13.9%) |
|  | **Verbal fluency** | | | | | |
| **Meat** | 0.7% (-13.9% to 15.2%) | -1.5% (-16.3% to 13.4%) | 2.9% (-12.3% to 18.1%) | -0.9% (-15.6% to 13.8%) | 1.9% (-15% to 18.7%) | -0.8% (-17.3% to 15.7%) |
| **Fish** | 0.6% (-30.7% to 31.8%) | -1.6% (-33% to 29.9%) | 2.7% (-28.8% to 34.3%) | -1% (-32.3% to 30.3%) | 1.8% (-30.7% to 34.2%) | -0.9% (-33.2% to 31.3%) |
| **Eggs** | 25.3% (-24.7% to 75.3%) | 23.2% (-26.9% to 73.3%) | 27.5% (-22.7% to 77.7%) | 23.8% (-26.3% to 73.8%) | 26.5% (-24.2% to 77.3%) | 23.8% (-26.8% to 74.5%) |
| **Vegetables** | -2.9% (-9% to 3.2%) | -5.1% (-12% to 1.8%) | -0.8% (-8.3% to 6.8%) | -4.5% (-10.9% to 1.9%) | -1.7% (-12.2% to 8.7%) | -4.4% (-14.4% to 5.5%) |
| **Fruits** | 2.8% (-2.2% to 7.8%) | 0.7% (-5.2% to 6.6%) | 5% (-1.6% to 11.7%) | 1.3% (-4.1% to 6.6%) | 4% (-5.8% to 13.9%) | 1.3% (-8% to 10.7%) |
|  | **Stroop** | | | | | |
| **Meat** | -0.5% (-15.3% to 14.3%) | -1.7% (-16.7% to 13.4%) | -0.1% (-6.3% to 6%) | -0.8% (-15.7% to 14.1%) | 0.6% (-15.7% to 16.9%) | -2.3% (-18.2% to 13.7%) |
| **Fish** | 2.9% (-24.9% to 30.6%) | 1.7% (-26.2% to 29.6%) | 0.7% (-27.3% to 28.7%) | 2.6% (-25.2% to 30.4%) | 4% (-24.6% to 32.6%) | 1.1% (-27.3% to 29.5%) |
| **Eggs** | -50.9% (-100.9% to -1%) | -52.1% (-102.1% to -2.1%) | -53.1% (-103.2% to -3%) | -51.2% (-101.2% to -1.3%) | -49.8% (-100.2% to 0.6%) | -52.7% (-103% to -2.4%) |
| **Vegetables** | -0.2% (-5.7% to 5.3%) | -1.4% (-7.5% to 4.7%) | -2.4% (-9.1% to 4.3%) | -0.5% (-6.2% to 5.2%) | 0.9% (-7.8% to 9.7%) | -2% (-10.1% to 6.1%) |
| **Fruits** | 0% (-4.1% to 4.2%) | -1.1% (-6.1% to 3.8%) | -2.1% (-7.8% to 3.6%) | -0.3% (-4.7% to 4.2%) | 1.2% (-6.8% to 9.2%) | -1.7% (-9% to 5.5%) |
|  | **DO40** | | | | | |
| **Meat** | 12.3% (-1.7% to 26.3%) | 11.7% (-2.5% to 25.9%) | 12.9% (-1.5% to 27.2%) | 11.4% (-2.9% to 25.6%) | 12% (-2.7% to 26.6%) | 11.9% (-3.5% to 27.3%) |
| **Fish** | -17.3% (-34.2% to -0.3%) | -17.9% (-35% to -0.7%) | -16.7% (-33.9% to 0.5%) | -18.2% (-35.3% to -1.1%) | -17.6% (-35% to -0.2%) | -17.6% (-35.8% to 0.5%) |
| **Eggs** | 25.6% (-12.1% to 63.4%) | 25% (-12.8% to 62.8%) | 26.2% (-11.7% to 64.1%) | 24.7% (-13.2% to 62.5%) | 25.3% (-12.7% to 63.2%) | 25.2% (-13.1% to 63.5%) |
| **Vegetables** | 0.6% (-3.6% to 4.8%) | 0% (-4.8% to 4.9%) | 1.2% (-4% to 6.4%) | -0.3% (-5.1% to 4.5%) | 0.3% (-5.7% to 6.2%) | 0.3% (-7.5% to 8%) |
| **Fruits** | 1.4% (-1.1% to 3.9%) | 0.9% (-2.6% to 4.4%) | 2% (-1.9% to 6%) | 0.01% (-2.9% to 3.9%) | 1.1% (-3.8% to 6%) | 1.1% (-5.8% to 8%) |
|  | **CERAD praxis items** | | | | | |
| **Meat** | -0.4% (-15.6% to 14.8%) | -0.8% (-16.3% to 14.7%) | -1.7% (-17.4% to 14.1%) | -1.1% (-16.4% to 14.3%) | 6.7% (-9.7% to 23.1%) | 7.6% (-8.9% to 24.2%) |
| **Fish** | 26.6% (-5.2% to 58.4%) | 26.2% (-5.8% to 58.2%) | 25.3% (-6.8% to 57.4%) | 25.9% (-6% to 57.8%) | 33.7% ( 1.3% to 66.1%) | 34.6% ( 2.1% to 67.1%) |
| **Eggs** | -21.6% (-74% to 30.9%) | -22% (-74.5% to 30.6%) | -22.9% (-75.5% to 29.8%) | -22.3% (-74.7% to 30.2%) | -14.5% (-67.3% to 38.3%) | -13.6% (-66.4% to 39.3%) |
| **Vegetables** | -1.3% (-8.3% to 5.7%) | -1.7% (-9.5% to 6%) | -2.6% (-10.8% to 5.7%) | -2% (-9.3% to 5.4%) | 5.8% (-3.6% to 15.2%) | 6.7% (-3% to 16.4%) |
| **Fruits** | 0.1% (-5% to 5.1%) | -0.3% (-6.4% to 5.7%) | -1.2% (-7.8% to 5.4%) | -0.6% (-6.1% to 4.9%) | 7.2% (-0.8% to 15.2%) | 8.1% (-0.2% to 16.4%) |

**Supplementary Table 6. Mean difference for total dairy consumption (100g/d) in the linear mixed effects models for the different cognitive domains.**

|  | Fixed effects (95% CI) |
| --- | --- |
| CDR (n=1,276) | 0.01 (-0.03, 0.02) |
| Time | -0.02 (-0.04,0.01) |
| SCD (n=1,301) | 0.005 (-0.005, 0.015) |
| Time | -0.01 (-0.03, 0.01) |
| Memory (n=1,265) | -0.0459 (-0.217, 0.125) |
| Time | 1.51 (1.15,1.87) |
| Verbal function (n= 1,269) | -0.165 (-0.395, 0.065) |
| Time | -0.09 (-0.40,0.23) |
| Stroop (n=1,296) | 0.004 (-0.043, 0.044) |
| Time | 0.001 (-0.08,0.09) |
| CERAD praxis items (n=1,306) | -0.00007 (-0.031,0.031) |
| Time | -0.26 (-0.32,-0.20) |
| D040 | 0.004 (-0.024, 0.032) |
| Time | -0.08 (-0.14, -0.02) |
