# Supplementary material for: Effect of dairy consumption on cognition in older adults: A population-based cohort study
Source: J Nutr Health Aging. 2024 Jan 1;28(2):100031. doi: 10.1016/j.jnha.2023.100031 (PMC12877266; doi:10.1016/j.jnha.2023.100031)
Supplement: Supplementary file 2 [file mmc2.pdf]

Dairy and Cognition. Addition effects

Author: Natalia Ortega

Last updated: 20.08.2023

Background

The current analysis is part of the project "Prospective international study on dairy and inflammation on cognitive decline". We will use PsyCoLaus data to answer the 1st goal which main aim is to assess the long-term effect of total dairy intake and subtypes of dairy (fermented vs. non-fermented, low vs. full fat) on cognitive function. This is the first file of two, here computing addition effects (total effects).

Contents

1. Loading packages and datasets
2. Covariate coding
3. Selection criteria
4. Data description
   4.1 Outcome coding and viz
   4.2 Exposure coding and viz
   4.3 Table 1
5. Missingness
6. IPTW and MSMs
7. Sensitivy analysis for total effects

The details on each method are available in SAP_Aim1.docx version 1.0 (Not approved version).

1. Loading packages and datasets

Packages

In [41]:
```
library(tidyverse)
library(MASS)
library(ggpubr)
library(tableone)
library(gridExtra)
library(rlang)
library(geepack)
library(naniar)
library(WeightIt)
library(cobalt)
library(survey)
#Ignore or not warnings
```

```
options(warn=-1)
dodge = position_dodge(width=0.1)
```

Datasets

In [42]:

```
baseline <- read.csv ("C:/Users/no22t395/OneDrive - Universitaet Bern/CoLaus doc/C(
fu1 <- read.csv ("C:/Users/no22t395/OneDrive - Universitaet Bern/CoLaus doc/COLAUS
fu2 <- read.csv ("C:/Users/no22t395/OneDrive - Universitaet Bern/CoLaus doc/COLAUS
fu3 <- read.csv ("C:/Users/no22t395/OneDrive - Universitaet Bern/CoLaus doc/COLAUS
cog <- read.csv ("C:/Users/no22t395/OneDrive - Universitaet Bern/CoLaus doc/COLAUS
```

2. Covariate coding

In [43]:

```
# Merge datasets by ID (pt)

ch <- dplyr::full_join(baseline, fu1, by = c("pt"))
ch <- ch %>%
    dplyr::full_join(fu2, by = c("pt")) %>%
    dplyr::full_join(fu3, by = c("pt")) %>%
    dplyr::full_join(cog, by = c("pt"))

summary(ch)
```

```
      pt              sex             datbirth            datexam         
 Min.   :   1   Min.   :0.0000   Length:6746        Length:6746       
 1st Qu.:1815   1st Qu.:0.0000   Class :character   Class :character  
 Median :3638   Median :0.0000   Mode  :character   Mode  :character  
 Mean   :3842   Mean   :0.4736                                        
 3rd Qu.:5500   3rd Qu.:1.0000                                        
 Max.   :9660   Max.   :1.0000                                        
                NA's   :13                                            
  bthpl_dem         ethori_self            edtyp            edlv      
 Length:6746        Length:6746        Min.   :0.000   Min.   : 0.00  
 Class :character   Class :character   1st Qu.:1.000   1st Qu.:10.00  
 Mode  :character   Mode  :character   Median :1.000   Median :13.00  
                                       Mean   :1.755   Mean   :13.11  
                                       3rd Qu.:3.000   3rd Qu.:16.00  
                                       Max.   :4.000   Max.   :57.00  
                                       NA's   :27      NA's   :41     
     mrtsts           sclhlp        job_curr1        job_curr4a    
 Min.   :0.0000   Min.   :0.000   Min.   :0.0000   Min.   :0.0000  
 1st Qu.:0.0000   1st Qu.:0.000   1st Qu.:0.0000   1st Qu.:0.0000  
 Median :0.0000   Median :0.000   Median :1.0000   Median :0.0000  
 Mean   :0.6419   Mean   :0.285   Mean   :0.6772   Mean   :0.7041  
 3rd Qu.:2.0000   3rd Qu.:0.000   3rd Qu.:1.0000   3rd Qu.:2.0000  
 Max.   :3.0000   Max.   :9.000   Max.   :1.0000   Max.   :2.0000  
 NA's   :1103     NA's   :20      NA's   :20       NA's   :20      
   job_curr8          cmp                 hdc                hdv         
 Min.   :1.000   Min.   :0.000000   Min.   :0.00000   Min.   :0.00000  
 1st Qu.:2.000   1st Qu.:0.000000   1st Qu.:0.00000   1st Qu.:0.00000  
 Median :2.000   Median :0.000000   Median :0.00000   Median :0.00000  
 Mean   :4.102   Mean   :0.005051   Mean   :0.01486   Mean   :0.04516  
 3rd Qu.:9.000   3rd Qu.:0.000000   3rd Qu.:0.00000   3rd Qu.:0.00000  
 Max.   :9.000   Max.   :9.000000   Max.   :9.00000   Max.   :9.00000  
 NA's   :30      NA's   :15         NA's   :15        NA's   :15       
      chf               artm               cad               angn        
 Min.   :0.00000   Min.   :0.00000   Min.   :0.0000   Min.   :0.00000  
 1st Qu.:0.00000   1st Qu.:0.00000   1st Qu.:0.0000   1st Qu.:0.00000  
 Median :0.00000   Median :0.00000   Median :0.0000   Median :0.00000  
 Mean   :0.01515   Mean   :0.07815   Mean   :0.0257   Mean   :0.02273  
 3rd Qu.:0.00000   3rd Qu.:0.00000   3rd Qu.:0.0000   3rd Qu.:0.00000  
 Max.   :9.00000   Max.   :9.00000   Max.   :9.0000   Max.   :9.00000  
 NA's   :15        NA's   :15        NA's   :15       NA's   :15       
      miac              strk               vslg               ccth        
 Min.   :0.00000   Min.   :0.00000   Min.   :0.00000   Min.   :0.00000  
 1st Qu.:0.00000   1st Qu.:0.00000   1st Qu.:0.00000   1st Qu.:0.00000  
 Median :0.00000   Median :0.00000   Median :0.00000   Median :0.00000  
 Mean   :0.01946   Mean   :0.01456   Mean   :0.03922   Mean   :0.01902  
 3rd Qu.:0.00000   3rd Qu.:0.00000   3rd Qu.:0.00000   3rd Qu.:0.00000  
 Max.   :9.00000   Max.   :9.00000   Max.   :9.00000   Max.   :9.00000  
 NA's   :15        NA's   :15        NA's   :15        NA's   :15       
      cabg               pcin              cvdbase         cvdbase_adj  
 Min.   :0.000000   Min.   :0.000000   Min.   :0.00000   Min.   :1     
 1st Qu.:0.000000   1st Qu.:0.000000   1st Qu.:0.00000   1st Qu.:1     
 Median :0.000000   Median :0.000000   Median :0.00000   Median :1     
 Mean   :0.009211   Mean   :0.005051   Mean   :0.06048   Mean   :1     
 3rd Qu.:0.000000   3rd Qu.:0.000000   3rd Qu.:0.00000   3rd Qu.:1     
 Max.   :1.000000   Max.   :9.000000   Max.   :1.00000   Max.   :1     
 NA's   :15         NA's   :15         NA's   :17        NA's   :6568  
    alcool1          sbsmk           antiHTA            HTA        
 Min.   :0.000   Min.   :0.0000   Min.   :0.0000   Min.   :0.0000  
 1st Qu.:0.000   1st Qu.:0.0000   1st Qu.:0.0000   1st Qu.:0.0000  
 Median :1.000   Median :1.0000   Median :0.0000   Median :0.0000  
 Mean   :1.386   Mean   :0.8632   Mean   :0.1952   Mean   :0.3717  
 3rd Qu.:2.000   3rd Qu.:2.0000   3rd Qu.:0.0000   3rd Qu.:1.0000  
 Max.   :6.000   Max.   :2.0000   Max.   :1.0000   Max.   :1.0000  
 NA's   :13      NA's   :19       NA's   :13       NA's   :20      
```

```
     mnwlk            phyact           MME            handgrip    
 Min.   :  0.00   Min.   :0.000   Min.   : 0.00   Min.   : 2.27  
 1st Qu.: 15.00   1st Qu.:0.000   1st Qu.:28.00   1st Qu.:24.95  
 Median : 30.00   Median :2.000   Median :29.00   Median :31.75  
 Mean   : 41.26   Mean   :1.296   Mean   :28.58   Mean   :33.42  
 3rd Qu.: 60.00   3rd Qu.:2.000   3rd Qu.:30.00   3rd Qu.:41.28  
 Max.   :720.00   Max.   :9.000   Max.   :30.00   Max.   :77.11  
 NA's   :51       NA's   :107     NA's   :5782    NA's   :3196   
       ht              wt              BMI           BMI_cat1     
 Min.   :131.0   Min.   : 36.90   Min.   :15.55   Min.   :0.0000  
 1st Qu.:162.0   1st Qu.: 62.00   1st Qu.:22.63   1st Qu.:0.0000  
 Median :168.0   Median : 72.00   Median :25.20   Median :1.0000  
 Mean   :168.4   Mean   : 73.34   Mean   :25.80   Mean   :0.6722  
 3rd Qu.:175.0   3rd Qu.: 82.50   3rd Qu.:28.21   3rd Qu.:1.0000  
 Max.   :199.0   Max.   :175.40   Max.   :59.17   Max.   :2.0000  
 NA's   :15      NA's   :16       NA's   :16      NA's   :16      
    BMI_cat2         waist          waist_cat1          hip       
 Min.   :0.000   Min.   : 58.00   Min.   :0.0000   Min.   : 42.0  
 1st Qu.:1.000   1st Qu.: 79.00   1st Qu.:0.0000   1st Qu.: 96.0  
 Median :2.000   Median : 89.00   Median :0.0000   Median :101.0  
 Mean   :1.656   Mean   : 89.06   Mean   :0.2936   Mean   :101.6  
 3rd Qu.:2.000   3rd Qu.: 98.00   3rd Qu.:1.0000   3rd Qu.:106.5  
 Max.   :3.000   Max.   :170.00   Max.   :1.0000   Max.   :170.0  
 NA's   :16      NA's   :20       NA's   :20       NA's   :23     
      WHR             bmpsc            SBP             DBP        
 Min.   :0.5750   Min.   : 0.00   Min.   : 81.5   Min.   : 42.50  
 1st Qu.:0.8132   1st Qu.:23.00   1st Qu.:115.5   1st Qu.: 72.00  
 Median :0.8762   Median :28.10   Median :125.5   Median : 78.50  
 Mean   :0.8746   Mean   :29.31   Mean   :128.0   Mean   : 79.27  
 3rd Qu.:0.9327   3rd Qu.:35.50   3rd Qu.:138.5   3rd Qu.: 86.00  
 Max.   :2.4286   Max.   :64.90   Max.   :219.0   Max.   :138.00  
 NA's   :23       NA's   :91      NA's   :22      NA's   :22      
     HRTRTE            chol            hdlch           ldlch      
 Min.   : 40.00   Min.   : 2.100   Min.   :0.30   Min.   :0.600  
 1st Qu.: 61.50   1st Qu.: 4.900   1st Qu.:1.30   1st Qu.:2.700  
 Median : 67.50   Median : 5.500   Median :1.60   Median :3.300  
 Mean   : 68.02   Mean   : 5.575   Mean   :1.63   Mean   :3.329  
 3rd Qu.: 74.00   3rd Qu.: 6.200   3rd Qu.:1.90   3rd Qu.:3.900  
 Max.   :133.00   Max.   :13.000   Max.   :4.10   Max.   :9.500  
 NA's   :23       NA's   :29       NA's   :29     NA's   :130    
      trig             apob              gluc            insulin     
 Min.   : 0.200   Min.   :  21.00   Min.   : 0.300   Min.   :  1.000  
 1st Qu.: 0.800   1st Qu.:  99.92   1st Qu.: 5.000   1st Qu.:  4.990  
 Median : 1.100   Median : 141.00   Median : 5.400   Median :  7.000  
 Mean   : 1.392   Mean   : 173.89   Mean   : 5.542   Mean   :  8.762  
 3rd Qu.: 1.600   3rd Qu.: 206.97   3rd Qu.: 5.800   3rd Qu.: 10.740  
 Max.   :29.200   Max.   :2628.00   Max.   :24.700   Max.   :106.070  
 NA's   :29       NA's   :648       NA's   :29       NA's   :1300     
      crpu             tnfa            F1datblood            F1age      
 Min.   : 0.110   Min.   :   0.000   Length:6746        Min.   :40.20  
 1st Qu.: 0.600   1st Qu.:   1.780   Class :character   1st Qu.:48.80  
 Median : 1.300   Median :   2.870   Mode  :character   Median :56.70  
 Mean   : 2.455   Mean   :   6.272                      Mean   :57.77  
 3rd Qu.: 2.700   3rd Qu.:   4.500                      3rd Qu.:66.10  
 Max.   :20.000   Max.   :3044.000                      Max.   :81.60  
 NA's   :42       NA's   :379                           NA's   :1682   
    F1mrtsts         F1sclhlp        F1job_curr1      F1job_curr4b  
 Min.   :0.0000   Min.   :0.0000   Min.   :0.0000   Min.   :1.000  
 1st Qu.:0.0000   1st Qu.:0.0000   1st Qu.:0.0000   1st Qu.:1.000  
 Median :0.0000   Median :0.0000   Median :1.0000   Median :1.000  
 Mean   :0.7253   Mean   :0.4054   Mean   :0.5639   Mean   :1.727  
 3rd Qu.:2.0000   3rd Qu.:1.0000   3rd Qu.:1.0000   3rd Qu.:3.000  
 Max.   :3.0000   Max.   :1.0000   Max.   :1.0000   Max.   :3.000  
 NA's   :2490     NA's   :1749     NA's   :1754     NA's   :1754   
```

```
  F1job_curr8       F1cmp          F1hdv            F1chf       
 Min.   :1.000   Min.   :0.000   Min.   :0.0000   Min.   :0.0000  
 1st Qu.:2.000   1st Qu.:0.000   1st Qu.:0.0000   1st Qu.:0.0000  
 Median :3.000   Median :0.000   Median :0.0000   Median :0.0000  
 Mean   :4.731   Mean   :0.003   Mean   :0.0434   Mean   :0.0076  
 3rd Qu.:9.000   3rd Qu.:0.000   3rd Qu.:0.0000   3rd Qu.:0.0000  
 Max.   :9.000   Max.   :1.000   Max.   :1.0000   Max.   :1.0000  
 NA's   :1926    NA's   :1716    NA's   :1721     NA's   :1723    
     F1artm           F1cad            F1angn           F1miac      
 Min.   :0.0000   Min.   :0.0000   Min.   :0.0000   Min.   :0.0000  
 1st Qu.:0.0000   1st Qu.:0.0000   1st Qu.:0.0000   1st Qu.:0.0000  
 Median :0.0000   Median :0.0000   Median :0.0000   Median :0.0000  
 Mean   :0.0781   Mean   :0.0334   Mean   :0.0207   Mean   :0.0223  
 3rd Qu.:0.0000   3rd Qu.:0.0000   3rd Qu.:0.0000   3rd Qu.:0.0000  
 Max.   :1.0000   Max.   :1.0000   Max.   :1.0000   Max.   :1.0000  
 NA's   :1728     NA's   :1717     NA's   :1722     NA's   :1716    
     F1strk           F1vslg           F1ccth           F1cabg      
 Min.   :0.0000   Min.   :0.0000   Min.   :0.0000   Min.   :0.0000  
 1st Qu.:0.0000   1st Qu.:0.0000   1st Qu.:0.0000   1st Qu.:0.0000  
 Median :0.0000   Median :0.0000   Median :0.0000   Median :0.0000  
 Mean   :0.0189   Mean   :0.0123   Mean   :0.0507   Mean   :0.0123  
 3rd Qu.:0.0000   3rd Qu.:0.0000   3rd Qu.:0.0000   3rd Qu.:0.0000  
 Max.   :1.0000   Max.   :1.0000   Max.   :1.0000   Max.   :1.0000  
 NA's   :1717     NA's   :1719     NA's   :1714     NA's   :1711    
   F1prdbs_f       F1prdbsage_f     F1prhcl_f      F1prhclage_f  
 Min.   :0.0000   Min.   :45.00   Min.   :0.000   Min.   :45.00  
 1st Qu.:0.0000   1st Qu.:55.00   1st Qu.:0.000   1st Qu.:50.00  
 Median :0.0000   Median :65.00   Median :0.000   Median :60.00  
 Mean   :0.1361   Mean   :63.26   Mean   :0.286   Mean   :59.56  
 3rd Qu.:0.0000   3rd Qu.:70.00   3rd Qu.:1.000   3rd Qu.:67.25  
 Max.   :1.0000   Max.   :99.00   Max.   :1.000   Max.   :99.00  
 NA's   :2809     NA's   :6374    NA's   :4311    NA's   :6418   
  F1prhyptn_f     F1prhypage_f    F1prhrtat_f      F1prtaage_f   
 Min.   :0.000   Min.   :45.00   Min.   :0.0000   Min.   :40.00  
 1st Qu.:0.000   1st Qu.:50.00   1st Qu.:0.0000   1st Qu.:55.25  
 Median :0.000   Median :60.00   Median :0.0000   Median :64.00  
 Mean   :0.341   Mean   :59.03   Mean   :0.1684   Mean   :63.91  
 3rd Qu.:1.000   3rd Qu.:66.00   3rd Qu.:0.0000   3rd Qu.:72.00  
 Max.   :1.000   Max.   :99.00   Max.   :1.0000   Max.   :95.00  
 NA's   :3976    NA's   :6216    NA's   :2387     NA's   :6068   
   F1prstrk_f      F1prstkage_f    F1prdbs_m        F1prdbsage_m  
 Min.   :0.0000   Min.   :45.0   Min.   :0.0000   Min.   :45.00  
 1st Qu.:0.0000   1st Qu.:64.0   1st Qu.:0.0000   1st Qu.:55.00  
 Median :0.0000   Median :72.0   Median :0.0000   Median :62.00  
 Mean   :0.1054   Mean   :71.5   Mean   :0.1544   Mean   :62.82  
 3rd Qu.:0.0000   3rd Qu.:80.0   3rd Qu.:0.0000   3rd Qu.:70.00  
 Max.   :1.0000   Max.   :94.0   Max.   :1.0000   Max.   :99.00  
 NA's   :2335     NA's   :6315   NA's   :2453     NA's   :6300   
   F1prhcl_m      F1prhclage_m    F1prhyptn_m     F1prhypage_m 
 Min.   :0.000   Min.   :45.00   Min.   :0.000   Min.   :45.0  
 1st Qu.:0.000   1st Qu.:55.00   1st Qu.:0.000   1st Qu.:50.0  
 Median :0.000   Median :60.00   Median :0.000   Median :60.0  
 Mean   :0.249   Mean   :62.48   Mean   :0.404   Mean   :60.7  
 3rd Qu.:0.000   3rd Qu.:70.00   3rd Qu.:1.000   3rd Qu.:70.0  
 Max.   :1.000   Max.   :90.00   Max.   :1.000   Max.   :99.0  
 NA's   :3743    NA's   :6343    NA's   :3235    NA's   :5931  
  F1prhrtat_m      F1prtaage_m      F1prstrk_m      F1prstkage_m  
 Min.   :0.0000   Min.   :40.00   Min.   :0.0000   Min.   :43.00  
 1st Qu.:0.0000   1st Qu.:60.00   1st Qu.:0.0000   1st Qu.:67.00  
 Median :0.0000   Median :70.00   Median :0.0000   Median :77.00  
 Mean   :0.0666   Mean   :68.82   Mean   :0.0997   Mean   :74.57  
 3rd Qu.:0.0000   3rd Qu.:77.00   3rd Qu.:0.0000   3rd Qu.:84.00  
 Max.   :1.0000   Max.   :95.00   Max.   :1.0000   Max.   :99.00  
 NA's   :2120     NA's   :6485    NA's   :2084     NA's   :6320   
```

```
  F1famno_db     F1famno_hcl      F1famno_htn      F1famno_mai    
 Min.   :0.000   Min.   : 0.000   Min.   : 0.000   Min.   :0.0000  
 1st Qu.:0.000   1st Qu.: 0.000   1st Qu.: 0.000   1st Qu.:0.0000  
 Median :0.000   Median : 0.000   Median : 0.000   Median :0.0000  
 Mean   :0.191   Mean   : 0.423   Mean   : 0.459   Mean   :0.0945  
 3rd Qu.:0.000   3rd Qu.: 1.000   3rd Qu.: 1.000   3rd Qu.:0.0000  
 Max.   :9.000   Max.   :10.000   Max.   :10.000   Max.   :9.0000  
 NA's   :2924    NA's   :3992     NA's   :3607     NA's   :2473    
 F1famno_str       F1alcool1        F1sbsmk          F1antiHTA     
 Min.   : 0.000   Min.   :0.000   Min.   :0.0000   Min.   :0.0000  
 1st Qu.: 0.000   1st Qu.:0.000   1st Qu.:0.0000   1st Qu.:0.0000  
 Median : 0.000   Median :1.000   Median :1.0000   Median :0.0000  
 Mean   : 0.076   Mean   :1.357   Mean   :0.8111   Mean   :0.2731  
 3rd Qu.: 0.000   3rd Qu.:2.000   3rd Qu.:1.0000   3rd Qu.:1.0000  
 Max.   :21.000   Max.   :6.000   Max.   :2.0000   Max.   :1.0000  
 NA's   :2482     NA's   :1682    NA's   :1739     NA's   :1682    
     F1HTA           F1dbtld           F1etsem       F1PSQI_score  
 Min.   :0.0000   Min.   :0.0000   Min.   :10662   Min.   : 0.00  
 1st Qu.:0.0000   1st Qu.:0.0000   1st Qu.:15462   1st Qu.: 3.00  
 Median :0.0000   Median :0.0000   Median :17850   Median : 4.00  
 Mean   :0.4147   Mean   :0.0792   Mean   :18765   Mean   : 4.99  
 3rd Qu.:1.0000   3rd Qu.:0.0000   3rd Qu.:21222   3rd Qu.: 7.00  
 Max.   :1.0000   Max.   :1.0000   Max.   :44913   Max.   :20.00  
 NA's   :1692     NA's   :1698     NA's   :2565    NA's   :2668   
     F1MME           F1CESD       F1depressed       F1handgrip    
 Min.   :16.00   Min.   : 0.00   Min.   :0.0000   Min.   : 2.722  
 1st Qu.:29.00   1st Qu.: 4.00   1st Qu.:0.0000   1st Qu.:24.494  
 Median :30.00   Median : 9.00   Median :0.0000   Median :31.298  
 Mean   :29.07   Mean   :10.74   Mean   :0.1401   Mean   :33.273  
 3rd Qu.:30.00   3rd Qu.:15.00   3rd Qu.:0.0000   3rd Qu.:41.730  
 Max.   :30.00   Max.   :57.00   Max.   :1.0000   Max.   :72.575  
 NA's   :4751    NA's   :2444    NA's   :2455     NA's   :1938    
      F1ht            F1wt             F1BMI         F1BMI_cat1    
 Min.   :130.0   Min.   : 38.40   Min.   :14.21   Min.   :0.0000  
 1st Qu.:161.5   1st Qu.: 63.00   1st Qu.:23.00   1st Qu.:0.0000  
 Median :168.0   Median : 72.90   Median :25.63   Median :1.0000  
 Mean   :168.3   Mean   : 74.39   Mean   :26.20   Mean   :0.7357  
 3rd Qu.:175.0   3rd Qu.: 83.80   3rd Qu.:28.64   3rd Qu.:1.0000  
 Max.   :197.0   Max.   :154.00   Max.   :54.24   Max.   :2.0000  
 NA's   :1732    NA's   :1746     NA's   :1747    NA's   :1747    
  F1BMI_cat2       F1waist        F1waist_cat1        F1hip      
 Min.   :0.000   Min.   : 58.00   Min.   :0.0000   Min.   : 60.8  
 1st Qu.:1.000   1st Qu.: 82.62   1st Qu.:0.0000   1st Qu.: 93.0  
 Median :2.000   Median : 91.50   Median :0.0000   Median : 99.0  
 Mean   :1.721   Mean   : 91.99   Mean   :0.3849   Mean   :100.0  
 3rd Qu.:2.000   3rd Qu.:100.38   3rd Qu.:1.0000   3rd Qu.:105.5  
 Max.   :3.000   Max.   :149.00   Max.   :1.0000   Max.   :159.0  
 NA's   :1747    NA's   :1711     NA's   :1711     NA's   :1714   
     F1WHR           F1bmpsc         F1BIAcom         F1SBP      
 Min.   :0.6824   Min.   : 5.00   Min.   :1.000   Min.   : 79.5  
 1st Qu.:0.8699   1st Qu.:24.30   1st Qu.:2.000   1st Qu.:113.5  
 Median :0.9205   Median :29.90   Median :3.000   Median :124.5  
 Mean   :0.9183   Mean   :30.84   Mean   :3.062   Mean   :126.1  
 3rd Qu.:0.9700   3rd Qu.:37.30   3rd Qu.:4.000   3rd Qu.:137.0  
 Max.   :1.2222   Max.   :60.00   Max.   :6.000   Max.   :220.0  
 NA's   :1714     NA's   :2559    NA's   :6008    NA's   :1698   
     F1DBP           F1HRTRTE           F1chol          F1hdlch     
 Min.   : 42.50   Min.   : 37.00   Min.   :2.100   Min.   :0.500  
 1st Qu.: 70.50   1st Qu.: 61.00   1st Qu.:5.000   1st Qu.:1.300  
 Median : 77.50   Median : 67.00   Median :5.700   Median :1.600  
 Mean   : 78.06   Mean   : 67.53   Mean   :5.696   Mean   :1.638  
 3rd Qu.: 85.00   3rd Qu.: 73.00   3rd Qu.:6.400   3rd Qu.:1.900  
 Max.   :124.50   Max.   :124.00   Max.   :9.700   Max.   :4.900  
 NA's   :1698     NA's   :1701     NA's   :1704    NA's   :1704   
```

```
   F1ldlch          F1trig           F1gluc          F1insulin      
 Min.   :0.700   Min.   : 0.200   Min.   : 1.500   Min.   :  0.300  
 1st Qu.:2.800   1st Qu.: 0.800   1st Qu.: 5.300   1st Qu.:  4.400  
 Median :3.400   Median : 1.100   Median : 5.700   Median :  6.600  
 Mean   :3.447   Mean   : 1.362   Mean   : 5.887   Mean   :  8.711  
 3rd Qu.:4.000   3rd Qu.: 1.600   3rd Qu.: 6.100   3rd Qu.: 10.000  
 Max.   :7.000   Max.   :12.800   Max.   :23.900   Max.   :843.500  
 NA's   :1750    NA's   :1704     NA's   :1705     NA's   :1728     
     F1crpu           F1il6             F1tnfa           F1freqFFQ1    
 Min.   : 0.110   Min.   :   0.00   Min.   :   0.000   Min.   :0.0000  
 1st Qu.: 0.700   1st Qu.:   0.97   1st Qu.:   2.600   1st Qu.:0.0000  
 Median : 1.300   Median :   2.51   Median :   4.710   Median :0.0000  
 Mean   : 2.546   Mean   :  18.47   Mean   :  10.127   Mean   :0.1978  
 3rd Qu.: 2.800   3rd Qu.:   8.02   3rd Qu.:   8.312   3rd Qu.:0.2143  
 Max.   :21.000   Max.   :4423.00   Max.   :3454.000   Max.   :2.5000  
 NA's   :1775     NA's   :2077      NA's   :2074       NA's   :2141    
   F1freqFFQ2       F1freqFFQ3       F1freqFFQ4       F1freqFFQ5    
 Min.   :0.0000   Min.   :0.0000   Min.   :0.0000   Min.   :0.0000  
 1st Qu.:0.0000   1st Qu.:0.0000   1st Qu.:0.0000   1st Qu.:0.0000  
 Median :0.0000   Median :0.0893   Median :0.0000   Median :0.0000  
 Mean   :0.0962   Mean   :0.2899   Mean   :0.0501   Mean   :0.0592  
 3rd Qu.:0.0000   3rd Qu.:0.5000   3rd Qu.:0.0000   3rd Qu.:0.0357  
 Max.   :2.5000   Max.   :2.5000   Max.   :2.5000   Max.   :2.5000  
 NA's   :2141     NA's   :2141     NA's   :2141     NA's   :2141    
   F1freqFFQ6       F1freqFFQ7       F1freqFFQ8       F1freqFFQ9    
 Min.   :0.0000   Min.   :0.0000   Min.   :0.0000   Min.   :0.0000  
 1st Qu.:0.0000   1st Qu.:0.0893   1st Qu.:0.0000   1st Qu.:0.0893  
 Median :0.0357   Median :0.2143   Median :0.0000   Median :0.2143  
 Mean   :0.1056   Mean   :0.3626   Mean   :0.0222   Mean   :0.6580  
 3rd Qu.:0.0893   3rd Qu.:0.5000   3rd Qu.:0.0357   3rd Qu.:1.0000  
 Max.   :2.5000   Max.   :2.5000   Max.   :2.5000   Max.   :2.5000  
 NA's   :2141     NA's   :2141     NA's   :2141     NA's   :2141    
  F1freqFFQ10      F1freqFFQ11      F1freqFFQ12      F1freqFFQ13    
 Min.   :0.0000   Min.   :0.0000   Min.   :0.0000   Min.   :0.0000  
 1st Qu.:0.0000   1st Qu.:0.0000   1st Qu.:0.0000   1st Qu.:0.0000  
 Median :0.2143   Median :0.0000   Median :0.0000   Median :0.0000  
 Mean   :0.5360   Mean   :0.1525   Mean   :0.0634   Mean   :0.1022  
 3rd Qu.:1.0000   3rd Qu.:0.0893   3rd Qu.:0.0000   3rd Qu.:0.0357  
 Max.   :2.5000   Max.   :2.5000   Max.   :2.5000   Max.   :2.5000  
 NA's   :2141     NA's   :2141     NA's   :2141     NA's   :2141    
  F1freqFFQ14      F1freqFFQ15      F1freqFFQ16      F1freqFFQ17    
 Min.   :0.0000   Min.   :0.0000   Min.   :0.0000   Min.   :0.0000  
 1st Qu.:0.0357   1st Qu.:0.0000   1st Qu.:0.0000   1st Qu.:0.0357  
 Median :0.0893   Median :0.0893   Median :0.0357   Median :0.0893  
 Mean   :0.1363   Mean   :0.1179   Mean   :0.0573   Mean   :0.1036  
 3rd Qu.:0.2143   3rd Qu.:0.2143   3rd Qu.:0.0893   3rd Qu.:0.2143  
 Max.   :2.5000   Max.   :2.5000   Max.   :2.5000   Max.   :2.5000  
 NA's   :2141     NA's   :2141     NA's   :2141     NA's   :2141    
  F1freqFFQ18      F1freqFFQ19      F1freqFFQ20      F1freqFFQ21    
 Min.   :0.0000   Min.   :0.0000   Min.   :0.0000   Min.   :0.0000  
 1st Qu.:0.0357   1st Qu.:0.0357   1st Qu.:0.0000   1st Qu.:0.0000  
 Median :0.0893   Median :0.0893   Median :0.0000   Median :0.0000  
 Mean   :0.0914   Mean   :0.1049   Mean   :0.0275   Mean   :0.0217  
 3rd Qu.:0.0893   3rd Qu.:0.0893   3rd Qu.:0.0357   3rd Qu.:0.0357  
 Max.   :2.5000   Max.   :2.5000   Max.   :2.5000   Max.   :2.5000  
 NA's   :2141     NA's   :2141     NA's   :2141     NA's   :2141    
  F1freqFFQ22      F1freqFFQ23      F1freqFFQ24      F1freqFFQ25    
 Min.   :0.0000   Min.   :0.0000   Min.   :0.0000   Min.   :0.0000  
 1st Qu.:0.0000   1st Qu.:0.0000   1st Qu.:0.0000   1st Qu.:0.0000  
 Median :0.0357   Median :0.0000   Median :0.0000   Median :0.0357  
 Mean   :0.0392   Mean   :0.0142   Mean   :0.0066   Mean   :0.0625  
 3rd Qu.:0.0357   3rd Qu.:0.0000   3rd Qu.:0.0000   3rd Qu.:0.0893  
 Max.   :2.5000   Max.   :2.5000   Max.   :2.5000   Max.   :2.5000  
 NA's   :2141     NA's   :2141     NA's   :2141     NA's   :2141    
```

```
  F1freqFFQ26       F1freqFFQ27       F1freqFFQ28       F1freqFFQ29    
 Min.   :0.0000   Min.   :0.0000   Min.   :0.0000   Min.   :0.0000  
 1st Qu.:0.0000   1st Qu.:0.0000   1st Qu.:0.0000   1st Qu.:0.0000  
 Median :0.0357   Median :0.0000   Median :0.0893   Median :0.0357  
 Mean   :0.0500   Mean   :0.0373   Mean   :0.0880   Mean   :0.0458  
 3rd Qu.:0.0893   3rd Qu.:0.0357   3rd Qu.:0.0893   3rd Qu.:0.0893  
 Max.   :2.5000   Max.   :2.5000   Max.   :2.5000   Max.   :1.0000  
 NA's   :2141     NA's   :2141     NA's   :2141     NA's   :2141    
  F1freqFFQ30       F1freqFFQ31       F1freqFFQ32       F1freqFFQ33    
 Min.   :0.0000   Min.   :0.0000   Min.   :0.0000   Min.   :0.0000  
 1st Qu.:0.0357   1st Qu.:0.0357   1st Qu.:0.0893   1st Qu.:0.0893  
 Median :0.0893   Median :0.0893   Median :0.2143   Median :0.2143  
 Mean   :0.1335   Mean   :0.1125   Mean   :0.3290   Mean   :0.2379  
 3rd Qu.:0.2143   3rd Qu.:0.2143   3rd Qu.:0.5000   3rd Qu.:0.2143  
 Max.   :2.5000   Max.   :2.5000   Max.   :2.5000   Max.   :2.5000  
 NA's   :2141     NA's   :2141     NA's   :2141     NA's   :2141    
  F1freqFFQ34       F1freqFFQ35       F1freqFFQ36       F1freqFFQ37    
 Min.   :0.0000   Min.   :0.0000   Min.   :0.0000   Min.   :0.0000  
 1st Qu.:0.2143   1st Qu.:0.0000   1st Qu.:0.0000   1st Qu.:0.0000  
 Median :0.5000   Median :0.2143   Median :0.0357   Median :0.0000  
 Mean   :0.5619   Mean   :0.3400   Mean   :0.0777   Mean   :0.0396  
 3rd Qu.:1.0000   3rd Qu.:0.5000   3rd Qu.:0.0893   3rd Qu.:0.0357  
 Max.   :2.5000   Max.   :2.5000   Max.   :2.5000   Max.   :2.5000  
 NA's   :2141     NA's   :2141     NA's   :2141     NA's   :2141    
  F1freqFFQ38       F1freqFFQ39       F1freqFFQ40       F1freqFFQ41    
 Min.   :0.0000   Min.   :0.0000   Min.   :0.0000   Min.   :0.0000  
 1st Qu.:0.0000   1st Qu.:0.0000   1st Qu.:0.0893   1st Qu.:0.0000  
 Median :0.0357   Median :0.0357   Median :0.2143   Median :0.0357  
 Mean   :0.1060   Mean   :0.0926   Mean   :0.2053   Mean   :0.0522  
 3rd Qu.:0.0893   3rd Qu.:0.0893   3rd Qu.:0.2143   3rd Qu.:0.0893  
 Max.   :2.5000   Max.   :2.5000   Max.   :2.5000   Max.   :2.5000  
 NA's   :2141     NA's   :2141     NA's   :2141     NA's   :2141    
  F1freqFFQ42       F1freqFFQ43       F1freqFFQ44       F1freqFFQ45    
 Min.   :0.0000   Min.   :0.0000   Min.   :0.0000   Min.   :0.0000  
 1st Qu.:0.0893   1st Qu.:0.0000   1st Qu.:0.0893   1st Qu.:0.0357  
 Median :0.2143   Median :0.0357   Median :0.2143   Median :0.0893  
 Mean   :0.2315   Mean   :0.0482   Mean   :0.1804   Mean   :0.1176  
 3rd Qu.:0.2143   3rd Qu.:0.0893   3rd Qu.:0.2143   3rd Qu.:0.2143  
 Max.   :2.5000   Max.   :1.0000   Max.   :2.5000   Max.   :2.5000  
 NA's   :2141     NA's   :2141     NA's   :2141     NA's   :2141    
  F1freqFFQ46       F1freqFFQ47       F1freqFFQ48     F1freqFFQ49    
 Min.   :0.0000   Min.   :0.0000   Min.   :0.000   Min.   :0.0000  
 1st Qu.:0.0000   1st Qu.:0.0000   1st Qu.:0.000   1st Qu.:0.0893  
 Median :0.0000   Median :0.0357   Median :0.000   Median :0.0893  
 Mean   :0.0372   Mean   :0.0565   Mean   :0.013   Mean   :0.1467  
 3rd Qu.:0.0357   3rd Qu.:0.0893   3rd Qu.:0.000   3rd Qu.:0.2143  
 Max.   :2.5000   Max.   :1.0000   Max.   :1.000   Max.   :2.5000  
 NA's   :2141     NA's   :2141     NA's   :2141    NA's   :2141    
  F1freqFFQ50       F1freqFFQ51       F1freqFFQ52       F1freqFFQ53    
 Min.   :0.0000   Min.   :0.0000   Min.   :0.0000   Min.   :0.0000  
 1st Qu.:0.0000   1st Qu.:0.0000   1st Qu.:0.0000   1st Qu.:0.0000  
 Median :0.0000   Median :0.0000   Median :0.0893   Median :0.0000  
 Mean   :0.0169   Mean   :0.1714   Mean   :0.3454   Mean   :0.0708  
 3rd Qu.:0.0000   3rd Qu.:0.0893   3rd Qu.:0.5000   3rd Qu.:0.0893  
 Max.   :2.5000   Max.   :2.5000   Max.   :2.5000   Max.   :2.5000  
 NA's   :2141     NA's   :2141     NA's   :2141     NA's   :2141    
  F1freqFFQ54       F1freqFFQ55       F1freqFFQ56       F1freqFFQ57    
 Min.   :0.0000   Min.   :0.0000   Min.   :0.0000   Min.   :0.0000  
 1st Qu.:0.0000   1st Qu.:0.2143   1st Qu.:0.0000   1st Qu.:0.0000  
 Median :0.0000   Median :0.5000   Median :0.0893   Median :0.0357  
 Mean   :0.0594   Mean   :0.7017   Mean   :0.3914   Mean   :0.2687  
 3rd Qu.:0.0893   3rd Qu.:1.0000   3rd Qu.:0.5000   3rd Qu.:0.2143  
 Max.   :2.5000   Max.   :2.5000   Max.   :2.5000   Max.   :2.5000  
 NA's   :2141     NA's   :2141     NA's   :2141     NA's   :2141    
```

```
  F1freqFFQ58      F1freqFFQ59      F1freqFFQ60      F1freqFFQ61    
 Min.   :0.0000   Min.   :0.0000   Min.   :0.0000   Min.   :0.0000  
 1st Qu.:0.0000   1st Qu.:0.0000   1st Qu.:0.0000   1st Qu.:0.0000  
 Median :0.0357   Median :0.0357   Median :0.0000   Median :0.0357  
 Mean   :0.1445   Mean   :0.1728   Mean   :0.0287   Mean   :0.1074  
 3rd Qu.:0.2143   3rd Qu.:0.2143   3rd Qu.:0.0000   3rd Qu.:0.0893  
 Max.   :2.5000   Max.   :2.5000   Max.   :2.5000   Max.   :2.5000  
 NA's   :2141     NA's   :2141     NA's   :2141     NA's   :2141    
  F1freqFFQ62      F1freqFFQ63      F1freqFFQ64      F1freqFFQ65    
 Min.   :0.0000   Min.   :0.0000   Min.   :0.0000   Min.   :0.0000  
 1st Qu.:0.0000   1st Qu.:0.0000   1st Qu.:0.0000   1st Qu.:0.0000  
 Median :0.0357   Median :0.0000   Median :0.0357   Median :0.0893  
 Mean   :0.0704   Mean   :0.0188   Mean   :0.0600   Mean   :0.1696  
 3rd Qu.:0.0893   3rd Qu.:0.0357   3rd Qu.:0.0893   3rd Qu.:0.2143  
 Max.   :1.0000   Max.   :1.0000   Max.   :1.0000   Max.   :2.5000  
 NA's   :2141     NA's   :2141     NA's   :2141     NA's   :2141    
  F1freqFFQ66      F1freqFFQ67      F1freqFFQ68      F1freqFFQ69    
 Min.   :0.0000   Min.   :0.0000   Min.   :0.0000   Min.   :0.0000  
 1st Qu.:0.0893   1st Qu.:0.0357   1st Qu.:0.0000   1st Qu.:0.0000  
 Median :0.2143   Median :0.2143   Median :0.0357   Median :0.0000  
 Mean   :0.3988   Mean   :0.4455   Mean   :0.0806   Mean   :0.3187  
 3rd Qu.:0.5000   3rd Qu.:1.0000   3rd Qu.:0.0893   3rd Qu.:0.0000  
 Max.   :2.5000   Max.   :2.5000   Max.   :2.5000   Max.   :2.5000  
 NA's   :2141     NA's   :2141     NA's   :2141     NA's   :2141    
  F1freqFFQ70      F1freqFFQ71      F1freqFFQ72      F1freqFFQ73    
 Min.   :0.0000   Min.   :0.0000   Min.   :0.0000   Min.   :0.0000  
 1st Qu.:0.0000   1st Qu.:0.0000   1st Qu.:0.0000   1st Qu.:0.2143  
 Median :0.0893   Median :0.0000   Median :0.0000   Median :0.5000  
 Mean   :0.5827   Mean   :0.1743   Mean   :0.0914   Mean   :0.6939  
 3rd Qu.:1.0000   3rd Qu.:0.2143   3rd Qu.:0.0000   3rd Qu.:1.0000  
 Max.   :2.5000   Max.   :2.5000   Max.   :2.5000   Max.   :2.5000  
 NA's   :2141     NA's   :2141     NA's   :2141     NA's   :2141    
  F1freqFFQ74      F1freqFFQ75      F1freqFFQ76      F1freqFFQ77    
 Min.   :0.0000   Min.   :0.0000   Min.   :0.0000   Min.   :0.0000  
 1st Qu.:0.0000   1st Qu.:0.0000   1st Qu.:0.0000   1st Qu.:0.0000  
 Median :0.0000   Median :0.0357   Median :0.0000   Median :0.0000  
 Mean   :0.0992   Mean   :0.2392   Mean   :0.0708   Mean   :0.0347  
 3rd Qu.:0.0357   3rd Qu.:0.2143   3rd Qu.:0.0000   3rd Qu.:0.0000  
 Max.   :2.5000   Max.   :2.5000   Max.   :2.5000   Max.   :2.5000  
 NA's   :2141     NA's   :2141     NA's   :2141     NA's   :2141    
  F1freqFFQ78      F1freqFFQ79      F1freqFFQ80      F1freqFFQ81   
 Min.   :0.0000   Min.   :0.0000   Min.   :0.0000   Min.   :0.000  
 1st Qu.:0.0000   1st Qu.:0.0000   1st Qu.:0.0000   1st Qu.:1.000  
 Median :0.0000   Median :0.0000   Median :0.0000   Median :2.500  
 Mean   :0.0954   Mean   :0.0262   Mean   :0.0094   Mean   :1.673  
 3rd Qu.:0.0000   3rd Qu.:0.0000   3rd Qu.:0.0000   3rd Qu.:2.500  
 Max.   :2.5000   Max.   :2.5000   Max.   :2.5000   Max.   :2.500  
 NA's   :2141     NA's   :2141     NA's   :2141     NA's   :2141   
  F1freqFFQ82      F1freqFFQ83      F1freqFFQ84      F1freqFFQ85    
 Min.   :0.0000   Min.   :0.0000   Min.   :0.0000   Min.   :0.0000  
 1st Qu.:0.0000   1st Qu.:0.0000   1st Qu.:0.0000   1st Qu.:0.0000  
 Median :0.0000   Median :0.0000   Median :0.0000   Median :0.0000  
 Mean   :0.1956   Mean   :0.4100   Mean   :0.2943   Mean   :0.0605  
 3rd Qu.:0.0000   3rd Qu.:0.2143   3rd Qu.:0.0893   3rd Qu.:0.0000  
 Max.   :2.5000   Max.   :2.5000   Max.   :2.5000   Max.   :2.5000  
 NA's   :2141     NA's   :2141     NA's   :2141     NA's   :2141    
  F1freqFFQ86      F1freqFFQ87      F1freqFFQ88      F1freqFFQ89   
 Min.   :0.0000   Min.   :0.0000   Min.   :0.0000   Min.   :0.000  
 1st Qu.:0.0000   1st Qu.:0.0000   1st Qu.:0.0000   1st Qu.:1.000  
 Median :0.0000   Median :0.0000   Median :0.0000   Median :2.500  
 Mean   :0.2070   Mean   :0.4923   Mean   :0.1763   Mean   :1.779  
 3rd Qu.:0.0893   3rd Qu.:0.5000   3rd Qu.:0.0000   3rd Qu.:2.500  
 Max.   :2.5000   Max.   :2.5000   Max.   :2.5000   Max.   :2.500  
 NA's   :2141     NA's   :2141     NA's   :2141     NA's   :2141   
```

```
  F1freqFFQ90       F1freqFFQ91       F1freqFFQ92       F1freqFFQ93    
 Min.   :0.0000   Min.   :0.0000   Min.   :0.0000   Min.   :0.0000  
 1st Qu.:0.0000   1st Qu.:0.0000   1st Qu.:0.0000   1st Qu.:0.0357  
 Median :0.0000   Median :0.0000   Median :0.0357   Median :0.5000  
 Mean   :0.2330   Mean   :0.1874   Mean   :0.2704   Mean   :0.8499  
 3rd Qu.:0.2143   3rd Qu.:0.2143   3rd Qu.:0.2143   3rd Qu.:1.0000  
 Max.   :2.5000   Max.   :2.5000   Max.   :2.5000   Max.   :2.5000  
 NA's   :2141     NA's   :2141     NA's   :2141     NA's   :2141    
  F1freqFFQ94       F1freqFFQ95       F1freqFFQ96       F1freqFFQ97    
 Min.   :0.0000   Min.   :0.0000   Min.   :0.0000   Min.   :0.0000  
 1st Qu.:0.0000   1st Qu.:0.0357   1st Qu.:0.0000   1st Qu.:0.0000  
 Median :0.0000   Median :0.2143   Median :0.0000   Median :0.0000  
 Mean   :0.1551   Mean   :0.4225   Mean   :0.0348   Mean   :0.0359  
 3rd Qu.:0.0893   3rd Qu.:0.5000   3rd Qu.:0.0000   3rd Qu.:0.0000  
 Max.   :2.5000   Max.   :2.5000   Max.   :2.5000   Max.   :2.5000  
 NA's   :2141     NA's   :2141     NA's   :2141     NA's   :2141    
  F1FFQ1amount      F1FFQ2amount      F1FFQ3amount      F1FFQ4amount   
 Min.   :  0.00   Min.   :  0.00   Min.   :  0.00   Min.   :  0.00  
 1st Qu.:  0.00   1st Qu.:  0.00   1st Qu.:  0.00   1st Qu.:  0.00  
 Median :  0.00   Median :  0.00   Median : 16.07   Median :  0.00  
 Mean   : 33.50   Mean   : 17.16   Mean   : 52.24   Mean   :  5.67  
 3rd Qu.: 38.57   3rd Qu.:  0.00   3rd Qu.: 57.86   3rd Qu.:  0.00  
 Max.   :675.00   Max.   :675.00   Max.   :675.00   Max.   :500.00  
 NA's   :2141     NA's   :2141     NA's   :2141     NA's   :2141    
  F1FFQ5amount       F1FFQ6amount       F1FFQ7amount       F1FFQ8amount     
 Min.   :  0.000   Min.   :  0.000   Min.   :  0.000   Min.   :   0.000  
 1st Qu.:  0.000   1st Qu.:  0.000   1st Qu.:  8.036   1st Qu.:   0.000  
 Median :  0.000   Median :  2.143   Median : 19.286   Median :   0.000  
 Mean   :  5.628   Mean   :  5.816   Mean   : 30.655   Mean   :   8.145  
 3rd Qu.:  3.571   3rd Qu.:  8.036   3rd Qu.: 45.000   3rd Qu.:  12.500  
 Max.   :375.000   Max.   :225.000   Max.   :300.000   Max.   :1312.500  
 NA's   :2141      NA's   :2141      NA's   :2141      NA's   :2141      
  F1FFQ9amount      F1FFQ10amount     F1FFQ11amount      F1FFQ12amount    
 Min.   :  0.000   Min.   :  0.00   Min.   :  0.000   Min.   :  0.000  
 1st Qu.:  2.679   1st Qu.:  0.00   1st Qu.:  0.000   1st Qu.:  0.000  
 Median : 16.071   Median : 12.86   Median :  0.000   Median :  0.000  
 Mean   : 42.409   Mean   : 36.92   Mean   :  8.522   Mean   :  2.078  
 3rd Qu.: 50.000   3rd Qu.: 50.00   3rd Qu.:  6.696   3rd Qu.:  0.000  
 Max.   :187.500   Max.   :225.00   Max.   :187.500   Max.   :112.500  
 NA's   :2141      NA's   :2141     NA's   :2141      NA's   :2141     
 F1FFQ13amount      F1FFQ14amount      F1FFQ15amount      F1FFQ16amount    
 Min.   : 0.0000   Min.   :  0.000   Min.   :  0.000   Min.   :  0.000  
 1st Qu.: 0.0000   1st Qu.:  4.643   1st Qu.:  0.000   1st Qu.:  0.000  
 Median : 0.0000   Median : 12.857   Median :  8.929   Median :  3.571  
 Mean   : 1.8592   Mean   : 19.428   Mean   : 13.237   Mean   :  6.738  
 3rd Qu.: 0.8036   3rd Qu.: 27.857   3rd Qu.: 21.429   3rd Qu.:  8.929  
 Max.   :67.5000   Max.   :562.500   Max.   :375.000   Max.   :375.000  
 NA's   :2141      NA's   :2141      NA's   :2141      NA's   :2141     
 F1FFQ17amount      F1FFQ18amount      F1FFQ19amount       F1FFQ20amount    
 Min.   :  0.000   Min.   :  0.000   Min.   :  0.0000   Min.   :  0.000  
 1st Qu.:  4.464   1st Qu.:  1.964   1st Qu.:  0.8929   1st Qu.:  0.000  
 Median : 11.161   Median :  9.821   Median :  4.4643   Median :  0.000  
 Mean   : 16.656   Mean   : 11.259   Mean   :  6.0229   Mean   :  2.366  
 3rd Qu.: 21.429   3rd Qu.: 14.732   3rd Qu.:  6.6964   3rd Qu.:  2.857  
 Max.   :500.000   Max.   :412.500   Max.   :187.5000   Max.   :300.000  
 NA's   :2141      NA's   :2141      NA's   :2141       NA's   :2141     
 F1FFQ21amount      F1FFQ22amount      F1FFQ23amount      F1FFQ24amount    
 Min.   :  0.000   Min.   :  0.000   Min.   :  0.000   Min.   : 0.0000  
 1st Qu.:  0.000   1st Qu.:  0.000   1st Qu.:  0.000   1st Qu.: 0.0000  
 Median :  0.000   Median :  1.429   Median :  0.000   Median : 0.0000  
 Mean   :  1.990   Mean   :  3.076   Mean   :  1.432   Mean   : 0.3819  
 3rd Qu.:  2.143   3rd Qu.:  3.571   3rd Qu.:  0.000   3rd Qu.: 0.0000  
 Max.   :137.500   Max.   :120.000   Max.   :125.000   Max.   :75.0000  
 NA's   :2141      NA's   :2141      NA's   :2141      NA's   :2141     
```

```
 F1FFQ25amount     F1FFQ26amount     F1FFQ27amount     F1FFQ28amount   
 Min.   :  0.000   Min.   :  0.000   Min.   :  0.000   Min.   :  0.00  
 1st Qu.:  0.000   1st Qu.:  0.000   1st Qu.:  0.000   1st Qu.:  0.00  
 Median :  5.357   Median :  5.357   Median :  0.000   Median : 13.39  
 Mean   :  9.955   Mean   :  7.822   Mean   :  3.122   Mean   : 13.69  
 3rd Qu.: 13.393   3rd Qu.: 13.393   3rd Qu.:  2.857   3rd Qu.: 20.09  
 Max.   :562.500   Max.   :375.000   Max.   :300.000   Max.   :375.00  
 NA's   :2141      NA's   :2141      NA's   :2141      NA's   :2141    
 F1FFQ29amount    F1FFQ30amount     F1FFQ31amount     F1FFQ32amount    
 Min.   : 0.000   Min.   :  0.000   Min.   :  0.000   Min.   :  0.000  
 1st Qu.: 0.000   1st Qu.:  6.696   1st Qu.:  7.143   1st Qu.:  8.036  
 Median : 1.786   Median : 13.393   Median : 17.857   Median : 19.286  
 Mean   : 2.521   Mean   : 22.472   Mean   : 24.886   Mean   : 32.388  
 3rd Qu.: 4.464   3rd Qu.: 32.143   3rd Qu.: 42.857   3rd Qu.: 45.000  
 Max.   :50.000   Max.   :562.500   Max.   :750.000   Max.   :337.500  
 NA's   :2141     NA's   :2141      NA's   :2141      NA's   :2141     
 F1FFQ33amount     F1FFQ34amount     F1FFQ35amount    F1FFQ36amount    
 Min.   :  0.000   Min.   :  0.00   Min.   : 0.000   Min.   :  0.000  
 1st Qu.:  6.696   1st Qu.: 10.71   1st Qu.: 0.000   1st Qu.:  0.000  
 Median : 16.071   Median : 25.00   Median : 2.232   Median :  2.143  
 Mean   : 19.774   Mean   : 34.47   Mean   : 5.825   Mean   :  4.915  
 3rd Qu.: 24.643   3rd Qu.: 50.00   3rd Qu.: 7.500   3rd Qu.:  5.357  
 Max.   :287.500   Max.   :187.50   Max.   :62.500   Max.   :225.000  
 NA's   :2141      NA's   :2141     NA's   :2141     NA's   :2141     
 F1FFQ37amount     F1FFQ38amount      F1FFQ39amount     F1FFQ40amount    
 Min.   :  0.000   Min.   :   0.00   Min.   :  0.000   Min.   :  0.000  
 1st Qu.:  0.000   1st Qu.:   0.00   1st Qu.:  0.000   1st Qu.:  8.929  
 Median :  0.000   Median :  10.71   Median :  8.929   Median : 21.429  
 Mean   :  7.669   Mean   :  34.30   Mean   : 25.557   Mean   : 28.723  
 3rd Qu.:  8.036   3rd Qu.:  26.79   3rd Qu.: 22.321   3rd Qu.: 32.143  
 Max.   :450.000   Max.   :1125.00   Max.   :937.500   Max.   :562.500  
 NA's   :2141      NA's   :2141      NA's   :2141      NA's   :2141     
 F1FFQ41amount     F1FFQ42amount      F1FFQ43amount     F1FFQ44amount    
 Min.   :  0.000   Min.   :   0.00   Min.   :  0.000   Min.   :  0.000  
 1st Qu.:  0.000   1st Qu.:  26.79   1st Qu.:  0.000   1st Qu.:  5.357  
 Median :  4.286   Median :  42.86   Median :  5.000   Median :  8.036  
 Mean   :  6.974   Mean   :  64.61   Mean   :  8.502   Mean   : 11.476  
 3rd Qu.: 10.714   3rd Qu.:  64.29   3rd Qu.: 12.500   3rd Qu.: 12.857  
 Max.   :450.000   Max.   :1125.00   Max.   :180.000   Max.   :225.000  
 NA's   :2141      NA's   :2141      NA's   :2141      NA's   :2141     
 F1FFQ45amount     F1FFQ46amount     F1FFQ47amount    F1FFQ48amount    
 Min.   :  0.000   Min.   :  0.000   Min.   :  0.00   Min.   :  0.000  
 1st Qu.:  2.857   1st Qu.:  0.000   1st Qu.:  0.00   1st Qu.:  0.000  
 Median :  7.143   Median :  0.000   Median : 13.39   Median :  0.000  
 Mean   : 10.589   Mean   :  1.290   Mean   : 16.73   Mean   :  2.265  
 3rd Qu.: 17.143   3rd Qu.:  1.607   3rd Qu.: 22.32   3rd Qu.:  0.000  
 Max.   :200.000   Max.   :112.500   Max.   :250.00   Max.   :225.000  
 NA's   :2141      NA's   :2141      NA's   :2141     NA's   :2141     
 F1FFQ49amount     F1FFQ50amount     F1FFQ51amount    F1FFQ52amount    
 Min.   :  0.000   Min.   :  0.000   Min.   : 0.000   Min.   : 0.0000  
 1st Qu.:  4.464   1st Qu.:  0.000   1st Qu.: 0.000   1st Qu.: 0.0000  
 Median :  8.929   Median :  0.000   Median : 0.000   Median : 0.8929  
 Mean   : 13.464   Mean   :  1.837   Mean   : 1.557   Mean   : 3.0991  
 3rd Qu.: 21.429   3rd Qu.:  0.000   3rd Qu.: 0.625   3rd Qu.: 5.0000  
 Max.   :375.000   Max.   :250.000   Max.   :37.500   Max.   :37.5000  
 NA's   :2141      NA's   :2141      NA's   :2141     NA's   :2141     
 F1FFQ53amount    F1FFQ54amount     F1FFQ55amount    F1FFQ56amount   
 Min.   : 0.000   Min.   : 0.0000   Min.   :  0.00   Min.   :  0.00  
 1st Qu.: 0.000   1st Qu.: 0.0000   1st Qu.: 27.86   1st Qu.:  0.00  
 Median : 0.000   Median : 0.0000   Median : 65.00   Median : 20.09  
 Mean   : 1.318   Mean   : 0.5495   Mean   :104.82   Mean   : 65.66  
 3rd Qu.: 1.714   3rd Qu.: 0.6250   3rd Qu.:130.00   3rd Qu.: 75.00  
 Max.   :50.000   Max.   :25.0000   Max.   :487.50   Max.   :562.50  
 NA's   :2141     NA's   :2141      NA's   :2141     NA's   :2141    
```

```
 F1FFQ57amount     F1FFQ58amount     F1FFQ59amount     F1FFQ60amount    
 Min.   :  0.000   Min.   :  0.000   Min.   :  0.000   Min.   :  0.000  
 1st Qu.:  0.000   1st Qu.:  0.000   1st Qu.:  0.000   1st Qu.:  0.000  
 Median :  3.571   Median :  3.929   Median :  1.607   Median :  0.000  
 Mean   : 31.397   Mean   : 18.651   Mean   : 16.729   Mean   :  4.953  
 3rd Qu.: 32.143   3rd Qu.: 16.071   3rd Qu.: 19.286   3rd Qu.:  0.000  
 Max.   :375.000   Max.   :562.500   Max.   :337.500   Max.   :750.000  
 NA's   :2141      NA's   :2141      NA's   :2141      NA's   :2141     
 F1FFQ61amount     F1FFQ62amount     F1FFQ63amount     F1FFQ64amount    
 Min.   :  0.000   Min.   :  0.000   Min.   :  0.000   Min.   :  0.000  
 1st Qu.:  0.000   1st Qu.:  0.000   1st Qu.:  0.000   1st Qu.:  0.000  
 Median :  1.429   Median :  6.429   Median :  0.000   Median :  1.786  
 Mean   :  4.643   Mean   : 10.646   Mean   :  1.899   Mean   :  6.002  
 3rd Qu.:  3.571   3rd Qu.: 13.393   3rd Qu.:  3.571   3rd Qu.:  8.929  
 Max.   :150.000   Max.   :150.000   Max.   :100.000   Max.   :150.000  
 NA's   :2141      NA's   :2141      NA's   :2141      NA's   :2141     
 F1FFQ65amount     F1FFQ66amount    F1FFQ67amount     F1FFQ68amount    
 Min.   :  0.000   Min.   : 0.000   Min.   : 0.0000   Min.   :  0.000  
 1st Qu.:  0.000   1st Qu.: 1.339   1st Qu.: 0.8929   1st Qu.:  0.000  
 Median :  1.339   Median : 4.286   Median : 4.2857   Median :  3.571  
 Mean   :  3.917   Mean   : 8.573   Mean   : 9.4225   Mean   : 10.434  
 3rd Qu.:  3.571   3rd Qu.:12.500   3rd Qu.:20.0000   3rd Qu.: 10.714  
 Max.   :100.000   Max.   :75.000   Max.   :75.0000   Max.   :450.000  
 NA's   :2141      NA's   :2141     NA's   :2141      NA's   :2141     
 F1FFQ70amount     F1FFQ71amount   F1FFQ72amount     F1FFQ73amount   
 Min.   : 0.0000   Min.   : 0.00   Min.   : 0.0000   Min.   : 0.000  
 1st Qu.: 0.0000   1st Qu.: 0.00   1st Qu.: 0.0000   1st Qu.: 2.143  
 Median : 0.7143   Median : 0.00   Median : 0.0000   Median : 5.000  
 Mean   : 4.9409   Mean   : 1.54   Mean   : 0.8138   Mean   : 6.366  
 3rd Qu.: 8.0000   3rd Qu.: 1.50   3rd Qu.: 0.0000   3rd Qu.:10.000  
 Max.   :30.0000   Max.   :37.50   Max.   :37.5000   Max.   :37.500  
 NA's   :2141      NA's   :2141    NA's   :2141      NA's   :2141    
 F1FFQ74amount     F1FFQ75amount    F1FFQ81amount   F1FFQ82amount    
 Min.   : 0.0000   Min.   : 0.000   Min.   :  0.0   Min.   :  0.000  
 1st Qu.: 0.0000   1st Qu.: 0.000   1st Qu.: 80.0   1st Qu.:  0.000  
 Median : 0.0000   Median : 0.250   Median :200.0   Median :  0.000  
 Mean   : 0.8655   Mean   : 2.101   Mean   :154.3   Mean   :  5.956  
 3rd Qu.: 0.2500   3rd Qu.: 2.143   3rd Qu.:200.0   3rd Qu.:  0.000  
 Max.   :25.0000   Max.   :37.500   Max.   :300.0   Max.   :112.500  
 NA's   :2141      NA's   :2141     NA's   :2141    NA's   :2141     
 F1FFQ83amount    F1FFQ84amount     F1FFQ85amount     F1FFQ86amount   
 Min.   :  0.00   Min.   : 0.000   Min.   :  0.000   Min.   :  0.00  
 1st Qu.:  0.00   1st Qu.: 0.000   1st Qu.:  0.000   1st Qu.:  0.00  
 Median :  0.00   Median : 0.000   Median :  0.000   Median :  0.00  
 Mean   : 12.68   Mean   : 3.678   Mean   :  9.819   Mean   : 33.57  
 3rd Qu.:  7.50   3rd Qu.: 1.071   3rd Qu.:  0.000   3rd Qu.: 16.07  
 Max.   :112.50   Max.   :45.000   Max.   :562.500   Max.   :562.50  
 NA's   :2141     NA's   :2141     NA's   :2141      NA's   :2141    
 F1FFQ87amount   F1FFQ88amount    F1FFQ89amount   F1FFQ90amount   
 Min.   :  0.0   Min.   :  0.00   Min.   :  0.0   Min.   :  0.00  
 1st Qu.:  0.0   1st Qu.:  0.00   1st Qu.:200.0   1st Qu.:  0.00  
 Median :  0.0   Median :  0.00   Median :500.0   Median :  0.00  
 Mean   :128.6   Mean   : 46.36   Mean   :472.1   Mean   : 55.98  
 3rd Qu.:100.0   3rd Qu.:  0.00   3rd Qu.:750.0   3rd Qu.: 42.86  
 Max.   :750.0   Max.   :750.00   Max.   :750.0   Max.   :750.00  
 NA's   :2141    NA's   :2141     NA's   :2141    NA's   :2141    
 F1FFQ91amount    F1FFQ92amount     F1FFQ93amount     F1FFQ94amount    
 Min.   :  0.00   Min.   :  0.000   Min.   :  0.000   Min.   :   0.00  
 1st Qu.:  0.00   1st Qu.:  0.000   1st Qu.:  7.143   1st Qu.:   0.00  
 Median :  0.00   Median :  7.143   Median :100.000   Median :   0.00  
 Mean   : 38.87   Mean   : 57.823   Mean   :212.221   Mean   :  51.96  
 3rd Qu.: 42.86   3rd Qu.: 64.286   3rd Qu.:300.000   3rd Qu.:  26.79  
 Max.   :750.00   Max.   :750.000   Max.   :750.000   Max.   :1125.00  
 NA's   :2141     NA's   :2141      NA's   :2141      NA's   :2141     
```

```
 F1FFQ95amount     F1FFQ96amount     F1FFQ97amount       F1numitems   
 Min.   :  0.000   Min.   :  0.000   Min.   :  0.000   Min.   : 4.00  
 1st Qu.:  5.357   1st Qu.:  0.000   1st Qu.:  0.000   1st Qu.:42.00  
 Median : 32.143   Median :  0.000   Median :  0.000   Median :50.00  
 Mean   : 74.910   Mean   :  1.804   Mean   :  3.366   Mean   :50.01  
 3rd Qu.:112.500   3rd Qu.:  0.000   3rd Qu.:  0.000   3rd Qu.:58.00  
 Max.   :562.500   Max.   :187.500   Max.   :375.000   Max.   :88.00  
 NA's   :2141      NA's   :2141      NA's   :2141      NA's   :2141   
   F1sumtot1      F1sumtot2      F1sumtot3      F1sumprot1       F1sumpveg1    
 Min.   :   0   Min.   :   0   Min.   :   0   Min.   :   0.0   Min.   :  0.00  
 1st Qu.:1272   1st Qu.:1275   1st Qu.:1204   1st Qu.: 197.3   1st Qu.: 57.04  
 Median :1628   Median :1659   Median :1556   Median : 263.8   Median : 77.48  
 Mean   :1715   Mean   :1758   Mean   :1639   Mean   : 280.8   Mean   : 85.86  
 3rd Qu.:2063   3rd Qu.:2119   3rd Qu.:1983   3rd Qu.: 341.0   3rd Qu.:107.67  
 Max.   :7137   Max.   :7587   Max.   :7086   Max.   :2242.1   Max.   :334.80  
 NA's   :2141   NA's   :2141   NA's   :2141   NA's   :2141     NA's   :2141    
   F1sumpani1       F1sumgluc1       F1sumgsuc1       F1sumgpol1    
 Min.   :   0.0   Min.   :   0.0   Min.   :   0.0   Min.   :   0.0  
 1st Qu.: 127.4   1st Qu.: 583.7   1st Qu.: 260.4   1st Qu.: 263.0  
 Median : 180.4   Median : 794.9   Median : 373.6   Median : 379.0  
 Mean   : 194.9   Mean   : 849.3   Mean   : 413.9   Mean   : 432.8  
 3rd Qu.: 239.8   3rd Qu.:1052.9   3rd Qu.: 512.5   3rd Qu.: 558.6  
 Max.   :2097.2   Max.   :3549.0   Max.   :2312.1   Max.   :1788.0  
 NA's   :2141     NA's   :2141     NA's   :2141     NA's   :2141    
   F1sumlipi1       F1sumlsat1       F1sumlmon1       F1sumlpol1    
 Min.   :   0.0   Min.   :   0.0   Min.   :   0.0   Min.   :  0.00  
 1st Qu.: 436.8   1st Qu.: 152.8   1st Qu.: 170.2   1st Qu.: 58.95  
 Median : 582.8   Median : 213.0   Median : 230.9   Median : 80.86  
 Mean   : 627.9   Mean   : 235.4   Mean   : 249.6   Mean   : 88.95  
 3rd Qu.: 770.1   3rd Qu.: 293.1   3rd Qu.: 306.8   3rd Qu.:107.68  
 Max.   :3541.0   Max.   :1728.9   Max.   :1648.2   Max.   :669.71  
 NA's   :2141     NA's   :2141     NA's   :2141     NA's   :2141    
   F1sumfibr1     F1sumchol1        F1sumalco          F1sumcalc1    
 Min.   : 0.00   Min.   :   0.0   Min.   :   0.00   Min.   :  72.6  
 1st Qu.: 9.20   1st Qu.: 189.6   1st Qu.:   8.25   1st Qu.: 618.4  
 Median :13.40   Median : 258.2   Median :  30.81   Median : 878.5  
 Mean   :15.32   Mean   : 282.7   Mean   :  72.44   Mean   : 982.1  
 3rd Qu.:19.20   3rd Qu.: 343.1   3rd Qu.:  92.40   3rd Qu.:1216.8  
 Max.   :85.50   Max.   :2227.4   Max.   :1304.62   Max.   :4662.1  
 NA's   :2141    NA's   :2141     NA's   :2141      NA's   :2141    
   F1sumfer1        F1sumret1          F1sumcaro1      F1sumvitd1    
 Min.   : 0.500   Min.   :    0.0   Min.   :    0   Min.   : 0.000  
 1st Qu.: 7.300   1st Qu.:  200.6   1st Qu.: 2014   1st Qu.: 1.200  
 Median : 9.400   Median :  318.5   Median : 3140   Median : 2.100  
 Mean   : 9.983   Mean   :  495.9   Mean   : 3902   Mean   : 2.461  
 3rd Qu.:12.000   3rd Qu.:  564.6   3rd Qu.: 4751   3rd Qu.: 3.100  
 Max.   :64.400   Max.   :17898.1   Max.   :47303   Max.   :64.200  
 NA's   :2141     NA's   :2141      NA's   :2141    NA's   :2141    
   F1sumeau1        F1sumprot3       F1sumpveg3       F1sumpani3    
 Min.   : 0.000   Min.   :   0.0   Min.   :  0.00   Min.   :   0.0  
 1st Qu.: 6.000   1st Qu.: 188.8   1st Qu.: 51.80   1st Qu.: 123.3  
 Median :10.500   Median : 248.4   Median : 71.30   Median : 170.8  
 Mean   : 8.743   Mean   : 263.6   Mean   : 78.78   Mean   : 184.8  
 3rd Qu.:10.600   3rd Qu.: 316.8   3rd Qu.: 99.00   3rd Qu.: 224.5  
 Max.   :31.500   Max.   :2242.1   Max.   :334.80   Max.   :2097.2  
 NA's   :2141     NA's   :2141     NA's   :2141     NA's   :2141    
   F1sumgluc3       F1sumgsuc3       F1sumgpol3       F1sumlipi3    
 Min.   :   0.0   Min.   :   0.0   Min.   :   0.0   Min.   :   0.0  
 1st Qu.: 546.2   1st Qu.: 247.6   1st Qu.: 242.1   1st Qu.: 411.3  
 Median : 740.0   Median : 358.9   Median : 345.3   Median : 541.0  
 Mean   : 795.7   Mean   : 398.7   Mean   : 394.5   Mean   : 579.6  
 3rd Qu.: 981.7   3rd Qu.: 498.4   3rd Qu.: 503.5   3rd Qu.: 703.7  
 Max.   :3549.0   Max.   :2312.1   Max.   :1785.9   Max.   :3540.9  
 NA's   :2141     NA's   :2141     NA's   :2141     NA's   :2141    
```

```
   F1sumlsat3        F1sumlmon3        F1sumlpol3        F1sumcalc3    
 Min.   :   0.0   Min.   :   0.0   Min.   :  0.00   Min.   :  72.0  
 1st Qu.: 144.8   1st Qu.: 160.1   1st Qu.: 55.00   1st Qu.: 611.7  
 Median : 198.0   Median : 215.5   Median : 73.90   Median : 872.3  
 Mean   : 215.7   Mean   : 231.8   Mean   : 80.68   Mean   : 974.0  
 3rd Qu.: 264.9   3rd Qu.: 282.7   3rd Qu.: 97.60   3rd Qu.:1211.5  
 Max.   :1632.1   Max.   :1508.0   Max.   :669.70   Max.   :4646.3  
 NA's   :2141     NA's   :2141     NA's   :2141     NA's   :2141    
   F1sumfer3         F1sumret3          F1sumcaro3       F1sumvitd3    
 Min.   : 0.500   Min.   :    0.0   Min.   :    0   Min.   : 0.000  
 1st Qu.: 6.700   1st Qu.:  200.6   1st Qu.: 2014   1st Qu.: 1.200  
 Median : 8.700   Median :  318.5   Median : 3140   Median : 2.100  
 Mean   : 9.294   Mean   :  495.9   Mean   : 3902   Mean   : 2.461  
 3rd Qu.:11.200   3rd Qu.:  564.6   3rd Qu.: 4751   3rd Qu.: 3.100  
 Max.   :64.400   Max.   :17898.1   Max.   :47303   Max.   :64.200  
 NA's   :2141     NA's   :2141      NA's   :2141    NA's   :2141    
   F1sumfibr3       F1sumchol3       F1pct_prot1      F1pct_pveg1   
 Min.   : 0.00   Min.   :   0.0   Min.   : 4.50   Min.   :0.000  
 1st Qu.: 9.20   1st Qu.: 189.6   1st Qu.:13.40   1st Qu.:3.800  
 Median :13.40   Median : 258.2   Median :15.20   Median :4.500  
 Mean   :15.32   Mean   : 282.7   Mean   :15.59   Mean   :4.648  
 3rd Qu.:19.20   3rd Qu.: 343.1   3rd Qu.:17.30   3rd Qu.:5.400  
 Max.   :85.50   Max.   :2227.4   Max.   :46.30   Max.   :9.500  
 NA's   :2141    NA's   :2141     NA's   :2142    NA's   :2142   
  F1pct_pani1      F1pct_gluc1     F1pct_gsuc1      F1pct_gpol1      F1pct_lipi1   
 Min.   : 1.20   Min.   :13.4   Min.   : 2.30   Min.   : 0.00   Min.   : 9.40  
 1st Qu.: 8.50   1st Qu.:40.4   1st Qu.:17.60   1st Qu.:17.40   1st Qu.:29.20  
 Median :10.50   Median :46.6   Median :22.60   Median :21.90   Median :33.80  
 Mean   :10.94   Mean   :46.4   Mean   :23.53   Mean   :22.74   Mean   :33.87  
 3rd Qu.:12.90   3rd Qu.:52.6   3rd Qu.:28.60   3rd Qu.:27.30   3rd Qu.:38.30  
 Max.   :43.00   Max.   :83.0   Max.   :75.70   Max.   :58.50   Max.   :59.50  
 NA's   :2142    NA's   :2142   NA's   :2142    NA's   :2142    NA's   :2142   
  F1pct_lsat1      F1pct_lmon1     F1pct_lpol1      F1pct_fibr1    
 Min.   : 1.00   Min.   : 1.3   Min.   : 0.50   Min.   :0.0000  
 1st Qu.:10.20   1st Qu.:11.2   1st Qu.: 3.70   1st Qu.:0.6000  
 Median :12.40   Median :13.3   Median : 4.50   Median :0.8000  
 Mean   :12.51   Mean   :13.6   Mean   : 4.75   Mean   :0.8927  
 3rd Qu.:14.60   3rd Qu.:15.7   3rd Qu.: 5.50   3rd Qu.:1.1000  
 Max.   :28.60   Max.   :31.2   Max.   :14.90   Max.   :3.1000  
 NA's   :2142    NA's   :2142   NA's   :2142    NA's   :2142    
  F1pct_chol1       F1pct_alco1       F1pct_prot3      F1pct_pveg3   
 Min.   :  2.20   Min.   : 0.000   Min.   : 6.60   Min.   :0.000  
 1st Qu.: 12.90   1st Qu.: 0.400   1st Qu.:14.00   1st Qu.:4.100  
 Median : 16.00   Median : 2.000   Median :16.00   Median :4.700  
 Mean   : 16.73   Mean   : 4.135   Mean   :16.37   Mean   :4.871  
 3rd Qu.: 19.40   3rd Qu.: 5.500   3rd Qu.:18.20   3rd Qu.:5.600  
 Max.   :138.60   Max.   :58.300   Max.   :46.30   Max.   :9.900  
 NA's   :2142     NA's   :2142     NA's   :2142    NA's   :2142   
  F1pct_pani3    F1pct_gluc3      F1pct_gsuc3      F1pct_gpol3      F1pct_lipi3   
 Min.   : 1.6   Min.   :13.60   Min.   : 2.30   Min.   : 0.00   Min.   : 9.40  
 1st Qu.: 8.8   1st Qu.:42.90   1st Qu.:18.70   1st Qu.:18.30   1st Qu.:30.70  
 Median :11.0   Median :48.70   Median :23.70   Median :23.00   Median :35.50  
 Mean   :11.5   Mean   :48.55   Mean   :24.58   Mean   :23.83   Mean   :35.57  
 3rd Qu.:13.6   3rd Qu.:54.30   3rd Qu.:29.60   3rd Qu.:28.50   3rd Qu.:40.30  
 Max.   :43.0   Max.   :83.00   Max.   :75.70   Max.   :58.50   Max.   :60.90  
 NA's   :2142   NA's   :2142    NA's   :2142    NA's   :2142    NA's   :2142   
  F1pct_lsat3      F1pct_lmon3      F1pct_lpol3       F1pct_fibr3    
 Min.   : 1.00   Min.   : 1.30   Min.   : 0.500   Min.   :0.0000  
 1st Qu.:10.80   1st Qu.:11.70   1st Qu.: 3.900   1st Qu.:0.7000  
 Median :13.00   Median :14.00   Median : 4.700   Median :0.9000  
 Mean   :13.14   Mean   :14.28   Mean   : 4.987   Mean   :0.9314  
 3rd Qu.:15.40   3rd Qu.:16.60   3rd Qu.: 5.800   3rd Qu.:1.1000  
 Max.   :28.60   Max.   :33.80   Max.   :16.000   Max.   :3.1000  
 NA's   :2142    NA's   :2142    NA's   :2142     NA's   :2142    
```

```
  F1pct_chol3       F1Fruits        F1Fruits_OK     F1Vegetables  
 Min.   :  2.30   Min.   : 0.0000   Min.   :0.000   Min.   : 0.000  
 1st Qu.: 13.50   1st Qu.: 0.7679   1st Qu.:0.000   1st Qu.: 0.875  
 Median : 16.70   Median : 1.5714   Median :0.000   Median : 1.268  
 Mean   : 17.58   Mean   : 2.0352   Mean   :0.413   Mean   : 1.514  
 3rd Qu.: 20.43   3rd Qu.: 2.8393   3rd Qu.:1.000   3rd Qu.: 1.857  
 Max.   :139.90   Max.   :15.0000   Max.   :1.000   Max.   :20.000  
 NA's   :2142     NA's   :2141      NA's   :2141    NA's   :2141    
 F1Vegetables_OK      F1Meat           F1Meat_OK          F1Fish      
 Min.   :0.0000   Min.   :  0.000   Min.   :0.0000   Min.   : 0.000  
 1st Qu.:0.0000   1st Qu.:  2.875   1st Qu.:0.0000   1st Qu.: 0.750  
 Median :0.0000   Median :  4.250   Median :1.0000   Median : 1.500  
 Mean   :0.0736   Mean   :  4.848   Mean   :0.6165   Mean   : 1.636  
 3rd Qu.:0.0000   3rd Qu.:  6.000   3rd Qu.:1.0000   3rd Qu.: 2.125  
 Max.   :1.0000   Max.   :192.500   Max.   :1.0000   Max.   :52.750  
 NA's   :2141     NA's   :2141      NA's   :2141     NA's   :2141    
  F1Fish_OK        F1Fish2         F1Fish2_OK        F1Dairy       
 Min.   :0.000   Min.   : 0.000   Min.   :0.0000   Min.   : 0.0000  
 1st Qu.:0.000   1st Qu.: 0.250   1st Qu.:0.0000   1st Qu.: 0.5893  
 Median :1.000   Median : 0.875   Median :0.0000   Median : 1.0893  
 Mean   :0.658   Mean   : 1.035   Mean   :0.3941   Mean   : 1.3639  
 3rd Qu.:1.000   3rd Qu.: 1.500   3rd Qu.:1.0000   3rd Qu.: 1.7321  
 Max.   :1.000   Max.   :18.125   Max.   :1.0000   Max.   :12.5357  
 NA's   :2141    NA's   :2141     NA's   :2141     NA's   :2141     
  F1Dairy_OK        F1Nb_OK         F1Cat_OK       F1Recom_OK    
 Min.   :0.0000   Min.   :0.000   Min.   :0.000   Min.   :0.0000  
 1st Qu.:0.0000   1st Qu.:1.000   1st Qu.:1.000   1st Qu.:0.0000  
 Median :0.0000   Median :2.000   Median :2.000   Median :0.0000  
 Mean   :0.0862   Mean   :1.847   Mean   :1.845   Mean   :0.2384  
 3rd Qu.:0.0000   3rd Qu.:2.000   3rd Qu.:2.000   3rd Qu.:0.0000  
 Max.   :1.0000   Max.   :5.000   Max.   :4.000   Max.   :1.0000  
 NA's   :2141     NA's   :2141    NA's   :2141    NA's   :2141    
   F1Nb2_OK       F1Cat2_OK      F1Recom2_OK       F1Mediter1  
 Min.   :0.000   Min.   :0.000   Min.   :0.0000   Min.   :0.00  
 1st Qu.:1.000   1st Qu.:1.000   1st Qu.:0.0000   1st Qu.:3.00  
 Median :1.000   Median :1.000   Median :0.0000   Median :4.00  
 Mean   :1.583   Mean   :1.582   Mean   :0.1755   Mean   :3.95  
 3rd Qu.:2.000   3rd Qu.:2.000   3rd Qu.:0.0000   3rd Qu.:5.00  
 Max.   :5.000   Max.   :4.000   Max.   :1.0000   Max.   :8.00  
 NA's   :2141    NA's   :2141    NA's   :2141     NA's   :2142  
  F1Mediter2       F1AHEI1         F1AHEI2       F1vegetarian   
 Min.   :0.000   Min.   : 3.00   Min.   : 3.00   Min.   :0.0000  
 1st Qu.:3.000   1st Qu.:25.00   1st Qu.:25.00   1st Qu.:0.0000  
 Median :5.000   Median :31.50   Median :32.00   Median :0.0000  
 Mean   :4.607   Mean   :31.79   Mean   :31.93   Mean   :0.0052  
 3rd Qu.:6.000   3rd Qu.:39.00   3rd Qu.:39.00   3rd Qu.:0.0000  
 Max.   :9.000   Max.   :69.50   Max.   :70.50   Max.   :1.0000  
 NA's   :2142    NA's   :2142    NA's   :2142    NA's   :2141    
  F2datquest           F2mrtsts       F2family18       F2income2    
 Length:6746        Min.   :0.00   Min.   : 1.000   Min.   :1.000  
 Class :character   1st Qu.:0.00   1st Qu.: 3.000   1st Qu.:1.000  
 Mode  :character   Median :0.00   Median : 4.000   Median :2.000  
                    Mean   :0.82   Mean   : 5.561   Mean   :2.014  
                    3rd Qu.:2.00   3rd Qu.: 7.000   3rd Qu.:3.000  
                    Max.   :3.00   Max.   :77.000   Max.   :3.000  
                    NA's   :2740   NA's   :2450     NA's   :3029   
  F2alcool1        F2sbsmk          F2antiHTA          F2HTA       
 Min.   :0.000   Min.   :0.0000   Min.   :0.0000   Min.   :0.0000  
 1st Qu.:0.000   1st Qu.:0.0000   1st Qu.:0.0000   1st Qu.:0.0000  
 Median :1.000   Median :1.0000   Median :0.0000   Median :0.0000  
 Mean   :1.272   Mean   :0.7706   Mean   :0.3477   Mean   :0.4829  
 3rd Qu.:2.000   3rd Qu.:1.0000   3rd Qu.:1.0000   3rd Qu.:1.0000  
 Max.   :6.000   Max.   :2.0000   Max.   :1.0000   Max.   :1.0000  
 NA's   :2506    NA's   :2251     NA's   :1865     NA's   :2060    
```

```
  F2PSQI_score       F2MME           F2CESD        F2depressed   
 Min.   : 0.00   Min.   : 0.00   Min.   : 0.00   Min.   :0.000  
 1st Qu.: 3.00   1st Qu.:28.00   1st Qu.: 4.00   1st Qu.:0.000  
 Median : 4.00   Median :29.00   Median : 8.00   Median :0.000  
 Mean   : 4.88   Mean   :28.85   Mean   :10.09   Mean   :0.119  
 3rd Qu.: 6.00   3rd Qu.:30.00   3rd Qu.:14.00   3rd Qu.:0.000  
 Max.   :17.00   Max.   :30.00   Max.   :54.00   Max.   :1.000  
 NA's   :4160    NA's   :4447    NA's   :3204    NA's   :3204   
   F2BPI_cat       F2handgrip          F2ht           F2BMI      
 Min.   :0.000   Min.   : 2.268   Min.   :130.0   Min.   :13.94  
 1st Qu.:1.000   1st Qu.:24.948   1st Qu.:160.5   1st Qu.:23.05  
 Median :1.000   Median :31.751   Median :167.0   Median :25.94  
 Mean   :1.304   Mean   :33.818   Mean   :167.5   Mean   :26.42  
 3rd Qu.:2.000   3rd Qu.:43.091   3rd Qu.:174.3   3rd Qu.:29.06  
 Max.   :3.000   Max.   :68.039   Max.   :196.5   Max.   :53.10  
 NA's   :5309    NA's   :2379     NA's   :2262    NA's   :2265   
   F2BMI_cat1       F2BMI_cat2        F2waist        F2waist_cat    
 Min.   :0.0000   Min.   :0.000   Min.   : 57.00   Min.   :0.0000  
 1st Qu.:0.0000   1st Qu.:1.000   1st Qu.: 82.25   1st Qu.:0.0000  
 Median :1.0000   Median :2.000   Median : 91.50   Median :0.0000  
 Mean   :0.7751   Mean   :1.758   Mean   : 91.89   Mean   :0.3776  
 3rd Qu.:1.0000   3rd Qu.:2.000   3rd Qu.:100.50   3rd Qu.:1.0000  
 Max.   :2.0000   Max.   :3.000   Max.   :146.50   Max.   :1.0000  
 NA's   :2265     NA's   :2265    NA's   :2263     NA's   :2263    
     F2hip           F2WHR           F2bmpsc         F2BIAcom    
 Min.   : 60.0   Min.   :0.5534   Min.   : 4.20   Min.   :1.000  
 1st Qu.: 97.0   1st Qu.:0.8242   1st Qu.:26.05   1st Qu.:1.000  
 Median :102.0   Median :0.8920   Median :32.10   Median :4.000  
 Mean   :102.9   Mean   :0.8915   Mean   :32.71   Mean   :2.893  
 3rd Qu.:108.0   3rd Qu.:0.9533   3rd Qu.:39.35   3rd Qu.:4.000  
 Max.   :158.0   Max.   :1.5167   Max.   :61.50   Max.   :6.000  
 NA's   :2263    NA's   :2263     NA's   :2975    NA's   :5651   
   F2Fasting        F2chol           F2hdlch        F2ldlch     
 Min.   :1.000   Min.   : 1.900   Min.   :0.3    Min.   :0.400  
 1st Qu.:1.000   1st Qu.: 4.700   1st Qu.:1.3    1st Qu.:2.500  
 Median :1.000   Median : 5.300   Median :1.5    Median :3.100  
 Mean   :1.157   Mean   : 5.327   Mean   :1.6    Mean   :3.147  
 3rd Qu.:1.000   3rd Qu.: 6.000   3rd Qu.:1.9    3rd Qu.:3.700  
 Max.   :9.000   Max.   :11.200   Max.   :5.0    Max.   :7.000  
 NA's   :1971    NA's   :2236     NA's   :2236   NA's   :2285   
     F2trig           F2gluc          F2insulin          F2hba1c     
 Min.   : 0.200   Min.   : 3.000   Min.   :  0.200   Min.   :18.00  
 1st Qu.: 0.800   1st Qu.: 4.900   1st Qu.:  5.225   1st Qu.:34.00  
 Median : 1.100   Median : 5.200   Median :  7.800   Median :37.00  
 Mean   : 1.303   Mean   : 5.464   Mean   :  9.944   Mean   :38.27  
 3rd Qu.: 1.500   3rd Qu.: 5.700   3rd Qu.: 12.000   3rd Qu.:40.00  
 Max.   :25.600   Max.   :16.800   Max.   :546.800   Max.   :99.00  
 NA's   :2236     NA's   :2237     NA's   :2252      NA's   :2236   
   F2totprot         F2alb           F2alkp         F2alkp_new    
 Min.   :54.00   Min.   :28.00   Min.   : 13.00   Min.   : 13.65  
 1st Qu.:68.00   1st Qu.:41.25   1st Qu.: 51.00   1st Qu.: 53.55  
 Median :70.00   Median :43.00   Median : 61.00   Median : 64.05  
 Mean   :70.55   Mean   :43.00   Mean   : 64.16   Mean   : 67.37  
 3rd Qu.:73.00   3rd Qu.:44.00   3rd Qu.: 74.00   3rd Qu.: 77.70  
 Max.   :96.00   Max.   :52.00   Max.   :260.00   Max.   :273.00  
 NA's   :2237    NA's   :2236    NA's   :2355     NA's   :2355    
     F2alat           F2asat           F2gamgt           F2ProBNP     
 Min.   :  6.00   Min.   :  8.00   Min.   :   4.00   Min.   :   0.0  
 1st Qu.: 17.00   1st Qu.: 20.00   1st Qu.:  16.00   1st Qu.:   0.0  
 Median : 22.00   Median : 23.00   Median :  23.00   Median :  63.0  
 Mean   : 25.61   Mean   : 24.73   Mean   :  35.67   Mean   : 120.9  
 3rd Qu.: 30.00   3rd Qu.: 27.00   3rd Qu.:  37.00   3rd Qu.: 120.0  
 Max.   :274.00   Max.   :279.00   Max.   :1896.00   Max.   :9212.0  
 NA's   :2236     NA's   :2236     NA's   :2236      NA's   :2238    
```

```
  F2ProBNP_d      F2ProBNP_t         F2Troponin       F2Troponin_d    
 Min.   :   5.0   Length:6746        Min.   :  0.000   Min.   :  3.000  
 1st Qu.:  66.0   Class :character   1st Qu.:  0.000   1st Qu.:  4.000  
 Median : 100.0   Mode  :character   Median :  5.000   Median :  6.000  
 Mean   : 190.3                      Mean   :  6.329   Mean   :  8.496  
 3rd Qu.: 167.0                      3rd Qu.:  8.000   3rd Qu.:  9.000  
 Max.   :9212.0                      Max.   :325.000   Max.   :325.000  
 NA's   :3882                        NA's   :2256      NA's   :3401     
 F2Troponin_t            F2na            F2k             F2ca      
 Length:6746        Min.   :126.0   Min.   :2.200   Min.   :1.840  
 Class :character   1st Qu.:140.0   1st Qu.:4.000   1st Qu.:2.270  
 Mode  :character   Median :141.0   Median :4.200   Median :2.320  
                    Mean   :141.4   Mean   :4.177   Mean   :2.326  
                    3rd Qu.:143.0   3rd Qu.:4.400   3rd Qu.:2.380  
                    Max.   :149.0   Max.   :6.500   Max.   :2.930  
                    NA's   :2237    NA's   :2237    NA's   :2236   
     F2cap            F2cr            F2uric      F2magnesium    
 Min.   :1.770   Min.   : 38.00   Min.   : 64    Min.   :0.3700  
 1st Qu.:2.210   1st Qu.: 68.00   1st Qu.:256    1st Qu.:0.8200  
 Median :2.260   Median : 77.00   Median :306    Median :0.8500  
 Mean   :2.267   Mean   : 79.01   Mean   :314    Mean   :0.8505  
 3rd Qu.:2.310   3rd Qu.: 87.00   3rd Qu.:363    3rd Qu.:0.8900  
 Max.   :2.900   Max.   :643.00   Max.   :667    Max.   :1.0800  
 NA's   :2236    NA's   :2236     NA's   :2237   NA's   :2255    
     F2iron        F2ferritin     F2transferrin       F2crpu       
 Min.   : 2.30   Min.   :   7.0   Min.   :16.00   Min.   : 0.000  
 1st Qu.:14.45   1st Qu.:  88.0   1st Qu.:28.00   1st Qu.: 0.500  
 Median :17.80   Median : 145.0   Median :31.00   Median : 1.100  
 Mean   :18.25   Mean   : 183.2   Mean   :31.07   Mean   : 2.163  
 3rd Qu.:21.50   3rd Qu.: 224.0   3rd Qu.:33.00   3rd Qu.: 2.300  
 Max.   :54.80   Max.   :3506.0   Max.   :52.00   Max.   :21.000  
 NA's   :2255    NA's   :2258     NA's   :2256    NA's   :2245    
   F2crpu_d        F2crpu_t             F2il1b            F2il6       
 Min.   : 0.070   Length:6746        Min.   : 0.000   Min.   : 0.000  
 1st Qu.: 0.500   Class :character   1st Qu.: 0.010   1st Qu.: 0.200  
 Median : 1.100   Mode  :character   Median : 0.030   Median : 0.360  
 Mean   : 2.029                      Mean   : 0.074   Mean   : 0.713  
 3rd Qu.: 2.300                      3rd Qu.: 0.060   3rd Qu.: 0.680  
 Max.   :19.800                      Max.   :41.270   Max.   :61.800  
 NA's   :2548                        NA's   :4787     NA's   :4400    
     F2tnfa             F2IFNg             F2il10            F2il8        
 Min.   :  0.190   Min.   :  0.030   Min.   : 0.003   Min.   :  0.030  
 1st Qu.:  1.240   1st Qu.:  1.780   1st Qu.: 0.107   1st Qu.:  5.870  
 Median :  1.650   Median :  3.086   Median : 0.156   Median :  8.120  
 Mean   :  1.944   Mean   :  5.731   Mean   : 0.292   Mean   :  9.191  
 3rd Qu.:  2.240   3rd Qu.:  5.670   3rd Qu.: 0.227   3rd Qu.: 10.972  
 Max.   :121.250   Max.   :722.967   Max.   :23.799   Max.   :215.859  
 NA's   :3764      NA's   :3878      NA's   :5653     NA's   :3775     
   F2il1223            F2il16            F2tnfb            F2VEGF       
 Min.   :   6.764   Min.   :  5.361   Min.   : 0.001   Min.   :   1.30  
 1st Qu.:  43.051   1st Qu.: 79.439   1st Qu.: 0.059   1st Qu.:  92.92  
 Median :  72.340   Median :102.930   Median : 0.120   Median : 162.26  
 Mean   :  87.260   Mean   :110.516   Mean   : 0.265   Mean   : 191.78  
 3rd Qu.: 109.823   3rd Qu.:134.460   3rd Qu.: 0.289   3rd Qu.: 255.31  
 Max.   :1323.850   Max.   :643.740   Max.   :23.280   Max.   :1274.78  
 NA's   :3766       NA's   :3765      NA's   :4451     NA's   :3764     
     F2TSH            F2FT4       F2TSH_Comment          F2ery      
 Min.   : 0.030   Min.   : 3.10   Length:6746        Min.   :2.320  
 1st Qu.: 1.490   1st Qu.:14.50   Class :character   1st Qu.:4.430  
 Median : 2.090   Median :16.00   Mode  :character   Median :4.700  
 Mean   : 2.496   Mean   :16.21                      Mean   :4.715  
 3rd Qu.: 2.950   3rd Qu.:17.60                      3rd Qu.:5.000  
 Max.   :69.700   Max.   :92.20                      Max.   :7.000  
 NA's   :2283     NA's   :2270                       NA's   :2236   
```

```
     F2hb          F2hematocr        F2leuc           F2baso     
 Min.   : 74.0   Min.   :22.00   Min.   : 2.10   Min.   :0.000  
 1st Qu.:134.0   1st Qu.:40.00   1st Qu.: 5.00   1st Qu.:0.030  
 Median :141.0   Median :42.00   Median : 5.90   Median :0.050  
 Mean   :141.7   Mean   :41.88   Mean   : 6.18   Mean   :0.047  
 3rd Qu.:150.0   3rd Qu.:44.00   3rd Qu.: 7.10   3rd Qu.:0.060  
 Max.   :183.0   Max.   :55.00   Max.   :63.30   Max.   :0.810  
 NA's   :2236    NA's   :2236    NA's   :2236    NA's   :2302   
   F2baso_pct       F2eosino        F2eosino_pct       F2lympho     
 Min.   :0.000   Min.   :0.0000   Min.   : 0.000   Min.   : 0.330  
 1st Qu.:1.000   1st Qu.:0.1000   1st Qu.: 2.000   1st Qu.: 1.415  
 Median :1.000   Median :0.1500   Median : 3.000   Median : 1.740  
 Mean   :0.792   Mean   :0.1764   Mean   : 2.944   Mean   : 1.854  
 3rd Qu.:1.000   3rd Qu.:0.2200   3rd Qu.: 4.000   3rd Qu.: 2.160  
 Max.   :7.000   Max.   :1.8200   Max.   :30.000   Max.   :53.800  
 NA's   :2250    NA's   :2303     NA's   :2251     NA's   :2303    
  F2lympho_pct       F2mono         F2mono_pct        F2neutro    
 Min.   : 4.00   Min.   :0.1400   Min.   : 1.000   Min.   : 0.34  
 1st Qu.:25.00   1st Qu.:0.4100   1st Qu.: 7.000   1st Qu.: 2.63  
 Median :30.00   Median :0.5000   Median : 8.000   Median : 3.32  
 Mean   :30.66   Mean   :0.5254   Mean   : 8.691   Mean   : 3.56  
 3rd Qu.:36.00   3rd Qu.:0.6200   3rd Qu.:10.000   3rd Qu.: 4.20  
 Max.   :88.00   Max.   :3.8000   Max.   :30.000   Max.   :16.40  
 NA's   :2250    NA's   :2303     NA's   :2250     NA's   :2303   
  F2neutro_pct       F2mcv         F2mch           F2mchc           F2rdw      
 Min.   : 9.00   Min.   : 59   Min.   :16.00   Min.   :259.0   Min.   :11.00  
 1st Qu.:51.00   1st Qu.: 86   1st Qu.:29.20   1st Qu.:332.0   1st Qu.:12.60  
 Median :57.00   Median : 89   Median :30.10   Median :338.0   Median :13.00  
 Mean   :56.91   Mean   : 89   Mean   :30.12   Mean   :338.7   Mean   :13.13  
 3rd Qu.:63.00   3rd Qu.: 92   3rd Qu.:31.10   3rd Qu.:345.0   3rd Qu.:13.50  
 Max.   :92.00   Max.   :112   Max.   :38.20   Max.   :365.0   Max.   :23.40  
 NA's   :2250    NA's   :2236   NA's   :2236    NA's   :2236    NA's   :2237   
    F2plaq          F2mpv           F2pdw           F2cru      
 Min.   : 23.0   Min.   : 7.90   Min.   : 7.70   Min.   : 1002  
 1st Qu.:203.0   1st Qu.: 9.90   1st Qu.:11.20   1st Qu.: 7745  
 Median :238.0   Median :10.50   Median :12.30   Median :11390  
 Mean   :242.9   Mean   :10.59   Mean   :12.62   Mean   :12156  
 3rd Qu.:276.0   3rd Qu.:11.20   3rd Qu.:13.70   3rd Qu.:15570  
 Max.   :807.0   Max.   :14.40   Max.   :25.70   Max.   :50881  
 NA's   :2237    NA's   :2243    NA's   :2243    NA's   :2405   
    F2malb             F2macr           F2Datepr          F2Dayofweek   
 Min.   :    1.0   Min.   :  0.100   Length:6746        Min.   :1.000  
 1st Qu.:    4.0   1st Qu.:  0.300   Class :character   1st Qu.:2.000  
 Median :    7.0   Median :  0.600   Mode  :character   Median :3.000  
 Mean   :   34.5   Mean   :  2.969                      Mean   :2.762  
 3rd Qu.:   15.0   3rd Qu.:  1.200                      3rd Qu.:4.000  
 Max.   :10785.0   Max.   :715.400                      Max.   :7.000  
 NA's   :3550      NA's   :3614                         NA's   :3948   
   F2Avlever       F2Heuredor     F2Cortisol_CAR    F2Cortisol_RT    
 Min.   :1.000   Min.   : 3.100   Min.   :-118.11   Min.   :-172.73  
 1st Qu.:1.000   1st Qu.: 6.300   1st Qu.:   1.78   1st Qu.: -27.30  
 Median :1.000   Median : 7.000   Median :  12.34   Median : -18.08  
 Mean   :1.038   Mean   : 6.959   Mean   :  13.80   Mean   : -20.22  
 3rd Qu.:1.000   3rd Qu.: 7.750   3rd Qu.:  23.94   3rd Qu.: -10.81  
 Max.   :9.000   Max.   :13.000   Max.   : 219.46   Max.   : 192.39  
 NA's   :3948    NA's   :3957     NA's   :4029      NA's   :4041     
 F2Cortisol_AVE   F2Cortisol_PK    F2Cortisol_SBP    F2Cortisol_AUCb   
 Min.   :  0.68   Min.   :  1.09   Min.   :   0.00   Min.   :   187.2  
 1st Qu.: 15.80   1st Qu.: 28.40   1st Qu.:  30.00   1st Qu.: 12872.9  
 Median : 20.21   Median : 38.59   Median :  30.00   Median : 18270.0  
 Mean   : 21.68   Mean   : 42.15   Mean   :  45.02   Mean   : 20827.8  
 3rd Qu.: 25.38   3rd Qu.: 51.27   3rd Qu.:  30.00   3rd Qu.: 25850.3  
 Max.   :118.01   Max.   :294.87   Max.   :1020.00   Max.   :166795.4  
 NA's   :3948     NA's   :3948     NA's   :3952      NA's   :4059      
```

```
 F2Cortisol_AUCg   F2Cortisol_AUCi   F2Amylase_CAR       F2Amylase_RT    
 Min.   :  441.5   Min.   :-128988   Min.   :-653.600   Min.   :-624.30  
 1st Qu.: 9409.2   1st Qu.: -13036   1st Qu.: -51.060   1st Qu.: -11.12  
 Median :11882.8   Median :  -6350   Median : -14.650   Median :  21.22  
 Mean   :12978.4   Mean   :  -7888   Mean   : -34.943   Mean   :  26.86  
 3rd Qu.:15291.6   3rd Qu.:  -1239   3rd Qu.:   3.545   3rd Qu.:  63.89  
 Max.   :92088.9   Max.   :  57188   Max.   : 282.040   Max.   : 536.31  
 NA's   :4094      NA's   :4094      NA's   :4164       NA's   :4147     
 F2Amylase_AVE      F2Amylase_PK     F2Amylase_SBP    F2Amylase_AUCb    
 Min.   :   2.75   Min.   :   2.75   Min.   :   0.0   Min.   :   442.5  
 1st Qu.:  35.40   1st Qu.:  59.65   1st Qu.: 150.0   1st Qu.: 16853.2  
 Median :  60.56   Median : 103.97   Median : 310.0   Median : 37719.0  
 Mean   :  77.37   Mean   : 135.33   Mean   : 432.1   Mean   : 59793.5  
 3rd Qu.:  98.35   3rd Qu.: 169.65   3rd Qu.: 780.0   3rd Qu.: 76799.8  
 Max.   :1038.75   Max.   :1244.39   Max.   :1080.0   Max.   :613148.3  
 NA's   :3956      NA's   :3956      NA's   :3959     NA's   :4160      
 F2Amylase_AUCg   F2Amylase_AUCi        F2SBP            F2DBP      
 Min.   :  1297   Min.   :-512375   Min.   : 77.5   Min.   : 45.0  
 1st Qu.: 31983   1st Qu.: -11056   1st Qu.:114.5   1st Qu.: 70.0  
 Median : 55270   Median :  12999   Median :125.0   Median : 77.0  
 Mean   : 69839   Mean   :   9462   Mean   :126.9   Mean   : 77.4  
 3rd Qu.: 89271   3rd Qu.:  38466   3rd Qu.:137.5   3rd Qu.: 84.0  
 Max.   :383904   Max.   : 264726   Max.   :223.5   Max.   :131.0  
 NA's   :4233     NA's   :4233      NA's   :2245    NA's   :2245   
    F2HRTRTE        F2freqFFQ1        F2freqFFQ2        F2freqFFQ3    
 Min.   : 36.50   Min.   :0.0000   Min.   :0.0000   Min.   :0.0000  
 1st Qu.: 61.50   1st Qu.:0.0000   1st Qu.:0.0000   1st Qu.:0.0000  
 Median : 67.00   Median :0.0357   Median :0.0000   Median :0.0893  
 Mean   : 67.72   Mean   :0.2228   Mean   :0.0654   Mean   :0.2758  
 3rd Qu.: 73.50   3rd Qu.:0.2143   3rd Qu.:0.0000   3rd Qu.:0.5000  
 Max.   :115.50   Max.   :2.5000   Max.   :2.5000   Max.   :2.5000  
 NA's   :2246     NA's   :3099     NA's   :3121     NA's   :3102    
   F2freqFFQ4       F2freqFFQ5       F2freqFFQ6       F2freqFFQ7    
 Min.   :0.0000   Min.   :0.0000   Min.   :0.0000   Min.   :0.0000  
 1st Qu.:0.0000   1st Qu.:0.0000   1st Qu.:0.0000   1st Qu.:0.0893  
 Median :0.0000   Median :0.0000   Median :0.0893   Median :0.2143  
 Mean   :0.0508   Mean   :0.0697   Mean   :0.1164   Mean   :0.3614  
 3rd Qu.:0.0000   3rd Qu.:0.0893   3rd Qu.:0.2143   3rd Qu.:0.5000  
 Max.   :2.5000   Max.   :2.5000   Max.   :2.5000   Max.   :2.5000  
 NA's   :3124     NA's   :3127     NA's   :3119     NA's   :3072    
   F2freqFFQ8       F2freqFFQ9       F2freqFFQ10      F2freqFFQ11    
 Min.   :0.0000   Min.   :0.0000   Min.   :0.0000   Min.   :0.0000  
 1st Qu.:0.0000   1st Qu.:0.0357   1st Qu.:0.0000   1st Qu.:0.0000  
 Median :0.0000   Median :0.2143   Median :0.2143   Median :0.0000  
 Mean   :0.0217   Mean   :0.6083   Mean   :0.5297   Mean   :0.1643  
 3rd Qu.:0.0357   3rd Qu.:1.0000   3rd Qu.:1.0000   3rd Qu.:0.2143  
 Max.   :2.5000   Max.   :2.5000   Max.   :2.5000   Max.   :2.5000  
 NA's   :3107     NA's   :3111     NA's   :3102     NA's   :3121    
  F2freqFFQ12      F2freqFFQ13      F2freqFFQ14      F2freqFFQ15    
 Min.   :0.0000   Min.   :0.0000   Min.   :0.0000   Min.   :0.0000  
 1st Qu.:0.0000   1st Qu.:0.0000   1st Qu.:0.0357   1st Qu.:0.0000  
 Median :0.0000   Median :0.0000   Median :0.0893   Median :0.0893  
 Mean   :0.0463   Mean   :0.1082   Mean   :0.1390   Mean   :0.1261  
 3rd Qu.:0.0000   3rd Qu.:0.0893   3rd Qu.:0.2143   3rd Qu.:0.2143  
 Max.   :2.5000   Max.   :2.5000   Max.   :2.5000   Max.   :1.0000  
 NA's   :3136     NA's   :3126     NA's   :3107     NA's   :3110    
  F2freqFFQ16      F2freqFFQ17      F2freqFFQ18      F2freqFFQ19    
 Min.   :0.0000   Min.   :0.0000   Min.   :0.0000   Min.   :0.0000  
 1st Qu.:0.0000   1st Qu.:0.0357   1st Qu.:0.0000   1st Qu.:0.0357  
 Median :0.0357   Median :0.0893   Median :0.0893   Median :0.0893  
 Mean   :0.0570   Mean   :0.1008   Mean   :0.0928   Mean   :0.1016  
 3rd Qu.:0.0893   3rd Qu.:0.0893   3rd Qu.:0.0893   3rd Qu.:0.0893  
 Max.   :1.0000   Max.   :2.5000   Max.   :2.5000   Max.   :2.5000  
 NA's   :3127     NA's   :3119     NA's   :3126     NA's   :3119    
```

```
  F2freqFFQ20       F2freqFFQ21       F2freqFFQ22       F2freqFFQ23    
 Min.   :0.0000   Min.   :0.0000   Min.   :0.0000   Min.   :0.0000  
 1st Qu.:0.0000   1st Qu.:0.0000   1st Qu.:0.0000   1st Qu.:0.0000  
 Median :0.0000   Median :0.0000   Median :0.0357   Median :0.0000  
 Mean   :0.0327   Mean   :0.0214   Mean   :0.0390   Mean   :0.0131  
 3rd Qu.:0.0357   3rd Qu.:0.0357   3rd Qu.:0.0357   3rd Qu.:0.0000  
 Max.   :2.5000   Max.   :2.5000   Max.   :1.0000   Max.   :1.0000  
 NA's   :3136     NA's   :3130     NA's   :3136     NA's   :3137    
  F2freqFFQ24       F2freqFFQ25       F2freqFFQ26       F2freqFFQ27    
 Min.   :0.0000   Min.   :0.0000   Min.   :0.0000   Min.   :0.0000  
 1st Qu.:0.0000   1st Qu.:0.0000   1st Qu.:0.0000   1st Qu.:0.0000  
 Median :0.0000   Median :0.0357   Median :0.0357   Median :0.0000  
 Mean   :0.0053   Mean   :0.0803   Mean   :0.0497   Mean   :0.0414  
 3rd Qu.:0.0000   3rd Qu.:0.0893   3rd Qu.:0.0893   3rd Qu.:0.0357  
 Max.   :1.0000   Max.   :2.5000   Max.   :1.0000   Max.   :1.0000  
 NA's   :3143     NA's   :3114     NA's   :3123     NA's   :3131    
  F2freqFFQ28       F2freqFFQ29       F2freqFFQ30       F2freqFFQ31    
 Min.   :0.0000   Min.   :0.0000   Min.   :0.0000   Min.   :0.0000  
 1st Qu.:0.0000   1st Qu.:0.0000   1st Qu.:0.0357   1st Qu.:0.0357  
 Median :0.0893   Median :0.0357   Median :0.0893   Median :0.0893  
 Mean   :0.0920   Mean   :0.0472   Mean   :0.1400   Mean   :0.1228  
 3rd Qu.:0.0893   3rd Qu.:0.0893   3rd Qu.:0.2143   3rd Qu.:0.2143  
 Max.   :1.0000   Max.   :1.0000   Max.   :2.5000   Max.   :2.5000  
 NA's   :3118     NA's   :3131     NA's   :3111     NA's   :3118    
  F2freqFFQ32       F2freqFFQ33       F2freqFFQ34       F2freqFFQ35    
 Min.   :0.0000   Min.   :0.0000   Min.   :0.0000   Min.   :0.0000  
 1st Qu.:0.0893   1st Qu.:0.0893   1st Qu.:0.2143   1st Qu.:0.0000  
 Median :0.2143   Median :0.2143   Median :0.5000   Median :0.2143  
 Mean   :0.3232   Mean   :0.2522   Mean   :0.5583   Mean   :0.3704  
 3rd Qu.:0.5000   3rd Qu.:0.2143   3rd Qu.:1.0000   3rd Qu.:0.5000  
 Max.   :2.5000   Max.   :2.5000   Max.   :2.5000   Max.   :2.5000  
 NA's   :3108     NA's   :3103     NA's   :3095     NA's   :3138    
  F2freqFFQ36       F2freqFFQ37       F2freqFFQ38       F2freqFFQ39    
 Min.   :0.0000   Min.   :0.0000   Min.   :0.0000   Min.   :0.0000  
 1st Qu.:0.0000   1st Qu.:0.0000   1st Qu.:0.0000   1st Qu.:0.0000  
 Median :0.0357   Median :0.0357   Median :0.0357   Median :0.0357  
 Mean   :0.0801   Mean   :0.0737   Mean   :0.0936   Mean   :0.1061  
 3rd Qu.:0.0893   3rd Qu.:0.0893   3rd Qu.:0.0893   3rd Qu.:0.0893  
 Max.   :2.5000   Max.   :2.5000   Max.   :2.5000   Max.   :2.5000  
 NA's   :3132     NA's   :3138     NA's   :3123     NA's   :3133    
  F2freqFFQ40       F2freqFFQ41       F2freqFFQ42       F2freqFFQ43    
 Min.   :0.0000   Min.   :0.0000   Min.   :0.0000   Min.   :0.0000  
 1st Qu.:0.0893   1st Qu.:0.0000   1st Qu.:0.0893   1st Qu.:0.0000  
 Median :0.2143   Median :0.0357   Median :0.2143   Median :0.0357  
 Mean   :0.2055   Mean   :0.0543   Mean   :0.2235   Mean   :0.0458  
 3rd Qu.:0.2143   3rd Qu.:0.0893   3rd Qu.:0.2143   3rd Qu.:0.0893  
 Max.   :2.5000   Max.   :2.5000   Max.   :2.5000   Max.   :1.0000  
 NA's   :3102     NA's   :3130     NA's   :3091     NA's   :3141    
  F2freqFFQ44       F2freqFFQ45       F2freqFFQ46       F2freqFFQ47    
 Min.   :0.0000   Min.   :0.0000   Min.   :0.0000   Min.   :0.0000  
 1st Qu.:0.0893   1st Qu.:0.0357   1st Qu.:0.0000   1st Qu.:0.0000  
 Median :0.2143   Median :0.0893   Median :0.0000   Median :0.0357  
 Mean   :0.1791   Mean   :0.1149   Mean   :0.0418   Mean   :0.0566  
 3rd Qu.:0.2143   3rd Qu.:0.2143   3rd Qu.:0.0357   3rd Qu.:0.0893  
 Max.   :2.5000   Max.   :2.5000   Max.   :1.0000   Max.   :1.0000  
 NA's   :3096     NA's   :3126     NA's   :3141     NA's   :3116    
  F2freqFFQ48       F2freqFFQ49       F2freqFFQ50       F2freqFFQ51    
 Min.   :0.0000   Min.   :0.0000   Min.   :0.0000   Min.   :0.0000  
 1st Qu.:0.0000   1st Qu.:0.0893   1st Qu.:0.0000   1st Qu.:0.0000  
 Median :0.0000   Median :0.0893   Median :0.0000   Median :0.0000  
 Mean   :0.0147   Mean   :0.1743   Mean   :0.0226   Mean   :0.1224  
 3rd Qu.:0.0000   3rd Qu.:0.2143   3rd Qu.:0.0000   3rd Qu.:0.0000  
 Max.   :0.5000   Max.   :2.5000   Max.   :2.5000   Max.   :2.5000  
 NA's   :3144     NA's   :3094     NA's   :3141     NA's   :3140    
```

```
  F2freqFFQ52      F2freqFFQ53      F2freqFFQ54      F2freqFFQ55    
 Min.   :0.0000   Min.   :0.0000   Min.   :0.0000   Min.   :0.0000  
 1st Qu.:0.0000   1st Qu.:0.0000   1st Qu.:0.0000   1st Qu.:0.2143  
 Median :0.2143   Median :0.0000   Median :0.0000   Median :0.5000  
 Mean   :0.4007   Mean   :0.0778   Mean   :0.0649   Mean   :0.6922  
 3rd Qu.:1.0000   3rd Qu.:0.0893   3rd Qu.:0.0893   3rd Qu.:1.0000  
 Max.   :2.5000   Max.   :2.5000   Max.   :2.5000   Max.   :2.5000  
 NA's   :3121     NA's   :3140     NA's   :3139     NA's   :3093    
  F2freqFFQ56      F2freqFFQ57      F2freqFFQ58      F2freqFFQ59    
 Min.   :0.0000   Min.   :0.0000   Min.   :0.0000   Min.   :0.0000  
 1st Qu.:0.0000   1st Qu.:0.0000   1st Qu.:0.0000   1st Qu.:0.0000  
 Median :0.2143   Median :0.0893   Median :0.0357   Median :0.0357  
 Mean   :0.3972   Mean   :0.2761   Mean   :0.1684   Mean   :0.1717  
 3rd Qu.:0.5000   3rd Qu.:0.5000   3rd Qu.:0.2143   3rd Qu.:0.2143  
 Max.   :2.5000   Max.   :2.5000   Max.   :2.5000   Max.   :2.5000  
 NA's   :3114     NA's   :3130     NA's   :3133     NA's   :3120    
  F2freqFFQ60      F2freqFFQ61      F2freqFFQ62      F2freqFFQ63    
 Min.   :0.0000   Min.   :0.0000   Min.   :0.0000   Min.   :0.0000  
 1st Qu.:0.0000   1st Qu.:0.0000   1st Qu.:0.0000   1st Qu.:0.0000  
 Median :0.0000   Median :0.0357   Median :0.0357   Median :0.0000  
 Mean   :0.0327   Mean   :0.0954   Mean   :0.0748   Mean   :0.0194  
 3rd Qu.:0.0000   3rd Qu.:0.0893   3rd Qu.:0.0893   3rd Qu.:0.0357  
 Max.   :2.5000   Max.   :2.5000   Max.   :1.0000   Max.   :2.5000  
 NA's   :3143     NA's   :3127     NA's   :3120     NA's   :3136    
  F2freqFFQ64      F2freqFFQ65      F2freqFFQ66      F2freqFFQ67    
 Min.   :0.0000   Min.   :0.0000   Min.   :0.0000   Min.   :0.0000  
 1st Qu.:0.0000   1st Qu.:0.0000   1st Qu.:0.0893   1st Qu.:0.0893  
 Median :0.0357   Median :0.0893   Median :0.2143   Median :0.2143  
 Mean   :0.0683   Mean   :0.1749   Mean   :0.4186   Mean   :0.4818  
 3rd Qu.:0.0893   3rd Qu.:0.2143   3rd Qu.:0.5000   3rd Qu.:1.0000  
 Max.   :2.5000   Max.   :2.5000   Max.   :2.5000   Max.   :2.5000  
 NA's   :3125     NA's   :3125     NA's   :3103     NA's   :3100    
  F2freqFFQ68      F2freqFFQ69      F2freqFFQ70      F2freqFFQ71    
 Min.   :0.0000   Min.   :0.0000   Min.   :0.0000   Min.   :0.0000  
 1st Qu.:0.0000   1st Qu.:0.0000   1st Qu.:0.0000   1st Qu.:0.0000  
 Median :0.0357   Median :0.0000   Median :0.0893   Median :0.0357  
 Mean   :0.0926   Mean   :0.2296   Mean   :0.5137   Mean   :0.1892  
 3rd Qu.:0.0893   3rd Qu.:0.0000   3rd Qu.:1.0000   3rd Qu.:0.2143  
 Max.   :2.5000   Max.   :2.5000   Max.   :2.5000   Max.   :2.5000  
 NA's   :3125     NA's   :3134     NA's   :3132     NA's   :3137    
  F2freqFFQ72      F2freqFFQ73      F2freqFFQ74      F2freqFFQ75    
 Min.   :0.0000   Min.   :0.0000   Min.   :0.0000   Min.   :0.0000  
 1st Qu.:0.0000   1st Qu.:0.2143   1st Qu.:0.0000   1st Qu.:0.0000  
 Median :0.0000   Median :0.5000   Median :0.0000   Median :0.0000  
 Mean   :0.0634   Mean   :0.7217   Mean   :0.0990   Mean   :0.2186  
 3rd Qu.:0.0000   3rd Qu.:1.0000   3rd Qu.:0.0357   3rd Qu.:0.2143  
 Max.   :2.5000   Max.   :2.5000   Max.   :2.5000   Max.   :2.5000  
 NA's   :3145     NA's   :3102     NA's   :3139     NA's   :3134    
  F2freqFFQ76      F2freqFFQ77      F2freqFFQ78     F2freqFFQ79    
 Min.   :0.0000   Min.   :0.0000   Min.   :0.000   Min.   :0.0000  
 1st Qu.:0.0000   1st Qu.:0.0000   1st Qu.:0.000   1st Qu.:0.0000  
 Median :0.0000   Median :0.0000   Median :0.000   Median :0.0000  
 Mean   :0.0809   Mean   :0.0392   Mean   :0.088   Mean   :0.0271  
 3rd Qu.:0.0000   3rd Qu.:0.0000   3rd Qu.:0.000   3rd Qu.:0.0000  
 Max.   :2.5000   Max.   :2.5000   Max.   :2.500   Max.   :2.5000  
 NA's   :3149     NA's   :3154     NA's   :3149    NA's   :3153    
  F2freqFFQ80      F2freqFFQ81     F2freqFFQ82      F2freqFFQ83    
 Min.   :0.0000   Min.   :0.000   Min.   :0.0000   Min.   :0.0000  
 1st Qu.:0.0000   1st Qu.:1.000   1st Qu.:0.0000   1st Qu.:0.0000  
 Median :0.0000   Median :2.500   Median :0.0000   Median :0.0000  
 Mean   :0.0129   Mean   :1.706   Mean   :0.2053   Mean   :0.3659  
 3rd Qu.:0.0000   3rd Qu.:2.500   3rd Qu.:0.0000   3rd Qu.:0.2143  
 Max.   :2.5000   Max.   :2.500   Max.   :2.5000   Max.   :2.5000  
 NA's   :3155     NA's   :3063    NA's   :3131     NA's   :3129    
```

```
  F2freqFFQ84      F2freqFFQ85     F2freqFFQ86      F2freqFFQ87    
 Min.   :0.0000   Min.   :0.000   Min.   :0.0000   Min.   :0.0000  
 1st Qu.:0.0000   1st Qu.:0.000   1st Qu.:0.0000   1st Qu.:0.0000  
 Median :0.0000   Median :0.000   Median :0.0000   Median :0.0000  
 Mean   :0.2804   Mean   :0.059   Mean   :0.1721   Mean   :0.5132  
 3rd Qu.:0.0357   3rd Qu.:0.000   3rd Qu.:0.0893   3rd Qu.:0.5000  
 Max.   :2.5000   Max.   :2.500   Max.   :2.5000   Max.   :2.5000  
 NA's   :3122     NA's   :3132    NA's   :3124     NA's   :3116    
  F2freqFFQ88    F2freqFFQ89     F2freqFFQ90      F2freqFFQ91    
 Min.   :0.0    Min.   :0.000   Min.   :0.0000   Min.   :0.0000  
 1st Qu.:0.0    1st Qu.:1.000   1st Qu.:0.0000   1st Qu.:0.0000  
 Median :0.0    Median :2.500   Median :0.0000   Median :0.0357  
 Mean   :0.2    Mean   :1.782   Mean   :0.1943   Mean   :0.2117  
 3rd Qu.:0.0    3rd Qu.:2.500   3rd Qu.:0.0893   3rd Qu.:0.2143  
 Max.   :2.5    Max.   :2.500   Max.   :2.5000   Max.   :2.5000  
 NA's   :3139   NA's   :3091    NA's   :3124     NA's   :3117    
  F2freqFFQ92      F2freqFFQ93       F2freqFFQ94      F2freqFFQ95    
 Min.   :0.0000   Min.   :0.0000   Min.   :0.0000   Min.   :0.0000  
 1st Qu.:0.0000   1st Qu.:0.0893   1st Qu.:0.0000   1st Qu.:0.0357  
 Median :0.0357   Median :0.5000   Median :0.0000   Median :0.2143  
 Mean   :0.2530   Mean   :0.9384   Mean   :0.1569   Mean   :0.4063  
 3rd Qu.:0.2143   3rd Qu.:2.5000   3rd Qu.:0.0893   3rd Qu.:0.5000  
 Max.   :2.5000   Max.   :2.5000   Max.   :2.5000   Max.   :2.5000  
 NA's   :3127     NA's   :3098     NA's   :3115     NA's   :3098    
  F2freqFFQ96      F2freqFFQ97      F2FFQ1amount      F2FFQ2amount   
 Min.   :0.0000   Min.   :0.0000   Min.   :  0.000   Min.   :  0.00  
 1st Qu.:0.0000   1st Qu.:0.0000   1st Qu.:  0.000   1st Qu.:  0.00  
 Median :0.0000   Median :0.0000   Median :  3.214   Median :  0.00  
 Mean   :0.0388   Mean   :0.0362   Mean   : 37.811   Mean   : 11.77  
 3rd Qu.:0.0000   3rd Qu.:0.0000   3rd Qu.: 38.571   3rd Qu.:  0.00  
 Max.   :2.5000   Max.   :2.5000   Max.   :675.000   Max.   :675.00  
 NA's   :3125     NA's   :3124     NA's   :3099      NA's   :3121    
  F2FFQ3amount     F2FFQ4amount      F2FFQ5amount      F2FFQ6amount    
 Min.   :  0.00   Min.   :  0.000   Min.   :  0.000   Min.   :  0.000  
 1st Qu.:  0.00   1st Qu.:  0.000   1st Qu.:  0.000   1st Qu.:  0.000  
 Median : 16.07   Median :  0.000   Median :  0.000   Median :  3.571  
 Mean   : 49.26   Mean   :  5.413   Mean   :  6.470   Mean   :  6.377  
 3rd Qu.: 57.86   3rd Qu.:  0.000   3rd Qu.:  4.464   3rd Qu.:  8.571  
 Max.   :675.00   Max.   :500.000   Max.   :375.000   Max.   :150.000  
 NA's   :3102     NA's   :3124      NA's   :3127      NA's   :3119     
  F2FFQ7amount      F2FFQ8amount       F2FFQ9amount     F2FFQ10amount  
 Min.   :  0.000   Min.   :   0.000   Min.   :  0.000   Min.   :  0.00  
 1st Qu.:  8.036   1st Qu.:   0.000   1st Qu.:  2.679   1st Qu.:  0.00  
 Median : 19.286   Median :   0.000   Median : 16.071   Median : 12.86  
 Mean   : 30.306   Mean   :   7.878   Mean   : 38.650   Mean   : 35.94  
 3rd Qu.: 45.000   3rd Qu.:  12.500   3rd Qu.: 50.000   3rd Qu.: 50.00  
 Max.   :300.000   Max.   :1312.500   Max.   :187.500   Max.   :225.00  
 NA's   :3072      NA's   :3107       NA's   :3111      NA's   :3102    
 F2FFQ11amount     F2FFQ12amount     F2FFQ13amount     F2FFQ14amount    
 Min.   :  0.000   Min.   :  0.000   Min.   : 0.0000   Min.   :  0.000  
 1st Qu.:  0.000   1st Qu.:  0.000   1st Qu.: 0.0000   1st Qu.:  4.643  
 Median :  0.000   Median :  0.000   Median : 0.0000   Median : 12.857  
 Mean   :  9.208   Mean   :  1.495   Mean   : 1.9962   Mean   : 19.836  
 3rd Qu.: 10.714   3rd Qu.:  0.000   3rd Qu.: 0.8036   3rd Qu.: 27.857  
 Max.   :187.500   Max.   :112.500   Max.   :67.5000   Max.   :500.000  
 NA's   :3121      NA's   :3136      NA's   :3126      NA's   :3107     
 F2FFQ15amount     F2FFQ16amount     F2FFQ17amount     F2FFQ18amount    
 Min.   :  0.000   Min.   :  0.000   Min.   :  0.000   Min.   :  0.000  
 1st Qu.:  0.000   1st Qu.:  0.000   1st Qu.:  4.464   1st Qu.:  0.000  
 Median :  8.929   Median :  3.571   Median : 11.161   Median :  9.821  
 Mean   : 13.999   Mean   :  6.535   Mean   : 16.049   Mean   : 11.203  
 3rd Qu.: 21.429   3rd Qu.:  8.929   3rd Qu.: 20.089   3rd Qu.: 14.732  
 Max.   :150.000   Max.   :150.000   Max.   :562.500   Max.   :275.000  
 NA's   :3110      NA's   :3127      NA's   :3119      NA's   :3126     
```

```
 F2FFQ19amount      F2FFQ20amount     F2FFQ21amount     F2FFQ22amount   
 Min.   :  0.0000   Min.   :  0.000   Min.   :  0.000   Min.   :  0.000  
 1st Qu.:  0.8929   1st Qu.:  0.000   1st Qu.:  0.000   1st Qu.:  0.000  
 Median :  4.4643   Median :  0.000   Median :  0.000   Median :  1.429  
 Mean   :  5.7220   Mean   :  2.756   Mean   :  2.037   Mean   :  3.025  
 3rd Qu.:  6.6964   3rd Qu.:  2.857   3rd Qu.:  2.143   3rd Qu.:  4.107  
 Max.   :187.5000   Max.   :200.000   Max.   :275.000   Max.   :120.000  
 NA's   :3119       NA's   :3136      NA's   :3130      NA's   :3136     
 F2FFQ23amount    F2FFQ24amount     F2FFQ25amount     F2FFQ26amount   
 Min.   :  0.00   Min.   : 0.0000   Min.   :  0.000   Min.   :  0.000  
 1st Qu.:  0.00   1st Qu.: 0.0000   1st Qu.:  0.000   1st Qu.:  0.000  
 Median :  0.00   Median : 0.0000   Median :  8.036   Median :  5.357  
 Mean   :  1.31   Mean   : 0.3024   Mean   : 12.681   Mean   :  7.702  
 3rd Qu.:  0.00   3rd Qu.: 0.0000   3rd Qu.: 13.393   3rd Qu.: 13.393  
 Max.   :100.00   Max.   :50.0000   Max.   :375.000   Max.   :150.000  
 NA's   :3137     NA's   :3143      NA's   :3114      NA's   :3123     
 F2FFQ27amount     F2FFQ28amount    F2FFQ29amount    F2FFQ30amount    
 Min.   :  0.000   Min.   :  0.00   Min.   : 0.000   Min.   :  0.000  
 1st Qu.:  0.000   1st Qu.:  0.00   1st Qu.: 0.000   1st Qu.:  5.357  
 Median :  0.000   Median : 13.39   Median : 1.786   Median : 13.393  
 Mean   :  3.521   Mean   : 14.19   Mean   : 2.600   Mean   : 23.302  
 3rd Qu.:  4.286   3rd Qu.: 20.09   3rd Qu.: 4.464   3rd Qu.: 32.143  
 Max.   :120.000   Max.   :150.00   Max.   :75.000   Max.   :562.500  
 NA's   :3131      NA's   :3118     NA's   :3131     NA's   :3111     
 F2FFQ31amount     F2FFQ32amount     F2FFQ33amount     F2FFQ34amount   
 Min.   :  0.000   Min.   :  0.000   Min.   :  0.000   Min.   :  0.00  
 1st Qu.:  7.143   1st Qu.:  8.036   1st Qu.:  6.696   1st Qu.: 10.71  
 Median : 17.857   Median : 19.286   Median : 16.071   Median : 25.00  
 Mean   : 27.010   Mean   : 31.347   Mean   : 20.873   Mean   : 33.63  
 3rd Qu.: 42.857   3rd Qu.: 45.000   3rd Qu.: 24.643   3rd Qu.: 50.00  
 Max.   :750.000   Max.   :337.500   Max.   :287.500   Max.   :187.50  
 NA's   :3118      NA's   :3108      NA's   :3103      NA's   :3095    
 F2FFQ35amount    F2FFQ36amount     F2FFQ37amount     F2FFQ38amount    
 Min.   : 0.000   Min.   :  0.000   Min.   :  0.000   Min.   :   0.00  
 1st Qu.: 0.000   1st Qu.:  0.000   1st Qu.:  0.000   1st Qu.:   0.00  
 Median : 3.214   Median :  2.143   Median :  6.429   Median :  10.71  
 Mean   : 6.225   Mean   :  5.003   Mean   : 14.696   Mean   :  29.40  
 3rd Qu.: 7.500   3rd Qu.:  5.357   3rd Qu.: 16.071   3rd Qu.:  26.79  
 Max.   :62.500   Max.   :150.000   Max.   :675.000   Max.   :1125.00  
 NA's   :3138     NA's   :3132      NA's   :3138      NA's   :3123     
 F2FFQ39amount     F2FFQ40amount     F2FFQ41amount     F2FFQ42amount   
 Min.   :  0.000   Min.   :  0.000   Min.   :  0.000   Min.   :  0.00  
 1st Qu.:  0.000   1st Qu.:  8.929   1st Qu.:  0.000   1st Qu.: 26.79  
 Median :  8.929   Median : 21.429   Median :  4.286   Median : 42.86  
 Mean   : 28.357   Mean   : 27.920   Mean   :  7.117   Mean   : 60.64  
 3rd Qu.: 33.482   3rd Qu.: 32.143   3rd Qu.: 10.714   3rd Qu.: 64.29  
 Max.   :937.500   Max.   :375.000   Max.   :300.000   Max.   :750.00  
 NA's   :3133      NA's   :3102      NA's   :3130      NA's   :3091    
 F2FFQ43amount     F2FFQ44amount     F2FFQ45amount     F2FFQ46amount   
 Min.   :  0.000   Min.   :  0.000   Min.   :  0.000   Min.   : 0.000  
 1st Qu.:  0.000   1st Qu.:  5.357   1st Qu.:  2.857   1st Qu.: 0.000  
 Median :  5.000   Median :  8.036   Median :  7.143   Median : 0.000  
 Mean   :  7.899   Mean   : 11.330   Mean   : 10.181   Mean   : 1.414  
 3rd Qu.: 12.500   3rd Qu.: 12.857   3rd Qu.: 17.143   3rd Qu.: 1.607  
 Max.   :270.000   Max.   :225.000   Max.   :300.000   Max.   :45.000  
 NA's   :3141      NA's   :3096      NA's   :3126      NA's   :3141    
 F2FFQ47amount     F2FFQ48amount    F2FFQ49amount     F2FFQ50amount    
 Min.   :  0.000   Min.   : 0.000   Min.   :  0.000   Min.   :  0.000  
 1st Qu.:  0.000   1st Qu.: 0.000   1st Qu.:  5.357   1st Qu.:  0.000  
 Median :  8.929   Median : 0.000   Median : 10.714   Median :  0.000  
 Mean   : 16.015   Mean   : 2.451   Mean   : 16.183   Mean   :  2.404  
 3rd Qu.: 22.321   3rd Qu.: 0.000   3rd Qu.: 21.429   3rd Qu.:  0.000  
 Max.   :250.000   Max.   :75.000   Max.   :375.000   Max.   :375.000  
 NA's   :3116      NA's   :3144     NA's   :3094      NA's   :3141     
```

```
 F2FFQ51amount   F2FFQ52amount    F2FFQ53amount    F2FFQ54amount   
 Min.   : 0.00   Min.   : 0.000   Min.   : 0.000   Min.   : 0.0000  
 1st Qu.: 0.00   1st Qu.: 0.000   1st Qu.: 0.000   1st Qu.: 0.0000  
 Median : 0.00   Median : 1.500   Median : 0.000   Median : 0.0000  
 Mean   : 1.09   Mean   : 3.535   Mean   : 1.428   Mean   : 0.5918  
 3rd Qu.: 0.00   3rd Qu.: 7.000   3rd Qu.: 1.786   3rd Qu.: 0.6250  
 Max.   :37.50   Max.   :37.500   Max.   :57.500   Max.   :25.0000  
 NA's   :3140    NA's   :3121     NA's   :3140     NA's   :3139     
 F2FFQ55amount    F2FFQ56amount    F2FFQ57amount     F2FFQ58amount    
 Min.   :  0.00   Min.   :  0.00   Min.   :  0.000   Min.   :  0.000  
 1st Qu.: 27.86   1st Qu.:  0.00   1st Qu.:  0.000   1st Qu.:  0.000  
 Median : 65.00   Median : 32.14   Median :  8.929   Median :  5.357  
 Mean   :101.98   Mean   : 66.19   Mean   : 31.262   Mean   : 21.789  
 3rd Qu.:130.00   3rd Qu.: 75.00   3rd Qu.: 36.607   3rd Qu.: 23.571  
 Max.   :487.50   Max.   :562.50   Max.   :375.000   Max.   :562.500  
 NA's   :3093     NA's   :3114     NA's   :3130      NA's   :3133     
 F2FFQ59amount     F2FFQ60amount     F2FFQ61amount     F2FFQ62amount    
 Min.   :  0.000   Min.   :  0.000   Min.   :  0.000   Min.   :  0.000  
 1st Qu.:  0.000   1st Qu.:  0.000   1st Qu.:  0.000   1st Qu.:  0.000  
 Median :  3.214   Median :  0.000   Median :  1.429   Median :  6.429  
 Mean   : 16.021   Mean   :  5.556   Mean   :  4.008   Mean   : 10.899  
 3rd Qu.: 19.286   3rd Qu.:  0.000   3rd Qu.:  3.571   3rd Qu.: 13.393  
 Max.   :337.500   Max.   :562.500   Max.   :100.000   Max.   :225.000  
 NA's   :3120      NA's   :3143      NA's   :3127      NA's   :3120     
 F2FFQ63amount     F2FFQ64amount     F2FFQ65amount     F2FFQ66amount   
 Min.   :  0.000   Min.   :  0.000   Min.   :  0.000   Min.   : 0.000  
 1st Qu.:  0.000   1st Qu.:  0.000   1st Qu.:  0.000   1st Qu.: 1.339  
 Median :  0.000   Median :  3.571   Median :  1.339   Median : 4.286  
 Mean   :  1.905   Mean   :  6.665   Mean   :  3.798   Mean   : 8.440  
 3rd Qu.:  3.571   3rd Qu.:  8.929   3rd Qu.:  4.000   3rd Qu.:12.500  
 Max.   :125.000   Max.   :375.000   Max.   :100.000   Max.   :75.000  
 NA's   :3136      NA's   :3125      NA's   :3125      NA's   :3103    
 F2FFQ67amount    F2FFQ68amount     F2FFQ70amount     F2FFQ71amount   
 Min.   : 0.000   Min.   :  0.000   Min.   : 0.0000   Min.   : 0.000  
 1st Qu.: 1.786   1st Qu.:  0.000   1st Qu.: 0.0000   1st Qu.: 0.000  
 Median : 4.286   Median :  4.286   Median : 0.3571   Median : 0.250  
 Mean   :10.068   Mean   : 11.590   Mean   : 4.1759   Mean   : 1.663  
 3rd Qu.:20.000   3rd Qu.: 10.714   3rd Qu.: 8.0000   3rd Qu.: 2.143  
 Max.   :75.000   Max.   :375.000   Max.   :30.0000   Max.   :37.500  
 NA's   :3100     NA's   :3125      NA's   :3132      NA's   :3137    
 F2FFQ72amount     F2FFQ73amount    F2FFQ74amount     F2FFQ75amount   
 Min.   : 0.0000   Min.   : 0.000   Min.   : 0.0000   Min.   : 0.000  
 1st Qu.: 0.0000   1st Qu.: 2.143   1st Qu.: 0.0000   1st Qu.: 0.000  
 Median : 0.0000   Median : 5.000   Median : 0.0000   Median : 0.000  
 Mean   : 0.5617   Mean   : 6.567   Mean   : 0.8364   Mean   : 1.895  
 3rd Qu.: 0.0000   3rd Qu.:10.000   3rd Qu.: 0.3571   3rd Qu.: 2.143  
 Max.   :25.0000   Max.   :37.500   Max.   :37.5000   Max.   :37.500  
 NA's   :3145      NA's   :3102     NA's   :3139      NA's   :3134    
 F2FFQ81amount  F2FFQ82amount    F2FFQ83amount     F2FFQ84amount    
 Min.   :  0    Min.   :  0.00   Min.   :  0.000   Min.   : 0.0000  
 1st Qu.: 80    1st Qu.:  0.00   1st Qu.:  0.000   1st Qu.: 0.0000  
 Median :200    Median :  0.00   Median :  0.000   Median : 0.0000  
 Mean   :155    Mean   :  6.24   Mean   : 11.361   Mean   : 3.4914  
 3rd Qu.:200    3rd Qu.:  0.00   3rd Qu.:  4.018   3rd Qu.: 0.4286  
 Max.   :300    Max.   :112.50   Max.   :112.500   Max.   :45.0000  
 NA's   :3063   NA's   :3131     NA's   :3129      NA's   :3122     
 F2FFQ85amount     F2FFQ86amount     F2FFQ87amount  F2FFQ88amount   
 Min.   :  0.000   Min.   :  0.00   Min.   :  0    Min.   :  0.00  
 1st Qu.:  0.000   1st Qu.:  0.00   1st Qu.:  0    1st Qu.:  0.00  
 Median :  0.000   Median :  0.00   Median :  0    Median :  0.00  
 Mean   :  9.184   Mean   : 27.20   Mean   :132    Mean   : 51.23  
 3rd Qu.:  0.000   3rd Qu.: 13.39   3rd Qu.:150    3rd Qu.:  0.00  
 Max.   :562.500   Max.   :562.50   Max.   :750    Max.   :750.00  
 NA's   :3132      NA's   :3124     NA's   :3116   NA's   :3139    
```

```
 F2FFQ89amount   F2FFQ90amount    F2FFQ91amount     F2FFQ92amount   
 Min.   :  0.0   Min.   :  0.00   Min.   :  0.000   Min.   :  0.000  
 1st Qu.:200.0   1st Qu.:  0.00   1st Qu.:  0.000   1st Qu.:  0.000  
 Median :500.0   Median :  0.00   Median :  7.143   Median :  3.571  
 Mean   :466.7   Mean   : 46.47   Mean   : 44.105   Mean   : 54.173  
 3rd Qu.:750.0   3rd Qu.: 26.79   3rd Qu.: 42.857   3rd Qu.: 42.857  
 Max.   :750.0   Max.   :750.00   Max.   :750.000   Max.   :750.000  
 NA's   :3091    NA's   :3124     NA's   :3117      NA's   :3127     
 F2FFQ93amount    F2FFQ94amount     F2FFQ95amount     F2FFQ96amount    
 Min.   :  0.00   Min.   :   0.00   Min.   :  0.000   Min.   :  0.000  
 1st Qu.: 17.86   1st Qu.:   0.00   1st Qu.:  5.357   1st Qu.:  0.000  
 Median :150.00   Median :   0.00   Median : 32.143   Median :  0.000  
 Mean   :230.60   Mean   :  52.36   Mean   : 70.853   Mean   :  2.036  
 3rd Qu.:500.00   3rd Qu.:  26.79   3rd Qu.: 75.000   3rd Qu.:  0.000  
 Max.   :750.00   Max.   :1125.00   Max.   :562.500   Max.   :187.500  
 NA's   :3098     NA's   :3115      NA's   :3098      NA's   :3125     
 F2FFQ97amount       F2numitems      F2sumtot1     F2sumtot3   
 Min.   :  0.000   Min.   : 1.00   Min.   :   0   Min.   :   0  
 1st Qu.:  0.000   1st Qu.:42.00   1st Qu.:1240   1st Qu.:1178  
 Median :  0.000   Median :50.00   Median :1594   Median :1518  
 Mean   :  3.451   Mean   :49.31   Mean   :1664   Mean   :1591  
 3rd Qu.:  0.000   3rd Qu.:57.00   3rd Qu.:1995   3rd Qu.:1914  
 Max.   :375.000   Max.   :91.00   Max.   :7865   Max.   :7734  
 NA's   :3124      NA's   :3014    NA's   :2995   NA's   :2995  
   F2sumprot1       F2sumpveg1       F2sumpani1       F2sumgluc1    
 Min.   :   0.0   Min.   :  0.00   Min.   :   0.0   Min.   :   0.0  
 1st Qu.: 197.4   1st Qu.: 54.40   1st Qu.: 129.0   1st Qu.: 548.2  
 Median : 258.8   Median : 73.80   Median : 175.9   Median : 736.9  
 Mean   : 275.0   Mean   : 82.07   Mean   : 192.9   Mean   : 801.1  
 3rd Qu.: 334.6   3rd Qu.:103.20   3rd Qu.: 239.0   3rd Qu.:1004.0  
 Max.   :1677.8   Max.   :368.60   Max.   :1308.7   Max.   :3618.8  
 NA's   :3017     NA's   :3017     NA's   :3017     NA's   :3017    
   F2sumgsuc1       F2sumgpol1       F2sumlipi1       F2sumlsat1    
 Min.   :   0.0   Min.   :   0.0   Min.   :   0.0   Min.   :   0.0  
 1st Qu.: 247.6   1st Qu.: 250.3   1st Qu.: 442.8   1st Qu.: 152.9  
 Median : 356.1   Median : 350.3   Median : 595.2   Median : 212.9  
 Mean   : 393.6   Mean   : 405.1   Mean   : 630.5   Mean   : 233.1  
 3rd Qu.: 492.4   3rd Qu.: 516.1   3rd Qu.: 768.1   3rd Qu.: 292.0  
 Max.   :2372.5   Max.   :1845.1   Max.   :3222.6   Max.   :1274.1  
 NA's   :3017     NA's   :3017     NA's   :3017     NA's   :3017    
   F2sumlmon1       F2sumlpol1       F2sumfibr1      F2sumchol1    
 Min.   :   0.0   Min.   :  0.00   Min.   : 0.00   Min.   :   0.0  
 1st Qu.: 176.2   1st Qu.: 58.50   1st Qu.: 8.90   1st Qu.: 192.5  
 Median : 239.1   Median : 79.80   Median :12.90   Median : 267.3  
 Mean   : 256.7   Mean   : 86.86   Mean   :14.76   Mean   : 292.8  
 3rd Qu.: 315.3   3rd Qu.:106.50   3rd Qu.:18.55   3rd Qu.: 356.6  
 Max.   :1420.9   Max.   :510.80   Max.   :87.70   Max.   :2255.2  
 NA's   :3017     NA's   :3017     NA's   :2995    NA's   :2995    
   F2sumalco        F2sumcalc1       F2sumfer1         F2sumret1      
 Min.   :   0.0   Min.   :   0.0   Min.   : 0.000   Min.   :    0.0  
 1st Qu.:   8.3   1st Qu.: 597.0   1st Qu.: 7.100   1st Qu.:  188.9  
 Median :  32.6   Median : 851.9   Median : 9.200   Median :  309.9  
 Mean   :  69.4   Mean   : 944.1   Mean   : 9.696   Mean   :  469.6  
 3rd Qu.:  92.4   3rd Qu.:1163.3   3rd Qu.:11.700   3rd Qu.:  518.0  
 Max.   :1022.0   Max.   :5412.2   Max.   :53.600   Max.   :18437.9  
 NA's   :3017     NA's   :2995     NA's   :2995     NA's   :2995     
   F2sumcaro1      F2sumvitd1       F2sumeau1        F2sumprot3    
 Min.   :    0   Min.   : 0.000   Min.   : 0.000   Min.   :   0.0  
 1st Qu.: 2028   1st Qu.: 1.300   1st Qu.: 4.900   1st Qu.: 186.5  
 Median : 3119   Median : 2.300   Median :10.500   Median : 243.3  
 Mean   : 3956   Mean   : 2.729   Mean   : 8.531   Mean   : 258.4  
 3rd Qu.: 4924   3rd Qu.: 3.400   3rd Qu.:10.600   3rd Qu.: 311.6  
 Max.   :45879   Max.   :38.800   Max.   :31.500   Max.   :1521.3  
 NA's   :2995    NA's   :2995     NA's   :2995     NA's   :2995    
```

```
  F2sumpveg3       F2sumpani3       F2sumgluc3       F2sumgsuc3    
 Min.   :  0.00   Min.   :   0.0   Min.   :   0.0   Min.   :   0.0  
 1st Qu.: 50.10   1st Qu.: 123.3   1st Qu.: 513.5   1st Qu.: 234.7  
 Median : 67.30   Median : 168.0   Median : 686.0   Median : 341.9  
 Mean   : 75.28   Mean   : 183.0   Mean   : 749.1   Mean   : 377.9  
 3rd Qu.: 94.45   3rd Qu.: 225.8   3rd Qu.: 938.0   3rd Qu.: 477.4  
 Max.   :334.00   Max.   :1201.8   Max.   :3336.1   Max.   :2372.4  
 NA's   :2995     NA's   :2995     NA's   :2995     NA's   :2995    
  F2sumgpol3       F2sumlipi3       F2sumlsat3       F2sumlmon3    
 Min.   :   0.0   Min.   :   0.0   Min.   :   0.0   Min.   :   0.0  
 1st Qu.: 231.7   1st Qu.: 419.0   1st Qu.: 144.5   1st Qu.: 164.8  
 Median : 322.2   Median : 549.8   Median : 198.3   Median : 223.7  
 Mean   : 368.7   Mean   : 583.9   Mean   : 213.9   Mean   : 239.3  
 3rd Qu.: 462.6   3rd Qu.: 710.8   3rd Qu.: 266.4   3rd Qu.: 292.2  
 Max.   :1716.8   Max.   :2996.6   Max.   :1253.2   Max.   :1419.9  
 NA's   :2995     NA's   :2995     NA's   :2995     NA's   :2995    
  F2sumlpol3       F2sumcalc3       F2sumfer3       F2sumret3      
 Min.   :  0.00   Min.   :   0.0   Min.   : 0.00   Min.   :    0.0  
 1st Qu.: 54.20   1st Qu.: 589.4   1st Qu.: 6.60   1st Qu.:  188.9  
 Median : 73.30   Median : 844.3   Median : 8.60   Median :  309.9  
 Mean   : 79.32   Mean   : 936.4   Mean   : 9.06   Mean   :  469.6  
 3rd Qu.: 96.75   3rd Qu.:1160.3   3rd Qu.:11.00   3rd Qu.:  518.0  
 Max.   :401.40   Max.   :5406.3   Max.   :53.60   Max.   :18437.9  
 NA's   :2995     NA's   :2995     NA's   :2995    NA's   :2995     
  F2sumcaro3      F2sumvitd3       F2sumfibr3      F2sumchol3    
 Min.   :    0   Min.   : 0.000   Min.   : 0.00   Min.   :   0.0  
 1st Qu.: 2028   1st Qu.: 1.300   1st Qu.: 8.90   1st Qu.: 192.5  
 Median : 3119   Median : 2.300   Median :12.90   Median : 267.3  
 Mean   : 3956   Mean   : 2.729   Mean   :14.76   Mean   : 292.8  
 3rd Qu.: 4924   3rd Qu.: 3.400   3rd Qu.:18.55   3rd Qu.: 356.6  
 Max.   :45879   Max.   :38.800   Max.   :87.70   Max.   :2255.2  
 NA's   :2995    NA's   :2995     NA's   :2995    NA's   :2995    
  F2pct_prot1     F2pct_pveg1      F2pct_pani1     F2pct_gluc1   
 Min.   : 0.00   Min.   : 0.000   Min.   : 0.00   Min.   : 0.00  
 1st Qu.:13.40   1st Qu.: 3.700   1st Qu.: 8.60   1st Qu.:39.10  
 Median :15.40   Median : 4.400   Median :10.80   Median :45.10  
 Mean   :15.81   Mean   : 4.556   Mean   :11.25   Mean   :44.93  
 3rd Qu.:17.60   3rd Qu.: 5.300   3rd Qu.:13.30   3rd Qu.:51.20  
 Max.   :61.20   Max.   :12.000   Max.   :61.20   Max.   :84.70  
 NA's   :3001    NA's   :3001     NA's   :3001    NA's   :3001   
  F2pct_gsuc1    F2pct_gpol1     F2pct_lipi1     F2pct_lsat1     F2pct_lmon1   
 Min.   : 0.0   Min.   : 0.00   Min.   : 0.00   Min.   : 0.00   Min.   : 0.00  
 1st Qu.:17.2   1st Qu.:16.50   1st Qu.:30.50   1st Qu.:10.50   1st Qu.:11.80  
 Median :22.2   Median :21.00   Median :35.20   Median :12.70   Median :14.10  
 Mean   :23.0   Mean   :21.79   Mean   :35.17   Mean   :12.85   Mean   :14.43  
 3rd Qu.:27.8   3rd Qu.:26.30   3rd Qu.:39.70   3rd Qu.:15.00   3rd Qu.:16.60  
 Max.   :82.2   Max.   :65.30   Max.   :68.10   Max.   :37.30   Max.   :41.50  
 NA's   :3001   NA's   :3001    NA's   :3001    NA's   :3001    NA's   :3001   
  F2pct_lpol1    F2pct_fibr1      F2pct_chol1      F2pct_alco1    
 Min.   : 0.0   Min.   :0.0000   Min.   :  0.00   Min.   : 0.000  
 1st Qu.: 3.8   1st Qu.:0.6000   1st Qu.: 13.60   1st Qu.: 0.400  
 Median : 4.6   Median :0.8000   Median : 17.00   Median : 1.900  
 Mean   : 4.8   Mean   :0.8808   Mean   : 17.83   Mean   : 4.089  
 3rd Qu.: 5.5   3rd Qu.:1.1000   3rd Qu.: 20.90   3rd Qu.: 5.400  
 Max.   :17.0   Max.   :3.0000   Max.   :100.80   Max.   :97.100  
 NA's   :3001   NA's   :3001     NA's   :3001     NA's   :3001    
  F2pct_prot3     F2pct_pveg3      F2pct_pani3     F2pct_gluc3   
 Min.   : 4.10   Min.   : 0.000   Min.   : 0.00   Min.   : 2.50  
 1st Qu.:14.10   1st Qu.: 4.000   1st Qu.: 9.00   1st Qu.:41.40  
 Median :16.20   Median : 4.700   Median :11.30   Median :47.00  
 Mean   :16.59   Mean   : 4.773   Mean   :11.81   Mean   :47.02  
 3rd Qu.:18.60   3rd Qu.: 5.500   3rd Qu.:14.10   3rd Qu.:52.90  
 Max.   :61.20   Max.   :12.400   Max.   :61.20   Max.   :99.60  
 NA's   :3002    NA's   :3002     NA's   :3002    NA's   :3002   
```

```
  F2pct_gsuc3     F2pct_gpol3     F2pct_lipi3     F2pct_lsat3   
 Min.   : 0.00   Min.   : 0.00   Min.   : 0.00   Min.   : 0.00  
 1st Qu.:18.20   1st Qu.:17.50   1st Qu.:32.00   1st Qu.:11.00  
 Median :23.10   Median :22.00   Median :36.90   Median :13.30  
 Mean   :24.06   Mean   :22.80   Mean   :36.89   Mean   :13.48  
 3rd Qu.:28.90   3rd Qu.:27.52   3rd Qu.:41.70   3rd Qu.:15.70  
 Max.   :99.60   Max.   :65.70   Max.   :69.10   Max.   :37.30  
 NA's   :3002    NA's   :3002    NA's   :3002    NA's   :3002   
  F2pct_lmon3     F2pct_lpol3      F2pct_fibr3      F2pct_chol3    
 Min.   : 0.00   Min.   : 0.000   Min.   :0.0000   Min.   :  0.00  
 1st Qu.:12.40   1st Qu.: 4.000   1st Qu.:0.7000   1st Qu.: 14.20  
 Median :14.80   Median : 4.800   Median :0.9000   Median : 17.80  
 Mean   :15.13   Mean   : 5.032   Mean   :0.9184   Mean   : 18.71  
 3rd Qu.:17.40   3rd Qu.: 5.800   3rd Qu.:1.1000   3rd Qu.: 21.80  
 Max.   :42.80   Max.   :18.100   Max.   :3.0000   Max.   :100.80  
 NA's   :3002    NA's   :3002     NA's   :3002     NA's   :3002    
    F2Fruits        F2Fruits_OK     F2Vegetables     F2Vegetables_OK
 Min.   : 0.000   Min.   :0.000   Min.   : 0.000   Min.   :0.00    
 1st Qu.: 0.964   1st Qu.:0.000   1st Qu.: 0.982   1st Qu.:0.00    
 Median : 1.714   Median :0.000   Median : 1.446   Median :0.00    
 Mean   : 2.152   Mean   :0.451   Mean   : 1.666   Mean   :0.09    
 3rd Qu.: 2.929   3rd Qu.:1.000   3rd Qu.: 2.054   3rd Qu.:0.00    
 Max.   :16.000   Max.   :1.000   Max.   :15.000   Max.   :1.00    
 NA's   :3169     NA's   :3169    NA's   :3164     NA's   :3164    
     F2Meat          F2Meat_OK         F2Fish          F2Fish_OK     
 Min.   : 0.000   Min.   :0.000   Min.   : 0.000   Min.   :0.0000  
 1st Qu.: 2.875   1st Qu.:0.000   1st Qu.: 0.875   1st Qu.:0.0000  
 Median : 4.500   Median :1.000   Median : 1.500   Median :1.0000  
 Mean   : 5.067   Mean   :0.589   Mean   : 1.824   Mean   :0.6952  
 3rd Qu.: 6.500   3rd Qu.:1.000   3rd Qu.: 2.375   3rd Qu.:1.0000  
 Max.   :78.875   Max.   :1.000   Max.   :28.000   Max.   :1.0000  
 NA's   :3163     NA's   :3163    NA's   :3153     NA's   :3153    
    F2Fish2         F2Fish2_OK        F2Dairy          F2Dairy_OK   
 Min.   : 0.000   Min.   :0.0000   Min.   : 0.000   Min.   :0.000  
 1st Qu.: 0.500   1st Qu.:0.0000   1st Qu.: 0.643   1st Qu.:0.000  
 Median : 0.875   Median :0.0000   Median : 1.161   Median :0.000  
 Mean   : 1.200   Mean   :0.4551   Mean   : 1.390   Mean   :0.082  
 3rd Qu.: 1.750   3rd Qu.:1.0000   3rd Qu.: 1.804   3rd Qu.:0.000  
 Max.   :17.750   Max.   :1.0000   Max.   :11.536   Max.   :1.000  
 NA's   :3136     NA's   :3136     NA's   :3174     NA's   :3174   
    F2Nb_OK         F2Cat_OK       F2Recom_OK       F2Nb2_OK    
 Min.   :0.000   Min.   :0.000   Min.   :0.000   Min.   :0.000  
 1st Qu.:1.000   1st Qu.:1.000   1st Qu.:0.000   1st Qu.:1.000  
 Median :2.000   Median :2.000   Median :0.000   Median :2.000  
 Mean   :1.907   Mean   :1.904   Mean   :0.256   Mean   :1.664  
 3rd Qu.:3.000   3rd Qu.:3.000   3rd Qu.:1.000   3rd Qu.:2.000  
 Max.   :5.000   Max.   :4.000   Max.   :1.000   Max.   :5.000  
 NA's   :3212    NA's   :3212    NA's   :3212    NA's   :3210   
   F2Cat2_OK      F2Recom2_OK      F2Mediter1      F2Mediter2   
 Min.   :0.000   Min.   :0.000   Min.   :0.000   Min.   :0.000  
 1st Qu.:1.000   1st Qu.:0.000   1st Qu.:3.000   1st Qu.:3.000  
 Median :2.000   Median :0.000   Median :4.000   Median :5.000  
 Mean   :1.661   Mean   :0.195   Mean   :3.974   Mean   :4.614  
 3rd Qu.:2.000   3rd Qu.:0.000   3rd Qu.:5.000   3rd Qu.:6.000  
 Max.   :4.000   Max.   :1.000   Max.   :8.000   Max.   :9.000  
 NA's   :3210    NA's   :3210    NA's   :3419    NA's   :3923   
    F2AHEI1         F2AHEI2       F2vegetarian      F3datquest       
 Min.   : 4.00   Min.   : 4.00   Min.   :0.0000   Length:6746       
 1st Qu.:25.00   1st Qu.:25.00   1st Qu.:0.0000   Class :character  
 Median :32.00   Median :32.00   Median :0.0000   Mode  :character  
 Mean   :32.02   Mean   :32.22   Mean   :0.0059                     
 3rd Qu.:39.00   3rd Qu.:39.00   3rd Qu.:0.0000                     
 Max.   :67.50   Max.   :67.50   Max.   :1.0000                     
 NA's   :3200    NA's   :3197    NA's   :1865                       
```

```
    F3mrtsts          F3MME            F3CESD          F3depressed   
 Min.   :0.000   Min.   : 1.00   Min.   : 0.00   Min.   :0.000  
 1st Qu.:0.000   1st Qu.:29.00   1st Qu.: 4.00   1st Qu.:0.000  
 Median :0.000   Median :30.00   Median : 9.00   Median :0.000  
 Mean   :0.858   Mean   :29.02   Mean   :10.11   Mean   :0.112  
 3rd Qu.:2.000   3rd Qu.:30.00   3rd Qu.:14.00   3rd Qu.:0.000  
 Max.   :3.000   Max.   :30.00   Max.   :50.00   Max.   :1.000  
 NA's   :3668    NA's   :4549    NA's   :4257    NA's   :4257   
   F3Fasting         F3chol           F3hdlch          F3ldlch     
 Min.   :1.000   Min.   : 1.800   Min.   :0.500   Min.   :0.200  
 1st Qu.:1.000   1st Qu.: 4.500   1st Qu.:1.200   1st Qu.:2.400  
 Median :1.000   Median : 5.200   Median :1.500   Median :3.000  
 Mean   :1.037   Mean   : 5.197   Mean   :1.562   Mean   :3.035  
 3rd Qu.:1.000   3rd Qu.: 5.900   3rd Qu.:1.800   3rd Qu.:3.600  
 Max.   :9.000   Max.   :10.200   Max.   :3.700   Max.   :6.800  
 NA's   :3196    NA's   :3206     NA's   :3207    NA's   :3234   
     F3trig          F3gluc           F3insulin          F3hba1c      
 Min.   : 0.30   Min.   : 3.100   Min.   :  0.40   Min.   : 17.00  
 1st Qu.: 0.90   1st Qu.: 5.000   1st Qu.:  5.80   1st Qu.: 34.00  
 Median : 1.10   Median : 5.300   Median :  8.50   Median : 37.00  
 Mean   : 1.34   Mean   : 5.558   Mean   : 11.07   Mean   : 37.48  
 3rd Qu.: 1.60   3rd Qu.: 5.800   3rd Qu.: 12.90   3rd Qu.: 39.00  
 Max.   :14.60   Max.   :19.000   Max.   :416.00   Max.   :114.00  
 NA's   :3207    NA's   :3210     NA's   :3212     NA's   :3212    
    F3hba1s           F3crpu          F3freqFFQ1       F3freqFFQ2    
 Min.   : 3.700   Min.   : 0.000   Min.   :0.000   Min.   :0.000  
 1st Qu.: 5.300   1st Qu.: 0.700   1st Qu.:0.000   1st Qu.:0.000  
 Median : 5.500   Median : 1.250   Median :0.036   Median :0.000  
 Mean   : 5.574   Mean   : 2.328   Mean   :0.216   Mean   :0.052  
 3rd Qu.: 5.700   3rd Qu.: 2.700   3rd Qu.:0.214   3rd Qu.:0.000  
 Max.   :12.600   Max.   :21.000   Max.   :2.500   Max.   :2.500  
 NA's   :3212     NA's   :3208     NA's   :4104    NA's   :4119   
   F3freqFFQ3      F3freqFFQ4      F3freqFFQ5      F3freqFFQ6    
 Min.   :0.000   Min.   :0.000   Min.   :0.000   Min.   :0.000  
 1st Qu.:0.000   1st Qu.:0.000   1st Qu.:0.000   1st Qu.:0.000  
 Median :0.089   Median :0.000   Median :0.000   Median :0.089  
 Mean   :0.236   Mean   :0.037   Mean   :0.066   Mean   :0.112  
 3rd Qu.:0.214   3rd Qu.:0.000   3rd Qu.:0.089   3rd Qu.:0.214  
 Max.   :2.500   Max.   :2.500   Max.   :2.500   Max.   :2.500  
 NA's   :4087    NA's   :4118    NA's   :4121    NA's   :4109   
   F3freqFFQ7      F3freqFFQ8      F3freqFFQ9      F3freqFFQ10   
 Min.   :0.000   Min.   :0.000   Min.   :0.000   Min.   :0.000  
 1st Qu.:0.089   1st Qu.:0.000   1st Qu.:0.036   1st Qu.:0.036  
 Median :0.214   Median :0.000   Median :0.214   Median :0.214  
 Mean   :0.370   Mean   :0.024   Mean   :0.513   Mean   :0.496  
 3rd Qu.:0.500   3rd Qu.:0.036   3rd Qu.:1.000   3rd Qu.:1.000  
 Max.   :2.500   Max.   :2.500   Max.   :2.500   Max.   :2.500  
 NA's   :4071    NA's   :4089    NA's   :4098    NA's   :4108   
  F3freqFFQ11     F3freqFFQ12     F3freqFFQ13     F3freqFFQ14    
 Min.   :0.000   Min.   :0.000   Min.   :0.000   Min.   :0.000  
 1st Qu.:0.000   1st Qu.:0.000   1st Qu.:0.000   1st Qu.:0.036  
 Median :0.000   Median :0.000   Median :0.000   Median :0.089  
 Mean   :0.167   Mean   :0.034   Mean   :0.094   Mean   :0.123  
 3rd Qu.:0.214   3rd Qu.:0.000   3rd Qu.:0.036   3rd Qu.:0.214  
 Max.   :2.500   Max.   :1.000   Max.   :2.500   Max.   :2.500  
 NA's   :4115    NA's   :4122    NA's   :4115    NA's   :4116   
  F3freqFFQ15     F3freqFFQ16     F3freqFFQ17     F3freqFFQ18    
 Min.   :0.000   Min.   :0.000   Min.   :0.000   Min.   :0.000  
 1st Qu.:0.036   1st Qu.:0.000   1st Qu.:0.036   1st Qu.:0.000  
 Median :0.089   Median :0.036   Median :0.089   Median :0.089  
 Mean   :0.125   Mean   :0.055   Mean   :0.094   Mean   :0.084  
 3rd Qu.:0.214   3rd Qu.:0.089   3rd Qu.:0.089   3rd Qu.:0.089  
 Max.   :2.500   Max.   :1.000   Max.   :2.500   Max.   :1.000  
 NA's   :4113    NA's   :4125    NA's   :4117    NA's   :4122   
```

```
  F3freqFFQ19     F3freqFFQ20     F3freqFFQ21     F3freqFFQ22  
 Min.   :0.000   Min.   :0.000   Min.   :0.000   Min.   :0.000  
 1st Qu.:0.036   1st Qu.:0.000   1st Qu.:0.000   1st Qu.:0.000  
 Median :0.089   Median :0.000   Median :0.000   Median :0.036  
 Mean   :0.104   Mean   :0.029   Mean   :0.020   Mean   :0.039  
 3rd Qu.:0.089   3rd Qu.:0.036   3rd Qu.:0.036   3rd Qu.:0.036  
 Max.   :1.000   Max.   :2.500   Max.   :1.000   Max.   :1.000  
 NA's   :4118    NA's   :4126    NA's   :4129    NA's   :4132   
  F3freqFFQ23     F3freqFFQ24     F3freqFFQ25     F3freqFFQ26  
 Min.   :0.000   Min.   :0.000   Min.   :0.000   Min.   :0.000  
 1st Qu.:0.000   1st Qu.:0.000   1st Qu.:0.036   1st Qu.:0.000  
 Median :0.000   Median :0.000   Median :0.089   Median :0.036  
 Mean   :0.013   Mean   :0.005   Mean   :0.080   Mean   :0.049  
 3rd Qu.:0.000   3rd Qu.:0.000   3rd Qu.:0.089   3rd Qu.:0.089  
 Max.   :0.500   Max.   :0.500   Max.   :2.500   Max.   :2.500  
 NA's   :4123    NA's   :4131    NA's   :4106    NA's   :4118   
  F3freqFFQ27     F3freqFFQ28     F3freqFFQ29     F3freqFFQ30  
 Min.   :0.000   Min.   :0.000   Min.   :0.000   Min.   :0.000  
 1st Qu.:0.000   1st Qu.:0.000   1st Qu.:0.000   1st Qu.:0.036  
 Median :0.036   Median :0.089   Median :0.036   Median :0.089  
 Mean   :0.042   Mean   :0.086   Mean   :0.045   Mean   :0.136  
 3rd Qu.:0.089   3rd Qu.:0.089   3rd Qu.:0.089   3rd Qu.:0.214  
 Max.   :2.500   Max.   :2.500   Max.   :2.500   Max.   :2.500  
 NA's   :4116    NA's   :4115    NA's   :4122    NA's   :4113   
  F3freqFFQ31     F3freqFFQ32     F3freqFFQ33     F3freqFFQ34  
 Min.   :0.000   Min.   :0.000   Min.   :0.000   Min.   :0.000  
 1st Qu.:0.036   1st Qu.:0.089   1st Qu.:0.089   1st Qu.:0.214  
 Median :0.089   Median :0.214   Median :0.214   Median :0.500  
 Mean   :0.121   Mean   :0.318   Mean   :0.248   Mean   :0.516  
 3rd Qu.:0.214   3rd Qu.:0.500   3rd Qu.:0.214   3rd Qu.:0.500  
 Max.   :2.500   Max.   :2.500   Max.   :2.500   Max.   :2.500  
 NA's   :4114    NA's   :4108    NA's   :4116    NA's   :4102   
  F3freqFFQ35     F3freqFFQ36     F3freqFFQ37     F3freqFFQ38  
 Min.   :0.000   Min.   :0.000   Min.   :0.000   Min.   :0.000  
 1st Qu.:0.000   1st Qu.:0.000   1st Qu.:0.000   1st Qu.:0.000  
 Median :0.214   Median :0.036   Median :0.036   Median :0.036  
 Mean   :0.337   Mean   :0.082   Mean   :0.090   Mean   :0.094  
 3rd Qu.:0.500   3rd Qu.:0.089   3rd Qu.:0.089   3rd Qu.:0.089  
 Max.   :2.500   Max.   :2.500   Max.   :2.500   Max.   :2.500  
 NA's   :4127    NA's   :4129    NA's   :4125    NA's   :4131   
  F3freqFFQ39     F3freqFFQ40     F3freqFFQ41     F3freqFFQ42  
 Min.   :0.000   Min.   :0.000   Min.   :0.000   Min.   :0.000  
 1st Qu.:0.000   1st Qu.:0.089   1st Qu.:0.000   1st Qu.:0.089  
 Median :0.036   Median :0.214   Median :0.036   Median :0.214  
 Mean   :0.109   Mean   :0.198   Mean   :0.053   Mean   :0.202  
 3rd Qu.:0.089   3rd Qu.:0.214   3rd Qu.:0.089   3rd Qu.:0.214  
 Max.   :2.500   Max.   :2.500   Max.   :2.500   Max.   :2.500  
 NA's   :4127    NA's   :4104    NA's   :4121    NA's   :4102   
  F3freqFFQ43     F3freqFFQ44     F3freqFFQ45     F3freqFFQ46  
 Min.   :0.000   Min.   :0.000   Min.   :0.000   Min.   :0.000  
 1st Qu.:0.000   1st Qu.:0.089   1st Qu.:0.036   1st Qu.:0.000  
 Median :0.036   Median :0.214   Median :0.089   Median :0.000  
 Mean   :0.047   Mean   :0.182   Mean   :0.116   Mean   :0.044  
 3rd Qu.:0.089   3rd Qu.:0.214   3rd Qu.:0.214   3rd Qu.:0.089  
 Max.   :1.000   Max.   :2.500   Max.   :2.500   Max.   :1.000  
 NA's   :4135    NA's   :4099    NA's   :4121    NA's   :4136   
  F3freqFFQ47     F3freqFFQ48     F3freqFFQ49     F3freqFFQ50     F3freqFFQ51 
 Min.   :0.000   Min.   :0.000   Min.   :0.000   Min.   :0.000   Min.   :0.00  
 1st Qu.:0.000   1st Qu.:0.000   1st Qu.:0.089   1st Qu.:0.000   1st Qu.:0.00  
 Median :0.036   Median :0.000   Median :0.214   Median :0.000   Median :0.00  
 Mean   :0.056   Mean   :0.015   Mean   :0.206   Mean   :0.027   Mean   :0.09  
 3rd Qu.:0.089   3rd Qu.:0.000   3rd Qu.:0.214   3rd Qu.:0.000   3rd Qu.:0.00  
 Max.   :1.000   Max.   :2.500   Max.   :2.500   Max.   :2.500   Max.   :2.50  
 NA's   :4119    NA's   :4137    NA's   :4095    NA's   :4131    NA's   :4147  
```

```
  F3freqFFQ52     F3freqFFQ53     F3freqFFQ54     F3freqFFQ55   
 Min.   :0.000   Min.   :0.000   Min.   :0.000   Min.   :0.000  
 1st Qu.:0.036   1st Qu.:0.000   1st Qu.:0.000   1st Qu.:0.214  
 Median :0.214   Median :0.000   Median :0.000   Median :0.500  
 Mean   :0.404   Mean   :0.071   Mean   :0.060   Mean   :0.679  
 3rd Qu.:1.000   3rd Qu.:0.089   3rd Qu.:0.089   3rd Qu.:1.000  
 Max.   :2.500   Max.   :1.000   Max.   :2.500   Max.   :2.500  
 NA's   :4126    NA's   :4146    NA's   :4144    NA's   :4099   
  F3freqFFQ56     F3freqFFQ57     F3freqFFQ58     F3freqFFQ59   
 Min.   :0.000   Min.   :0.000   Min.   :0.000   Min.   :0.000  
 1st Qu.:0.000   1st Qu.:0.000   1st Qu.:0.000   1st Qu.:0.000  
 Median :0.214   Median :0.089   Median :0.036   Median :0.036  
 Mean   :0.413   Mean   :0.245   Mean   :0.172   Mean   :0.165  
 3rd Qu.:0.500   3rd Qu.:0.214   3rd Qu.:0.214   3rd Qu.:0.214  
 Max.   :2.500   Max.   :2.500   Max.   :2.500   Max.   :2.500  
 NA's   :4116    NA's   :4122    NA's   :4125    NA's   :4129   
  F3freqFFQ60     F3freqFFQ61     F3freqFFQ62     F3freqFFQ63   
 Min.   :0.000   Min.   :0.000   Min.   :0.000   Min.   :0.000  
 1st Qu.:0.000   1st Qu.:0.000   1st Qu.:0.000   1st Qu.:0.000  
 Median :0.000   Median :0.036   Median :0.036   Median :0.000  
 Mean   :0.027   Mean   :0.096   Mean   :0.076   Mean   :0.020  
 3rd Qu.:0.000   3rd Qu.:0.089   3rd Qu.:0.089   3rd Qu.:0.036  
 Max.   :2.500   Max.   :2.500   Max.   :2.500   Max.   :2.500  
 NA's   :4140    NA's   :4123    NA's   :4127    NA's   :4132   
  F3freqFFQ64     F3freqFFQ65     F3freqFFQ66     F3freqFFQ67   
 Min.   :0.000   Min.   :0.000   Min.   :0.000   Min.   :0.000  
 1st Qu.:0.000   1st Qu.:0.000   1st Qu.:0.089   1st Qu.:0.089  
 Median :0.036   Median :0.089   Median :0.214   Median :0.214  
 Mean   :0.062   Mean   :0.174   Mean   :0.411   Mean   :0.465  
 3rd Qu.:0.089   3rd Qu.:0.214   3rd Qu.:0.500   3rd Qu.:1.000  
 Max.   :2.500   Max.   :2.500   Max.   :2.500   Max.   :2.500  
 NA's   :4132    NA's   :4124    NA's   :4114    NA's   :4111   
  F3freqFFQ68     F3freqFFQ69     F3freqFFQ70     F3freqFFQ71   
 Min.   :0.000   Min.   :0.000   Min.   :0.000   Min.   :0.000  
 1st Qu.:0.000   1st Qu.:0.000   1st Qu.:0.000   1st Qu.:0.000  
 Median :0.036   Median :0.000   Median :0.036   Median :0.036  
 Mean   :0.088   Mean   :0.177   Mean   :0.443   Mean   :0.182  
 3rd Qu.:0.089   3rd Qu.:0.000   3rd Qu.:1.000   3rd Qu.:0.214  
 Max.   :2.500   Max.   :2.500   Max.   :2.500   Max.   :2.500  
 NA's   :4126    NA's   :4130    NA's   :4133    NA's   :4150   
  F3freqFFQ72     F3freqFFQ73     F3freqFFQ74     F3freqFFQ75   
 Min.   :0.000   Min.   :0.000   Min.   :0.000   Min.   :0.000  
 1st Qu.:0.000   1st Qu.:0.214   1st Qu.:0.000   1st Qu.:0.000  
 Median :0.000   Median :0.500   Median :0.000   Median :0.000  
 Mean   :0.048   Mean   :0.692   Mean   :0.088   Mean   :0.198  
 3rd Qu.:0.000   3rd Qu.:1.000   3rd Qu.:0.036   3rd Qu.:0.214  
 Max.   :2.500   Max.   :2.500   Max.   :2.500   Max.   :2.500  
 NA's   :4155    NA's   :4107    NA's   :4153    NA's   :4139   
  F3freqFFQ76     F3freqFFQ77     F3freqFFQ78     F3freqFFQ79   
 Min.   :0.000   Min.   :0.000   Min.   :0.000   Min.   :0.000  
 1st Qu.:0.000   1st Qu.:0.000   1st Qu.:0.000   1st Qu.:0.000  
 Median :0.000   Median :0.000   Median :0.000   Median :0.000  
 Mean   :0.093   Mean   :0.035   Mean   :0.078   Mean   :0.024  
 3rd Qu.:0.000   3rd Qu.:0.000   3rd Qu.:0.000   3rd Qu.:0.000  
 Max.   :2.500   Max.   :2.500   Max.   :2.500   Max.   :2.500  
 NA's   :4148    NA's   :4153    NA's   :4143    NA's   :4147   
  F3freqFFQ80     F3freqFFQ81     F3freqFFQ82     F3freqFFQ83   
 Min.   :0.000   Min.   :0.000   Min.   :0.000   Min.   :0.000  
 1st Qu.:0.000   1st Qu.:1.000   1st Qu.:0.000   1st Qu.:0.000  
 Median :0.000   Median :2.500   Median :0.000   Median :0.000  
 Mean   :0.015   Mean   :1.649   Mean   :0.191   Mean   :0.373  
 3rd Qu.:0.000   3rd Qu.:2.500   3rd Qu.:0.000   3rd Qu.:0.214  
 Max.   :2.500   Max.   :2.500   Max.   :2.500   Max.   :2.500  
 NA's   :4147    NA's   :4102    NA's   :4138    NA's   :4132   
```

```
  F3freqFFQ84     F3freqFFQ85     F3freqFFQ86     F3freqFFQ87   
 Min.   :0.000   Min.   :0.000   Min.   :0.000   Min.   :0.000  
 1st Qu.:0.000   1st Qu.:0.000   1st Qu.:0.000   1st Qu.:0.000  
 Median :0.000   Median :0.000   Median :0.000   Median :0.000  
 Mean   :0.262   Mean   :0.037   Mean   :0.166   Mean   :0.497  
 3rd Qu.:0.000   3rd Qu.:0.000   3rd Qu.:0.089   3rd Qu.:0.500  
 Max.   :2.500   Max.   :2.500   Max.   :2.500   Max.   :2.500  
 NA's   :4135    NA's   :4139    NA's   :4137    NA's   :4134   
  F3freqFFQ88     F3freqFFQ89     F3freqFFQ90     F3freqFFQ91   
 Min.   :0.000   Min.   :0.000   Min.   :0.000   Min.   :0.000  
 1st Qu.:0.000   1st Qu.:1.000   1st Qu.:0.000   1st Qu.:0.000  
 Median :0.000   Median :2.500   Median :0.000   Median :0.000  
 Mean   :0.184   Mean   :1.854   Mean   :0.166   Mean   :0.191  
 3rd Qu.:0.000   3rd Qu.:2.500   3rd Qu.:0.089   3rd Qu.:0.214  
 Max.   :2.500   Max.   :2.500   Max.   :2.500   Max.   :2.500  
 NA's   :4145    NA's   :4106    NA's   :4131    NA's   :4135   
  F3freqFFQ92     F3freqFFQ93     F3freqFFQ94     F3freqFFQ95   
 Min.   :0.000   Min.   :0.000   Min.   :0.000   Min.   :0.000  
 1st Qu.:0.000   1st Qu.:0.089   1st Qu.:0.000   1st Qu.:0.036  
 Median :0.000   Median :0.500   Median :0.000   Median :0.214  
 Mean   :0.206   Mean   :0.948   Mean   :0.164   Mean   :0.378  
 3rd Qu.:0.214   3rd Qu.:2.500   3rd Qu.:0.089   3rd Qu.:0.500  
 Max.   :2.500   Max.   :2.500   Max.   :2.500   Max.   :2.500  
 NA's   :4137    NA's   :4107    NA's   :4127    NA's   :4124   
  F3freqFFQ96     F3freqFFQ97     F3FFQ1amount      F3FFQ2amount    
 Min.   :0.000   Min.   :0.000   Min.   :  0.000   Min.   :  0.000  
 1st Qu.:0.000   1st Qu.:0.000   1st Qu.:  0.000   1st Qu.:  0.000  
 Median :0.000   Median :0.000   Median :  6.429   Median :  0.000  
 Mean   :0.035   Mean   :0.038   Mean   : 35.996   Mean   :  9.152  
 3rd Qu.:0.000   3rd Qu.:0.000   3rd Qu.: 38.571   3rd Qu.:  0.000  
 Max.   :2.500   Max.   :2.500   Max.   :675.000   Max.   :675.000  
 NA's   :4137    NA's   :4137    NA's   :4104      NA's   :4119     
  F3FFQ3amount     F3FFQ4amount      F3FFQ5amount      F3FFQ6amount    
 Min.   :  0.00   Min.   :  0.000   Min.   :  0.000   Min.   :  0.000  
 1st Qu.:  0.00   1st Qu.:  0.000   1st Qu.:  0.000   1st Qu.:  0.000  
 Median : 16.07   Median :  0.000   Median :  0.000   Median :  3.571  
 Mean   : 41.91   Mean   :  4.193   Mean   :  6.395   Mean   :  6.267  
 3rd Qu.: 38.57   3rd Qu.:  0.000   3rd Qu.:  4.464   3rd Qu.:  8.036  
 Max.   :675.00   Max.   :312.500   Max.   :375.000   Max.   :150.000  
 NA's   :4087     NA's   :4118      NA's   :4121      NA's   :4109     
  F3FFQ7amount      F3FFQ8amount       F3FFQ9amount      F3FFQ10amount    
 Min.   :  0.000   Min.   :   0.000   Min.   :  0.000   Min.   :  0.000  
 1st Qu.:  8.036   1st Qu.:   0.000   1st Qu.:  1.786   1st Qu.:  1.786  
 Median : 19.286   Median :   0.000   Median : 10.714   Median : 12.857  
 Mean   : 30.824   Mean   :   8.839   Mean   : 32.420   Mean   : 33.561  
 3rd Qu.: 45.000   3rd Qu.:  12.500   3rd Qu.: 50.000   3rd Qu.: 50.000  
 Max.   :300.000   Max.   :1312.500   Max.   :187.500   Max.   :225.000  
 NA's   :4071      NA's   :4089       NA's   :4098      NA's   :4108     
 F3FFQ11amount     F3FFQ12amount    F3FFQ13amount    F3FFQ14amount    
 Min.   :  0.000   Min.   : 0.000   Min.   : 0.000   Min.   :  0.000  
 1st Qu.:  0.000   1st Qu.: 0.000   1st Qu.: 0.000   1st Qu.:  2.143  
 Median :  0.000   Median : 0.000   Median : 0.000   Median : 11.607  
 Mean   :  9.138   Mean   : 1.093   Mean   : 1.749   Mean   : 17.559  
 3rd Qu.: 10.714   3rd Qu.: 0.000   3rd Qu.: 0.804   3rd Qu.: 27.857  
 Max.   :187.500   Max.   :45.000   Max.   :67.500   Max.   :562.500  
 NA's   :4115      NA's   :4122     NA's   :4115     NA's   :4116     
 F3FFQ15amount     F3FFQ16amount     F3FFQ17amount     F3FFQ18amount    
 Min.   :  0.000   Min.   :  0.000   Min.   :  0.000   Min.   :  0.000  
 1st Qu.:  3.571   1st Qu.:  0.000   1st Qu.:  3.571   1st Qu.:  0.000  
 Median :  8.929   Median :  1.786   Median : 11.161   Median :  5.893  
 Mean   : 13.770   Mean   :  6.364   Mean   : 15.082   Mean   : 10.260  
 3rd Qu.: 21.429   3rd Qu.:  8.929   3rd Qu.: 20.089   3rd Qu.: 14.732  
 Max.   :250.000   Max.   :150.000   Max.   :562.500   Max.   :165.000  
 NA's   :4113      NA's   :4125      NA's   :4117      NA's   :4122     
```

```
 F3FFQ19amount    F3FFQ20amount     F3FFQ21amount     F3FFQ22amount    
 Min.   : 0.000   Min.   :  0.000   Min.   :  0.000   Min.   :  0.000  
 1st Qu.: 1.786   1st Qu.:  0.000   1st Qu.:  0.000   1st Qu.:  0.000  
 Median : 4.464   Median :  0.000   Median :  0.000   Median :  1.429  
 Mean   : 5.896   Mean   :  2.574   Mean   :  1.868   Mean   :  3.035  
 3rd Qu.: 6.696   3rd Qu.:  2.857   3rd Qu.:  1.964   3rd Qu.:  4.107  
 Max.   :75.000   Max.   :300.000   Max.   :110.000   Max.   :120.000  
 NA's   :4118     NA's   :4126      NA's   :4129      NA's   :4132     
 F3FFQ23amount   F3FFQ24amount    F3FFQ25amount     F3FFQ26amount    
 Min.   : 0.00   Min.   : 0.000   Min.   :  0.000   Min.   :  0.000  
 1st Qu.: 0.00   1st Qu.: 0.000   1st Qu.:  2.679   1st Qu.:  0.000  
 Median : 0.00   Median : 0.000   Median :  8.036   Median :  5.357  
 Mean   : 1.26   Mean   : 0.275   Mean   : 12.560   Mean   :  7.516  
 3rd Qu.: 0.00   3rd Qu.: 0.000   3rd Qu.: 13.393   3rd Qu.: 13.393  
 Max.   :50.00   Max.   :25.000   Max.   :375.000   Max.   :375.000  
 NA's   :4123    NA's   :4131     NA's   :4106      NA's   :4118     
 F3FFQ27amount     F3FFQ28amount     F3FFQ29amount     F3FFQ30amount    
 Min.   :  0.000   Min.   :  0.000   Min.   :  0.000   Min.   :  0.000  
 1st Qu.:  0.000   1st Qu.:  0.000   1st Qu.:  0.000   1st Qu.:  5.357  
 Median :  1.429   Median :  8.036   Median :  1.786   Median : 13.393  
 Mean   :  3.612   Mean   : 13.213   Mean   :  2.435   Mean   : 22.575  
 3rd Qu.:  4.286   3rd Qu.: 13.393   3rd Qu.:  4.464   3rd Qu.: 32.143  
 Max.   :300.000   Max.   :375.000   Max.   :125.000   Max.   :562.500  
 NA's   :4116      NA's   :4115      NA's   :4122      NA's   :4113     
 F3FFQ31amount     F3FFQ32amount     F3FFQ33amount     F3FFQ34amount   
 Min.   :  0.000   Min.   :  0.000   Min.   :  0.000   Min.   :  0.00  
 1st Qu.:  7.143   1st Qu.:  8.036   1st Qu.:  6.696   1st Qu.: 10.71  
 Median : 17.857   Median : 19.286   Median : 16.071   Median : 25.00  
 Mean   : 26.366   Mean   : 30.922   Mean   : 20.374   Mean   : 30.91  
 3rd Qu.: 42.857   3rd Qu.: 45.000   3rd Qu.: 24.643   3rd Qu.: 37.50  
 Max.   :500.000   Max.   :337.500   Max.   :287.500   Max.   :187.50  
 NA's   :4114      NA's   :4108      NA's   :4116      NA's   :4102    
 F3FFQ35amount    F3FFQ36amount     F3FFQ37amount     F3FFQ38amount    
 Min.   : 0.000   Min.   :  0.000   Min.   :  0.000   Min.   :   0.00  
 1st Qu.: 0.000   1st Qu.:  0.000   1st Qu.:  0.000   1st Qu.:   0.00  
 Median : 3.214   Median :  2.143   Median :  6.429   Median :  10.71  
 Mean   : 5.624   Mean   :  5.154   Mean   : 17.859   Mean   :  29.86  
 3rd Qu.: 7.500   3rd Qu.:  5.357   3rd Qu.: 16.071   3rd Qu.:  26.79  
 Max.   :62.500   Max.   :150.000   Max.   :675.000   Max.   :1125.00  
 NA's   :4127     NA's   :4129      NA's   :4125      NA's   :4131     
 F3FFQ39amount     F3FFQ40amount     F3FFQ41amount     F3FFQ42amount   
 Min.   :  0.000   Min.   :  0.000   Min.   :  0.000   Min.   :  0.00  
 1st Qu.:  0.000   1st Qu.:  8.929   1st Qu.:  0.000   1st Qu.: 17.86  
 Median :  8.929   Median : 21.429   Median :  4.286   Median : 42.86  
 Mean   : 29.197   Mean   : 27.464   Mean   :  6.989   Mean   : 55.21  
 3rd Qu.: 33.482   3rd Qu.: 32.143   3rd Qu.: 10.714   3rd Qu.: 64.29  
 Max.   :625.000   Max.   :375.000   Max.   :300.000   Max.   :750.00  
 NA's   :4127      NA's   :4104      NA's   :4121      NA's   :4102    
 F3FFQ43amount     F3FFQ44amount     F3FFQ45amount     F3FFQ46amount   
 Min.   :  0.000   Min.   :  0.000   Min.   :  0.000   Min.   : 0.000  
 1st Qu.:  0.000   1st Qu.:  5.357   1st Qu.:  2.857   1st Qu.: 0.000  
 Median :  5.000   Median :  8.036   Median :  7.143   Median : 0.000  
 Mean   :  7.975   Mean   : 11.359   Mean   : 10.260   Mean   : 1.479  
 3rd Qu.: 12.500   3rd Qu.: 12.857   3rd Qu.: 17.143   3rd Qu.: 2.679  
 Max.   :270.000   Max.   :150.000   Max.   :300.000   Max.   :30.000  
 NA's   :4135      NA's   :4099      NA's   :4121      NA's   :4136    
 F3FFQ47amount     F3FFQ48amount     F3FFQ49amount     F3FFQ50amount    
 Min.   :  0.000   Min.   :  0.000   Min.   :  0.000   Min.   :  0.000  
 1st Qu.:  0.000   1st Qu.:  0.000   1st Qu.:  8.929   1st Qu.:  0.000  
 Median :  8.929   Median :  0.000   Median : 13.393   Median :  0.000  
 Mean   : 15.916   Mean   :  2.509   Mean   : 19.066   Mean   :  2.884  
 3rd Qu.: 22.321   3rd Qu.:  0.000   3rd Qu.: 21.429   3rd Qu.:  0.000  
 Max.   :250.000   Max.   :187.500   Max.   :250.000   Max.   :375.000  
 NA's   :4119      NA's   :4137      NA's   :4095      NA's   :4131     
```

```
 F3FFQ51amount    F3FFQ52amount    F3FFQ53amount    F3FFQ54amount   
 Min.   : 0.000   Min.   : 0.000   Min.   : 0.000   Min.   : 0.000  
 1st Qu.: 0.000   1st Qu.: 0.143   1st Qu.: 0.000   1st Qu.: 0.000  
 Median : 0.000   Median : 1.500   Median : 0.000   Median : 0.000  
 Mean   : 0.811   Mean   : 3.574   Mean   : 1.321   Mean   : 0.545  
 3rd Qu.: 0.000   3rd Qu.: 7.000   3rd Qu.: 1.786   3rd Qu.: 0.625  
 Max.   :37.500   Max.   :37.500   Max.   :30.000   Max.   :17.500  
 NA's   :4147     NA's   :4126     NA's   :4146     NA's   :4144    
 F3FFQ55amount    F3FFQ56amount    F3FFQ57amount     F3FFQ58amount    
 Min.   :  0.00   Min.   :  0.00   Min.   :  0.000   Min.   :  0.000  
 1st Qu.: 27.86   1st Qu.:  0.00   1st Qu.:  0.000   1st Qu.:  0.000  
 Median : 65.00   Median : 32.14   Median :  8.929   Median :  5.357  
 Mean   : 99.41   Mean   : 68.46   Mean   : 27.269   Mean   : 21.708  
 3rd Qu.:130.00   3rd Qu.: 75.00   3rd Qu.: 32.143   3rd Qu.: 23.571  
 Max.   :487.50   Max.   :562.50   Max.   :375.000   Max.   :562.500  
 NA's   :4099     NA's   :4116     NA's   :4122      NA's   :4125     
 F3FFQ59amount     F3FFQ60amount     F3FFQ61amount     F3FFQ62amount    
 Min.   :  0.000   Min.   :  0.000   Min.   :  0.000   Min.   :  0.000  
 1st Qu.:  0.000   1st Qu.:  0.000   1st Qu.:  0.000   1st Qu.:  0.000  
 Median :  3.214   Median :  0.000   Median :  1.429   Median :  6.429  
 Mean   : 15.321   Mean   :  4.892   Mean   :  4.090   Mean   : 11.254  
 3rd Qu.: 19.286   3rd Qu.:  0.000   3rd Qu.:  3.571   3rd Qu.: 13.393  
 Max.   :337.500   Max.   :750.000   Max.   :150.000   Max.   :450.000  
 NA's   :4129      NA's   :4140      NA's   :4123      NA's   :4127     
 F3FFQ63amount     F3FFQ64amount     F3FFQ65amount     F3FFQ66amount   
 Min.   :  0.000   Min.   :  0.000   Min.   :  0.000   Min.   : 0.000  
 1st Qu.:  0.000   1st Qu.:  0.000   1st Qu.:  0.000   1st Qu.: 1.339  
 Median :  0.000   Median :  1.786   Median :  1.339   Median : 4.286  
 Mean   :  1.972   Mean   :  6.131   Mean   :  3.830   Mean   : 8.233  
 3rd Qu.:  3.571   3rd Qu.:  8.929   3rd Qu.:  5.018   3rd Qu.:10.000  
 Max.   :250.000   Max.   :250.000   Max.   :100.000   Max.   :75.000  
 NA's   :4132      NA's   :4132      NA's   :4124      NA's   :4114    
 F3FFQ67amount    F3FFQ68amount     F3FFQ70amount    F3FFQ71amount   
 Min.   : 0.000   Min.   :  0.000   Min.   : 0.000   Min.   : 0.000  
 1st Qu.: 1.786   1st Qu.:  0.000   1st Qu.: 0.000   1st Qu.: 0.000  
 Median : 4.286   Median :  4.286   Median : 0.286   Median : 0.250  
 Mean   : 9.648   Mean   : 11.089   Mean   : 3.581   Mean   : 1.633  
 3rd Qu.:20.000   3rd Qu.: 10.714   3rd Qu.: 4.000   3rd Qu.: 2.143  
 Max.   :75.000   Max.   :450.000   Max.   :30.000   Max.   :37.500  
 NA's   :4111     NA's   :4126      NA's   :4133     NA's   :4150    
 F3FFQ72amount    F3FFQ73amount    F3FFQ74amount    F3FFQ75amount   
 Min.   : 0.000   Min.   : 0.000   Min.   : 0.000   Min.   : 0.000  
 1st Qu.: 0.000   1st Qu.: 2.143   1st Qu.: 0.000   1st Qu.: 0.000  
 Median : 0.000   Median : 5.000   Median : 0.000   Median : 0.000  
 Mean   : 0.405   Mean   : 6.284   Mean   : 0.737   Mean   : 1.698  
 3rd Qu.: 0.000   3rd Qu.:10.000   3rd Qu.: 0.250   3rd Qu.: 2.143  
 Max.   :25.000   Max.   :37.500   Max.   :25.000   Max.   :37.500  
 NA's   :4155     NA's   :4107     NA's   :4153     NA's   :4139    
 F3FFQ81amount   F3FFQ82amount     F3FFQ83amount     F3FFQ84amount   
 Min.   :  0.0   Min.   :  0.000   Min.   :  0.000   Min.   : 0.000  
 1st Qu.: 80.0   1st Qu.:  0.000   1st Qu.:  0.000   1st Qu.: 0.000  
 Median :200.0   Median :  0.000   Median :  0.000   Median : 0.000  
 Mean   :150.2   Mean   :  5.959   Mean   : 11.412   Mean   : 3.329  
 3rd Qu.:200.0   3rd Qu.:  0.000   3rd Qu.:  6.429   3rd Qu.: 0.000  
 Max.   :300.0   Max.   :112.500   Max.   :112.500   Max.   :45.000  
 NA's   :4102    NA's   :4138      NA's   :4132      NA's   :4135    
 F3FFQ85amount     F3FFQ86amount     F3FFQ87amount    F3FFQ88amount   
 Min.   :  0.000   Min.   :  0.00   Min.   :  0.0   Min.   :  0.00  
 1st Qu.:  0.000   1st Qu.:  0.00   1st Qu.:  0.0   1st Qu.:  0.00  
 Median :  0.000   Median :  0.00   Median :  0.0   Median :  0.00  
 Mean   :  5.705   Mean   : 26.57   Mean   :129.7   Mean   : 47.31  
 3rd Qu.:  0.000   3rd Qu.: 13.39   3rd Qu.:100.0   3rd Qu.:  0.00  
 Max.   :562.500   Max.   :562.50   Max.   :750.0   Max.   :750.00  
 NA's   :4139      NA's   :4137     NA's   :4134    NA's   :4145    
```

```
 F3FFQ89amount   F3FFQ90amount    F3FFQ91amount    F3FFQ92amount   
 Min.   :  0.0   Min.   :  0.00   Min.   :  0.00   Min.   :  0.00  
 1st Qu.:200.0   1st Qu.:  0.00   1st Qu.:  0.00   1st Qu.:  0.00  
 Median :500.0   Median :  0.00   Median :  0.00   Median :  0.00  
 Mean   :484.5   Mean   : 38.87   Mean   : 39.00   Mean   : 42.97  
 3rd Qu.:750.0   3rd Qu.: 17.86   3rd Qu.: 42.86   3rd Qu.: 42.86  
 Max.   :750.0   Max.   :750.00   Max.   :750.00   Max.   :750.00  
 NA's   :4106    NA's   :4131     NA's   :4135     NA's   :4137    
 F3FFQ93amount    F3FFQ94amount     F3FFQ95amount     F3FFQ96amount    
 Min.   :  0.00   Min.   :   0.00   Min.   :  0.000   Min.   :  0.000  
 1st Qu.: 17.86   1st Qu.:   0.00   1st Qu.:  5.357   1st Qu.:  0.000  
 Median :150.00   Median :   0.00   Median : 32.143   Median :  0.000  
 Mean   :233.41   Mean   :  55.72   Mean   : 66.749   Mean   :  1.871  
 3rd Qu.:500.00   3rd Qu.:  26.79   3rd Qu.: 75.000   3rd Qu.:  0.000  
 Max.   :750.00   Max.   :1125.00   Max.   :562.500   Max.   :187.500  
 NA's   :4107     NA's   :4127      NA's   :4124      NA's   :4137     
 F3FFQ97amount       F3numitems      F3sumprot1       F3sumpveg1    
 Min.   :  0.000   Min.   : 1.00   Min.   :   0.0   Min.   :  0.00  
 1st Qu.:  0.000   1st Qu.:43.00   1st Qu.: 199.7   1st Qu.: 55.61  
 Median :  0.000   Median :50.00   Median : 260.6   Median : 75.86  
 Mean   :  3.653   Mean   :49.67   Mean   : 277.2   Mean   : 82.38  
 3rd Qu.:  0.000   3rd Qu.:58.00   3rd Qu.: 336.9   3rd Qu.:101.31  
 Max.   :375.000   Max.   :88.00   Max.   :2066.7   Max.   :396.82  
 NA's   :4137      NA's   :4035    NA's   :4035     NA's   :4035    
   F3sumpani1       F3sumgluc1       F3sumgsuc1       F3sumgpol1    
 Min.   :   0.0   Min.   :   0.0   Min.   :   0.0   Min.   :   0.0  
 1st Qu.: 131.0   1st Qu.: 539.1   1st Qu.: 233.7   1st Qu.: 256.3  
 Median : 180.0   Median : 733.0   Median : 335.0   Median : 370.0  
 Mean   : 194.8   Mean   : 788.2   Mean   : 375.0   Mean   : 410.5  
 3rd Qu.: 239.4   3rd Qu.: 973.0   3rd Qu.: 473.1   3rd Qu.: 516.1  
 Max.   :1686.4   Max.   :4292.5   Max.   :2776.6   Max.   :2187.8  
 NA's   :4035     NA's   :4035     NA's   :4035     NA's   :4035    
   F3sumlipi1       F3sumlsat1       F3sumlmon1       F3sumlpol1    
 Min.   :   0.0   Min.   :   0.0   Min.   :   0.0   Min.   :  0.00  
 1st Qu.: 480.6   1st Qu.: 169.3   1st Qu.: 191.0   1st Qu.: 61.93  
 Median : 626.2   Median : 231.7   Median : 253.5   Median : 83.33  
 Mean   : 669.3   Mean   : 250.7   Mean   : 273.0   Mean   : 90.66  
 3rd Qu.: 816.3   3rd Qu.: 307.2   3rd Qu.: 334.8   3rd Qu.:109.47  
 Max.   :5254.7   Max.   :2580.6   Max.   :1987.5   Max.   :579.29  
 NA's   :4035     NA's   :4035     NA's   :4035     NA's   :4035    
   F3sumalco          F3sumtot1       F3sumcalc1       F3sumfer1     
 Min.   :   0.000   Min.   :   0   Min.   :   0.0   Min.   : 0.000  
 1st Qu.:   5.125   1st Qu.:1171   1st Qu.: 572.5   1st Qu.: 6.800  
 Median :  29.500   Median :1502   Median : 819.5   Median : 8.700  
 Mean   :  66.753   Mean   :1585   Mean   : 917.5   Mean   : 9.242  
 3rd Qu.:  92.400   3rd Qu.:1898   3rd Qu.:1154.9   3rd Qu.:11.100  
 Max.   :1722.000   Max.   :9745   Max.   :4528.1   Max.   :68.900  
 NA's   :4035       NA's   :4035   NA's   :4035     NA's   :4035    
   F3sumret1        F3sumcaro1      F3sumvitd1       F3sumfibr1   
 Min.   :   0.0   Min.   :    0   Min.   : 0.000   Min.   : 0.00  
 1st Qu.: 200.3   1st Qu.: 1959   1st Qu.: 1.400   1st Qu.: 8.65  
 Median : 311.5   Median : 3075   Median : 2.300   Median :12.40  
 Mean   : 459.1   Mean   : 3829   Mean   : 2.715   Mean   :14.06  
 3rd Qu.: 532.8   3rd Qu.: 4940   3rd Qu.: 3.300   3rd Qu.:17.40  
 Max.   :6964.9   Max.   :38581   Max.   :45.500   Max.   :77.80  
 NA's   :4035     NA's   :4035    NA's   :4035     NA's   :4035   
   F3sumchol1       F3sumprot3       F3sumpveg3       F3sumpani3    
 Min.   :   0.0   Min.   :   0.0   Min.   :  0.00   Min.   :   0.0  
 1st Qu.: 200.8   1st Qu.: 177.7   1st Qu.: 47.00   1st Qu.: 118.8  
 Median : 269.8   Median : 231.3   Median : 64.20   Median : 162.4  
 Mean   : 298.4   Mean   : 246.8   Mean   : 70.96   Mean   : 175.8  
 3rd Qu.: 365.0   3rd Qu.: 298.8   3rd Qu.: 87.50   3rd Qu.: 215.4  
 Max.   :2517.4   Max.   :1863.7   Max.   :367.40   Max.   :1511.5  
 NA's   :4035     NA's   :4035     NA's   :4035     NA's   :4035    
```

```
  F3sumgluc3       F3sumgsuc3       F3sumgpol3       F3sumlipi3    
 Min.   :   0.0   Min.   :   0.0   Min.   :   0.0   Min.   :   0.0  
 1st Qu.: 465.4   1st Qu.: 212.8   1st Qu.: 209.8   1st Qu.: 411.6  
 Median : 643.7   Median : 310.3   Median : 301.1   Median : 535.1  
 Mean   : 695.4   Mean   : 350.7   Mean   : 341.9   Mean   : 572.2  
 3rd Qu.: 862.8   3rd Qu.: 443.5   3rd Qu.: 426.6   3rd Qu.: 698.1  
 Max.   :3893.2   Max.   :2507.4   Max.   :2090.2   Max.   :4843.2  
 NA's   :4035     NA's   :4035     NA's   :4035     NA's   :4035    
  F3sumlsat3       F3sumlmon3       F3sumlpol3       F3sumtot3    
 Min.   :   0.0   Min.   :   0.0   Min.   :  0.00   Min.   :   0  
 1st Qu.: 143.6   1st Qu.: 163.3   1st Qu.: 52.45   1st Qu.:1106  
 Median : 195.0   Median : 218.9   Median : 70.40   Median :1436  
 Mean   : 210.1   Mean   : 236.2   Mean   : 75.95   Mean   :1514  
 3rd Qu.: 256.6   3rd Qu.: 290.0   3rd Qu.: 91.70   3rd Qu.:1801  
 Max.   :2351.8   Max.   :1848.4   Max.   :556.40   Max.   :9450  
 NA's   :4035     NA's   :4035     NA's   :4035     NA's   :4035  
  F3sumcalc3       F3sumfer3        F3sumret3        F3sumcaro3    
 Min.   :   0.0   Min.   : 0.000   Min.   :   0.0   Min.   :    0  
 1st Qu.: 564.0   1st Qu.: 6.400   1st Qu.: 200.3   1st Qu.: 1959  
 Median : 811.0   Median : 8.100   Median : 311.5   Median : 3075  
 Mean   : 909.9   Mean   : 8.645   Mean   : 459.1   Mean   : 3829  
 3rd Qu.:1146.8   3rd Qu.:10.300   3rd Qu.: 532.8   3rd Qu.: 4940  
 Max.   :4517.6   Max.   :68.600   Max.   :6964.9   Max.   :38581  
 NA's   :4035     NA's   :4035     NA's   :4035     NA's   :4035   
  F3sumvitd3       F3sumfibr3      F3sumchol3       F3sumeau1     
 Min.   : 0.000   Min.   : 0.00   Min.   :   0.0   Min.   : 0.000  
 1st Qu.: 1.400   1st Qu.: 8.65   1st Qu.: 200.8   1st Qu.: 6.000  
 Median : 2.300   Median :12.40   Median : 269.8   Median :10.500  
 Mean   : 2.715   Mean   :14.06   Mean   : 298.4   Mean   : 8.672  
 3rd Qu.: 3.300   3rd Qu.:17.40   3rd Qu.: 365.0   3rd Qu.:10.600  
 Max.   :45.500   Max.   :77.80   Max.   :2517.4   Max.   :31.500  
 NA's   :4035     NA's   :4035    NA's   :4035     NA's   :4035    
 F3pct_prot1     F3pct_pveg1      F3pct_pani1     F3pct_gluc1   
 Min.   : 2.20   Min.   : 0.000   Min.   : 0.00   Min.   : 0.00  
 1st Qu.:13.70   1st Qu.: 3.700   1st Qu.: 8.80   1st Qu.:38.10  
 Median :15.50   Median : 4.500   Median :11.00   Median :43.80  
 Mean   :15.88   Mean   : 4.553   Mean   :11.32   Mean   :43.86  
 3rd Qu.:17.60   3rd Qu.: 5.200   3rd Qu.:13.40   3rd Qu.:49.80  
 Max.   :72.70   Max.   :72.700   Max.   :42.90   Max.   :79.70  
 NA's   :4038    NA's   :4038     NA's   :4038    NA's   :4038   
 F3pct_gsuc1     F3pct_gpol1     F3pct_lipi1     F3pct_lsat1   
 Min.   : 0.00   Min.   : 0.00   Min.   : 0.90   Min.   : 0.00  
 1st Qu.:16.40   1st Qu.:16.40   1st Qu.:31.60   1st Qu.:10.90  
 Median :21.70   Median :20.50   Median :36.30   Median :13.10  
 Mean   :22.45   Mean   :21.26   Mean   :36.25   Mean   :13.25  
 3rd Qu.:27.30   3rd Qu.:25.60   3rd Qu.:40.90   3rd Qu.:15.30  
 Max.   :71.00   Max.   :77.00   Max.   :69.20   Max.   :41.30  
 NA's   :4038    NA's   :4038    NA's   :4038    NA's   :4038   
 F3pct_lmon1     F3pct_lpol1      F3pct_fibr1     F3pct_chol1    
 Min.   : 0.00   Min.   : 0.000   Min.   :0.000   Min.   :  0.00  
 1st Qu.:12.30   1st Qu.: 3.900   1st Qu.:0.600   1st Qu.: 14.60  
 Median :14.60   Median : 4.700   Median :0.800   Median : 18.00  
 Mean   :14.98   Mean   : 4.844   Mean   :0.883   Mean   : 19.24  
 3rd Qu.:17.30   3rd Qu.: 5.600   3rd Qu.:1.100   3rd Qu.: 22.20  
 Max.   :39.10   Max.   :22.200   Max.   :2.700   Max.   :123.20  
 NA's   :4038    NA's   :4038     NA's   :4038    NA's   :4038    
 F3pct_alco1      F3pct_prot3      F3pct_pveg3       F3pct_pani3   
 Min.   : 0.000   Min.   :  4.20   Min.   :  0.000   Min.   : 0.00  
 1st Qu.: 0.300   1st Qu.: 14.30   1st Qu.:  4.000   1st Qu.: 9.20  
 Median : 1.900   Median : 16.20   Median :  4.700   Median :11.50  
 Mean   : 4.015   Mean   : 16.73   Mean   :  4.842   Mean   :11.88  
 3rd Qu.: 5.200   3rd Qu.: 18.60   3rd Qu.:  5.400   3rd Qu.:14.10  
 Max.   :79.400   Max.   :217.10   Max.   :217.100   Max.   :42.90  
 NA's   :4038     NA's   :4038     NA's   :4038      NA's   :4038   
```

```
 F3pct_gluc3       F3pct_gsuc3      F3pct_gpol3     F3pct_lipi3
 Min.   :   0.00   Min.   :   0.0   Min.   : 0.00   Min.   : 5.70
 1st Qu.:  40.30   1st Qu.:  17.4   1st Qu.:17.30   1st Qu.:33.08
 Median :  45.90   Median :  22.7   Median :21.50   Median :38.05
 Mean   :  46.29   Mean   :  23.9   Mean   :22.23   Mean   :38.01
 3rd Qu.:  51.60   3rd Qu.:  28.3   3rd Qu.:26.70   3rd Qu.:42.60
 Max.   :1187.80   Max.   :1185.4   Max.   :77.00   Max.   :69.70
 NA's   :4038      NA's   :4038     NA's   :4038    NA's   :4038
 F3pct_lsat3     F3pct_lmon3    F3pct_lpol3     F3pct_fibr3
 Min.   : 0.00   Min.   : 0.0   Min.   : 0.00   Min.   :0.000
 1st Qu.:11.50   1st Qu.:12.9   1st Qu.: 4.10   1st Qu.:0.700
 Median :13.70   Median :15.3   Median : 4.90   Median :0.900
 Mean   :13.89   Mean   :15.7   Mean   : 5.08   Mean   :0.921
 3rd Qu.:16.00   3rd Qu.:18.1   3rd Qu.: 5.90   3rd Qu.:1.100
 Max.   :41.30   Max.   :39.1   Max.   :34.10   Max.   :2.700
 NA's   :4038    NA's   :4038   NA's   :4038    NA's   :4038
 F3pct_chol3        F3Fruits        F3Fruits_OK     F3Vegetables
 Min.   :  0.00   Min.   : 0.000   Min.   :0.000   Min.   : 0.000
 1st Qu.: 15.20   1st Qu.: 0.875   1st Qu.:0.000   1st Qu.: 0.964
 Median : 18.90   Median : 1.625   Median :0.000   Median : 1.393
 Mean   : 20.18   Mean   : 2.053   Mean   :0.423   Mean   : 1.611
 3rd Qu.: 23.20   3rd Qu.: 2.750   3rd Qu.:1.000   3rd Qu.: 1.987
 Max.   :127.10   Max.   :15.000   Max.   :1.000   Max.   :20.000
 NA's   :4038     NA's   :4157     NA's   :4157    NA's   :4162
 F3Vegetables_OK     F3Meat          F3Meat_OK         F3Fish
 Min.   :0.000   Min.   : 0.000   Min.   :0.000   Min.   : 0.000
 1st Qu.:0.000   1st Qu.: 2.625   1st Qu.:0.000   1st Qu.: 0.875
 Median :0.000   Median : 4.250   Median :1.000   Median : 1.500
 Mean   :0.076   Mean   : 4.801   Mean   :0.624   Mean   : 1.760
 3rd Qu.:0.000   3rd Qu.: 6.062   3rd Qu.:1.000   3rd Qu.: 2.375
 Max.   :1.000   Max.   :61.500   Max.   :1.000   Max.   :52.500
 NA's   :4162    NA's   :4167     NA's   :4167    NA's   :4142
  F3Fish_OK         F3Fish2          F3Fish2_OK       F3Dairy
 Min.   :0.000   Min.   : 0.000   Min.   :0.000   Min.   :0.000
 1st Qu.:0.000   1st Qu.: 0.500   1st Qu.:0.000   1st Qu.:0.607
 Median :1.000   Median : 0.875   Median :0.000   Median :1.089
 Mean   :0.684   Mean   : 1.153   Mean   :0.185   Mean   :1.297
 3rd Qu.:1.000   3rd Qu.: 1.500   3rd Qu.:0.000   3rd Qu.:1.679
 Max.   :1.000   Max.   :19.000   Max.   :1.000   Max.   :8.036
 NA's   :4142    NA's   :4123     NA's   :4123    NA's   :4179
  F3Dairy_OK        F3Nb_OK          F3Cat_OK        F3Recom_OK
 Min.   :0.000   Min.   :0.000   Min.   :0.000   Min.   :0.000
 1st Qu.:0.000   1st Qu.:1.000   1st Qu.:1.000   1st Qu.:0.000
 Median :0.000   Median :2.000   Median :2.000   Median :0.000
 Mean   :0.069   Mean   :1.877   Mean   :1.875   Mean   :0.242
 3rd Qu.:0.000   3rd Qu.:2.000   3rd Qu.:2.000   3rd Qu.:0.000
 Max.   :1.000   Max.   :5.000   Max.   :4.000   Max.   :1.000
 NA's   :4179    NA's   :4218    NA's   :4218    NA's   :4218
   F3Nb2_OK        F3Cat2_OK      F3Recom2_OK      F3Mediter1
 Min.   :0.000   Min.   :0.000   Min.   :0.000   Min.   :0.000
 1st Qu.:1.000   1st Qu.:1.000   1st Qu.:0.000   1st Qu.:3.000
 Median :1.000   Median :1.000   Median :0.000   Median :4.000
 Mean   :1.376   Mean   :1.375   Mean   :0.408   Mean   :3.945
 3rd Qu.:2.000   3rd Qu.:2.000   3rd Qu.:1.000   3rd Qu.:5.000
 Max.   :5.000   Max.   :4.000   Max.   :1.000   Max.   :8.000
 NA's   :4217    NA's   :4217    NA's   :4217    NA's   :4223
  F3Mediter2        F3AHEI1          F3AHEI2        F3vegetarian
 Min.   :0.000   Min.   : 4.00   Min.   : 4.00   Min.   :0.0000
 1st Qu.:3.000   1st Qu.:24.50   1st Qu.:25.00   1st Qu.:0.0000
 Median :5.000   Median :32.00   Median :32.00   Median :0.0000
 Mean   :4.483   Mean   :31.92   Mean   :31.97   Mean   :0.0043
 3rd Qu.:6.000   3rd Qu.:39.00   3rd Qu.:39.00   3rd Qu.:0.0000
 Max.   :9.000   Max.   :66.00   Max.   :66.00   Max.   :1.0000
 NA's   :4757    NA's   :4204    NA's   :4199    NA's   :2995
```

```
      FAM              male           edtyp4_new          F0_mmesc
 Min.   :50001   Min.   :0.0000   Length:6746        Length:6746
 1st Qu.:51676   1st Qu.:0.0000   Class :character   Class :character
 Median :53325   Median :0.0000   Mode  :character   Mode  :character
 Mean   :53566   Mean   :0.4507
 3rd Qu.:55135   3rd Qu.:1.0000
 Max.   :60652   Max.   :1.0000
 NA's   :3105    NA's   :3105
  F0_CL_age          mmesc_fup0          F1_mmesc           F1_CL_age
 Length:6746        Length:6746        Length:6746        Length:6746
 Class :character   Class :character   Class :character   Class :character
 Mode  :character   Mode  :character   Mode  :character   Mode  :character



  mmesc_fup1           F1_Age          F1_cur_mddpd2        F1_QPC_A
 Length:6746        Length:6746        Length:6746        Length:6746
 Class :character   Class :character   Class :character   Class :character
 Mode  :character   Mode  :character   Mode  :character   Mode  :character



   F1_QPC_B           F1_QPC_1           F1_QPC_2           F1_QPC_3
 Length:6746        Length:6746        Length:6746        Length:6746
 Class :character   Class :character   Class :character   Class :character
 Mode  :character   Mode  :character   Mode  :character   Mode  :character



   F1_QPC_4           F1_QPC_5           F1_QPC_6           F1_QPC_7
 Length:6746        Length:6746        Length:6746        Length:6746
 Class :character   Class :character   Class :character   Class :character
 Mode  :character   Mode  :character   Mode  :character   Mode  :character



   F1_QPC_8            F1_CDR           F1_FV_A_TOT        F1_FV_FA_TOT
 Length:6746        Length:6746        Length:6746        Length:6746
 Class :character   Class :character   Class :character   Class :character
 Mode  :character   Mode  :character   Mode  :character   Mode  :character



 F1_FV_P_TOT         F1_B16_ID           F1_B16_RI          F1_B16_S1RL
 Length:6746        Length:6746        Length:6746        Length:6746
 Class :character   Class :character   Class :character   Class :character
 Mode  :character   Mode  :character   Mode  :character   Mode  :character



 F1_B16_S1RI        F1_B16_S2RL        F1_B16_S2RI        F1_B16_S3RL
 Length:6746        Length:6746        Length:6746        Length:6746
 Class :character   Class :character   Class :character   Class :character
 Mode  :character   Mode  :character   Mode  :character   Mode  :character
```

```
  F1_B16_S3RI        F1_B16_EDRL        F1_B16_EDRI         F1_B16_REC
 Length:6746        Length:6746        Length:6746        Length:6746
 Class :character   Class :character   Class :character   Class :character
 Mode  :character   Mode  :character   Mode  :character   Mode  :character

 F1_STROOP_D        F1_STROOP_D_T      F1_STROOP_IFAIBLE  F1_STROOP_IFAIBLE_T
 Length:6746        Length:6746        Length:6746        Length:6746
 Class :character   Class :character   Class :character   Class :character
 Mode  :character   Mode  :character   Mode  :character   Mode  :character

 F1_STROOP_IFORTE   F1_STROOP_IFORTE_T   F1_DO40          F1_CERAD_CERCLE
 Length:6746        Length:6746        Length:6746        Length:6746
 Class :character   Class :character   Class :character   Class :character
 Mode  :character   Mode  :character   Mode  :character   Mode  :character

 F1_CERAD_RECTANLES F1_CERAD_LOSANGE   F1_CERAD_CUBE      F1_CERAD_TOT
 Length:6746        Length:6746        Length:6746        Length:6746
 Class :character   Class :character   Class :character   Class :character
 Mode  :character   Mode  :character   Mode  :character   Mode  :character

 cogn_psy_fup1        F2_mmesc           F2_CL_age           mmesc_fup2
 Length:6746        Length:6746        Length:6746        Length:6746
 Class :character   Class :character   Class :character   Class :character
 Mode  :character   Mode  :character   Mode  :character   Mode  :character

    F2_Age          F2_cur_mddpd2        F2_QPC_A            F2_QPC_B
 Length:6746        Length:6746        Length:6746        Length:6746
 Class :character   Class :character   Class :character   Class :character
 Mode  :character   Mode  :character   Mode  :character   Mode  :character

   F2_QPC_1           F2_QPC_2           F2_QPC_3            F2_QPC_4
 Length:6746        Length:6746        Length:6746        Length:6746
 Class :character   Class :character   Class :character   Class :character
 Mode  :character   Mode  :character   Mode  :character   Mode  :character

   F2_QPC_5           F2_QPC_6           F2_QPC_7            F2_QPC_8
 Length:6746        Length:6746        Length:6746        Length:6746
 Class :character   Class :character   Class :character   Class :character
 Mode  :character   Mode  :character   Mode  :character   Mode  :character
```

```
    F2_CDR           F2_FV_A_TOT        F2_FV_FA_TOT       F2_FV_P_TOT       
 Length:6746        Length:6746        Length:6746        Length:6746       
 Class :character   Class :character   Class :character   Class :character  
 Mode  :character   Mode  :character   Mode  :character   Mode  :character  
                                                                            
                                                                            
                                                                            
   F2_B16_ID          F2_B16_RI         F2_B16_S1RL        F2_B16_S1RI       
 Length:6746        Length:6746        Length:6746        Length:6746       
 Class :character   Class :character   Class :character   Class :character  
 Mode  :character   Mode  :character   Mode  :character   Mode  :character  
                                                                            
                                                                            
                                                                            
 F2_B16_S2RL        F2_B16_S2RI        F2_B16_S3RL        F2_B16_S3RI       
 Length:6746        Length:6746        Length:6746        Length:6746       
 Class :character   Class :character   Class :character   Class :character  
 Mode  :character   Mode  :character   Mode  :character   Mode  :character  
                                                                            
                                                                            
                                                                            
 F2_B16_EDRL        F2_B16_EDRI          F2_B16_REC        F2_STROOP_D       
 Length:6746        Length:6746        Length:6746        Length:6746       
 Class :character   Class :character   Class :character   Class :character  
 Mode  :character   Mode  :character   Mode  :character   Mode  :character  
                                                                            
                                                                            
                                                                            
 F2_STROOP_D_T      F2_STROOP_IFAIBLE  F2_STROOP_IFAIBLE_T F2_STROOP_IFORTE  
 Length:6746        Length:6746        Length:6746         Length:6746       
 Class :character   Class :character   Class :character    Class :character  
 Mode  :character   Mode  :character   Mode  :character    Mode  :character  
                                                                             
                                                                             
                                                                             
 F2_STROOP_IFORTE_T   F2_DO40          F2_CERAD_CERCLE    F2_CERAD_RECTANLES
 Length:6746        Length:6746        Length:6746        Length:6746       
 Class :character   Class :character   Class :character   Class :character  
 Mode  :character   Mode  :character   Mode  :character   Mode  :character  
                                                                            
                                                                            
                                                                            
 F2_CERAD_LOSANGE   F2_CERAD_CUBE      F2_CERAD_TOT       cogn_psy_fup2     
 Length:6746        Length:6746        Length:6746        Length:6746       
 Class :character   Class :character   Class :character   Class :character  
 Mode  :character   Mode  :character   Mode  :character   Mode  :character  
                                                                            
                                                                            
                                                                            
   F3_mmesc           F3_CL_age           mmesc_fup3           F3_Age         
 Length:6746        Length:6746        Length:6746        Length:6746       
 Class :character   Class :character   Class :character   Class :character  
 Mode  :character   Mode  :character   Mode  :character   Mode  :character  
```

```
 F3_cur_mddpd2       F3_QPC_A           F3_QPC_B           F3_QPC_1        
 Length:6746        Length:6746        Length:6746        Length:6746       
 Class :character   Class :character   Class :character   Class :character  
 Mode  :character   Mode  :character   Mode  :character   Mode  :character  
                                                                            
                                                                            
                                                                            
   F3_QPC_2           F3_QPC_3           F3_QPC_4           F3_QPC_5        
 Length:6746        Length:6746        Length:6746        Length:6746       
 Class :character   Class :character   Class :character   Class :character  
 Mode  :character   Mode  :character   Mode  :character   Mode  :character  
                                                                            
                                                                            
                                                                            
   F3_QPC_6           F3_QPC_7           F3_QPC_8            F3_CDR         
 Length:6746        Length:6746        Length:6746        Length:6746       
 Class :character   Class :character   Class :character   Class :character  
 Mode  :character   Mode  :character   Mode  :character   Mode  :character  
                                                                            
                                                                            
                                                                            
 F3_FV_A_TOT        F3_FV_FA_TOT       F3_FV_P_TOT         F3_B16_ID        
 Length:6746        Length:6746        Length:6746        Length:6746       
 Class :character   Class :character   Class :character   Class :character  
 Mode  :character   Mode  :character   Mode  :character   Mode  :character  
                                                                            
                                                                            
                                                                            
  F3_B16_RI         F3_B16_S1RL        F3_B16_S1RI        F3_B16_S2RL       
 Length:6746        Length:6746        Length:6746        Length:6746       
 Class :character   Class :character   Class :character   Class :character  
 Mode  :character   Mode  :character   Mode  :character   Mode  :character  
                                                                            
                                                                            
                                                                            
 F3_B16_S2RI        F3_B16_S3RL        F3_B16_S3RI        F3_B16_EDRL       
 Length:6746        Length:6746        Length:6746        Length:6746       
 Class :character   Class :character   Class :character   Class :character  
 Mode  :character   Mode  :character   Mode  :character   Mode  :character  
                                                                            
                                                                            
                                                                            
 F3_B16_EDRI         F3_B16_REC        F3_STROOP_D        F3_STROOP_D_T     
 Length:6746        Length:6746        Length:6746        Length:6746       
 Class :character   Class :character   Class :character   Class :character  
 Mode  :character   Mode  :character   Mode  :character   Mode  :character  
                                                                            
                                                                            
                                                                            
 F3_STROOP_IFAIBLE  F3_STROOP_IFAIBLE_T F3_STROOP_IFORTE   F3_STROOP_IFORTE_T
 Length:6746        Length:6746         Length:6746        Length:6746       
 Class :character   Class :character    Class :character   Class :character  
 Mode  :character   Mode  :character    Mode  :character   Mode  :character  
```

```
  F3_DO40          F3_CERAD_CERCLE    F3_CERAD_RECTANLES F3_CERAD_LOSANGE
 Length:6746       Length:6746        Length:6746        Length:6746
 Class :character  Class :character   Class :character   Class :character
 Mode  :character  Mode  :character   Mode  :character   Mode  :character
 
 
 
 
 F3_CERAD_CUBE     F3_CERAD_TOT       cogn_psy_fup3
 Length:6746       Length:6746        Length:6746
 Class :character  Class :character   Class :character
 Mode  :character  Mode  :character   Mode  :character
```

Recoding baseline covariates

Sex (sex), MMSE (MME) and occupation (job_curr8), total calories (F1sumtot1, F2sumtot1), smoking (F1sbsmk), bmi (F1BMI), depression (F1depressed), diabetes (F1dbtld) and the cognitive tests are coded as needed in the original dataset. See below.

In [44]:

```
ch <- ch %>% dplyr::rename(MMSE_b = F1MME,
                          occ_b = F1job_curr8,
                          bmi_b = F1BMI,
                          sm_b = F1sbsmk,
                          depre_b = F1depressed,
                          totalcal_b = F1sumtot1,
                          F2totalcal = F2sumtot1,
                          famincome_b = F2income2, #many NAs
                          F1datquest = F1datblood,
                          diab_b = F1dbtld,
                          memory_b = F1_B16_REC,#max 48
                          verbal_b = F1_FV_A_TOT,
                          stroop_b = F1_STROOP_IFORTE,#max 24
                          do40_b = F1_DO40, #max 40
                          CERAD_b = F1_CERAD_TOT, #max 11
                          CDR_b = F1_CDR,
                          F2memory = F2_B16_REC,#max 48
                          F2verbal = F2_FV_A_TOT,
                          F2stroop = F2_STROOP_IFORTE,#max 24
                          F2do40 = F2_DO40, #max 40
                          F2CERAD = F2_CERAD_TOT, #max 11
                          F2CDR = F2_CDR,
                          F3memory = F3_B16_REC,#max 48
                          F3verbal = F3_FV_A_TOT,
                          F3stroop = F3_STROOP_IFORTE,#max 24
                          F3do40 = F3_DO40, #max 40
                          F3CERAD = F3_CERAD_TOT, #max 11
                          F3CDR = F3_CDR)
ch[ch == "."] <- NA

ch$sm_b <- as.factor(ch$sm_b)
ch$occ_b <- as.factor(ch$occ_b)
ch$famincome_b <- as.factor(ch$famincome_b)

# Recode cognitive funciton variables as numeric
ch <- ch %>%
        mutate_at(c("F3_QPC_B","F3_QPC_A","F3_QPC_1","F3_QPC_2","F3_QPC_3","F3_QPC_
                    'F3_QPC_8', 'F3_QPC_7',"F3_QPC_6","F2_QPC_B","F2_QPC_A","F2_QP(
                    "F2_QPC_4","F2_QPC_5",'F2_QPC_8', 'F2_QPC_7',"F2_QPC_6","F1_QP(
```

```
                    "F1_QPC_2","F1_QPC_3","F1_QPC_4","F1_QPC_5",'F1_QPC_8', 'F1_QP(
                    "stroop_b","do40_b","CERAD_b","F2memory","F2verbal","F2stroop",
                    "F3verbal","F3stroop", "F3do40", "F3CERAD"), as.numeric)

#We pool the few participants with mild cognitive impairment and recode them to ver
ch$F2CDR[ch$F2CDR == "1.0"] <- "0.5"
```

Education

In [45]:
```
ch <- ch %>% mutate(edu = ifelse(edtyp == 0 |edtyp == 1 | edtyp == 2, 1, #elementa
                                ifelse(edtyp == 3, 2,                   #high sch
                                      ifelse(edtyp == 4, 3, NA)))         #supe

ch$edu <- factor(ch$edu,
levels = c(1,2,3),
labels = c("Elementary", "High school", "Superior"))
```

BMI categorical

In [46]:
```
ch$bmi_cat <- as.factor(ifelse(ch$bmi_b < 25, 1,
                    ifelse(ch$bmi_b < 30, 2,
                          ifelse(ch$bmi_b >= 30, 3, NA))))

table(ch$bmi_cat)
```

```
   1    2    3
2180 1960  859
```

Age at recruitment

In [47]:
```
rep_str = c('jan'='-01-','feb'='-02-','mar'='-03-','apr'='-04-','may'='-05-','jun'=
            'sep'='-09-', 'oct'='-10-','nov'='-11-','dec'='-12-')
ch$datbirth <- str_replace_all(ch$datbirth, rep_str)
ch$datexam <- str_replace_all(ch$datexam, rep_str)
ch$F1datquest <- str_replace_all(ch$F1datquest, rep_str)

table(is.na(ch$datbirth))

ch$datbirth <- as.Date(ch$datbirth,
  format = "%d-%m-%Y")
ch$datexam <- as.Date(ch$datexam,
  format = "%d-%m-%Y")
ch$F1datquest <- as.Date(ch$F1datquest,
  format = "%d-%m-%Y")

ch$age_recruit <- difftime(ch$datexam, ch$datbirth, units = "days")
ch$age_recruit <- as.integer(round(ch$age_recruit/365,digits = 0))

ch$age_b <- difftime(ch$F1datquest, ch$datbirth, units = "days")
ch$age_b <- as.integer(round(ch$age_b/365,digits = 0))

ch <- ch %>%
    dplyr::mutate(age_cat = as.factor(ifelse(age_b < 70, 1,
                                ifelse(age_b < 75, 2,
                                      ifelse(age_b >= 75, 3, NA)))))
```

```
FALSE  TRUE
 6733    13
```

Past major cardiovascular event

In [48]:
```
ch <- ch %>% mutate(cvevent_b = ifelse(cmp == 1 | hdc == 1 |hdv == 1 | chf == 1 |c
                                       strk == 1 | ccth == 1 | cabg == 1 |pcin == 
                                       F1cmp == 1 | F1hdv == 1 | F1chf == 1 |F1cad
                                       F1strk == 1 | F1ccth == 1 | F1cabg == 1 , 1
```

Hypertension

In [49]:
```
ch <- ch %>% mutate(HTA_b = ifelse(antiHTA == 1 | HTA == 1 | F1antiHTA == 1 | F1HT
```

Alcohol use/day

In [50]:
```
ch <- ch %>% mutate(alc_b = ifelse(alcool1 == 0, 0,
                                   ifelse(sex == 0 & F1alcool1 == 1, 1,
                                      ifelse(sex == 0 & (F1alcool1 == 2 | F1alcool
                                        ifelse(sex == 0 & (F1alcool1 == 4 | F1alco
                                            ifelse(sex == 1 & (F1alcool1 == 1|F1al
                                                ifelse(sex == 1 & (F1alcool1 == 3
                                                    ifelse(sex == 1 & (F1alcool1 =
```

Physical activity

In [51]:
```
# Find tertiles
vTert = quantile(na.omit(ch$F1etsem), c(0:3/3))

ch <- ch %>% mutate(pa_b = ifelse(is.na(F1etsem), NA,
                                 ifelse(F1etsem < 16200.16, "Low",
                                       ifelse(F1etsem < 19807.82, "Medium",
                                             ifelse(F1etsem < 44913.35, "High"))))
```

Time after recruitment

In [52]:
```
ch["F2datquest"][ch["F2datquest"] == "03jul2004"] <- "03jul2014"

ch$F2datquest <- str_replace_all(ch$F2datquest, rep_str)
ch$F3datquest <- str_replace_all(ch$F3datquest, rep_str)

ch$F2datquest <- as.Date(ch$F2datquest,
  format = "%d-%m-%Y")
ch$F3datquest <- as.Date(ch$F3datquest,
  format = "%d-%m-%Y")

ch$futime_b <- difftime(ch$F1datquest, ch$datexam, units = "days")
ch$futime_b <- as.numeric(round(ch$futime_b/365,digits = 2))

ch$futime_F1 <- difftime(ch$F2datquest, ch$F1datquest, units = "days")
ch$futime_F1 <- as.numeric(round(ch$futime_F1/365,digits = 2))

ch$futime_F2 <- difftime(ch$F3datquest, ch$F1datquest, units = "days")
ch$futime_F2 <- as.numeric(round(ch$futime_F2/365,digits = 2))
```

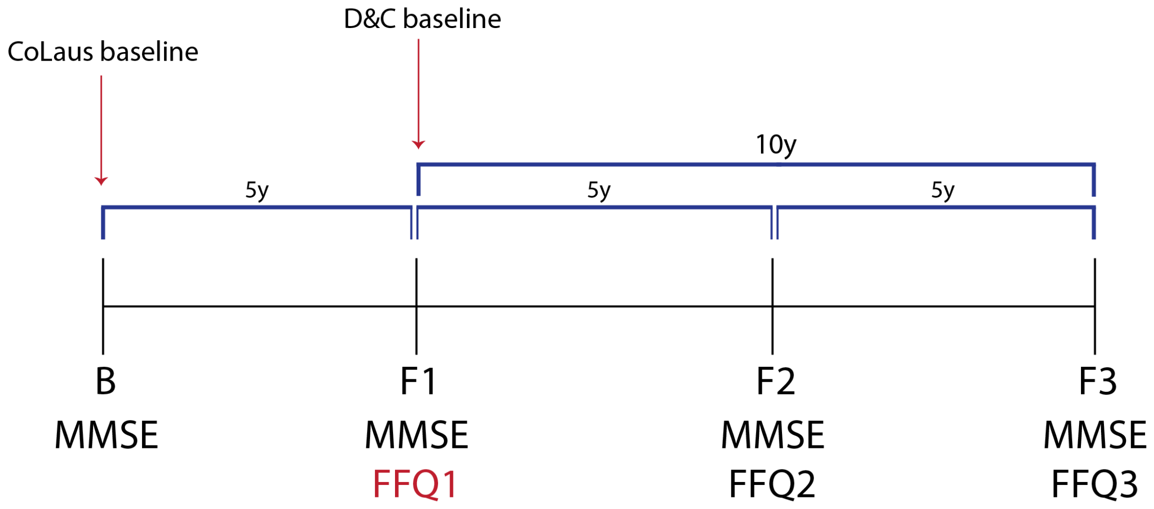

In [53]:
```
#Follow-up times (mean and 95% CI)

min(na.omit(c(ch$futime_b))) #5.57 (5.56-5.58) years
mean(na.omit(c(ch$futime_F1))) #10.86 (10.85-10.88) years
mean(na.omit(c(ch$futime_F2))) #14.59 (14.58-14.61) years

t.test(na.omit(c(ch$futime_b)))$"conf.int"
t.test(na.omit(c(ch$futime_F1)))$"conf.int"
t.test(na.omit(c(ch$futime_F2)))$"conf.int"
```

4.52

5.27936437718277

9.04193557748114

5.55559762534958 · 5.58077678223336

5.26214785011949 · 5.29658090424605

9.02407560415633 · 9.05979555080594

In [54]:
```
#pdf(file = "a.pdf",
#    width = 4,
#    height = 4)

fut1 <- data.frame(FUT = c(ch$futime_F1), tp = c("F1"))
fut2 <- data.frame(FUT = c(ch$futime_F2), tp = c("F2"))
id <- c(1:13609)
follow_ups <- bind_rows(fut1,fut2)


ggplot(follow_ups, aes(x = FUT))+
          geom_density(aes(fill = tp), alpha = 0.4) +
          xlim(0, 13)+
          scale_color_manual(values = c("#00AFBB","#868686FF", "#EFC000FF"))+
          scale_fill_manual(values = c("#00AFBB","#868686FF", "#EFC000FF"))

#dev.off()
```

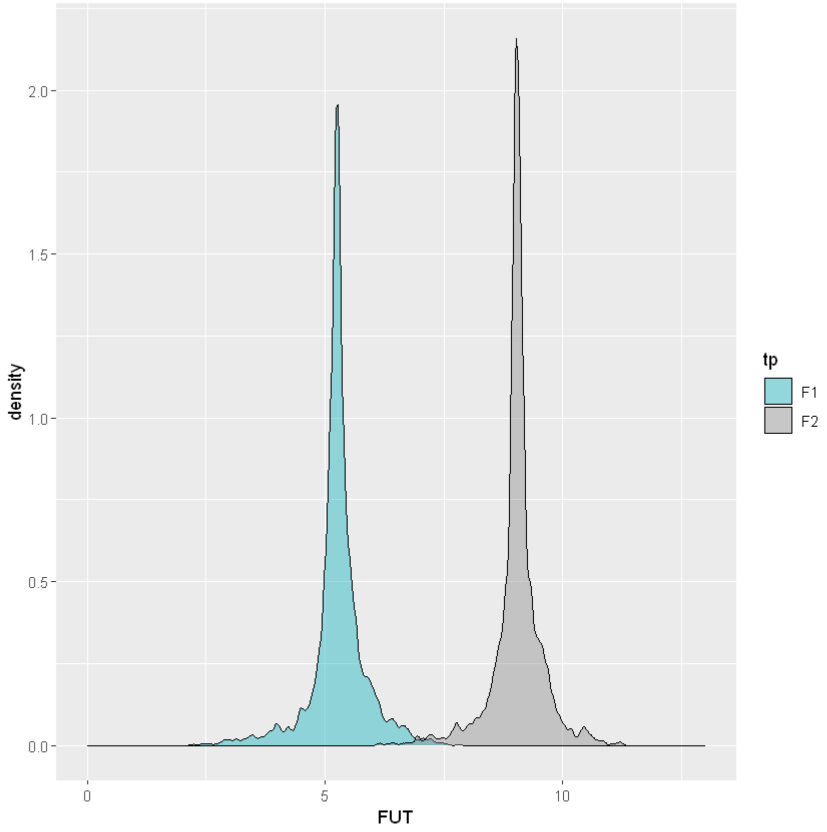

3. Selection criteria

PsyCoLaus inclusion criteria

All 35 to 66-year old subjects of the CoLaus sample (n = 5,535), were invited by letters to also participate in the psychiatric evaluation. Those who did not respond to the letter were contacted by phone. All subjects who were sufficiently fluent in French or English and agreed to participate were included into the PsyCoLaus sub-study and underwent the psychiatric assessment between 2004 and 2008.

Inclusion criteria

- >55 years old at baseline
- They signed the agreement of further use of the study data.
- They underwent a baseline dietary assessment at least 3 years before the cognitive function assessment.

Participant Exclusion Criteria

- The negation of the above
- Cognitive impairment at baseline MMSE < 24
- Caloric intake outside the normal range of 500 and 4200.

In [55]:
```
ch_sc <- ch %>% #6733 at ch
         filter(!is.na(MMSE_b)) %>% #n=1995
```

```
        filter((sex == 1 & totalcal_b > 799 & totalcal_b < 4001)|(sex == 0 & totalc
        filter(age_b > 55)%>% #<55 years old at baseline (n=0)
        filter(MMSE_b > 23)#baseline dietary assessment at least 3 years before the


#Assessment of missing baseline variables
table(ch_sc$age_b) # 0 missing
table(is.na(ch_sc$sex)) # 0 missing
table(is.na(ch_sc$edu)) # 0 missing
table(is.na(ch_sc$occ_b)) # 35 missing
table(is.na(ch_sc$bmi_b)) # 19 missing
table(is.na(ch_sc$sm_b)) # 5 missing
table(is.na(ch_sc$depre_b))  # 161 missing
summary(ch_sc$totalcal_b) # # 0 missing
table(is.na(ch_sc$famincome_b)) # 619 missing!!!!!!!!
table(is.na(ch_sc$diab_b)) # 0 missing
table(is.na(ch_sc$cvevent_b)) # 4 missing
table(is.na(ch_sc$HTA_b)) # 2 missing
table(is.na(ch_sc$alc_b)) # 0 missing
table(is.na(ch_sc$pa_b)) # 218 missing!!!!!!!!
table(is.na(ch_sc$MMSE_b)) # 0 missing

summary(ch_sc$futime_F1)

table(ch_sc$F2MME)
table(ch_sc$F2CDR)
```

```
 59  60  61  62  63  64  65  66  67  68  69  70  71  72  73  74  75  76  77  78 
  2  56  84 108 115 127 114 125 135 106  92 122  86  76  46  55  38  49  47  57 
 79  80  81  82 
 44  54   5   2 
FALSE 
 1745 
FALSE 
 1745 
FALSE  TRUE 
 1710    35 
FALSE  TRUE 
 1726    19 
FALSE  TRUE 
 1740     5 
FALSE  TRUE 
 1584   161 
   Min. 1st Qu.  Median    Mean 3rd Qu.    Max. 
  517.3  1262.3  1612.0  1685.1  2052.6  3767.0 
FALSE  TRUE 
 1126   619 
FALSE 
 1745 
FALSE  TRUE 
 1741     4 
FALSE  TRUE 
 1743     2 
FALSE  TRUE 
 1589   156 
FALSE  TRUE 
 1527   218 
FALSE 
 1745 
   Min. 1st Qu.  Median    Mean 3rd Qu.    Max.    NA's 
  2.240   5.140   5.270   5.272   5.430   7.430     245 
  0   1   9  15  21  22  23  24  25  26  27  28  29  30 
  1   5   1   1   2   3   3   5  16  36  78 169 344 673 
```

```
0.0 0.5
487 426
```

In [16]:

```
ch_sc #N= 1745
id_FU2 <- ch_sc %>% filter(futime_F1 < 3)
length(id_FU2 <- c(id_FU2$pt)) #there are 8 people with a follow-up lower than 3 ye
```

A data.frame: 174

pt	sex	datbirth	datexam	bthpl_dem	ethori_self	edtyp	edlv	mrtsts	sclhlp	⋯	age_re
<int>	<int>	<date>	<date>	<chr>	<chr>	<int>	<int>	<int>	<int>	⋯	[illegible]
6	0	1943-01-30	2003-07-08	SZ	W	1	12	3	0	⋯	
7	0	1933-03-26	2003-07-04	EY	W	0	8	0	1	⋯	
19	1	1948-01-23	2003-08-04	SZ	W	1	11	2	0	⋯	
20	0	1943-05-26	2003-07-16	SZ	W	3	14	2	0	⋯	
24	1	1936-09-16	2003-07-14	PD	W	3	50	2	1	⋯	
25	0	1943-12-28	2003-08-28	SZ	W	1	12	3	0	⋯	
28	1	1948-04-29	2003-07-24	SZ	W	3	14	0	0	⋯	
31	0	1948-03-10	2003-07-23	EG	W	2	24	2	0	⋯	
46	0	1942-11-07	2003-07-31	SZ	W	0	10	0	0	⋯	
48	1	1947-05-05	2003-08-13	SZ	W	2	16	2	0	⋯	
51	0	1948-10-16	2003-09-01	SZ	W	1	14	NA	0	⋯	
55	0	1943-12-26	2003-09-09	FR	W	2	18	0	0	⋯	
56	1	1939-01-01	2003-08-04	SZ	W	3	12	0	1	⋯	
68	0	1940-07-10	2003-08-05	SZ	W	0	10	3	0	⋯	
72	0	1941-06-09	2003-08-08	RM	W	2	18	0	0	⋯	
74	0	1946-11-15	2003-09-09	SZ	W	0	9	3	0	⋯	
76	1	1939-05-23	2003-07-31	SZ	W	2	16	0	0	⋯	
80	1	1944-05-04	2003-07-29	SZ	W	1	14	0	0	⋯	
84	1	1949-01-14	2003-08-05	SZ	W	1	14	0	0	⋯	
85	0	1945-11-02	2003-08-05	FR	W	2	20	0	0	⋯	
87	1	1943-04-30	2003-08-12	SZ	W	3	15	0	0	⋯	
95	1	1945-10-03	2003-08-20	SZ	W	3	21	2	0	⋯	

pt	sex	datbirth	datexam	bthpl_dem	ethori_self	edtyp	edlv	mrtsts	sclhlp	···	age_re
<int>	<int>	<date>	<date>	<chr>	<chr>	<int>	<int>	<int>	<int>	···	<
102	0	1943-11-22	2003-08-25	SZ	W	1	15	2	0	···	
103	0	1946-02-04	2003-08-19	SZ	W	3	15	0	0	···	
112	1	1929-11-24	2003-09-02	SZ	W	0	10	0	1	···	
119	0	1945-10-10	2003-08-26	SZ	W	1	12	2	0	···	
121	1	1945-07-17	2003-08-25	FR	W	1	13	0	0	···	
122	1	1949-05-27	2003-08-25	SZ	W	4	16	0	0	···	
128	0	1933-08-27	2003-08-22	SZ	W	1	12	0	1	···	
130	1	1943-09-06	2003-08-26	CT	X	1	12	2	1	···	
⋮	⋮	⋮	⋮	⋮	⋮	⋮	⋮	⋮	⋮	⋱	
9291	0	1943-03-12	2005-02-22	AN	O	2	11	2	0	···	
9292	0	1939-07-17	2005-02-02	CO	O	0	12	0	1	···	
9298	1	1932-07-11	2005-01-10	AG	B	4	18	2	1	···	
9316	1	1936-12-04	2005-01-31	BN	B	4	18	0	0	···	
9323	1	1944-02-09	2005-02-04	MT	A	3	15	0	0	···	
9327	0	1933-03-24	2005-02-02	LE	A	2	12	3	1	···	
9336	0	1934-08-24	2005-02-18	CL	O	4	9	0	1	···	
9341	0	1946-12-20	2005-03-22	MT	B	0	5	0	0	···	
9344	0	1946-04-09	2005-03-03	RW	B	4	12	0	0	···	
9366	1	1946-08-20	2005-03-31	CI	O	4	25	0	0	···	
9374	1	1943-09-17	2005-04-11	CO	B	4	20	0	0	···	
9394	0	1948-09-30	2005-04-26	PR	O	3	13	0	0	···	
9397	0	1949-11-11	2005-05-04	MY	A	3	17	0	0	···	

pt	sex	datbirth	datexam	bthpl_dem	ethori_self	edtyp	edlv	mrtsts	sclhlp	⋯	age_re
<int>	<int>	<date>	<date>	<chr>	<chr>	<int>	<int>	<int>	<int>	⋯	
9413	0	1947-01-13	2005-05-18	X	O	0	8	0	0	⋯	
9429	1	1947-11-02	2005-06-06	X	O	3	18	0	0	⋯	
9440	1	1950-01-27	2005-06-29	AN	O	4	21	2	0	⋯	
9448	0	1941-04-05	2005-07-25	X	O	2	3	3	0	⋯	
9488	1	1943-03-14	2005-09-02	AG	O	4	12	2	0	⋯	
9490	1	1941-05-01	2005-09-13	TY	A	4	12	0	0	⋯	
9492	0	1946-08-19	2005-09-16	SZ	W	1	10	2	1	⋯	
9494	0	1950-09-29	2005-09-12	PS	A	4	17	0	0	⋯	
9520	0	1947-09-01	2005-11-01	TY	A	3	14	0	1	⋯	
9549	0	1948-06-21	2005-12-06	HT	O	2	18	NA	0	⋯	
9551	1	1940-10-23	2005-12-06	TY	A	4	17	0	1	⋯	
9565	0	1941-07-09	2006-01-12	IR	A	4	17	0	1	⋯	
9569	0	1944-06-18	2006-02-16	TU	B	0	9	2	1	⋯	
9573	1	1948-11-08	2006-01-04	CI	O	3	14	0	0	⋯	
9592	0	1943-06-07	2006-01-26	IR	A	1	12	2	1	⋯	
9604	1	1939-12-20	2006-02-06	CN	A	4	19	0	1	⋯	
9639	0	1944-09-12	2006-03-17	MT	B	1	10	3	0	⋯	

In [17]:

```
#Corrections sent by Pedro Marques-Vidal - email 08.09.22

ch_sc_look <- ch_sc %>% dplyr::select(pt, F2MME, F3MME) %>% filter(pt %in% id_FU2)
ch_sc_look

# Put the values of those followed up less than 3 years in the 1st follow up

ch_sc$F2MME[ch_sc$pt == 122] <- 30
ch_sc$F2MME[ch_sc$pt == 171] <- 30
ch_sc$F2MME[ch_sc$pt == 2058] <- 27
ch_sc$F2MME[ch_sc$pt == 1361] <- 27
ch_sc$F2MME[ch_sc$pt == 1734] <- 29
```

```
ch_sc$F2MME[ch_sc$pt == 1751] <- 29
ch_sc$F2MME[ch_sc$pt == 2235] <- 25
ch_sc$F2MME[ch_sc$pt == 5628] <- 29
ch_sc$F2MME[ch_sc$pt == 9604] <- 30
ch_sc$F3MME[ch_sc$pt == 122] <- NA
ch_sc$F3MME[ch_sc$pt == 171] <- NA
ch_sc$F3MME[ch_sc$pt == 2058] <- NA
ch_sc$F3MME[ch_sc$pt == 196] <- NA
ch_sc$F3MME[ch_sc$pt == 214] <- NA
ch_sc$F3MME[ch_sc$pt == 994] <- NA
ch_sc$F3MME[ch_sc$pt == 1477] <- NA
ch_sc$F3MME[ch_sc$pt == 9323] <- NA
ch_sc$F3MME[ch_sc$pt == 1383] <- 27
ch_sc$F3MME[ch_sc$pt == 3455] <- 30

#Check that it is correct
ch_sc_look <- ch_sc %>% dplyr::select(pt, F2MME, F3MME) %>% filter(pt %in% id_FU2)
ch_sc_look

#replace the time & outcome vars to the adequate tp
ch_sc$futime_F1[ch_sc$pt == 122] <- ch_sc$futime_F2
ch_sc$futime_F1[ch_sc$pt == 2058] <- ch_sc$futime_F2
ch_sc$futime_F1[ch_sc$pt == 196] <- ch_sc$futime_F2
ch_sc$futime_F1[ch_sc$pt == 214] <- ch_sc$futime_F2
ch_sc$futime_F1[ch_sc$pt == 994] <- ch_sc$futime_F2
ch_sc$futime_F1[ch_sc$pt == 1477] <- ch_sc$futime_F2
ch_sc$futime_F1[ch_sc$pt == 9323] <- ch_sc$futime_F2
ch_sc$futime_F2[ch_sc$pt == 171] <- NA

ch_sc$futime_F2[ch_sc$pt == 122] <- NA
ch_sc$futime_F2[ch_sc$pt == 171] <- NA
ch_sc$futime_F2[ch_sc$pt == 2058] <- NA
ch_sc$futime_F2[ch_sc$pt == 196] <- NA
ch_sc$futime_F2[ch_sc$pt == 214] <- NA
ch_sc$futime_F2[ch_sc$pt == 994] <- NA
ch_sc$futime_F2[ch_sc$pt == 1477] <- NA
ch_sc$futime_F2[ch_sc$pt == 9323] <- NA
```

A data.frame: 10 × 3

pt	F2MME	F3MME
<int>	<int>	<int>
122	28	30
171	NA	30
196	30	30
214	30	30
815	30	NA
865	30	NA
994	29	29
1477	29	29
2058	29	27
9323	29	29

A data.frame: 10 × 3

pt	F2MME	F3MME
<int>	<dbl>	<dbl>
122	30	NA
171	30	NA
196	30	NA
214	30	NA
815	30	NA
865	30	NA
994	29	NA
1477	29	NA
2058	27	NA
9323	29	NA

3.1 Not recruited for PsyColaus

In [18]:
```
included <- unique(c(ch_sc$pt))
length(included) #1347

total_included <- unique(c(ch$pt))
length(total_included)#6733

excluded <- setdiff(total_included, included)
length(excluded) #5269

ch <- ch %>% mutate(psy = ifelse(pt %in% included, 1, 0))
```

1745

6746

5001

3.2 Loss to follow-up

In [19]:
```
ch_baseline <- ch %>% #6733 at ch
        filter(!is.na(MMSE_b))%>%
        filter(age_b > 55)%>%#>55 years old at baseline (n=0)
        filter(MMSE_b > 23)%>%
        filter(futime_F1 > 3 |futime_F2 > 3)%>%#baseline dietary assessment at lea
        filter((sex == 1 & totalcal_b > 799 & totalcal_b < 4201)|(sex == 0 & total
        filter(!is.na(F2MME))

baseline_b <- unique(c(ch_baseline$pt))
length(baseline_b) #1347

excluded <- setdiff(included, baseline_b)
length(excluded) #432

ch_sc <- ch_sc %>% mutate(ltfu = ifelse(pt %in% baseline_b, 1,
                                 ifelse(pt %in% excluded, 0, NA)))
```

1334

411

In [20]:
```
table(ch_sc$ltfu)
```

```
   0    1 
 411 1334 
```

4. Data descriptives

4.1 Main outcome at each time point

Categorical cognitive measures

In [21]:
```
#CDR
table(ch_sc$CDR_b) #we cannot include participants with MCI at baseline in the surv
table(ch_sc$F2CDR)
table(ch_sc$F3CDR)

#SCD
ch_sc$SCD_b = ch_sc$F1_QPC_A+ch_sc$F1_QPC_B+ch_sc$F1_QPC_1+ch_sc$F1_QPC_2+ch_sc$F1_

ch_sc$F2SCD = ch_sc$F2_QPC_A+ch_sc$F2_QPC_B+ch_sc$F2_QPC_1+ch_sc$F2_QPC_2+ch_sc$F2_

ch_sc$F3SCD = ch_sc$F3_QPC_A+ch_sc$F3_QPC_B+ch_sc$F3_QPC_1+ch_sc$F3_QPC_2+ch_sc$F3_

ch_sc <- mutate(ch_sc, SCD_b_yn = ifelse(SCD_b >= 3 |F1_QPC_5==1|(F1_QPC_A+F1_QPC_
ch_sc <- mutate(ch_sc, F2SCD_yn = ifelse(F2SCD >= 3 |F2_QPC_5==1|(F2_QPC_A+F2_QPC_
ch_sc <- mutate(ch_sc, F3SCD_yn = ifelse(F3SCD >= 3 |F3_QPC_5==1|(F3_QPC_A+F3_QPC_

table(ch_sc$SCD_b_yn)
table(ch_sc$F2SCD_yn)
table(ch_sc$F3SCD_yn)
```

```
0.0 0.5 1.0 
509 465   1 
0.0 0.5 
487 426 
0.0 0.5 
247 181 
  0   1 
789 177 
  0   1 
793 179 
  0   1 
383  76 
```

Continuous cognitive measures

In [22]:
```
hist(ch_sc$F2memory,breaks=48) #max 48
hist(ch_sc$F2verbal,breaks=58) #max 58
t.test(ch_sc$F2verbal)
hist(ch_sc$F2stroop,breaks=24)#max 24
hist(ch_sc$F2do40,breaks=40)  #max 40
hist(ch_sc$F2CERAD,breaks=11)  #max 11
```

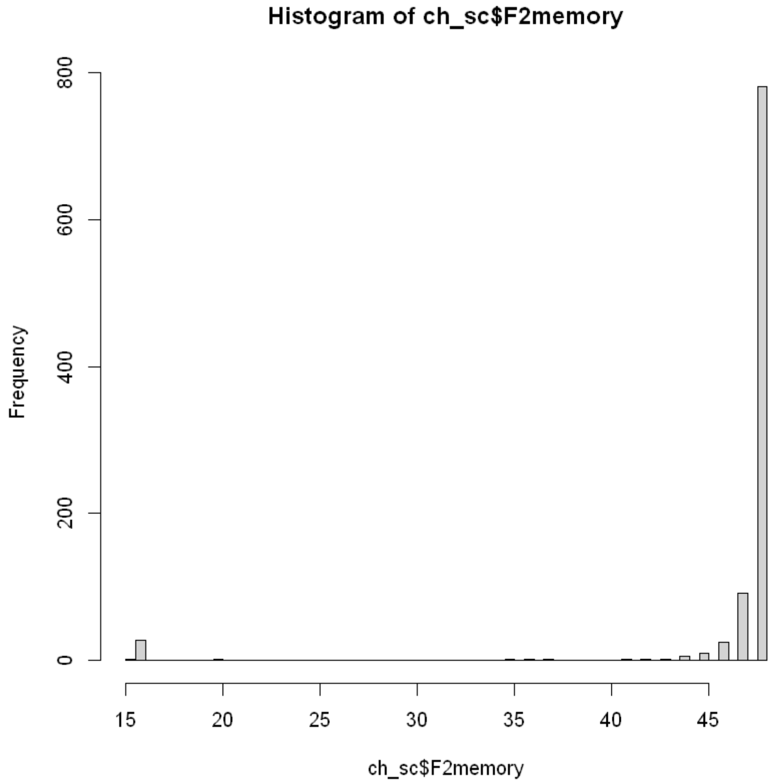


```
       One Sample t-test

data:  ch_sc$F2verbal
t = 108.75, df = 953, p-value < 2.2e-16
alternative hypothesis: true mean is not equal to 0
95 percent confidence interval:
 29.35363 30.43254
sample estimates:
mean of x 
 29.89308 
```

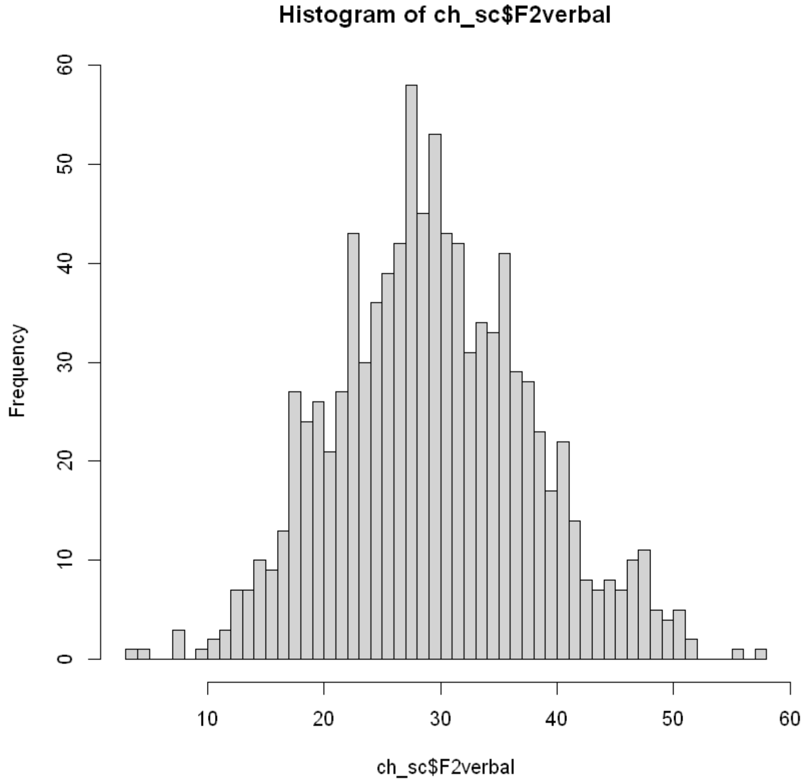

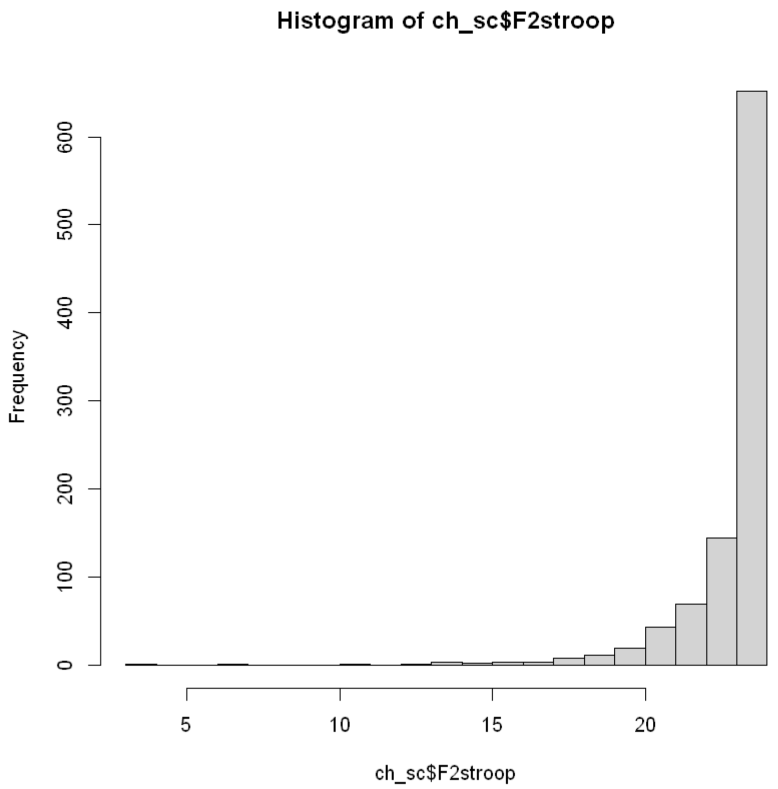
Histogram of ch_sc$F2stroop
Frequency
0
100
200
300
400
500
600
5
10
15
20
ch_sc$F2stroop

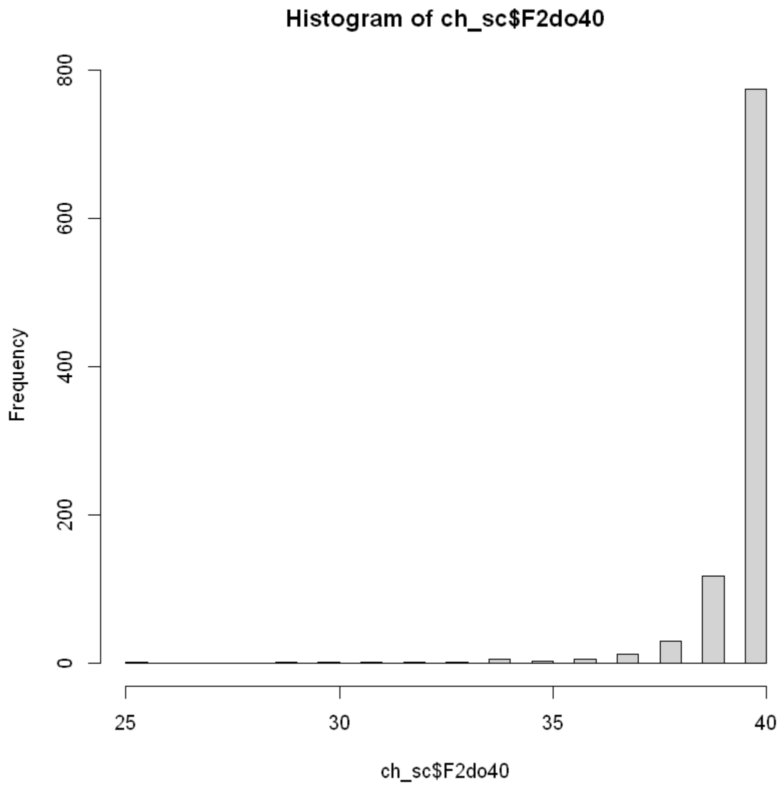
Histogram of ch_sc$F2do40
Frequency
0
200
400
600
800
25
30
35
40
ch_sc$F2do40

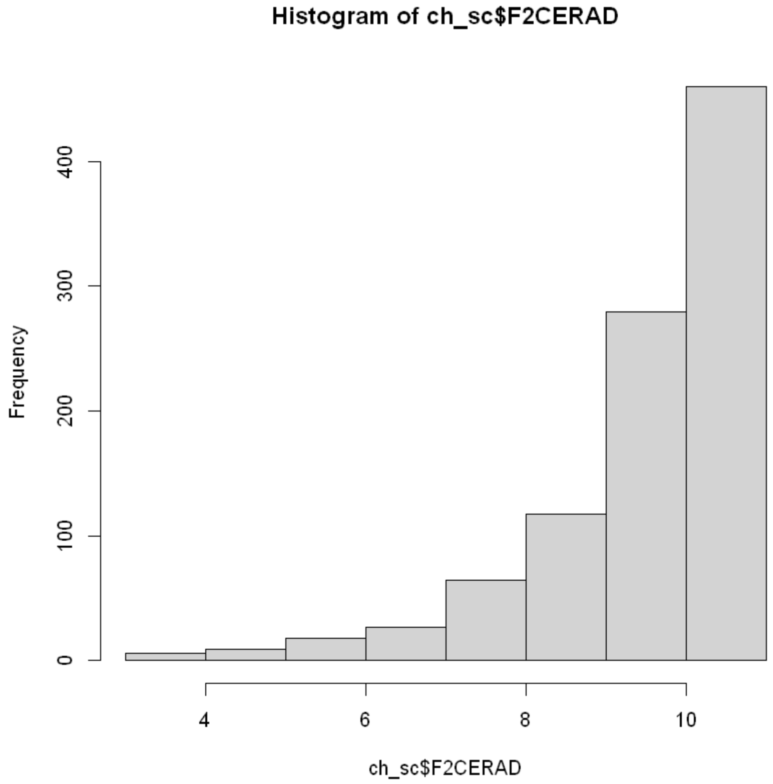

Dicotomization of continuous outcomes

In [23]:
```
quantile(na.omit(ch_sc$F2verbal), 0.75)

ch_sc <- mutate(ch_sc,
                memory_b_cat = ifelse(memory_b<48,1,0),
                verbal_b_cat =ifelse(verbal_b<30,1,0) ,
                stroop_b_cat = ifelse(stroop_b<24,1,0),
                do40_b_cat =ifelse(do40_b<40,1,0),
                CERAD_b_cat =ifelse(CERAD_b<11,1,0),
                F2memory_cat = ifelse(F2memory<48,1,0),
                F2verbal_cat =ifelse(F2verbal<36,1,0) ,
                F2stroop_cat = ifelse(F2stroop<24,1,0),
                F2do40_cat =ifelse(F2do40<40,1,0),
                F2CERAD_cat =ifelse(F2CERAD<11,1,0),
                F3memory_cat = ifelse(F3memory<48,1,0),
                F3verbal_cat =ifelse(F3verbal<= 36,1,0) ,
                F3stroop_cat = ifelse(F3stroop<24,1,0),
                F3do40_cat =ifelse(F3do40<40,1,0),
                F3CERAD_cat =ifelse(F3CERAD<11,1,0))

table(ch_sc$F2CDR)
table(ch_sc$F2SCD_yn)
table(ch_sc$F2verbal_cat)
table(ch_sc$F2memory_cat)
table(ch_sc$F2do40_cat)
table(ch_sc$F2stroop_cat)
table(ch_sc$F2CERAD_cat)
```

75%: 36

```
0.0 0.5
487 426
  0   1
793 179
  0   1
243 711
```

```
  0   1 
781 171 
  0   1 
774 181 
  0   1 
652 313 
  0   1 
460 520 
```

4.2 Main exposure at each time point

DAIRY

Continuous dairy intake and subtypes of dairy

In [24]:
```
#grams
ch_sc <- ch_sc %>%
        mutate(total_dairy_b = F1FFQ1amount+F1FFQ2amount+F1FFQ3amount+F1FFQ4amount
                    F1FFQ8amount+F1FFQ52amount+F1FFQ53amount+F1FFQ71amount+F1FFQ68
                    F1FFQ84amount+F1FFQ85amount+F1FFQ86amount+F1FFQ63amount,
                ferm_dairy_b = F1FFQ1amount+F1FFQ2amount+F1FFQ3amount+F1FFQ4amount
                    F1FFQ8amount,
                nonferm_dairy_b = F1FFQ52amount+F1FFQ53amount+F1FFQ71amount+F1FFQ6
                    F1FFQ84amount+F1FFQ85amount+F1FFQ86amount+F1FFQ63amount,
                fullfat_dairy_b = F1FFQ1amount+F1FFQ3amount+F1FFQ5amount+F1FFQ6amo
                    F1FFQ53amount+F1FFQ71amount+F1FFQ68amount+F1FFQ83amount+F1FFQ8
                nonfat_dairy_b = F1FFQ2amount+F1FFQ4amount+F1FFQ82amount+F1FFQ85am
                sugar_dairy_b = F1FFQ3amount+F1FFQ68amount+F1FFQ63amount,
               nonsug_dairy_b = F1FFQ1amount+F1FFQ2amount+F1FFQ4amount+F1FFQ5amoun
                    F1FFQ8amount+F1FFQ52amount+F1FFQ53amount+F1FFQ71amount+F1FFQ82
                    F1FFQ84amount+F1FFQ85amount+F1FFQ86amount,
                F2total_dairy = F2FFQ1amount+F2FFQ2amount+F2FFQ3amount+F2FFQ4amoun
                    F2FFQ8amount+F2FFQ52amount+F2FFQ53amount+F2FFQ71amount+F2FFQ68
                    F2FFQ84amount+F2FFQ85amount+F2FFQ86amount+F2FFQ63amount,
                F2ferm_dairy = F2FFQ1amount+F2FFQ2amount+F2FFQ3amount+F2FFQ4amount
                    F2FFQ8amount,
                F2nonferm_dairy = F2FFQ52amount+F2FFQ53amount+F2FFQ71amount+F2FFQ6
                    F2FFQ84amount+F2FFQ85amount+F2FFQ86amount+F2FFQ63amount,
                F2fullfat_dairy = F2FFQ1amount+F2FFQ3amount+F2FFQ5amount+F2FFQ6amo
                    F2FFQ53amount+F2FFQ71amount+F2FFQ68amount+F2FFQ83amount+F2FFQ8
                F2nonfat_dairy = F2FFQ2amount+F2FFQ4amount+F2FFQ82amount+F2FFQ85am
                F2sugar_dairy = F2FFQ3amount + F2FFQ68amount+F2FFQ63amount,
                F3total_dairy = F3FFQ1amount+F3FFQ2amount+F3FFQ3amount+F3FFQ4amoun
                    F3FFQ8amount+F3FFQ52amount+F3FFQ53amount+F3FFQ71amount+F3FFQ68
                    F3FFQ84amount+F3FFQ85amount+F3FFQ86amount+F3FFQ63amount,
                F3ferm_dairy = F3FFQ1amount+F3FFQ2amount+F3FFQ3amount+F3FFQ4amount
                    F3FFQ8amount,
                F3nonferm_dairy = F3FFQ52amount+F3FFQ53amount+F3FFQ71amount+F3FFQ6
                    F3FFQ84amount+F3FFQ85amount+F3FFQ86amount+F3FFQ63amount,
                F3fullfat_dairy = F3FFQ1amount+F3FFQ3amount+F3FFQ5amount+F3FFQ6amo
                    F3FFQ53amount+F3FFQ71amount+F3FFQ68amount+F3FFQ83amount+F3FFQ8
                F3nonfat_dairy = F3FFQ2amount+F3FFQ4amount+F3FFQ82amount+F3FFQ85am
                F3sugar_dairy = F3FFQ3amount + F3FFQ68amount+F3FFQ63amount)
```

In [40]:
```
summary(ch_sc$ferm_dairy_b)
summary(ch_sc$nonferm_dairy_b)
summary(ch_sc$fullfat_dairy_b)
summary(ch_sc$nonfat_dairy_b)
summary(ch_sc$sugar_dairy_b)
```

```
       pt             sex        datbirth             datexam          
 Min.   :6176   Min.   :1   Min.   :1941-07-29   Min.   :2006-01-24  
 1st Qu.:6176   1st Qu.:1   1st Qu.:1941-07-29   1st Qu.:2006-01-24  
 Median :6176   Median :1   Median :1941-07-29   Median :2006-01-24  
 Mean   :6176   Mean   :1   Mean   :1941-07-29   Mean   :2006-01-24  
 3rd Qu.:6176   3rd Qu.:1   3rd Qu.:1941-07-29   3rd Qu.:2006-01-24  
 Max.   :6176   Max.   :1   Max.   :1941-07-29   Max.   :2006-01-24  
                                                                     
  bthpl_dem         ethori_self            edtyp        edlv        mrtsts   
 Length:1           Length:1           Min.   :4   Min.   :16   Min.   : NA  
 Class :character   Class :character   1st Qu.:4   1st Qu.:16   1st Qu.: NA  
 Mode  :character   Mode  :character   Median :4   Median :16   Median : NA  
                                       Mean   :4   Mean   :16   Mean   :NaN  
                                       3rd Qu.:4   3rd Qu.:16   3rd Qu.: NA  
                                       Max.   :4   Max.   :16   Max.   : NA  
                                                                NA's   :1    
     sclhlp    job_curr1   job_curr4a   job_curr8      cmp          hdc   
 Min.   :0   Min.   :0   Min.   :2    Min.   :9   Min.   :0   Min.   :0  
 1st Qu.:0   1st Qu.:0   1st Qu.:2    1st Qu.:9   1st Qu.:0   1st Qu.:0  
 Median :0   Median :0   Median :2    Median :9   Median :0   Median :0  
 Mean   :0   Mean   :0   Mean   :2    Mean   :9   Mean   :0   Mean   :0  
 3rd Qu.:0   3rd Qu.:0   3rd Qu.:2    3rd Qu.:9   3rd Qu.:0   3rd Qu.:0  
 Max.   :0   Max.   :0   Max.   :2    Max.   :9   Max.   :0   Max.   :0  
                                                                         
      hdv         chf         artm         cad         angn         miac  
 Min.   :0   Min.   :0   Min.   :0   Min.   :0   Min.   :0   Min.   :0  
 1st Qu.:0   1st Qu.:0   1st Qu.:0   1st Qu.:0   1st Qu.:0   1st Qu.:0  
 Median :0   Median :0   Median :0   Median :0   Median :0   Median :0  
 Mean   :0   Mean   :0   Mean   :0   Mean   :0   Mean   :0   Mean   :0  
 3rd Qu.:0   3rd Qu.:0   3rd Qu.:0   3rd Qu.:0   3rd Qu.:0   3rd Qu.:0  
 Max.   :0   Max.   :0   Max.   :0   Max.   :0   Max.   :0   Max.   :0  
                                                                        
      strk         vslg         ccth         cabg         pcin      cvdbase  
 Min.   :0   Min.   :0   Min.   :0   Min.   :0   Min.   :0   Min.   :0  
 1st Qu.:0   1st Qu.:0   1st Qu.:0   1st Qu.:0   1st Qu.:0   1st Qu.:0  
 Median :0   Median :0   Median :0   Median :0   Median :0   Median :0  
 Mean   :0   Mean   :0   Mean   :0   Mean   :0   Mean   :0   Mean   :0  
 3rd Qu.:0   3rd Qu.:0   3rd Qu.:0   3rd Qu.:0   3rd Qu.:0   3rd Qu.:0  
 Max.   :0   Max.   :0   Max.   :0   Max.   :0   Max.   :0   Max.   :0  
                                                                        
  cvdbase_adj     alcool1      sbsmk       antiHTA       HTA         mnwlk   
 Min.   : NA   Min.   :0   Min.   :1   Min.   :0   Min.   :1   Min.   :45  
 1st Qu.: NA   1st Qu.:0   1st Qu.:1   1st Qu.:0   1st Qu.:1   1st Qu.:45  
 Median : NA   Median :0   Median :1   Median :0   Median :1   Median :45  
 Mean   :NaN   Mean   :0   Mean   :1   Mean   :0   Mean   :1   Mean   :45  
 3rd Qu.: NA   3rd Qu.:0   3rd Qu.:1   3rd Qu.:0   3rd Qu.:1   3rd Qu.:45  
 Max.   : NA   Max.   :0   Max.   :1   Max.   :0   Max.   :1   Max.   :45  
 NA's   :1                                                                 
     phyact        MME         handgrip           ht             wt      
 Min.   :2   Min.   : NA   Min.   :38.1   Min.   :172   Min.   :74.8  
 1st Qu.:2   1st Qu.: NA   1st Qu.:38.1   1st Qu.:172   1st Qu.:74.8  
 Median :2   Median : NA   Median :38.1   Median :172   Median :74.8  
 Mean   :2   Mean   :NaN   Mean   :38.1   Mean   :172   Mean   :74.8  
 3rd Qu.:2   3rd Qu.: NA   3rd Qu.:38.1   3rd Qu.:172   3rd Qu.:74.8  
 Max.   :2   Max.   : NA   Max.   :38.1   Max.   :172   Max.   :74.8  
             NA's   :1                                                
      BMI           BMI_cat1    BMI_cat2     waist      waist_cat1
 Min.   :25.28   Min.   :1   Min.   :2   Min.   :92   Min.   :0  
 1st Qu.:25.28   1st Qu.:1   1st Qu.:2   1st Qu.:92   1st Qu.:0  
 Median :25.28   Median :1   Median :2   Median :92   Median :0  
 Mean   :25.28   Mean   :1   Mean   :2   Mean   :92   Mean   :0  
 3rd Qu.:25.28   3rd Qu.:1   3rd Qu.:2   3rd Qu.:92   3rd Qu.:0  
 Max.   :25.28   Max.   :1   Max.   :2   Max.   :92   Max.   :0  
```

```
      hip             WHR               bmpsc              SBP               DBP     
 Min.   :104   Min.   :0.8846   Min.   :21.9   Min.   :140.5   Min.   :74  
 1st Qu.:104   1st Qu.:0.8846   1st Qu.:21.9   1st Qu.:140.5   1st Qu.:74  
 Median :104   Median :0.8846   Median :21.9   Median :140.5   Median :74  
 Mean   :104   Mean   :0.8846   Mean   :21.9   Mean   :140.5   Mean   :74  
 3rd Qu.:104   3rd Qu.:0.8846   3rd Qu.:21.9   3rd Qu.:140.5   3rd Qu.:74  
 Max.   :104   Max.   :0.8846   Max.   :21.9   Max.   :140.5   Max.   :74  
                                                                           
     HRTRTE           chol          hdlch          ldlch           trig    
 Min.   :57.5   Min.   :5.1   Min.   :2.6   Min.   :2.2   Min.   :0.7  
 1st Qu.:57.5   1st Qu.:5.1   1st Qu.:2.6   1st Qu.:2.2   1st Qu.:0.7  
 Median :57.5   Median :5.1   Median :2.6   Median :2.2   Median :0.7  
 Mean   :57.5   Mean   :5.1   Mean   :2.6   Mean   :2.2   Mean   :0.7  
 3rd Qu.:57.5   3rd Qu.:5.1   3rd Qu.:2.6   3rd Qu.:2.2   3rd Qu.:0.7  
 Max.   :57.5   Max.   :5.1   Max.   :2.6   Max.   :2.2   Max.   :0.7  
                                                                       
      apob            gluc         insulin          crpu           tnfa     
 Min.   :108.7   Min.   :4.4   Min.   : NA   Min.   :0.7   Min.   :3.79  
 1st Qu.:108.7   1st Qu.:4.4   1st Qu.: NA   1st Qu.:0.7   1st Qu.:3.79  
 Median :108.7   Median :4.4   Median : NA   Median :0.7   Median :3.79  
 Mean   :108.7   Mean   :4.4   Mean   :NaN   Mean   :0.7   Mean   :3.79  
 3rd Qu.:108.7   3rd Qu.:4.4   3rd Qu.: NA   3rd Qu.:0.7   3rd Qu.:3.79  
 Max.   :108.7   Max.   :4.4   Max.   : NA   Max.   :0.7   Max.   :3.79  
                               NA's   :1                                
   F1datquest              F1age          F1mrtsts      F1sclhlp  F1job_curr1
 Min.   :2011-05-05   Min.   :69.8   Min.   : NA   Min.   :1   Min.   :0   
 1st Qu.:2011-05-05   1st Qu.:69.8   1st Qu.: NA   1st Qu.:1   1st Qu.:0   
 Median :2011-05-05   Median :69.8   Median : NA   Median :1   Median :0   
 Mean   :2011-05-05   Mean   :69.8   Mean   :NaN   Mean   :1   Mean   :0   
 3rd Qu.:2011-05-05   3rd Qu.:69.8   3rd Qu.: NA   3rd Qu.:1   3rd Qu.:0   
 Max.   :2011-05-05   Max.   :69.8   Max.   : NA   Max.   :1   Max.   :0   
                                     NA's   :1                             
  F1job_curr4b occ_b     F1cmp       F1hdv        F1chf        F1artm   
 Min.   :3     1:0   Min.   :0   Min.   :0   Min.   :0   Min.   :1  
 1st Qu.:3     2:0   1st Qu.:0   1st Qu.:0   1st Qu.:0   1st Qu.:1  
 Median :3     3:0   Median :0   Median :0   Median :0   Median :1  
 Mean   :3     9:1   Mean   :0   Mean   :0   Mean   :0   Mean   :1  
 3rd Qu.:3           3rd Qu.:0   3rd Qu.:0   3rd Qu.:0   3rd Qu.:1  
 Max.   :3           Max.   :0   Max.   :0   Max.   :0   Max.   :1  
                                                                    
     F1cad       F1angn       F1miac       F1strk       F1vslg       F1ccth  
 Min.   :0   Min.   :0   Min.   :0   Min.   :0   Min.   :0   Min.   :0  
 1st Qu.:0   1st Qu.:0   1st Qu.:0   1st Qu.:0   1st Qu.:0   1st Qu.:0  
 Median :0   Median :0   Median :0   Median :0   Median :0   Median :0  
 Mean   :0   Mean   :0   Mean   :0   Mean   :0   Mean   :0   Mean   :0  
 3rd Qu.:0   3rd Qu.:0   3rd Qu.:0   3rd Qu.:0   3rd Qu.:0   3rd Qu.:0  
 Max.   :0   Max.   :0   Max.   :0   Max.   :0   Max.   :0   Max.   :0  
                                                                        
     F1cabg    F1prdbs_f  F1prdbsage_f   F1prhcl_f    F1prhclage_f  F1prhyptn_f
 Min.   :0   Min.   :0   Min.   : NA   Min.   : NA   Min.   : NA   Min.   :0   
 1st Qu.:0   1st Qu.:0   1st Qu.: NA   1st Qu.: NA   1st Qu.: NA   1st Qu.:0   
 Median :0   Median :0   Median : NA   Median : NA   Median : NA   Median :0   
 Mean   :0   Mean   :0   Mean   :NaN   Mean   :NaN   Mean   :NaN   Mean   :0   
 3rd Qu.:0   3rd Qu.:0   3rd Qu.: NA   3rd Qu.: NA   3rd Qu.: NA   3rd Qu.:0   
 Max.   :0   Max.   :0   Max.   : NA   Max.   : NA   Max.   : NA   Max.   :0   
                         NA's   :1     NA's   :1     NA's   :1                 
  F1prhypage_f  F1prhrtat_f  F1prtaage_f    F1prstrk_f  F1prstkage_f
 Min.   : NA   Min.   :0    Min.   : NA   Min.   :0    Min.   : NA  
 1st Qu.: NA   1st Qu.:0    1st Qu.: NA   1st Qu.:0    1st Qu.: NA  
 Median : NA   Median :0    Median : NA   Median :0    Median : NA  
 Mean   :NaN   Mean   :0    Mean   :NaN   Mean   :0    Mean   :NaN  
 3rd Qu.: NA   3rd Qu.:0    3rd Qu.: NA   3rd Qu.:0    3rd Qu.: NA  
 Max.   : NA   Max.   :0    Max.   : NA   Max.   :0    Max.   : NA  
 NA's   :1                  NA's   :1                  NA's   :1    
```

```
   F1prdbs_m  F1prdbsage_m   F1prhcl_m    F1prhclage_m  F1prhyptn_m
 Min.   :0   Min.   : NA   Min.   : NA   Min.   : NA   Min.   :1  
 1st Qu.:0   1st Qu.: NA   1st Qu.: NA   1st Qu.: NA   1st Qu.:1  
 Median :0   Median : NA   Median : NA   Median : NA   Median :1  
 Mean   :0   Mean   :NaN   Mean   :NaN   Mean   :NaN   Mean   :1  
 3rd Qu.:0   3rd Qu.: NA   3rd Qu.: NA   3rd Qu.: NA   3rd Qu.:1  
 Max.   :0   Max.   : NA   Max.   : NA   Max.   : NA   Max.   :1  
             NA's   :1     NA's   :1     NA's   :1                
  F1prhypage_m  F1prhrtat_m  F1prtaage_m    F1prstrk_m  F1prstkage_m
 Min.   : NA   Min.   :0    Min.   : NA   Min.   :0    Min.   : NA  
 1st Qu.: NA   1st Qu.:0    1st Qu.: NA   1st Qu.:0    1st Qu.: NA  
 Median : NA   Median :0    Median : NA   Median :0    Median : NA  
 Mean   :NaN   Mean   :0    Mean   :NaN   Mean   :0    Mean   :NaN  
 3rd Qu.: NA   3rd Qu.:0    3rd Qu.: NA   3rd Qu.:0    3rd Qu.: NA  
 Max.   : NA   Max.   :0    Max.   : NA   Max.   :0    Max.   : NA  
 NA's   :1                  NA's   :1                  NA's   :1    
   F1famno_db  F1famno_hcl  F1famno_htn  F1famno_mai  F1famno_str   F1alcool1
 Min.   :1    Min.   :9    Min.   :1    Min.   :0    Min.   :0    Min.   :3  
 1st Qu.:1    1st Qu.:9    1st Qu.:1    1st Qu.:0    1st Qu.:0    1st Qu.:3  
 Median :1    Median :9    Median :1    Median :0    Median :0    Median :3  
 Mean   :1    Mean   :9    Mean   :1    Mean   :0    Mean   :0    Mean   :3  
 3rd Qu.:1    3rd Qu.:9    3rd Qu.:1    3rd Qu.:0    3rd Qu.:0    3rd Qu.:3  
 Max.   :1    Max.   :9    Max.   :1    Max.   :0    Max.   :0    Max.   :3  

 sm_b    F1antiHTA     F1HTA       diab_b     F1etsem       F1PSQI_score
 0:0   Min.   :0   Min.   :1   Min.   :0   Min.   :18768   Min.   :5   
 1:0   1st Qu.:0   1st Qu.:1   1st Qu.:0   1st Qu.:18768   1st Qu.:5   
 2:1   Median :0   Median :1   Median :0   Median :18768   Median :5   
       Mean   :0   Mean   :1   Mean   :0   Mean   :18768   Mean   :5   
       3rd Qu.:0   3rd Qu.:1   3rd Qu.:0   3rd Qu.:18768   3rd Qu.:5   
       Max.   :0   Max.   :1   Max.   :0   Max.   :18768   Max.   :5   

     MMSE_b       F1CESD      depre_b    F1handgrip         F1ht    
 Min.   :29   Min.   :14   Min.   :0   Min.   :31.75   Min.   :172  
 1st Qu.:29   1st Qu.:14   1st Qu.:0   1st Qu.:31.75   1st Qu.:172  
 Median :29   Median :14   Median :0   Median :31.75   Median :172  
 Mean   :29   Mean   :14   Mean   :0   Mean   :31.75   Mean   :172  
 3rd Qu.:29   3rd Qu.:14   3rd Qu.:0   3rd Qu.:31.75   3rd Qu.:172  
 Max.   :29   Max.   :14   Max.   :0   Max.   :31.75   Max.   :172  

      F1wt          bmi_b         F1BMI_cat1   F1BMI_cat2    F1waist    
 Min.   :81.4   Min.   :27.51   Min.   :1    Min.   :2    Min.   :98.5  
 1st Qu.:81.4   1st Qu.:27.51   1st Qu.:1    1st Qu.:2    1st Qu.:98.5  
 Median :81.4   Median :27.51   Median :1    Median :2    Median :98.5  
 Mean   :81.4   Mean   :27.51   Mean   :1    Mean   :2    Mean   :98.5  
 3rd Qu.:81.4   3rd Qu.:27.51   3rd Qu.:1    3rd Qu.:2    3rd Qu.:98.5  
 Max.   :81.4   Max.   :27.51   Max.   :1    Max.   :2    Max.   :98.5  

  F1waist_cat1     F1hip            F1WHR            F1bmpsc        F1BIAcom  
 Min.   :0     Min.   :100.5   Min.   :0.9801   Min.   :27.3   Min.   : NA  
 1st Qu.:0     1st Qu.:100.5   1st Qu.:0.9801   1st Qu.:27.3   1st Qu.: NA  
 Median :0     Median :100.5   Median :0.9801   Median :27.3   Median : NA  
 Mean   :0     Mean   :100.5   Mean   :0.9801   Mean   :27.3   Mean   :NaN  
 3rd Qu.:0     3rd Qu.:100.5   3rd Qu.:0.9801   3rd Qu.:27.3   3rd Qu.: NA  
 Max.   :0     Max.   :100.5   Max.   :0.9801   Max.   :27.3   Max.   : NA  
                                                               NA's   :1    
     F1SBP         F1DBP          F1HRTRTE      F1chol      F1hdlch   
 Min.   :160   Min.   :89.5   Min.   :57   Min.   :5   Min.   :2.1  
 1st Qu.:160   1st Qu.:89.5   1st Qu.:57   1st Qu.:5   1st Qu.:2.1  
 Median :160   Median :89.5   Median :57   Median :5   Median :2.1  
 Mean   :160   Mean   :89.5   Mean   :57   Mean   :5   Mean   :2.1  
 3rd Qu.:160   3rd Qu.:89.5   3rd Qu.:57   3rd Qu.:5   3rd Qu.:2.1  
 Max.   :160   Max.   :89.5   Max.   :57   Max.   :5   Max.   :2.1  
```

```
   F1ldlch         F1trig          F1gluc        F1insulin        F1crpu     
 Min.   :2.5   Min.   :0.9   Min.   :6.5   Min.   :6.6   Min.   :1.1  
 1st Qu.:2.5   1st Qu.:0.9   1st Qu.:6.5   1st Qu.:6.6   1st Qu.:1.1  
 Median :2.5   Median :0.9   Median :6.5   Median :6.6   Median :1.1  
 Mean   :2.5   Mean   :0.9   Mean   :6.5   Mean   :6.6   Mean   :1.1  
 3rd Qu.:2.5   3rd Qu.:0.9   3rd Qu.:6.5   3rd Qu.:6.6   3rd Qu.:1.1  
 Max.   :2.5   Max.   :0.9   Max.   :6.5   Max.   :6.6   Max.   :1.1  

     F1il6          F1tnfa        F1freqFFQ1   F1freqFFQ2    F1freqFFQ3 
 Min.   :0.29   Min.   :1.35   Min.   :0    Min.   :2.5   Min.   :2.5  
 1st Qu.:0.29   1st Qu.:1.35   1st Qu.:0    1st Qu.:2.5   1st Qu.:2.5  
 Median :0.29   Median :1.35   Median :0    Median :2.5   Median :2.5  
 Mean   :0.29   Mean   :1.35   Mean   :0    Mean   :2.5   Mean   :2.5  
 3rd Qu.:0.29   3rd Qu.:1.35   3rd Qu.:0    3rd Qu.:2.5   3rd Qu.:2.5  
 Max.   :0.29   Max.   :1.35   Max.   :0    Max.   :2.5   Max.   :2.5  

   F1freqFFQ4        F1freqFFQ5   F1freqFFQ6   F1freqFFQ7    F1freqFFQ8
 Min.   :0.03571   Min.   :0    Min.   :0    Min.   :2.5   Min.   :0   
 1st Qu.:0.03571   1st Qu.:0    1st Qu.:0    1st Qu.:2.5   1st Qu.:0   
 Median :0.03571   Median :0    Median :0    Median :2.5   Median :0   
 Mean   :0.03571   Mean   :0    Mean   :0    Mean   :2.5   Mean   :0   
 3rd Qu.:0.03571   3rd Qu.:0    3rd Qu.:0    3rd Qu.:2.5   3rd Qu.:0   
 Max.   :0.03571   Max.   :0    Max.   :0    Max.   :2.5   Max.   :0   

   F1freqFFQ9  F1freqFFQ10   F1freqFFQ11      F1freqFFQ12  F1freqFFQ13
 Min.   :0    Min.   :2.5   Min.   :0.2143   Min.   :0    Min.   :0   
 1st Qu.:0    1st Qu.:2.5   1st Qu.:0.2143   1st Qu.:0    1st Qu.:0   
 Median :0    Median :2.5   Median :0.2143   Median :0    Median :0   
 Mean   :0    Mean   :2.5   Mean   :0.2143   Mean   :0    Mean   :0   
 3rd Qu.:0    3rd Qu.:2.5   3rd Qu.:0.2143   3rd Qu.:0    3rd Qu.:0   
 Max.   :0    Max.   :2.5   Max.   :0.2143   Max.   :0    Max.   :0   

  F1freqFFQ14  F1freqFFQ15   F1freqFFQ16  F1freqFFQ17  F1freqFFQ18  F1freqFFQ19
 Min.   :0    Min.   :0.5   Min.   :0    Min.   :0    Min.   :0    Min.   :0   
 1st Qu.:0    1st Qu.:0.5   1st Qu.:0    1st Qu.:0    1st Qu.:0    1st Qu.:0   
 Median :0    Median :0.5   Median :0    Median :0    Median :0    Median :0   
 Mean   :0    Mean   :0.5   Mean   :0    Mean   :0    Mean   :0    Mean   :0   
 3rd Qu.:0    3rd Qu.:0.5   3rd Qu.:0    3rd Qu.:0    3rd Qu.:0    3rd Qu.:0   
 Max.   :0    Max.   :0.5   Max.   :0    Max.   :0    Max.   :0    Max.   :0   

  F1freqFFQ20  F1freqFFQ21  F1freqFFQ22  F1freqFFQ23  F1freqFFQ24  F1freqFFQ25
 Min.   :0    Min.   :0    Min.   :0    Min.   :0    Min.   :0    Min.   :0   
 1st Qu.:0    1st Qu.:0    1st Qu.:0    1st Qu.:0    1st Qu.:0    1st Qu.:0   
 Median :0    Median :0    Median :0    Median :0    Median :0    Median :0   
 Mean   :0    Mean   :0    Mean   :0    Mean   :0    Mean   :0    Mean   :0   
 3rd Qu.:0    3rd Qu.:0    3rd Qu.:0    3rd Qu.:0    3rd Qu.:0    3rd Qu.:0   
 Max.   :0    Max.   :0    Max.   :0    Max.   :0    Max.   :0    Max.   :0   

  F1freqFFQ26  F1freqFFQ27      F1freqFFQ28  F1freqFFQ29  F1freqFFQ30    
 Min.   :0    Min.   :0.2143   Min.   :0    Min.   :0    Min.   :0.2143  
 1st Qu.:0    1st Qu.:0.2143   1st Qu.:0    1st Qu.:0    1st Qu.:0.2143  
 Median :0    Median :0.2143   Median :0    Median :0    Median :0.2143  
 Mean   :0    Mean   :0.2143   Mean   :0    Mean   :0    Mean   :0.2143  
 3rd Qu.:0    3rd Qu.:0.2143   3rd Qu.:0    3rd Qu.:0    3rd Qu.:0.2143  
 Max.   :0    Max.   :0.2143   Max.   :0    Max.   :0    Max.   :0.2143  

  F1freqFFQ31      F1freqFFQ32   F1freqFFQ33   F1freqFFQ34   F1freqFFQ35 
 Min.   :0.2143   Min.   :0.5   Min.   :0.5   Min.   :0.5   Min.   :0.5  
 1st Qu.:0.2143   1st Qu.:0.5   1st Qu.:0.5   1st Qu.:0.5   1st Qu.:0.5  
 Median :0.2143   Median :0.5   Median :0.5   Median :0.5   Median :0.5  
 Mean   :0.2143   Mean   :0.5   Mean   :0.5   Mean   :0.5   Mean   :0.5  
 3rd Qu.:0.2143   3rd Qu.:0.5   3rd Qu.:0.5   3rd Qu.:0.5   3rd Qu.:0.5  
 Max.   :0.2143   Max.   :0.5   Max.   :0.5   Max.   :0.5   Max.   :0.5  
```

```
  F1freqFFQ36      F1freqFFQ37      F1freqFFQ38  F1freqFFQ39  F1freqFFQ40   
 Min.   :0.2143   Min.   :0.2143   Min.   :0    Min.   :0    Min.   :0.2143  
 1st Qu.:0.2143   1st Qu.:0.2143   1st Qu.:0    1st Qu.:0    1st Qu.:0.2143  
 Median :0.2143   Median :0.2143   Median :0    Median :0    Median :0.2143  
 Mean   :0.2143   Mean   :0.2143   Mean   :0    Mean   :0    Mean   :0.2143  
 3rd Qu.:0.2143   3rd Qu.:0.2143   3rd Qu.:0    3rd Qu.:0    3rd Qu.:0.2143  
 Max.   :0.2143   Max.   :0.2143   Max.   :0    Max.   :0    Max.   :0.2143  

  F1freqFFQ41  F1freqFFQ42   F1freqFFQ43  F1freqFFQ44  F1freqFFQ45   
 Min.   :0    Min.   :0.5   Min.   :0    Min.   :1    Min.   :0.2143  
 1st Qu.:0    1st Qu.:0.5   1st Qu.:0    1st Qu.:1    1st Qu.:0.2143  
 Median :0    Median :0.5   Median :0    Median :1    Median :0.2143  
 Mean   :0    Mean   :0.5   Mean   :0    Mean   :1    Mean   :0.2143  
 3rd Qu.:0    3rd Qu.:0.5   3rd Qu.:0    3rd Qu.:1    3rd Qu.:0.2143  
 Max.   :0    Max.   :0.5   Max.   :0    Max.   :1    Max.   :0.2143  

  F1freqFFQ46  F1freqFFQ47       F1freqFFQ48  F1freqFFQ49      F1freqFFQ50
 Min.   :0    Min.   :0.03571   Min.   :0    Min.   :0.2143   Min.   :0   
 1st Qu.:0    1st Qu.:0.03571   1st Qu.:0    1st Qu.:0.2143   1st Qu.:0   
 Median :0    Median :0.03571   Median :0    Median :0.2143   Median :0   
 Mean   :0    Mean   :0.03571   Mean   :0    Mean   :0.2143   Mean   :0   
 3rd Qu.:0    3rd Qu.:0.03571   3rd Qu.:0    3rd Qu.:0.2143   3rd Qu.:0   
 Max.   :0    Max.   :0.03571   Max.   :0    Max.   :0.2143   Max.   :0   

  F1freqFFQ51  F1freqFFQ52  F1freqFFQ53  F1freqFFQ54  F1freqFFQ55  F1freqFFQ56
 Min.   :0    Min.   :1    Min.   :0    Min.   :0    Min.   :1    Min.   :1   
 1st Qu.:0    1st Qu.:1    1st Qu.:0    1st Qu.:0    1st Qu.:1    1st Qu.:1   
 Median :0    Median :1    Median :0    Median :0    Median :1    Median :1   
 Mean   :0    Mean   :1    Mean   :0    Mean   :0    Mean   :1    Mean   :1   
 3rd Qu.:0    3rd Qu.:1    3rd Qu.:0    3rd Qu.:0    3rd Qu.:1    3rd Qu.:1   
 Max.   :0    Max.   :1    Max.   :0    Max.   :0    Max.   :1    Max.   :1   

  F1freqFFQ57       F1freqFFQ58      F1freqFFQ59       F1freqFFQ60  F1freqFFQ61
 Min.   :0.03571   Min.   :0.2143   Min.   :0.08929   Min.   :0    Min.   :0   
 1st Qu.:0.03571   1st Qu.:0.2143   1st Qu.:0.08929   1st Qu.:0    1st Qu.:0   
 Median :0.03571   Median :0.2143   Median :0.08929   Median :0    Median :0   
 Mean   :0.03571   Mean   :0.2143   Mean   :0.08929   Mean   :0    Mean   :0   
 3rd Qu.:0.03571   3rd Qu.:0.2143   3rd Qu.:0.08929   3rd Qu.:0    3rd Qu.:0   
 Max.   :0.03571   Max.   :0.2143   Max.   :0.08929   Max.   :0    Max.   :0   

  F1freqFFQ62  F1freqFFQ63  F1freqFFQ64  F1freqFFQ65  F1freqFFQ66   F1freqFFQ67
 Min.   :0    Min.   :0    Min.   :0    Min.   :0    Min.   :0.5   Min.   :1   
 1st Qu.:0    1st Qu.:0    1st Qu.:0    1st Qu.:0    1st Qu.:0.5   1st Qu.:1   
 Median :0    Median :0    Median :0    Median :0    Median :0.5   Median :1   
 Mean   :0    Mean   :0    Mean   :0    Mean   :0    Mean   :0.5   Mean   :1   
 3rd Qu.:0    3rd Qu.:0    3rd Qu.:0    3rd Qu.:0    3rd Qu.:0.5   3rd Qu.:1   
 Max.   :0    Max.   :0    Max.   :0    Max.   :0    Max.   :0.5   Max.   :1   

  F1freqFFQ68       F1freqFFQ69  F1freqFFQ70  F1freqFFQ71  F1freqFFQ72
 Min.   :0.03571   Min.   :0    Min.   :0    Min.   :0    Min.   :0   
 1st Qu.:0.03571   1st Qu.:0    1st Qu.:0    1st Qu.:0    1st Qu.:0   
 Median :0.03571   Median :0    Median :0    Median :0    Median :0   
 Mean   :0.03571   Mean   :0    Mean   :0    Mean   :0    Mean   :0   
 3rd Qu.:0.03571   3rd Qu.:0    3rd Qu.:0    3rd Qu.:0    3rd Qu.:0   
 Max.   :0.03571   Max.   :0    Max.   :0    Max.   :0    Max.   :0   

  F1freqFFQ73      F1freqFFQ74  F1freqFFQ75  F1freqFFQ76  F1freqFFQ77
 Min.   :0.2143   Min.   :0    Min.   :0    Min.   :0    Min.   :0   
 1st Qu.:0.2143   1st Qu.:0    1st Qu.:0    1st Qu.:0    1st Qu.:0   
 Median :0.2143   Median :0    Median :0    Median :0    Median :0   
 Mean   :0.2143   Mean   :0    Mean   :0    Mean   :0    Mean   :0   
 3rd Qu.:0.2143   3rd Qu.:0    3rd Qu.:0    3rd Qu.:0    3rd Qu.:0   
 Max.   :0.2143   Max.   :0    Max.   :0    Max.   :0    Max.   :0   
```

```
  F1freqFFQ78  F1freqFFQ79  F1freqFFQ80  F1freqFFQ81       F1freqFFQ82
 Min.   :0    Min.   :0    Min.   :0    Min.   :0.08929   Min.   :0   
 1st Qu.:0    1st Qu.:0    1st Qu.:0    1st Qu.:0.08929   1st Qu.:0   
 Median :0    Median :0    Median :0    Median :0.08929   Median :0   
 Mean   :0    Mean   :0    Mean   :0    Mean   :0.08929   Mean   :0   
 3rd Qu.:0    3rd Qu.:0    3rd Qu.:0    3rd Qu.:0.08929   3rd Qu.:0   
 Max.   :0    Max.   :0    Max.   :0    Max.   :0.08929   Max.   :0   

  F1freqFFQ83  F1freqFFQ84  F1freqFFQ85      F1freqFFQ86  F1freqFFQ87
 Min.   :0    Min.   :0    Min.   :0.2143   Min.   :0    Min.   :0   
 1st Qu.:0    1st Qu.:0    1st Qu.:0.2143   1st Qu.:0    1st Qu.:0   
 Median :0    Median :0    Median :0.2143   Median :0    Median :0   
 Mean   :0    Mean   :0    Mean   :0.2143   Mean   :0    Mean   :0   
 3rd Qu.:0    3rd Qu.:0    3rd Qu.:0.2143   3rd Qu.:0    3rd Qu.:0   
 Max.   :0    Max.   :0    Max.   :0.2143   Max.   :0    Max.   :0   

  F1freqFFQ88  F1freqFFQ89   F1freqFFQ90  F1freqFFQ91   F1freqFFQ92
 Min.   :0    Min.   :2.5   Min.   :0    Min.   :0.5   Min.   :0   
 1st Qu.:0    1st Qu.:2.5   1st Qu.:0    1st Qu.:0.5   1st Qu.:0   
 Median :0    Median :2.5   Median :0    Median :0.5   Median :0   
 Mean   :0    Mean   :2.5   Mean   :0    Mean   :0.5   Mean   :0   
 3rd Qu.:0    3rd Qu.:2.5   3rd Qu.:0    3rd Qu.:0.5   3rd Qu.:0   
 Max.   :0    Max.   :2.5   Max.   :0    Max.   :0.5   Max.   :0   

  F1freqFFQ93   F1freqFFQ94  F1freqFFQ95  F1freqFFQ96  F1freqFFQ97
 Min.   :2.5   Min.   :0    Min.   :1    Min.   :0    Min.   :0   
 1st Qu.:2.5   1st Qu.:0    1st Qu.:1    1st Qu.:0    1st Qu.:0   
 Median :2.5   Median :0    Median :1    Median :0    Median :0   
 Mean   :2.5   Mean   :0    Mean   :1    Mean   :0    Mean   :0   
 3rd Qu.:2.5   3rd Qu.:0    3rd Qu.:1    3rd Qu.:0    3rd Qu.:0   
 Max.   :2.5   Max.   :0    Max.   :1    Max.   :0    Max.   :0   

  F1FFQ1amount  F1FFQ2amount  F1FFQ3amount  F1FFQ4amount    F1FFQ5amount
 Min.   :0     Min.   :675   Min.   :675   Min.   :1.786   Min.   :0    
 1st Qu.:0     1st Qu.:675   1st Qu.:675   1st Qu.:1.786   1st Qu.:0    
 Median :0     Median :675   Median :675   Median :1.786   Median :0    
 Mean   :0     Mean   :675   Mean   :675   Mean   :1.786   Mean   :0    
 3rd Qu.:0     3rd Qu.:675   3rd Qu.:675   3rd Qu.:1.786   3rd Qu.:0    
 Max.   :0     Max.   :675   Max.   :675   Max.   :1.786   Max.   :0    

  F1FFQ6amount  F1FFQ7amount  F1FFQ8amount  F1FFQ9amount F1FFQ10amount
 Min.   :0     Min.   :300   Min.   :0     Min.   :0     Min.   :225  
 1st Qu.:0     1st Qu.:300   1st Qu.:0     1st Qu.:0     1st Qu.:225  
 Median :0     Median :300   Median :0     Median :0     Median :225  
 Mean   :0     Mean   :300   Mean   :0     Mean   :0     Mean   :225  
 3rd Qu.:0     3rd Qu.:300   3rd Qu.:0     3rd Qu.:0     3rd Qu.:225  
 Max.   :0     Max.   :300   Max.   :0     Max.   :0     Max.   :225  

 F1FFQ11amount   F1FFQ12amount F1FFQ13amount F1FFQ14amount F1FFQ15amount
 Min.   :10.71   Min.   :0     Min.   :0     Min.   :0     Min.   :75   
 1st Qu.:10.71   1st Qu.:0     1st Qu.:0     1st Qu.:0     1st Qu.:75   
 Median :10.71   Median :0     Median :0     Median :0     Median :75   
 Mean   :10.71   Mean   :0     Mean   :0     Mean   :0     Mean   :75   
 3rd Qu.:10.71   3rd Qu.:0     3rd Qu.:0     3rd Qu.:0     3rd Qu.:75   
 Max.   :10.71   Max.   :0     Max.   :0     Max.   :0     Max.   :75   

 F1FFQ16amount F1FFQ17amount F1FFQ18amount F1FFQ19amount F1FFQ20amount
 Min.   :0     Min.   :0     Min.   :0     Min.   :0     Min.   :0    
 1st Qu.:0     1st Qu.:0     1st Qu.:0     1st Qu.:0     1st Qu.:0    
 Median :0     Median :0     Median :0     Median :0     Median :0    
 Mean   :0     Mean   :0     Mean   :0     Mean   :0     Mean   :0    
 3rd Qu.:0     3rd Qu.:0     3rd Qu.:0     3rd Qu.:0     3rd Qu.:0    
 Max.   :0     Max.   :0     Max.   :0     Max.   :0     Max.   :0    
```

```
 F1FFQ21amount F1FFQ22amount F1FFQ23amount F1FFQ24amount F1FFQ25amount
 Min.   :0     Min.   :0     Min.   :0     Min.   :0     Min.   :0    
 1st Qu.:0     1st Qu.:0     1st Qu.:0     1st Qu.:0     1st Qu.:0    
 Median :0     Median :0     Median :0     Median :0     Median :0    
 Mean   :0     Mean   :0     Mean   :0     Mean   :0     Mean   :0    
 3rd Qu.:0     3rd Qu.:0     3rd Qu.:0     3rd Qu.:0     3rd Qu.:0    
 Max.   :0     Max.   :0     Max.   :0     Max.   :0     Max.   :0    

 F1FFQ26amount F1FFQ27amount   F1FFQ28amount F1FFQ29amount F1FFQ30amount
 Min.   :0     Min.   :25.71   Min.   :0     Min.   :0     Min.   :32.14
 1st Qu.:0     1st Qu.:25.71   1st Qu.:0     1st Qu.:0     1st Qu.:32.14
 Median :0     Median :25.71   Median :0     Median :0     Median :32.14
 Mean   :0     Mean   :25.71   Mean   :0     Mean   :0     Mean   :32.14
 3rd Qu.:0     3rd Qu.:25.71   3rd Qu.:0     3rd Qu.:0     3rd Qu.:32.14
 Max.   :0     Max.   :25.71   Max.   :0     Max.   :0     Max.   :32.14

 F1FFQ31amount   F1FFQ32amount F1FFQ33amount  F1FFQ34amount F1FFQ35amount
 Min.   :42.86   Min.   :45    Min.   :37.5   Min.   :25    Min.   :7.5  
 1st Qu.:42.86   1st Qu.:45    1st Qu.:37.5   1st Qu.:25    1st Qu.:7.5  
 Median :42.86   Median :45    Median :37.5   Median :25    Median :7.5  
 Mean   :42.86   Mean   :45    Mean   :37.5   Mean   :25    Mean   :7.5  
 3rd Qu.:42.86   3rd Qu.:45    3rd Qu.:37.5   3rd Qu.:25    3rd Qu.:7.5  
 Max.   :42.86   Max.   :45    Max.   :37.5   Max.   :25    Max.   :7.5  

 F1FFQ36amount   F1FFQ37amount   F1FFQ38amount F1FFQ39amount F1FFQ40amount
 Min.   :12.86   Min.   :38.57   Min.   :0     Min.   :0     Min.   :32.14
 1st Qu.:12.86   1st Qu.:38.57   1st Qu.:0     1st Qu.:0     1st Qu.:32.14
 Median :12.86   Median :38.57   Median :0     Median :0     Median :32.14
 Mean   :12.86   Mean   :38.57   Mean   :0     Mean   :0     Mean   :32.14
 3rd Qu.:12.86   3rd Qu.:38.57   3rd Qu.:0     3rd Qu.:0     3rd Qu.:32.14
 Max.   :12.86   Max.   :38.57   Max.   :0     Max.   :0     Max.   :32.14

 F1FFQ41amount F1FFQ42amount F1FFQ43amount F1FFQ44amount F1FFQ45amount
 Min.   :0     Min.   :150   Min.   :0     Min.   :60    Min.   :17.14
 1st Qu.:0     1st Qu.:150   1st Qu.:0     1st Qu.:60    1st Qu.:17.14
 Median :0     Median :150   Median :0     Median :60    Median :17.14
 Mean   :0     Mean   :150   Mean   :0     Mean   :60    Mean   :17.14
 3rd Qu.:0     3rd Qu.:150   3rd Qu.:0     3rd Qu.:60    3rd Qu.:17.14
 Max.   :0     Max.   :150   Max.   :0     Max.   :60    Max.   :17.14

 F1FFQ46amount F1FFQ47amount   F1FFQ48amount F1FFQ49amount   F1FFQ50amount
 Min.   :0     Min.   :8.929   Min.   :0     Min.   :21.43   Min.   :0    
 1st Qu.:0     1st Qu.:8.929   1st Qu.:0     1st Qu.:21.43   1st Qu.:0    
 Median :0     Median :8.929   Median :0     Median :21.43   Median :0    
 Mean   :0     Mean   :8.929   Mean   :0     Mean   :21.43   Mean   :0    
 3rd Qu.:0     3rd Qu.:8.929   3rd Qu.:0     3rd Qu.:21.43   3rd Qu.:0    
 Max.   :0     Max.   :8.929   Max.   :0     Max.   :21.43   Max.   :0    

 F1FFQ51amount F1FFQ52amount F1FFQ53amount F1FFQ54amount F1FFQ55amount
 Min.   :0     Min.   :10    Min.   :0     Min.   :0     Min.   :130  
 1st Qu.:0     1st Qu.:10    1st Qu.:0     1st Qu.:0     1st Qu.:130  
 Median :0     Median :10    Median :0     Median :0     Median :130  
 Mean   :0     Mean   :10    Mean   :0     Mean   :0     Mean   :130  
 3rd Qu.:0     3rd Qu.:10    3rd Qu.:0     3rd Qu.:0     3rd Qu.:130  
 Max.   :0     Max.   :10    Max.   :0     Max.   :0     Max.   :130  

 F1FFQ56amount F1FFQ57amount   F1FFQ58amount   F1FFQ59amount   F1FFQ60amount
 Min.   :150   Min.   :3.571   Min.   :32.14   Min.   :8.036   Min.   :0    
 1st Qu.:150   1st Qu.:3.571   1st Qu.:32.14   1st Qu.:8.036   1st Qu.:0    
 Median :150   Median :3.571   Median :32.14   Median :8.036   Median :0    
 Mean   :150   Mean   :3.571   Mean   :32.14   Mean   :8.036   Mean   :0    
 3rd Qu.:150   3rd Qu.:3.571   3rd Qu.:32.14   3rd Qu.:8.036   3rd Qu.:0    
 Max.   :150   Max.   :3.571   Max.   :32.14   Max.   :8.036   Max.   :0    
```

```
 F1FFQ61amount F1FFQ62amount F1FFQ63amount F1FFQ64amount F1FFQ65amount
 Min.   :0     Min.   :0     Min.   :0     Min.   :0     Min.   :0    
 1st Qu.:0     1st Qu.:0     1st Qu.:0     1st Qu.:0     1st Qu.:0    
 Median :0     Median :0     Median :0     Median :0     Median :0    
 Mean   :0     Mean   :0     Mean   :0     Mean   :0     Mean   :0    
 3rd Qu.:0     3rd Qu.:0     3rd Qu.:0     3rd Qu.:0     3rd Qu.:0    
 Max.   :0     Max.   :0     Max.   :0     Max.   :0     Max.   :0    

 F1FFQ66amount F1FFQ67amount F1FFQ68amount   F1FFQ70amount F1FFQ71amount
 Min.   :15    Min.   :20    Min.   :4.286   Min.   :0     Min.   :0    
 1st Qu.:15    1st Qu.:20    1st Qu.:4.286   1st Qu.:0     1st Qu.:0    
 Median :15    Median :20    Median :4.286   Median :0     Median :0    
 Mean   :15    Mean   :20    Mean   :4.286   Mean   :0     Mean   :0    
 3rd Qu.:15    3rd Qu.:20    3rd Qu.:4.286   3rd Qu.:0     3rd Qu.:0    
 Max.   :15    Max.   :20    Max.   :4.286   Max.   :0     Max.   :0    

 F1FFQ72amount F1FFQ73amount   F1FFQ74amount F1FFQ75amount F1FFQ81amount
 Min.   :0     Min.   :1.071   Min.   :0     Min.   :0     Min.   :10.71
 1st Qu.:0     1st Qu.:1.071   1st Qu.:0     1st Qu.:0     1st Qu.:10.71
 Median :0     Median :1.071   Median :0     Median :0     Median :10.71
 Mean   :0     Mean   :1.071   Mean   :0     Mean   :0     Mean   :10.71
 3rd Qu.:0     3rd Qu.:1.071   3rd Qu.:0     3rd Qu.:0     3rd Qu.:10.71
 Max.   :0     Max.   :1.071   Max.   :0     Max.   :0     Max.   :10.71

 F1FFQ82amount F1FFQ83amount F1FFQ84amount F1FFQ85amount   F1FFQ86amount
 Min.   :0     Min.   :0     Min.   :0     Min.   :32.14   Min.   :0    
 1st Qu.:0     1st Qu.:0     1st Qu.:0     1st Qu.:32.14   1st Qu.:0    
 Median :0     Median :0     Median :0     Median :32.14   Median :0    
 Mean   :0     Mean   :0     Mean   :0     Mean   :32.14   Mean   :0    
 3rd Qu.:0     3rd Qu.:0     3rd Qu.:0     3rd Qu.:32.14   3rd Qu.:0    
 Max.   :0     Max.   :0     Max.   :0     Max.   :32.14   Max.   :0    

 F1FFQ87amount F1FFQ88amount F1FFQ89amount F1FFQ90amount F1FFQ91amount
 Min.   :0     Min.   :0     Min.   :750   Min.   :0     Min.   :100  
 1st Qu.:0     1st Qu.:0     1st Qu.:750   1st Qu.:0     1st Qu.:100  
 Median :0     Median :0     Median :750   Median :0     Median :100  
 Mean   :0     Mean   :0     Mean   :750   Mean   :0     Mean   :100  
 3rd Qu.:0     3rd Qu.:0     3rd Qu.:750   3rd Qu.:0     3rd Qu.:100  
 Max.   :0     Max.   :0     Max.   :750   Max.   :0     Max.   :100  

 F1FFQ92amount F1FFQ93amount F1FFQ94amount F1FFQ95amount F1FFQ96amount
 Min.   :0     Min.   :750   Min.   :0     Min.   :225   Min.   :0    
 1st Qu.:0     1st Qu.:750   1st Qu.:0     1st Qu.:225   1st Qu.:0    
 Median :0     Median :750   Median :0     Median :225   Median :0    
 Mean   :0     Mean   :750   Mean   :0     Mean   :225   Mean   :0    
 3rd Qu.:0     3rd Qu.:750   3rd Qu.:0     3rd Qu.:225   3rd Qu.:0    
 Max.   :0     Max.   :750   Max.   :0     Max.   :225   Max.   :0    

 F1FFQ97amount   F1numitems   totalcal_b     F1sumtot2      F1sumtot3   
 Min.   :0     Min.   :38   Min.   :3669   Min.   :4075   Min.   :3527  
 1st Qu.:0     1st Qu.:38   1st Qu.:3669   1st Qu.:4075   1st Qu.:3527  
 Median :0     Median :38   Median :3669   Median :4075   Median :3527  
 Mean   :0     Mean   :38   Mean   :3669   Mean   :4075   Mean   :3527  
 3rd Qu.:0     3rd Qu.:38   3rd Qu.:3669   3rd Qu.:4075   3rd Qu.:3527  
 Max.   :0     Max.   :38   Max.   :3669   Max.   :4075   Max.   :3527  

   F1sumprot1      F1sumpveg1      F1sumpani1      F1sumgluc1     F1sumgsuc1   
 Min.   :797.5   Min.   :181.2   Min.   :616.3   Min.   :1927   Min.   :941.8  
 1st Qu.:797.5   1st Qu.:181.2   1st Qu.:616.3   1st Qu.:1927   1st Qu.:941.8  
 Median :797.5   Median :181.2   Median :616.3   Median :1927   Median :941.8  
 Mean   :797.5   Mean   :181.2   Mean   :616.3   Mean   :1927   Mean   :941.8  
 3rd Qu.:797.5   3rd Qu.:181.2   3rd Qu.:616.3   3rd Qu.:1927   3rd Qu.:941.8  
 Max.   :797.5   Max.   :181.2   Max.   :616.3   Max.   :1927   Max.   :941.8  
```

```
  F1sumgpol1       F1sumlipi1     F1sumlsat1       F1sumlmon1       F1sumlpol1    
 Min.   :985.4   Min.   :1350   Min.   :631.8   Min.   :505.4   Min.   :116.5  
 1st Qu.:985.4   1st Qu.:1350   1st Qu.:631.8   1st Qu.:505.4   1st Qu.:116.5  
 Median :985.4   Median :1350   Median :631.8   Median :505.4   Median :116.5  
 Mean   :985.4   Mean   :1350   Mean   :631.8   Mean   :505.4   Mean   :116.5  
 3rd Qu.:985.4   3rd Qu.:1350   3rd Qu.:631.8   3rd Qu.:505.4   3rd Qu.:116.5  
 Max.   :985.4   Max.   :1350   Max.   :631.8   Max.   :505.4   Max.   :116.5  

  F1sumfibr1     F1sumchol1      F1sumalco        F1sumcalc1     F1sumfer1   
 Min.   :32.9   Min.   :549.1   Min.   :138.6   Min.   :4662   Min.   :16.1  
 1st Qu.:32.9   1st Qu.:549.1   1st Qu.:138.6   1st Qu.:4662   1st Qu.:16.1  
 Median :32.9   Median :549.1   Median :138.6   Median :4662   Median :16.1  
 Mean   :32.9   Mean   :549.1   Mean   :138.6   Mean   :4662   Mean   :16.1  
 3rd Qu.:32.9   3rd Qu.:549.1   3rd Qu.:138.6   3rd Qu.:4662   3rd Qu.:16.1  
 Max.   :32.9   Max.   :549.1   Max.   :138.6   Max.   :4662   Max.   :16.1  

  F1sumret1        F1sumcaro1     F1sumvitd1    F1sumeau1      F1sumprot3   
 Min.   :857.1   Min.   :6391   Min.   :4.1   Min.   :10.5   Min.   :687.8  
 1st Qu.:857.1   1st Qu.:6391   1st Qu.:4.1   1st Qu.:10.5   1st Qu.:687.8  
 Median :857.1   Median :6391   Median :4.1   Median :10.5   Median :687.8  
 Mean   :857.1   Mean   :6391   Mean   :4.1   Mean   :10.5   Mean   :687.8  
 3rd Qu.:857.1   3rd Qu.:6391   3rd Qu.:4.1   3rd Qu.:10.5   3rd Qu.:687.8  
 Max.   :857.1   Max.   :6391   Max.   :4.1   Max.   :10.5   Max.   :687.8  

  F1sumpveg3    F1sumpani3      F1sumgluc3     F1sumgsuc3      F1sumgpol3   
 Min.   :147   Min.   :540.7   Min.   :1700   Min.   :921.2   Min.   :778.7  
 1st Qu.:147   1st Qu.:540.7   1st Qu.:1700   1st Qu.:921.2   1st Qu.:778.7  
 Median :147   Median :540.7   Median :1700   Median :921.2   Median :778.7  
 Mean   :147   Mean   :540.7   Mean   :1700   Mean   :921.2   Mean   :778.7  
 3rd Qu.:147   3rd Qu.:540.7   3rd Qu.:1700   3rd Qu.:921.2   3rd Qu.:778.7  
 Max.   :147   Max.   :540.7   Max.   :1700   Max.   :921.2   Max.   :778.7  

  F1sumlipi3     F1sumlsat3      F1sumlmon3       F1sumlpol3     F1sumcalc3  
 Min.   :1139   Min.   :512.4   Min.   :446.2   Min.   :95.6   Min.   :4646  
 1st Qu.:1139   1st Qu.:512.4   1st Qu.:446.2   1st Qu.:95.6   1st Qu.:4646  
 Median :1139   Median :512.4   Median :446.2   Median :95.6   Median :4646  
 Mean   :1139   Mean   :512.4   Mean   :446.2   Mean   :95.6   Mean   :4646  
 3rd Qu.:1139   3rd Qu.:512.4   3rd Qu.:446.2   3rd Qu.:95.6   3rd Qu.:4646  
 Max.   :1139   Max.   :512.4   Max.   :446.2   Max.   :95.6   Max.   :4646  

  F1sumfer3      F1sumret3        F1sumcaro3     F1sumvitd3    F1sumfibr3   
 Min.   :14.1   Min.   :857.1   Min.   :6391   Min.   :4.1   Min.   :32.9  
 1st Qu.:14.1   1st Qu.:857.1   1st Qu.:6391   1st Qu.:4.1   1st Qu.:32.9  
 Median :14.1   Median :857.1   Median :6391   Median :4.1   Median :32.9  
 Mean   :14.1   Mean   :857.1   Mean   :6391   Mean   :4.1   Mean   :32.9  
 3rd Qu.:14.1   3rd Qu.:857.1   3rd Qu.:6391   3rd Qu.:4.1   3rd Qu.:32.9  
 Max.   :14.1   Max.   :857.1   Max.   :6391   Max.   :4.1   Max.   :32.9  

  F1sumchol3      F1pct_prot1     F1pct_pveg1   F1pct_pani1     F1pct_gluc1  
 Min.   :549.1   Min.   :18.8   Min.   :4.1   Min.   :14.7   Min.   :46.4  
 1st Qu.:549.1   1st Qu.:18.8   1st Qu.:4.1   1st Qu.:14.7   1st Qu.:46.4  
 Median :549.1   Median :18.8   Median :4.1   Median :14.7   Median :46.4  
 Mean   :549.1   Mean   :18.8   Mean   :4.1   Mean   :14.7   Mean   :46.4  
 3rd Qu.:549.1   3rd Qu.:18.8   3rd Qu.:4.1   3rd Qu.:14.7   3rd Qu.:46.4  
 Max.   :549.1   Max.   :18.8   Max.   :4.1   Max.   :14.7   Max.   :46.4  

 F1pct_gsuc1     F1pct_gpol1     F1pct_lipi1  F1pct_lsat1  F1pct_lmon1  
 Min.   :25.2   Min.   :21.2   Min.   :31   Min.   :14   Min.   :12.2  
 1st Qu.:25.2   1st Qu.:21.2   1st Qu.:31   1st Qu.:14   1st Qu.:12.2  
 Median :25.2   Median :21.2   Median :31   Median :14   Median :12.2  
 Mean   :25.2   Mean   :21.2   Mean   :31   Mean   :14   Mean   :12.2  
 3rd Qu.:25.2   3rd Qu.:21.2   3rd Qu.:31   3rd Qu.:14   3rd Qu.:12.2  
 Max.   :25.2   Max.   :21.2   Max.   :31   Max.   :14   Max.   :12.2  
```

```
  F1pct_lpol1   F1pct_fibr1   F1pct_chol1  F1pct_alco1   F1pct_prot3
 Min.   :2.6   Min.   :0.9   Min.   :15   Min.   :3.8   Min.   :19.6
 1st Qu.:2.6   1st Qu.:0.9   1st Qu.:15   1st Qu.:3.8   1st Qu.:19.6
 Median :2.6   Median :0.9   Median :15   Median :3.8   Median :19.6
 Mean   :2.6   Mean   :0.9   Mean   :15   Mean   :3.8   Mean   :19.6
 3rd Qu.:2.6   3rd Qu.:0.9   3rd Qu.:15   3rd Qu.:3.8   3rd Qu.:19.6
 Max.   :2.6   Max.   :0.9   Max.   :15   Max.   :3.8   Max.   :19.6

  F1pct_pveg3   F1pct_pani3    F1pct_gluc3    F1pct_gsuc3    F1pct_gpol3
 Min.   :4.2   Min.   :15.3   Min.   :48.3   Min.   :26.2   Min.   :22.1
 1st Qu.:4.2   1st Qu.:15.3   1st Qu.:48.3   1st Qu.:26.2   1st Qu.:22.1
 Median :4.2   Median :15.3   Median :48.3   Median :26.2   Median :22.1
 Mean   :4.2   Mean   :15.3   Mean   :48.3   Mean   :26.2   Mean   :22.1
 3rd Qu.:4.2   3rd Qu.:15.3   3rd Qu.:48.3   3rd Qu.:26.2   3rd Qu.:22.1
 Max.   :4.2   Max.   :15.3   Max.   :48.3   Max.   :26.2   Max.   :22.1

  F1pct_lipi3    F1pct_lsat3    F1pct_lmon3    F1pct_lpol3   F1pct_fibr3
 Min.   :32.3   Min.   :14.5   Min.   :12.7   Min.   :2.7   Min.   :0.9
 1st Qu.:32.3   1st Qu.:14.5   1st Qu.:12.7   1st Qu.:2.7   1st Qu.:0.9
 Median :32.3   Median :14.5   Median :12.7   Median :2.7   Median :0.9
 Mean   :32.3   Mean   :14.5   Mean   :12.7   Mean   :2.7   Mean   :0.9
 3rd Qu.:32.3   3rd Qu.:14.5   3rd Qu.:12.7   3rd Qu.:2.7   3rd Qu.:0.9
 Max.   :32.3   Max.   :14.5   Max.   :12.7   Max.   :2.7   Max.   :0.9

  F1pct_chol3       F1Fruits      F1Fruits_OK  F1Vegetables   F1Vegetables_OK
 Min.   :15.6   Min.   :2.696   Min.   :1    Min.   :1.714   Min.   :0
 1st Qu.:15.6   1st Qu.:2.696   1st Qu.:1    1st Qu.:1.714   1st Qu.:0
 Median :15.6   Median :2.696   Median :1    Median :1.714   Median :0
 Mean   :15.6   Mean   :2.696   Mean   :1    Mean   :1.714   Mean   :0
 3rd Qu.:15.6   3rd Qu.:2.696   3rd Qu.:1    3rd Qu.:1.714   3rd Qu.:0
 Max.   :15.6   Max.   :2.696   Max.   :1    Max.   :1.714   Max.   :0

     F1Meat      F1Meat_OK      F1Fish       F1Fish_OK     F1Fish2    F1Fish2_OK
 Min.   :2.5   Min.   :1   Min.   :1.5   Min.   :1   Min.   :0   Min.   :0
 1st Qu.:2.5   1st Qu.:1   1st Qu.:1.5   1st Qu.:1   1st Qu.:0   1st Qu.:0
 Median :2.5   Median :1   Median :1.5   Median :1   Median :0   Median :0
 Mean   :2.5   Mean   :1   Mean   :1.5   Mean   :1   Mean   :0   Mean   :0
 3rd Qu.:2.5   3rd Qu.:1   3rd Qu.:1.5   3rd Qu.:1   3rd Qu.:0   3rd Qu.:0
 Max.   :2.5   Max.   :1   Max.   :1.5   Max.   :1   Max.   :0   Max.   :0

    F1Dairy        F1Dairy_OK     F1Nb_OK      F1Cat_OK    F1Recom_OK    F1Nb2_OK
 Min.   :7.75   Min.   :1    Min.   :4   Min.   :4   Min.   :1    Min.   :3
 1st Qu.:7.75   1st Qu.:1    1st Qu.:4   1st Qu.:4   1st Qu.:1    1st Qu.:3
 Median :7.75   Median :1    Median :4   Median :4   Median :1    Median :3
 Mean   :7.75   Mean   :1    Mean   :4   Mean   :4   Mean   :1    Mean   :3
 3rd Qu.:7.75   3rd Qu.:1    3rd Qu.:4   3rd Qu.:4   3rd Qu.:1    3rd Qu.:3
 Max.   :7.75   Max.   :1    Max.   :4   Max.   :4   Max.   :1    Max.   :3

   F1Cat2_OK  F1Recom2_OK   F1Mediter1   F1Mediter2    F1AHEI1       F1AHEI2
 Min.   :3   Min.   :1    Min.   :6    Min.   :8    Min.   :45   Min.   :45
 1st Qu.:3   1st Qu.:1    1st Qu.:6    1st Qu.:8    1st Qu.:45   1st Qu.:45
 Median :3   Median :1    Median :6    Median :8    Median :45   Median :45
 Mean   :3   Mean   :1    Mean   :6    Mean   :8    Mean   :45   Mean   :45
 3rd Qu.:3   3rd Qu.:1    3rd Qu.:6    3rd Qu.:8    3rd Qu.:45   3rd Qu.:45
 Max.   :3   Max.   :1    Max.   :6    Max.   :8    Max.   :45   Max.   :45

  F1vegetarian   F2datquest             F2mrtsts     F2family18 famincome_b
 Min.   :0     Min.   :2016-12-08   Min.   : NA   Min.   :3    1:0
 1st Qu.:0     1st Qu.:2016-12-08   1st Qu.: NA   1st Qu.:3    2:1
 Median :0     Median :2016-12-08   Median : NA   Median :3    3:0
 Mean   :0     Mean   :2016-12-08   Mean   :NaN   Mean   :3
 3rd Qu.:0     3rd Qu.:2016-12-08   3rd Qu.: NA   3rd Qu.:3
 Max.   :0     Max.   :2016-12-08   Max.   : NA   Max.   :3
                                    NA's   :1
```

```
   F2alcool1      F2sbsmk      F2antiHTA     F2HTA      F2PSQI_score
 Min.   : NA   Min.   : NA   Min.   :0   Min.   : NA   Min.   : NA  
 1st Qu.: NA   1st Qu.: NA   1st Qu.:0   1st Qu.: NA   1st Qu.: NA  
 Median : NA   Median : NA   Median :0   Median : NA   Median : NA  
 Mean   :NaN   Mean   :NaN   Mean   :0   Mean   :NaN   Mean   :NaN  
 3rd Qu.: NA   3rd Qu.: NA   3rd Qu.:0   3rd Qu.: NA   3rd Qu.: NA  
 Max.   : NA   Max.   : NA   Max.   :0   Max.   : NA   Max.   : NA  
 NA's   :1     NA's   :1                 NA's   :1     NA's   :1    
     F2MME         F2CESD      F2depressed    F2BPI_cat     F2handgrip 
 Min.   : NA   Min.   : NA   Min.   : NA   Min.   : NA   Min.   : NA  
 1st Qu.: NA   1st Qu.: NA   1st Qu.: NA   1st Qu.: NA   1st Qu.: NA  
 Median : NA   Median : NA   Median : NA   Median : NA   Median : NA  
 Mean   :NaN   Mean   :NaN   Mean   :NaN   Mean   :NaN   Mean   :NaN  
 3rd Qu.: NA   3rd Qu.: NA   3rd Qu.: NA   3rd Qu.: NA   3rd Qu.: NA  
 Max.   : NA   Max.   : NA   Max.   : NA   Max.   : NA   Max.   : NA  
 NA's   :1     NA's   :1     NA's   :1     NA's   :1     NA's   :1    
      F2ht          F2BMI       F2BMI_cat1    F2BMI_cat2     F2waist   
 Min.   : NA   Min.   : NA   Min.   : NA   Min.   : NA   Min.   : NA  
 1st Qu.: NA   1st Qu.: NA   1st Qu.: NA   1st Qu.: NA   1st Qu.: NA  
 Median : NA   Median : NA   Median : NA   Median : NA   Median : NA  
 Mean   :NaN   Mean   :NaN   Mean   :NaN   Mean   :NaN   Mean   :NaN  
 3rd Qu.: NA   3rd Qu.: NA   3rd Qu.: NA   3rd Qu.: NA   3rd Qu.: NA  
 Max.   : NA   Max.   : NA   Max.   : NA   Max.   : NA   Max.   : NA  
 NA's   :1     NA's   :1     NA's   :1     NA's   :1     NA's   :1    
  F2waist_cat      F2hip          F2WHR         F2bmpsc       F2BIAcom   
 Min.   : NA   Min.   : NA   Min.   : NA   Min.   : NA   Min.   : NA  
 1st Qu.: NA   1st Qu.: NA   1st Qu.: NA   1st Qu.: NA   1st Qu.: NA  
 Median : NA   Median : NA   Median : NA   Median : NA   Median : NA  
 Mean   :NaN   Mean   :NaN   Mean   :NaN   Mean   :NaN   Mean   :NaN  
 3rd Qu.: NA   3rd Qu.: NA   3rd Qu.: NA   3rd Qu.: NA   3rd Qu.: NA  
 Max.   : NA   Max.   : NA   Max.   : NA   Max.   : NA   Max.   : NA  
 NA's   :1     NA's   :1     NA's   :1     NA's   :1     NA's   :1    
   F2Fasting     F2chol        F2hdlch        F2ldlch         F2trig   
 Min.   :2   Min.   : NA   Min.   : NA   Min.   : NA   Min.   : NA  
 1st Qu.:2   1st Qu.: NA   1st Qu.: NA   1st Qu.: NA   1st Qu.: NA  
 Median :2   Median : NA   Median : NA   Median : NA   Median : NA  
 Mean   :2   Mean   :NaN   Mean   :NaN   Mean   :NaN   Mean   :NaN  
 3rd Qu.:2   3rd Qu.: NA   3rd Qu.: NA   3rd Qu.: NA   3rd Qu.: NA  
 Max.   :2   Max.   : NA   Max.   : NA   Max.   : NA   Max.   : NA  
             NA's   :1     NA's   :1     NA's   :1     NA's   :1    
     F2gluc       F2insulin      F2hba1c       F2totprot       F2alb    
 Min.   : NA   Min.   : NA   Min.   : NA   Min.   : NA   Min.   : NA  
 1st Qu.: NA   1st Qu.: NA   1st Qu.: NA   1st Qu.: NA   1st Qu.: NA  
 Median : NA   Median : NA   Median : NA   Median : NA   Median : NA  
 Mean   :NaN   Mean   :NaN   Mean   :NaN   Mean   :NaN   Mean   :NaN  
 3rd Qu.: NA   3rd Qu.: NA   3rd Qu.: NA   3rd Qu.: NA   3rd Qu.: NA  
 Max.   : NA   Max.   : NA   Max.   : NA   Max.   : NA   Max.   : NA  
 NA's   :1     NA's   :1     NA's   :1     NA's   :1     NA's   :1    
     F2alkp      F2alkp_new      F2alat         F2asat        F2gamgt   
 Min.   : NA   Min.   : NA   Min.   : NA   Min.   : NA   Min.   : NA  
 1st Qu.: NA   1st Qu.: NA   1st Qu.: NA   1st Qu.: NA   1st Qu.: NA  
 Median : NA   Median : NA   Median : NA   Median : NA   Median : NA  
 Mean   :NaN   Mean   :NaN   Mean   :NaN   Mean   :NaN   Mean   :NaN  
 3rd Qu.: NA   3rd Qu.: NA   3rd Qu.: NA   3rd Qu.: NA   3rd Qu.: NA  
 Max.   : NA   Max.   : NA   Max.   : NA   Max.   : NA   Max.   : NA  
 NA's   :1     NA's   :1     NA's   :1     NA's   :1     NA's   :1    
    F2ProBNP     F2ProBNP_d   F2ProBNP_t          F2Troponin    F2Troponin_d
 Min.   : NA   Min.   : NA   Length:1           Min.   : NA   Min.   : NA  
 1st Qu.: NA   1st Qu.: NA   Class :character   1st Qu.: NA   1st Qu.: NA  
 Median : NA   Median : NA   Mode  :character   Median : NA   Median : NA  
 Mean   :NaN   Mean   :NaN                      Mean   :NaN   Mean   :NaN  
 3rd Qu.: NA   3rd Qu.: NA                      3rd Qu.: NA   3rd Qu.: NA  
 Max.   : NA   Max.   : NA                      Max.   : NA   Max.   : NA  
 NA's   :1     NA's   :1                        NA's   :1     NA's   :1    
```

```
 F2Troponin_t            F2na          F2k           F2ca          F2cap    
 Length:1           Min.   : NA   Min.   : NA   Min.   : NA   Min.   : NA  
 Class :character   1st Qu.: NA   1st Qu.: NA   1st Qu.: NA   1st Qu.: NA  
 Mode  :character   Median : NA   Median : NA   Median : NA   Median : NA  
                    Mean   :NaN   Mean   :NaN   Mean   :NaN   Mean   :NaN  
                    3rd Qu.: NA   3rd Qu.: NA   3rd Qu.: NA   3rd Qu.: NA  
                    Max.   : NA   Max.   : NA   Max.   : NA   Max.   : NA  
                    NA's   :1     NA's   :1     NA's   :1     NA's   :1    
      F2cr         F2uric     F2magnesium      F2iron      F2ferritin  
 Min.   : NA   Min.   : NA   Min.   : NA   Min.   : NA   Min.   : NA  
 1st Qu.: NA   1st Qu.: NA   1st Qu.: NA   1st Qu.: NA   1st Qu.: NA  
 Median : NA   Median : NA   Median : NA   Median : NA   Median : NA  
 Mean   :NaN   Mean   :NaN   Mean   :NaN   Mean   :NaN   Mean   :NaN  
 3rd Qu.: NA   3rd Qu.: NA   3rd Qu.: NA   3rd Qu.: NA   3rd Qu.: NA  
 Max.   : NA   Max.   : NA   Max.   : NA   Max.   : NA   Max.   : NA  
 NA's   :1     NA's   :1     NA's   :1     NA's   :1     NA's   :1    
 F2transferrin     F2crpu       F2crpu_d     F2crpu_t            F2il1b    
 Min.   : NA   Min.   : NA   Min.   : NA   Length:1           Min.   : NA  
 1st Qu.: NA   1st Qu.: NA   1st Qu.: NA   Class :character   1st Qu.: NA  
 Median : NA   Median : NA   Median : NA   Mode  :character   Median : NA  
 Mean   :NaN   Mean   :NaN   Mean   :NaN                      Mean   :NaN  
 3rd Qu.: NA   3rd Qu.: NA   3rd Qu.: NA                      3rd Qu.: NA  
 Max.   : NA   Max.   : NA   Max.   : NA                      Max.   : NA  
 NA's   :1     NA's   :1     NA's   :1                        NA's   :1    
     F2il6         F2tnfa        F2IFNg        F2il10        F2il8    
 Min.   : NA   Min.   : NA   Min.   : NA   Min.   : NA   Min.   : NA  
 1st Qu.: NA   1st Qu.: NA   1st Qu.: NA   1st Qu.: NA   1st Qu.: NA  
 Median : NA   Median : NA   Median : NA   Median : NA   Median : NA  
 Mean   :NaN   Mean   :NaN   Mean   :NaN   Mean   :NaN   Mean   :NaN  
 3rd Qu.: NA   3rd Qu.: NA   3rd Qu.: NA   3rd Qu.: NA   3rd Qu.: NA  
 Max.   : NA   Max.   : NA   Max.   : NA   Max.   : NA   Max.   : NA  
 NA's   :1     NA's   :1     NA's   :1     NA's   :1     NA's   :1    
    F2il1223       F2il16        F2tnfb        F2VEGF        F2TSH    
 Min.   : NA   Min.   : NA   Min.   : NA   Min.   : NA   Min.   : NA  
 1st Qu.: NA   1st Qu.: NA   1st Qu.: NA   1st Qu.: NA   1st Qu.: NA  
 Median : NA   Median : NA   Median : NA   Median : NA   Median : NA  
 Mean   :NaN   Mean   :NaN   Mean   :NaN   Mean   :NaN   Mean   :NaN  
 3rd Qu.: NA   3rd Qu.: NA   3rd Qu.: NA   3rd Qu.: NA   3rd Qu.: NA  
 Max.   : NA   Max.   : NA   Max.   : NA   Max.   : NA   Max.   : NA  
 NA's   :1     NA's   :1     NA's   :1     NA's   :1     NA's   :1    
     F2FT4     F2TSH_Comment          F2ery          F2hb       F2hematocr  
 Min.   : NA   Length:1           Min.   : NA   Min.   : NA   Min.   : NA  
 1st Qu.: NA   Class :character   1st Qu.: NA   1st Qu.: NA   1st Qu.: NA  
 Median : NA   Mode  :character   Median : NA   Median : NA   Median : NA  
 Mean   :NaN                      Mean   :NaN   Mean   :NaN   Mean   :NaN  
 3rd Qu.: NA                      3rd Qu.: NA   3rd Qu.: NA   3rd Qu.: NA  
 Max.   : NA                      Max.   : NA   Max.   : NA   Max.   : NA  
 NA's   :1                        NA's   :1     NA's   :1     NA's   :1    
     F2leuc        F2baso      F2baso_pct     F2eosino    F2eosino_pct 
 Min.   : NA   Min.   : NA   Min.   : NA   Min.   : NA   Min.   : NA  
 1st Qu.: NA   1st Qu.: NA   1st Qu.: NA   1st Qu.: NA   1st Qu.: NA  
 Median : NA   Median : NA   Median : NA   Median : NA   Median : NA  
 Mean   :NaN   Mean   :NaN   Mean   :NaN   Mean   :NaN   Mean   :NaN  
 3rd Qu.: NA   3rd Qu.: NA   3rd Qu.: NA   3rd Qu.: NA   3rd Qu.: NA  
 Max.   : NA   Max.   : NA   Max.   : NA   Max.   : NA   Max.   : NA  
 NA's   :1     NA's   :1     NA's   :1     NA's   :1     NA's   :1    
    F2lympho    F2lympho_pct     F2mono      F2mono_pct     F2neutro   
 Min.   : NA   Min.   : NA   Min.   : NA   Min.   : NA   Min.   : NA  
 1st Qu.: NA   1st Qu.: NA   1st Qu.: NA   1st Qu.: NA   1st Qu.: NA  
 Median : NA   Median : NA   Median : NA   Median : NA   Median : NA  
 Mean   :NaN   Mean   :NaN   Mean   :NaN   Mean   :NaN   Mean   :NaN  
 3rd Qu.: NA   3rd Qu.: NA   3rd Qu.: NA   3rd Qu.: NA   3rd Qu.: NA  
 Max.   : NA   Max.   : NA   Max.   : NA   Max.   : NA   Max.   : NA  
 NA's   :1     NA's   :1     NA's   :1     NA's   :1     NA's   :1    
```

```
 F2neutro_pct     F2mcv         F2mch         F2mchc         F2rdw     
 Min.   : NA   Min.   : NA   Min.   : NA   Min.   : NA   Min.   : NA  
 1st Qu.: NA   1st Qu.: NA   1st Qu.: NA   1st Qu.: NA   1st Qu.: NA  
 Median : NA   Median : NA   Median : NA   Median : NA   Median : NA  
 Mean   :NaN   Mean   :NaN   Mean   :NaN   Mean   :NaN   Mean   :NaN  
 3rd Qu.: NA   3rd Qu.: NA   3rd Qu.: NA   3rd Qu.: NA   3rd Qu.: NA  
 Max.   : NA   Max.   : NA   Max.   : NA   Max.   : NA   Max.   : NA  
 NA's   :1     NA's   :1     NA's   :1     NA's   :1     NA's   :1    
    F2plaq        F2mpv         F2pdw         F2cru         F2malb    
 Min.   : NA   Min.   : NA   Min.   : NA   Min.   : NA   Min.   : NA  
 1st Qu.: NA   1st Qu.: NA   1st Qu.: NA   1st Qu.: NA   1st Qu.: NA  
 Median : NA   Median : NA   Median : NA   Median : NA   Median : NA  
 Mean   :NaN   Mean   :NaN   Mean   :NaN   Mean   :NaN   Mean   :NaN  
 3rd Qu.: NA   3rd Qu.: NA   3rd Qu.: NA   3rd Qu.: NA   3rd Qu.: NA  
 Max.   : NA   Max.   : NA   Max.   : NA   Max.   : NA   Max.   : NA  
 NA's   :1     NA's   :1     NA's   :1     NA's   :1     NA's   :1    
    F2macr      F2Datepr          F2Dayofweek    F2Avlever     F2Heuredor  
 Min.   : NA   Length:1           Min.   : NA   Min.   : NA   Min.   : NA  
 1st Qu.: NA   Class :character   1st Qu.: NA   1st Qu.: NA   1st Qu.: NA  
 Median : NA   Mode  :character   Median : NA   Median : NA   Median : NA  
 Mean   :NaN                      Mean   :NaN   Mean   :NaN   Mean   :NaN  
 3rd Qu.: NA                      3rd Qu.: NA   3rd Qu.: NA   3rd Qu.: NA  
 Max.   : NA                      Max.   : NA   Max.   : NA   Max.   : NA  
 NA's   :1                        NA's   :1     NA's   :1     NA's   :1    
 F2Cortisol_CAR F2Cortisol_RT F2Cortisol_AVE F2Cortisol_PK F2Cortisol_SBP
 Min.   : NA    Min.   : NA   Min.   : NA    Min.   : NA   Min.   : NA   
 1st Qu.: NA    1st Qu.: NA   1st Qu.: NA    1st Qu.: NA   1st Qu.: NA   
 Median : NA    Median : NA   Median : NA    Median : NA   Median : NA   
 Mean   :NaN    Mean   :NaN   Mean   :NaN    Mean   :NaN   Mean   :NaN   
 3rd Qu.: NA    3rd Qu.: NA   3rd Qu.: NA    3rd Qu.: NA   3rd Qu.: NA   
 Max.   : NA    Max.   : NA   Max.   : NA    Max.   : NA   Max.   : NA   
 NA's   :1      NA's   :1     NA's   :1      NA's   :1     NA's   :1     
 F2Cortisol_AUCb F2Cortisol_AUCg F2Cortisol_AUCi F2Amylase_CAR  F2Amylase_RT
 Min.   : NA     Min.   : NA     Min.   : NA     Min.   : NA   Min.   : NA  
 1st Qu.: NA     1st Qu.: NA     1st Qu.: NA     1st Qu.: NA   1st Qu.: NA  
 Median : NA     Median : NA     Median : NA     Median : NA   Median : NA  
 Mean   :NaN     Mean   :NaN     Mean   :NaN     Mean   :NaN   Mean   :NaN  
 3rd Qu.: NA     3rd Qu.: NA     3rd Qu.: NA     3rd Qu.: NA   3rd Qu.: NA  
 Max.   : NA     Max.   : NA     Max.   : NA     Max.   : NA   Max.   : NA  
 NA's   :1       NA's   :1       NA's   :1       NA's   :1     NA's   :1    
 F2Amylase_AVE  F2Amylase_PK F2Amylase_SBP F2Amylase_AUCb F2Amylase_AUCg
 Min.   : NA   Min.   : NA   Min.   : NA   Min.   : NA    Min.   : NA   
 1st Qu.: NA   1st Qu.: NA   1st Qu.: NA   1st Qu.: NA    1st Qu.: NA   
 Median : NA   Median : NA   Median : NA   Median : NA    Median : NA   
 Mean   :NaN   Mean   :NaN   Mean   :NaN   Mean   :NaN    Mean   :NaN   
 3rd Qu.: NA   3rd Qu.: NA   3rd Qu.: NA   3rd Qu.: NA    3rd Qu.: NA   
 Max.   : NA   Max.   : NA   Max.   : NA   Max.   : NA    Max.   : NA   
 NA's   :1     NA's   :1     NA's   :1     NA's   :1      NA's   :1     
 F2Amylase_AUCi     F2SBP          F2DBP         F2HRTRTE     F2freqFFQ1  
 Min.   : NA    Min.   : NA   Min.   : NA   Min.   : NA   Min.   : NA  
 1st Qu.: NA    1st Qu.: NA   1st Qu.: NA   1st Qu.: NA   1st Qu.: NA  
 Median : NA    Median : NA   Median : NA   Median : NA   Median : NA  
 Mean   :NaN    Mean   :NaN   Mean   :NaN   Mean   :NaN   Mean   :NaN  
 3rd Qu.: NA    3rd Qu.: NA   3rd Qu.: NA   3rd Qu.: NA   3rd Qu.: NA  
 Max.   : NA    Max.   : NA   Max.   : NA   Max.   : NA   Max.   : NA  
 NA's   :1      NA's   :1     NA's   :1     NA's   :1     NA's   :1    
  F2freqFFQ2    F2freqFFQ3    F2freqFFQ4    F2freqFFQ5    F2freqFFQ6  
 Min.   : NA   Min.   : NA   Min.   : NA   Min.   : NA   Min.   : NA  
 1st Qu.: NA   1st Qu.: NA   1st Qu.: NA   1st Qu.: NA   1st Qu.: NA  
 Median : NA   Median : NA   Median : NA   Median : NA   Median : NA  
 Mean   :NaN   Mean   :NaN   Mean   :NaN   Mean   :NaN   Mean   :NaN  
 3rd Qu.: NA   3rd Qu.: NA   3rd Qu.: NA   3rd Qu.: NA   3rd Qu.: NA  
 Max.   : NA   Max.   : NA   Max.   : NA   Max.   : NA   Max.   : NA  
 NA's   :1     NA's   :1     NA's   :1     NA's   :1     NA's   :1    
```

```
  F2freqFFQ7    F2freqFFQ8    F2freqFFQ9   F2freqFFQ10   F2freqFFQ11 
 Min.   : NA   Min.   : NA   Min.   : NA   Min.   : NA   Min.   : NA  
 1st Qu.: NA   1st Qu.: NA   1st Qu.: NA   1st Qu.: NA   1st Qu.: NA  
 Median : NA   Median : NA   Median : NA   Median : NA   Median : NA  
 Mean   :NaN   Mean   :NaN   Mean   :NaN   Mean   :NaN   Mean   :NaN  
 3rd Qu.: NA   3rd Qu.: NA   3rd Qu.: NA   3rd Qu.: NA   3rd Qu.: NA  
 Max.   : NA   Max.   : NA   Max.   : NA   Max.   : NA   Max.   : NA  
 NA's   :1     NA's   :1     NA's   :1     NA's   :1     NA's   :1    
 F2freqFFQ12   F2freqFFQ13   F2freqFFQ14   F2freqFFQ15   F2freqFFQ16 
 Min.   : NA   Min.   : NA   Min.   : NA   Min.   : NA   Min.   : NA  
 1st Qu.: NA   1st Qu.: NA   1st Qu.: NA   1st Qu.: NA   1st Qu.: NA  
 Median : NA   Median : NA   Median : NA   Median : NA   Median : NA  
 Mean   :NaN   Mean   :NaN   Mean   :NaN   Mean   :NaN   Mean   :NaN  
 3rd Qu.: NA   3rd Qu.: NA   3rd Qu.: NA   3rd Qu.: NA   3rd Qu.: NA  
 Max.   : NA   Max.   : NA   Max.   : NA   Max.   : NA   Max.   : NA  
 NA's   :1     NA's   :1     NA's   :1     NA's   :1     NA's   :1    
 F2freqFFQ17   F2freqFFQ18   F2freqFFQ19   F2freqFFQ20   F2freqFFQ21 
 Min.   : NA   Min.   : NA   Min.   : NA   Min.   : NA   Min.   : NA  
 1st Qu.: NA   1st Qu.: NA   1st Qu.: NA   1st Qu.: NA   1st Qu.: NA  
 Median : NA   Median : NA   Median : NA   Median : NA   Median : NA  
 Mean   :NaN   Mean   :NaN   Mean   :NaN   Mean   :NaN   Mean   :NaN  
 3rd Qu.: NA   3rd Qu.: NA   3rd Qu.: NA   3rd Qu.: NA   3rd Qu.: NA  
 Max.   : NA   Max.   : NA   Max.   : NA   Max.   : NA   Max.   : NA  
 NA's   :1     NA's   :1     NA's   :1     NA's   :1     NA's   :1    
 F2freqFFQ22   F2freqFFQ23   F2freqFFQ24   F2freqFFQ25   F2freqFFQ26 
 Min.   : NA   Min.   : NA   Min.   : NA   Min.   : NA   Min.   : NA  
 1st Qu.: NA   1st Qu.: NA   1st Qu.: NA   1st Qu.: NA   1st Qu.: NA  
 Median : NA   Median : NA   Median : NA   Median : NA   Median : NA  
 Mean   :NaN   Mean   :NaN   Mean   :NaN   Mean   :NaN   Mean   :NaN  
 3rd Qu.: NA   3rd Qu.: NA   3rd Qu.: NA   3rd Qu.: NA   3rd Qu.: NA  
 Max.   : NA   Max.   : NA   Max.   : NA   Max.   : NA   Max.   : NA  
 NA's   :1     NA's   :1     NA's   :1     NA's   :1     NA's   :1    
 F2freqFFQ27   F2freqFFQ28   F2freqFFQ29   F2freqFFQ30   F2freqFFQ31 
 Min.   : NA   Min.   : NA   Min.   : NA   Min.   : NA   Min.   : NA  
 1st Qu.: NA   1st Qu.: NA   1st Qu.: NA   1st Qu.: NA   1st Qu.: NA  
 Median : NA   Median : NA   Median : NA   Median : NA   Median : NA  
 Mean   :NaN   Mean   :NaN   Mean   :NaN   Mean   :NaN   Mean   :NaN  
 3rd Qu.: NA   3rd Qu.: NA   3rd Qu.: NA   3rd Qu.: NA   3rd Qu.: NA  
 Max.   : NA   Max.   : NA   Max.   : NA   Max.   : NA   Max.   : NA  
 NA's   :1     NA's   :1     NA's   :1     NA's   :1     NA's   :1    
 F2freqFFQ32   F2freqFFQ33   F2freqFFQ34   F2freqFFQ35   F2freqFFQ36 
 Min.   : NA   Min.   : NA   Min.   : NA   Min.   : NA   Min.   : NA  
 1st Qu.: NA   1st Qu.: NA   1st Qu.: NA   1st Qu.: NA   1st Qu.: NA  
 Median : NA   Median : NA   Median : NA   Median : NA   Median : NA  
 Mean   :NaN   Mean   :NaN   Mean   :NaN   Mean   :NaN   Mean   :NaN  
 3rd Qu.: NA   3rd Qu.: NA   3rd Qu.: NA   3rd Qu.: NA   3rd Qu.: NA  
 Max.   : NA   Max.   : NA   Max.   : NA   Max.   : NA   Max.   : NA  
 NA's   :1     NA's   :1     NA's   :1     NA's   :1     NA's   :1    
 F2freqFFQ37   F2freqFFQ38   F2freqFFQ39   F2freqFFQ40   F2freqFFQ41 
 Min.   : NA   Min.   : NA   Min.   : NA   Min.   : NA   Min.   : NA  
 1st Qu.: NA   1st Qu.: NA   1st Qu.: NA   1st Qu.: NA   1st Qu.: NA  
 Median : NA   Median : NA   Median : NA   Median : NA   Median : NA  
 Mean   :NaN   Mean   :NaN   Mean   :NaN   Mean   :NaN   Mean   :NaN  
 3rd Qu.: NA   3rd Qu.: NA   3rd Qu.: NA   3rd Qu.: NA   3rd Qu.: NA  
 Max.   : NA   Max.   : NA   Max.   : NA   Max.   : NA   Max.   : NA  
 NA's   :1     NA's   :1     NA's   :1     NA's   :1     NA's   :1    
 F2freqFFQ42   F2freqFFQ43   F2freqFFQ44   F2freqFFQ45   F2freqFFQ46 
 Min.   : NA   Min.   : NA   Min.   : NA   Min.   : NA   Min.   : NA  
 1st Qu.: NA   1st Qu.: NA   1st Qu.: NA   1st Qu.: NA   1st Qu.: NA  
 Median : NA   Median : NA   Median : NA   Median : NA   Median : NA  
 Mean   :NaN   Mean   :NaN   Mean   :NaN   Mean   :NaN   Mean   :NaN  
 3rd Qu.: NA   3rd Qu.: NA   3rd Qu.: NA   3rd Qu.: NA   3rd Qu.: NA  
 Max.   : NA   Max.   : NA   Max.   : NA   Max.   : NA   Max.   : NA  
 NA's   :1     NA's   :1     NA's   :1     NA's   :1     NA's   :1    
```

```
  F2freqFFQ47    F2freqFFQ48    F2freqFFQ49    F2freqFFQ50    F2freqFFQ51  
 Min.   : NA   Min.   : NA   Min.   : NA   Min.   : NA   Min.   : NA  
 1st Qu.: NA   1st Qu.: NA   1st Qu.: NA   1st Qu.: NA   1st Qu.: NA  
 Median : NA   Median : NA   Median : NA   Median : NA   Median : NA  
 Mean   :NaN   Mean   :NaN   Mean   :NaN   Mean   :NaN   Mean   :NaN  
 3rd Qu.: NA   3rd Qu.: NA   3rd Qu.: NA   3rd Qu.: NA   3rd Qu.: NA  
 Max.   : NA   Max.   : NA   Max.   : NA   Max.   : NA   Max.   : NA  
 NA's   :1     NA's   :1     NA's   :1     NA's   :1     NA's   :1    
  F2freqFFQ52    F2freqFFQ53    F2freqFFQ54    F2freqFFQ55    F2freqFFQ56  
 Min.   : NA   Min.   : NA   Min.   : NA   Min.   : NA   Min.   : NA  
 1st Qu.: NA   1st Qu.: NA   1st Qu.: NA   1st Qu.: NA   1st Qu.: NA  
 Median : NA   Median : NA   Median : NA   Median : NA   Median : NA  
 Mean   :NaN   Mean   :NaN   Mean   :NaN   Mean   :NaN   Mean   :NaN  
 3rd Qu.: NA   3rd Qu.: NA   3rd Qu.: NA   3rd Qu.: NA   3rd Qu.: NA  
 Max.   : NA   Max.   : NA   Max.   : NA   Max.   : NA   Max.   : NA  
 NA's   :1     NA's   :1     NA's   :1     NA's   :1     NA's   :1    
  F2freqFFQ57    F2freqFFQ58    F2freqFFQ59    F2freqFFQ60    F2freqFFQ61  
 Min.   : NA   Min.   : NA   Min.   : NA   Min.   : NA   Min.   : NA  
 1st Qu.: NA   1st Qu.: NA   1st Qu.: NA   1st Qu.: NA   1st Qu.: NA  
 Median : NA   Median : NA   Median : NA   Median : NA   Median : NA  
 Mean   :NaN   Mean   :NaN   Mean   :NaN   Mean   :NaN   Mean   :NaN  
 3rd Qu.: NA   3rd Qu.: NA   3rd Qu.: NA   3rd Qu.: NA   3rd Qu.: NA  
 Max.   : NA   Max.   : NA   Max.   : NA   Max.   : NA   Max.   : NA  
 NA's   :1     NA's   :1     NA's   :1     NA's   :1     NA's   :1    
  F2freqFFQ62    F2freqFFQ63    F2freqFFQ64    F2freqFFQ65    F2freqFFQ66  
 Min.   : NA   Min.   : NA   Min.   : NA   Min.   : NA   Min.   : NA  
 1st Qu.: NA   1st Qu.: NA   1st Qu.: NA   1st Qu.: NA   1st Qu.: NA  
 Median : NA   Median : NA   Median : NA   Median : NA   Median : NA  
 Mean   :NaN   Mean   :NaN   Mean   :NaN   Mean   :NaN   Mean   :NaN  
 3rd Qu.: NA   3rd Qu.: NA   3rd Qu.: NA   3rd Qu.: NA   3rd Qu.: NA  
 Max.   : NA   Max.   : NA   Max.   : NA   Max.   : NA   Max.   : NA  
 NA's   :1     NA's   :1     NA's   :1     NA's   :1     NA's   :1    
  F2freqFFQ67    F2freqFFQ68    F2freqFFQ69    F2freqFFQ70    F2freqFFQ71  
 Min.   : NA   Min.   : NA   Min.   : NA   Min.   : NA   Min.   : NA  
 1st Qu.: NA   1st Qu.: NA   1st Qu.: NA   1st Qu.: NA   1st Qu.: NA  
 Median : NA   Median : NA   Median : NA   Median : NA   Median : NA  
 Mean   :NaN   Mean   :NaN   Mean   :NaN   Mean   :NaN   Mean   :NaN  
 3rd Qu.: NA   3rd Qu.: NA   3rd Qu.: NA   3rd Qu.: NA   3rd Qu.: NA  
 Max.   : NA   Max.   : NA   Max.   : NA   Max.   : NA   Max.   : NA  
 NA's   :1     NA's   :1     NA's   :1     NA's   :1     NA's   :1    
  F2freqFFQ72    F2freqFFQ73    F2freqFFQ74    F2freqFFQ75    F2freqFFQ76  
 Min.   : NA   Min.   : NA   Min.   : NA   Min.   : NA   Min.   : NA  
 1st Qu.: NA   1st Qu.: NA   1st Qu.: NA   1st Qu.: NA   1st Qu.: NA  
 Median : NA   Median : NA   Median : NA   Median : NA   Median : NA  
 Mean   :NaN   Mean   :NaN   Mean   :NaN   Mean   :NaN   Mean   :NaN  
 3rd Qu.: NA   3rd Qu.: NA   3rd Qu.: NA   3rd Qu.: NA   3rd Qu.: NA  
 Max.   : NA   Max.   : NA   Max.   : NA   Max.   : NA   Max.   : NA  
 NA's   :1     NA's   :1     NA's   :1     NA's   :1     NA's   :1    
  F2freqFFQ77    F2freqFFQ78    F2freqFFQ79    F2freqFFQ80    F2freqFFQ81  
 Min.   : NA   Min.   : NA   Min.   : NA   Min.   : NA   Min.   : NA  
 1st Qu.: NA   1st Qu.: NA   1st Qu.: NA   1st Qu.: NA   1st Qu.: NA  
 Median : NA   Median : NA   Median : NA   Median : NA   Median : NA  
 Mean   :NaN   Mean   :NaN   Mean   :NaN   Mean   :NaN   Mean   :NaN  
 3rd Qu.: NA   3rd Qu.: NA   3rd Qu.: NA   3rd Qu.: NA   3rd Qu.: NA  
 Max.   : NA   Max.   : NA   Max.   : NA   Max.   : NA   Max.   : NA  
 NA's   :1     NA's   :1     NA's   :1     NA's   :1     NA's   :1    
  F2freqFFQ82    F2freqFFQ83    F2freqFFQ84    F2freqFFQ85    F2freqFFQ86  
 Min.   : NA   Min.   : NA   Min.   : NA   Min.   : NA   Min.   : NA  
 1st Qu.: NA   1st Qu.: NA   1st Qu.: NA   1st Qu.: NA   1st Qu.: NA  
 Median : NA   Median : NA   Median : NA   Median : NA   Median : NA  
 Mean   :NaN   Mean   :NaN   Mean   :NaN   Mean   :NaN   Mean   :NaN  
 3rd Qu.: NA   3rd Qu.: NA   3rd Qu.: NA   3rd Qu.: NA   3rd Qu.: NA  
 Max.   : NA   Max.   : NA   Max.   : NA   Max.   : NA   Max.   : NA  
 NA's   :1     NA's   :1     NA's   :1     NA's   :1     NA's   :1    
```

```
  F2freqFFQ87   F2freqFFQ88   F2freqFFQ89   F2freqFFQ90   F2freqFFQ91
 Min.   : NA   Min.   : NA   Min.   : NA   Min.   : NA   Min.   : NA  
 1st Qu.: NA   1st Qu.: NA   1st Qu.: NA   1st Qu.: NA   1st Qu.: NA  
 Median : NA   Median : NA   Median : NA   Median : NA   Median : NA  
 Mean   :NaN   Mean   :NaN   Mean   :NaN   Mean   :NaN   Mean   :NaN  
 3rd Qu.: NA   3rd Qu.: NA   3rd Qu.: NA   3rd Qu.: NA   3rd Qu.: NA  
 Max.   : NA   Max.   : NA   Max.   : NA   Max.   : NA   Max.   : NA  
 NA's   :1     NA's   :1     NA's   :1     NA's   :1     NA's   :1    
  F2freqFFQ92   F2freqFFQ93   F2freqFFQ94   F2freqFFQ95   F2freqFFQ96
 Min.   : NA   Min.   : NA   Min.   : NA   Min.   : NA   Min.   : NA  
 1st Qu.: NA   1st Qu.: NA   1st Qu.: NA   1st Qu.: NA   1st Qu.: NA  
 Median : NA   Median : NA   Median : NA   Median : NA   Median : NA  
 Mean   :NaN   Mean   :NaN   Mean   :NaN   Mean   :NaN   Mean   :NaN  
 3rd Qu.: NA   3rd Qu.: NA   3rd Qu.: NA   3rd Qu.: NA   3rd Qu.: NA  
 Max.   : NA   Max.   : NA   Max.   : NA   Max.   : NA   Max.   : NA  
 NA's   :1     NA's   :1     NA's   :1     NA's   :1     NA's   :1    
  F2freqFFQ97   F2FFQ1amount  F2FFQ2amount  F2FFQ3amount  F2FFQ4amount
 Min.   : NA   Min.   : NA   Min.   : NA   Min.   : NA   Min.   : NA  
 1st Qu.: NA   1st Qu.: NA   1st Qu.: NA   1st Qu.: NA   1st Qu.: NA  
 Median : NA   Median : NA   Median : NA   Median : NA   Median : NA  
 Mean   :NaN   Mean   :NaN   Mean   :NaN   Mean   :NaN   Mean   :NaN  
 3rd Qu.: NA   3rd Qu.: NA   3rd Qu.: NA   3rd Qu.: NA   3rd Qu.: NA  
 Max.   : NA   Max.   : NA   Max.   : NA   Max.   : NA   Max.   : NA  
 NA's   :1     NA's   :1     NA's   :1     NA's   :1     NA's   :1    
  F2FFQ5amount  F2FFQ6amount  F2FFQ7amount  F2FFQ8amount  F2FFQ9amount
 Min.   : NA   Min.   : NA   Min.   : NA   Min.   : NA   Min.   : NA  
 1st Qu.: NA   1st Qu.: NA   1st Qu.: NA   1st Qu.: NA   1st Qu.: NA  
 Median : NA   Median : NA   Median : NA   Median : NA   Median : NA  
 Mean   :NaN   Mean   :NaN   Mean   :NaN   Mean   :NaN   Mean   :NaN  
 3rd Qu.: NA   3rd Qu.: NA   3rd Qu.: NA   3rd Qu.: NA   3rd Qu.: NA  
 Max.   : NA   Max.   : NA   Max.   : NA   Max.   : NA   Max.   : NA  
 NA's   :1     NA's   :1     NA's   :1     NA's   :1     NA's   :1    
 F2FFQ10amount F2FFQ11amount F2FFQ12amount F2FFQ13amount F2FFQ14amount
 Min.   : NA   Min.   : NA   Min.   : NA   Min.   : NA   Min.   : NA  
 1st Qu.: NA   1st Qu.: NA   1st Qu.: NA   1st Qu.: NA   1st Qu.: NA  
 Median : NA   Median : NA   Median : NA   Median : NA   Median : NA  
 Mean   :NaN   Mean   :NaN   Mean   :NaN   Mean   :NaN   Mean   :NaN  
 3rd Qu.: NA   3rd Qu.: NA   3rd Qu.: NA   3rd Qu.: NA   3rd Qu.: NA  
 Max.   : NA   Max.   : NA   Max.   : NA   Max.   : NA   Max.   : NA  
 NA's   :1     NA's   :1     NA's   :1     NA's   :1     NA's   :1    
 F2FFQ15amount F2FFQ16amount F2FFQ17amount F2FFQ18amount F2FFQ19amount
 Min.   : NA   Min.   : NA   Min.   : NA   Min.   : NA   Min.   : NA  
 1st Qu.: NA   1st Qu.: NA   1st Qu.: NA   1st Qu.: NA   1st Qu.: NA  
 Median : NA   Median : NA   Median : NA   Median : NA   Median : NA  
 Mean   :NaN   Mean   :NaN   Mean   :NaN   Mean   :NaN   Mean   :NaN  
 3rd Qu.: NA   3rd Qu.: NA   3rd Qu.: NA   3rd Qu.: NA   3rd Qu.: NA  
 Max.   : NA   Max.   : NA   Max.   : NA   Max.   : NA   Max.   : NA  
 NA's   :1     NA's   :1     NA's   :1     NA's   :1     NA's   :1    
 F2FFQ20amount F2FFQ21amount F2FFQ22amount F2FFQ23amount F2FFQ24amount
 Min.   : NA   Min.   : NA   Min.   : NA   Min.   : NA   Min.   : NA  
 1st Qu.: NA   1st Qu.: NA   1st Qu.: NA   1st Qu.: NA   1st Qu.: NA  
 Median : NA   Median : NA   Median : NA   Median : NA   Median : NA  
 Mean   :NaN   Mean   :NaN   Mean   :NaN   Mean   :NaN   Mean   :NaN  
 3rd Qu.: NA   3rd Qu.: NA   3rd Qu.: NA   3rd Qu.: NA   3rd Qu.: NA  
 Max.   : NA   Max.   : NA   Max.   : NA   Max.   : NA   Max.   : NA  
 NA's   :1     NA's   :1     NA's   :1     NA's   :1     NA's   :1    
 F2FFQ25amount F2FFQ26amount F2FFQ27amount F2FFQ28amount F2FFQ29amount
 Min.   : NA   Min.   : NA   Min.   : NA   Min.   : NA   Min.   : NA  
 1st Qu.: NA   1st Qu.: NA   1st Qu.: NA   1st Qu.: NA   1st Qu.: NA  
 Median : NA   Median : NA   Median : NA   Median : NA   Median : NA  
 Mean   :NaN   Mean   :NaN   Mean   :NaN   Mean   :NaN   Mean   :NaN  
 3rd Qu.: NA   3rd Qu.: NA   3rd Qu.: NA   3rd Qu.: NA   3rd Qu.: NA  
 Max.   : NA   Max.   : NA   Max.   : NA   Max.   : NA   Max.   : NA  
 NA's   :1     NA's   :1     NA's   :1     NA's   :1     NA's   :1    
```

```
 F2FFQ30amount F2FFQ31amount F2FFQ32amount F2FFQ33amount F2FFQ34amount
 Min.   : NA   Min.   : NA   Min.   : NA   Min.   : NA   Min.   : NA  
 1st Qu.: NA   1st Qu.: NA   1st Qu.: NA   1st Qu.: NA   1st Qu.: NA  
 Median : NA   Median : NA   Median : NA   Median : NA   Median : NA  
 Mean   :NaN   Mean   :NaN   Mean   :NaN   Mean   :NaN   Mean   :NaN  
 3rd Qu.: NA   3rd Qu.: NA   3rd Qu.: NA   3rd Qu.: NA   3rd Qu.: NA  
 Max.   : NA   Max.   : NA   Max.   : NA   Max.   : NA   Max.   : NA  
 NA's   :1     NA's   :1     NA's   :1     NA's   :1     NA's   :1    
 F2FFQ35amount F2FFQ36amount F2FFQ37amount F2FFQ38amount F2FFQ39amount
 Min.   : NA   Min.   : NA   Min.   : NA   Min.   : NA   Min.   : NA  
 1st Qu.: NA   1st Qu.: NA   1st Qu.: NA   1st Qu.: NA   1st Qu.: NA  
 Median : NA   Median : NA   Median : NA   Median : NA   Median : NA  
 Mean   :NaN   Mean   :NaN   Mean   :NaN   Mean   :NaN   Mean   :NaN  
 3rd Qu.: NA   3rd Qu.: NA   3rd Qu.: NA   3rd Qu.: NA   3rd Qu.: NA  
 Max.   : NA   Max.   : NA   Max.   : NA   Max.   : NA   Max.   : NA  
 NA's   :1     NA's   :1     NA's   :1     NA's   :1     NA's   :1    
 F2FFQ40amount F2FFQ41amount F2FFQ42amount F2FFQ43amount F2FFQ44amount
 Min.   : NA   Min.   : NA   Min.   : NA   Min.   : NA   Min.   : NA  
 1st Qu.: NA   1st Qu.: NA   1st Qu.: NA   1st Qu.: NA   1st Qu.: NA  
 Median : NA   Median : NA   Median : NA   Median : NA   Median : NA  
 Mean   :NaN   Mean   :NaN   Mean   :NaN   Mean   :NaN   Mean   :NaN  
 3rd Qu.: NA   3rd Qu.: NA   3rd Qu.: NA   3rd Qu.: NA   3rd Qu.: NA  
 Max.   : NA   Max.   : NA   Max.   : NA   Max.   : NA   Max.   : NA  
 NA's   :1     NA's   :1     NA's   :1     NA's   :1     NA's   :1    
 F2FFQ45amount F2FFQ46amount F2FFQ47amount F2FFQ48amount F2FFQ49amount
 Min.   : NA   Min.   : NA   Min.   : NA   Min.   : NA   Min.   : NA  
 1st Qu.: NA   1st Qu.: NA   1st Qu.: NA   1st Qu.: NA   1st Qu.: NA  
 Median : NA   Median : NA   Median : NA   Median : NA   Median : NA  
 Mean   :NaN   Mean   :NaN   Mean   :NaN   Mean   :NaN   Mean   :NaN  
 3rd Qu.: NA   3rd Qu.: NA   3rd Qu.: NA   3rd Qu.: NA   3rd Qu.: NA  
 Max.   : NA   Max.   : NA   Max.   : NA   Max.   : NA   Max.   : NA  
 NA's   :1     NA's   :1     NA's   :1     NA's   :1     NA's   :1    
 F2FFQ50amount F2FFQ51amount F2FFQ52amount F2FFQ53amount F2FFQ54amount
 Min.   : NA   Min.   : NA   Min.   : NA   Min.   : NA   Min.   : NA  
 1st Qu.: NA   1st Qu.: NA   1st Qu.: NA   1st Qu.: NA   1st Qu.: NA  
 Median : NA   Median : NA   Median : NA   Median : NA   Median : NA  
 Mean   :NaN   Mean   :NaN   Mean   :NaN   Mean   :NaN   Mean   :NaN  
 3rd Qu.: NA   3rd Qu.: NA   3rd Qu.: NA   3rd Qu.: NA   3rd Qu.: NA  
 Max.   : NA   Max.   : NA   Max.   : NA   Max.   : NA   Max.   : NA  
 NA's   :1     NA's   :1     NA's   :1     NA's   :1     NA's   :1    
 F2FFQ55amount F2FFQ56amount F2FFQ57amount F2FFQ58amount F2FFQ59amount
 Min.   : NA   Min.   : NA   Min.   : NA   Min.   : NA   Min.   : NA  
 1st Qu.: NA   1st Qu.: NA   1st Qu.: NA   1st Qu.: NA   1st Qu.: NA  
 Median : NA   Median : NA   Median : NA   Median : NA   Median : NA  
 Mean   :NaN   Mean   :NaN   Mean   :NaN   Mean   :NaN   Mean   :NaN  
 3rd Qu.: NA   3rd Qu.: NA   3rd Qu.: NA   3rd Qu.: NA   3rd Qu.: NA  
 Max.   : NA   Max.   : NA   Max.   : NA   Max.   : NA   Max.   : NA  
 NA's   :1     NA's   :1     NA's   :1     NA's   :1     NA's   :1    
 F2FFQ60amount F2FFQ61amount F2FFQ62amount F2FFQ63amount F2FFQ64amount
 Min.   : NA   Min.   : NA   Min.   : NA   Min.   : NA   Min.   : NA  
 1st Qu.: NA   1st Qu.: NA   1st Qu.: NA   1st Qu.: NA   1st Qu.: NA  
 Median : NA   Median : NA   Median : NA   Median : NA   Median : NA  
 Mean   :NaN   Mean   :NaN   Mean   :NaN   Mean   :NaN   Mean   :NaN  
 3rd Qu.: NA   3rd Qu.: NA   3rd Qu.: NA   3rd Qu.: NA   3rd Qu.: NA  
 Max.   : NA   Max.   : NA   Max.   : NA   Max.   : NA   Max.   : NA  
 NA's   :1     NA's   :1     NA's   :1     NA's   :1     NA's   :1    
 F2FFQ65amount F2FFQ66amount F2FFQ67amount F2FFQ68amount F2FFQ70amount
 Min.   : NA   Min.   : NA   Min.   : NA   Min.   : NA   Min.   : NA  
 1st Qu.: NA   1st Qu.: NA   1st Qu.: NA   1st Qu.: NA   1st Qu.: NA  
 Median : NA   Median : NA   Median : NA   Median : NA   Median : NA  
 Mean   :NaN   Mean   :NaN   Mean   :NaN   Mean   :NaN   Mean   :NaN  
 3rd Qu.: NA   3rd Qu.: NA   3rd Qu.: NA   3rd Qu.: NA   3rd Qu.: NA  
 Max.   : NA   Max.   : NA   Max.   : NA   Max.   : NA   Max.   : NA  
 NA's   :1     NA's   :1     NA's   :1     NA's   :1     NA's   :1    
```

```
 F2FFQ71amount F2FFQ72amount F2FFQ73amount F2FFQ74amount F2FFQ75amount
 Min.   : NA   Min.   : NA   Min.   : NA   Min.   : NA   Min.   : NA  
 1st Qu.: NA   1st Qu.: NA   1st Qu.: NA   1st Qu.: NA   1st Qu.: NA  
 Median : NA   Median : NA   Median : NA   Median : NA   Median : NA  
 Mean   :NaN   Mean   :NaN   Mean   :NaN   Mean   :NaN   Mean   :NaN  
 3rd Qu.: NA   3rd Qu.: NA   3rd Qu.: NA   3rd Qu.: NA   3rd Qu.: NA  
 Max.   : NA   Max.   : NA   Max.   : NA   Max.   : NA   Max.   : NA  
 NA's   :1     NA's   :1     NA's   :1     NA's   :1     NA's   :1    
 F2FFQ81amount F2FFQ82amount F2FFQ83amount F2FFQ84amount F2FFQ85amount
 Min.   : NA   Min.   : NA   Min.   : NA   Min.   : NA   Min.   : NA  
 1st Qu.: NA   1st Qu.: NA   1st Qu.: NA   1st Qu.: NA   1st Qu.: NA  
 Median : NA   Median : NA   Median : NA   Median : NA   Median : NA  
 Mean   :NaN   Mean   :NaN   Mean   :NaN   Mean   :NaN   Mean   :NaN  
 3rd Qu.: NA   3rd Qu.: NA   3rd Qu.: NA   3rd Qu.: NA   3rd Qu.: NA  
 Max.   : NA   Max.   : NA   Max.   : NA   Max.   : NA   Max.   : NA  
 NA's   :1     NA's   :1     NA's   :1     NA's   :1     NA's   :1    
 F2FFQ86amount F2FFQ87amount F2FFQ88amount F2FFQ89amount F2FFQ90amount
 Min.   : NA   Min.   : NA   Min.   : NA   Min.   : NA   Min.   : NA  
 1st Qu.: NA   1st Qu.: NA   1st Qu.: NA   1st Qu.: NA   1st Qu.: NA  
 Median : NA   Median : NA   Median : NA   Median : NA   Median : NA  
 Mean   :NaN   Mean   :NaN   Mean   :NaN   Mean   :NaN   Mean   :NaN  
 3rd Qu.: NA   3rd Qu.: NA   3rd Qu.: NA   3rd Qu.: NA   3rd Qu.: NA  
 Max.   : NA   Max.   : NA   Max.   : NA   Max.   : NA   Max.   : NA  
 NA's   :1     NA's   :1     NA's   :1     NA's   :1     NA's   :1    
 F2FFQ91amount F2FFQ92amount F2FFQ93amount F2FFQ94amount F2FFQ95amount
 Min.   : NA   Min.   : NA   Min.   : NA   Min.   : NA   Min.   : NA  
 1st Qu.: NA   1st Qu.: NA   1st Qu.: NA   1st Qu.: NA   1st Qu.: NA  
 Median : NA   Median : NA   Median : NA   Median : NA   Median : NA  
 Mean   :NaN   Mean   :NaN   Mean   :NaN   Mean   :NaN   Mean   :NaN  
 3rd Qu.: NA   3rd Qu.: NA   3rd Qu.: NA   3rd Qu.: NA   3rd Qu.: NA  
 Max.   : NA   Max.   : NA   Max.   : NA   Max.   : NA   Max.   : NA  
 NA's   :1     NA's   :1     NA's   :1     NA's   :1     NA's   :1    
 F2FFQ96amount F2FFQ97amount   F2numitems    F2totalcal    F2sumtot3  
 Min.   : NA   Min.   : NA   Min.   : NA   Min.   : NA   Min.   : NA  
 1st Qu.: NA   1st Qu.: NA   1st Qu.: NA   1st Qu.: NA   1st Qu.: NA  
 Median : NA   Median : NA   Median : NA   Median : NA   Median : NA  
 Mean   :NaN   Mean   :NaN   Mean   :NaN   Mean   :NaN   Mean   :NaN  
 3rd Qu.: NA   3rd Qu.: NA   3rd Qu.: NA   3rd Qu.: NA   3rd Qu.: NA  
 Max.   : NA   Max.   : NA   Max.   : NA   Max.   : NA   Max.   : NA  
 NA's   :1     NA's   :1     NA's   :1     NA's   :1     NA's   :1    
   F2sumprot1    F2sumpveg1    F2sumpani1    F2sumgluc1    F2sumgsuc1  
 Min.   : NA   Min.   : NA   Min.   : NA   Min.   : NA   Min.   : NA  
 1st Qu.: NA   1st Qu.: NA   1st Qu.: NA   1st Qu.: NA   1st Qu.: NA  
 Median : NA   Median : NA   Median : NA   Median : NA   Median : NA  
 Mean   :NaN   Mean   :NaN   Mean   :NaN   Mean   :NaN   Mean   :NaN  
 3rd Qu.: NA   3rd Qu.: NA   3rd Qu.: NA   3rd Qu.: NA   3rd Qu.: NA  
 Max.   : NA   Max.   : NA   Max.   : NA   Max.   : NA   Max.   : NA  
 NA's   :1     NA's   :1     NA's   :1     NA's   :1     NA's   :1    
   F2sumgpol1    F2sumlipi1    F2sumlsat1    F2sumlmon1    F2sumlpol1  
 Min.   : NA   Min.   : NA   Min.   : NA   Min.   : NA   Min.   : NA  
 1st Qu.: NA   1st Qu.: NA   1st Qu.: NA   1st Qu.: NA   1st Qu.: NA  
 Median : NA   Median : NA   Median : NA   Median : NA   Median : NA  
 Mean   :NaN   Mean   :NaN   Mean   :NaN   Mean   :NaN   Mean   :NaN  
 3rd Qu.: NA   3rd Qu.: NA   3rd Qu.: NA   3rd Qu.: NA   3rd Qu.: NA  
 Max.   : NA   Max.   : NA   Max.   : NA   Max.   : NA   Max.   : NA  
 NA's   :1     NA's   :1     NA's   :1     NA's   :1     NA's   :1    
   F2sumfibr1    F2sumchol1    F2sumalco     F2sumcalc1    F2sumfer1   
 Min.   : NA   Min.   : NA   Min.   : NA   Min.   : NA   Min.   : NA  
 1st Qu.: NA   1st Qu.: NA   1st Qu.: NA   1st Qu.: NA   1st Qu.: NA  
 Median : NA   Median : NA   Median : NA   Median : NA   Median : NA  
 Mean   :NaN   Mean   :NaN   Mean   :NaN   Mean   :NaN   Mean   :NaN  
 3rd Qu.: NA   3rd Qu.: NA   3rd Qu.: NA   3rd Qu.: NA   3rd Qu.: NA  
 Max.   : NA   Max.   : NA   Max.   : NA   Max.   : NA   Max.   : NA  
 NA's   :1     NA's   :1     NA's   :1     NA's   :1     NA's   :1    
```

```
   F2sumret1      F2sumcaro1     F2sumvitd1     F2sumeau1      F2sumprot3  
 Min.   : NA   Min.   : NA   Min.   : NA   Min.   : NA   Min.   : NA  
 1st Qu.: NA   1st Qu.: NA   1st Qu.: NA   1st Qu.: NA   1st Qu.: NA  
 Median : NA   Median : NA   Median : NA   Median : NA   Median : NA  
 Mean   :NaN   Mean   :NaN   Mean   :NaN   Mean   :NaN   Mean   :NaN  
 3rd Qu.: NA   3rd Qu.: NA   3rd Qu.: NA   3rd Qu.: NA   3rd Qu.: NA  
 Max.   : NA   Max.   : NA   Max.   : NA   Max.   : NA   Max.   : NA  
 NA's   :1     NA's   :1     NA's   :1     NA's   :1     NA's   :1    
   F2sumpveg3     F2sumpani3     F2sumgluc3     F2sumgsuc3     F2sumgpol3  
 Min.   : NA   Min.   : NA   Min.   : NA   Min.   : NA   Min.   : NA  
 1st Qu.: NA   1st Qu.: NA   1st Qu.: NA   1st Qu.: NA   1st Qu.: NA  
 Median : NA   Median : NA   Median : NA   Median : NA   Median : NA  
 Mean   :NaN   Mean   :NaN   Mean   :NaN   Mean   :NaN   Mean   :NaN  
 3rd Qu.: NA   3rd Qu.: NA   3rd Qu.: NA   3rd Qu.: NA   3rd Qu.: NA  
 Max.   : NA   Max.   : NA   Max.   : NA   Max.   : NA   Max.   : NA  
 NA's   :1     NA's   :1     NA's   :1     NA's   :1     NA's   :1    
   F2sumlipi3     F2sumlsat3     F2sumlmon3     F2sumlpol3     F2sumcalc3  
 Min.   : NA   Min.   : NA   Min.   : NA   Min.   : NA   Min.   : NA  
 1st Qu.: NA   1st Qu.: NA   1st Qu.: NA   1st Qu.: NA   1st Qu.: NA  
 Median : NA   Median : NA   Median : NA   Median : NA   Median : NA  
 Mean   :NaN   Mean   :NaN   Mean   :NaN   Mean   :NaN   Mean   :NaN  
 3rd Qu.: NA   3rd Qu.: NA   3rd Qu.: NA   3rd Qu.: NA   3rd Qu.: NA  
 Max.   : NA   Max.   : NA   Max.   : NA   Max.   : NA   Max.   : NA  
 NA's   :1     NA's   :1     NA's   :1     NA's   :1     NA's   :1    
   F2sumfer3      F2sumret3      F2sumcaro3     F2sumvitd3     F2sumfibr3  
 Min.   : NA   Min.   : NA   Min.   : NA   Min.   : NA   Min.   : NA  
 1st Qu.: NA   1st Qu.: NA   1st Qu.: NA   1st Qu.: NA   1st Qu.: NA  
 Median : NA   Median : NA   Median : NA   Median : NA   Median : NA  
 Mean   :NaN   Mean   :NaN   Mean   :NaN   Mean   :NaN   Mean   :NaN  
 3rd Qu.: NA   3rd Qu.: NA   3rd Qu.: NA   3rd Qu.: NA   3rd Qu.: NA  
 Max.   : NA   Max.   : NA   Max.   : NA   Max.   : NA   Max.   : NA  
 NA's   :1     NA's   :1     NA's   :1     NA's   :1     NA's   :1    
   F2sumchol3    F2pct_prot1    F2pct_pveg1    F2pct_pani1    F2pct_gluc1  
 Min.   : NA   Min.   : NA   Min.   : NA   Min.   : NA   Min.   : NA  
 1st Qu.: NA   1st Qu.: NA   1st Qu.: NA   1st Qu.: NA   1st Qu.: NA  
 Median : NA   Median : NA   Median : NA   Median : NA   Median : NA  
 Mean   :NaN   Mean   :NaN   Mean   :NaN   Mean   :NaN   Mean   :NaN  
 3rd Qu.: NA   3rd Qu.: NA   3rd Qu.: NA   3rd Qu.: NA   3rd Qu.: NA  
 Max.   : NA   Max.   : NA   Max.   : NA   Max.   : NA   Max.   : NA  
 NA's   :1     NA's   :1     NA's   :1     NA's   :1     NA's   :1    
  F2pct_gsuc1    F2pct_gpol1    F2pct_lipi1    F2pct_lsat1    F2pct_lmon1  
 Min.   : NA   Min.   : NA   Min.   : NA   Min.   : NA   Min.   : NA  
 1st Qu.: NA   1st Qu.: NA   1st Qu.: NA   1st Qu.: NA   1st Qu.: NA  
 Median : NA   Median : NA   Median : NA   Median : NA   Median : NA  
 Mean   :NaN   Mean   :NaN   Mean   :NaN   Mean   :NaN   Mean   :NaN  
 3rd Qu.: NA   3rd Qu.: NA   3rd Qu.: NA   3rd Qu.: NA   3rd Qu.: NA  
 Max.   : NA   Max.   : NA   Max.   : NA   Max.   : NA   Max.   : NA  
 NA's   :1     NA's   :1     NA's   :1     NA's   :1     NA's   :1    
  F2pct_lpol1    F2pct_fibr1    F2pct_chol1    F2pct_alco1    F2pct_prot3  
 Min.   : NA   Min.   : NA   Min.   : NA   Min.   : NA   Min.   : NA  
 1st Qu.: NA   1st Qu.: NA   1st Qu.: NA   1st Qu.: NA   1st Qu.: NA  
 Median : NA   Median : NA   Median : NA   Median : NA   Median : NA  
 Mean   :NaN   Mean   :NaN   Mean   :NaN   Mean   :NaN   Mean   :NaN  
 3rd Qu.: NA   3rd Qu.: NA   3rd Qu.: NA   3rd Qu.: NA   3rd Qu.: NA  
 Max.   : NA   Max.   : NA   Max.   : NA   Max.   : NA   Max.   : NA  
 NA's   :1     NA's   :1     NA's   :1     NA's   :1     NA's   :1    
  F2pct_pveg3    F2pct_pani3    F2pct_gluc3    F2pct_gsuc3    F2pct_gpol3  
 Min.   : NA   Min.   : NA   Min.   : NA   Min.   : NA   Min.   : NA  
 1st Qu.: NA   1st Qu.: NA   1st Qu.: NA   1st Qu.: NA   1st Qu.: NA  
 Median : NA   Median : NA   Median : NA   Median : NA   Median : NA  
 Mean   :NaN   Mean   :NaN   Mean   :NaN   Mean   :NaN   Mean   :NaN  
 3rd Qu.: NA   3rd Qu.: NA   3rd Qu.: NA   3rd Qu.: NA   3rd Qu.: NA  
 Max.   : NA   Max.   : NA   Max.   : NA   Max.   : NA   Max.   : NA  
 NA's   :1     NA's   :1     NA's   :1     NA's   :1     NA's   :1    
```

```
  F2pct_lipi3   F2pct_lsat3   F2pct_lmon3   F2pct_lpol3   F2pct_fibr3
 Min.   : NA   Min.   : NA   Min.   : NA   Min.   : NA   Min.   : NA
 1st Qu.: NA   1st Qu.: NA   1st Qu.: NA   1st Qu.: NA   1st Qu.: NA
 Median : NA   Median : NA   Median : NA   Median : NA   Median : NA
 Mean   :NaN   Mean   :NaN   Mean   :NaN   Mean   :NaN   Mean   :NaN
 3rd Qu.: NA   3rd Qu.: NA   3rd Qu.: NA   3rd Qu.: NA   3rd Qu.: NA
 Max.   : NA   Max.   : NA   Max.   : NA   Max.   : NA   Max.   : NA
 NA's   :1     NA's   :1     NA's   :1     NA's   :1     NA's   :1
  F2pct_chol3     F2Fruits    F2Fruits_OK   F2Vegetables F2Vegetables_OK
 Min.   : NA   Min.   : NA   Min.   : NA   Min.   : NA   Min.   : NA
 1st Qu.: NA   1st Qu.: NA   1st Qu.: NA   1st Qu.: NA   1st Qu.: NA
 Median : NA   Median : NA   Median : NA   Median : NA   Median : NA
 Mean   :NaN   Mean   :NaN   Mean   :NaN   Mean   :NaN   Mean   :NaN
 3rd Qu.: NA   3rd Qu.: NA   3rd Qu.: NA   3rd Qu.: NA   3rd Qu.: NA
 Max.   : NA   Max.   : NA   Max.   : NA   Max.   : NA   Max.   : NA
 NA's   :1     NA's   :1     NA's   :1     NA's   :1     NA's   :1
     F2Meat      F2Meat_OK       F2Fish       F2Fish_OK      F2Fish2
 Min.   : NA   Min.   : NA   Min.   : NA   Min.   : NA   Min.   : NA
 1st Qu.: NA   1st Qu.: NA   1st Qu.: NA   1st Qu.: NA   1st Qu.: NA
 Median : NA   Median : NA   Median : NA   Median : NA   Median : NA
 Mean   :NaN   Mean   :NaN   Mean   :NaN   Mean   :NaN   Mean   :NaN
 3rd Qu.: NA   3rd Qu.: NA   3rd Qu.: NA   3rd Qu.: NA   3rd Qu.: NA
 Max.   : NA   Max.   : NA   Max.   : NA   Max.   : NA   Max.   : NA
 NA's   :1     NA's   :1     NA's   :1     NA's   :1     NA's   :1
   F2Fish2_OK     F2Dairy      F2Dairy_OK     F2Nb_OK       F2Cat_OK
 Min.   : NA   Min.   : NA   Min.   : NA   Min.   : NA   Min.   : NA
 1st Qu.: NA   1st Qu.: NA   1st Qu.: NA   1st Qu.: NA   1st Qu.: NA
 Median : NA   Median : NA   Median : NA   Median : NA   Median : NA
 Mean   :NaN   Mean   :NaN   Mean   :NaN   Mean   :NaN   Mean   :NaN
 3rd Qu.: NA   3rd Qu.: NA   3rd Qu.: NA   3rd Qu.: NA   3rd Qu.: NA
 Max.   : NA   Max.   : NA   Max.   : NA   Max.   : NA   Max.   : NA
 NA's   :1     NA's   :1     NA's   :1     NA's   :1     NA's   :1
   F2Recom_OK     F2Nb2_OK     F2Cat2_OK     F2Recom2_OK    F2Mediter1
 Min.   : NA   Min.   : NA   Min.   : NA   Min.   : NA   Min.   : NA
 1st Qu.: NA   1st Qu.: NA   1st Qu.: NA   1st Qu.: NA   1st Qu.: NA
 Median : NA   Median : NA   Median : NA   Median : NA   Median : NA
 Mean   :NaN   Mean   :NaN   Mean   :NaN   Mean   :NaN   Mean   :NaN
 3rd Qu.: NA   3rd Qu.: NA   3rd Qu.: NA   3rd Qu.: NA   3rd Qu.: NA
 Max.   : NA   Max.   : NA   Max.   : NA   Max.   : NA   Max.   : NA
 NA's   :1     NA's   :1     NA's   :1     NA's   :1     NA's   :1
   F2Mediter2     F2AHEI1       F2AHEI2     F2vegetarian   F3datquest
 Min.   : NA   Min.   : NA   Min.   : NA   Min.   :0     Min.   :2020-05-12
 1st Qu.: NA   1st Qu.: NA   1st Qu.: NA   1st Qu.:0     1st Qu.:2020-05-12
 Median : NA   Median : NA   Median : NA   Median :0     Median :2020-05-12
 Mean   :NaN   Mean   :NaN   Mean   :NaN   Mean   :0     Mean   :2020-05-12
 3rd Qu.: NA   3rd Qu.: NA   3rd Qu.: NA   3rd Qu.:0     3rd Qu.:2020-05-12
 Max.   : NA   Max.   : NA   Max.   : NA   Max.   :0     Max.   :2020-05-12
 NA's   :1     NA's   :1     NA's   :1
    F3mrtsts        F3MME         F3CESD      F3depressed    F3Fasting
 Min.   : NA   Min.   : NA   Min.   : NA   Min.   : NA   Min.   : NA
 1st Qu.: NA   1st Qu.: NA   1st Qu.: NA   1st Qu.: NA   1st Qu.: NA
 Median : NA   Median : NA   Median : NA   Median : NA   Median : NA
 Mean   :NaN   Mean   :NaN   Mean   :NaN   Mean   :NaN   Mean   :NaN
 3rd Qu.: NA   3rd Qu.: NA   3rd Qu.: NA   3rd Qu.: NA   3rd Qu.: NA
 Max.   : NA   Max.   : NA   Max.   : NA   Max.   : NA   Max.   : NA
 NA's   :1     NA's   :1     NA's   :1     NA's   :1     NA's   :1
     F3chol       F3hdlch       F3ldlch         F3trig        F3gluc
 Min.   : NA   Min.   : NA   Min.   : NA   Min.   : NA   Min.   : NA
 1st Qu.: NA   1st Qu.: NA   1st Qu.: NA   1st Qu.: NA   1st Qu.: NA
 Median : NA   Median : NA   Median : NA   Median : NA   Median : NA
 Mean   :NaN   Mean   :NaN   Mean   :NaN   Mean   :NaN   Mean   :NaN
 3rd Qu.: NA   3rd Qu.: NA   3rd Qu.: NA   3rd Qu.: NA   3rd Qu.: NA
 Max.   : NA   Max.   : NA   Max.   : NA   Max.   : NA   Max.   : NA
 NA's   :1     NA's   :1     NA's   :1     NA's   :1     NA's   :1
```

```
   F3insulin      F3hba1c       F3hba1s        F3crpu      F3freqFFQ1 
 Min.   : NA   Min.   : NA   Min.   : NA   Min.   : NA   Min.   : NA  
 1st Qu.: NA   1st Qu.: NA   1st Qu.: NA   1st Qu.: NA   1st Qu.: NA  
 Median : NA   Median : NA   Median : NA   Median : NA   Median : NA  
 Mean   :NaN   Mean   :NaN   Mean   :NaN   Mean   :NaN   Mean   :NaN  
 3rd Qu.: NA   3rd Qu.: NA   3rd Qu.: NA   3rd Qu.: NA   3rd Qu.: NA  
 Max.   : NA   Max.   : NA   Max.   : NA   Max.   : NA   Max.   : NA  
 NA's   :1     NA's   :1     NA's   :1     NA's   :1     NA's   :1    
   F3freqFFQ2    F3freqFFQ3    F3freqFFQ4    F3freqFFQ5    F3freqFFQ6 
 Min.   : NA   Min.   : NA   Min.   : NA   Min.   : NA   Min.   : NA  
 1st Qu.: NA   1st Qu.: NA   1st Qu.: NA   1st Qu.: NA   1st Qu.: NA  
 Median : NA   Median : NA   Median : NA   Median : NA   Median : NA  
 Mean   :NaN   Mean   :NaN   Mean   :NaN   Mean   :NaN   Mean   :NaN  
 3rd Qu.: NA   3rd Qu.: NA   3rd Qu.: NA   3rd Qu.: NA   3rd Qu.: NA  
 Max.   : NA   Max.   : NA   Max.   : NA   Max.   : NA   Max.   : NA  
 NA's   :1     NA's   :1     NA's   :1     NA's   :1     NA's   :1    
   F3freqFFQ7    F3freqFFQ8    F3freqFFQ9   F3freqFFQ10   F3freqFFQ11 
 Min.   : NA   Min.   : NA   Min.   : NA   Min.   : NA   Min.   : NA  
 1st Qu.: NA   1st Qu.: NA   1st Qu.: NA   1st Qu.: NA   1st Qu.: NA  
 Median : NA   Median : NA   Median : NA   Median : NA   Median : NA  
 Mean   :NaN   Mean   :NaN   Mean   :NaN   Mean   :NaN   Mean   :NaN  
 3rd Qu.: NA   3rd Qu.: NA   3rd Qu.: NA   3rd Qu.: NA   3rd Qu.: NA  
 Max.   : NA   Max.   : NA   Max.   : NA   Max.   : NA   Max.   : NA  
 NA's   :1     NA's   :1     NA's   :1     NA's   :1     NA's   :1    
  F3freqFFQ12   F3freqFFQ13   F3freqFFQ14   F3freqFFQ15   F3freqFFQ16 
 Min.   : NA   Min.   : NA   Min.   : NA   Min.   : NA   Min.   : NA  
 1st Qu.: NA   1st Qu.: NA   1st Qu.: NA   1st Qu.: NA   1st Qu.: NA  
 Median : NA   Median : NA   Median : NA   Median : NA   Median : NA  
 Mean   :NaN   Mean   :NaN   Mean   :NaN   Mean   :NaN   Mean   :NaN  
 3rd Qu.: NA   3rd Qu.: NA   3rd Qu.: NA   3rd Qu.: NA   3rd Qu.: NA  
 Max.   : NA   Max.   : NA   Max.   : NA   Max.   : NA   Max.   : NA  
 NA's   :1     NA's   :1     NA's   :1     NA's   :1     NA's   :1    
  F3freqFFQ17   F3freqFFQ18   F3freqFFQ19   F3freqFFQ20   F3freqFFQ21 
 Min.   : NA   Min.   : NA   Min.   : NA   Min.   : NA   Min.   : NA  
 1st Qu.: NA   1st Qu.: NA   1st Qu.: NA   1st Qu.: NA   1st Qu.: NA  
 Median : NA   Median : NA   Median : NA   Median : NA   Median : NA  
 Mean   :NaN   Mean   :NaN   Mean   :NaN   Mean   :NaN   Mean   :NaN  
 3rd Qu.: NA   3rd Qu.: NA   3rd Qu.: NA   3rd Qu.: NA   3rd Qu.: NA  
 Max.   : NA   Max.   : NA   Max.   : NA   Max.   : NA   Max.   : NA  
 NA's   :1     NA's   :1     NA's   :1     NA's   :1     NA's   :1    
  F3freqFFQ22   F3freqFFQ23   F3freqFFQ24   F3freqFFQ25   F3freqFFQ26 
 Min.   : NA   Min.   : NA   Min.   : NA   Min.   : NA   Min.   : NA  
 1st Qu.: NA   1st Qu.: NA   1st Qu.: NA   1st Qu.: NA   1st Qu.: NA  
 Median : NA   Median : NA   Median : NA   Median : NA   Median : NA  
 Mean   :NaN   Mean   :NaN   Mean   :NaN   Mean   :NaN   Mean   :NaN  
 3rd Qu.: NA   3rd Qu.: NA   3rd Qu.: NA   3rd Qu.: NA   3rd Qu.: NA  
 Max.   : NA   Max.   : NA   Max.   : NA   Max.   : NA   Max.   : NA  
 NA's   :1     NA's   :1     NA's   :1     NA's   :1     NA's   :1    
  F3freqFFQ27   F3freqFFQ28   F3freqFFQ29   F3freqFFQ30   F3freqFFQ31 
 Min.   : NA   Min.   : NA   Min.   : NA   Min.   : NA   Min.   : NA  
 1st Qu.: NA   1st Qu.: NA   1st Qu.: NA   1st Qu.: NA   1st Qu.: NA  
 Median : NA   Median : NA   Median : NA   Median : NA   Median : NA  
 Mean   :NaN   Mean   :NaN   Mean   :NaN   Mean   :NaN   Mean   :NaN  
 3rd Qu.: NA   3rd Qu.: NA   3rd Qu.: NA   3rd Qu.: NA   3rd Qu.: NA  
 Max.   : NA   Max.   : NA   Max.   : NA   Max.   : NA   Max.   : NA  
 NA's   :1     NA's   :1     NA's   :1     NA's   :1     NA's   :1    
  F3freqFFQ32   F3freqFFQ33   F3freqFFQ34   F3freqFFQ35   F3freqFFQ36 
 Min.   : NA   Min.   : NA   Min.   : NA   Min.   : NA   Min.   : NA  
 1st Qu.: NA   1st Qu.: NA   1st Qu.: NA   1st Qu.: NA   1st Qu.: NA  
 Median : NA   Median : NA   Median : NA   Median : NA   Median : NA  
 Mean   :NaN   Mean   :NaN   Mean   :NaN   Mean   :NaN   Mean   :NaN  
 3rd Qu.: NA   3rd Qu.: NA   3rd Qu.: NA   3rd Qu.: NA   3rd Qu.: NA  
 Max.   : NA   Max.   : NA   Max.   : NA   Max.   : NA   Max.   : NA  
 NA's   :1     NA's   :1     NA's   :1     NA's   :1     NA's   :1    
```

```
  F3freqFFQ37   F3freqFFQ38   F3freqFFQ39   F3freqFFQ40   F3freqFFQ41  
 Min.   : NA   Min.   : NA   Min.   : NA   Min.   : NA   Min.   : NA  
 1st Qu.: NA   1st Qu.: NA   1st Qu.: NA   1st Qu.: NA   1st Qu.: NA  
 Median : NA   Median : NA   Median : NA   Median : NA   Median : NA  
 Mean   :NaN   Mean   :NaN   Mean   :NaN   Mean   :NaN   Mean   :NaN  
 3rd Qu.: NA   3rd Qu.: NA   3rd Qu.: NA   3rd Qu.: NA   3rd Qu.: NA  
 Max.   : NA   Max.   : NA   Max.   : NA   Max.   : NA   Max.   : NA  
 NA's   :1     NA's   :1     NA's   :1     NA's   :1     NA's   :1    
  F3freqFFQ42   F3freqFFQ43   F3freqFFQ44   F3freqFFQ45   F3freqFFQ46  
 Min.   : NA   Min.   : NA   Min.   : NA   Min.   : NA   Min.   : NA  
 1st Qu.: NA   1st Qu.: NA   1st Qu.: NA   1st Qu.: NA   1st Qu.: NA  
 Median : NA   Median : NA   Median : NA   Median : NA   Median : NA  
 Mean   :NaN   Mean   :NaN   Mean   :NaN   Mean   :NaN   Mean   :NaN  
 3rd Qu.: NA   3rd Qu.: NA   3rd Qu.: NA   3rd Qu.: NA   3rd Qu.: NA  
 Max.   : NA   Max.   : NA   Max.   : NA   Max.   : NA   Max.   : NA  
 NA's   :1     NA's   :1     NA's   :1     NA's   :1     NA's   :1    
  F3freqFFQ47   F3freqFFQ48   F3freqFFQ49   F3freqFFQ50   F3freqFFQ51  
 Min.   : NA   Min.   : NA   Min.   : NA   Min.   : NA   Min.   : NA  
 1st Qu.: NA   1st Qu.: NA   1st Qu.: NA   1st Qu.: NA   1st Qu.: NA  
 Median : NA   Median : NA   Median : NA   Median : NA   Median : NA  
 Mean   :NaN   Mean   :NaN   Mean   :NaN   Mean   :NaN   Mean   :NaN  
 3rd Qu.: NA   3rd Qu.: NA   3rd Qu.: NA   3rd Qu.: NA   3rd Qu.: NA  
 Max.   : NA   Max.   : NA   Max.   : NA   Max.   : NA   Max.   : NA  
 NA's   :1     NA's   :1     NA's   :1     NA's   :1     NA's   :1    
  F3freqFFQ52   F3freqFFQ53   F3freqFFQ54   F3freqFFQ55   F3freqFFQ56  
 Min.   : NA   Min.   : NA   Min.   : NA   Min.   : NA   Min.   : NA  
 1st Qu.: NA   1st Qu.: NA   1st Qu.: NA   1st Qu.: NA   1st Qu.: NA  
 Median : NA   Median : NA   Median : NA   Median : NA   Median : NA  
 Mean   :NaN   Mean   :NaN   Mean   :NaN   Mean   :NaN   Mean   :NaN  
 3rd Qu.: NA   3rd Qu.: NA   3rd Qu.: NA   3rd Qu.: NA   3rd Qu.: NA  
 Max.   : NA   Max.   : NA   Max.   : NA   Max.   : NA   Max.   : NA  
 NA's   :1     NA's   :1     NA's   :1     NA's   :1     NA's   :1    
  F3freqFFQ57   F3freqFFQ58   F3freqFFQ59   F3freqFFQ60   F3freqFFQ61  
 Min.   : NA   Min.   : NA   Min.   : NA   Min.   : NA   Min.   : NA  
 1st Qu.: NA   1st Qu.: NA   1st Qu.: NA   1st Qu.: NA   1st Qu.: NA  
 Median : NA   Median : NA   Median : NA   Median : NA   Median : NA  
 Mean   :NaN   Mean   :NaN   Mean   :NaN   Mean   :NaN   Mean   :NaN  
 3rd Qu.: NA   3rd Qu.: NA   3rd Qu.: NA   3rd Qu.: NA   3rd Qu.: NA  
 Max.   : NA   Max.   : NA   Max.   : NA   Max.   : NA   Max.   : NA  
 NA's   :1     NA's   :1     NA's   :1     NA's   :1     NA's   :1    
  F3freqFFQ62   F3freqFFQ63   F3freqFFQ64   F3freqFFQ65   F3freqFFQ66  
 Min.   : NA   Min.   : NA   Min.   : NA   Min.   : NA   Min.   : NA  
 1st Qu.: NA   1st Qu.: NA   1st Qu.: NA   1st Qu.: NA   1st Qu.: NA  
 Median : NA   Median : NA   Median : NA   Median : NA   Median : NA  
 Mean   :NaN   Mean   :NaN   Mean   :NaN   Mean   :NaN   Mean   :NaN  
 3rd Qu.: NA   3rd Qu.: NA   3rd Qu.: NA   3rd Qu.: NA   3rd Qu.: NA  
 Max.   : NA   Max.   : NA   Max.   : NA   Max.   : NA   Max.   : NA  
 NA's   :1     NA's   :1     NA's   :1     NA's   :1     NA's   :1    
  F3freqFFQ67   F3freqFFQ68   F3freqFFQ69   F3freqFFQ70   F3freqFFQ71  
 Min.   : NA   Min.   : NA   Min.   : NA   Min.   : NA   Min.   : NA  
 1st Qu.: NA   1st Qu.: NA   1st Qu.: NA   1st Qu.: NA   1st Qu.: NA  
 Median : NA   Median : NA   Median : NA   Median : NA   Median : NA  
 Mean   :NaN   Mean   :NaN   Mean   :NaN   Mean   :NaN   Mean   :NaN  
 3rd Qu.: NA   3rd Qu.: NA   3rd Qu.: NA   3rd Qu.: NA   3rd Qu.: NA  
 Max.   : NA   Max.   : NA   Max.   : NA   Max.   : NA   Max.   : NA  
 NA's   :1     NA's   :1     NA's   :1     NA's   :1     NA's   :1    
  F3freqFFQ72   F3freqFFQ73   F3freqFFQ74   F3freqFFQ75   F3freqFFQ76  
 Min.   : NA   Min.   : NA   Min.   : NA   Min.   : NA   Min.   : NA  
 1st Qu.: NA   1st Qu.: NA   1st Qu.: NA   1st Qu.: NA   1st Qu.: NA  
 Median : NA   Median : NA   Median : NA   Median : NA   Median : NA  
 Mean   :NaN   Mean   :NaN   Mean   :NaN   Mean   :NaN   Mean   :NaN  
 3rd Qu.: NA   3rd Qu.: NA   3rd Qu.: NA   3rd Qu.: NA   3rd Qu.: NA  
 Max.   : NA   Max.   : NA   Max.   : NA   Max.   : NA   Max.   : NA  
 NA's   :1     NA's   :1     NA's   :1     NA's   :1     NA's   :1    
```

```
  F3freqFFQ77   F3freqFFQ78   F3freqFFQ79   F3freqFFQ80   F3freqFFQ81 
 Min.   : NA   Min.   : NA   Min.   : NA   Min.   : NA   Min.   : NA  
 1st Qu.: NA   1st Qu.: NA   1st Qu.: NA   1st Qu.: NA   1st Qu.: NA  
 Median : NA   Median : NA   Median : NA   Median : NA   Median : NA  
 Mean   :NaN   Mean   :NaN   Mean   :NaN   Mean   :NaN   Mean   :NaN  
 3rd Qu.: NA   3rd Qu.: NA   3rd Qu.: NA   3rd Qu.: NA   3rd Qu.: NA  
 Max.   : NA   Max.   : NA   Max.   : NA   Max.   : NA   Max.   : NA  
 NA's   :1     NA's   :1     NA's   :1     NA's   :1     NA's   :1    
  F3freqFFQ82   F3freqFFQ83   F3freqFFQ84   F3freqFFQ85   F3freqFFQ86 
 Min.   : NA   Min.   : NA   Min.   : NA   Min.   : NA   Min.   : NA  
 1st Qu.: NA   1st Qu.: NA   1st Qu.: NA   1st Qu.: NA   1st Qu.: NA  
 Median : NA   Median : NA   Median : NA   Median : NA   Median : NA  
 Mean   :NaN   Mean   :NaN   Mean   :NaN   Mean   :NaN   Mean   :NaN  
 3rd Qu.: NA   3rd Qu.: NA   3rd Qu.: NA   3rd Qu.: NA   3rd Qu.: NA  
 Max.   : NA   Max.   : NA   Max.   : NA   Max.   : NA   Max.   : NA  
 NA's   :1     NA's   :1     NA's   :1     NA's   :1     NA's   :1    
  F3freqFFQ87   F3freqFFQ88   F3freqFFQ89   F3freqFFQ90   F3freqFFQ91 
 Min.   : NA   Min.   : NA   Min.   : NA   Min.   : NA   Min.   : NA  
 1st Qu.: NA   1st Qu.: NA   1st Qu.: NA   1st Qu.: NA   1st Qu.: NA  
 Median : NA   Median : NA   Median : NA   Median : NA   Median : NA  
 Mean   :NaN   Mean   :NaN   Mean   :NaN   Mean   :NaN   Mean   :NaN  
 3rd Qu.: NA   3rd Qu.: NA   3rd Qu.: NA   3rd Qu.: NA   3rd Qu.: NA  
 Max.   : NA   Max.   : NA   Max.   : NA   Max.   : NA   Max.   : NA  
 NA's   :1     NA's   :1     NA's   :1     NA's   :1     NA's   :1    
  F3freqFFQ92   F3freqFFQ93   F3freqFFQ94   F3freqFFQ95   F3freqFFQ96 
 Min.   : NA   Min.   : NA   Min.   : NA   Min.   : NA   Min.   : NA  
 1st Qu.: NA   1st Qu.: NA   1st Qu.: NA   1st Qu.: NA   1st Qu.: NA  
 Median : NA   Median : NA   Median : NA   Median : NA   Median : NA  
 Mean   :NaN   Mean   :NaN   Mean   :NaN   Mean   :NaN   Mean   :NaN  
 3rd Qu.: NA   3rd Qu.: NA   3rd Qu.: NA   3rd Qu.: NA   3rd Qu.: NA  
 Max.   : NA   Max.   : NA   Max.   : NA   Max.   : NA   Max.   : NA  
 NA's   :1     NA's   :1     NA's   :1     NA's   :1     NA's   :1    
  F3freqFFQ97   F3FFQ1amount  F3FFQ2amount  F3FFQ3amount  F3FFQ4amount
 Min.   : NA   Min.   : NA   Min.   : NA   Min.   : NA   Min.   : NA  
 1st Qu.: NA   1st Qu.: NA   1st Qu.: NA   1st Qu.: NA   1st Qu.: NA  
 Median : NA   Median : NA   Median : NA   Median : NA   Median : NA  
 Mean   :NaN   Mean   :NaN   Mean   :NaN   Mean   :NaN   Mean   :NaN  
 3rd Qu.: NA   3rd Qu.: NA   3rd Qu.: NA   3rd Qu.: NA   3rd Qu.: NA  
 Max.   : NA   Max.   : NA   Max.   : NA   Max.   : NA   Max.   : NA  
 NA's   :1     NA's   :1     NA's   :1     NA's   :1     NA's   :1    
  F3FFQ5amount  F3FFQ6amount  F3FFQ7amount  F3FFQ8amount  F3FFQ9amount
 Min.   : NA   Min.   : NA   Min.   : NA   Min.   : NA   Min.   : NA  
 1st Qu.: NA   1st Qu.: NA   1st Qu.: NA   1st Qu.: NA   1st Qu.: NA  
 Median : NA   Median : NA   Median : NA   Median : NA   Median : NA  
 Mean   :NaN   Mean   :NaN   Mean   :NaN   Mean   :NaN   Mean   :NaN  
 3rd Qu.: NA   3rd Qu.: NA   3rd Qu.: NA   3rd Qu.: NA   3rd Qu.: NA  
 Max.   : NA   Max.   : NA   Max.   : NA   Max.   : NA   Max.   : NA  
 NA's   :1     NA's   :1     NA's   :1     NA's   :1     NA's   :1    
 F3FFQ10amount F3FFQ11amount F3FFQ12amount F3FFQ13amount F3FFQ14amount
 Min.   : NA   Min.   : NA   Min.   : NA   Min.   : NA   Min.   : NA  
 1st Qu.: NA   1st Qu.: NA   1st Qu.: NA   1st Qu.: NA   1st Qu.: NA  
 Median : NA   Median : NA   Median : NA   Median : NA   Median : NA  
 Mean   :NaN   Mean   :NaN   Mean   :NaN   Mean   :NaN   Mean   :NaN  
 3rd Qu.: NA   3rd Qu.: NA   3rd Qu.: NA   3rd Qu.: NA   3rd Qu.: NA  
 Max.   : NA   Max.   : NA   Max.   : NA   Max.   : NA   Max.   : NA  
 NA's   :1     NA's   :1     NA's   :1     NA's   :1     NA's   :1    
 F3FFQ15amount F3FFQ16amount F3FFQ17amount F3FFQ18amount F3FFQ19amount
 Min.   : NA   Min.   : NA   Min.   : NA   Min.   : NA   Min.   : NA  
 1st Qu.: NA   1st Qu.: NA   1st Qu.: NA   1st Qu.: NA   1st Qu.: NA  
 Median : NA   Median : NA   Median : NA   Median : NA   Median : NA  
 Mean   :NaN   Mean   :NaN   Mean   :NaN   Mean   :NaN   Mean   :NaN  
 3rd Qu.: NA   3rd Qu.: NA   3rd Qu.: NA   3rd Qu.: NA   3rd Qu.: NA  
 Max.   : NA   Max.   : NA   Max.   : NA   Max.   : NA   Max.   : NA  
 NA's   :1     NA's   :1     NA's   :1     NA's   :1     NA's   :1    
```

```
 F3FFQ20amount F3FFQ21amount F3FFQ22amount F3FFQ23amount F3FFQ24amount
 Min.   : NA   Min.   : NA   Min.   : NA   Min.   : NA   Min.   : NA  
 1st Qu.: NA   1st Qu.: NA   1st Qu.: NA   1st Qu.: NA   1st Qu.: NA  
 Median : NA   Median : NA   Median : NA   Median : NA   Median : NA  
 Mean   :NaN   Mean   :NaN   Mean   :NaN   Mean   :NaN   Mean   :NaN  
 3rd Qu.: NA   3rd Qu.: NA   3rd Qu.: NA   3rd Qu.: NA   3rd Qu.: NA  
 Max.   : NA   Max.   : NA   Max.   : NA   Max.   : NA   Max.   : NA  
 NA's   :1     NA's   :1     NA's   :1     NA's   :1     NA's   :1    
 F3FFQ25amount F3FFQ26amount F3FFQ27amount F3FFQ28amount F3FFQ29amount
 Min.   : NA   Min.   : NA   Min.   : NA   Min.   : NA   Min.   : NA  
 1st Qu.: NA   1st Qu.: NA   1st Qu.: NA   1st Qu.: NA   1st Qu.: NA  
 Median : NA   Median : NA   Median : NA   Median : NA   Median : NA  
 Mean   :NaN   Mean   :NaN   Mean   :NaN   Mean   :NaN   Mean   :NaN  
 3rd Qu.: NA   3rd Qu.: NA   3rd Qu.: NA   3rd Qu.: NA   3rd Qu.: NA  
 Max.   : NA   Max.   : NA   Max.   : NA   Max.   : NA   Max.   : NA  
 NA's   :1     NA's   :1     NA's   :1     NA's   :1     NA's   :1    
 F3FFQ30amount F3FFQ31amount F3FFQ32amount F3FFQ33amount F3FFQ34amount
 Min.   : NA   Min.   : NA   Min.   : NA   Min.   : NA   Min.   : NA  
 1st Qu.: NA   1st Qu.: NA   1st Qu.: NA   1st Qu.: NA   1st Qu.: NA  
 Median : NA   Median : NA   Median : NA   Median : NA   Median : NA  
 Mean   :NaN   Mean   :NaN   Mean   :NaN   Mean   :NaN   Mean   :NaN  
 3rd Qu.: NA   3rd Qu.: NA   3rd Qu.: NA   3rd Qu.: NA   3rd Qu.: NA  
 Max.   : NA   Max.   : NA   Max.   : NA   Max.   : NA   Max.   : NA  
 NA's   :1     NA's   :1     NA's   :1     NA's   :1     NA's   :1    
 F3FFQ35amount F3FFQ36amount F3FFQ37amount F3FFQ38amount F3FFQ39amount
 Min.   : NA   Min.   : NA   Min.   : NA   Min.   : NA   Min.   : NA  
 1st Qu.: NA   1st Qu.: NA   1st Qu.: NA   1st Qu.: NA   1st Qu.: NA  
 Median : NA   Median : NA   Median : NA   Median : NA   Median : NA  
 Mean   :NaN   Mean   :NaN   Mean   :NaN   Mean   :NaN   Mean   :NaN  
 3rd Qu.: NA   3rd Qu.: NA   3rd Qu.: NA   3rd Qu.: NA   3rd Qu.: NA  
 Max.   : NA   Max.   : NA   Max.   : NA   Max.   : NA   Max.   : NA  
 NA's   :1     NA's   :1     NA's   :1     NA's   :1     NA's   :1    
 F3FFQ40amount F3FFQ41amount F3FFQ42amount F3FFQ43amount F3FFQ44amount
 Min.   : NA   Min.   : NA   Min.   : NA   Min.   : NA   Min.   : NA  
 1st Qu.: NA   1st Qu.: NA   1st Qu.: NA   1st Qu.: NA   1st Qu.: NA  
 Median : NA   Median : NA   Median : NA   Median : NA   Median : NA  
 Mean   :NaN   Mean   :NaN   Mean   :NaN   Mean   :NaN   Mean   :NaN  
 3rd Qu.: NA   3rd Qu.: NA   3rd Qu.: NA   3rd Qu.: NA   3rd Qu.: NA  
 Max.   : NA   Max.   : NA   Max.   : NA   Max.   : NA   Max.   : NA  
 NA's   :1     NA's   :1     NA's   :1     NA's   :1     NA's   :1    
 F3FFQ45amount F3FFQ46amount F3FFQ47amount F3FFQ48amount F3FFQ49amount
 Min.   : NA   Min.   : NA   Min.   : NA   Min.   : NA   Min.   : NA  
 1st Qu.: NA   1st Qu.: NA   1st Qu.: NA   1st Qu.: NA   1st Qu.: NA  
 Median : NA   Median : NA   Median : NA   Median : NA   Median : NA  
 Mean   :NaN   Mean   :NaN   Mean   :NaN   Mean   :NaN   Mean   :NaN  
 3rd Qu.: NA   3rd Qu.: NA   3rd Qu.: NA   3rd Qu.: NA   3rd Qu.: NA  
 Max.   : NA   Max.   : NA   Max.   : NA   Max.   : NA   Max.   : NA  
 NA's   :1     NA's   :1     NA's   :1     NA's   :1     NA's   :1    
 F3FFQ50amount F3FFQ51amount F3FFQ52amount F3FFQ53amount F3FFQ54amount
 Min.   : NA   Min.   : NA   Min.   : NA   Min.   : NA   Min.   : NA  
 1st Qu.: NA   1st Qu.: NA   1st Qu.: NA   1st Qu.: NA   1st Qu.: NA  
 Median : NA   Median : NA   Median : NA   Median : NA   Median : NA  
 Mean   :NaN   Mean   :NaN   Mean   :NaN   Mean   :NaN   Mean   :NaN  
 3rd Qu.: NA   3rd Qu.: NA   3rd Qu.: NA   3rd Qu.: NA   3rd Qu.: NA  
 Max.   : NA   Max.   : NA   Max.   : NA   Max.   : NA   Max.   : NA  
 NA's   :1     NA's   :1     NA's   :1     NA's   :1     NA's   :1    
 F3FFQ55amount F3FFQ56amount F3FFQ57amount F3FFQ58amount F3FFQ59amount
 Min.   : NA   Min.   : NA   Min.   : NA   Min.   : NA   Min.   : NA  
 1st Qu.: NA   1st Qu.: NA   1st Qu.: NA   1st Qu.: NA   1st Qu.: NA  
 Median : NA   Median : NA   Median : NA   Median : NA   Median : NA  
 Mean   :NaN   Mean   :NaN   Mean   :NaN   Mean   :NaN   Mean   :NaN  
 3rd Qu.: NA   3rd Qu.: NA   3rd Qu.: NA   3rd Qu.: NA   3rd Qu.: NA  
 Max.   : NA   Max.   : NA   Max.   : NA   Max.   : NA   Max.   : NA  
 NA's   :1     NA's   :1     NA's   :1     NA's   :1     NA's   :1    
```

```
F3FFQ60amount F3FFQ61amount F3FFQ62amount F3FFQ63amount F3FFQ64amount
Min.   : NA   Min.   : NA   Min.   : NA   Min.   : NA   Min.   : NA  
1st Qu.: NA   1st Qu.: NA   1st Qu.: NA   1st Qu.: NA   1st Qu.: NA  
Median : NA   Median : NA   Median : NA   Median : NA   Median : NA  
Mean   :NaN   Mean   :NaN   Mean   :NaN   Mean   :NaN   Mean   :NaN  
3rd Qu.: NA   3rd Qu.: NA   3rd Qu.: NA   3rd Qu.: NA   3rd Qu.: NA  
Max.   : NA   Max.   : NA   Max.   : NA   Max.   : NA   Max.   : NA  
NA's   :1     NA's   :1     NA's   :1     NA's   :1     NA's   :1    
F3FFQ65amount F3FFQ66amount F3FFQ67amount F3FFQ68amount F3FFQ70amount
Min.   : NA   Min.   : NA   Min.   : NA   Min.   : NA   Min.   : NA  
1st Qu.: NA   1st Qu.: NA   1st Qu.: NA   1st Qu.: NA   1st Qu.: NA  
Median : NA   Median : NA   Median : NA   Median : NA   Median : NA  
Mean   :NaN   Mean   :NaN   Mean   :NaN   Mean   :NaN   Mean   :NaN  
3rd Qu.: NA   3rd Qu.: NA   3rd Qu.: NA   3rd Qu.: NA   3rd Qu.: NA  
Max.   : NA   Max.   : NA   Max.   : NA   Max.   : NA   Max.   : NA  
NA's   :1     NA's   :1     NA's   :1     NA's   :1     NA's   :1    
F3FFQ71amount F3FFQ72amount F3FFQ73amount F3FFQ74amount F3FFQ75amount
Min.   : NA   Min.   : NA   Min.   : NA   Min.   : NA   Min.   : NA  
1st Qu.: NA   1st Qu.: NA   1st Qu.: NA   1st Qu.: NA   1st Qu.: NA  
Median : NA   Median : NA   Median : NA   Median : NA   Median : NA  
Mean   :NaN   Mean   :NaN   Mean   :NaN   Mean   :NaN   Mean   :NaN  
3rd Qu.: NA   3rd Qu.: NA   3rd Qu.: NA   3rd Qu.: NA   3rd Qu.: NA  
Max.   : NA   Max.   : NA   Max.   : NA   Max.   : NA   Max.   : NA  
NA's   :1     NA's   :1     NA's   :1     NA's   :1     NA's   :1    
F3FFQ81amount F3FFQ82amount F3FFQ83amount F3FFQ84amount F3FFQ85amount
Min.   : NA   Min.   : NA   Min.   : NA   Min.   : NA   Min.   : NA  
1st Qu.: NA   1st Qu.: NA   1st Qu.: NA   1st Qu.: NA   1st Qu.: NA  
Median : NA   Median : NA   Median : NA   Median : NA   Median : NA  
Mean   :NaN   Mean   :NaN   Mean   :NaN   Mean   :NaN   Mean   :NaN  
3rd Qu.: NA   3rd Qu.: NA   3rd Qu.: NA   3rd Qu.: NA   3rd Qu.: NA  
Max.   : NA   Max.   : NA   Max.   : NA   Max.   : NA   Max.   : NA  
NA's   :1     NA's   :1     NA's   :1     NA's   :1     NA's   :1    
F3FFQ86amount F3FFQ87amount F3FFQ88amount F3FFQ89amount F3FFQ90amount
Min.   : NA   Min.   : NA   Min.   : NA   Min.   : NA   Min.   : NA  
1st Qu.: NA   1st Qu.: NA   1st Qu.: NA   1st Qu.: NA   1st Qu.: NA  
Median : NA   Median : NA   Median : NA   Median : NA   Median : NA  
Mean   :NaN   Mean   :NaN   Mean   :NaN   Mean   :NaN   Mean   :NaN  
3rd Qu.: NA   3rd Qu.: NA   3rd Qu.: NA   3rd Qu.: NA   3rd Qu.: NA  
Max.   : NA   Max.   : NA   Max.   : NA   Max.   : NA   Max.   : NA  
NA's   :1     NA's   :1     NA's   :1     NA's   :1     NA's   :1    
F3FFQ91amount F3FFQ92amount F3FFQ93amount F3FFQ94amount F3FFQ95amount
Min.   : NA   Min.   : NA   Min.   : NA   Min.   : NA   Min.   : NA  
1st Qu.: NA   1st Qu.: NA   1st Qu.: NA   1st Qu.: NA   1st Qu.: NA  
Median : NA   Median : NA   Median : NA   Median : NA   Median : NA  
Mean   :NaN   Mean   :NaN   Mean   :NaN   Mean   :NaN   Mean   :NaN  
3rd Qu.: NA   3rd Qu.: NA   3rd Qu.: NA   3rd Qu.: NA   3rd Qu.: NA  
Max.   : NA   Max.   : NA   Max.   : NA   Max.   : NA   Max.   : NA  
NA's   :1     NA's   :1     NA's   :1     NA's   :1     NA's   :1    
F3FFQ96amount F3FFQ97amount   F3numitems     F3sumprot1     F3sumpveg1 
Min.   : NA   Min.   : NA   Min.   : NA   Min.   : NA   Min.   : NA  
1st Qu.: NA   1st Qu.: NA   1st Qu.: NA   1st Qu.: NA   1st Qu.: NA  
Median : NA   Median : NA   Median : NA   Median : NA   Median : NA  
Mean   :NaN   Mean   :NaN   Mean   :NaN   Mean   :NaN   Mean   :NaN  
3rd Qu.: NA   3rd Qu.: NA   3rd Qu.: NA   3rd Qu.: NA   3rd Qu.: NA  
Max.   : NA   Max.   : NA   Max.   : NA   Max.   : NA   Max.   : NA  
NA's   :1     NA's   :1     NA's   :1     NA's   :1     NA's   :1    
  F3sumpani1    F3sumgluc1    F3sumgsuc1    F3sumgpol1    F3sumlipi1 
Min.   : NA   Min.   : NA   Min.   : NA   Min.   : NA   Min.   : NA  
1st Qu.: NA   1st Qu.: NA   1st Qu.: NA   1st Qu.: NA   1st Qu.: NA  
Median : NA   Median : NA   Median : NA   Median : NA   Median : NA  
Mean   :NaN   Mean   :NaN   Mean   :NaN   Mean   :NaN   Mean   :NaN  
3rd Qu.: NA   3rd Qu.: NA   3rd Qu.: NA   3rd Qu.: NA   3rd Qu.: NA  
Max.   : NA   Max.   : NA   Max.   : NA   Max.   : NA   Max.   : NA  
NA's   :1     NA's   :1     NA's   :1     NA's   :1     NA's   :1    
```

```
  F3sumlsat1    F3sumlmon1    F3sumlpol1    F3sumalco     F3sumtot1   
 Min.   : NA   Min.   : NA   Min.   : NA   Min.   : NA   Min.   : NA  
 1st Qu.: NA   1st Qu.: NA   1st Qu.: NA   1st Qu.: NA   1st Qu.: NA  
 Median : NA   Median : NA   Median : NA   Median : NA   Median : NA  
 Mean   :NaN   Mean   :NaN   Mean   :NaN   Mean   :NaN   Mean   :NaN  
 3rd Qu.: NA   3rd Qu.: NA   3rd Qu.: NA   3rd Qu.: NA   3rd Qu.: NA  
 Max.   : NA   Max.   : NA   Max.   : NA   Max.   : NA   Max.   : NA  
 NA's   :1     NA's   :1     NA's   :1     NA's   :1     NA's   :1    
  F3sumcalc1    F3sumfer1     F3sumret1     F3sumcaro1    F3sumvitd1  
 Min.   : NA   Min.   : NA   Min.   : NA   Min.   : NA   Min.   : NA  
 1st Qu.: NA   1st Qu.: NA   1st Qu.: NA   1st Qu.: NA   1st Qu.: NA  
 Median : NA   Median : NA   Median : NA   Median : NA   Median : NA  
 Mean   :NaN   Mean   :NaN   Mean   :NaN   Mean   :NaN   Mean   :NaN  
 3rd Qu.: NA   3rd Qu.: NA   3rd Qu.: NA   3rd Qu.: NA   3rd Qu.: NA  
 Max.   : NA   Max.   : NA   Max.   : NA   Max.   : NA   Max.   : NA  
 NA's   :1     NA's   :1     NA's   :1     NA's   :1     NA's   :1    
  F3sumfibr1    F3sumchol1    F3sumprot3    F3sumpveg3    F3sumpani3  
 Min.   : NA   Min.   : NA   Min.   : NA   Min.   : NA   Min.   : NA  
 1st Qu.: NA   1st Qu.: NA   1st Qu.: NA   1st Qu.: NA   1st Qu.: NA  
 Median : NA   Median : NA   Median : NA   Median : NA   Median : NA  
 Mean   :NaN   Mean   :NaN   Mean   :NaN   Mean   :NaN   Mean   :NaN  
 3rd Qu.: NA   3rd Qu.: NA   3rd Qu.: NA   3rd Qu.: NA   3rd Qu.: NA  
 Max.   : NA   Max.   : NA   Max.   : NA   Max.   : NA   Max.   : NA  
 NA's   :1     NA's   :1     NA's   :1     NA's   :1     NA's   :1    
  F3sumgluc3    F3sumgsuc3    F3sumgpol3    F3sumlipi3    F3sumlsat3  
 Min.   : NA   Min.   : NA   Min.   : NA   Min.   : NA   Min.   : NA  
 1st Qu.: NA   1st Qu.: NA   1st Qu.: NA   1st Qu.: NA   1st Qu.: NA  
 Median : NA   Median : NA   Median : NA   Median : NA   Median : NA  
 Mean   :NaN   Mean   :NaN   Mean   :NaN   Mean   :NaN   Mean   :NaN  
 3rd Qu.: NA   3rd Qu.: NA   3rd Qu.: NA   3rd Qu.: NA   3rd Qu.: NA  
 Max.   : NA   Max.   : NA   Max.   : NA   Max.   : NA   Max.   : NA  
 NA's   :1     NA's   :1     NA's   :1     NA's   :1     NA's   :1    
  F3sumlmon3    F3sumlpol3    F3sumtot3     F3sumcalc3    F3sumfer3   
 Min.   : NA   Min.   : NA   Min.   : NA   Min.   : NA   Min.   : NA  
 1st Qu.: NA   1st Qu.: NA   1st Qu.: NA   1st Qu.: NA   1st Qu.: NA  
 Median : NA   Median : NA   Median : NA   Median : NA   Median : NA  
 Mean   :NaN   Mean   :NaN   Mean   :NaN   Mean   :NaN   Mean   :NaN  
 3rd Qu.: NA   3rd Qu.: NA   3rd Qu.: NA   3rd Qu.: NA   3rd Qu.: NA  
 Max.   : NA   Max.   : NA   Max.   : NA   Max.   : NA   Max.   : NA  
 NA's   :1     NA's   :1     NA's   :1     NA's   :1     NA's   :1    
  F3sumret3     F3sumcaro3    F3sumvitd3    F3sumfibr3    F3sumchol3  
 Min.   : NA   Min.   : NA   Min.   : NA   Min.   : NA   Min.   : NA  
 1st Qu.: NA   1st Qu.: NA   1st Qu.: NA   1st Qu.: NA   1st Qu.: NA  
 Median : NA   Median : NA   Median : NA   Median : NA   Median : NA  
 Mean   :NaN   Mean   :NaN   Mean   :NaN   Mean   :NaN   Mean   :NaN  
 3rd Qu.: NA   3rd Qu.: NA   3rd Qu.: NA   3rd Qu.: NA   3rd Qu.: NA  
 Max.   : NA   Max.   : NA   Max.   : NA   Max.   : NA   Max.   : NA  
 NA's   :1     NA's   :1     NA's   :1     NA's   :1     NA's   :1    
  F3sumeau1    F3pct_prot1   F3pct_pveg1   F3pct_pani1   F3pct_gluc1 
 Min.   : NA   Min.   : NA   Min.   : NA   Min.   : NA   Min.   : NA  
 1st Qu.: NA   1st Qu.: NA   1st Qu.: NA   1st Qu.: NA   1st Qu.: NA  
 Median : NA   Median : NA   Median : NA   Median : NA   Median : NA  
 Mean   :NaN   Mean   :NaN   Mean   :NaN   Mean   :NaN   Mean   :NaN  
 3rd Qu.: NA   3rd Qu.: NA   3rd Qu.: NA   3rd Qu.: NA   3rd Qu.: NA  
 Max.   : NA   Max.   : NA   Max.   : NA   Max.   : NA   Max.   : NA  
 NA's   :1     NA's   :1     NA's   :1     NA's   :1     NA's   :1    
 F3pct_gsuc1   F3pct_gpol1   F3pct_lipi1   F3pct_lsat1   F3pct_lmon1 
 Min.   : NA   Min.   : NA   Min.   : NA   Min.   : NA   Min.   : NA  
 1st Qu.: NA   1st Qu.: NA   1st Qu.: NA   1st Qu.: NA   1st Qu.: NA  
 Median : NA   Median : NA   Median : NA   Median : NA   Median : NA  
 Mean   :NaN   Mean   :NaN   Mean   :NaN   Mean   :NaN   Mean   :NaN  
 3rd Qu.: NA   3rd Qu.: NA   3rd Qu.: NA   3rd Qu.: NA   3rd Qu.: NA  
 Max.   : NA   Max.   : NA   Max.   : NA   Max.   : NA   Max.   : NA  
 NA's   :1     NA's   :1     NA's   :1     NA's   :1     NA's   :1    
```

```
  F3pct_lpol1   F3pct_fibr1   F3pct_chol1   F3pct_alco1   F3pct_prot3 
 Min.   : NA   Min.   : NA   Min.   : NA   Min.   : NA   Min.   : NA  
 1st Qu.: NA   1st Qu.: NA   1st Qu.: NA   1st Qu.: NA   1st Qu.: NA  
 Median : NA   Median : NA   Median : NA   Median : NA   Median : NA  
 Mean   :NaN   Mean   :NaN   Mean   :NaN   Mean   :NaN   Mean   :NaN  
 3rd Qu.: NA   3rd Qu.: NA   3rd Qu.: NA   3rd Qu.: NA   3rd Qu.: NA  
 Max.   : NA   Max.   : NA   Max.   : NA   Max.   : NA   Max.   : NA  
 NA's   :1     NA's   :1     NA's   :1     NA's   :1     NA's   :1    
  F3pct_pveg3   F3pct_pani3   F3pct_gluc3   F3pct_gsuc3   F3pct_gpol3 
 Min.   : NA   Min.   : NA   Min.   : NA   Min.   : NA   Min.   : NA  
 1st Qu.: NA   1st Qu.: NA   1st Qu.: NA   1st Qu.: NA   1st Qu.: NA  
 Median : NA   Median : NA   Median : NA   Median : NA   Median : NA  
 Mean   :NaN   Mean   :NaN   Mean   :NaN   Mean   :NaN   Mean   :NaN  
 3rd Qu.: NA   3rd Qu.: NA   3rd Qu.: NA   3rd Qu.: NA   3rd Qu.: NA  
 Max.   : NA   Max.   : NA   Max.   : NA   Max.   : NA   Max.   : NA  
 NA's   :1     NA's   :1     NA's   :1     NA's   :1     NA's   :1    
  F3pct_lipi3   F3pct_lsat3   F3pct_lmon3   F3pct_lpol3   F3pct_fibr3 
 Min.   : NA   Min.   : NA   Min.   : NA   Min.   : NA   Min.   : NA  
 1st Qu.: NA   1st Qu.: NA   1st Qu.: NA   1st Qu.: NA   1st Qu.: NA  
 Median : NA   Median : NA   Median : NA   Median : NA   Median : NA  
 Mean   :NaN   Mean   :NaN   Mean   :NaN   Mean   :NaN   Mean   :NaN  
 3rd Qu.: NA   3rd Qu.: NA   3rd Qu.: NA   3rd Qu.: NA   3rd Qu.: NA  
 Max.   : NA   Max.   : NA   Max.   : NA   Max.   : NA   Max.   : NA  
 NA's   :1     NA's   :1     NA's   :1     NA's   :1     NA's   :1    
  F3pct_chol3     F3Fruits     F3Fruits_OK   F3Vegetables F3Vegetables_OK
 Min.   : NA   Min.   : NA   Min.   : NA   Min.   : NA   Min.   : NA   
 1st Qu.: NA   1st Qu.: NA   1st Qu.: NA   1st Qu.: NA   1st Qu.: NA   
 Median : NA   Median : NA   Median : NA   Median : NA   Median : NA   
 Mean   :NaN   Mean   :NaN   Mean   :NaN   Mean   :NaN   Mean   :NaN   
 3rd Qu.: NA   3rd Qu.: NA   3rd Qu.: NA   3rd Qu.: NA   3rd Qu.: NA   
 Max.   : NA   Max.   : NA   Max.   : NA   Max.   : NA   Max.   : NA   
 NA's   :1     NA's   :1     NA's   :1     NA's   :1     NA's   :1     
     F3Meat      F3Meat_OK       F3Fish       F3Fish_OK      F3Fish2   
 Min.   : NA   Min.   : NA   Min.   : NA   Min.   : NA   Min.   : NA  
 1st Qu.: NA   1st Qu.: NA   1st Qu.: NA   1st Qu.: NA   1st Qu.: NA  
 Median : NA   Median : NA   Median : NA   Median : NA   Median : NA  
 Mean   :NaN   Mean   :NaN   Mean   :NaN   Mean   :NaN   Mean   :NaN  
 3rd Qu.: NA   3rd Qu.: NA   3rd Qu.: NA   3rd Qu.: NA   3rd Qu.: NA  
 Max.   : NA   Max.   : NA   Max.   : NA   Max.   : NA   Max.   : NA  
 NA's   :1     NA's   :1     NA's   :1     NA's   :1     NA's   :1    
   F3Fish2_OK     F3Dairy      F3Dairy_OK     F3Nb_OK       F3Cat_OK  
 Min.   : NA   Min.   : NA   Min.   : NA   Min.   : NA   Min.   : NA  
 1st Qu.: NA   1st Qu.: NA   1st Qu.: NA   1st Qu.: NA   1st Qu.: NA  
 Median : NA   Median : NA   Median : NA   Median : NA   Median : NA  
 Mean   :NaN   Mean   :NaN   Mean   :NaN   Mean   :NaN   Mean   :NaN  
 3rd Qu.: NA   3rd Qu.: NA   3rd Qu.: NA   3rd Qu.: NA   3rd Qu.: NA  
 Max.   : NA   Max.   : NA   Max.   : NA   Max.   : NA   Max.   : NA  
 NA's   :1     NA's   :1     NA's   :1     NA's   :1     NA's   :1    
   F3Recom_OK     F3Nb2_OK     F3Cat2_OK    F3Recom2_OK    F3Mediter1  
 Min.   : NA   Min.   : NA   Min.   : NA   Min.   : NA   Min.   : NA  
 1st Qu.: NA   1st Qu.: NA   1st Qu.: NA   1st Qu.: NA   1st Qu.: NA  
 Median : NA   Median : NA   Median : NA   Median : NA   Median : NA  
 Mean   :NaN   Mean   :NaN   Mean   :NaN   Mean   :NaN   Mean   :NaN  
 3rd Qu.: NA   3rd Qu.: NA   3rd Qu.: NA   3rd Qu.: NA   3rd Qu.: NA  
 Max.   : NA   Max.   : NA   Max.   : NA   Max.   : NA   Max.   : NA  
 NA's   :1     NA's   :1     NA's   :1     NA's   :1     NA's   :1    
   F3Mediter2     F3AHEI1       F3AHEI2     F3vegetarian      FAM       
 Min.   : NA   Min.   : NA   Min.   : NA   Min.   :0     Min.   :56176  
 1st Qu.: NA   1st Qu.: NA   1st Qu.: NA   1st Qu.:0     1st Qu.:56176  
 Median : NA   Median : NA   Median : NA   Median :0     Median :56176  
 Mean   :NaN   Mean   :NaN   Mean   :NaN   Mean   :0     Mean   :56176  
 3rd Qu.: NA   3rd Qu.: NA   3rd Qu.: NA   3rd Qu.:0     3rd Qu.:56176  
 Max.   : NA   Max.   : NA   Max.   : NA   Max.   :0     Max.   :56176  
 NA's   :1     NA's   :1     NA's   :1                                  
```

```
     male    edtyp4_new          F0_mmesc          F0_CL_age        
 Min.   :1   Length:1           Length:1           Length:1          
 1st Qu.:1   Class :character   Class :character   Class :character  
 Median :1   Mode  :character   Mode  :character   Mode  :character  
 Mean   :1                                                            
 3rd Qu.:1                                                            
 Max.   :1                                                            

  mmesc_fup0          F1_mmesc           F1_CL_age          mmesc_fup1       
 Length:1           Length:1           Length:1           Length:1          
 Class :character   Class :character   Class :character   Class :character  
 Mode  :character   Mode  :character   Mode  :character   Mode  :character  




    F1_Age          F1_cur_mddpd2        F1_QPC_A    F1_QPC_B    F1_QPC_1
 Length:1           Length:1           Min.   :0   Min.   :0   Min.   :0  
 Class :character   Class :character   1st Qu.:0   1st Qu.:0   1st Qu.:0  
 Mode  :character   Mode  :character   Median :0   Median :0   Median :0  
                                       Mean   :0   Mean   :0   Mean   :0  
                                       3rd Qu.:0   3rd Qu.:0   3rd Qu.:0  
                                       Max.   :0   Max.   :0   Max.   :0  

    F1_QPC_2    F1_QPC_3    F1_QPC_4    F1_QPC_5    F1_QPC_6    F1_QPC_7
 Min.   :0   Min.   :0   Min.   :0   Min.   :0   Min.   :0   Min.   :0  
 1st Qu.:0   1st Qu.:0   1st Qu.:0   1st Qu.:0   1st Qu.:0   1st Qu.:0  
 Median :0   Median :0   Median :0   Median :0   Median :0   Median :0  
 Mean   :0   Mean   :0   Mean   :0   Mean   :0   Mean   :0   Mean   :0  
 3rd Qu.:0   3rd Qu.:0   3rd Qu.:0   3rd Qu.:0   3rd Qu.:0   3rd Qu.:0  
 Max.   :0   Max.   :0   Max.   :0   Max.   :0   Max.   :0   Max.   :0  

    F1_QPC_8    CDR_b              verbal_b  F1_FV_FA_TOT      
 Min.   :0   Length:1           Min.   :34   Length:1          
 1st Qu.:0   Class :character   1st Qu.:34   Class :character  
 Median :0   Mode  :character   Median :34   Mode  :character  
 Mean   :0                      Mean   :34                     
 3rd Qu.:0                      3rd Qu.:34                     
 Max.   :0                      Max.   :34                     

 F1_FV_P_TOT         F1_B16_ID          F1_B16_RI          F1_B16_S1RL       
 Length:1           Length:1           Length:1           Length:1          
 Class :character   Class :character   Class :character   Class :character  
 Mode  :character   Mode  :character   Mode  :character   Mode  :character  




 F1_B16_S1RI        F1_B16_S2RL        F1_B16_S2RI        F1_B16_S3RL       
 Length:1           Length:1           Length:1           Length:1          
 Class :character   Class :character   Class :character   Class :character  
 Mode  :character   Mode  :character   Mode  :character   Mode  :character  




 F1_B16_S3RI        F1_B16_EDRL        F1_B16_EDRI           memory_b 
 Length:1           Length:1           Length:1           Min.   :48  
 Class :character   Class :character   Class :character   1st Qu.:48  
 Mode  :character   Mode  :character   Mode  :character   Median :48  
                                                          Mean   :48  
                                                          3rd Qu.:48  
                                                          Max.   :48  
```

```
F1_STROOP_D        F1_STROOP_D_T      F1_STROOP_IFAIBLE  F1_STROOP_IFAIBLE_T
Length:1           Length:1           Length:1           Length:1
Class :character   Class :character   Class :character   Class :character
Mode  :character   Mode  :character   Mode  :character   Mode  :character




   stroop_b  F1_STROOP_IFORTE_T     do40_b   F1_CERAD_CERCLE
Min.   :24   Length:1           Min.   :40   Length:1
1st Qu.:24   Class :character   1st Qu.:40   Class :character
Median :24   Mode  :character   Median :40   Mode  :character
Mean   :24                      Mean   :40
3rd Qu.:24                      3rd Qu.:40
Max.   :24                      Max.   :40

F1_CERAD_RECTANLES F1_CERAD_LOSANGE   F1_CERAD_CUBE         CERAD_b
Length:1           Length:1           Length:1           Min.   :10
Class :character   Class :character   Class :character   1st Qu.:10
Mode  :character   Mode  :character   Mode  :character   Median :10
                                                         Mean   :10
                                                         3rd Qu.:10
                                                         Max.   :10

cognn_psy_fup1       F2_mmesc          F2_CL_age          mmesc_fup2
Length:1           Length:1           Length:1           Length:1
Class :character   Class :character   Class :character   Class :character
Mode  :character   Mode  :character   Mode  :character   Mode  :character




   F2_Age          F2_cur_mddpd2         F2_QPC_A      F2_QPC_B
Length:1           Length:1           Min.   : NA   Min.   : NA
Class :character   Class :character   1st Qu.: NA   1st Qu.: NA
Mode  :character   Mode  :character   Median : NA   Median : NA
                                      Mean   :NaN   Mean   :NaN
                                      3rd Qu.: NA   3rd Qu.: NA
                                      Max.   : NA   Max.   : NA
                                      NA's   :1     NA's   :1
   F2_QPC_1      F2_QPC_2      F2_QPC_3      F2_QPC_4      F2_QPC_5
Min.   : NA   Min.   : NA   Min.   : NA   Min.   : NA   Min.   : NA
1st Qu.: NA   1st Qu.: NA   1st Qu.: NA   1st Qu.: NA   1st Qu.: NA
Median : NA   Median : NA   Median : NA   Median : NA   Median : NA
Mean   :NaN   Mean   :NaN   Mean   :NaN   Mean   :NaN   Mean   :NaN
3rd Qu.: NA   3rd Qu.: NA   3rd Qu.: NA   3rd Qu.: NA   3rd Qu.: NA
Max.   : NA   Max.   : NA   Max.   : NA   Max.   : NA   Max.   : NA
NA's   :1     NA's   :1     NA's   :1     NA's   :1     NA's   :1
   F2_QPC_6      F2_QPC_7      F2_QPC_8      F2CDR                F2verbal
Min.   : NA   Min.   : NA   Min.   : NA   Length:1           Min.   : NA
1st Qu.: NA   1st Qu.: NA   1st Qu.: NA   Class :character   1st Qu.: NA
Median : NA   Median : NA   Median : NA   Mode  :character   Median : NA
Mean   :NaN   Mean   :NaN   Mean   :NaN                      Mean   :NaN
3rd Qu.: NA   3rd Qu.: NA   3rd Qu.: NA                      3rd Qu.: NA
Max.   : NA   Max.   : NA   Max.   : NA                      Max.   : NA
NA's   :1     NA's   :1     NA's   :1                        NA's   :1
F2_FV_FA_TOT       F2_FV_P_TOT         F2_B16_ID          F2_B16_RI
Length:1           Length:1           Length:1           Length:1
Class :character   Class :character   Class :character   Class :character
Mode  :character   Mode  :character   Mode  :character   Mode  :character
```

```
 F2_B16_S1RL        F2_B16_S1RI        F2_B16_S2RL        F2_B16_S2RI       
 Length:1           Length:1           Length:1           Length:1          
 Class :character   Class :character   Class :character   Class :character  
 Mode  :character   Mode  :character   Mode  :character   Mode  :character  
                                                                            
                                                                            
                                                                            
 F2_B16_S3RL        F2_B16_S3RI        F2_B16_EDRL        F2_B16_EDRI       
 Length:1           Length:1           Length:1           Length:1          
 Class :character   Class :character   Class :character   Class :character  
 Mode  :character   Mode  :character   Mode  :character   Mode  :character  
                                                                            
                                                                            
                                                                            
    F2memory   F2_STROOP_D        F2_STROOP_D_T      F2_STROOP_IFAIBLE 
 Min.   : NA   Length:1           Length:1           Length:1          
 1st Qu.: NA   Class :character   Class :character   Class :character  
 Median : NA   Mode  :character   Mode  :character   Mode  :character  
 Mean   :NaN                                                           
 3rd Qu.: NA                                                           
 Max.   : NA                                                           
 NA's   :1                                                             
 F2_STROOP_IFAIBLE_T    F2stroop   F2_STROOP_IFORTE_T     F2do40    
 Length:1            Min.   : NA   Length:1           Min.   : NA  
 Class :character    1st Qu.: NA   Class :character   1st Qu.: NA  
 Mode  :character    Median : NA   Mode  :character   Median : NA  
                     Mean   :NaN                      Mean   :NaN  
                     3rd Qu.: NA                      3rd Qu.: NA  
                     Max.   : NA                      Max.   : NA  
                     NA's   :1                        NA's   :1    
 F2_CERAD_CERCLE    F2_CERAD_RECTANLES F2_CERAD_LOSANGE   F2_CERAD_CUBE     
 Length:1           Length:1           Length:1           Length:1          
 Class :character   Class :character   Class :character   Class :character  
 Mode  :character   Mode  :character   Mode  :character   Mode  :character  
                                                                            
                                                                            
                                                                            
    F2CERAD    cogn_psy_fup2        F3_mmesc           F3_CL_age        
 Min.   : NA   Length:1           Length:1           Length:1          
 1st Qu.: NA   Class :character   Class :character   Class :character  
 Median : NA   Mode  :character   Mode  :character   Mode  :character  
 Mean   :NaN                                                           
 3rd Qu.: NA                                                           
 Max.   : NA                                                           
 NA's   :1                                                             
  mmesc_fup3           F3_Age          F3_cur_mddpd2         F3_QPC_A   
 Length:1           Length:1           Length:1           Min.   : NA  
 Class :character   Class :character   Class :character   1st Qu.: NA  
 Mode  :character   Mode  :character   Mode  :character   Median : NA  
                                                          Mean   :NaN  
                                                          3rd Qu.: NA  
                                                          Max.   : NA  
                                                          NA's   :1    
    F3_QPC_B      F3_QPC_1      F3_QPC_2      F3_QPC_3      F3_QPC_4   
 Min.   : NA   Min.   : NA   Min.   : NA   Min.   : NA   Min.   : NA  
 1st Qu.: NA   1st Qu.: NA   1st Qu.: NA   1st Qu.: NA   1st Qu.: NA  
 Median : NA   Median : NA   Median : NA   Median : NA   Median : NA  
 Mean   :NaN   Mean   :NaN   Mean   :NaN   Mean   :NaN   Mean   :NaN  
 3rd Qu.: NA   3rd Qu.: NA   3rd Qu.: NA   3rd Qu.: NA   3rd Qu.: NA  
 Max.   : NA   Max.   : NA   Max.   : NA   Max.   : NA   Max.   : NA  
 NA's   :1     NA's   :1     NA's   :1     NA's   :1     NA's   :1    
```

```
   F3_QPC_5      F3_QPC_6      F3_QPC_7      F3_QPC_8      F3CDR          
 Min.   : NA   Min.   : NA   Min.   : NA   Min.   : NA   Length:1          
 1st Qu.: NA   1st Qu.: NA   1st Qu.: NA   1st Qu.: NA   Class :character  
 Median : NA   Median : NA   Median : NA   Median : NA   Mode  :character  
 Mean   :NaN   Mean   :NaN   Mean   :NaN   Mean   :NaN                     
 3rd Qu.: NA   3rd Qu.: NA   3rd Qu.: NA   3rd Qu.: NA                     
 Max.   : NA   Max.   : NA   Max.   : NA   Max.   : NA                     
 NA's   :1     NA's   :1     NA's   :1     NA's   :1                       
   F3verbal    F3_FV_FA_TOT       F3_FV_P_TOT         F3_B16_ID        
 Min.   : NA   Length:1           Length:1           Length:1          
 1st Qu.: NA   Class :character   Class :character   Class :character  
 Median : NA   Mode  :character   Mode  :character   Mode  :character  
 Mean   :NaN                                                           
 3rd Qu.: NA                                                           
 Max.   : NA                                                           
 NA's   :1                                                             
  F3_B16_RI          F3_B16_S1RL        F3_B16_S1RI        F3_B16_S2RL       
 Length:1           Length:1           Length:1           Length:1          
 Class :character   Class :character   Class :character   Class :character  
 Mode  :character   Mode  :character   Mode  :character   Mode  :character  
                                                                            
                                                                            
                                                                            
                                                                            
 F3_B16_S2RI        F3_B16_S3RL        F3_B16_S3RI        F3_B16_EDRL       
 Length:1           Length:1           Length:1           Length:1          
 Class :character   Class :character   Class :character   Class :character  
 Mode  :character   Mode  :character   Mode  :character   Mode  :character  
                                                                            
                                                                            
                                                                            
                                                                            
 F3_B16_EDRI           F3memory   F3_STROOP_D        F3_STROOP_D_T     
 Length:1           Min.   : NA   Length:1           Length:1          
 Class :character   1st Qu.: NA   Class :character   Class :character  
 Mode  :character   Median : NA   Mode  :character   Mode  :character  
                    Mean   :NaN                                        
                    3rd Qu.: NA                                        
                    Max.   : NA                                        
                    NA's   :1                                          
 F3_STROOP_IFAIBLE  F3_STROOP_IFAIBLE_T    F3stroop    F3_STROOP_IFORTE_T
 Length:1           Length:1            Min.   : NA   Length:1          
 Class :character   Class :character    1st Qu.: NA   Class :character  
 Mode  :character   Mode  :character    Median : NA   Mode  :character  
                                        Mean   :NaN                     
                                        3rd Qu.: NA                     
                                        Max.   : NA                     
                                        NA's   :1                       
     F3do40    F3_CERAD_CERCLE    F3_CERAD_RECTANLES F3_CERAD_LOSANGE  
 Min.   : NA   Length:1           Length:1           Length:1          
 1st Qu.: NA   Class :character   Class :character   Class :character  
 Median : NA   Mode  :character   Mode  :character   Mode  :character  
 Mean   :NaN                                                           
 3rd Qu.: NA                                                           
 Max.   : NA                                                           
 NA's   :1                                                             
 F3_CERAD_CUBE         F3CERAD    cogn_psy_fup3                edu    bmi_cat
 Length:1           Min.   : NA   Length:1           Elementary :0   1:0    
 Class :character   1st Qu.: NA   Class :character   High school:0   2:1    
 Mode  :character   Median : NA   Mode  :character   Superior   :1   3:0    
                    Mean   :NaN                                             
                    3rd Qu.: NA                                             
                    Max.   : NA                                             
                    NA's   :1                                               
```

```
  age_recruit     age_b      age_cat   cvevent_b     HTA_b        alc_b   
 Min.   :65   Min.   :70   1:0     Min.   :0   Min.   :1   Min.   :0  
 1st Qu.:65   1st Qu.:70   2:1     1st Qu.:0   1st Qu.:1   1st Qu.:0  
 Median :65   Median :70   3:0     Median :0   Median :1   Median :0  
 Mean   :65   Mean   :70           Mean   :0   Mean   :1   Mean   :0  
 3rd Qu.:65   3rd Qu.:70           3rd Qu.:0   3rd Qu.:1   3rd Qu.:0  
 Max.   :65   Max.   :70           Max.   :0   Max.   :1   Max.   :0  
                                                                    
     pa_b              futime_b      futime_F1     futime_F2         ltfu  
 Length:1           Min.   :5.28   Min.   :5.6   Min.   :9.03   Min.   :0  
 Class :character   1st Qu.:5.28   1st Qu.:5.6   1st Qu.:9.03   1st Qu.:0  
 Mode  :character   Median :5.28   Median :5.6   Median :9.03   Median :0  
                    Mean   :5.28   Mean   :5.6   Mean   :9.03   Mean   :0  
                    3rd Qu.:5.28   3rd Qu.:5.6   3rd Qu.:9.03   3rd Qu.:0  
                    Max.   :5.28   Max.   :5.6   Max.   :9.03   Max.   :0  
                                                                           
     SCD_b       F2SCD         F3SCD        SCD_b_yn    F2SCD_yn  
 Min.   :0   Min.   : NA   Min.   : NA   Min.   :0   Min.   : NA  
 1st Qu.:0   1st Qu.: NA   1st Qu.: NA   1st Qu.:0   1st Qu.: NA  
 Median :0   Median : NA   Median : NA   Median :0   Median : NA  
 Mean   :0   Mean   :NaN   Mean   :NaN   Mean   :0   Mean   :NaN  
 3rd Qu.:0   3rd Qu.: NA   3rd Qu.: NA   3rd Qu.:0   3rd Qu.: NA  
 Max.   :0   Max.   : NA   Max.   : NA   Max.   :0   Max.   : NA  
             NA's   :1     NA's   :1                 NA's   :1    
   F3SCD_yn    memory_b_cat  verbal_b_cat  stroop_b_cat   do40_b_cat
 Min.   : NA   Min.   :0     Min.   :0     Min.   :0     Min.   :0  
 1st Qu.: NA   1st Qu.:0     1st Qu.:0     1st Qu.:0     1st Qu.:0  
 Median : NA   Median :0     Median :0     Median :0     Median :0  
 Mean   :NaN   Mean   :0     Mean   :0     Mean   :0     Mean   :0  
 3rd Qu.: NA   3rd Qu.:0     3rd Qu.:0     3rd Qu.:0     3rd Qu.:0  
 Max.   : NA   Max.   :0     Max.   :0     Max.   :0     Max.   :0  
 NA's   :1                                                          
  CERAD_b_cat  F2memory_cat  F2verbal_cat  F2stroop_cat   F2do40_cat 
 Min.   :1    Min.   : NA   Min.   : NA   Min.   : NA   Min.   : NA  
 1st Qu.:1    1st Qu.: NA   1st Qu.: NA   1st Qu.: NA   1st Qu.: NA  
 Median :1    Median : NA   Median : NA   Median : NA   Median : NA  
 Mean   :1    Mean   :NaN   Mean   :NaN   Mean   :NaN   Mean   :NaN  
 3rd Qu.:1    3rd Qu.: NA   3rd Qu.: NA   3rd Qu.: NA   3rd Qu.: NA  
 Max.   :1    Max.   : NA   Max.   : NA   Max.   : NA   Max.   : NA  
              NA's   :1     NA's   :1     NA's   :1     NA's   :1    
  F2CERAD_cat   F3memory_cat  F3verbal_cat  F3stroop_cat   F3do40_cat 
 Min.   : NA   Min.   : NA   Min.   : NA   Min.   : NA   Min.   : NA  
 1st Qu.: NA   1st Qu.: NA   1st Qu.: NA   1st Qu.: NA   1st Qu.: NA  
 Median : NA   Median : NA   Median : NA   Median : NA   Median : NA  
 Mean   :NaN   Mean   :NaN   Mean   :NaN   Mean   :NaN   Mean   :NaN  
 3rd Qu.: NA   3rd Qu.: NA   3rd Qu.: NA   3rd Qu.: NA   3rd Qu.: NA  
 Max.   : NA   Max.   : NA   Max.   : NA   Max.   : NA   Max.   : NA  
 NA's   :1     NA's   :1     NA's   :1     NA's   :1     NA's   :1    
  F3CERAD_cat  total_dairy_b   ferm_dairy_b  nonferm_dairy_b fullfat_dairy_b
 Min.   : NA   Min.   :1698   Min.   :1652   Min.   :46.43   Min.   :989.3  
 1st Qu.: NA   1st Qu.:1698   1st Qu.:1652   1st Qu.:46.43   1st Qu.:989.3  
 Median : NA   Median :1698   Median :1652   Median :46.43   Median :989.3  
 Mean   :NaN   Mean   :1698   Mean   :1652   Mean   :46.43   Mean   :989.3  
 3rd Qu.: NA   3rd Qu.:1698   3rd Qu.:1652   3rd Qu.:46.43   3rd Qu.:989.3  
 Max.   : NA   Max.   :1698   Max.   :1652   Max.   :46.43   Max.   :989.3  
 NA's   :1                                                                  
 nonfat_dairy_b  sugar_dairy_b   nonsug_dairy_b F2total_dairy  F2ferm_dairy 
 Min.   :708.9   Min.   :679.3   Min.   :1019   Min.   : NA   Min.   : NA  
 1st Qu.:708.9   1st Qu.:679.3   1st Qu.:1019   1st Qu.: NA   1st Qu.: NA  
 Median :708.9   Median :679.3   Median :1019   Median : NA   Median : NA  
 Mean   :708.9   Mean   :679.3   Mean   :1019   Mean   :NaN   Mean   :NaN  
 3rd Qu.:708.9   3rd Qu.:679.3   3rd Qu.:1019   3rd Qu.: NA   3rd Qu.: NA  
 Max.   :708.9   Max.   :679.3   Max.   :1019   Max.   : NA   Max.   : NA  
                                                NA's   :1     NA's   :1    
```

```
 F2nonferm_dairy F2fullfat_dairy F2nonfat_dairy F2sugar_dairy F3total_dairy
 Min.   : NA     Min.   : NA     Min.   : NA    Min.   : NA   Min.   : NA  
 1st Qu.: NA     1st Qu.: NA     1st Qu.: NA    1st Qu.: NA   1st Qu.: NA  
 Median : NA     Median : NA     Median : NA    Median : NA   Median : NA  
 Mean   :NaN     Mean   :NaN     Mean   :NaN    Mean   :NaN   Mean   :NaN  
 3rd Qu.: NA     3rd Qu.: NA     3rd Qu.: NA    3rd Qu.: NA   3rd Qu.: NA  
 Max.   : NA     Max.   : NA     Max.   : NA    Max.   : NA   Max.   : NA  
 NA's   :1       NA's   :1       NA's   :1      NA's   :1     NA's   :1    
  F3ferm_dairy F3nonferm_dairy F3fullfat_dairy F3nonfat_dairy F3sugar_dairy
 Min.   : NA   Min.   : NA     Min.   : NA     Min.   : NA    Min.   : NA  
 1st Qu.: NA   1st Qu.: NA     1st Qu.: NA     1st Qu.: NA    1st Qu.: NA  
 Median : NA   Median : NA     Median : NA     Median : NA    Median : NA  
 Mean   :NaN   Mean   :NaN     Mean   :NaN     Mean   :NaN    Mean   :NaN  
 3rd Qu.: NA   3rd Qu.: NA     3rd Qu.: NA     3rd Qu.: NA    3rd Qu.: NA  
 Max.   : NA   Max.   : NA     Max.   : NA     Max.   : NA    Max.   : NA  
 NA's   :1     NA's   :1       NA's   :1       NA's   :1      NA's   :1    
   Min. 1st Qu.  Median    Mean 3rd Qu.    Max. 
   0.00   67.32  128.39  164.08  214.29 1651.79 
   Min. 1st Qu.  Median    Mean 3rd Qu.    Max. 
   0.00   15.89   44.93   82.05  104.46  964.84 
   Min. 1st Qu.  Median    Mean 3rd Qu.    Max. 
   0.00   93.75  172.32  207.62  268.39 1418.93 
   Min. 1st Qu.  Median    Mean 3rd Qu.    Max. 
   0.00    0.00    0.00   38.51   37.50  932.14 
   Min. 1st Qu.  Median    Mean 3rd Qu.    Max. 
  0.000   7.857  32.143  66.017  90.000 712.500 
```

In [145…

```
ch_sc <- mutate(ch_sc,nonfat_dairy_b_b= ifelse(is.na(nonfat_dairy_b), NA,
                                          ifelse(nonfat_dairy_b == 0, 0, 1)),
                    F2nonfat_dairy_b= ifelse(is.na(F2nonfat_dairy), NA,
                                          ifelse(F2nonfat_dairy == 0, 0, 1)),
                    F3nonfat_dairy_b= ifelse(is.na(F3nonfat_dairy), NA,
                                          ifelse(F3nonfat_dairy == 0, 0, 1)),
                    total_dairy_avg = (total_dairy_b+F2total_dairy)/2,
                    ferm_dairy_avg = (ferm_dairy_b+F2ferm_dairy)/2,
                    nonferm_dairy_avg=(nonferm_dairy_b+F2nonferm_dairy)/2,
                    fullfat_dairy_avg = (fullfat_dairy_b+F2fullfat_dairy)/2,
                    nonfat_dairy_avg = (nonfat_dairy_b+F2nonfat_dairy)/2,
                    sugar_dairy_avg=(sugar_dairy_b+F2sugar_dairy)/2)


ch_sc <- ch_sc %>%
        mutate(total_dairy_freq_b = F1freqFFQ1+F1freqFFQ2+F1freqFFQ3+F1freqFFQ4+F1
                    F1freqFFQ8+F1freqFFQ52+F1freqFFQ53+F1freqFFQ71+F1freqFFQ68+F1f
                    F1freqFFQ84+F1freqFFQ85+F1freqFFQ86+F1freqFFQ63,
                ferm_dairy_freq_b = F1freqFFQ1+F1freqFFQ2+F1freqFFQ3+F1freqFFQ4+F1
                    F1freqFFQ8,
                nonferm_dairy_freq_b = F1freqFFQ52+F1freqFFQ53+F1freqFFQ71+F1freqF
                    F1freqFFQ84+F1freqFFQ85+F1freqFFQ86+F1freqFFQ63,
                fullfat_dairy_freq_b = F1freqFFQ1+F1freqFFQ3+F1freqFFQ5+F1freqFFQ6
                    F1freqFFQ53+F1freqFFQ71+F1freqFFQ68+F1freqFFQ83+F1freqFFQ84+F1
                nonfat_dairy_freq_b = F1freqFFQ2+F1freqFFQ4+F1freqFFQ82+F1freqFFQ8
                sugar_dairy_freq_b = F1freqFFQ3+F1freqFFQ68+F1freqFFQ63,
                F2total_dairy_freq = F2freqFFQ1+F2freqFFQ2+F2freqFFQ3+F2freqFFQ4+F
                    F2freqFFQ8+F2freqFFQ52+F2freqFFQ53+F2freqFFQ71+F2freqFFQ68+F2f
                    F2freqFFQ84+F2freqFFQ85+F2freqFFQ86+F2freqFFQ63,
                F2ferm_dairy_freq = F2freqFFQ1+F2freqFFQ2+F2freqFFQ3+F2freqFFQ4+F2
                    F2freqFFQ8,
                F2nonferm_dairy_freq = F2freqFFQ52+F2freqFFQ53+F2freqFFQ71+F2freqF
                    F2freqFFQ84+F2freqFFQ85+F2freqFFQ86+F2freqFFQ63,
                F2fullfat_dairy_freq = F2freqFFQ1+F2freqFFQ3+F2freqFFQ5+F2freqFFQ6
                    F2freqFFQ53+F2freqFFQ71+F2freqFFQ68+F2freqFFQ83+F2freqFFQ84+F2
                F2nonfat_dairy_freq = F2freqFFQ2+F2freqFFQ4+F2freqFFQ82+F2freqFFQ8
                F2sugar_dairy_freq = F2freqFFQ3 + F2freqFFQ68+F2freqFFQ63,
                F3total_dairy_freq = F3freqFFQ1+F3freqFFQ2+F3freqFFQ3+F3freqFFQ4+F
```

```
                    F3freqFFQ8+F3freqFFQ52+F3freqFFQ53+F3freqFFQ71+F3freqFFQ68+F3fr
                    F3freqFFQ84+F3freqFFQ85+F3freqFFQ86+F3freqFFQ63,
                F3ferm_dairy_freq = F3freqFFQ1+F3freqFFQ2+F3freqFFQ3+F3freqFFQ4+F3
                    F3freqFFQ8,
                F3nonferm_dairy_freq = F3freqFFQ52+F3freqFFQ53+F3freqFFQ71+F3freqFF
                    F3freqFFQ84+F3freqFFQ85+F3freqFFQ86+F3freqFFQ63,
                F3fullfat_dairy_freq = F3freqFFQ1+F3freqFFQ3+F3freqFFQ5+F3freqFFQ6
                    F3freqFFQ53+F3freqFFQ71+F3freqFFQ68+F3freqFFQ83+F3freqFFQ84+F3
                F3nonfat_dairy_freq = F3freqFFQ2+F3freqFFQ4+F3freqFFQ82+F3freqFFQ8
                F3sugar_dairy_freq = F3freqFFQ3 + F3freqFFQ68+F3freqFFQ63)
```

In [146…

```
#Calculate number serving/day in 3 categories for the baseline table

ch_sc <- ch_sc %>% mutate(total_dairy_freq_b_serv3 = ifelse(total_dairy_freq_b <=1
                                          ifelse(total_dairy_freq_b < 3, 2,
                                                  ifelse(total_dairy_freq_b >=
                          ferm_dairy_freq_b_serv3 = ifelse(ferm_dairy_freq_b <=1, 1
                                                        ifelse(ferm_dairy_freq_b <
                                                            ifelse(ferm_dairy_freq
                          nonferm_dairy_freq_b_serv3 = ifelse(nonferm_dairy_freq_b
                                          ifelse(nonferm_dairy_freq_b< 3, 2,
                                                 ifelse(nonferm_dairy_freq_b>=
                          fullfat_dairy_freq_b_serv3 = ifelse(fullfat_dairy_freq_b
                                          ifelse(fullfat_dairy_freq_b< 3, 2,
                                                 ifelse(fullfat_dairy_freq_b>=
                          nonfat_dairy_freq_b_serv3 = ifelse(nonfat_dairy_freq_b <=
                                          ifelse(nonfat_dairy_freq_b < 3, 2,
                                                 ifelse(nonfat_dairy_freq_b >=
                          sugar_dairy_freq_b_serv3 = ifelse(sugar_dairy_freq_b <=1,
                                          ifelse(sugar_dairy_freq_b < 3, 2,
                                                 ifelse(sugar_dairy_freq_b >= 3

ch_sc$total_dairy_freq_b_serv3 <- factor(ch_sc$total_dairy_freq_b_serv3, levels =
                                labels = c("Once or less than once per day","More
ch_sc$ferm_dairy_freq_b_serv3 <- factor(ch_sc$ferm_dairy_freq_b_serv3, levels = c(
                                labels = c("Once or less than once per day","More
ch_sc$nonferm_dairy_freq_b_serv3 <- factor(ch_sc$nonferm_dairy_freq_b_serv3, level
                                labels = c("Once or less than once per day","More
ch_sc$fullfat_dairy_freq_b_serv3 <- factor(ch_sc$fullfat_dairy_freq_b_serv3, level
                                labels = c("Once or less than once per day","More
ch_sc$nonfat_dairy_freq_b_serv3 <- factor(ch_sc$nonfat_dairy_freq_b_serv3, levels
                                labels = c("Once or less than once per day","More
ch_sc$sugar_dairy_freq_b_serv3 <- factor(ch_sc$sugar_dairy_freq_b_serv3, levels =
                                labels = c("Once or less than once per day","More

ggplot(ch_sc, aes(x = total_dairy_freq_b_serv3)) +
        geom_bar() +
        theme(axis.text.x = element_text(angle = 45, hjust = 1))
```

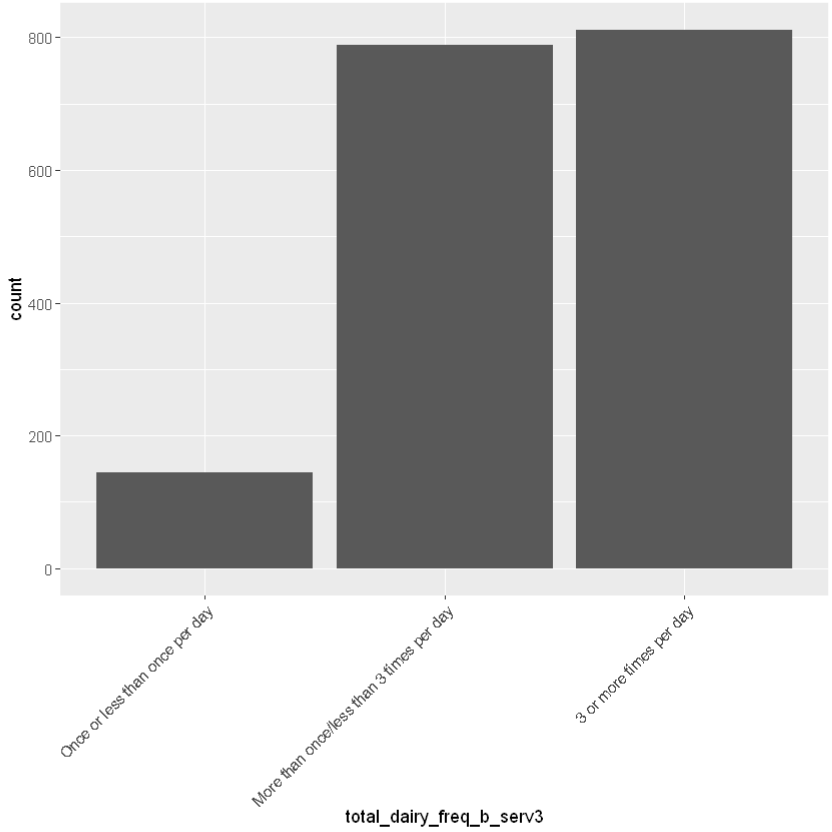

In [147…

```
# Mean and 95% CI of continuous exposures
mean(na.omit(ch_sc$total_dairy_b))
mean(na.omit(ch_sc$ferm_dairy_b))
mean(na.omit(ch_sc$nonferm_dairy_b))
mean(na.omit(ch_sc$fullfat_dairy_b))
mean(na.omit(ch_sc$nonfat_dairy_b))
mean(na.omit(ch_sc$sugar_dairy_b))

t.test(na.omit(ch_sc$total_dairy_b))$"conf.int"
t.test(na.omit(ch_sc$ferm_dairy_b))$"conf.int"
t.test(na.omit(ch_sc$nonferm_dairy_b))$"conf.int"
t.test(na.omit(ch_sc$fullfat_dairy_b))$"conf.int"
t.test(na.omit(ch_sc$nonfat_dairy_b))$"conf.int"
t.test(na.omit(ch_sc$sugar_dairy_b))$"conf.int"

mean(na.omit(ch_sc$F2total_dairy))
mean(na.omit(ch_sc$F2ferm_dairy))
mean(na.omit(ch_sc$F2nonferm_dairy))
mean(na.omit(ch_sc$F2fullfat_dairy))
mean(na.omit(ch_sc$F2nonfat_dairy))
mean(na.omit(ch_sc$F2sugar_dairy))

mean(na.omit(ch_sc$F3total_dairy))
mean(na.omit(ch_sc$F3ferm_dairy))
mean(na.omit(ch_sc$F3nonferm_dairy))
mean(na.omit(ch_sc$F3fullfat_dairy))
mean(na.omit(ch_sc$F3nonfat_dairy))
mean(na.omit(ch_sc$F3sugar_dairy))
```

246.131356155496

164.0844258149

82.046930340596

207.619536568762

38.5118195867335

66.0174994162178

237.376187489132 · 254.886524821859

157.223071241309 · 170.94578038849

76.945806719561 · 87.148053961631

199.829588654473 · 215.409484483052

34.4513196978138 · 42.5723194756532

61.2973136987034 · 70.7376851337321

250.445090966103

165.665648220086

83.1498799634399

214.294216653429

36.6110048020156

68.004752135794

236.845940091824

156.753681395471

78.8512517174936

210.093601199806

27.2583699257982

65.0383786424779

Compare if the means of total dairy consumption change over time

In [148…

```
ch_sc_assumpt_test1 <- ch_sc %>% mutate(ind_diff = total_dairy_b - F2total_dairy)
ch_sc_assumpt_test2 <- ch_sc %>% mutate(ind_diff = total_dairy_b - F3total_dairy)
ch_sc_assumpt_test3 <- ch_sc %>% mutate(ind_diff = F2total_dairy - F3total_dairy)

mean(na.omit(ch_sc_assumpt_test1$ind_diff)) #Very small individual variation from o
mean(na.omit(ch_sc_assumpt_test2$ind_diff)) #Quite a lot of variation from one time
mean(na.omit(ch_sc_assumpt_test3$ind_diff)) #Very small individual variation from o
```

-1.94772073065388

13.2547127274515

6.56456055647929

OTHER FOOD GROUPS

In [149…

```
ch_sc <- ch_sc %>%
        mutate(veg_b = F1FFQ30amount+F1FFQ31amount+F1FFQ32amount+F1FFQ33amount+F1FF
                    F1FFQ37amount+F1FFQ38amount+F1FFQ39amount+F1FFQ45amount+F1FFQ50
                fru_b  = F1FFQ55amount+F1FFQ56amount+F1FFQ57amount+F1FFQ58amount+F1F
                sea_b  = F1FFQ25amount+F1FFQ26amount+F1FFQ27amount+F1FFQ28amount+F1F
                meat_b  = F1FFQ14amount+F1FFQ17amount+F1FFQ18amount+F1FFQ23amount+F1
                    F1FFQ19amount+F1FFQ20amount+F1FFQ21amount+F1FFQ22amount,
                eggs_b  = F1FFQ49amount,
                grains_b  = F1FFQ9amount+F1FFQ10amount+F1FFQ11amount+F1FFQ12amount+F
                    F1FFQ42amount+F1FFQ43amount+F1FFQ44amount+F1FFQ46amount,
                alcohol_b  = F1FFQ94amount+F1FFQ95amount+F1FFQ96amount+F1FFQ97amount
                sugary_b  = F1FFQ61amount+F1FFQ62amount+F1FFQ64amount+F1FFQ65amount+
                    F1FFQ90amount+F1FFQ91amount+F1FFQ92amount,
                fats_b  = F1FFQ73amount+F1FFQ74amount+F1FFQ75amount+F1FFQ51amount+F1
                       F1FFQ47amount,
               nonsug_dairy_b  = F1FFQ1amount+F1FFQ2amount+F1FFQ4amount+F1FFQ5amount
                    F1FFQ8amount+F1FFQ52amount+F1FFQ53amount+F1FFQ71amount+F1FFQ82a
                    F1FFQ84amount+F1FFQ85amount+F1FFQ86amount,
```

```
                F2nonsug_dairy = F2FFQ1amount+F2FFQ2amount+F2FFQ4amount+F2FFQ5amoun
                     F2FFQ8amount+F2FFQ52amount+F2FFQ53amount+F2FFQ71amount+F2FFQ82
                     F2FFQ84amount+F2FFQ85amount+F2FFQ86amount,
                 F2veg = F2FFQ30amount+F2FFQ31amount+F2FFQ32amount+F2FFQ33amount+F2
                     F2FFQ37amount+F2FFQ38amount+F2FFQ39amount+F2FFQ45amount+F2FFQ5
                 F2fru = F2FFQ55amount+F2FFQ56amount+F2FFQ57amount+F2FFQ58amount+F2
                 F2sea = F2FFQ25amount+F2FFQ26amount+F2FFQ27amount+F2FFQ28amount+F2
                 F2meat = F2FFQ14amount+F2FFQ17amount+F2FFQ18amount+F2FFQ23amount+F
                     F2FFQ19amount+F2FFQ20amount+F2FFQ21amount+F2FFQ22amount,
                 F2eggs = F2FFQ49amount,
                 F2grains = F2FFQ9amount+F2FFQ10amount+F2FFQ11amount+F2FFQ12amount+
                     F2FFQ42amount+F2FFQ43amount+F2FFQ44amount+F2FFQ46amount,
                 F2alcohol = F2FFQ94amount+F2FFQ95amount+F2FFQ96amount+F2FFQ97amoun
                 F2sugary = F2FFQ61amount+F2FFQ62amount+F2FFQ64amount+F2FFQ65amount
                     F2FFQ90amount+F2FFQ91amount+F2FFQ92amount,
                 F2fats = F2FFQ73amount+F2FFQ74amount+F2FFQ75amount+F2FFQ51amount+F
                        F2FFQ47amount,
                 F3veg = F3FFQ30amount+F3FFQ31amount+F3FFQ32amount+F3FFQ33amount+F3
                     F3FFQ37amount+F3FFQ38amount+F3FFQ39amount+F3FFQ45amount+F3FFQ5
                 F3fru = F3FFQ55amount+F3FFQ56amount+F3FFQ57amount+F3FFQ58amount+F3
                 F3sea = F3FFQ25amount+F3FFQ26amount+F3FFQ27amount+F3FFQ28amount+F3
                 F3meat = F3FFQ14amount+F3FFQ17amount+F3FFQ18amount+F3FFQ23amount+F
                     F3FFQ19amount+F3FFQ20amount+F3FFQ21amount+F3FFQ22amount,
                 F3eggs = F3FFQ49amount,
                 F3grains = F3FFQ9amount+F3FFQ10amount+F3FFQ11amount+F3FFQ12amount+
                     F3FFQ42amount+F3FFQ43amount+F3FFQ44amount+F3FFQ46amount,
                 F3alcohol = F3FFQ94amount+F3FFQ95amount+F3FFQ96amount+F3FFQ97amoun
                 F3sugary = F3FFQ61amount+F3FFQ62amount+F3FFQ64amount+F3FFQ65amount
                     F3FFQ90amount+F3FFQ91amount+F3FFQ92amount,
                 F3fats = F3FFQ73amount+F3FFQ74amount+F3FFQ75amount+F3FFQ51amount+F
                        F3FFQ47amount)
```

4.3 Table 1

In [150…

```
## Vector of variables to summarize (ADD DIABETES AND PA)
baselinevars <- c("sex", "age_cat", "edu","occ_b", "bmi_cat", "sm_b", "cvevent_b",

## Vector of categorical variables that need transformation
catvars <- c("sex", "age_cat","bmi_cat", "edu", "occ_b","sm_b","cvevent_b", "HTA_b

## Create the table object
tab1 <- CreateTableOne(vars = baselinevars, data = ch_sc, factorVars = catvars, te
tab1Mat <- print(tab1, smd = TRUE)
tab1_csv <- write.csv(tab1Mat, file = "Table1.csv")

tab1_str <- CreateTableOne(vars = baselinevars, strata = "total_dairy_freq_b_serv3
tab1Mat_str <- print(tab1_str, smd = TRUE)
tab1_csv_str <- write.csv(tab1Mat_str, file = "Table1_strtertiles.csv")
```

```
                              Overall
n                             1745
sex = 1 (%)                    739 (42.3)
age_cat (%)
   1                          1064 (61.0)
   2                           385 (22.1)
   3                           296 (17.0)
edu (%)
   Elementary                 1189 (68.1)
   High school                 278 (15.9)
   Superior                    278 (15.9)
occ_b (%)
   1                            85 ( 5.0)
   2                           192 (11.2)
   3                           207 (12.1)
   9                          1226 (71.7)
bmi_cat (%)
   1                           664 (38.5)
   2                           720 (41.7)
   3                           342 (19.8)
sm_b (%)
   0                           730 (42.0)
   1                           755 (43.4)
   2                           255 (14.7)
cvevent_b = 1 (%)              353 (20.3)
HTA_b = 1 (%)                 1167 (67.0)
alc_b (%)
   0                           410 (25.8)
   1                           753 (47.4)
   2                           426 (26.8)
depre_b = 1 (%)                190 (12.0)
totalcal_b (mean (SD)) 1685.07 (561.83)
famincome_b (%)
   1                           444 (39.4)
   2                           523 (46.4)
   3                           159 (14.1)
diab_b = 1 (%)                 206 (11.8)
pa_b (%)
   High                        293 (19.2)
   Low                         696 (45.6)
   Medium                      538 (35.2)
                             Stratified by total_dairy_freq_b_serv3
                              Once or less than once per day
n                              145
sex = 1 (%)                     54 (37.2)
age_cat (%)
   1                           101 (69.7)
   2                            33 (22.8)
   3                            11 ( 7.6)
edu (%)
   Elementary                  104 (71.7)
   High school                  18 (12.4)
   Superior                     23 (15.9)
occ_b (%)
   1                             4 ( 2.8)
   2                            16 (11.3)
   3                            25 (17.6)
   9                            97 (68.3)
bmi_cat (%)
   1                            60 (41.7)
   2                            54 (37.5)
   3                            30 (20.8)
sm_b (%)
```

```
     0                        61 (42.1)
     1                        57 (39.3)
     2                        27 (18.6)
  cvevent_b = 1 (%)           28 (19.4)
  HTA_b = 1 (%)               94 (64.8)
  alc_b (%)
     0                        33 (24.6)
     1                        58 (43.3)
     2                        43 (32.1)
  depre_b = 1 (%)             21 (16.9)
  totalcal_b (mean (SD)) 1235.55 (420.53)
  famincome_b (%)
     1                        39 (44.8)
     2                        40 (46.0)
     3                         8 ( 9.2)
  diab_b = 1 (%)              19 (13.1)
  pa_b (%)
     High                     25 (20.5)
     Low                      58 (47.5)
     Medium                   39 (32.0)
                         Stratified by total_dairy_freq_b_serv3
                          More than once/less than 3 times per day
  n                          788
  sex = 1 (%)                360 (45.7)
  age_cat (%)
     1                       492 (62.4)
     2                       174 (22.1)
     3                       122 (15.5)
  edu (%)
     Elementary              518 (65.7)
     High school             134 (17.0)
     Superior                136 (17.3)
  occ_b (%)
     1                        39 ( 5.1)
     2                        95 (12.4)
     3                        85 (11.1)
     9                       546 (71.4)
  bmi_cat (%)
     1                       291 (37.4)
     2                       333 (42.8)
     3                       154 (19.8)
  sm_b (%)
     0                       310 (39.5)
     1                       362 (46.1)
     2                       113 (14.4)
  cvevent_b = 1 (%)          159 (20.3)
  HTA_b = 1 (%)              530 (67.3)
  alc_b (%)
     0                       163 (22.7)
     1                       348 (48.5)
     2                       207 (28.8)
  depre_b = 1 (%)             71 ( 9.9)
  totalcal_b (mean (SD)) 1596.03 (505.46)
  famincome_b (%)
     1                       194 (37.5)
     2                       242 (46.7)
     3                        82 (15.8)
  diab_b = 1 (%)              80 (10.2)
  pa_b (%)
     High                    139 (20.1)
     Low                     302 (43.8)
     Medium                  249 (36.1)
                         Stratified by total_dairy_freq_b_serv3
                          3 or more times per day SMD
```

```
  n                         812                 
  sex = 1 (%)               325 (40.0)          0.115
  age_cat (%)                                   0.249
     1                      471 (58.0)          
     2                      178 (21.9)          
     3                      163 (20.1)          
  edu (%)                                       0.109
     Elementary             567 (69.8)          
     High school            126 (15.5)          
     Superior               119 (14.7)          
  occ_b (%)                                     0.163
     1                       42 ( 5.2)          
     2                       81 (10.1)          
     3                       97 (12.1)          
     9                      583 (72.6)          
  bmi_cat (%)                                   0.074
     1                      313 (38.9)          
     2                      333 (41.4)          
     3                      158 (19.7)          
  sm_b (%)                                      0.125
     0                      359 (44.3)          
     1                      336 (41.5)          
     2                      115 (14.2)          
  cvevent_b = 1 (%)         166 (20.4)          0.017
  HTA_b = 1 (%)             543 (67.0)          0.035
  alc_b (%)                                     0.150
     0                      214 (29.0)          
     1                      347 (47.1)          
     2                      176 (23.9)          
  depre_b = 1 (%)            98 (13.2)          0.139
  totalcal_b (mean (SD)) 1851.76 (572.55)       0.825
  famincome_b (%)                               0.148
     1                      211 (40.5)          
     2                      241 (46.3)          
     3                       69 (13.2)          
  diab_b = 1 (%)            107 (13.2)          0.063
  pa_b (%)                                      0.079
     High                   129 (18.0)          
     Low                    336 (47.0)          
     Medium                 250 (35.0)          
```

In [151…

```
do.call(rbind , by(ch_sc$totalcal_b, ch_sc$total_dairy_freq_b_serv3, summary))
```

A matrix: 3 × 6 of type dbl

	Min.	1st Qu.	Median	Mean	3rd Qu.	Max.
**Once or less than once per day**	517.3	952.5	1179	1236	1488	2753
**More than once/less than 3 times per day**	554.8	1222.2	1529	1596	1913	3564
**3 or more times per day**	633.2	1422.1	1823	1852	2205	3767

LTFU

In [152…

```
tab1_str <- CreateTableOne(vars = baselinevars, strata = "ltfu", data = ch_sc, fac
tab1Mat_str <- print(tab1_str, smd = TRUE)
tab1_csv_str <- write.csv(tab1Mat_str, file = "Table1_strLTFU.csv")
```

```
                          Stratified by ltfu
                           0                 1                 SMD
  n                            411              1334
  sex = 1 (%)                  232 (56.4)        507 (38.0)     0.376
  age_cat (%)                                                   0.322
     1                         214 (52.1)        850 (63.7)
     2                          88 (21.4)        297 (22.3)
     3                         109 (26.5)        187 (14.0)
  edu (%)                                                       0.098
     Elementary                294 (71.5)        895 (67.1)
     High school                60 (14.6)        218 (16.3)
     Superior                   57 (13.9)        221 (16.6)
  occ_b (%)                                                     0.034
     1                          21 ( 5.2)         64 ( 4.9)
     2                          48 (11.9)        144 (11.0)
     3                          47 (11.6)        160 (12.3)
     9                         289 (71.4)        937 (71.8)
  bmi_cat (%)                                                   0.199
     1                         135 (33.3)        529 (40.0)
     2                         166 (41.0)        554 (41.9)
     3                         104 (25.7)        238 (18.0)
  sm_b (%)                                                      0.211
     0                         140 (34.3)        590 (44.3)
     1                         195 (47.8)        560 (42.0)
     2                          73 (17.9)        182 (13.7)
  cvevent_b = 1 (%)            108 (26.3)        245 (18.4)     0.191
  HTA_b = 1 (%)                310 (75.4)        857 (64.3)     0.243
  alc_b (%)                                                     0.008
     0                          94 (25.6)        316 (25.9)
     1                         175 (47.7)        578 (47.3)
     2                          98 (26.7)        328 (26.8)
  depre_b = 1 (%)               53 (14.3)        137 (11.3)     0.090
  totalcal_b (mean (SD)) 1677.35 (571.69) 1687.45 (558.96)  0.018
  famincome_b (%)                                               0.211
     1                          38 (43.7)        406 (39.1)
     2                          42 (48.3)        481 (46.3)
     3                           7 ( 8.0)        152 (14.6)
  diab_b = 1 (%)                74 (18.0)        132 ( 9.9)     0.236
  pa_b (%)                                                      0.094
     High                       77 (22.0)        216 (18.4)
     Low                       151 (43.1)        545 (46.3)
     Medium                    122 (34.9)        416 (35.3)
```

5. Missingness

In [153…

```
ch_sc_miss <- ch_sc %>%
    dplyr::select(pt, sex, age_b, occ_b, pa_b, sm_b,bmi_b,cvevent_b,HTA_b,depre_b,
                     F2SCD_yn, memory_b, verbal_b,stroop_b,do40_b,CERAD_b, F2memory
                      F3verbal,F3stroop,F3do40,F3CERAD, veg_b ,fru_b ,sea_b ,meat_
                F3fru,F3sea,F3meat,F3eggs,F3grains ,F3alcohol ,F3sugary , F3fats ,

                  F2nonferm_dairy, F2fullfat_dairy,F2nonfat_dairy,F2sugar_dairy,F3

pct_miss(ch_sc_miss) #percentage missingness
gg_miss_var(ch_sc_miss, show_pct = TRUE)
vis_miss(ch_sc_miss)
ch_sc_miss %>%
  gg_miss_var(show_pct = TRUE, facet = CDR_b)
```

32.1797994269341

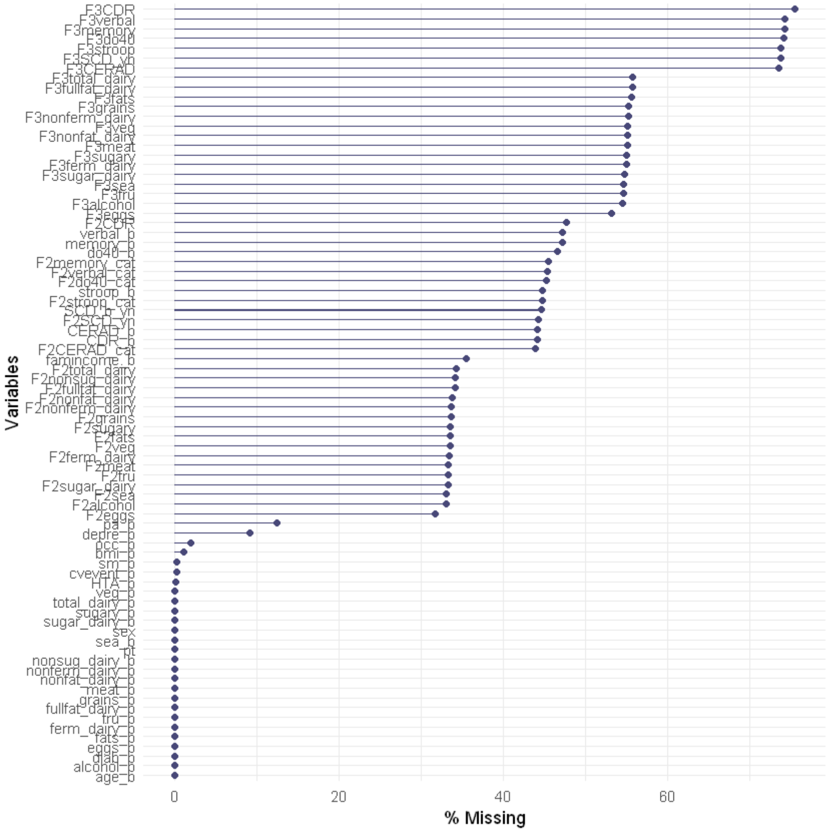

Variables
% Missing
0
20
40
60

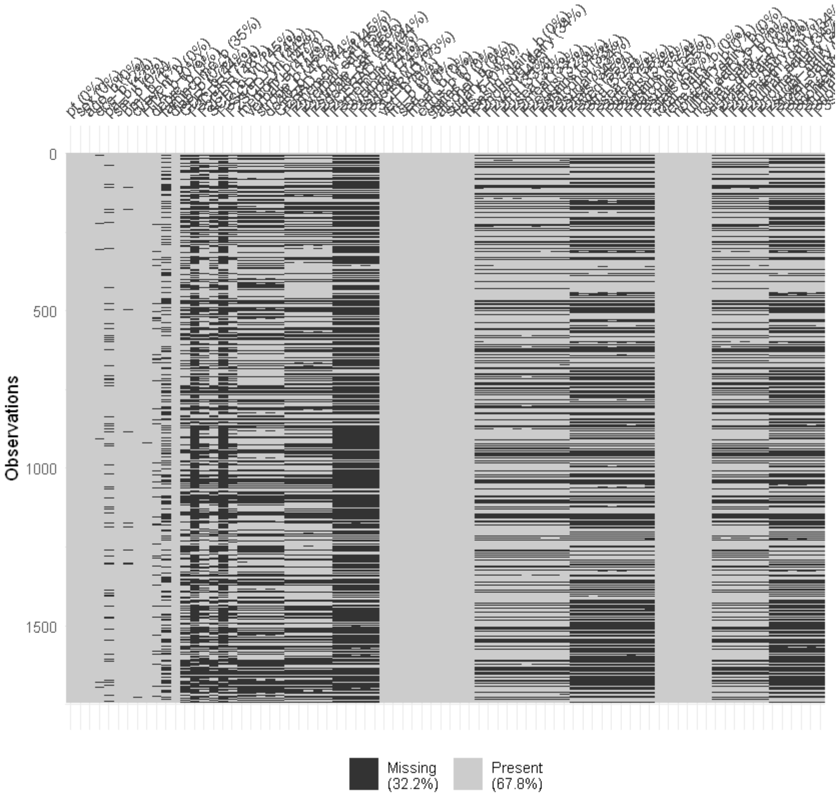

Observations
0
500
1000
1500
Missing (32.2%)
Present (67.8%)

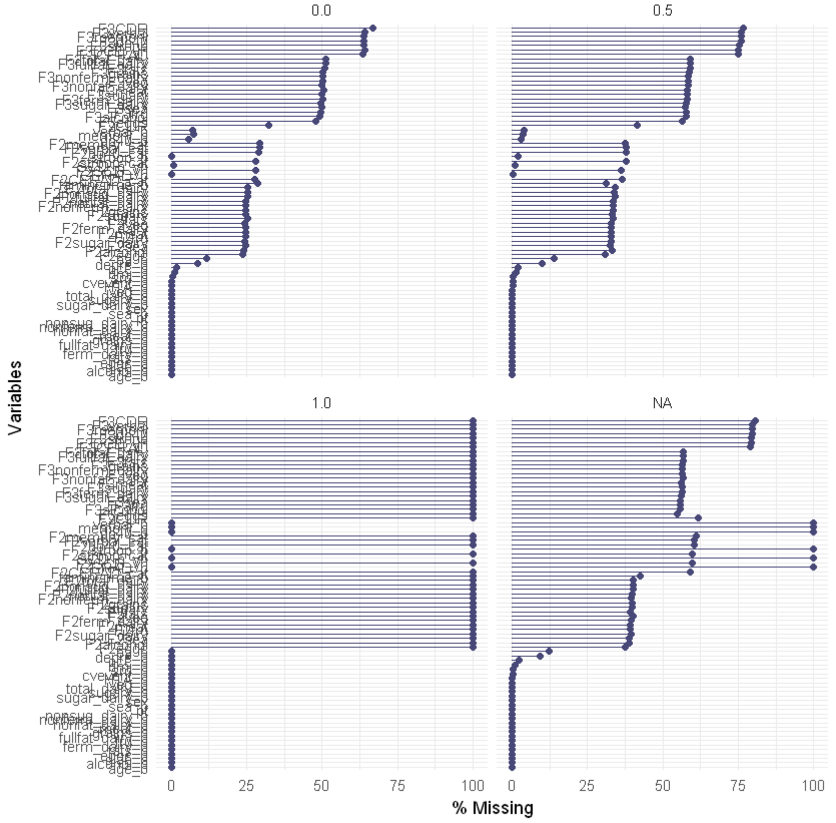

7. IPTW and MSMs

Covariate selection

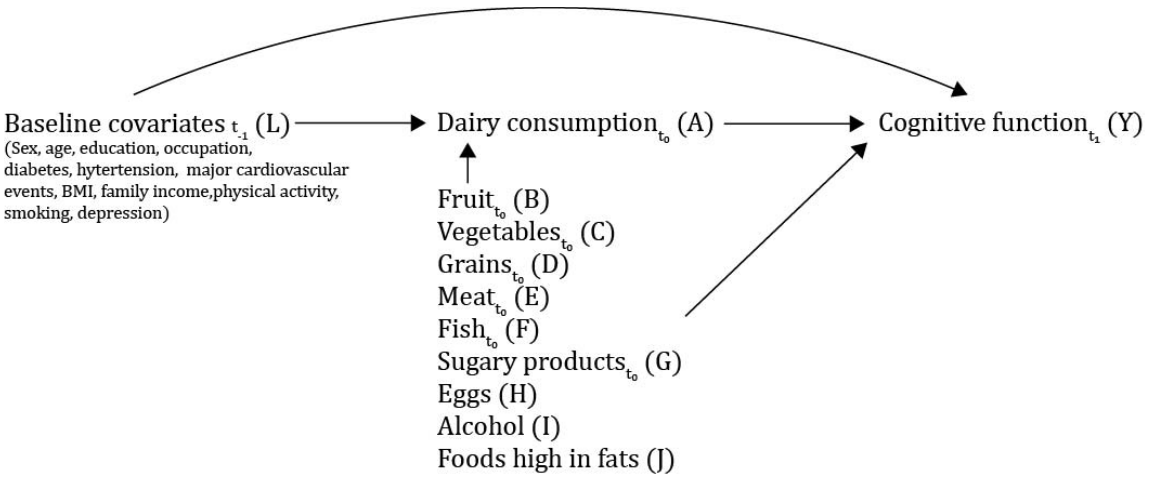

Here, we target the Average Total Causal effect of increasing dairy consumption by 100 g while maintaing other food groups constant (increasing total amount of food only due to dairy consumption) using **all components models**

In [154…

```
#estimate weights with a linear model for the treatment dose
#and with kerney density estimation for obtaining the generalized propensity score
#see https://en.wikipedia.org/wiki/Kernel_density_estimation
#using the weightit package

#################
###TOTAL DAIRY###
#################
```

```
weight.model = weightit(formula("total_dairy_b ~ veg_b+fru_b+sea_b+meat_b+eggs_b+gr
                 age_cat+edu+sm_b+HTA_b+depre_b+pa_b+cvevent_b+famincome_b+occ_b+br
                        method = "ps", use.kernel=T)

#check weights
summary(weight.model$weights)

#add weights to data
ch_sc$ipw_totaldairy <- weight.model$weights

#covariate balance for weights obtained with linear model for treatment dose
#and kernel density estimation
balance.table.2 = bal.tab(weight.model, r.threshold=0.1)$Balance
balance.table.2

#################
###FERM DAIRY###
#################
weight.model = weightit(formula("ferm_dairy_b ~ nonferm_dairy_b+veg_b+fru_b+sea_b+r
                 age_cat+edu+sm_b+HTA_b+depre_b+pa_b+cvevent_b+famincome_b+diab_b+
                        method = "ps", use.kernel=T)

#check weights
summary(weight.model$weights)

#add weights to data
ch_sc$ipw_ferm <- weight.model$weights

#covariate balance for weights obtained with linear model for treatment dose
#and kernel density estimation
balance.table.2 = bal.tab(weight.model, r.threshold=0.1)$Balance
balance.table.2

#################
##NONFERM DAIRY##
#################
weight.model = weightit(formula("nonferm_dairy_b ~ ferm_dairy_b+veg_b+fru_b+sea_b+r
                 age_cat+edu+sm_b+HTA_b+depre_b+pa_b+cvevent_b+famincome_b+diab_b+
                        method = "ps", use.kernel=T)

#check weights
summary(weight.model$weights)

#add weights to data
ch_sc$ipw_nonferm <- weight.model$weights

#covariate balance for weights obtained with linear model for treatment dose
#and kernel density estimation
balance.table.2 = bal.tab(weight.model, r.threshold=0.1)$Balance
balance.table.2

#################
##FULLFAT DAIRY##
#################
weight.model = weightit(formula("fullfat_dairy_b ~ nonfat_dairy_b+veg_b+fru_b+sea_
                 age_cat+edu+sm_b+HTA_b+depre_b+pa_b+cvevent_b+famincome_b+diab_b+
                        method = "ps", use.kernel=T)

#check weights
summary(weight.model$weights)

#add weights to data
ch_sc$ipw_fullfat <- weight.model$weights
```

```
#covariate balance for weights obtained with linear model for treatment dose
#and kernel density estimation
balance.table.2 = bal.tab(weight.model, r.threshold=0.1)$Balance
balance.table.2

#################
###NONFAT DAIRY##
#################
weight.model = weightit(formula("nonfat_dairy_b ~ fullfat_dairy_b+veg_b+fru_b+sea_l
                 age_cat+edu+sm_b+HTA_b+depre_b+pa_b+cvevent_b+famincome_b+diab_b+c
                        method = "ps", use.kernel=T)

#check weights
summary(weight.model$weights)

#add weights to data
ch_sc$ipw_nonfat <- weight.model$weights

#covariate balance for weights obtained with linear model for treatment dose
#and kernel density estimation
balance.table.2 = bal.tab(weight.model, r.threshold=0.1)$Balance
balance.table.2

#################
###SUGARY DAIRY##
#################
weight.model = weightit(formula("sugar_dairy_b ~ nonsug_dairy_b+veg_b+fru_b+sea_b+r
                 age_cat+edu+sm_b+HTA_b+depre_b+pa_b+cvevent_b+famincome_b+diab_b+c
                        method = "ps", use.kernel=T)

#check weights
summary(weight.model$weights)

#add weights to data
ch_sc$ipw_sugdairy <- weight.model$weights

#covariate balance for weights obtained with linear model for treatment dose
#and kernel density estimation
balance.table.2 = bal.tab(weight.model, r.threshold=0.1)$Balance
balance.table.2
```

```
   Min. 1st Qu.  Median    Mean 3rd Qu.    Max.
   0.26    0.90    0.97    1.11    1.09   33.38
```

A data.frame: 43 × 4

	Type	Corr.Un	Corr.Adj	R.Threshold
	<chr>	<dbl>	<dbl>	<chr>
**veg_b**	Contin.	NA	-0.0282222	Balanced, <0.1
**fru_b**	Contin.	NA	-0.0186061	Balanced, <0.1
**sea_b**	Contin.	NA	0.0028679	Balanced, <0.1
**meat_b**	Contin.	NA	0.0130271	Balanced, <0.1
**eggs_b**	Contin.	NA	-0.0869466	Balanced, <0.1
**grains_b**	Contin.	NA	0.0087258	Balanced, <0.1
**alcohol_b**	Contin.	NA	-0.0075635	Balanced, <0.1
**sugary_b**	Contin.	NA	0.0009908	Balanced, <0.1
**fats_b**	Contin.	NA	0.0204500	Balanced, <0.1
**sex**	Binary	NA	0.0092303	Balanced, <0.1
**age_cat_1**	Binary	NA	-0.0215910	Balanced, <0.1
**age_cat_2**	Binary	NA	0.0056876	Balanced, <0.1
**age_cat_3**	Binary	NA	0.0217789	Balanced, <0.1
**edu_Elementary**	Binary	NA	-0.0164239	Balanced, <0.1
**edu_High school**	Binary	NA	0.0030044	Balanced, <0.1
**edu_Superior**	Binary	NA	0.0179063	Balanced, <0.1
**sm_b_0**	Binary	NA	-0.0132610	Balanced, <0.1
**sm_b_1**	Binary	NA	0.0180487	Balanced, <0.1
**sm_b_2**	Binary	NA	-0.0067892	Balanced, <0.1
**sm_b:<NA>**	Binary	NA	0.0018585	Balanced, <0.1
**HTA_b**	Binary	NA	0.0298071	Balanced, <0.1
**HTA_b:<NA>**	Binary	NA	0.0133765	Balanced, <0.1
**depre_b**	Binary	NA	-0.0250559	Balanced, <0.1
**depre_b:<NA>**	Binary	NA	0.0060506	Balanced, <0.1
**pa_b_High**	Binary	NA	0.0043410	Balanced, <0.1
**pa_b_Low**	Binary	NA	0.0050346	Balanced, <0.1
**pa_b_Medium**	Binary	NA	-0.0088274	Balanced, <0.1
**pa_b:<NA>**	Binary	NA	-0.0097893	Balanced, <0.1
**cvevent_b**	Binary	NA	0.0041898	Balanced, <0.1
**cvevent_b:<NA>**	Binary	NA	-0.0267105	Balanced, <0.1
**famincome_b_1**	Binary	NA	-0.0189931	Balanced, <0.1
**famincome_b_2**	Binary	NA	0.0178243	Balanced, <0.1
**famincome_b_3**	Binary	NA	0.0011267	Balanced, <0.1
**famincome_b:<NA>**	Binary	NA	-0.0433482	Balanced, <0.1

	Type	Corr.Un	Corr.Adj	R.Threshold
	<chr>	<dbl>	<dbl>	<chr>
**occ_b_1**	Binary	NA	0.0191806	Balanced, <0.1
**occ_b_2**	Binary	NA	-0.0008519	Balanced, <0.1
**occ_b_3**	Binary	NA	-0.0067995	Balanced, <0.1
**occ_b_9**	Binary	NA	-0.0037335	Balanced, <0.1
**occ_b:<NA>**	Binary	NA	0.0027626	Balanced, <0.1
**bmi_cat_1**	Binary	NA	-0.0117858	Balanced, <0.1
**bmi_cat_2**	Binary	NA	-0.0058169	Balanced, <0.1
**bmi_cat_3**	Binary	NA	0.0215813	Balanced, <0.1
**bmi_cat:<NA>**	Binary	NA	-0.0009052	Balanced, <0.1

```
   Min. 1st Qu.  Median    Mean 3rd Qu.    Max. 
   0.21    0.88    0.98    1.20    1.14   70.12 
```

A data.frame: 45 × 4

	Type	Corr.Un	Corr.Adj	R.Threshold
	<chr>	<dbl>	<dbl>	<chr>
**nonferm_dairy_b**	Contin.	NA	-0.0218025	Balanced, <0.1
**veg_b**	Contin.	NA	0.0071093	Balanced, <0.1
**fru_b**	Contin.	NA	-0.1272407	Not Balanced, >0.1
**sea_b**	Contin.	NA	-0.0254006	Balanced, <0.1
**meat_b**	Contin.	NA	0.0211915	Balanced, <0.1
**eggs_b**	Contin.	NA	-0.0385304	Balanced, <0.1
**grains_b**	Contin.	NA	-0.0139661	Balanced, <0.1
**alcohol_b**	Contin.	NA	0.0206342	Balanced, <0.1
**sugary_b**	Contin.	NA	-0.0125768	Balanced, <0.1
**fats_b**	Contin.	NA	0.0030490	Balanced, <0.1
**sex**	Binary	NA	0.0221371	Balanced, <0.1
**age_cat_1**	Binary	NA	-0.0301910	Balanced, <0.1
**age_cat_2**	Binary	NA	0.0027076	Balanced, <0.1
**age_cat_3**	Binary	NA	0.0362496	Balanced, <0.1
**edu_Elementary**	Binary	NA	-0.0298511	Balanced, <0.1
**edu_High school**	Binary	NA	0.0224870	Balanced, <0.1
**edu_Superior**	Binary	NA	0.0155189	Balanced, <0.1
**sm_b_0**	Binary	NA	0.0255641	Balanced, <0.1
**sm_b_1**	Binary	NA	0.0044964	Balanced, <0.1
**sm_b_2**	Binary	NA	-0.0419726	Balanced, <0.1
**sm_b:<NA>**	Binary	NA	0.0026981	Balanced, <0.1
**HTA_b**	Binary	NA	0.0579515	Balanced, <0.1
**HTA_b:<NA>**	Binary	NA	0.0102727	Balanced, <0.1
**depre_b**	Binary	NA	-0.1075504	Not Balanced, >0.1
**depre_b:<NA>**	Binary	NA	-0.0067773	Balanced, <0.1
**pa_b_High**	Binary	NA	0.0251437	Balanced, <0.1
**pa_b_Low**	Binary	NA	-0.0485760	Balanced, <0.1
**pa_b_Medium**	Binary	NA	0.0299184	Balanced, <0.1
**pa_b:<NA>**	Binary	NA	-0.0067150	Balanced, <0.1
**cvevent_b**	Binary	NA	0.0074249	Balanced, <0.1
**cvevent_b:<NA>**	Binary	NA	-0.0290929	Balanced, <0.1
**famincome_b_1**	Binary	NA	-0.0210312	Balanced, <0.1
**famincome_b_2**	Binary	NA	0.0090378	Balanced, <0.1
**famincome_b_3**	Binary	NA	0.0165708	Balanced, <0.1

	Type	Corr.Un	Corr.Adj	R.Threshold
	<chr>	<dbl>	<dbl>	<chr>
**famincome_b:<NA>**	Binary	NA	-0.0299182	Balanced, <0.1
**diab_b**	Binary	NA	-0.0738345	Balanced, <0.1
**occ_b_1**	Binary	NA	0.0076348	Balanced, <0.1
**occ_b_2**	Binary	NA	0.0064638	Balanced, <0.1
**occ_b_3**	Binary	NA	-0.0127275	Balanced, <0.1
**occ_b_9**	Binary	NA	0.0010023	Balanced, <0.1
**occ_b:<NA>**	Binary	NA	0.0105061	Balanced, <0.1
**bmi_cat_1**	Binary	NA	-0.0021976	Balanced, <0.1
**bmi_cat_2**	Binary	NA	-0.0178952	Balanced, <0.1
**bmi_cat_3**	Binary	NA	0.0248194	Balanced, <0.1
**bmi_cat:<NA>**	Binary	NA	-0.0008877	Balanced, <0.1

```
Min. 1st Qu.  Median    Mean 3rd Qu.    Max.
0.32    0.92    1.11    1.42    1.29   78.43
```

A data.frame: 45 × 4

	Type	Corr.Un	Corr.Adj	R.Threshold
	<chr>	<dbl>	<dbl>	<chr>
**ferm_dairy_b**	Contin.	NA	-0.0300446	Balanced, <0.1
**veg_b**	Contin.	NA	-0.0668009	Balanced, <0.1
**fru_b**	Contin.	NA	-0.0237010	Balanced, <0.1
**sea_b**	Contin.	NA	-0.0050150	Balanced, <0.1
**meat_b**	Contin.	NA	-0.0177580	Balanced, <0.1
**eggs_b**	Contin.	NA	-0.0760161	Balanced, <0.1
**grains_b**	Contin.	NA	-0.0346220	Balanced, <0.1
**alcohol_b**	Contin.	NA	0.0152488	Balanced, <0.1
**sugary_b**	Contin.	NA	-0.2283182	Not Balanced, >0.1
**fats_b**	Contin.	NA	-0.0645535	Balanced, <0.1
**sex**	Binary	NA	-0.0248629	Balanced, <0.1
**age_cat_1**	Binary	NA	0.0576489	Balanced, <0.1
**age_cat_2**	Binary	NA	-0.0338675	Balanced, <0.1
**age_cat_3**	Binary	NA	-0.0375100	Balanced, <0.1
**edu_Elementary**	Binary	NA	-0.0162217	Balanced, <0.1
**edu_High school**	Binary	NA	0.0226437	Balanced, <0.1
**edu_Superior**	Binary	NA	-0.0019906	Balanced, <0.1
**sm_b_0**	Binary	NA	-0.0007157	Balanced, <0.1
**sm_b_1**	Binary	NA	0.0076708	Balanced, <0.1
**sm_b_2**	Binary	NA	-0.0097511	Balanced, <0.1
**sm_b:<NA>**	Binary	NA	0.0047895	Balanced, <0.1
**HTA_b**	Binary	NA	-0.0338985	Balanced, <0.1
**HTA_b:<NA>**	Binary	NA	-0.1641734	Not Balanced, >0.1
**depre_b**	Binary	NA	-0.0012477	Balanced, <0.1
**depre_b:<NA>**	Binary	NA	0.0194686	Balanced, <0.1
**pa_b_High**	Binary	NA	-0.0034224	Balanced, <0.1
**pa_b_Low**	Binary	NA	0.0444556	Balanced, <0.1
**pa_b_Medium**	Binary	NA	-0.0435280	Balanced, <0.1
**pa_b:<NA>**	Binary	NA	-0.0030008	Balanced, <0.1
**cvevent_b**	Binary	NA	0.0142575	Balanced, <0.1
**cvevent_b:<NA>**	Binary	NA	-0.0188613	Balanced, <0.1
**famincome_b_1**	Binary	NA	0.0037873	Balanced, <0.1
**famincome_b_2**	Binary	NA	0.0092358	Balanced, <0.1
**famincome_b_3**	Binary	NA	-0.0185423	Balanced, <0.1

	Type	Corr.Un	Corr.Adj	R.Threshold
	<chr>	<dbl>	<dbl>	<chr>
**famincome_b:<NA>**	Binary	NA	0.0166541	Balanced, <0.1
**diab_b**	Binary	NA	0.0189377	Balanced, <0.1
**occ_b_1**	Binary	NA	-0.0422981	Balanced, <0.1
**occ_b_2**	Binary	NA	0.0477797	Balanced, <0.1
**occ_b_3**	Binary	NA	0.0091053	Balanced, <0.1
**occ_b_9**	Binary	NA	-0.0196716	Balanced, <0.1
**occ_b:<NA>**	Binary	NA	-0.0098542	Balanced, <0.1
**bmi_cat_1**	Binary	NA	-0.0079291	Balanced, <0.1
**bmi_cat_2**	Binary	NA	0.0027750	Balanced, <0.1
**bmi_cat_3**	Binary	NA	0.0062452	Balanced, <0.1
**bmi_cat:<NA>**	Binary	NA	-0.0286595	Balanced, <0.1

```
   Min. 1st Qu.  Median    Mean 3rd Qu.    Max. 
  0.269   0.895   0.992   1.099   1.077  12.840 
```

A data.frame: 45 × 4

	Type	Corr.Un	Corr.Adj	R.Threshold
	<chr>	<dbl>	<dbl>	<chr>
**nonfat_dairy_b**	Contin.	NA	-0.0430353	Balanced, <0.1
**veg_b**	Contin.	NA	-0.0177009	Balanced, <0.1
**fru_b**	Contin.	NA	-0.0284389	Balanced, <0.1
**sea_b**	Contin.	NA	-0.0044185	Balanced, <0.1
**meat_b**	Contin.	NA	0.0101543	Balanced, <0.1
**eggs_b**	Contin.	NA	-0.0253491	Balanced, <0.1
**grains_b**	Contin.	NA	-0.0091859	Balanced, <0.1
**alcohol_b**	Contin.	NA	-0.0045346	Balanced, <0.1
**sugary_b**	Contin.	NA	0.0191201	Balanced, <0.1
**fats_b**	Contin.	NA	0.0121112	Balanced, <0.1
**sex**	Binary	NA	-0.0128899	Balanced, <0.1
**age_cat_1**	Binary	NA	-0.0107188	Balanced, <0.1
**age_cat_2**	Binary	NA	-0.0009584	Balanced, <0.1
**age_cat_3**	Binary	NA	0.0149908	Balanced, <0.1
**edu_Elementary**	Binary	NA	-0.0049611	Balanced, <0.1
**edu_High school**	Binary	NA	-0.0092101	Balanced, <0.1
**edu_Superior**	Binary	NA	0.0155265	Balanced, <0.1
**sm_b_0**	Binary	NA	-0.0077748	Balanced, <0.1
**sm_b_1**	Binary	NA	0.0148696	Balanced, <0.1
**sm_b_2**	Binary	NA	-0.0099894	Balanced, <0.1
**sm_b:<NA>**	Binary	NA	-0.0209008	Balanced, <0.1
**HTA_b**	Binary	NA	0.0089592	Balanced, <0.1
**HTA_b:<NA>**	Binary	NA	0.0140978	Balanced, <0.1
**depre_b**	Binary	NA	0.0011053	Balanced, <0.1
**depre_b:<NA>**	Binary	NA	-0.0017503	Balanced, <0.1
**pa_b_High**	Binary	NA	0.0158376	Balanced, <0.1
**pa_b_Low**	Binary	NA	0.0211327	Balanced, <0.1
**pa_b_Medium**	Binary	NA	-0.0350883	Balanced, <0.1
**pa_b:<NA>**	Binary	NA	0.0095596	Balanced, <0.1
**cvevent_b**	Binary	NA	-0.0090843	Balanced, <0.1
**cvevent_b:<NA>**	Binary	NA	-0.0298657	Balanced, <0.1
**famincome_b_1**	Binary	NA	-0.0194036	Balanced, <0.1
**famincome_b_2**	Binary	NA	0.0204739	Balanced, <0.1
**famincome_b_3**	Binary	NA	-0.0020919	Balanced, <0.1

	Type	Corr.Un	Corr.Adj	R.Threshold
	<chr>	<dbl>	<dbl>	<chr>
**famincome_b:<NA>**	Binary	NA	-0.0207708	Balanced, <0.1
**diab_b**	Binary	NA	-0.0128921	Balanced, <0.1
**occ_b_1**	Binary	NA	0.0126724	Balanced, <0.1
**occ_b_2**	Binary	NA	-0.0119018	Balanced, <0.1
**occ_b_3**	Binary	NA	-0.0109583	Balanced, <0.1
**occ_b_9**	Binary	NA	0.0101621	Balanced, <0.1
**occ_b:<NA>**	Binary	NA	-0.0056694	Balanced, <0.1
**bmi_cat_1**	Binary	NA	-0.0079825	Balanced, <0.1
**bmi_cat_2**	Binary	NA	-0.0089993	Balanced, <0.1
**bmi_cat_3**	Binary	NA	0.0208757	Balanced, <0.1
**bmi_cat:<NA>**	Binary	NA	0.0069484	Balanced, <0.1

```
   Min. 1st Qu.  Median    Mean 3rd Qu.    Max. 
    0.1     1.2     2.7     4.8     3.5   903.6 
```

A data.frame: 45 × 4

	Type	Corr.Un	Corr.Adj	R.Threshold
	<chr>	<dbl>	<dbl>	<chr>
**fullfat_dairy_b**	Contin.	NA	-0.0080601	Balanced, <0.1
**veg_b**	Contin.	NA	-0.0255636	Balanced, <0.1
**fru_b**	Contin.	NA	-0.0257349	Balanced, <0.1
**sea_b**	Contin.	NA	-0.0466832	Balanced, <0.1
**meat_b**	Contin.	NA	-0.0220019	Balanced, <0.1
**eggs_b**	Contin.	NA	-0.0162059	Balanced, <0.1
**grains_b**	Contin.	NA	0.0087252	Balanced, <0.1
**alcohol_b**	Contin.	NA	-0.0126262	Balanced, <0.1
**sugary_b**	Contin.	NA	0.0213750	Balanced, <0.1
**fats_b**	Contin.	NA	0.0099025	Balanced, <0.1
**sex**	Binary	NA	0.0007728	Balanced, <0.1
**age_cat_1**	Binary	NA	-0.0183224	Balanced, <0.1
**age_cat_2**	Binary	NA	0.0270797	Balanced, <0.1
**age_cat_3**	Binary	NA	-0.0061054	Balanced, <0.1
**edu_Elementary**	Binary	NA	-0.0168273	Balanced, <0.1
**edu_High school**	Binary	NA	0.0149031	Balanced, <0.1
**edu_Superior**	Binary	NA	0.0065212	Balanced, <0.1
**sm_b_0**	Binary	NA	-0.0114733	Balanced, <0.1
**sm_b_1**	Binary	NA	0.0038430	Balanced, <0.1
**sm_b_2**	Binary	NA	0.0106239	Balanced, <0.1
**sm_b:<NA>**	Binary	NA	-0.2564128	Not Balanced, >0.1
**HTA_b**	Binary	NA	0.0061691	Balanced, <0.1
**HTA_b:<NA>**	Binary	NA	-0.0085644	Balanced, <0.1
**depre_b**	Binary	NA	-0.0073460	Balanced, <0.1
**depre_b:<NA>**	Binary	NA	0.0068246	Balanced, <0.1
**pa_b_High**	Binary	NA	-0.0005783	Balanced, <0.1
**pa_b_Low**	Binary	NA	-0.0076348	Balanced, <0.1
**pa_b_Medium**	Binary	NA	0.0084367	Balanced, <0.1
**pa_b:<NA>**	Binary	NA	0.0136567	Balanced, <0.1
**cvevent_b**	Binary	NA	0.0261497	Balanced, <0.1
**cvevent_b:<NA>**	Binary	NA	-0.0082937	Balanced, <0.1
**famincome_b_1**	Binary	NA	0.0185532	Balanced, <0.1
**famincome_b_2**	Binary	NA	-0.0333830	Balanced, <0.1
**famincome_b_3**	Binary	NA	0.0217734	Balanced, <0.1

	Type	Corr.Un	Corr.Adj	R.Threshold
	<chr>	<dbl>	<dbl>	<chr>
**famincome_b:<NA>**	Binary	NA	0.0229316	Balanced, <0.1
**diab_b**	Binary	NA	0.0198146	Balanced, <0.1
**occ_b_1**	Binary	NA	-0.0122751	Balanced, <0.1
**occ_b_2**	Binary	NA	0.0095210	Balanced, <0.1
**occ_b_3**	Binary	NA	0.0023423	Balanced, <0.1
**occ_b_9**	Binary	NA	-0.0024464	Balanced, <0.1
**occ_b:<NA>**	Binary	NA	0.0024691	Balanced, <0.1
**bmi_cat_1**	Binary	NA	-0.0114161	Balanced, <0.1
**bmi_cat_2**	Binary	NA	0.0070417	Balanced, <0.1
**bmi_cat_3**	Binary	NA	0.0052234	Balanced, <0.1
**bmi_cat:<NA>**	Binary	NA	-0.0097421	Balanced, <0.1

```
   Min. 1st Qu.  Median    Mean 3rd Qu.    Max. 
   0.09    1.03    1.27    1.54    1.58   64.12 
```

A data.frame: 45 × 4

	Type	Corr.Un	Corr.Adj	R.Threshold
	<chr>	<dbl>	<dbl>	<chr>
**nonsug_dairy_b**	Contin.	NA	-0.0217310	Balanced, <0.1
**veg_b**	Contin.	NA	0.0098691	Balanced, <0.1
**fru_b**	Contin.	NA	0.0678218	Balanced, <0.1
**sea_b**	Contin.	NA	-0.0409041	Balanced, <0.1
**meat_b**	Contin.	NA	-0.0353202	Balanced, <0.1
**eggs_b**	Contin.	NA	-0.0182576	Balanced, <0.1
**grains_b**	Contin.	NA	-0.0353798	Balanced, <0.1
**alcohol_b**	Contin.	NA	-0.0442139	Balanced, <0.1
**sugary_b**	Contin.	NA	-0.0042697	Balanced, <0.1
**fats_b**	Contin.	NA	-0.0796242	Balanced, <0.1
**sex**	Binary	NA	0.0125395	Balanced, <0.1
**age_cat_1**	Binary	NA	0.0052998	Balanced, <0.1
**age_cat_2**	Binary	NA	0.0191646	Balanced, <0.1
**age_cat_3**	Binary	NA	-0.0280633	Balanced, <0.1
**edu_Elementary**	Binary	NA	0.0358881	Balanced, <0.1
**edu_High school**	Binary	NA	-0.0015330	Balanced, <0.1
**edu_Superior**	Binary	NA	-0.0441591	Balanced, <0.1
**sm_b_0**	Binary	NA	-0.0232072	Balanced, <0.1
**sm_b_1**	Binary	NA	0.0607003	Balanced, <0.1
**sm_b_2**	Binary	NA	-0.0526821	Balanced, <0.1
**sm_b:<NA>**	Binary	NA	0.0072594	Balanced, <0.1
**HTA_b**	Binary	NA	-0.0339875	Balanced, <0.1
**HTA_b:<NA>**	Binary	NA	-0.4097464	Not Balanced, >0.1
**depre_b**	Binary	NA	0.1114150	Not Balanced, >0.1
**depre_b:<NA>**	Binary	NA	-0.0084992	Balanced, <0.1
**pa_b_High**	Binary	NA	0.0304905	Balanced, <0.1
**pa_b_Low**	Binary	NA	-0.0154127	Balanced, <0.1
**pa_b_Medium**	Binary	NA	-0.0090651	Balanced, <0.1
**pa_b:<NA>**	Binary	NA	-0.0337785	Balanced, <0.1
**cvevent_b**	Binary	NA	-0.0100260	Balanced, <0.1
**cvevent_b:<NA>**	Binary	NA	-0.0542219	Balanced, <0.1
**famincome_b_1**	Binary	NA	-0.0291234	Balanced, <0.1
**famincome_b_2**	Binary	NA	0.0539524	Balanced, <0.1
**famincome_b_3**	Binary	NA	-0.0363987	Balanced, <0.1

	Type	Corr.Un	Corr.Adj	R.Threshold
	<chr>	<dbl>	<dbl>	<chr>
**famincome_b:<NA>**	Binary	NA	-0.0495398	Balanced, <0.1
**diab_b**	Binary	NA	0.0300845	Balanced, <0.1
**occ_b_1**	Binary	NA	-0.0001412	Balanced, <0.1
**occ_b_2**	Binary	NA	-0.0098785	Balanced, <0.1
**occ_b_3**	Binary	NA	-0.0216955	Balanced, <0.1
**occ_b_9**	Binary	NA	0.0227010	Balanced, <0.1
**occ_b:<NA>**	Binary	NA	-0.0108673	Balanced, <0.1
**bmi_cat_1**	Binary	NA	0.0265141	Balanced, <0.1
**bmi_cat_2**	Binary	NA	-0.0687771	Balanced, <0.1
**bmi_cat_3**	Binary	NA	0.0527176	Balanced, <0.1
**bmi_cat:<NA>**	Binary	NA	0.0043081	Balanced, <0.1

IPFollow-up Weighting

In [155…

```
weight.model = weightit(formula("ltfu ~ age_cat + sex +occ_b +bmi_cat + sm_b+ cveve
                        method = "ps", use.kernel=T)

#check weights
summary(weight.model$weights)

#add weights to data
ch_sc$ipcw <- weight.model$weights

#covariate balance for weights obtained with linear model for treatment dose
#and kernel density estimation
balance.table.2 = bal.tab(weight.model, r.threshold=0.1)$Balance
balance.table.2
```

```
   Min. 1st Qu.  Median    Mean 3rd Qu.    Max. 
   1.00    1.18    1.31    2.00    1.87   10.15 
```

A data.frame: 23 × 3

	Type	Diff.Un	Diff.Adj
	<chr>	<dbl>	<dbl>
**prop.score**	Distance	NA	-0.0008682
**age_cat_1**	Binary	NA	-0.0006170
**age_cat_2**	Binary	NA	-0.0028425
**age_cat_3**	Binary	NA	0.0034596
**sex**	Binary	NA	0.0063235
**occ_b_1**	Binary	NA	0.0022377
**occ_b_2**	Binary	NA	-0.0027403
**occ_b_3**	Binary	NA	0.0014802
**occ_b_9**	Binary	NA	-0.0009776
**occ_b:<NA>**	Binary	NA	-0.0005958
**bmi_cat_1**	Binary	NA	0.0027023
**bmi_cat_2**	Binary	NA	-0.0008739
**bmi_cat_3**	Binary	NA	-0.0018284
**bmi_cat:<NA>**	Binary	NA	0.0014189
**sm_b_0**	Binary	NA	0.0018465
**sm_b_1**	Binary	NA	0.0028603
**sm_b_2**	Binary	NA	-0.0047067
**sm_b:<NA>**	Binary	NA	-0.0001373
**cvevent_b**	Binary	NA	0.0085507
**cvevent_b:<NA>**	Binary	NA	0.0003433
**HTA_b**	Binary	NA	-0.0159594
**HTA_b:<NA>**	Binary	NA	0.0011471
**diab_b**	Binary	NA	0.0027625

Product of weights

In [156…

```
ch_sc <- mutate(ch_sc, ipw_totaldairy = ipw_totaldairy * ipcw,
                   ipw_ferm = ipw_ferm * ipcw,
                   ipw_nonferm = ipw_nonferm * ipcw,
                   ipw_fullfat= ipw_fullfat * ipcw,
                   ipw_nonfat= ipw_nonfat * ipcw,
                   ipw_sugdairy= ipw_sugdairy * ipcw)

threshold_99 <- quantile(ch_sc$ipw_totaldairy, 0.995)
ch_sc$ipw_totaldairy[ch_sc$ipw_totaldairy > threshold_99] <- threshold_99
plot(ch_sc$ipw_totaldairy)

threshold_99 <- quantile(ch_sc$ipw_ferm, 0.995)
ch_sc$ipw_ferm[ch_sc$ipw_ferm > threshold_99] <- threshold_99
plot(ch_sc$ipw_ferm)
```

```
threshold_99 <- quantile(ch_sc$ipw_nonferm, 0.995)
ch_sc$ipw_nonferm[ch_sc$ipw_nonferm > threshold_99] <- threshold_99
plot(ch_sc$ipw_nonferm)

threshold_99 <- quantile(ch_sc$ipw_fullfat, 0.995)
ch_sc$ipw_fullfat[ch_sc$ipw_fullfat > threshold_99] <- threshold_99
plot(ch_sc$ipw_fullfat)

threshold_99 <- quantile(ch_sc$ipw_nonfat, 0.995)
ch_sc$ipw_nonfat[ch_sc$ipw_nonfat > threshold_99] <- threshold_99
plot(ch_sc$ipw_nonfat)

threshold_99 <- quantile(ch_sc$ipw_sugdairy, 0.995)
ch_sc$ipw_sugdairy[ch_sc$ipw_sugdairy > threshold_99] <- threshold_99
plot(ch_sc$ipw_sugdairy)
```

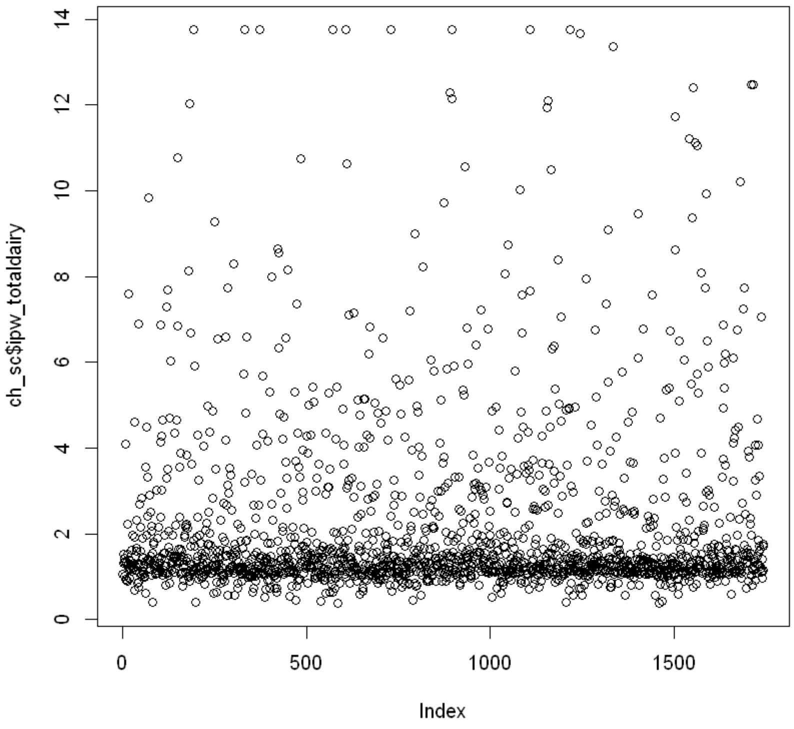

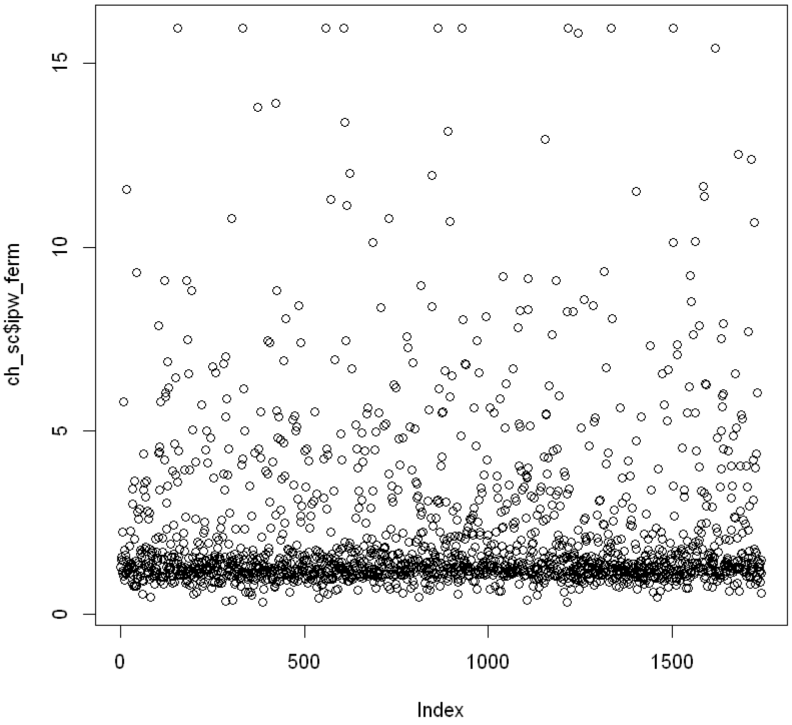
ch_sc$ipw_ferm
Index
0
5
10
15
0
500
1000
1500

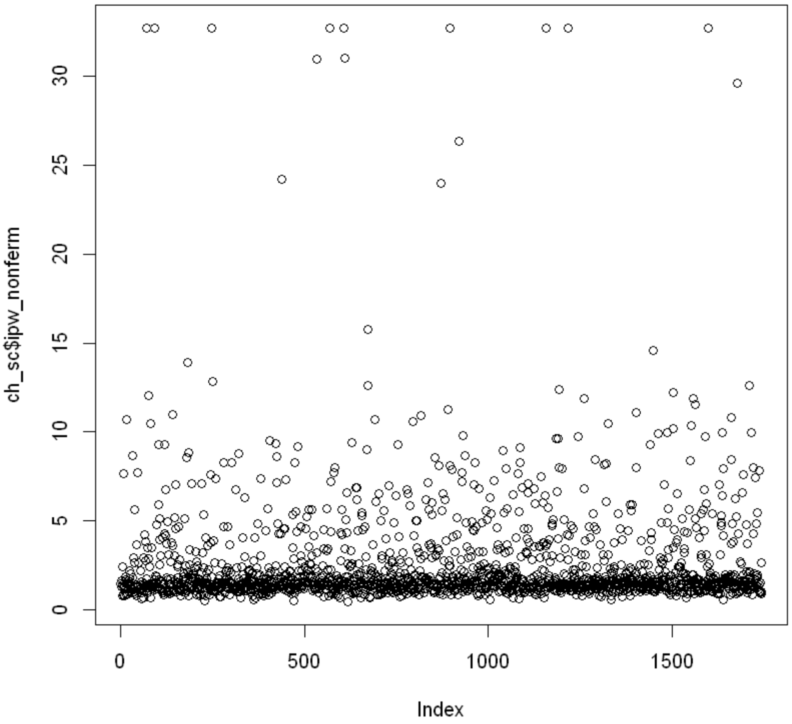
ch_sc$ipw_nonferm
Index
0
5
10
15
20
25
30
0
500
1000
1500

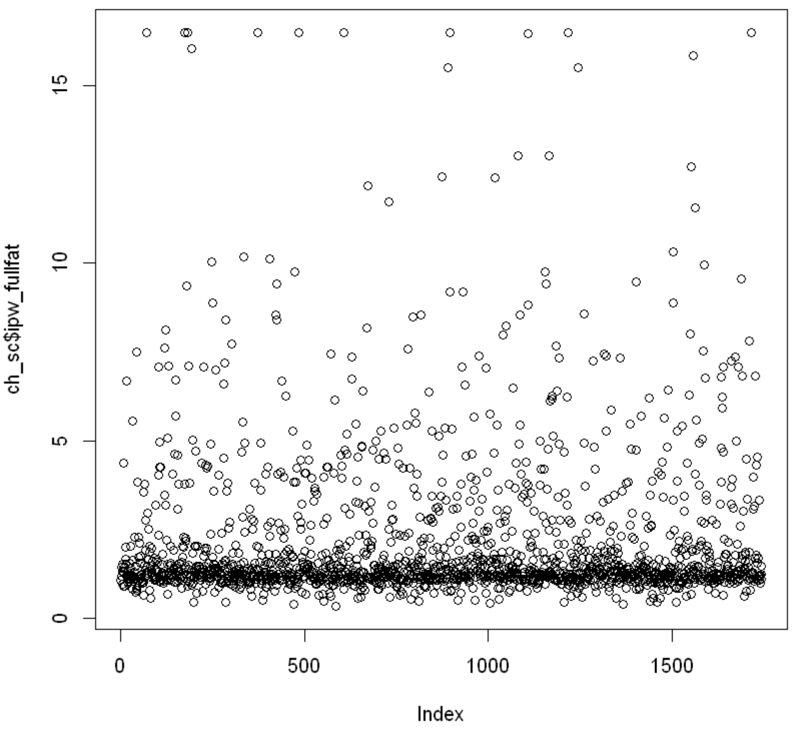
ch_sc$ipw_fullfat
Index
0
5
10
15
0
500
1000
1500

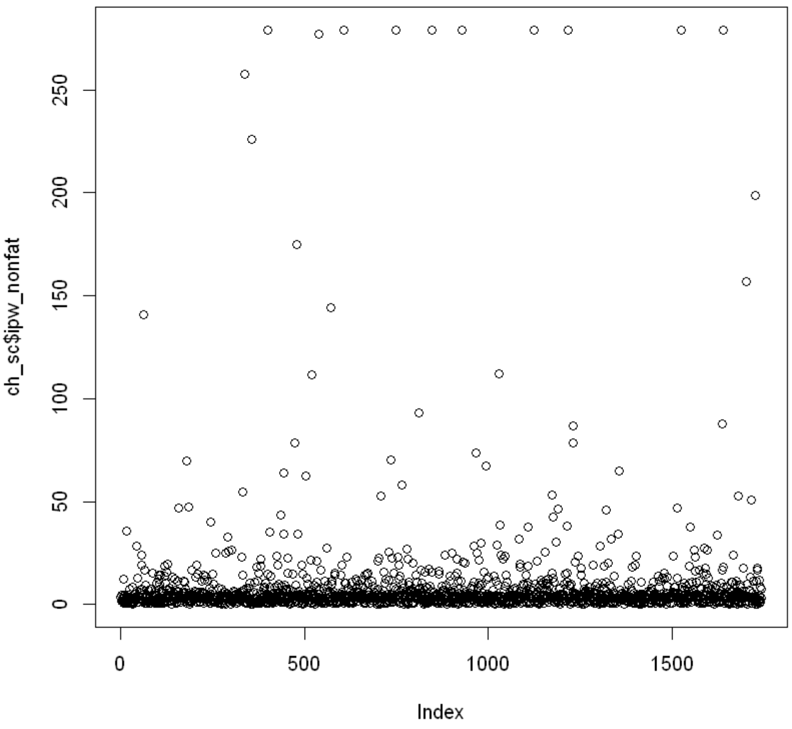
ch_sc$ipw_nonfat
Index
0
50
100
150
200
250
0
500
1000
1500

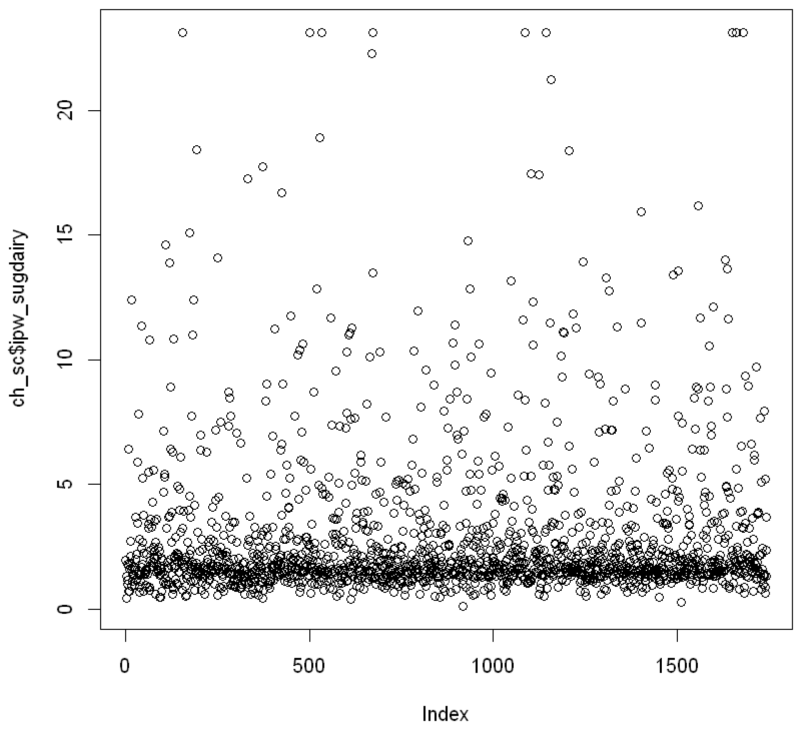

Balanced product weights

In [157…

```
#define  covariates
covariateNames <- c("veg_b","fru_b","sea_b",
                    "meat_b","eggs_b",
                    "grains_b","alcohol_b" ,
                    "sugary_b","fats_b",
                    "sex","age_cat","edu","sm_b","cvevent_b",
                   "HTA_b","depre_b","famincome_b","diab_b", "pa_b", "bmi_cat", "o

designIPW <- svydesign(ids=~1,weights=~ipw_totaldairy,data=ch_sc) #design with wei
designBase <- svydesign(ids=~1,weights=~1,data=ch_sc)

#create empty balance table
balanceTableIPW <- data.frame() #storage

#loop through covariates
for (var in 1:length(covariateNames)) {

  #formula with treatment dose as a function of a single covariate
balanceFormula <- paste("total_dairy_b ~",covariateNames[var],sep="")

#regress dose on covariate without weights
maxEffBaseline <- max(abs(coef(svyglm(balanceFormula,designBase))[-1]))
#regress dose on covariate with IPW
maxEffIPW <- max(abs(coef(svyglm(balanceFormula,designIPW))[-1]))

balanceTableIPW <- rbind(balanceTableIPW,c(var,maxEffBaseline,maxEffIPW))

} #close loop

#put  variable names on table
names(balanceTableIPW) <- c("variable","coefBaseline","coefIPW")
balanceTableIPW$variable <- covariateNames

#standardize coefficients with respect to sd of outcome
```

```
balanceTableIPW$coefBaseline <- balanceTableIPW$coefBaseline/
    sqrt(coef(svyvar(~total_dairy_b,designBase)))
balanceTableIPW$coefIPW <- balanceTableIPW$coefIPW/
  sqrt(coef(svyvar(~total_dairy_b,designIPW)))

#save balance table
write.csv(balanceTableIPW,file="covariatebalanceFinal.csv",row.names=F)
```

CDR

In [158...

```
#See how many people has CDR and transform as a factor

ch_sc$CDR_b <- as.factor(ch_sc$CDR_b)
ch_sc$F2CDR <- as.factor(ch_sc$F2CDR)
ch_sc$F2CDR <- ifelse(ch_sc$F2CDR == "0.0", 0,
                     ifelse(ch_sc$F2CDR == "0.5", 1, NA))
table(ch_sc$F2CDR)
```

```
  0   1
487 426
```

Total dairy

In [159...

```
msm.w <- geeglm(F2CDR ~ total_dairy_b, data=ch_sc, weights=ipw_totaldairy, id=pt,
                corstr="independence")
summary(msm.w)

beta <- coef(msm.w)
SE <- coef(summary(msm.w))[,2]
lcl <- beta-qnorm(0.975)*SE
ucl <- beta+qnorm(0.975)*SE
cdra <- cbind(beta*100, lcl*100, ucl*100)
```

```
Call:
geeglm(formula = F2CDR ~ total_dairy_b, data = ch_sc, weights = ipw_totaldairy,
    id = pt, corstr = "independence")

 Coefficients:
              Estimate  Std.err   Wald Pr(>|W|)
(Intercept)   0.445634 0.034123 170.55   <2e-16 ***
total_dairy_b 0.000152 0.000102   2.21     0.14
---
Signif. codes:  0 '***' 0.001 '**' 0.01 '*' 0.05 '.' 0.1 ' ' 1

Correlation structure = independence
Estimated Scale Parameters:

            Estimate Std.err
(Intercept)    0.249 0.00137
Number of clusters:   913  Maximum cluster size: 1
```

Fermented dairy

In [160...

```
msm.w <- geeglm(F2CDR ~ ferm_dairy_b, data=ch_sc, weights=ipw_ferm, id=pt,
                corstr="independence")
summary(msm.w)

beta <- coef(msm.w)
SE <- coef(summary(msm.w))[,2]
lcl <- beta-qnorm(0.975)*SE
ucl <- beta+qnorm(0.975)*SE
cdrb <- cbind(beta*100, lcl*100, ucl*100)
```

```
Call:
geeglm(formula = F2CDR ~ ferm_dairy_b, data = ch_sc, weights = ipw_ferm,
    id = pt, corstr = "independence")

 Coefficients:
             Estimate  Std.err   Wald Pr(>|W|)
(Intercept)  0.471312 0.035088 180.43   <2e-16 ***
ferm_dairy_b 0.000110 0.000147   0.56     0.46
---
Signif. codes:  0 '***' 0.001 '**' 0.01 '*' 0.05 '.' 0.1 ' ' 1

Correlation structure = independence
Estimated Scale Parameters:

            Estimate  Std.err
(Intercept)     0.25 0.000947
Number of clusters:   913  Maximum cluster size: 1
```

Non fermented dairy

In [161…

```
msm.w <- geeglm(F2CDR ~ nonferm_dairy_b, data=ch_sc, weights=ipw_nonferm, id=pt,
                corstr="independence")
summary(msm.w)

beta <- coef(msm.w)
SE <- coef(summary(msm.w))[,2]
lcl <- beta-qnorm(0.975)*SE
ucl <- beta+qnorm(0.975)*SE
cdrc <-cbind(beta*100, lcl*100, ucl*100)
```

```
Call:
geeglm(formula = F2CDR ~ nonferm_dairy_b, data = ch_sc, weights = ipw_nonferm,
    id = pt, corstr = "independence")

 Coefficients:
                Estimate  Std.err Wald Pr(>|W|)
(Intercept)     4.86e-01 3.42e-02  202   <2e-16 ***
nonferm_dairy_b 1.04e-05 1.87e-04    0     0.96
---
Signif. codes:  0 '***' 0.001 '**' 0.01 '*' 0.05 '.' 0.1 ' ' 1

Correlation structure = independence
Estimated Scale Parameters:

            Estimate  Std.err
(Intercept)     0.25 0.000727
Number of clusters:   913  Maximum cluster size: 1
```

Fullfat dairy

In [162…

```
msm.w <- geeglm(F2CDR ~ fullfat_dairy_b, data=ch_sc, weights=ipw_fullfat, id=pt,
                corstr="independence")
summary(msm.w)

beta <- coef(msm.w)
SE <- coef(summary(msm.w))[,2]
lcl <- beta-qnorm(0.975)*SE
ucl <- beta+qnorm(0.975)*SE
cdrd <- cbind(beta*100, lcl*100, ucl*100)
```

```
Call:
geeglm(formula = F2CDR ~ fullfat_dairy_b, data = ch_sc, weights = ipw_fullfat,
    id = pt, corstr = "independence")

 Coefficients:
                Estimate  Std.err  Wald Pr(>|W|)
(Intercept)     0.429918 0.033383 165.8   <2e-16 ***
fullfat_dairy_b 0.000235 0.000116   4.1    0.043 *
---
Signif. codes:  0 '***' 0.001 '**' 0.01 '*' 0.05 '.' 0.1 ' ' 1

Correlation structure = independence
Estimated Scale Parameters:

            Estimate Std.err
(Intercept)    0.248  0.0019
Number of clusters:   913  Maximum cluster size: 1
```

Non fat dairy

In [163…

```
msm.w <- geeglm(F2CDR ~ nonfat_dairy_b, data=ch_sc, weights=ipw_nonfat, id=pt,
                corstr="independence")
summary(msm.w)

beta <- coef(msm.w)
SE <- coef(summary(msm.w))[,2]
lcl <- beta-qnorm(0.975)*SE
ucl <- beta+qnorm(0.975)*SE
cdre <-cbind(beta*100, lcl*100, ucl*100)
```

```
Call:
geeglm(formula = F2CDR ~ nonfat_dairy_b, data = ch_sc, weights = ipw_nonfat,
    id = pt, corstr = "independence")

 Coefficients:
                Estimate   Std.err  Wald Pr(>|W|)
(Intercept)     0.513206  0.060713 71.45   <2e-16 ***
nonfat_dairy_b -0.000785  0.000421  3.48    0.062 .
---
Signif. codes:  0 '***' 0.001 '**' 0.01 '*' 0.05 '.' 0.1 ' ' 1

Correlation structure = independence
Estimated Scale Parameters:

            Estimate Std.err
(Intercept)    0.249 0.00169
Number of clusters:   913  Maximum cluster size: 1
```

Sugary dairy

In [164…

```
msm.w <- geeglm(F2CDR ~ sugar_dairy_b, data=ch_sc, weights=ipw_sugdairy, id=pt,
                corstr="independence")
summary(msm.w)

beta <- coef(msm.w)
SE <- coef(summary(msm.w))[,2]
lcl <- beta-qnorm(0.975)*SE
ucl <- beta+qnorm(0.975)*SE
cdrf <- cbind(beta*100, lcl*100, ucl*100)
```

```
Call:
geeglm(formula = F2CDR ~ sugar_dairy_b, data = ch_sc, weights = ipw_sugdairy,
    id = pt, corstr = "independence")

 Coefficients:
              Estimate  Std.err   Wald Pr(>|W|)
(Intercept)   0.454168 0.026340 297.31   <2e-16 ***
sugar_dairy_b 0.000440 0.000186   5.61    0.018 *
---
Signif. codes:  0 '***' 0.001 '**' 0.01 '*' 0.05 '.' 0.1 ' ' 1

Correlation structure = independence
Estimated Scale Parameters:

            Estimate Std.err
(Intercept)    0.248 0.00207
Number of clusters:   913  Maximum cluster size: 1
```

SCD

In [165…

```
#####################################
##########TOTAL DAIRY################
#####################################

msm.w <- geeglm(F2SCD_yn ~ total_dairy_b, data=ch_sc, weights=ipw_totaldairy, id=p
                corstr="independence")
summary(msm.w)

beta <- coef(msm.w)
SE <- coef(summary(msm.w))[,2]
lcl <- beta-qnorm(0.975)*SE
ucl <- beta+qnorm(0.975)*SE
scda <-cbind(beta*100, lcl*100, ucl*100)

#####################################
##########FERMENTED DAIRY############
#####################################

msm.w <- geeglm(F2SCD_yn ~ ferm_dairy_b, data=ch_sc, weights=ipw_ferm, id=pt,
                corstr="independence")
summary(msm.w)

beta <- coef(msm.w)
SE <- coef(summary(msm.w))[,2]
lcl <- beta-qnorm(0.975)*SE
ucl <- beta+qnorm(0.975)*SE
scdb <-cbind(beta*100, lcl*100, ucl*100)

#####################################
##########NON FERMENTED DAIRY########
#####################################

msm.w <- geeglm(F2SCD_yn ~ nonferm_dairy_b, data=ch_sc, weights=ipw_nonferm, id=pt
                corstr="independence")
summary(msm.w)

beta <- coef(msm.w)
SE <- coef(summary(msm.w))[,2]
lcl <- beta-qnorm(0.975)*SE
ucl <- beta+qnorm(0.975)*SE
scdc <-cbind(beta*100, lcl*100, ucl*100)

#####################################
```

```
##########FULL FAT DAIRY#############
######################################

msm.w <- geeglm(F2SCD_yn ~ fullfat_dairy_b, data=ch_sc, weights=ipw_fullfat, id=pt
                corstr="independence")
summary(msm.w)

beta <- coef(msm.w)
SE <- coef(summary(msm.w))[,2]
lcl <- beta-qnorm(0.975)*SE
ucl <- beta+qnorm(0.975)*SE
scdd <-cbind(beta*100, lcl*100, ucl*100)
######################################
###########NON FAT DAIRY#############
######################################

msm.w <- geeglm(F2SCD_yn ~ nonferm_dairy_b, data=ch_sc, weights=ipw_nonfat, id=pt,
                corstr="independence")
summary(msm.w)

beta <- coef(msm.w)
SE <- coef(summary(msm.w))[,2]
lcl <- beta-qnorm(0.975)*SE
ucl <- beta+qnorm(0.975)*SE
scde <-cbind(beta*100, lcl*100, ucl*100)
######################################
############SUGARY DAIRY#############
######################################

msm.w <- geeglm(F2SCD_yn ~ sugar_dairy_b, data=ch_sc, weights=ipw_sugdairy, id=pt,
                corstr="independence")
summary(msm.w)

beta <- coef(msm.w)
SE <- coef(summary(msm.w))[,2]
lcl <- beta-qnorm(0.975)*SE
ucl <- beta+qnorm(0.975)*SE
scdf <-cbind(beta*100, lcl*100, ucl*100)
```

```
Call:
geeglm(formula = F2SCD_yn ~ total_dairy_b, data = ch_sc, weights = ipw_totaldairy,
    id = pt, corstr = "independence")

 Coefficients:
              Estimate  Std.err Wald Pr(>|W|)
(Intercept)   1.71e-01 2.96e-02 33.3  7.8e-09 ***
total_dairy_b 8.82e-05 9.85e-05  0.8     0.37
---
Signif. codes:  0 '***' 0.001 '**' 0.01 '*' 0.05 '.' 0.1 ' ' 1

Correlation structure = independence
Estimated Scale Parameters:

            Estimate Std.err
(Intercept)    0.154  0.0106
Number of clusters:   972  Maximum cluster size: 1
```

```
Call:
geeglm(formula = F2SCD_yn ~ ferm_dairy_b, data = ch_sc, weights = ipw_ferm,
    id = pt, corstr = "independence")

 Coefficients:
             Estimate  Std.err  Wald Pr(>|W|)
(Intercept)  1.91e-01 3.03e-02 39.85  2.7e-10 ***
ferm_dairy_b 1.35e-05 1.31e-04  0.01     0.92
---
Signif. codes:  0 '***' 0.001 '**' 0.01 '*' 0.05 '.' 0.1 ' ' 1

Correlation structure = independence
Estimated Scale Parameters:

            Estimate Std.err
(Intercept)    0.156  0.0115
Number of clusters:   972  Maximum cluster size: 1
Call:
geeglm(formula = F2SCD_yn ~ nonferm_dairy_b, data = ch_sc, weights = ipw_nonferm,
    id = pt, corstr = "independence")

 Coefficients:
                Estimate  Std.err  Wald Pr(>|W|)
(Intercept)     0.164932 0.020685 63.58  1.6e-15 ***
nonferm_dairy_b 0.000187 0.000133  1.96     0.16
---
Signif. codes:  0 '***' 0.001 '**' 0.01 '*' 0.05 '.' 0.1 ' ' 1

Correlation structure = independence
Estimated Scale Parameters:

            Estimate Std.err
(Intercept)    0.146  0.0111
Number of clusters:   972  Maximum cluster size: 1
Call:
geeglm(formula = F2SCD_yn ~ fullfat_dairy_b, data = ch_sc, weights = ipw_fullfat,
    id = pt, corstr = "independence")

 Coefficients:
                Estimate  Std.err  Wald Pr(>|W|)
(Intercept)     0.163117 0.027556 35.04  3.2e-09 ***
fullfat_dairy_b 0.000110 0.000104  1.12     0.29
---
Signif. codes:  0 '***' 0.001 '**' 0.01 '*' 0.05 '.' 0.1 ' ' 1

Correlation structure = independence
Estimated Scale Parameters:

            Estimate Std.err
(Intercept)     0.15  0.0107
Number of clusters:   972  Maximum cluster size: 1
```

```
Call:
geeglm(formula = F2SCD_yn ~ nonferm_dairy_b, data = ch_sc, weights = ipw_nonfat,
    id = pt, corstr = "independence")

 Coefficients:
                Estimate  Std.err  Wald Pr(>|W|)
(Intercept)     0.171389 0.035877 22.82  1.8e-06 ***
nonferm_dairy_b 0.000460 0.000359  1.64      0.2
---
Signif. codes:  0 '***' 0.001 '**' 0.01 '*' 0.05 '.' 0.1 ' ' 1

Correlation structure = independence
Estimated Scale Parameters:

            Estimate Std.err
(Intercept)    0.164  0.0218
Number of clusters:   972  Maximum cluster size: 1
Call:
geeglm(formula = F2SCD_yn ~ sugar_dairy_b, data = ch_sc, weights = ipw_sugdairy,
    id = pt, corstr = "independence")

 Coefficients:
              Estimate  Std.err  Wald Pr(>|W|)
(Intercept)   0.163809 0.019811 68.37   <2e-16 ***
sugar_dairy_b 0.000239 0.000221  1.17     0.28
---
Signif. codes:  0 '***' 0.001 '**' 0.01 '*' 0.05 '.' 0.1 ' ' 1

Correlation structure = independence
Estimated Scale Parameters:

            Estimate Std.err
(Intercept)    0.146  0.0102
Number of clusters:   972  Maximum cluster size: 1
```

Memory

In [166…

```
#####################################
##########TOTAL DAIRY################
#####################################

msm.w <- geeglm(F2memory_cat ~ total_dairy_b, data=ch_sc, weights=ipw_totaldairy, 
                corstr="independence")
summary(msm.w)

beta <- coef(msm.w)
SE <- coef(summary(msm.w))[,2]
lcl <- beta-qnorm(0.975)*SE
ucl <- beta+qnorm(0.975)*SE
mema <- cbind(beta*100, lcl*100, ucl*100)

#####################################
##########FERMENTED DAIRY############
#####################################

msm.w <- geeglm(F2memory_cat ~ ferm_dairy_b, data=ch_sc, weights=ipw_ferm, id=pt,
                corstr="independence")
summary(msm.w)

beta <- coef(msm.w)
SE <- coef(summary(msm.w))[,2]
lcl <- beta-qnorm(0.975)*SE
ucl <- beta+qnorm(0.975)*SE
```

```
memb <- cbind(beta*100, lcl*100, ucl*100)

#####################################
##########NON FERMENTED DAIRY########
#####################################

msm.w <- geeglm(F2memory_cat ~ nonferm_dairy_b, data=ch_sc, weights=ipw_nonferm, i
                corstr="independence")
summary(msm.w)

beta <- coef(msm.w)
SE <- coef(summary(msm.w))[,2]
lcl <- beta-qnorm(0.975)*SE
ucl <- beta+qnorm(0.975)*SE
memc <- cbind(beta*100, lcl*100, ucl*100)

#####################################
##########FULL FAT DAIRY#############
#####################################

msm.w <- geeglm(F2memory_cat ~ fullfat_dairy_b, data=ch_sc, weights=ipw_fullfat, i
                corstr="independence")
summary(msm.w)

beta <- coef(msm.w)
SE <- coef(summary(msm.w))[,2]
lcl <- beta-qnorm(0.975)*SE
ucl <- beta+qnorm(0.975)*SE
memd <- cbind(beta*100, lcl*100, ucl*100)
#####################################
###########NON FAT DAIRY#############
#####################################

msm.w <- geeglm(F2memory_cat ~ nonferm_dairy_b, data=ch_sc, weights=ipw_nonfat, id
                corstr="independence")
summary(msm.w)

beta <- coef(msm.w)
SE <- coef(summary(msm.w))[,2]
lcl <- beta-qnorm(0.975)*SE
ucl <- beta+qnorm(0.975)*SE
meme <- cbind(beta*100, lcl*100, ucl*100)
#####################################
############SUGARY DAIRY#############
#####################################

msm.w <- geeglm(F2memory_cat ~ sugar_dairy_b, data=ch_sc, weights=ipw_sugdairy, id
                corstr="independence")
summary(msm.w)

beta <- coef(msm.w)
SE <- coef(summary(msm.w))[,2]
lcl <- beta-qnorm(0.975)*SE
ucl <- beta+qnorm(0.975)*SE
memf <- cbind(beta*100, lcl*100, ucl*100)
```

```
Call:
geeglm(formula = F2memory_cat ~ total_dairy_b, data = ch_sc,
    weights = ipw_totaldairy, id = pt, corstr = "independence")

 Coefficients:
              Estimate  Std.err  Wald Pr(>|W|)
(Intercept)   0.142546 0.030503 21.84    3e-06 ***
total_dairy_b 0.000197 0.000111  3.16    0.076 .
---
Signif. codes:  0 '***' 0.001 '**' 0.01 '*' 0.05 '.' 0.1 ' ' 1

Correlation structure = independence
Estimated Scale Parameters:

            Estimate Std.err
(Intercept)    0.151  0.0109
Number of clusters:   952  Maximum cluster size: 1
Call:
geeglm(formula = F2memory_cat ~ ferm_dairy_b, data = ch_sc, weights = ipw_ferm,
    id = pt, corstr = "independence")

 Coefficients:
             Estimate  Std.err  Wald Pr(>|W|)
(Intercept)  0.152173 0.030656 24.64  6.9e-07 ***
ferm_dairy_b 0.000262 0.000162  2.62     0.11
---
Signif. codes:  0 '***' 0.001 '**' 0.01 '*' 0.05 '.' 0.1 ' ' 1

Correlation structure = independence
Estimated Scale Parameters:

            Estimate Std.err
(Intercept)    0.153  0.0116
Number of clusters:   952  Maximum cluster size: 1
Call:
geeglm(formula = F2memory_cat ~ nonferm_dairy_b, data = ch_sc,
    weights = ipw_nonferm, id = pt, corstr = "independence")

 Coefficients:
                Estimate  Std.err  Wald Pr(>|W|)
(Intercept)     0.163521 0.020060 66.45  3.3e-16 ***
nonferm_dairy_b 0.000192 0.000132  2.09     0.15
---
Signif. codes:  0 '***' 0.001 '**' 0.01 '*' 0.05 '.' 0.1 ' ' 1

Correlation structure = independence
Estimated Scale Parameters:

            Estimate Std.err
(Intercept)    0.145  0.0109
Number of clusters:   952  Maximum cluster size: 1
```

```
Call:
geeglm(formula = F2memory_cat ~ fullfat_dairy_b, data = ch_sc,
    weights = ipw_fullfat, id = pt, corstr = "independence")

 Coefficients:
                Estimate  Std.err  Wald Pr(>|W|)
(Intercept)     0.139020 0.027066 26.38  2.8e-07 ***
fullfat_dairy_b 0.000209 0.000109  3.71    0.054 .
---
Signif. codes:  0 '***' 0.001 '**' 0.01 '*' 0.05 '.' 0.1 ' ' 1

Correlation structure = independence
Estimated Scale Parameters:

            Estimate Std.err
(Intercept)    0.146  0.0111
Number of clusters:   952  Maximum cluster size: 1
Call:
geeglm(formula = F2memory_cat ~ nonferm_dairy_b, data = ch_sc,
    weights = ipw_nonfat, id = pt, corstr = "independence")

 Coefficients:
                Estimate  Std.err  Wald Pr(>|W|)
(Intercept)     0.133548 0.030192 19.57  9.7e-06 ***
nonferm_dairy_b 0.000591 0.000514  1.32     0.25
---
Signif. codes:  0 '***' 0.001 '**' 0.01 '*' 0.05 '.' 0.1 ' ' 1

Correlation structure = independence
Estimated Scale Parameters:

            Estimate Std.err
(Intercept)    0.144  0.0234
Number of clusters:   952  Maximum cluster size: 1
Call:
geeglm(formula = F2memory_cat ~ sugar_dairy_b, data = ch_sc,
    weights = ipw_sugdairy, id = pt, corstr = "independence")

 Coefficients:
              Estimate  Std.err  Wald Pr(>|W|)
(Intercept)   0.185903 0.021126 77.44   <2e-16 ***
sugar_dairy_b 0.000123 0.000217  0.32     0.57
---
Signif. codes:  0 '***' 0.001 '**' 0.01 '*' 0.05 '.' 0.1 ' ' 1

Correlation structure = independence
Estimated Scale Parameters:

            Estimate Std.err
(Intercept)    0.156  0.0109
Number of clusters:   952  Maximum cluster size: 1
```

Verbal

In [167…

```
#####################################
#########TOTAL DAIRY################
#####################################

msm.w <- geeglm(F2verbal_cat ~ total_dairy_b, data=ch_sc, weights=ipw_totaldairy, 
                corstr="independence")
summary(msm.w)

beta <- coef(msm.w)
```

```
SE <- coef(summary(msm.w))[,2]
lcl <- beta-qnorm(0.975)*SE
ucl <- beta+qnorm(0.975)*SE
vera <- cbind(beta*100, lcl*100, ucl*100)

#####################################
##########FERMENTED DAIRY############
#####################################

msm.w <- geeglm(F2verbal_cat ~ ferm_dairy_b, data=ch_sc, weights=ipw_ferm, id=pt,
                corstr="independence")
summary(msm.w)

beta <- coef(msm.w)
SE <- coef(summary(msm.w))[,2]
lcl <- beta-qnorm(0.975)*SE
ucl <- beta+qnorm(0.975)*SE
verb <- cbind(beta*100, lcl*100, ucl*100)

#####################################
##########NON FERMENTED DAIRY########
#####################################

msm.w <- geeglm(F2verbal_cat ~ nonferm_dairy_b, data=ch_sc, weights=ipw_nonferm, i
                corstr="independence")
summary(msm.w)

beta <- coef(msm.w)
SE <- coef(summary(msm.w))[,2]
lcl <- beta-qnorm(0.975)*SE
ucl <- beta+qnorm(0.975)*SE
verc <- cbind(beta*100, lcl*100, ucl*100)

#####################################
##########FULL FAT DAIRY#############
#####################################

msm.w <- geeglm(F2verbal_cat ~ fullfat_dairy_b, data=ch_sc, weights=ipw_fullfat, i
                corstr="independence")
summary(msm.w)

beta <- coef(msm.w)
SE <- coef(summary(msm.w))[,2]
lcl <- beta-qnorm(0.975)*SE
ucl <- beta+qnorm(0.975)*SE
verd <- cbind(beta*100, lcl*100, ucl*100)
#####################################
###########NON FAT DAIRY#############
#####################################

msm.w <- geeglm(F2verbal_cat ~ nonferm_dairy_b, data=ch_sc, weights=ipw_nonfat, id
                corstr="independence")
summary(msm.w)

beta <- coef(msm.w)
SE <- coef(summary(msm.w))[,2]
lcl <- beta-qnorm(0.975)*SE
ucl <- beta+qnorm(0.975)*SE
vere <- cbind(beta*100, lcl*100, ucl*100)
#####################################
###########SUGARY DAIRY#############
#####################################

msm.w <- geeglm(F2verbal_cat ~ sugar_dairy_b, data=ch_sc, weights=ipw_sugdairy, id
```

```
                corstr="independence")
summary(msm.w)

beta <- coef(msm.w)
SE <- coef(summary(msm.w))[,2]
lcl <- beta-qnorm(0.975)*SE
ucl <- beta+qnorm(0.975)*SE
verf <- cbind(beta*100, lcl*100, ucl*100)
```

```
Call:
geeglm(formula = F2verbal_cat ~ total_dairy_b, data = ch_sc, 
    weights = ipw_totaldairy, id = pt, corstr = "independence")

 Coefficients:
              Estimate   Std.err   Wald Pr(>|W|)    
(Intercept)    7.65e-01  3.00e-02 648.07   <2e-16 ***
total_dairy_b -3.15e-05  9.44e-05   0.11     0.74    
---
Signif. codes:  0 '***' 0.001 '**' 0.01 '*' 0.05 '.' 0.1 ' ' 1

Correlation structure = independence 
Estimated Scale Parameters:

            Estimate Std.err
(Intercept)    0.183 0.00952
Number of clusters:   954  Maximum cluster size: 1 
Call:
geeglm(formula = F2verbal_cat ~ ferm_dairy_b, data = ch_sc, weights = ipw_ferm, 
    id = pt, corstr = "independence")

 Coefficients:
             Estimate   Std.err   Wald Pr(>|W|)    
(Intercept)   7.72e-01  2.81e-02 757.00   <2e-16 ***
ferm_dairy_b -6.84e-05  1.27e-04   0.29     0.59    
---
Signif. codes:  0 '***' 0.001 '**' 0.01 '*' 0.05 '.' 0.1 ' ' 1

Correlation structure = independence 
Estimated Scale Parameters:

            Estimate Std.err
(Intercept)    0.181    0.01
Number of clusters:   954  Maximum cluster size: 1 
Call:
geeglm(formula = F2verbal_cat ~ nonferm_dairy_b, data = ch_sc, 
    weights = ipw_nonferm, id = pt, corstr = "independence")

 Coefficients:
                Estimate   Std.err   Wald Pr(>|W|)    
(Intercept)      7.59e-01  2.52e-02 908.67   <2e-16 ***
nonferm_dairy_b -6.17e-05  1.64e-04   0.14     0.71    
---
Signif. codes:  0 '***' 0.001 '**' 0.01 '*' 0.05 '.' 0.1 ' ' 1

Correlation structure = independence 
Estimated Scale Parameters:

            Estimate Std.err
(Intercept)    0.185  0.0102
Number of clusters:   954  Maximum cluster size: 1 
```

```
Call:
geeglm(formula = F2verbal_cat ~ fullfat_dairy_b, data = ch_sc,
    weights = ipw_fullfat, id = pt, corstr = "independence")

 Coefficients:
                 Estimate   Std.err   Wald Pr(>|W|)
(Intercept)      7.77e-01  2.82e-02 761.89   <2e-16 ***
fullfat_dairy_b -5.37e-05  1.06e-04   0.26     0.61
---
Signif. codes:  0 '***' 0.001 '**' 0.01 '*' 0.05 '.' 0.1 ' ' 1

Correlation structure = independence
Estimated Scale Parameters:

            Estimate Std.err
(Intercept)    0.179 0.00981
Number of clusters:   954  Maximum cluster size: 1
Call:
geeglm(formula = F2verbal_cat ~ nonferm_dairy_b, data = ch_sc,
    weights = ipw_nonfat, id = pt, corstr = "independence")

 Coefficients:
                 Estimate   Std.err   Wald Pr(>|W|)
(Intercept)      0.807500  0.038006 451.41   <2e-16 ***
nonferm_dairy_b -0.000810  0.000471   2.96    0.086 .
---
Signif. codes:  0 '***' 0.001 '**' 0.01 '*' 0.05 '.' 0.1 ' ' 1

Correlation structure = independence
Estimated Scale Parameters:

            Estimate Std.err
(Intercept)    0.183  0.0198
Number of clusters:   954  Maximum cluster size: 1
Call:
geeglm(formula = F2verbal_cat ~ sugar_dairy_b, data = ch_sc,
    weights = ipw_sugdairy, id = pt, corstr = "independence")

 Coefficients:
              Estimate  Std.err    Wald Pr(>|W|)
(Intercept)   0.751570 0.022004 1166.67   <2e-16 ***
sugar_dairy_b 0.000191 0.000155    1.52     0.22
---
Signif. codes:  0 '***' 0.001 '**' 0.01 '*' 0.05 '.' 0.1 ' ' 1

Correlation structure = independence
Estimated Scale Parameters:

            Estimate Std.err
(Intercept)     0.18 0.00926
Number of clusters:   954  Maximum cluster size: 1
```

Stroop

In [168…

```
#####################################
#########TOTAL DAIRY################
#####################################

msm.w <- geeglm(F2stroop_cat ~ total_dairy_b, data=ch_sc, weights=ipw_totaldairy, 
                corstr="independence")
summary(msm.w)

beta <- coef(msm.w)
```

```
SE <- coef(summary(msm.w))[,2]
lcl <- beta-qnorm(0.975)*SE
ucl <- beta+qnorm(0.975)*SE
stra <- cbind(beta*100, lcl*100, ucl*100)

#####################################
##########FERMENTED DAIRY############
#####################################

msm.w <- geeglm(F2stroop_cat ~ ferm_dairy_b, data=ch_sc, weights=ipw_ferm, id=pt,
                corstr="independence")
summary(msm.w)

beta <- coef(msm.w)
SE <- coef(summary(msm.w))[,2]
lcl <- beta-qnorm(0.975)*SE
ucl <- beta+qnorm(0.975)*SE
strb <- cbind(beta*100, lcl*100, ucl*100)

#####################################
##########NON FERMENTED DAIRY########
#####################################

msm.w <- geeglm(F2stroop_cat ~ nonferm_dairy_b, data=ch_sc, weights=ipw_nonferm, i
                corstr="independence")
summary(msm.w)

beta <- coef(msm.w)
SE <- coef(summary(msm.w))[,2]
lcl <- beta-qnorm(0.975)*SE
ucl <- beta+qnorm(0.975)*SE
strc <- cbind(beta*100, lcl*100, ucl*100)

#####################################
##########FULL FAT DAIRY#############
#####################################

msm.w <- geeglm(F2stroop_cat ~ fullfat_dairy_b, data=ch_sc, weights=ipw_fullfat, i
                corstr="independence")
summary(msm.w)

beta <- coef(msm.w)
SE <- coef(summary(msm.w))[,2]
lcl <- beta-qnorm(0.975)*SE
ucl <- beta+qnorm(0.975)*SE
strd <- cbind(beta*100, lcl*100, ucl*100)
#####################################
###########NON FAT DAIRY#############
#####################################

msm.w <- geeglm(F2stroop_cat ~ nonferm_dairy_b, data=ch_sc, weights=ipw_nonfat, id
                corstr="independence")
summary(msm.w)

beta <- coef(msm.w)
SE <- coef(summary(msm.w))[,2]
lcl <- beta-qnorm(0.975)*SE
ucl <- beta+qnorm(0.975)*SE
stre <- cbind(beta*100, lcl*100, ucl*100)
#####################################
###########SUGARY DAIRY#############
#####################################

msm.w <- geeglm(F2stroop_cat ~ sugar_dairy_b, data=ch_sc, weights=ipw_sugdairy, id
```

```
                corstr="independence")
summary(msm.w)

beta <- coef(msm.w)
SE <- coef(summary(msm.w))[,2]
lcl <- beta-qnorm(0.975)*SE
ucl <- beta+qnorm(0.975)*SE
strf <- cbind(beta*100, lcl*100, ucl*100)
```

```
Call:
geeglm(formula = F2stroop_cat ~ total_dairy_b, data = ch_sc,
    weights = ipw_totaldairy, id = pt, corstr = "independence")

 Coefficients:
              Estimate  Std.err  Wald Pr(>|W|)
(Intercept)   3.09e-01 3.54e-02 76.16   <2e-16 ***
total_dairy_b 9.99e-05 1.16e-04  0.74     0.39
---
Signif. codes:  0 '***' 0.001 '**' 0.01 '*' 0.05 '.' 0.1 ' ' 1

Correlation structure = independence
Estimated Scale Parameters:

            Estimate Std.err
(Intercept)    0.221 0.00714
Number of clusters:   965  Maximum cluster size: 1
Call:
geeglm(formula = F2stroop_cat ~ ferm_dairy_b, data = ch_sc, weights = ipw_ferm,
    id = pt, corstr = "independence")

 Coefficients:
             Estimate  Std.err  Wald Pr(>|W|)
(Intercept)  0.297136 0.034674 73.44   <2e-16 ***
ferm_dairy_b 0.000212 0.000163  1.69     0.19
---
Signif. codes:  0 '***' 0.001 '**' 0.01 '*' 0.05 '.' 0.1 ' ' 1

Correlation structure = independence
Estimated Scale Parameters:

            Estimate Std.err
(Intercept)    0.219  0.0079
Number of clusters:   965  Maximum cluster size: 1
Call:
geeglm(formula = F2stroop_cat ~ nonferm_dairy_b, data = ch_sc,
    weights = ipw_nonferm, id = pt, corstr = "independence")

 Coefficients:
                 Estimate   Std.err   Wald Pr(>|W|)
(Intercept)      3.35e-01  3.13e-02 114.54   <2e-16 ***
nonferm_dairy_b -8.44e-05  1.65e-04   0.26     0.61
---
Signif. codes:  0 '***' 0.001 '**' 0.01 '*' 0.05 '.' 0.1 ' ' 1

Correlation structure = independence
Estimated Scale Parameters:

            Estimate Std.err
(Intercept)    0.221 0.00823
Number of clusters:   965  Maximum cluster size: 1
```

```
Call:
geeglm(formula = F2stroop_cat ~ fullfat_dairy_b, data = ch_sc,
    weights = ipw_fullfat, id = pt, corstr = "independence")

 Coefficients:
                Estimate  Std.err  Wald Pr(>|W|)
(Intercept)     0.303188 0.035220 74.10   <2e-16 ***
fullfat_dairy_b 0.000143 0.000128  1.25     0.26
---
Signif. codes:  0 '***' 0.001 '**' 0.01 '*' 0.05 '.' 0.1 ' ' 1

Correlation structure = independence
Estimated Scale Parameters:

            Estimate Std.err
(Intercept)    0.221 0.00757
Number of clusters:   965  Maximum cluster size: 1
Call:
geeglm(formula = F2stroop_cat ~ nonferm_dairy_b, data = ch_sc,
    weights = ipw_nonfat, id = pt, corstr = "independence")

 Coefficients:
                 Estimate   Std.err  Wald Pr(>|W|)
(Intercept)      0.353674  0.067774 27.23  1.8e-07 ***
nonferm_dairy_b -0.000345  0.000332  1.08      0.3
---
Signif. codes:  0 '***' 0.001 '**' 0.01 '*' 0.05 '.' 0.1 ' ' 1

Correlation structure = independence
Estimated Scale Parameters:

            Estimate Std.err
(Intercept)    0.219  0.0184
Number of clusters:   965  Maximum cluster size: 1
Call:
geeglm(formula = F2stroop_cat ~ sugar_dairy_b, data = ch_sc,
    weights = ipw_sugdairy, id = pt, corstr = "independence")

 Coefficients:
              Estimate  Std.err   Wald Pr(>|W|)
(Intercept)   0.317712 0.024493 168.26   <2e-16 ***
sugar_dairy_b 0.000298 0.000231   1.66      0.2
---
Signif. codes:  0 '***' 0.001 '**' 0.01 '*' 0.05 '.' 0.1 ' ' 1

Correlation structure = independence
Estimated Scale Parameters:

            Estimate Std.err
(Intercept)    0.222 0.00686
Number of clusters:   965  Maximum cluster size: 1
```

DO40

In [169…

```
#####################################
##########TOTAL DAIRY################
#####################################

msm.w <- geeglm(F2do40_cat ~ total_dairy_b, data=ch_sc, weights=ipw_totaldairy, id
                corstr="independence")
summary(msm.w)

beta <- coef(msm.w)
```

```
SE <- coef(summary(msm.w))[,2]
lcl <- beta-qnorm(0.975)*SE
ucl <- beta+qnorm(0.975)*SE
doa <- cbind(beta*100, lcl*100, ucl*100)

#####################################
##########FERMENTED DAIRY############
#####################################

msm.w <- geeglm(F2do40_cat ~ ferm_dairy_b, data=ch_sc, weights=ipw_ferm, id=pt,
                corstr="independence")
summary(msm.w)

beta <- coef(msm.w)
SE <- coef(summary(msm.w))[,2]
lcl <- beta-qnorm(0.975)*SE
ucl <- beta+qnorm(0.975)*SE
dob <- cbind(beta*100, lcl*100, ucl*100)

#####################################
##########NON FERMENTED DAIRY########
#####################################

msm.w <- geeglm(F2do40_cat ~ nonferm_dairy_b, data=ch_sc, weights=ipw_nonferm, id=
                corstr="independence")
summary(msm.w)

beta <- coef(msm.w)
SE <- coef(summary(msm.w))[,2]
lcl <- beta-qnorm(0.975)*SE
ucl <- beta+qnorm(0.975)*SE
doc <- cbind(beta*100, lcl*100, ucl*100)

#####################################
##########FULL FAT DAIRY#############
#####################################

msm.w <- geeglm(F2do40_cat ~ fullfat_dairy_b, data=ch_sc, weights=ipw_fullfat, id=
                corstr="independence")
summary(msm.w)

beta <- coef(msm.w)
SE <- coef(summary(msm.w))[,2]
lcl <- beta-qnorm(0.975)*SE
ucl <- beta+qnorm(0.975)*SE
dod <- cbind(beta*100, lcl*100, ucl*100)
#####################################
###########NON FAT DAIRY#############
#####################################

msm.w <- geeglm(F2do40_cat ~ nonfat_dairy_b, data=ch_sc, weights=ipw_nonfat, id=pt
                corstr="independence")
summary(msm.w)

beta <- coef(msm.w)
SE <- coef(summary(msm.w))[,2]
lcl <- beta-qnorm(0.975)*SE
ucl <- beta+qnorm(0.975)*SE
doe <- cbind(beta*100, lcl*100, ucl*100)

#####################################
###########SUGARY DAIRY#############
#####################################
```

```
msm.w <- geeglm(F2do40_cat ~ sugar_dairy_b, data=ch_sc, weights=ipw_sugdairy, id=pt
                corstr="independence")
summary(msm.w)

beta <- coef(msm.w)
SE <- coef(summary(msm.w))[,2]
lcl <- beta-qnorm(0.975)*SE
ucl <- beta+qnorm(0.975)*SE
dof <- cbind(beta*100, lcl*100, ucl*100)
```

```
Call:
geeglm(formula = F2do40_cat ~ total_dairy_b, data = ch_sc, weights = ipw_totaldair
y,
    id = pt, corstr = "independence")

 Coefficients:
               Estimate   Std.err  Wald Pr(>|W|)
(Intercept)    2.21e-01  3.69e-02 36.06  1.9e-09 ***
total_dairy_b -2.89e-05  1.19e-04  0.06     0.81
---
Signif. codes:  0 '***' 0.001 '**' 0.01 '*' 0.05 '.' 0.1 ' ' 1

Correlation structure = independence
Estimated Scale Parameters:

            Estimate Std.err
(Intercept)    0.169  0.0109
Number of clusters:   955  Maximum cluster size: 1
Call:
geeglm(formula = F2do40_cat ~ ferm_dairy_b, data = ch_sc, weights = ipw_ferm,
    id = pt, corstr = "independence")

 Coefficients:
              Estimate   Std.err  Wald Pr(>|W|)
(Intercept)   0.240873  0.032054 56.47  5.7e-14 ***
ferm_dairy_b -0.000218  0.000141  2.38     0.12
---
Signif. codes:  0 '***' 0.001 '**' 0.01 '*' 0.05 '.' 0.1 ' ' 1

Correlation structure = independence
Estimated Scale Parameters:

            Estimate Std.err
(Intercept)    0.164  0.0106
Number of clusters:   955  Maximum cluster size: 1
Call:
geeglm(formula = F2do40_cat ~ nonferm_dairy_b, data = ch_sc,
    weights = ipw_nonferm, id = pt, corstr = "independence")

 Coefficients:
                Estimate  Std.err  Wald Pr(>|W|)
(Intercept)     0.174643 0.028224 38.29  6.1e-10 ***
nonferm_dairy_b 0.000290 0.000172  2.85    0.092 .
---
Signif. codes:  0 '***' 0.001 '**' 0.01 '*' 0.05 '.' 0.1 ' ' 1

Correlation structure = independence
Estimated Scale Parameters:

            Estimate Std.err
(Intercept)    0.155  0.0137
Number of clusters:   955  Maximum cluster size: 1
```

```
Call:
geeglm(formula = F2do40_cat ~ fullfat_dairy_b, data = ch_sc,
    weights = ipw_fullfat, id = pt, corstr = "independence")

 Coefficients:
                 Estimate   Std.err  Wald Pr(>|W|)
(Intercept)      0.260439  0.042314 37.88  7.5e-10 ***
fullfat_dairy_b -0.000192  0.000144  1.79     0.18
---
Signif. codes:  0 '***' 0.001 '**' 0.01 '*' 0.05 '.' 0.1 ' ' 1

Correlation structure = independence
Estimated Scale Parameters:

            Estimate Std.err
(Intercept)    0.173  0.0117
Number of clusters:   955  Maximum cluster size: 1
Call:
geeglm(formula = F2do40_cat ~ nonfat_dairy_b, data = ch_sc, weights = ipw_nonfat,
    id = pt, corstr = "independence")

 Coefficients:
               Estimate  Std.err  Wald Pr(>|W|)
(Intercept)    0.297365 0.062162 22.88  1.7e-06 ***
nonfat_dairy_b 0.000101 0.000472  0.05     0.83
---
Signif. codes:  0 '***' 0.001 '**' 0.01 '*' 0.05 '.' 0.1 ' ' 1

Correlation structure = independence
Estimated Scale Parameters:

            Estimate Std.err
(Intercept)    0.209  0.0244
Number of clusters:   955  Maximum cluster size: 1
Call:
geeglm(formula = F2do40_cat ~ sugar_dairy_b, data = ch_sc, weights = ipw_sugdairy,
    id = pt, corstr = "independence")

 Coefficients:
               Estimate   Std.err Wald Pr(>|W|)
(Intercept)    2.03e-01  2.38e-02 72.5   <2e-16 ***
sugar_dairy_b -1.48e-05  2.36e-04  0.0     0.95
---
Signif. codes:  0 '***' 0.001 '**' 0.01 '*' 0.05 '.' 0.1 ' ' 1

Correlation structure = independence
Estimated Scale Parameters:

            Estimate Std.err
(Intercept)    0.161  0.0113
Number of clusters:   955  Maximum cluster size: 1
```

CERAD

In [170…

```
#####################################
#########TOTAL DAIRY################
#####################################

msm.w <- geeglm(F2CERAD_cat ~ total_dairy_b, data=ch_sc, weights=ipw_totaldairy, i
                corstr="independence")
summary(msm.w)

beta <- coef(msm.w)
```

```
SE <- coef(summary(msm.w))[,2]
lcl <- beta-qnorm(0.975)*SE
ucl <- beta+qnorm(0.975)*SE
cera <- cbind(beta*100, lcl*100, ucl*100)

#####################################
##########FERMENTED DAIRY############
#####################################

msm.w <- geeglm(F2CERAD_cat ~ ferm_dairy_b, data=ch_sc, weights=ipw_ferm, id=pt,
                corstr="independence")
summary(msm.w)

beta <- coef(msm.w)
SE <- coef(summary(msm.w))[,2]
lcl <- beta-qnorm(0.975)*SE
ucl <- beta+qnorm(0.975)*SE
cerb <- cbind(beta*100, lcl*100, ucl*100)

#####################################
##########NON FERMENTED DAIRY########
#####################################

msm.w <- geeglm(F2CERAD_cat ~ nonferm_dairy_b, data=ch_sc, weights=ipw_nonferm, id
                corstr="independence")
summary(msm.w)

beta <- coef(msm.w)
SE <- coef(summary(msm.w))[,2]
lcl <- beta-qnorm(0.975)*SE
ucl <- beta+qnorm(0.975)*SE
cerc <- cbind(beta*100, lcl*100, ucl*100)

#####################################
##########FULL FAT DAIRY#############
#####################################

msm.w <- geeglm(F2CERAD_cat ~ fullfat_dairy_b, data=ch_sc, weights=ipw_fullfat, id
                corstr="independence")
summary(msm.w)

beta <- coef(msm.w)
SE <- coef(summary(msm.w))[,2]
lcl <- beta-qnorm(0.975)*SE
ucl <- beta+qnorm(0.975)*SE
cerd <- cbind(beta*100, lcl*100, ucl*100)
#####################################
###########NON FAT DAIRY#############
#####################################

msm.w <- geeglm(F2CERAD_cat ~ nonferm_dairy_b, data=ch_sc, weights=ipw_nonfat, id=
                corstr="independence")
summary(msm.w)

beta <- coef(msm.w)
SE <- coef(summary(msm.w))[,2]
lcl <- beta-qnorm(0.975)*SE
ucl <- beta+qnorm(0.975)*SE
cere <- cbind(beta*100, lcl*100, ucl*100)
#####################################
###########SUGARY DAIRY#############
#####################################

msm.w <- geeglm(F2CERAD_cat ~ sugar_dairy_b, data=ch_sc, weights=ipw_sugdairy, id=
```

```
                corstr="independence")
summary(msm.w)

beta <- coef(msm.w)
SE <- coef(summary(msm.w))[,2]
lcl <- beta-qnorm(0.975)*SE
ucl <- beta+qnorm(0.975)*SE
cerf <- cbind(beta*100, lcl*100, ucl*100)
```

```
Call:
geeglm(formula = F2CERAD_cat ~ total_dairy_b, data = ch_sc, weights = ipw_totaldai
ry,
    id = pt, corstr = "independence")

 Coefficients:
              Estimate  Std.err  Wald Pr(>|W|)
(Intercept)   5.25e-01 3.73e-02 198.5   <2e-16 ***
total_dairy_b 8.67e-05 1.12e-04   0.6     0.44
---
Signif. codes:  0 '***' 0.001 '**' 0.01 '*' 0.05 '.' 0.1 ' ' 1

Correlation structure = independence
Estimated Scale Parameters:

            Estimate Std.err
(Intercept)    0.248 0.00188
Number of clusters:   980  Maximum cluster size: 1
Call:
geeglm(formula = F2CERAD_cat ~ ferm_dairy_b, data = ch_sc, weights = ipw_ferm,
    id = pt, corstr = "independence")

 Coefficients:
             Estimate  Std.err   Wald Pr(>|W|)
(Intercept)  0.522339 0.035273 219.29   <2e-16 ***
ferm_dairy_b 0.000113 0.000154   0.54     0.46
---
Signif. codes:  0 '***' 0.001 '**' 0.01 '*' 0.05 '.' 0.1 ' ' 1

Correlation structure = independence
Estimated Scale Parameters:

            Estimate Std.err
(Intercept)    0.248 0.00172
Number of clusters:   980  Maximum cluster size: 1
Call:
geeglm(formula = F2CERAD_cat ~ nonferm_dairy_b, data = ch_sc,
    weights = ipw_nonferm, id = pt, corstr = "independence")

 Coefficients:
                Estimate  Std.err   Wald Pr(>|W|)
(Intercept)     0.513413 0.033487 235.07   <2e-16 ***
nonferm_dairy_b 0.000214 0.000181   1.41     0.24
---
Signif. codes:  0 '***' 0.001 '**' 0.01 '*' 0.05 '.' 0.1 ' ' 1

Correlation structure = independence
Estimated Scale Parameters:

            Estimate Std.err
(Intercept)    0.249 0.00126
Number of clusters:   980  Maximum cluster size: 1
```

```
Call:
geeglm(formula = F2CERAD_cat ~ fullfat_dairy_b, data = ch_sc,
    weights = ipw_fullfat, id = pt, corstr = "independence")

 Coefficients:
                Estimate  Std.err   Wald Pr(>|W|)
(Intercept)     5.58e-01 3.73e-02 223.60   <2e-16 ***
fullfat_dairy_b 9.84e-06 1.29e-04   0.01     0.94
---
Signif. codes:  0 '***' 0.001 '**' 0.01 '*' 0.05 '.' 0.1 ' ' 1

Correlation structure = independence
Estimated Scale Parameters:

            Estimate Std.err
(Intercept)    0.246 0.00265
Number of clusters:   980  Maximum cluster size: 1
Call:
geeglm(formula = F2CERAD_cat ~ nonferm_dairy_b, data = ch_sc,
    weights = ipw_nonfat, id = pt, corstr = "independence")

 Coefficients:
                 Estimate   Std.err  Wald Pr(>|W|)
(Intercept)      0.604985  0.060235 100.9   <2e-16 ***
nonferm_dairy_b -0.000448  0.000428   1.1      0.3
---
Signif. codes:  0 '***' 0.001 '**' 0.01 '*' 0.05 '.' 0.1 ' ' 1

Correlation structure = independence
Estimated Scale Parameters:

            Estimate Std.err
(Intercept)    0.243 0.00836
Number of clusters:   980  Maximum cluster size: 1
Call:
geeglm(formula = F2CERAD_cat ~ sugar_dairy_b, data = ch_sc, weights = ipw_sugdair
y,
    id = pt, corstr = "independence")

 Coefficients:
              Estimate  Std.err   Wald Pr(>|W|)
(Intercept)   5.48e-01 2.61e-02 439.55   <2e-16 ***
sugar_dairy_b 2.04e-05 2.14e-04   0.01     0.92
---
Signif. codes:  0 '***' 0.001 '**' 0.01 '*' 0.05 '.' 0.1 ' ' 1

Correlation structure = independence
Estimated Scale Parameters:

            Estimate Std.err
(Intercept)    0.248  0.0021
Number of clusters:   980  Maximum cluster size: 1
```

In [171…

```
totaleffects <- data.frame(c(cdra[2,],cdrb[2,],cdrc[2,],cdrd[2,], cdre[2,],cdrf[2,
                        c(scda[2,],scdb[2,],scdc[2,],scdd[2,],scde[2,],scdf[2,]),
                        c(mema[2,],memb[2,],memc[2,],memd[2,],meme[2,],memf[2,]),
                        c(vera[2,],verb[2,],verc[2,],verd[2,],vere[2,],verf[2,]),
                        c(stra[2,],strb[2,],strc[2,],strd[2,],stre[2,],strf[2,]),
                        c(doa[2,],dob[2,],doc[2,],dod[2,],doe[2,],dof[2,]),
                        c(cera[2,],cerb[2,],cerc[2,],cerd[2,],cere[2,],cerf[2,]))
write.csv(totaleffects, "totaleffects.csv")
```

7. Sensitivy analysis for total effects

New selected dataset

In [172…

```
ch_sensi <- ch_sc %>%
        filter(!is.na(MMSE_b)) %>%
        filter(age_b > 55) %>% #<55 years old at baseline (n=0)
        filter(futime_F2 > 3)%>%#baseline dietary assessment at least 3 years befo
        filter(MMSE_b > 23)%>% ## n=9
        filter(totalcal_b > 500 & totalcal_b < 4200 & F2totalcal > 500 & F2totalca
        mutate(totalcal_avg = (totalcal_b + F2totalcal)/2,
                veg_avg = (F2veg+veg_b)/2,
                fru_avg  = (F2fru +fru_b )/2,
                sea_avg  = (F2sea +sea_b )/2,
                meat_avg  = (F2meat +meat_b )/2,
                eggs_avg  = (F2eggs +eggs_b )/2,
                grains_avg  = (F2grains +grains_b )/2,
                alcohol_avg = (F2alcohol+alcohol_b)/2,
                sugary_avg  = (F2sugary +sugary_b )/2,
                fats_avg = (F2fats+fats_b)/2,
                total_dairy_avg  = (F2total_dairy +total_dairy_b) /2,
                ferm_dairy_avg  = (F2ferm_dairy +ferm_dairy_b) /2,
                nonferm_dairy_avg  = (F2nonferm_dairy +nonferm_dairy_b) /2,
                fullfat_dairy_avg  = (F2fullfat_dairy +fullfat_dairy_b) /2,
                nonfat_dairy_avg = (F2nonfat_dairy+nonfat_dairy_b)/2,
                sugar_dairy_avg = (F2sugar_dairy+sugar_dairy_b)/2,
              nonsug_dairy_avg = (F2nonsug_dairy+nonsug_dairy_b)/2)%>%
        filter(!is.na(total_dairy_avg))

ch_sensi #n= 883

ch_sensi$F3CDR <- as.numeric(ch_sensi$F3CDR)
ch_sensi$F3SCD_yn <- as.numeric(ch_sensi$F3SCD_yn)
```

A data.

pt	sex	datbirth	datexam	bthpl_dem	ethori_self	edtyp	edlv	mrtsts	sclhlp	⋯	veg_a[illegible]
<int>	<int>	<date>	<date>	<chr>	<chr>	<int>	<int>	<int>	<int>	⋯	<db[illegible]
6	0	1943-01-30	2003-07-08	SZ	W	1	12	3	0	⋯	2[illegible]
7	0	1933-03-26	2003-07-04	EY	W	0	8	0	1	⋯	1[illegible]
20	0	1943-05-26	2003-07-16	SZ	W	3	14	2	0	⋯	1[illegible]
24	1	1936-09-16	2003-07-14	PD	W	3	50	2	1	⋯	1.[illegible]
25	0	1943-12-28	2003-08-28	SZ	W	1	12	3	0	⋯	1[illegible]
31	0	1948-03-10	2003-07-23	EG	W	2	24	2	0	⋯	2[illegible]
46	0	1942-11-07	2003-07-31	SZ	W	0	10	0	0	⋯	1[illegible]
48	1	1947-05-05	2003-08-13	SZ	W	2	16	2	0	⋯	1.[illegible]
51	0	1948-10-16	2003-09-01	SZ	W	1	14	NA	0	⋯	2.[illegible]
55	0	1943-12-26	2003-09-09	FR	W	2	18	0	0	⋯	3[illegible]
56	1	1939-01-01	2003-08-04	SZ	W	3	12	0	1	⋯	1[illegible]
68	0	1940-07-10	2003-08-05	SZ	W	0	10	3	0	⋯	1.[illegible]
72	0	1941-06-09	2003-08-08	RM	W	2	18	0	0	⋯	1[illegible]
87	1	1943-04-30	2003-08-12	SZ	W	3	15	0	0	⋯	2.[illegible]
102	0	1943-11-22	2003-08-25	SZ	W	1	15	2	0	⋯	1[illegible]
103	0	1946-02-04	2003-08-19	SZ	W	3	15	0	0	⋯	2[illegible]
112	1	1929-11-24	2003-09-02	SZ	W	0	10	0	1	⋯	1.[illegible]
121	1	1945-07-17	2003-08-25	FR	W	1	13	0	0	⋯	1[illegible]
130	1	1943-09-06	2003-08-26	CT	X	1	12	2	1	⋯	2[illegible]
144	0	1948-09-25	2003-09-19	SZ	W	3	16	0	0	⋯	3[illegible]
157	0	1943-05-06	2003-09-12	SZ	W	1	10	2	1	⋯	1[illegible]
167	1	1944-02-25	2003-09-17	SZ	W	2	18	0	0	⋯	1[illegible]

pt	sex	datbirth	datexam	bthpl_dem	ethori_self	edtyp	edlv	mrtsts	sclhlp	⋯	veg_a[illegible]
<int>	<int>	<date>	<date>	<chr>	<chr>	<int>	<int>	<int>	<int>	⋯	<db[illegible]
170	1	1940-06-23	2003-09-04	SZ	W	2	19	0	0	⋯	1[illegible]
175	0	1949-03-03	2003-10-08	SZ	W	1	12	2	0	⋯	4[illegible]
182	0	1943-07-12	2003-09-02	FR	W	2	14	0	0	⋯	1[illegible]
186	0	1942-05-02	2003-09-01	SZ	W	1	12	0	0	⋯	1[illegible]
191	0	1945-02-03	2003-09-19	HL	W	3	15	0	0	⋯	1[illegible]
203	1	1945-03-31	2003-09-08	SZ	W	3	14	2	0	⋯	5[illegible]
206	0	1943-09-30	2003-09-19	SZ	W	0	9	0	0	⋯	1[illegible]
208	0	1941-05-04	2003-09-23	SZ	W	0	9	0	0	⋯	2[illegible]
⋮	⋮	⋮	⋮	⋮	⋮	⋮	⋮	⋮	⋮	⋱	
6607	0	1945-12-02	2006-03-24	SZ	W	1	12	0	0	⋯	143[illegible]
6611	0	1941-06-10	2006-03-16	IT	W	0	9	0	1	⋯	282[illegible]
6631	1	1944-09-04	2006-04-04	SZ	W	1	11	2	0	⋯	93[illegible]
6632	0	1932-11-07	2006-04-03	SZ	W	2	15	0	0	⋯	173[illegible]
6650	1	1950-04-12	2006-04-28	IT	W	4	17	0	0	⋯	195[illegible]
6661	0	1946-08-27	2006-04-07	FR	W	2	14	NA	0	⋯	230[illegible]
6662	1	1950-11-05	2006-04-06	SZ	W	1	13	NA	0	⋯	164[illegible]
6686	1	1949-12-07	2006-04-19	IT	W	1	12	0	0	⋯	140[illegible]
6746	0	1943-12-13	2006-04-26	SZ	W	1	12	NA	0	⋯	199[illegible]
9029	0	1943-12-31	2003-10-18	PR	O	4	14	NA	0	⋯	156[illegible]
9056	0	1949-05-18	2003-12-03	TL	A	4	14	2	0	⋯	145[illegible]
9094	1	1948-12-04	2004-02-03	BV	O	4	18	0	0	⋯	388[illegible]
9179	0	1948-07-07	2004-07-15	CI	O	0	13	2	0	⋯	89[illegible]

pt	sex	datbirth	datexam	bthpl_dem	ethori_self	edtyp	edlv	mrtsts	sclhlp	⋯	veg_av
<int>	<int>	<date>	<date>	<chr>	<chr>	<int>	<int>	<int>	<int>	⋯	<db
9208	1	1940-07-25	2004-08-23	CI	O	3	15	0	0	⋯	427
9237	0	1938-05-02	2004-09-16	TY	O	1	12	0	0	⋯	89
9269	1	1944-03-22	2004-10-29	EY	B	4	21	0	0	⋯	367
9292	0	1939-07-17	2005-02-02	CO	O	0	12	0	1	⋯	274
9366	1	1946-08-20	2005-03-31	CI	O	4	25	0	0	⋯	299
9397	0	1949-11-11	2005-05-04	MY	A	3	17	0	0	⋯	401
9413	0	1947-01-13	2005-05-18	X	O	0	8	0	0	⋯	415
9429	1	1947-11-02	2005-06-06	X	O	3	18	0	0	⋯	127
9448	0	1941-04-05	2005-07-25	X	O	2	3	3	0	⋯	287
9490	1	1941-05-01	2005-09-13	TY	A	4	12	0	0	⋯	601
9494	0	1950-09-29	2005-09-12	PS	A	4	17	0	0	⋯	123
9520	0	1947-09-01	2005-11-01	TY	A	3	14	0	1	⋯	407
9549	0	1948-06-21	2005-12-06	HT	O	2	18	NA	0	⋯	220
9551	1	1940-10-23	2005-12-06	TY	A	4	17	0	1	⋯	744
9565	0	1941-07-09	2006-01-12	IR	A	4	17	0	1	⋯	243
9569	0	1944-06-18	2006-02-16	TU	B	0	9	2	1	⋯	51
9592	0	1943-[illegible]	2006-01-[illegible]	IR	A	1	12	2	1	⋯	272

In [175…

```
########################################
##########TOTAL DAIRY#################
########################################

weight.model = weightit(formula("total_dairy_avg ~ veg_avg+fru_avg+sea_avg+meat_avg
               grains_avg+alcohol_avg+sugary_avg+fats_avg+sex+
                 age_cat+edu+sm_b+HTA_b+depre_b+pa_b+cvevent_b+famincome_b+diab_b+c
                        method = "ps", use.kernel=T)

#check weights
summary(weight.model$weights)

#add weights to data
```

```
ch_sensi$ipwtotal <- weight.model$weights

#covariate balance for weights obtained with linear model for treatment dose
#and kernel density estimation
balance.table.2 = bal.tab(weight.model, r.threshold=0.1)$Balance
balance.table.2

########################################
##########FERMENTED DAIRY############
########################################
weight.model = weightit(formula("ferm_dairy_avg ~ nonferm_dairy_avg+veg_avg+fru_av
               grains_avg+alcohol_avg+sugary_avg+fats_avg+sex+
                 age_cat+edu+sm_b+HTA_b+depre_b+pa_b+cvevent_b+famincome_b+diab_b+
                        method = "ps", use.kernel=T)

#check weights
summary(weight.model$weights)

#add weights to data
ch_sensi$ipwferm <- weight.model$weights

#covariate balance for weights obtained with linear model for treatment dose
#and kernel density estimation
balance.table.2 = bal.tab(weight.model, r.threshold=0.1)$Balance
balance.table.2

########################################
##########NON FERMENTED DAIRY########
########################################
weight.model = weightit(formula("nonferm_dairy_avg ~ ferm_dairy_avg+veg_avg+fru_av
               grains_avg+alcohol_avg+sugary_avg+fats_avg+sex+
                 age_cat+edu+sm_b+HTA_b+depre_b+pa_b+cvevent_b+famincome_b+diab_b+
                        method = "ps", use.kernel=T)

#check weights
summary(weight.model$weights)

#add weights to data
ch_sensi$ipwnonferm <- weight.model$weights

#covariate balance for weights obtained with linear model for treatment dose
#and kernel density estimation
balance.table.2 = bal.tab(weight.model, r.threshold=0.1)$Balance
balance.table.2

########################################
##########FULL FAT DAIRY#############
########################################
weight.model = weightit(formula("fullfat_dairy_avg ~ nonfat_dairy_avg+veg_avg+fru_
               grains_avg+alcohol_avg+sugary_avg+fats_avg+sex+
                 age_cat+edu+sm_b+HTA_b+depre_b+pa_b+cvevent_b+famincome_b+diab_b+
                        method = "ps", use.kernel=T)

#check weights
summary(weight.model$weights)

#add weights to data
ch_sensi$ipwff <- weight.model$weights

#covariate balance for weights obtained with linear model for treatment dose
#and kernel density estimation
balance.table.2 = bal.tab(weight.model, r.threshold=0.1)$Balance
balance.table.2
```

```
########################################
###########NON FAT DAIRY#############
########################################

weight.model = weightit(formula("nonferm_dairy_avg ~ ferm_dairy_avg+veg_avg+fru_av
               grains_avg+alcohol_avg+sugary_avg+fats_avg+sex+
                 age_cat+edu+sm_b+HTA_b+depre_b+pa_b+cvevent_b+famincome_b+diab_b+
                        method = "ps", use.kernel=T)

#check weights
summary(weight.model$weights)

#add weights to data
ch_sensi$ipwnonfat <- weight.model$weights

#covariate balance for weights obtained with linear model for treatment dose
#and kernel density estimation
balance.table.2 = bal.tab(weight.model, r.threshold=0.1)$Balance
balance.table.2

########################################
###########SUGARY DAIRY#############
########################################
weight.model = weightit(formula("sugar_dairy_avg ~ nonsug_dairy_avg+veg_avg+fru_av
               grains_avg+alcohol_avg+sugary_avg+fats_avg+sex+
                 age_cat+edu+sm_b+HTA_b+depre_b+pa_b+cvevent_b+famincome_b+diab_b+
                        method = "ps", use.kernel=T)

#check weights
summary(weight.model$weights)

#add weights to data
ch_sensi$ipwsg <- weight.model$weights

#covariate balance for weights obtained with linear model for treatment dose
#and kernel density estimation
balance.table.2 = bal.tab(weight.model, r.threshold=0.1)$Balance
balance.table.2

threshold_99a <- quantile(ch_sensi$ipwtotal, 0.995)
ch_sensi$ipwtotal[ch_sensi$ipwtotal > threshold_99] <- threshold_99a
threshold_99b <- quantile(ch_sensi$ipwferm, 0.995)
ch_sensi$ipwferm[ch_sensi$ipwferm > threshold_99] <- threshold_99b
threshold_99c <- quantile(ch_sensi$ipwnonferm, 0.995)
ch_sensi$ipwnonferm[ch_sensi$ipwnonferm > threshold_99] <- threshold_99c
threshold_99d <- quantile(ch_sensi$ipwff, 0.995)
ch_sensi$ipwff[ch_sensi$ipwff > threshold_99] <- threshold_99d
threshold_99e <- quantile(ch_sensi$ipwnonfat, 0.995)
ch_sensi$ipwnonfat[ch_sensi$ipwnonfat > threshold_99] <- threshold_99e
threshold_99f <- quantile(ch_sensi$ipwff, 0.995)
ch_sensi$ipwff[ch_sensi$ipwff > threshold_99] <- threshold_99f
```

```
   Min. 1st Qu.  Median    Mean 3rd Qu.    Max.
    0.2     0.8     1.0     1.1     1.1    41.6
```

A data.frame: 50 × 4

	Type	Corr.Un	Corr.Adj	R.Threshold
	<chr>	<dbl>	<dbl>	<chr>
**veg_avg**	Contin.	NA	-0.114345	Not Balanced, >0.1
**fru_avg**	Contin.	NA	-0.089456	Balanced, <0.1
**fru_avg:<NA>**	Binary	NA	-0.010250	Balanced, <0.1
**sea_avg**	Contin.	NA	-0.063961	Balanced, <0.1
**sea_avg:<NA>**	Binary	NA	-0.019255	Balanced, <0.1
**meat_avg**	Contin.	NA	-0.059130	Balanced, <0.1
**meat_avg:<NA>**	Binary	NA	-0.005928	Balanced, <0.1
**eggs_avg**	Contin.	NA	-0.101318	Not Balanced, >0.1
**grains_avg**	Contin.	NA	-0.071979	Balanced, <0.1
**grains_avg:<NA>**	Binary	NA	-0.003112	Balanced, <0.1
**alcohol_avg**	Contin.	NA	-0.014981	Balanced, <0.1
**alcohol_avg:<NA>**	Binary	NA	0.020765	Balanced, <0.1
**sugary_avg**	Contin.	NA	-0.072813	Balanced, <0.1
**sugary_avg:<NA>**	Binary	NA	-0.008427	Balanced, <0.1
**fats_avg**	Contin.	NA	-0.039141	Balanced, <0.1
**fats_avg:<NA>**	Binary	NA	0.009141	Balanced, <0.1
**sex**	Binary	NA	-0.085799	Balanced, <0.1
**age_cat_1**	Binary	NA	0.081557	Balanced, <0.1
**age_cat_2**	Binary	NA	-0.113003	Not Balanced, >0.1
**age_cat_3**	Binary	NA	0.030104	Balanced, <0.1
**edu_Elementary**	Binary	NA	0.084481	Balanced, <0.1
**edu_High school**	Binary	NA	-0.065354	Balanced, <0.1
**edu_Superior**	Binary	NA	-0.040693	Balanced, <0.1
**sm_b_0**	Binary	NA	0.014902	Balanced, <0.1
**sm_b_1**	Binary	NA	-0.022973	Balanced, <0.1
**sm_b_2**	Binary	NA	0.011457	Balanced, <0.1
**sm_b:<NA>**	Binary	NA	-0.000993	Balanced, <0.1
**HTA_b**	Binary	NA	0.067941	Balanced, <0.1
**HTA_b:<NA>**	Binary	NA	-0.012611	Balanced, <0.1
**depre_b**	Binary	NA	-0.125991	Not Balanced, >0.1
**depre_b:<NA>**	Binary	NA	0.019249	Balanced, <0.1
**pa_b_High**	Binary	NA	-0.030308	Balanced, <0.1
**pa_b_Low**	Binary	NA	0.035395	Balanced, <0.1
**pa_b_Medium**	Binary	NA	-0.011954	Balanced, <0.1

	Type	Corr.Un	Corr.Adj	R.Threshold
	<chr>	<dbl>	<dbl>	<chr>
**pa_b:<NA>**	Binary	NA	-0.171153	Not Balanced, >0.1
**cvevent_b**	Binary	NA	-0.009114	Balanced, <0.1
**famincome_b_1**	Binary	NA	-0.056142	Balanced, <0.1
**famincome_b_2**	Binary	NA	0.030694	Balanced, <0.1
**famincome_b_3**	Binary	NA	0.032169	Balanced, <0.1
**famincome_b:<NA>**	Binary	NA	0.002788	Balanced, <0.1
**diab_b**	Binary	NA	0.020157	Balanced, <0.1
**occ_b_1**	Binary	NA	0.036167	Balanced, <0.1
**occ_b_2**	Binary	NA	-0.004794	Balanced, <0.1
**occ_b_3**	Binary	NA	0.026704	Balanced, <0.1
**occ_b_9**	Binary	NA	-0.034382	Balanced, <0.1
**occ_b:<NA>**	Binary	NA	0.002884	Balanced, <0.1
**bmi_cat_1**	Binary	NA	0.031349	Balanced, <0.1
**bmi_cat_2**	Binary	NA	-0.033022	Balanced, <0.1
**bmi_cat_3**	Binary	NA	0.002211	Balanced, <0.1
**bmi_cat:<NA>**	Binary	NA	-0.010967	Balanced, <0.1

```
   Min. 1st Qu.  Median    Mean 3rd Qu.    Max. 
   0.21    0.86    0.95    1.13    1.11   13.16 
```

A data.frame: 51 × 4

	Type	Corr.Un	Corr.Adj	R.Threshold
	<chr>	<dbl>	<dbl>	<chr>
**nonferm_dairy_avg**	Contin.	NA	-0.02964	Balanced, <0.1
**veg_avg**	Contin.	NA	0.00579	Balanced, <0.1
**fru_avg**	Contin.	NA	0.00100	Balanced, <0.1
**fru_avg:<NA>**	Binary	NA	0.00138	Balanced, <0.1
**sea_avg**	Contin.	NA	-0.02672	Balanced, <0.1
**sea_avg:<NA>**	Binary	NA	-0.01045	Balanced, <0.1
**meat_avg**	Contin.	NA	0.03649	Balanced, <0.1
**meat_avg:<NA>**	Binary	NA	-0.00534	Balanced, <0.1
**eggs_avg**	Contin.	NA	-0.03118	Balanced, <0.1
**grains_avg**	Contin.	NA	0.01406	Balanced, <0.1
**grains_avg:<NA>**	Binary	NA	-0.00890	Balanced, <0.1
**alcohol_avg**	Contin.	NA	0.00304	Balanced, <0.1
**alcohol_avg:<NA>**	Binary	NA	0.04598	Balanced, <0.1
**sugary_avg**	Contin.	NA	0.00903	Balanced, <0.1
**sugary_avg:<NA>**	Binary	NA	-0.06175	Balanced, <0.1
**fats_avg**	Contin.	NA	-0.00346	Balanced, <0.1
**fats_avg:<NA>**	Binary	NA	0.01381	Balanced, <0.1
**sex**	Binary	NA	-0.01121	Balanced, <0.1
**age_cat_1**	Binary	NA	0.01363	Balanced, <0.1
**age_cat_2**	Binary	NA	-0.01893	Balanced, <0.1
**age_cat_3**	Binary	NA	0.00511	Balanced, <0.1
**edu_Elementary**	Binary	NA	-0.00373	Balanced, <0.1
**edu_High school**	Binary	NA	-0.01024	Balanced, <0.1
**edu_Superior**	Binary	NA	0.01520	Balanced, <0.1
**sm_b_0**	Binary	NA	0.00600	Balanced, <0.1
**sm_b_1**	Binary	NA	0.00606	Balanced, <0.1
**sm_b_2**	Binary	NA	-0.01751	Balanced, <0.1
**sm_b:<NA>**	Binary	NA	0.00693	Balanced, <0.1
**HTA_b**	Binary	NA	0.00995	Balanced, <0.1
**HTA_b:<NA>**	Binary	NA	-0.00531	Balanced, <0.1
**depre_b**	Binary	NA	-0.02908	Balanced, <0.1
**depre_b:<NA>**	Binary	NA	0.02097	Balanced, <0.1
**pa_b_High**	Binary	NA	-0.01554	Balanced, <0.1
**pa_b_Low**	Binary	NA	0.02105	Balanced, <0.1

	Type	Corr.Un	Corr.Adj	R.Threshold
	<chr>	<dbl>	<dbl>	<chr>
**pa_b_Medium**	Binary	NA	-0.00914	Balanced, <0.1
**pa_b:<NA>**	Binary	NA	-0.02788	Balanced, <0.1
**cvevent_b**	Binary	NA	-0.00520	Balanced, <0.1
**famincome_b_1**	Binary	NA	0.00488	Balanced, <0.1
**famincome_b_2**	Binary	NA	-0.02047	Balanced, <0.1
**famincome_b_3**	Binary	NA	0.02207	Balanced, <0.1
**famincome_b:<NA>**	Binary	NA	0.01055	Balanced, <0.1
**diab_b**	Binary	NA	0.00756	Balanced, <0.1
**occ_b_1**	Binary	NA	0.02645	Balanced, <0.1
**occ_b_2**	Binary	NA	0.01064	Balanced, <0.1
**occ_b_3**	Binary	NA	0.02629	Balanced, <0.1
**occ_b_9**	Binary	NA	-0.03996	Balanced, <0.1
**occ_b:<NA>**	Binary	NA	0.01347	Balanced, <0.1
**bmi_cat_1**	Binary	NA	0.01213	Balanced, <0.1
**bmi_cat_2**	Binary	NA	-0.01705	Balanced, <0.1
**bmi_cat_3**	Binary	NA	0.00650	Balanced, <0.1
**bmi_cat:<NA>**	Binary	NA	-0.00141	Balanced, <0.1

```
 Min. 1st Qu.  Median    Mean 3rd Qu.    Max. 
  0.3     0.9     1.1     1.5     1.3   154.8 
```

A data.frame: 51 × 4

	Type	Corr.Un	Corr.Adj	R.Threshold
	<chr>	<dbl>	<dbl>	<chr>
**ferm_dairy_avg**	Contin.	NA	-0.051529	Balanced, <0.1
**veg_avg**	Contin.	NA	0.022681	Balanced, <0.1
**fru_avg**	Contin.	NA	-0.024633	Balanced, <0.1
**fru_avg:<NA>**	Binary	NA	-0.020482	Balanced, <0.1
**sea_avg**	Contin.	NA	0.074181	Balanced, <0.1
**sea_avg:<NA>**	Binary	NA	-0.026524	Balanced, <0.1
**meat_avg**	Contin.	NA	0.000508	Balanced, <0.1
**meat_avg:<NA>**	Binary	NA	-0.023339	Balanced, <0.1
**eggs_avg**	Contin.	NA	0.033004	Balanced, <0.1
**grains_avg**	Contin.	NA	-0.180440	Not Balanced, >0.1
**grains_avg:<NA>**	Binary	NA	-0.008352	Balanced, <0.1
**alcohol_avg**	Contin.	NA	0.046890	Balanced, <0.1
**alcohol_avg:<NA>**	Binary	NA	0.004746	Balanced, <0.1
**sugary_avg**	Contin.	NA	-0.192751	Not Balanced, >0.1
**sugary_avg:<NA>**	Binary	NA	0.003160	Balanced, <0.1
**fats_avg**	Contin.	NA	-0.029405	Balanced, <0.1
**fats_avg:<NA>**	Binary	NA	-0.002179	Balanced, <0.1
**sex**	Binary	NA	0.055757	Balanced, <0.1
**age_cat_1**	Binary	NA	0.152630	Not Balanced, >0.1
**age_cat_2**	Binary	NA	0.020482	Balanced, <0.1
**age_cat_3**	Binary	NA	-0.274248	Not Balanced, >0.1
**edu_Elementary**	Binary	NA	-0.050998	Balanced, <0.1
**edu_High school**	Binary	NA	0.027792	Balanced, <0.1
**edu_Superior**	Binary	NA	0.036470	Balanced, <0.1
**sm_b_0**	Binary	NA	0.036305	Balanced, <0.1
**sm_b_1**	Binary	NA	0.052912	Balanced, <0.1
**sm_b_2**	Binary	NA	-0.129465	Not Balanced, >0.1
**sm_b:<NA>**	Binary	NA	-0.010153	Balanced, <0.1
**HTA_b**	Binary	NA	-0.022718	Balanced, <0.1
**HTA_b:<NA>**	Binary	NA	-1.271925	Not Balanced, >0.1
**depre_b**	Binary	NA	0.027246	Balanced, <0.1
**depre_b:<NA>**	Binary	NA	0.003140	Balanced, <0.1
**pa_b_High**	Binary	NA	0.018139	Balanced, <0.1
**pa_b_Low**	Binary	NA	0.032769	Balanced, <0.1

	Type	Corr.Un	Corr.Adj	R.Threshold
	<chr>	<dbl>	<dbl>	<chr>
**pa_b_Medium**	Binary	NA	-0.048959	Balanced, <0.1
**pa_b:<NA>**	Binary	NA	-0.169914	Not Balanced, >0.1
**cvevent_b**	Binary	NA	0.020602	Balanced, <0.1
**famincome_b_1**	Binary	NA	-0.034058	Balanced, <0.1
**famincome_b_2**	Binary	NA	0.032006	Balanced, <0.1
**famincome_b_3**	Binary	NA	0.000813	Balanced, <0.1
**famincome_b:<NA>**	Binary	NA	-0.127138	Not Balanced, >0.1
**diab_b**	Binary	NA	0.046163	Balanced, <0.1
**occ_b_1**	Binary	NA	0.027086	Balanced, <0.1
**occ_b_2**	Binary	NA	0.010695	Balanced, <0.1
**occ_b_3**	Binary	NA	0.032888	Balanced, <0.1
**occ_b_9**	Binary	NA	-0.045263	Balanced, <0.1
**occ_b:<NA>**	Binary	NA	0.001905	Balanced, <0.1
**bmi_cat_1**	Binary	NA	0.049684	Balanced, <0.1
**bmi_cat_2**	Binary	NA	-0.079620	Balanced, <0.1
**bmi_cat_3**	Binary	NA	0.039563	Balanced, <0.1
**bmi_cat:<NA>**	Binary	NA	-0.008768	Balanced, <0.1

```
   Min. 1st Qu.  Median    Mean 3rd Qu.    Max. 
   0.24    0.82    0.96    1.13    1.10   27.43 
```

A data.frame: 51 × 4

	Type	Corr.Un	Corr.Adj	R.Threshold
	<chr>	<dbl>	<dbl>	<chr>
**nonfat_dairy_avg**	Contin.	NA	-0.177418	Not Balanced, >0.1
**veg_avg**	Contin.	NA	0.003373	Balanced, <0.1
**fru_avg**	Contin.	NA	-0.063273	Balanced, <0.1
**fru_avg:<NA>**	Binary	NA	-0.005825	Balanced, <0.1
**sea_avg**	Contin.	NA	-0.045638	Balanced, <0.1
**sea_avg:<NA>**	Binary	NA	-0.017785	Balanced, <0.1
**meat_avg**	Contin.	NA	0.003280	Balanced, <0.1
**meat_avg:<NA>**	Binary	NA	-0.005977	Balanced, <0.1
**eggs_avg**	Contin.	NA	-0.017303	Balanced, <0.1
**grains_avg**	Contin.	NA	-0.025210	Balanced, <0.1
**grains_avg:<NA>**	Binary	NA	-0.009140	Balanced, <0.1
**alcohol_avg**	Contin.	NA	-0.024120	Balanced, <0.1
**alcohol_avg:<NA>**	Binary	NA	0.018684	Balanced, <0.1
**sugary_avg**	Contin.	NA	-0.045858	Balanced, <0.1
**sugary_avg:<NA>**	Binary	NA	0.005984	Balanced, <0.1
**fats_avg**	Contin.	NA	0.001460	Balanced, <0.1
**fats_avg:<NA>**	Binary	NA	0.001538	Balanced, <0.1
**sex**	Binary	NA	-0.063519	Balanced, <0.1
**age_cat_1**	Binary	NA	-0.019433	Balanced, <0.1
**age_cat_2**	Binary	NA	0.001145	Balanced, <0.1
**age_cat_3**	Binary	NA	0.029570	Balanced, <0.1
**edu_Elementary**	Binary	NA	0.073876	Balanced, <0.1
**edu_High school**	Binary	NA	-0.079982	Balanced, <0.1
**edu_Superior**	Binary	NA	-0.012272	Balanced, <0.1
**sm_b_0**	Binary	NA	-0.035583	Balanced, <0.1
**sm_b_1**	Binary	NA	0.032926	Balanced, <0.1
**sm_b_2**	Binary	NA	0.004340	Balanced, <0.1
**sm_b:<NA>**	Binary	NA	0.003890	Balanced, <0.1
**HTA_b**	Binary	NA	-0.047751	Balanced, <0.1
**HTA_b:<NA>**	Binary	NA	0.008330	Balanced, <0.1
**depre_b**	Binary	NA	-0.009909	Balanced, <0.1
**depre_b:<NA>**	Binary	NA	0.016737	Balanced, <0.1
**pa_b_High**	Binary	NA	-0.087412	Balanced, <0.1
**pa_b_Low**	Binary	NA	0.058653	Balanced, <0.1

	Type	Corr.Un	Corr.Adj	R.Threshold
	<chr>	<dbl>	<dbl>	<chr>
**pa_b_Medium**	Binary	NA	0.010693	Balanced, <0.1
**pa_b:<NA>**	Binary	NA	-0.030292	Balanced, <0.1
**cvevent_b**	Binary	NA	-0.051393	Balanced, <0.1
**famincome_b_1**	Binary	NA	0.008619	Balanced, <0.1
**famincome_b_2**	Binary	NA	-0.005968	Balanced, <0.1
**famincome_b_3**	Binary	NA	-0.003185	Balanced, <0.1
**famincome_b:<NA>**	Binary	NA	0.001293	Balanced, <0.1
**diab_b**	Binary	NA	0.015866	Balanced, <0.1
**occ_b_1**	Binary	NA	-0.026072	Balanced, <0.1
**occ_b_2**	Binary	NA	-0.014833	Balanced, <0.1
**occ_b_3**	Binary	NA	0.040234	Balanced, <0.1
**occ_b_9**	Binary	NA	-0.007287	Balanced, <0.1
**occ_b:<NA>**	Binary	NA	-0.000818	Balanced, <0.1
**bmi_cat_1**	Binary	NA	-0.000922	Balanced, <0.1
**bmi_cat_2**	Binary	NA	0.001497	Balanced, <0.1
**bmi_cat_3**	Binary	NA	-0.000759	Balanced, <0.1
**bmi_cat:<NA>**	Binary	NA	-0.003045	Balanced, <0.1

```
   Min. 1st Qu.  Median    Mean 3rd Qu.    Max. 
    0.3     0.9     1.1     1.5     1.3   154.8 
```

A data.frame: 51 × 4

	Type	Corr.Un	Corr.Adj	R.Threshold
	<chr>	<dbl>	<dbl>	<chr>
**ferm_dairy_avg**	Contin.	NA	-0.051529	Balanced, <0.1
**veg_avg**	Contin.	NA	0.022681	Balanced, <0.1
**fru_avg**	Contin.	NA	-0.024633	Balanced, <0.1
**fru_avg:<NA>**	Binary	NA	-0.020482	Balanced, <0.1
**sea_avg**	Contin.	NA	0.074181	Balanced, <0.1
**sea_avg:<NA>**	Binary	NA	-0.026524	Balanced, <0.1
**meat_avg**	Contin.	NA	0.000508	Balanced, <0.1
**meat_avg:<NA>**	Binary	NA	-0.023339	Balanced, <0.1
**eggs_avg**	Contin.	NA	0.033004	Balanced, <0.1
**grains_avg**	Contin.	NA	-0.180440	Not Balanced, >0.1
**grains_avg:<NA>**	Binary	NA	-0.008352	Balanced, <0.1
**alcohol_avg**	Contin.	NA	0.046890	Balanced, <0.1
**alcohol_avg:<NA>**	Binary	NA	0.004746	Balanced, <0.1
**sugary_avg**	Contin.	NA	-0.192751	Not Balanced, >0.1
**sugary_avg:<NA>**	Binary	NA	0.003160	Balanced, <0.1
**fats_avg**	Contin.	NA	-0.029405	Balanced, <0.1
**fats_avg:<NA>**	Binary	NA	-0.002179	Balanced, <0.1
**sex**	Binary	NA	0.055757	Balanced, <0.1
**age_cat_1**	Binary	NA	0.152630	Not Balanced, >0.1
**age_cat_2**	Binary	NA	0.020482	Balanced, <0.1
**age_cat_3**	Binary	NA	-0.274248	Not Balanced, >0.1
**edu_Elementary**	Binary	NA	-0.050998	Balanced, <0.1
**edu_High school**	Binary	NA	0.027792	Balanced, <0.1
**edu_Superior**	Binary	NA	0.036470	Balanced, <0.1
**sm_b_0**	Binary	NA	0.036305	Balanced, <0.1
**sm_b_1**	Binary	NA	0.052912	Balanced, <0.1
**sm_b_2**	Binary	NA	-0.129465	Not Balanced, >0.1
**sm_b:<NA>**	Binary	NA	-0.010153	Balanced, <0.1
**HTA_b**	Binary	NA	-0.022718	Balanced, <0.1
**HTA_b:<NA>**	Binary	NA	-1.271925	Not Balanced, >0.1
**depre_b**	Binary	NA	0.027246	Balanced, <0.1
**depre_b:<NA>**	Binary	NA	0.003140	Balanced, <0.1
**pa_b_High**	Binary	NA	0.018139	Balanced, <0.1
**pa_b_Low**	Binary	NA	0.032769	Balanced, <0.1

	Type	Corr.Un	Corr.Adj	R.Threshold
	<chr>	<dbl>	<dbl>	<chr>
**pa_b_Medium**	Binary	NA	-0.048959	Balanced, <0.1
**pa_b:<NA>**	Binary	NA	-0.169914	Not Balanced, >0.1
**cvevent_b**	Binary	NA	0.020602	Balanced, <0.1
**famincome_b_1**	Binary	NA	-0.034058	Balanced, <0.1
**famincome_b_2**	Binary	NA	0.032006	Balanced, <0.1
**famincome_b_3**	Binary	NA	0.000813	Balanced, <0.1
**famincome_b:<NA>**	Binary	NA	-0.127138	Not Balanced, >0.1
**diab_b**	Binary	NA	0.046163	Balanced, <0.1
**occ_b_1**	Binary	NA	0.027086	Balanced, <0.1
**occ_b_2**	Binary	NA	0.010695	Balanced, <0.1
**occ_b_3**	Binary	NA	0.032888	Balanced, <0.1
**occ_b_9**	Binary	NA	-0.045263	Balanced, <0.1
**occ_b:<NA>**	Binary	NA	0.001905	Balanced, <0.1
**bmi_cat_1**	Binary	NA	0.049684	Balanced, <0.1
**bmi_cat_2**	Binary	NA	-0.079620	Balanced, <0.1
**bmi_cat_3**	Binary	NA	0.039563	Balanced, <0.1
**bmi_cat:<NA>**	Binary	NA	-0.008768	Balanced, <0.1

```
Min. 1st Qu.  Median    Mean 3rd Qu.    Max.
 0.2     0.9     1.1     1.5     1.3    92.4
```

A data.frame: 51 × 4

	Type	Corr.Un	Corr.Adj	R.Threshold
	<chr>	<dbl>	<dbl>	<chr>
**nonsug_dairy_avg**	Contin.	NA	-0.195177	Not Balanced, >0.1
**veg_avg**	Contin.	NA	0.023399	Balanced, <0.1
**fru_avg**	Contin.	NA	-0.065696	Balanced, <0.1
**fru_avg:<NA>**	Binary	NA	0.007919	Balanced, <0.1
**sea_avg**	Contin.	NA	-0.080074	Balanced, <0.1
**sea_avg:<NA>**	Binary	NA	-0.007460	Balanced, <0.1
**meat_avg**	Contin.	NA	0.006590	Balanced, <0.1
**meat_avg:<NA>**	Binary	NA	-0.007803	Balanced, <0.1
**eggs_avg**	Contin.	NA	0.050695	Balanced, <0.1
**grains_avg**	Contin.	NA	-0.150455	Not Balanced, >0.1
**grains_avg:<NA>**	Binary	NA	-0.032716	Balanced, <0.1
**alcohol_avg**	Contin.	NA	-0.003791	Balanced, <0.1
**alcohol_avg:<NA>**	Binary	NA	0.004409	Balanced, <0.1
**sugary_avg**	Contin.	NA	-0.171336	Not Balanced, >0.1
**sugary_avg:<NA>**	Binary	NA	-0.031270	Balanced, <0.1
**fats_avg**	Contin.	NA	0.012364	Balanced, <0.1
**fats_avg:<NA>**	Binary	NA	0.008782	Balanced, <0.1
**sex**	Binary	NA	-0.076327	Balanced, <0.1
**age_cat_1**	Binary	NA	-0.059793	Balanced, <0.1
**age_cat_2**	Binary	NA	0.045617	Balanced, <0.1
**age_cat_3**	Binary	NA	0.030990	Balanced, <0.1
**edu_Elementary**	Binary	NA	0.039409	Balanced, <0.1
**edu_High school**	Binary	NA	0.063267	Balanced, <0.1
**edu_Superior**	Binary	NA	-0.114711	Not Balanced, >0.1
**sm_b_0**	Binary	NA	-0.016167	Balanced, <0.1
**sm_b_1**	Binary	NA	0.019956	Balanced, <0.1
**sm_b_2**	Binary	NA	-0.005250	Balanced, <0.1
**sm_b:<NA>**	Binary	NA	0.008622	Balanced, <0.1
**HTA_b**	Binary	NA	-0.014818	Balanced, <0.1
**HTA_b:<NA>**	Binary	NA	-0.082825	Balanced, <0.1
**depre_b**	Binary	NA	0.027754	Balanced, <0.1
**depre_b:<NA>**	Binary	NA	-0.004550	Balanced, <0.1
**pa_b_High**	Binary	NA	-0.056935	Balanced, <0.1
**pa_b_Low**	Binary	NA	0.073280	Balanced, <0.1

	Type	Corr.Un	Corr.Adj	R.Threshold
	<chr>	<dbl>	<dbl>	<chr>
**pa_b_Medium**	Binary	NA	-0.029518	Balanced, <0.1
**pa_b:<NA>**	Binary	NA	-0.000457	Balanced, <0.1
**cvevent_b**	Binary	NA	0.032163	Balanced, <0.1
**famincome_b_1**	Binary	NA	0.054005	Balanced, <0.1
**famincome_b_2**	Binary	NA	0.017282	Balanced, <0.1
**famincome_b_3**	Binary	NA	-0.096339	Balanced, <0.1
**famincome_b:<NA>**	Binary	NA	0.010348	Balanced, <0.1
**diab_b**	Binary	NA	0.019303	Balanced, <0.1
**occ_b_1**	Binary	NA	-0.153873	Not Balanced, >0.1
**occ_b_2**	Binary	NA	0.025610	Balanced, <0.1
**occ_b_3**	Binary	NA	-0.005812	Balanced, <0.1
**occ_b_9**	Binary	NA	0.061744	Balanced, <0.1
**occ_b:<NA>**	Binary	NA	0.014911	Balanced, <0.1
**bmi_cat_1**	Binary	NA	0.028572	Balanced, <0.1
**bmi_cat_2**	Binary	NA	-0.050067	Balanced, <0.1
**bmi_cat_3**	Binary	NA	0.028406	Balanced, <0.1
**bmi_cat:<NA>**	Binary	NA	0.015077	Balanced, <0.1

Computing ATEs (TOTAL EFFECTS)

CDR

In [176…

```
########################################
#########TOTAL DAIRY################
########################################

msm.w <- geeglm(F3CDR ~ total_dairy_avg, data=ch_sensi, weights=ipwtotal, id=pt,
                corstr="independence")
summary(msm.w)

beta <- coef(msm.w)
SE <- coef(summary(msm.w))[,2]
lcl <- beta-qnorm(0.975)*SE
ucl <- beta+qnorm(0.975)*SE
scda <-cbind(beta*100, lcl*100, ucl*100)

########################################
#########FERMENTED DAIRY############
########################################

msm.w <- geeglm(F3CDR ~ ferm_dairy_avg, data=ch_sensi, weights=ipwferm, id=pt,
                corstr="independence")
summary(msm.w)

beta <- coef(msm.w)
```

```
SE <- coef(summary(msm.w))[,2]
lcl <- beta-qnorm(0.975)*SE
ucl <- beta+qnorm(0.975)*SE
scdb <-cbind(beta*100, lcl*100, ucl*100)

#####################################
##########NON FERMENTED DAIRY########
#####################################

msm.w <- geeglm(F3CDR ~ nonferm_dairy_avg, data=ch_sensi, weights=ipwnonferm, id=pt
                corstr="independence")
summary(msm.w)

beta <- coef(msm.w)
SE <- coef(summary(msm.w))[,2]
lcl <- beta-qnorm(0.975)*SE
ucl <- beta+qnorm(0.975)*SE
scdc <-cbind(beta*100, lcl*100, ucl*100)

#####################################
##########FULL FAT DAIRY#############
#####################################

msm.w <- geeglm(F3CDR ~ fullfat_dairy_avg, data=ch_sensi, weights=ipwff, id=pt,
                corstr="independence")
summary(msm.w)

beta <- coef(msm.w)
SE <- coef(summary(msm.w))[,2]
lcl <- beta-qnorm(0.975)*SE
ucl <- beta+qnorm(0.975)*SE
scdd <-cbind(beta*100, lcl*100, ucl*100)
#####################################
###########NON FAT DAIRY#############
#####################################

msm.w <- geeglm(F3CDR ~ nonferm_dairy_avg, data=ch_sensi, weights=ipwnonfat, id=pt
                corstr="independence")
summary(msm.w)

beta <- coef(msm.w)
SE <- coef(summary(msm.w))[,2]
lcl <- beta-qnorm(0.975)*SE
ucl <- beta+qnorm(0.975)*SE
scde <-cbind(beta*100, lcl*100, ucl*100)
#####################################
############SUGARY DAIRY#############
#####################################

msm.w <- geeglm(F3CDR ~ sugar_dairy_avg, data=ch_sensi, weights=ipwsg, id=pt,
                corstr="independence")
summary(msm.w)

beta <- coef(msm.w)
SE <- coef(summary(msm.w))[,2]
lcl <- beta-qnorm(0.975)*SE
ucl <- beta+qnorm(0.975)*SE
scdf <-cbind(beta*100, lcl*100, ucl*100)
```

```
Call:
geeglm(formula = F3CDR ~ total_dairy_avg, data = ch_sensi, weights = ipwtotal,
    id = pt, corstr = "independence")

 Coefficients:
                Estimate  Std.err  Wald Pr(>|W|)
(Intercept)     1.66e-01 2.84e-02 34.12  5.2e-09 ***
total_dairy_avg 1.41e-04 8.96e-05  2.49     0.11
---
Signif. codes:  0 '***' 0.001 '**' 0.01 '*' 0.05 '.' 0.1 ' ' 1

Correlation structure = independence
Estimated Scale Parameters:

            Estimate Std.err
(Intercept)   0.0595 0.00172
Number of clusters:   372  Maximum cluster size: 1
Call:
geeglm(formula = F3CDR ~ ferm_dairy_avg, data = ch_sensi, weights = ipwferm,
    id = pt, corstr = "independence")

 Coefficients:
               Estimate  Std.err Wald Pr(>|W|)
(Intercept)    0.187358 0.027541 46.3    1e-11 ***
ferm_dairy_avg 0.000128 0.000128  1.0     0.32
---
Signif. codes:  0 '***' 0.001 '**' 0.01 '*' 0.05 '.' 0.1 ' ' 1

Correlation structure = independence
Estimated Scale Parameters:

            Estimate Std.err
(Intercept)   0.0604 0.00152
Number of clusters:   372  Maximum cluster size: 1
Call:
geeglm(formula = F3CDR ~ nonferm_dairy_avg, data = ch_sensi,
    weights = ipwnonferm, id = pt, corstr = "independence")

 Coefficients:
                  Estimate  Std.err  Wald Pr(>|W|)
(Intercept)       1.91e-01 2.25e-02 71.79   <2e-16 ***
nonferm_dairy_avg 6.44e-05 1.46e-04  0.19     0.66
---
Signif. codes:  0 '***' 0.001 '**' 0.01 '*' 0.05 '.' 0.1 ' ' 1

Correlation structure = independence
Estimated Scale Parameters:

            Estimate Std.err
(Intercept)   0.0595 0.00182
Number of clusters:   372  Maximum cluster size: 1
```

```
Call:
geeglm(formula = F3CDR ~ fullfat_dairy_avg, data = ch_sensi,
    weights = ipwff, id = pt, corstr = "independence")

 Coefficients:
                  Estimate  Std.err  Wald Pr(>|W|)
(Intercept)       1.95e-01 3.18e-02 37.39  9.7e-10 ***
fullfat_dairy_avg 5.27e-05 1.12e-04  0.22     0.64
---
Signif. codes:  0 '***' 0.001 '**' 0.01 '*' 0.05 '.' 0.1 ' ' 1

Correlation structure = independence
Estimated Scale Parameters:

            Estimate Std.err
(Intercept)   0.0604 0.00157
Number of clusters:   372  Maximum cluster size: 1
Call:
geeglm(formula = F3CDR ~ nonferm_dairy_avg, data = ch_sensi,
    weights = ipwnonfat, id = pt, corstr = "independence")

 Coefficients:
                  Estimate  Std.err  Wald Pr(>|W|)
(Intercept)       1.91e-01 2.25e-02 71.79   <2e-16 ***
nonferm_dairy_avg 6.44e-05 1.46e-04  0.19     0.66
---
Signif. codes:  0 '***' 0.001 '**' 0.01 '*' 0.05 '.' 0.1 ' ' 1

Correlation structure = independence
Estimated Scale Parameters:

            Estimate Std.err
(Intercept)   0.0595 0.00182
Number of clusters:   372  Maximum cluster size: 1
Call:
geeglm(formula = F3CDR ~ sugar_dairy_avg, data = ch_sensi, weights = ipwsg,
    id = pt, corstr = "independence")

 Coefficients:
                 Estimate   Std.err  Wald Pr(>|W|)
(Intercept)      0.283907  0.053752 27.90  1.3e-07 ***
sugar_dairy_avg -0.000427  0.000393  1.18     0.28
---
Signif. codes:  0 '***' 0.001 '**' 0.01 '*' 0.05 '.' 0.1 ' ' 1

Correlation structure = independence
Estimated Scale Parameters:

            Estimate Std.err
(Intercept)   0.0615 0.00243
Number of clusters:   372  Maximum cluster size: 1
```

SCD

In [177…

```
#####################################
#########TOTAL DAIRY################
#####################################

msm.w <- geeglm(F3SCD_yn ~ total_dairy_avg, data=ch_sensi, weights=ipwtotal, id=pt
                corstr="independence")
summary(msm.w)

beta <- coef(msm.w)
```

```
SE <- coef(summary(msm.w))[,2]
lcl <- beta-qnorm(0.975)*SE
ucl <- beta+qnorm(0.975)*SE
scda <-cbind(beta*100, lcl*100, ucl*100)

#####################################
##########FERMENTED DAIRY############
#####################################

msm.w <- geeglm(F3SCD_yn ~ ferm_dairy_avg, data=ch_sensi, weights=ipwferm, id=pt,
                corstr="independence")
summary(msm.w)

beta <- coef(msm.w)
SE <- coef(summary(msm.w))[,2]
lcl <- beta-qnorm(0.975)*SE
ucl <- beta+qnorm(0.975)*SE
scdb <-cbind(beta*100, lcl*100, ucl*100)

#####################################
##########NON FERMENTED DAIRY########
#####################################

msm.w <- geeglm(F3SCD_yn ~ nonferm_dairy_avg, data=ch_sensi, weights=ipwnonferm, i
                corstr="independence")
summary(msm.w)

beta <- coef(msm.w)
SE <- coef(summary(msm.w))[,2]
lcl <- beta-qnorm(0.975)*SE
ucl <- beta+qnorm(0.975)*SE
scdc <-cbind(beta*100, lcl*100, ucl*100)

#####################################
##########FULL FAT DAIRY#############
#####################################

msm.w <- geeglm(F3SCD_yn ~ fullfat_dairy_avg, data=ch_sensi, weights=ipwff, id=pt,
                corstr="independence")
summary(msm.w)

beta <- coef(msm.w)
SE <- coef(summary(msm.w))[,2]
lcl <- beta-qnorm(0.975)*SE
ucl <- beta+qnorm(0.975)*SE
scdd <-cbind(beta*100, lcl*100, ucl*100)
#####################################
###########NON FAT DAIRY#############
#####################################

msm.w <- geeglm(F3SCD_yn ~ nonferm_dairy_avg, data=ch_sensi, weights=ipwnonfat, id
                corstr="independence")
summary(msm.w)

beta <- coef(msm.w)
SE <- coef(summary(msm.w))[,2]
lcl <- beta-qnorm(0.975)*SE
ucl <- beta+qnorm(0.975)*SE
scde <-cbind(beta*100, lcl*100, ucl*100)
#####################################
###########SUGARY DAIRY#############
#####################################

msm.w <- geeglm(F3SCD_yn ~ sugar_dairy_avg, data=ch_sensi, weights=ipwsg, id=pt,
```

```
                corstr="independence")
summary(msm.w)

beta <- coef(msm.w)
SE <- coef(summary(msm.w))[,2]
lcl <- beta-qnorm(0.975)*SE
ucl <- beta+qnorm(0.975)*SE
scdf <-cbind(beta*100, lcl*100, ucl*100)
```

```
Call:
geeglm(formula = F3SCD_yn ~ total_dairy_avg, data = ch_sensi,
    weights = ipwtotal, id = pt, corstr = "independence")

 Coefficients:
                Estimate  Std.err  Wald Pr(>|W|)
(Intercept)     0.126141 0.030561 17.04  3.7e-05 ***
total_dairy_avg 0.000127 0.000107  1.42     0.23
---
Signif. codes:  0 '***' 0.001 '**' 0.01 '*' 0.05 '.' 0.1 ' ' 1

Correlation structure = independence
Estimated Scale Parameters:

            Estimate Std.err
(Intercept)    0.132  0.0135
Number of clusters:   379  Maximum cluster size: 1
Call:
geeglm(formula = F3SCD_yn ~ ferm_dairy_avg, data = ch_sensi,
    weights = ipwferm, id = pt, corstr = "independence")

 Coefficients:
               Estimate  Std.err  Wald Pr(>|W|)
(Intercept)    0.131575 0.033327 15.59  7.9e-05 ***
ferm_dairy_avg 0.000181 0.000170  1.14     0.29
---
Signif. codes:  0 '***' 0.001 '**' 0.01 '*' 0.05 '.' 0.1 ' ' 1

Correlation structure = independence
Estimated Scale Parameters:

            Estimate Std.err
(Intercept)    0.133  0.0143
Number of clusters:   379  Maximum cluster size: 1
Call:
geeglm(formula = F3SCD_yn ~ nonferm_dairy_avg, data = ch_sensi,
    weights = ipwnonferm, id = pt, corstr = "independence")

 Coefficients:
                  Estimate  Std.err  Wald Pr(>|W|)
(Intercept)       1.61e-01 3.41e-02 22.25  2.4e-06 ***
nonferm_dairy_avg 3.84e-05 2.15e-04  0.03     0.86
---
Signif. codes:  0 '***' 0.001 '**' 0.01 '*' 0.05 '.' 0.1 ' ' 1

Correlation structure = independence
Estimated Scale Parameters:

            Estimate Std.err
(Intercept)    0.137  0.0172
Number of clusters:   379  Maximum cluster size: 1
```

```
Call:
geeglm(formula = F3SCD_yn ~ fullfat_dairy_avg, data = ch_sensi,
    weights = ipwff, id = pt, corstr = "independence")

 Coefficients:
                  Estimate  Std.err  Wald Pr(>|W|)    
(Intercept)       0.113439 0.030334 13.99  0.00018 ***
fullfat_dairy_avg 0.000196 0.000128  2.36  0.12435    
---
Signif. codes:  0 '***' 0.001 '**' 0.01 '*' 0.05 '.' 0.1 ' ' 1

Correlation structure = independence 
Estimated Scale Parameters:

            Estimate Std.err
(Intercept)    0.128  0.0138
Number of clusters:   379  Maximum cluster size: 1 
Call:
geeglm(formula = F3SCD_yn ~ nonferm_dairy_avg, data = ch_sensi,
    weights = ipwnonfat, id = pt, corstr = "independence")

 Coefficients:
                  Estimate  Std.err  Wald Pr(>|W|)    
(Intercept)       1.61e-01 3.41e-02 22.25  2.4e-06 ***
nonferm_dairy_avg 3.84e-05 2.15e-04  0.03     0.86    
---
Signif. codes:  0 '***' 0.001 '**' 0.01 '*' 0.05 '.' 0.1 ' ' 1

Correlation structure = independence 
Estimated Scale Parameters:

            Estimate Std.err
(Intercept)    0.137  0.0172
Number of clusters:   379  Maximum cluster size: 1 
Call:
geeglm(formula = F3SCD_yn ~ sugar_dairy_avg, data = ch_sensi,
    weights = ipwsg, id = pt, corstr = "independence")

 Coefficients:
                Estimate  Std.err  Wald Pr(>|W|)    
(Intercept)     0.103882 0.029823 12.13   0.0005 ***
sugar_dairy_avg 0.000337 0.000281  1.43   0.2316    
---
Signif. codes:  0 '***' 0.001 '**' 0.01 '*' 0.05 '.' 0.1 ' ' 1

Correlation structure = independence 
Estimated Scale Parameters:

            Estimate Std.err
(Intercept)    0.103  0.0192
Number of clusters:   379  Maximum cluster size: 1 
```

Memory

In [205…

```
#########TOTAL DAIRY################

msm.w <- geeglm(F2memory_cat ~ total_dairy_avg, data=ch_sensi, weights=ipwtotal, i
                corstr="independence")
summary(msm.w)

beta <- coef(msm.w)
SE <- coef(summary(msm.w))[,2]
lcl <- beta-qnorm(0.975)*SE
```

```
ucl <- beta+qnorm(0.975)*SE
mema <- cbind(beta*100, lcl*100, ucl*100)

##########FERMENTED DAIRY############

msm.w <- geeglm(F2memory_cat ~ ferm_dairy_avg, data=ch_sensi, weights=ipwferm, id=
                corstr="independence")
summary(msm.w)

beta <- coef(msm.w)
SE <- coef(summary(msm.w))[,2]
lcl <- beta-qnorm(0.975)*SE
ucl <- beta+qnorm(0.975)*SE
memb <- cbind(beta*100, lcl*100, ucl*100)

##########NON FERMENTED DAIRY########

msm.w <- geeglm(F2memory_cat ~ nonferm_dairy_avg, data=ch_sensi, weights=ipwnonfer
                corstr="independence")
summary(msm.w)

beta <- coef(msm.w)
SE <- coef(summary(msm.w))[,2]
lcl <- beta-qnorm(0.975)*SE
ucl <- beta+qnorm(0.975)*SE
memc <- cbind(beta*100, lcl*100, ucl*100)

##########FULL FAT DAIRY#############

msm.w <- geeglm(F2memory_cat ~ fullfat_dairy_avg, data=ch_sensi, weights=ipwff, id
                corstr="independence")
summary(msm.w)

beta <- coef(msm.w)
SE <- coef(summary(msm.w))[,2]
lcl <- beta-qnorm(0.975)*SE
ucl <- beta+qnorm(0.975)*SE
memd <- cbind(beta*100, lcl*100, ucl*100)

###########NON FAT DAIRY#############

msm.w <- geeglm(F2memory_cat ~ nonferm_dairy_avg, data=ch_sensi, weights=ipwnonfat
                corstr="independence")
summary(msm.w)

beta <- coef(msm.w)
SE <- coef(summary(msm.w))[,2]
lcl <- beta-qnorm(0.975)*SE
ucl <- beta+qnorm(0.975)*SE
meme <- cbind(beta*100, lcl*100, ucl*100)

###########SUGARY DAIRY#############

msm.w <- geeglm(F2memory_cat ~ sugar_dairy_avg, data=ch_sensi, weights=ipwsg, id=p
                corstr="independence")
summary(msm.w)

beta <- coef(msm.w)
SE <- coef(summary(msm.w))[,2]
lcl <- beta-qnorm(0.975)*SE
ucl <- beta+qnorm(0.975)*SE
memf <- cbind(beta*100, lcl*100, ucl*100)
```

```
Call:
geeglm(formula = F2memory_cat ~ total_dairy_avg, data = ch_sensi_ipw,
    weights = ipwtotal, id = pt, corstr = "independence")

 Coefficients:
                Estimate  Std.err Wald Pr(>|W|)
(Intercept)     0.086530 0.039242 4.86    0.027 *
total_dairy_avg 0.000332 0.000162 4.23    0.040 *
---
Signif. codes:  0 '***' 0.001 '**' 0.01 '*' 0.05 '.' 0.1 ' ' 1

Correlation structure = independence
Estimated Scale Parameters:

            Estimate Std.err
(Intercept)    0.141  0.0112
Number of clusters:   648  Maximum cluster size: 1
Call:
geeglm(formula = F2memory_cat ~ ferm_dairy_avg, data = ch_sensi_ipw,
    weights = ipwferm, id = pt, corstr = "independence")

 Coefficients:
               Estimate  Std.err Wald Pr(>|W|)
(Intercept)    0.116806 0.040990 8.12   0.0044 **
ferm_dairy_avg 0.000280 0.000261 1.15   0.2833
---
Signif. codes:  0 '***' 0.001 '**' 0.01 '*' 0.05 '.' 0.1 ' ' 1

Correlation structure = independence
Estimated Scale Parameters:

            Estimate Std.err
(Intercept)    0.138  0.0112
Number of clusters:   648  Maximum cluster size: 1
Call:
geeglm(formula = F2memory_cat ~ nonferm_dairy_avg, data = ch_sensi_ipw,
    weights = ipwnonferm, id = pt, corstr = "independence")

 Coefficients:
                  Estimate Std.err  Wald Pr(>|W|)
(Intercept)        0.13515 0.02255 35.93    2e-09 ***
nonferm_dairy_avg  0.00034 0.00024  2.01     0.16
---
Signif. codes:  0 '***' 0.001 '**' 0.01 '*' 0.05 '.' 0.1 ' ' 1

Correlation structure = independence
Estimated Scale Parameters:

            Estimate Std.err
(Intercept)    0.137  0.0103
Number of clusters:   648  Maximum cluster size: 1
```

```
Call:
geeglm(formula = F2memory_cat ~ fullfat_dairy_avg, data = ch_sensi_ipw,
    weights = ipwff, id = pt, corstr = "independence")

 Coefficients:
                  Estimate  Std.err Wald Pr(>|W|)
(Intercept)       0.102069 0.034025 9.00   0.0027 **
fullfat_dairy_avg 0.000255 0.000164 2.42   0.1195
---
Signif. codes:  0 '***' 0.001 '**' 0.01 '*' 0.05 '.' 0.1 ' ' 1

Correlation structure = independence
Estimated Scale Parameters:

            Estimate Std.err
(Intercept)    0.132  0.0106
Number of clusters:   648  Maximum cluster size: 1
Call:
geeglm(formula = F2memory_cat ~ nonferm_dairy_avg, data = ch_sensi_ipw,
    weights = ipwnonfat, id = pt, corstr = "independence")

 Coefficients:
                  Estimate  Std.err  Wald Pr(>|W|)
(Intercept)       0.132607 0.023461 31.95  1.6e-08 ***
nonferm_dairy_avg 0.000490 0.000329  2.22     0.14
---
Signif. codes:  0 '***' 0.001 '**' 0.01 '*' 0.05 '.' 0.1 ' ' 1

Correlation structure = independence
Estimated Scale Parameters:

            Estimate Std.err
(Intercept)    0.129  0.0114
Number of clusters:   648  Maximum cluster size: 1
Call:
geeglm(formula = F2memory_cat ~ sugar_dairy_avg, data = ch_sensi_ipw,
    weights = ipwsg, id = pt, corstr = "independence")

 Coefficients:
                 Estimate   Std.err  Wald Pr(>|W|)
(Intercept)      0.165088  0.016930 95.09   <2e-16 ***
sugar_dairy_avg -0.000227  0.000106  4.62    0.032 *
---
Signif. codes:  0 '***' 0.001 '**' 0.01 '*' 0.05 '.' 0.1 ' ' 1

Correlation structure = independence
Estimated Scale Parameters:

            Estimate Std.err
(Intercept)    0.125  0.0103
Number of clusters:   648  Maximum cluster size: 1
```

Verbal

In [178…

```
##########TOTAL DAIRY################

msm.w <- geeglm(F3verbal_cat ~ total_dairy_avg, data=ch_sensi, weights=ipwtotal, i
                corstr="independence")
summary(msm.w)

beta <- coef(msm.w)
SE <- coef(summary(msm.w))[,2]
lcl <- beta-qnorm(0.975)*SE
```

```
ucl <- beta+qnorm(0.975)*SE
vera <- cbind(beta*100, lcl*100, ucl*100)

##########FERMENTED DAIRY############

msm.w <- geeglm(F3verbal_cat ~ ferm_dairy_avg, data=ch_sensi, weights=ipwferm, id=
                corstr="independence")
summary(msm.w)

beta <- coef(msm.w)
SE <- coef(summary(msm.w))[,2]
lcl <- beta-qnorm(0.975)*SE
ucl <- beta+qnorm(0.975)*SE
verb <- cbind(beta*100, lcl*100, ucl*100)

##########NON FERMENTED DAIRY########

msm.w <- geeglm(F3verbal_cat ~ nonferm_dairy_avg, data=ch_sensi, weights=ipwnonfer
                corstr="independence")
summary(msm.w)

beta <- coef(msm.w)
SE <- coef(summary(msm.w))[,2]
lcl <- beta-qnorm(0.975)*SE
ucl <- beta+qnorm(0.975)*SE
verc <- cbind(beta*100, lcl*100, ucl*100)

##########FULL FAT DAIRY#############

msm.w <- geeglm(F3verbal_cat ~ fullfat_dairy_avg, data=ch_sensi, weights=ipwff, id
                corstr="independence")
summary(msm.w)

beta <- coef(msm.w)
SE <- coef(summary(msm.w))[,2]
lcl <- beta-qnorm(0.975)*SE
ucl <- beta+qnorm(0.975)*SE
verd <- cbind(beta*100, lcl*100, ucl*100)

###########NON FAT DAIRY#############

msm.w <- geeglm(F3verbal_cat ~ nonferm_dairy_avg, data=ch_sensi, weights=ipwnonfat
                corstr="independence")
summary(msm.w)

beta <- coef(msm.w)
SE <- coef(summary(msm.w))[,2]
lcl <- beta-qnorm(0.975)*SE
ucl <- beta+qnorm(0.975)*SE
vere <- cbind(beta*100, lcl*100, ucl*100)

###########SUGARY DAIRY#############

msm.w <- geeglm(F3verbal_cat ~ sugar_dairy_avg, data=ch_sensi, weights=ipwsg, id=p
                corstr="independence")
summary(msm.w)

beta <- coef(msm.w)
SE <- coef(summary(msm.w))[,2]
lcl <- beta-qnorm(0.975)*SE
ucl <- beta+qnorm(0.975)*SE
verf <- cbind(beta*100, lcl*100, ucl*100)
```

```
Call:
geeglm(formula = F3verbal_cat ~ total_dairy_avg, data = ch_sensi,
    weights = ipwtotal, id = pt, corstr = "independence")

 Coefficients:
                Estimate  Std.err   Wald Pr(>|W|)
(Intercept)     6.79e-01 4.99e-02 185.37   <2e-16 ***
total_dairy_avg 5.75e-05 1.50e-04   0.15      0.7
---
Signif. codes:  0 '***' 0.001 '**' 0.01 '*' 0.05 '.' 0.1 ' ' 1

Correlation structure = independence
Estimated Scale Parameters:

            Estimate Std.err
(Intercept)    0.213  0.0104
Number of clusters:   375  Maximum cluster size: 1
Call:
geeglm(formula = F3verbal_cat ~ ferm_dairy_avg, data = ch_sensi,
    weights = ipwferm, id = pt, corstr = "independence")

 Coefficients:
                Estimate   Std.err   Wald Pr(>|W|)
(Intercept)     0.705416  0.047470 220.82   <2e-16 ***
ferm_dairy_avg -0.000138  0.000227   0.37     0.54
---
Signif. codes:  0 '***' 0.001 '**' 0.01 '*' 0.05 '.' 0.1 ' ' 1

Correlation structure = independence
Estimated Scale Parameters:

            Estimate Std.err
(Intercept)    0.216  0.0105
Number of clusters:   375  Maximum cluster size: 1
Call:
geeglm(formula = F3verbal_cat ~ nonferm_dairy_avg, data = ch_sensi,
    weights = ipwnonferm, id = pt, corstr = "independence")

 Coefficients:
                  Estimate  Std.err   Wald Pr(>|W|)
(Intercept)       0.700357 0.037266 353.19   <2e-16 ***
nonferm_dairy_avg 0.000120 0.000256   0.22     0.64
---
Signif. codes:  0 '***' 0.001 '**' 0.01 '*' 0.05 '.' 0.1 ' ' 1

Correlation structure = independence
Estimated Scale Parameters:

            Estimate Std.err
(Intercept)    0.206  0.0112
Number of clusters:   375  Maximum cluster size: 1
```

```
Call:
geeglm(formula = F3verbal_cat ~ fullfat_dairy_avg, data = ch_sensi,
    weights = ipwff, id = pt, corstr = "independence")

 Coefficients:
                   Estimate   Std.err   Wald Pr(>|W|)
(Intercept)        7.27e-01  4.62e-02 247.47   <2e-16 ***
fullfat_dairy_avg -8.31e-05  1.76e-04   0.22     0.64
---
Signif. codes:  0 '***' 0.001 '**' 0.01 '*' 0.05 '.' 0.1 ' ' 1

Correlation structure = independence
Estimated Scale Parameters:

            Estimate Std.err
(Intercept)    0.206   0.011
Number of clusters:   375  Maximum cluster size: 1
Call:
geeglm(formula = F3verbal_cat ~ nonferm_dairy_avg, data = ch_sensi,
    weights = ipwnonfat, id = pt, corstr = "independence")

 Coefficients:
                  Estimate  Std.err   Wald Pr(>|W|)
(Intercept)       0.700357 0.037266 353.19   <2e-16 ***
nonferm_dairy_avg 0.000120 0.000256   0.22     0.64
---
Signif. codes:  0 '***' 0.001 '**' 0.01 '*' 0.05 '.' 0.1 ' ' 1

Correlation structure = independence
Estimated Scale Parameters:

            Estimate Std.err
(Intercept)    0.206  0.0112
Number of clusters:   375  Maximum cluster size: 1
Call:
geeglm(formula = F3verbal_cat ~ sugar_dairy_avg, data = ch_sensi,
    weights = ipwsg, id = pt, corstr = "independence")

 Coefficients:
                 Estimate   Std.err   Wald Pr(>|W|)
(Intercept)      0.793357  0.056316 198.46   <2e-16 ***
sugar_dairy_avg -0.000671  0.000478   1.97     0.16
---
Signif. codes:  0 '***' 0.001 '**' 0.01 '*' 0.05 '.' 0.1 ' ' 1

Correlation structure = independence
Estimated Scale Parameters:

            Estimate Std.err
(Intercept)    0.178  0.0254
Number of clusters:   375  Maximum cluster size: 1
```

Stroop

In [180…

```
#########TOTAL DAIRY################

msm.w <- geeglm(F3stroop_cat ~ total_dairy_avg, data=ch_sensi, weights=ipwtotal, i
                corstr="independence")
summary(msm.w)

beta <- coef(msm.w)
SE <- coef(summary(msm.w))[,2]
lcl <- beta-qnorm(0.975)*SE
```

```
ucl <- beta+qnorm(0.975)*SE
stra <- cbind(beta*100, lcl*100, ucl*100)

##########FERMENTED DAIRY############

msm.w <- geeglm(F3stroop_cat ~ ferm_dairy_avg, data=ch_sensi, weights=ipwferm, id=
                corstr="independence")
summary(msm.w)

beta <- coef(msm.w)
SE <- coef(summary(msm.w))[,2]
lcl <- beta-qnorm(0.975)*SE
ucl <- beta+qnorm(0.975)*SE
strb <- cbind(beta*100, lcl*100, ucl*100)

##########NON FERMENTED DAIRY########

msm.w <- geeglm(F3stroop_cat ~ nonferm_dairy_avg, data=ch_sensi, weights=ipwnonfer
                corstr="independence")
summary(msm.w)

beta <- coef(msm.w)
SE <- coef(summary(msm.w))[,2]
lcl <- beta-qnorm(0.975)*SE
ucl <- beta+qnorm(0.975)*SE
strc <- cbind(beta*100, lcl*100, ucl*100)

##########FULL FAT DAIRY#############

msm.w <- geeglm(F3stroop_cat ~ fullfat_dairy_avg, data=ch_sensi, weights=ipwff, id
                corstr="independence")
summary(msm.w)

beta <- coef(msm.w)
SE <- coef(summary(msm.w))[,2]
lcl <- beta-qnorm(0.975)*SE
ucl <- beta+qnorm(0.975)*SE
strd <- cbind(beta*100, lcl*100, ucl*100)

###########NON FAT DAIRY#############

msm.w <- geeglm(F3stroop_cat ~ nonferm_dairy_avg, data=ch_sensi, weights=ipwnonfat
                corstr="independence")
summary(msm.w)

beta <- coef(msm.w)
SE <- coef(summary(msm.w))[,2]
lcl <- beta-qnorm(0.975)*SE
ucl <- beta+qnorm(0.975)*SE
stre <- cbind(beta*100, lcl*100, ucl*100)

###########SUGARY DAIRY#############

msm.w <- geeglm(F3stroop_cat ~ sugar_dairy_avg, data=ch_sensi, weights=ipwsg, id=p
                corstr="independence")
summary(msm.w)

beta <- coef(msm.w)
SE <- coef(summary(msm.w))[,2]
lcl <- beta-qnorm(0.975)*SE
ucl <- beta+qnorm(0.975)*SE
strf <- cbind(beta*100, lcl*100, ucl*100)
```

```
Call:
geeglm(formula = F3stroop_cat ~ total_dairy_avg, data = ch_sensi,
    weights = ipwtotal, id = pt, corstr = "independence")

 Coefficients:
                Estimate  Std.err  Wald Pr(>|W|)
(Intercept)     0.249061 0.052657 22.37  2.2e-06 ***
total_dairy_avg 0.000062 0.000171  0.13     0.72
---
Signif. codes:  0 '***' 0.001 '**' 0.01 '*' 0.05 '.' 0.1 ' ' 1

Correlation structure = independence
Estimated Scale Parameters:

            Estimate Std.err
(Intercept)    0.194  0.0125
Number of clusters:   380  Maximum cluster size: 1
Call:
geeglm(formula = F3stroop_cat ~ ferm_dairy_avg, data = ch_sensi,
    weights = ipwferm, id = pt, corstr = "independence")

 Coefficients:
                Estimate   Std.err  Wald Pr(>|W|)
(Intercept)     2.69e-01  4.96e-02 29.39  5.9e-08 ***
ferm_dairy_avg -2.27e-05  2.27e-04  0.01     0.92
---
Signif. codes:  0 '***' 0.001 '**' 0.01 '*' 0.05 '.' 0.1 ' ' 1

Correlation structure = independence
Estimated Scale Parameters:

            Estimate Std.err
(Intercept)    0.195  0.0128
Number of clusters:   380  Maximum cluster size: 1
Call:
geeglm(formula = F3stroop_cat ~ nonferm_dairy_avg, data = ch_sensi,
    weights = ipwnonferm, id = pt, corstr = "independence")

 Coefficients:
                   Estimate   Std.err  Wald Pr(>|W|)
(Intercept)        0.284204  0.042895 43.90  3.5e-11 ***
nonferm_dairy_avg -0.000204  0.000249  0.67     0.41
---
Signif. codes:  0 '***' 0.001 '**' 0.01 '*' 0.05 '.' 0.1 ' ' 1

Correlation structure = independence
Estimated Scale Parameters:

            Estimate Std.err
(Intercept)    0.196  0.0136
Number of clusters:   380  Maximum cluster size: 1
```

```
Call:
geeglm(formula = F3stroop_cat ~ fullfat_dairy_avg, data = ch_sensi,
    weights = ipwff, id = pt, corstr = "independence")

 Coefficients:
                  Estimate  Std.err  Wald Pr(>|W|)
(Intercept)       2.46e-01 5.03e-02 23.86    1e-06 ***
fullfat_dairy_avg 5.18e-05 1.89e-04  0.07     0.78
---
Signif. codes:  0 '***' 0.001 '**' 0.01 '*' 0.05 '.' 0.1 ' ' 1

Correlation structure = independence
Estimated Scale Parameters:

            Estimate Std.err
(Intercept)     0.19  0.0129
Number of clusters:   380  Maximum cluster size: 1
Call:
geeglm(formula = F3stroop_cat ~ nonferm_dairy_avg, data = ch_sensi,
    weights = ipwnonfat, id = pt, corstr = "independence")

 Coefficients:
                   Estimate   Std.err  Wald Pr(>|W|)
(Intercept)        0.284204  0.042895 43.90  3.5e-11 ***
nonferm_dairy_avg -0.000204  0.000249  0.67     0.41
---
Signif. codes:  0 '***' 0.001 '**' 0.01 '*' 0.05 '.' 0.1 ' ' 1

Correlation structure = independence
Estimated Scale Parameters:

            Estimate Std.err
(Intercept)    0.196  0.0136
Number of clusters:   380  Maximum cluster size: 1
Call:
geeglm(formula = F3stroop_cat ~ sugar_dairy_avg, data = ch_sensi,
    weights = ipwsg, id = pt, corstr = "independence")

 Coefficients:
                 Estimate   Std.err Wald Pr(>|W|)
(Intercept)      2.80e-01  8.95e-02 9.82   0.0017 **
sugar_dairy_avg -9.59e-05  6.16e-04 0.02   0.8764
---
Signif. codes:  0 '***' 0.001 '**' 0.01 '*' 0.05 '.' 0.1 ' ' 1

Correlation structure = independence
Estimated Scale Parameters:

            Estimate Std.err
(Intercept)      0.2  0.0293
Number of clusters:   380  Maximum cluster size: 1
```

DO40

In [179…

```
##########TOTAL DAIRY################

msm.w <- geeglm(F3do40_cat ~ total_dairy_avg, data=ch_sensi, weights=ipwtotal, id=
                corstr="independence")
summary(msm.w)

beta <- coef(msm.w)
SE <- coef(summary(msm.w))[,2]
lcl <- beta-qnorm(0.975)*SE
```

```
ucl <- beta+qnorm(0.975)*SE
doa <- cbind(beta*100, lcl*100, ucl*100)

##########FERMENTED DAIRY############

msm.w <- geeglm(F3do40_cat ~ ferm_dairy_avg, data=ch_sensi, weights=ipwferm, id=pt
                corstr="independence")
summary(msm.w)

beta <- coef(msm.w)
SE <- coef(summary(msm.w))[,2]
lcl <- beta-qnorm(0.975)*SE
ucl <- beta+qnorm(0.975)*SE
dob <- cbind(beta*100, lcl*100, ucl*100)

##########NON FERMENTED DAIRY########

msm.w <- geeglm(F3do40_cat ~ nonferm_dairy_avg, data=ch_sensi, weights=ipwnonferm,
                corstr="independence")
summary(msm.w)

beta <- coef(msm.w)
SE <- coef(summary(msm.w))[,2]
lcl <- beta-qnorm(0.975)*SE
ucl <- beta+qnorm(0.975)*SE
doc <- cbind(beta*100, lcl*100, ucl*100)

##########FULL FAT DAIRY#############

msm.w <- geeglm(F3do40_cat ~ fullfat_dairy_avg, data=ch_sensi, weights=ipwff, id=p
                corstr="independence")
summary(msm.w)

beta <- coef(msm.w)
SE <- coef(summary(msm.w))[,2]
lcl <- beta-qnorm(0.975)*SE
ucl <- beta+qnorm(0.975)*SE
dod <- cbind(beta*100, lcl*100, ucl*100)

###########NON FAT DAIRY#############

msm.w <- geeglm(F3do40_cat ~ nonfat_dairy_avg, data=ch_sensi, weights=ipwnonfat, i
                corstr="independence")
summary(msm.w)

beta <- coef(msm.w)
SE <- coef(summary(msm.w))[,2]
lcl <- beta-qnorm(0.975)*SE
ucl <- beta+qnorm(0.975)*SE
doe <- cbind(beta*100, lcl*100, ucl*100)

############SUGARY DAIRY#############

msm.w <- geeglm(F3do40_cat ~ sugar_dairy_avg, data=ch_sensi, weights=ipwsg, id=pt,
                corstr="independence")
summary(msm.w)

beta <- coef(msm.w)
SE <- coef(summary(msm.w))[,2]
lcl <- beta-qnorm(0.975)*SE
ucl <- beta+qnorm(0.975)*SE
dof <- cbind(beta*100, lcl*100, ucl*100)
```

```
Call:
geeglm(formula = F3do40_cat ~ total_dairy_avg, data = ch_sensi,
    weights = ipwtotal, id = pt, corstr = "independence")

 Coefficients:
                Estimate  Std.err  Wald Pr(>|W|)
(Intercept)     1.41e-01 3.52e-02 16.07  6.1e-05 ***
total_dairy_avg 6.93e-05 1.17e-04  0.35     0.55
---
Signif. codes:  0 '***' 0.001 '**' 0.01 '*' 0.05 '.' 0.1 ' ' 1

Correlation structure = independence
Estimated Scale Parameters:

            Estimate Std.err
(Intercept)    0.133   0.014
Number of clusters:   377  Maximum cluster size: 1
Call:
geeglm(formula = F3do40_cat ~ ferm_dairy_avg, data = ch_sensi,
    weights = ipwferm, id = pt, corstr = "independence")

 Coefficients:
               Estimate  Std.err Wald Pr(>|W|)
(Intercept)    1.62e-01 3.82e-02   18  2.2e-05 ***
ferm_dairy_avg 1.21e-05 1.77e-04    0     0.95
---
Signif. codes:  0 '***' 0.001 '**' 0.01 '*' 0.05 '.' 0.1 ' ' 1

Correlation structure = independence
Estimated Scale Parameters:

            Estimate Std.err
(Intercept)    0.137  0.0149
Number of clusters:   377  Maximum cluster size: 1
Call:
geeglm(formula = F3do40_cat ~ nonferm_dairy_avg, data = ch_sensi,
    weights = ipwnonferm, id = pt, corstr = "independence")

 Coefficients:
                  Estimate  Std.err  Wald Pr(>|W|)
(Intercept)       0.139191 0.026871 26.83  2.2e-07 ***
nonferm_dairy_avg 0.000106 0.000192  0.31     0.58
---
Signif. codes:  0 '***' 0.001 '**' 0.01 '*' 0.05 '.' 0.1 ' ' 1

Correlation structure = independence
Estimated Scale Parameters:

            Estimate Std.err
(Intercept)    0.125  0.0143
Number of clusters:   377  Maximum cluster size: 1
```

```
Call:
geeglm(formula = F3do40_cat ~ fullfat_dairy_avg, data = ch_sensi,
    weights = ipwff, id = pt, corstr = "independence")

 Coefficients:
                   Estimate   Std.err  Wald Pr(>|W|)
(Intercept)        1.83e-01  5.12e-02 12.80  0.00035 ***
fullfat_dairy_avg -2.94e-05  1.79e-04  0.03  0.86981
---
Signif. codes:  0 '***' 0.001 '**' 0.01 '*' 0.05 '.' 0.1 ' ' 1

Correlation structure = independence
Estimated Scale Parameters:

            Estimate Std.err
(Intercept)    0.146  0.0171
Number of clusters:   377  Maximum cluster size: 1
Call:
geeglm(formula = F3do40_cat ~ nonfat_dairy_avg, data = ch_sensi,
    weights = ipwnonfat, id = pt, corstr = "independence")

 Coefficients:
                 Estimate  Std.err Wald Pr(>|W|)
(Intercept)      1.47e-01 2.33e-02 39.9  2.7e-10 ***
nonfat_dairy_avg 1.21e-06 2.34e-04  0.0        1
---
Signif. codes:  0 '***' 0.001 '**' 0.01 '*' 0.05 '.' 0.1 ' ' 1

Correlation structure = independence
Estimated Scale Parameters:

            Estimate Std.err
(Intercept)    0.125  0.0142
Number of clusters:   377  Maximum cluster size: 1
Call:
geeglm(formula = F3do40_cat ~ sugar_dairy_avg, data = ch_sensi,
    weights = ipwsg, id = pt, corstr = "independence")

 Coefficients:
                Estimate  Std.err Wald Pr(>|W|)
(Intercept)      0.38800  0.14718 6.95   0.0084 **
sugar_dairy_avg -0.00141  0.00101 1.93   0.1647
---
Signif. codes:  0 '***' 0.001 '**' 0.01 '*' 0.05 '.' 0.1 ' ' 1

Correlation structure = independence
Estimated Scale Parameters:

            Estimate Std.err
(Intercept)    0.213  0.0301
Number of clusters:   377  Maximum cluster size: 1
```

CERAD

In [181…

```
#########TOTAL DAIRY################

msm.w <- geeglm(F3CERAD_cat ~ total_dairy_avg, data=ch_sensi, weights=ipwtotal, id
                corstr="independence")
summary(msm.w)

beta <- coef(msm.w)
SE <- coef(summary(msm.w))[,2]
lcl <- beta-qnorm(0.975)*SE
```

```
ucl <- beta+qnorm(0.975)*SE
cera <- cbind(beta*100, lcl*100, ucl*100)

##########FERMENTED DAIRY############

msm.w <- geeglm(F3CERAD_cat ~ ferm_dairy_avg, data=ch_sensi, weights=ipwferm, id=p
                corstr="independence")
summary(msm.w)

beta <- coef(msm.w)
SE <- coef(summary(msm.w))[,2]
lcl <- beta-qnorm(0.975)*SE
ucl <- beta+qnorm(0.975)*SE
cerb <- cbind(beta*100, lcl*100, ucl*100)

##########NON FERMENTED DAIRY########

msm.w <- geeglm(F3CERAD_cat ~ nonferm_dairy_avg, data=ch_sensi, weights=ipwnonferm
                corstr="independence")
summary(msm.w)

beta <- coef(msm.w)
SE <- coef(summary(msm.w))[,2]
lcl <- beta-qnorm(0.975)*SE
ucl <- beta+qnorm(0.975)*SE
cerc <- cbind(beta*100, lcl*100, ucl*100)

##########FULL FAT DAIRY#############

msm.w <- geeglm(F3CERAD_cat ~ fullfat_dairy_avg, data=ch_sensi, weights=ipwff, id=
                corstr="independence")
summary(msm.w)

beta <- coef(msm.w)
SE <- coef(summary(msm.w))[,2]
lcl <- beta-qnorm(0.975)*SE
ucl <- beta+qnorm(0.975)*SE
cerd <- cbind(beta*100, lcl*100, ucl*100)

###########NON FAT DAIRY#############

msm.w <- geeglm(F3CERAD_cat ~ nonferm_dairy_avg, data=ch_sensi, weights=ipwnonfat,
                corstr="independence")
summary(msm.w)

beta <- coef(msm.w)
SE <- coef(summary(msm.w))[,2]
lcl <- beta-qnorm(0.975)*SE
ucl <- beta+qnorm(0.975)*SE
cere <- cbind(beta*100, lcl*100, ucl*100)

############SUGARY DAIRY#############

msm.w <- geeglm(F3CERAD_cat ~ sugar_dairy_avg, data=ch_sensi, weights=ipwsg, id=pt
                corstr="independence")
summary(msm.w)

beta <- coef(msm.w)
SE <- coef(summary(msm.w))[,2]
lcl <- beta-qnorm(0.975)*SE
ucl <- beta+qnorm(0.975)*SE
cerf <- cbind(beta*100, lcl*100, ucl*100)
```

```
Call:
geeglm(formula = F3CERAD_cat ~ total_dairy_avg, data = ch_sensi,
    weights = ipwtotal, id = pt, corstr = "independence")

 Coefficients:
                 Estimate   Std.err Wald Pr(>|W|)
(Intercept)      0.472564  0.058284 65.7  5.6e-16 ***
total_dairy_avg -0.000081  0.000183  0.2     0.66
---
Signif. codes:  0 '***' 0.001 '**' 0.01 '*' 0.05 '.' 0.1 ' ' 1

Correlation structure = independence
Estimated Scale Parameters:

            Estimate Std.err
(Intercept)    0.248 0.00268
Number of clusters:   384  Maximum cluster size: 1
Call:
geeglm(formula = F3CERAD_cat ~ ferm_dairy_avg, data = ch_sensi,
    weights = ipwferm, id = pt, corstr = "independence")

 Coefficients:
                Estimate   Std.err  Wald Pr(>|W|)
(Intercept)     0.496113  0.056529 77.02   <2e-16 ***
ferm_dairy_avg -0.000123  0.000261  0.22     0.64
---
Signif. codes:  0 '***' 0.001 '**' 0.01 '*' 0.05 '.' 0.1 ' ' 1

Correlation structure = independence
Estimated Scale Parameters:

            Estimate Std.err
(Intercept)    0.249 0.00137
Number of clusters:   384  Maximum cluster size: 1
Call:
geeglm(formula = F3CERAD_cat ~ nonferm_dairy_avg, data = ch_sensi,
    weights = ipwnonferm, id = pt, corstr = "independence")

 Coefficients:
                   Estimate   Std.err   Wald Pr(>|W|)
(Intercept)        0.479067  0.043803 119.62   <2e-16 ***
nonferm_dairy_avg -0.000305  0.000274   1.24     0.27
---
Signif. codes:  0 '***' 0.001 '**' 0.01 '*' 0.05 '.' 0.1 ' ' 1

Correlation structure = independence
Estimated Scale Parameters:

            Estimate Std.err
(Intercept)    0.247 0.00266
Number of clusters:   384  Maximum cluster size: 1
```

```
Call:
geeglm(formula = F3CERAD_cat ~ fullfat_dairy_avg, data = ch_sensi,
    weights = ipwff, id = pt, corstr = "independence")

 Coefficients:
                   Estimate   Std.err  Wald Pr(>|W|)
(Intercept)        0.509996  0.065624 60.40  7.8e-15 ***
fullfat_dairy_avg -0.000246  0.000229  1.15     0.28
---
Signif. codes:  0 '***' 0.001 '**' 0.01 '*' 0.05 '.' 0.1 ' ' 1

Correlation structure = independence
Estimated Scale Parameters:

            Estimate Std.err
(Intercept)    0.247 0.00258
Number of clusters:   384  Maximum cluster size: 1
Call:
geeglm(formula = F3CERAD_cat ~ nonferm_dairy_avg, data = ch_sensi,
    weights = ipwnonfat, id = pt, corstr = "independence")

 Coefficients:
                   Estimate   Std.err   Wald Pr(>|W|)
(Intercept)        0.479067  0.043803 119.62   <2e-16 ***
nonferm_dairy_avg -0.000305  0.000274   1.24     0.27
---
Signif. codes:  0 '***' 0.001 '**' 0.01 '*' 0.05 '.' 0.1 ' ' 1

Correlation structure = independence
Estimated Scale Parameters:

            Estimate Std.err
(Intercept)    0.247 0.00266
Number of clusters:   384  Maximum cluster size: 1
Call:
geeglm(formula = F3CERAD_cat ~ sugar_dairy_avg, data = ch_sensi,
    weights = ipwsg, id = pt, corstr = "independence")

 Coefficients:
                Estimate  Std.err  Wald Pr(>|W|)
(Intercept)     0.359348 0.097770 13.51  0.00024 ***
sugar_dairy_avg 0.000939 0.000688  1.86  0.17219
---
Signif. codes:  0 '***' 0.001 '**' 0.01 '*' 0.05 '.' 0.1 ' ' 1

Correlation structure = independence
Estimated Scale Parameters:

            Estimate Std.err
(Intercept)    0.236  0.0195
Number of clusters:   384  Maximum cluster size: 1
```

In [182…

```
totaleffects_sensi <- data.frame(c(cdra[2,],cdrb[2,],cdrc[2,],cdrd[2,], cdre[2,],c
                        c(scda[2,],scdb[2,],scdc[2,],scdd[2,],scde[2,],scdf[2,]),
                        c(mema[2,],memb[2,],memc[2,],memd[2,],meme[2,],memf[2,]),
                        c(vera[2,],verb[2,],verc[2,],verd[2,],vere[2,],verf[2,]),
                        c(stra[2,],strb[2,],strc[2,],strd[2,],stre[2,],strf[2,]),
                        c(doa[2,],dob[2,],doc[2,],dod[2,],doe[2,],dof[2,]),
                        c(cera[2,],cerb[2,],cerc[2,],cerd[2,],cere[2,],cerf[2,]))
write.csv(totaleffects_sensi, "totaleffects_sensi.csv")
```

Dairy and Cognition. Substitution effects

Author: Natalia Ortega

Last updated: 06.06.2023

Background

The current analysis is part of the project "Prospective international study on dairy and inflammation on cognitive decline". We will use PsyCoLaus data to answer the 1st goal which main aim is to assess the long-term effect of total dairy intake and subtypes of dairy (fermented vs. non-fermented, low vs. full fat) on cognitive function. This is the 2nd file computing substitution effects (relative effects).

Contents

1. Loading packages and datasets
2. Covariate coding
3. Selection criteria 3.1 Not recruited for PsyColaus 3.2 Lost to follow up (LTFU)
4. Data description
   - 4.1 Outcome coding and viz
   - 4.2 Exposure coding and viz
   - 4.3 Table 1
5. Missingness
6. IPTW and MSMs
7. Sensitivy analysis for substition effects

The details on each method are available in SAP_Aim1.docx version 1.0 (Not approved version).

1. Loading packages and files

Packages

In [1]:
```
library(tidyverse)
library(MASS)
library(ggpubr)
library(tableone)
library(gridExtra)
library(rlang)
library(geepack)
library(naniar)
library(WeightIt)
library(cobalt)
library(survey)
#Ignore or not warnings
```

```
options(warn=-1)
dodge = position_dodge(width=0.1)
```

```
── Attaching core tidyverse packages ──────────────────────── tidyverse 2.0.0 ──
✔ dplyr     1.1.2     ✔ readr     2.1.4
✔ forcats   1.0.0     ✔ stringr   1.5.0
✔ ggplot2   3.4.2     ✔ tibble    3.2.1
✔ lubridate 1.9.2     ✔ tidyr     1.3.0
✔ purrr     1.0.1
── Conflicts ────────────────────────────────────────── tidyverse_conflicts() ──
✖ dplyr::filter() masks stats::filter()
✖ dplyr::lag()    masks stats::lag()
ℹ Use the conflicted package (<http://conflicted.r-lib.org/>) to force all conflic
ts to become errors

Attache Paket: 'MASS'


Das folgende Objekt ist maskiert 'package:dplyr':

    select



Attache Paket: 'gridExtra'


Das folgende Objekt ist maskiert 'package:dplyr':

    combine



Attache Paket: 'rlang'


Die folgenden Objekte sind maskiert von 'package:purrr':

    %@%, flatten, flatten_chr, flatten_dbl, flatten_int, flatten_lgl,
    flatten_raw, invoke, splice


 cobalt (Version 4.5.1, Build Date: 2023-04-27)

Lade nötiges Paket: grid

Lade nötiges Paket: Matrix


Attache Paket: 'Matrix'


Die folgenden Objekte sind maskiert von 'package:tidyr':

    expand, pack, unpack


Lade nötiges Paket: survival


Attache Paket: 'survey'


Das folgende Objekt ist maskiert 'package:graphics':

    dotchart
```

Datasets

In [2]:
```
baseline <- read.csv ("C:/Users/no22t395/OneDrive - Universitaet Bern/CoLaus doc/C(
fu1 <- read.csv ("C:/Users/no22t395/OneDrive - Universitaet Bern/CoLaus doc/COLAUS
fu2 <- read.csv ("C:/Users/no22t395/OneDrive - Universitaet Bern/CoLaus doc/COLAUS
fu3 <- read.csv ("C:/Users/no22t395/OneDrive - Universitaet Bern/CoLaus doc/COLAUS
cog <- read.csv ("C:/Users/no22t395/OneDrive - Universitaet Bern/CoLaus doc/COLAUS
```

2. Covariate coding

In [3]:
```
#merge datasets

ch <- dplyr::full_join(baseline, fu1, by = c("pt"))
ch <- ch %>%
    dplyr::full_join(fu2, by = c("pt")) %>%
    dplyr::full_join(fu3, by = c("pt")) %>%
    dplyr::full_join(cog, by = c("pt"))

#summary(ch) see 1st file of the addition effects for Summary of the initial datase
```

Recoding baseline covariates

Sex (sex), MMSE (MME) and occupation (job_curr8), total calories (F1sumtot1, F2sumtot1), smoking (F1sbsmk), bmi (F1BMI), depression (F1depressed), diabetes (F1dbtld) and the cognitive tests are coded as needed in the original dataset. See below.

In [4]:
```
ch <- ch %>% dplyr::rename(MMSE_b = F1MME,
                          occ_b = F1job_curr8,
                          bmi_b = F1BMI, #Ideally we would use Bioimpedance
                          sm_b = F1sbsmk,
                          depre_b = F1depressed,
                          totalcal_b = F1sumtot1,
                          F2totalcal = F2sumtot1,
                          famincome_b = F2income2, #many NAs
                          F1datquest = F1datblood,
                          diab_b = F1dbtld,
                          memory_b = F1_B16_REC,#max 48
                          verbal_b = F1_FV_A_TOT,
                          stroop_b = F1_STROOP_IFORTE,#max 24
                          do40_b = F1_DO40, #max 40
                          CERAD_b = F1_CERAD_TOT, #max 11
                          CDR_b = F1_CDR,
                          F2memory = F2_B16_REC,#max 48
                          F2verbal = F2_FV_A_TOT,
                          F2stroop = F2_STROOP_IFORTE,#max 24
                          F2do40 = F2_DO40, #max 40
                          F2CERAD = F2_CERAD_TOT, #max 11
                          F2CDR = F2_CDR,
                          F3memory = F3_B16_REC,#max 48
                          F3verbal = F3_FV_A_TOT,
                          F3stroop = F3_STROOP_IFORTE,#max 24
                          F3do40 = F3_DO40, #max 40
                          F3CERAD = F3_CERAD_TOT, #max 11
                          F3CDR = F3_CDR)
ch[ch == "."] <- NA
```

```
ch$occ_b <- as.factor(ch$occ_b)
ch$sm_b <- as.factor(ch$sm_b)
ch$famincome_b <- as.factor(ch$famincome_b)

# Recode cognitive funciton variables as numeric
ch <- ch %>%
        mutate_at(c("F3_QPC_B","F3_QPC_A","F3_QPC_1","F3_QPC_2","F3_QPC_3","F3_QPC_
                    'F3_QPC_8', 'F3_QPC_7','F3_QPC_6',"F2_QPC_B","F2_QPC_A","F2_QP
                    "F2_QPC_4","F2_QPC_5",'F2_QPC_8', 'F2_QPC_7',"F2_QPC_6","F1_QP
                    "F1_QPC_2","F1_QPC_3","F1_QPC_4","F1_QPC_5",'F1_QPC_8', 'F1_QP
                    "stroop_b","do40_b","CERAD_b","F2memory","F2verbal","F2stroop",
                    "F3verbal","F3stroop", "F3do40", "F3CERAD"), as.numeric)

#We pool the few participants with mild cognitive impairment and recode them to ve
ch$F2CDR[ch$F2CDR == "1.0"] <- "0.5"
```

Education

In [5]:

```
ch <- ch %>% mutate(edu = ifelse(edtyp == 0 |edtyp == 1 | edtyp == 2, 1, #elementa
                           ifelse(edtyp == 3, 2,                    #high sch
                                 ifelse(edtyp == 4, 3, NA))))          #supe

ch$edu <- factor(ch$edu,
levels = c(1,2,3),
labels = c("Elementary", "High school", "Superior"))

table(ch$edu)
```

```
 Elementary High school    Superior
       4511         888        1320
```

Age at recruitment

In [6]:

```
rep_str = c('jan'='-01-','feb'='-02-','mar'='-03-','apr'='-04-','may'='-05-','jun'
            'sep'='-09-', 'oct'='-10-','nov'='-11-','dec'='-12-')
ch$datbirth <- str_replace_all(ch$datbirth, rep_str)
ch$datexam <- str_replace_all(ch$datexam, rep_str)
ch$F1datquest <- str_replace_all(ch$F1datquest, rep_str)

table(is.na(ch$datbirth))

ch$datbirth <- as.Date(ch$datbirth,
  format = "%d-%m-%Y")
ch$datexam <- as.Date(ch$datexam,
  format = "%d-%m-%Y")
ch$F1datquest <- as.Date(ch$F1datquest,
  format = "%d-%m-%Y")

ch$age_recruit <- difftime(ch$datexam, ch$datbirth, units = "days")
ch$age_recruit <- as.integer(round(ch$age_recruit/365,digits = 0))

ch$age_b <- difftime(ch$F1datquest, ch$datbirth, units = "days")
ch$age_b <- as.integer(round(ch$age_b/365,digits = 0))

ch <- ch %>%
    dplyr::mutate(age_cat = ifelse(age_b < 70, 1,
                              ifelse(age_b < 75, 2,
                                    ifelse(age_b >= 75, 3, NA))))
```

```
FALSE  TRUE
 6733    13
```

BMI categorical

In [7]:
```
ch$bmi_cat <- ifelse(ch$bmi_b < 25, 1,
                     ifelse(ch$bmi_b < 30, 2,
                           ifelse(ch$bmi_b >= 30, 3, NA)))
table(ch$bmi_cat)
```

```
   1    2    3
2180 1960  859
```

Past major cardiovascular event

In [8]:
```
ch <- ch %>% mutate(cvevent_b = ifelse(cmp == 1 | hdc == 1 |hdv == 1 | chf == 1 |c
                                       strk == 1 | ccth == 1 | cabg == 1 |pcin == 
                                       F1cmp == 1 | F1hdv == 1 | F1chf == 1 |F1cad
                                       F1strk == 1 | F1ccth == 1 | F1cabg == 1 , 1
table(ch$cvevent_b)
```

```
   0    1
4320  882
```

Hypertension

In [9]:
```
ch <- ch %>% mutate(HTA_b = ifelse(F1antiHTA == 1 | F1HTA == 1, 1, 0))
```

Alcohol use/day

In [10]:
```
ch <- ch %>% mutate(alc_b = ifelse(F1alcool1 == 0, 0,
                                   ifelse(sex == 0 & F1alcool1 == 1, 1,
                                      ifelse(sex == 0 & (F1alcool1 == 2 | F1alcool
                                        ifelse(sex == 0 & (F1alcool1 == 4 | F1alco
                                            ifelse(sex == 1 & (F1alcool1 == 1|F1al
                                                ifelse(sex == 1 & (F1alcool1 == 3
                                                    ifelse(sex == 1 & (F1alcool1 =
table(ch$alc_b)
```

```
   0    1    2
1281 2601 1182
```

Physical activity

In [11]:
```
# Find tertiles
vTert = quantile(na.omit(ch$F1etsem), c(0:3/3))
vTert

ch <- ch %>% mutate(pa_b = ifelse(is.na(F1etsem), NA,
                                  ifelse(F1etsem < 16200.16, "Low",
                                        ifelse(F1etsem < 19807.82, "Medium",
                                              ifelse(F1etsem < 44913.35, "High"))))
```

0%: 10662.4075646646 **33.33333%:** 16200.1594910438 **66.66667%:** 19807.822075486 **100%:** 44913.349235205

Time after recruitment

In [12]:
```
ch["F2datquest"][ch["F2datquest"] == "03jul2004"] <- "03jul2014"

ch$F2datquest <- str_replace_all(ch$F2datquest, rep_str)
ch$F3datquest <- str_replace_all(ch$F3datquest, rep_str)

ch$F2datquest <- as.Date(ch$F2datquest,
  format = "%d-%m-%Y")
ch$F3datquest <- as.Date(ch$F3datquest,
```

```
  format = "%d-%m-%Y")

ch$futime_b <- difftime(ch$F1datquest, ch$datexam, units = "days")
ch$futime_b <- as.numeric(round(ch$futime_b/365,digits = 2))

ch$futime_F1 <- difftime(ch$F2datquest, ch$F1datquest, units = "days")
ch$futime_F1 <- as.numeric(round(ch$futime_F1/365,digits = 2))

ch$futime_F2 <- difftime(ch$F3datquest, ch$F1datquest, units = "days")
ch$futime_F2 <- as.numeric(round(ch$futime_F2/365,digits = 2))
```

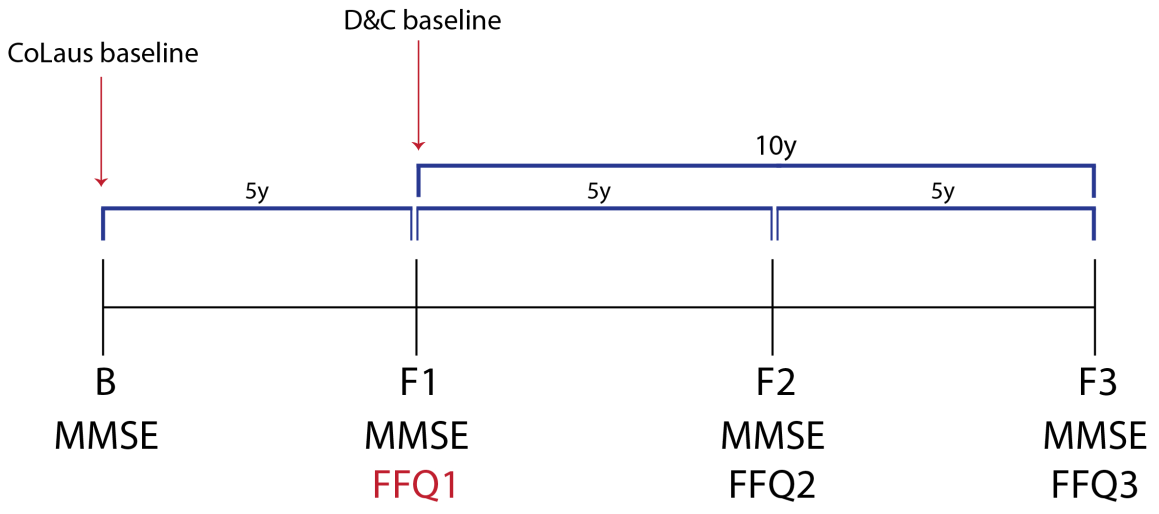

In [13]:
```
#Follow-up times (mean and 95% CI)

mean(na.omit(c(ch$futime_b))) #5.57 (5.56-5.58) years
mean(na.omit(c(ch$futime_F1))) #10.86 (10.85-10.88) years
mean(na.omit(c(ch$futime_F2))) #14.59 (14.58-14.61) years

t.test(na.omit(c(ch$futime_b)))$"conf.int"
t.test(na.omit(c(ch$futime_F1)))$"conf.int"
t.test(na.omit(c(ch$futime_F2)))$"conf.int"
```

5.56818720379147

5.27936437718277

9.04193557748114

5.55559762534958 · 5.58077678223336

5.26214785011949 · 5.29658090424605

9.02407560415633 · 9.05979555080594

In [14]:
```
#pdf(file = "a.pdf",
#    width = 4,
#    height = 4)

fut1 <- data.frame(FUT = c(ch$futime_F1), tp = c("F1"))
fut2 <- data.frame(FUT = c(ch$futime_F2), tp = c("F2"))
id <- c(1:13609)
follow_ups <- bind_rows(fut1,fut2)


ggplot(follow_ups, aes(x = FUT))+
          geom_density(aes(fill = tp), alpha = 0.4) +
          xlim(0, 13)+
          scale_color_manual(values = c("#00AFBB","#868686FF", "#EFC000FF"))+
          scale_fill_manual(values = c("#00AFBB","#868686FF", "#EFC000FF"))
```

```
#dev.off()
```

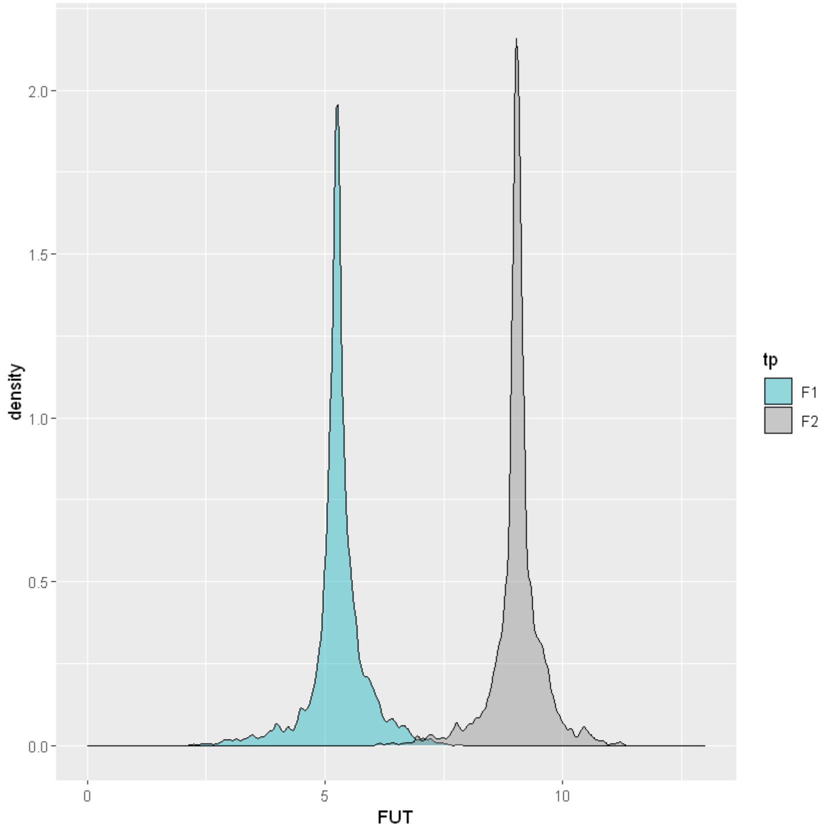

3. Selection criteria

PsyCoLaus inclusion criteria

All 35 to 66-year old subjects of the CoLaus sample (n = 5,535), were invited by letters to also participate in the psychiatric evaluation. Those who did not respond to the letter were contacted by phone. All subjects who were sufficiently fluent in French or English and agreed to participate were included into the PsyCoLaus sub-study and underwent the psychiatric assessment between 2004 and 2008.

Inclusion criteria

```
• >55 years old at baseline

• They signed the agreement of further use of the study data.

• They underwent a baseline dietary assessment at least 3 years
before the cognitive function assessment.
```

Participant Exclusion Criteria

```
• The negation of the above

• Cognitive impairment at baseline MMSE < 24

• Caloric intake outside the normal range of 500 and 4200.
```

In [15]:
```
ch_sc <- ch %>% #6733 at ch
        filter(!is.na(MMSE_b)) %>% #n=1995
       filter(MMSE_b > 23)%>%
        filter(age_b > 55)%>%#<55 years old at baseline (n=0)
        filter((sex == 1 & totalcal_b > 799 & totalcal_b < 4201)|(sex == 0 & total

#Assessment of missing baseline variables
table(is.na(ch_sc$age_b)) # 0 missing
table(is.na(ch_sc$sex)) # 0 missing
table(is.na(ch_sc$edu)) # 0 missing
table(is.na(ch_sc$occ_b)) # 36 missing
table(is.na(ch_sc$bmi_b)) # 18 missing
table(is.na(ch_sc$sm_b)) # 5 missing
table(is.na(ch_sc$depre_b))  # 138 missing
table(is.na(ch_sc$totalcal_b)) # # 0 missing
table(is.na(ch_sc$famincome_b)) # 396 missing!!!!!!!!
table(is.na(ch_sc$diab_b)) # 0 missing
table(is.na(ch_sc$cvevent_b)) # 5 missing
table(is.na(ch_sc$HTA_b)) # 2 missing
table(is.na(ch_sc$alc_b)) # 0 missing
table(is.na(ch_sc$pa_b)) # 188 missing!!!!!!!
table(is.na(ch_sc$MMSE_b)) # 0 missing
```

```
FALSE
 1746
FALSE
 1746
FALSE
 1746
FALSE  TRUE
 1711    35
FALSE  TRUE
 1727    19
FALSE  TRUE
 1741     5
FALSE  TRUE
 1584   162
FALSE
 1746
FALSE  TRUE
 1126   620
FALSE
 1746
FALSE  TRUE
 1742     4
FALSE  TRUE
 1744     2
FALSE
 1746
FALSE  TRUE
 1527   219
FALSE
 1746
```

In [16]:
```
ch_sc #N= 1536
id_FU2 <- ch_sc %>% filter(futime_F1 < 3)
length(id_FU2 <- c(id_FU2$pt)) #there are 8 people with a follow-up lower than 3 ye
```

A data.frame: 1746

pt	sex	datbirth	datexam	bthpl_dem	ethori_self	edtyp	edlv	mrtsts	sclhlp	⋯	age_b
<int>	<int>	<date>	<date>	<chr>	<chr>	<int>	<int>	<int>	<int>	⋯	<int>
6	0	1943-01-30	2003-07-08	SZ	W	1	12	3	0	⋯	66
7	0	1933-03-26	2003-07-04	EY	W	0	8	0	1	⋯	76
19	1	1948-01-23	2003-08-04	SZ	W	1	11	2	0	⋯	61
20	0	1943-05-26	2003-07-16	SZ	W	3	14	2	0	⋯	67
24	1	1936-09-16	2003-07-14	PD	W	3	50	2	1	⋯	73
25	0	1943-12-28	2003-08-28	SZ	W	1	12	3	0	⋯	66
28	1	1948-04-29	2003-07-24	SZ	W	3	14	0	0	⋯	61
31	0	1948-03-10	2003-07-23	EG	W	2	24	2	0	⋯	61
46	0	1942-11-07	2003-07-31	SZ	W	0	10	0	0	⋯	68
48	1	1947-05-05	2003-08-13	SZ	W	2	16	2	0	⋯	62
51	0	1948-10-16	2003-09-01	SZ	W	1	14	NA	0	⋯	61
55	0	1943-12-26	2003-09-09	FR	W	2	18	0	0	⋯	65
56	1	1939-01-01	2003-08-04	SZ	W	3	12	0	1	⋯	70
68	0	1940-07-10	2003-08-05	SZ	W	0	10	3	0	⋯	69
72	0	1941-06-09	2003-08-08	RM	W	2	18	0	0	⋯	68
74	0	1946-11-15	2003-09-09	SZ	W	0	9	3	0	⋯	63
76	1	1939-05-23	2003-07-31	SZ	W	2	16	0	0	⋯	70
80	1	1944-05-04	2003-07-29	SZ	W	1	14	0	0	⋯	65
84	1	1949-01-14	2003-08-05	SZ	W	1	14	0	0	⋯	60
85	0	1945-11-02	2003-08-05	FR	W	2	20	0	0	⋯	64
87	1	1943-04-30	2003-08-12	SZ	W	3	15	0	0	⋯	66
95	1	1945-10-03	2003-08-20	SZ	W	3	21	2	0	⋯	65

pt	sex	datbirth	datexam	bthpl_dem	ethori_self	edtyp	edlv	mrtsts	sclhlp	⋯	age_b
<int>	<int>	<date>	<date>	<chr>	<chr>	<int>	<int>	<int>	<int>	⋯	<int>
102	0	1943-11-22	2003-08-25	SZ	W	1	15	2	0	⋯	67
103	0	1946-02-04	2003-08-19	SZ	W	3	15	0	0	⋯	63
112	1	1929-11-24	2003-09-02	SZ	W	0	10	0	1	⋯	81
119	0	1945-10-10	2003-08-26	SZ	W	1	12	2	0	⋯	65
121	1	1945-07-17	2003-08-25	FR	W	1	13	0	0	⋯	64
122	1	1949-05-27	2003-08-25	SZ	W	4	16	0	0	⋯	62
128	0	1933-08-27	2003-08-22	SZ	W	1	12	0	1	⋯	76
130	1	1943-09-06	2003-08-26	CT	X	1	12	2	1	⋯	68
⋮	⋮	⋮	⋮	⋮	⋮	⋮	⋮	⋮	⋮	⋱	⋮
9291	0	1943-03-12	2005-02-22	AN	O	2	11	2	0	⋯	67
9292	0	1939-07-17	2005-02-02	CO	O	0	12	0	1	⋯	71
9298	1	1932-07-11	2005-01-10	AG	B	4	18	2	1	⋯	78
9316	1	1936-12-04	2005-01-31	BN	B	4	18	0	0	⋯	74
9323	1	1944-02-09	2005-02-04	MT	A	3	15	0	0	⋯	68
9327	0	1933-03-24	2005-02-02	LE	A	2	12	3	1	⋯	77
9336	0	1934-08-24	2005-02-18	CL	O	4	9	0	1	⋯	76
9341	0	1946-12-20	2005-03-22	MT	B	0	5	0	0	⋯	64
9344	0	1946-04-09	2005-03-03	RW	B	4	12	0	0	⋯	64
9366	1	1946-08-20	2005-03-31	CI	O	4	25	0	0	⋯	64
9374	1	1943-09-17	2005-04-11	CO	B	4	20	0	0	⋯	67
9394	0	1948-09-30	2005-04-26	PR	O	3	13	0	0	⋯	62
9397	0	1949-11-11	2005-05-04	MY	A	3	17	0	0	⋯	61

pt	sex	datbirth	datexam	bthpl_dem	ethori_self	edtyp	edlv	mrtsts	sclhlp	⋯	age_b
<int>	<int>	<date>	<date>	<chr>	<chr>	<int>	<int>	<int>	<int>	⋯	<int>
9413	0	1947-01-13	2005-05-18	X	O	0	8	0	0	⋯	64
9429	1	1947-11-02	2005-06-06	X	O	3	18	0	0	⋯	63
9440	1	1950-01-27	2005-06-29	AN	O	4	21	2	0	⋯	61
9448	0	1941-04-05	2005-07-25	X	O	2	3	3	0	⋯	69
9488	1	1943-03-14	2005-09-02	AG	O	4	12	2	0	⋯	68
9490	1	1941-05-01	2005-09-13	TY	A	4	12	0	0	⋯	71
9492	0	1946-08-19	2005-09-16	SZ	W	1	10	2	1	⋯	65
9494	0	1950-09-29	2005-09-12	PS	A	4	17	0	0	⋯	60
9520	0	1947-09-01	2005-11-01	TY	A	3	14	0	1	⋯	64
9549	0	1948-06-21	2005-12-06	HT	O	2	18	NA	0	⋯	63
9551	1	1940-10-23	2005-12-06	TY	A	4	17	0	1	⋯	72
9565	0	1941-07-09	2006-01-12	IR	A	4	17	0	1	⋯	70
9569	0	1944-06-18	2006-02-16	TU	B	0	9	2	1	⋯	67
9573	1	1948-11-08	2006-01-04	CI	O	3	14	0	0	⋯	63
9592	0	1943-06-07	2006-01-26	IR	A	1	12	2	1	⋯	68
9604	1	1939-12-20	2006-02-06	CN	A	4	19	0	1	⋯	71
9639	0	1944-09-12	2006-03-17	MT	B	1	10	3	0	⋯	67

In [17]:

```
#Corrections sent by Pedro Marques-Vidal - email 08.09.22

ch_sc_look <- ch_sc %>% dplyr::select(pt, F2MME, F3MME) %>% filter(pt %in% id_FU2)
ch_sc_look

# Put the values of those followed up less than 3 years in the 1st follow up

ch_sc$F2MME[ch_sc$pt == 122] <- 30
ch_sc$F2MME[ch_sc$pt == 171] <- 30
ch_sc$F2MME[ch_sc$pt == 2058] <- 27
ch_sc$F2MME[ch_sc$pt == 1361] <- 27
ch_sc$F2MME[ch_sc$pt == 1734] <- 29
```

```
ch_sc$F2MME[ch_sc$pt == 1751] <- 29
ch_sc$F2MME[ch_sc$pt == 2235] <- 25
ch_sc$F2MME[ch_sc$pt == 5628] <- 29
ch_sc$F2MME[ch_sc$pt == 9604] <- 30
ch_sc$F3MME[ch_sc$pt == 122] <- NA
ch_sc$F3MME[ch_sc$pt == 171] <- NA
ch_sc$F3MME[ch_sc$pt == 2058] <- NA
ch_sc$F3MME[ch_sc$pt == 196] <- NA
ch_sc$F3MME[ch_sc$pt == 214] <- NA
ch_sc$F3MME[ch_sc$pt == 994] <- NA
ch_sc$F3MME[ch_sc$pt == 1477] <- NA
ch_sc$F3MME[ch_sc$pt == 9323] <- NA
ch_sc$F3MME[ch_sc$pt == 1383] <- 27
ch_sc$F3MME[ch_sc$pt == 3455] <- 30

#Check that it is correct
ch_sc_look <- ch_sc %>% dplyr::select(pt, F2MME, F3MME) %>% filter(pt %in% id_FU2)
ch_sc_look

#replace the time & outcome vars to the adequate tp
ch_sc$futime_F1[ch_sc$pt == 122] <- ch_sc$futime_F2
ch_sc$futime_F1[ch_sc$pt == 2058] <- ch_sc$futime_F2
ch_sc$futime_F1[ch_sc$pt == 196] <- ch_sc$futime_F2
ch_sc$futime_F1[ch_sc$pt == 214] <- ch_sc$futime_F2
ch_sc$futime_F1[ch_sc$pt == 994] <- ch_sc$futime_F2
ch_sc$futime_F1[ch_sc$pt == 1477] <- ch_sc$futime_F2
ch_sc$futime_F1[ch_sc$pt == 9323] <- ch_sc$futime_F2
ch_sc$futime_F2[ch_sc$pt == 171] <- NA

ch_sc$futime_F2[ch_sc$pt == 122] <- NA
ch_sc$futime_F2[ch_sc$pt == 171] <- NA
ch_sc$futime_F2[ch_sc$pt == 2058] <- NA
ch_sc$futime_F2[ch_sc$pt == 196] <- NA
ch_sc$futime_F2[ch_sc$pt == 214] <- NA
ch_sc$futime_F2[ch_sc$pt == 994] <- NA
ch_sc$futime_F2[ch_sc$pt == 1477] <- NA
ch_sc$futime_F2[ch_sc$pt == 9323] <- NA
```

A data.frame: 10 × 3

pt	F2MME	F3MME
<int>	<int>	<int>
122	28	30
171	NA	30
196	30	30
214	30	30
815	30	NA
865	30	NA
994	29	29
1477	29	29
2058	29	27
9323	29	29

A data.frame: 10 × 3

pt	F2MME	F3MME
<int>	<dbl>	<dbl>
122	30	NA
171	30	NA
196	30	NA
214	30	NA
815	30	NA
865	30	NA
994	29	NA
1477	29	NA
2058	27	NA
9323	29	NA

3.1 Not recruited for PsyColaus

In [18]:

```
included <- unique(c(ch_sc$pt))
length(included) #1347

total_included <- unique(c(ch$pt))
length(total_included)#6733

excluded <- setdiff(total_included, included)
length(excluded) #5269

ch <- ch %>% mutate(psy = ifelse(pt %in% included, 1, 0))
```

1746

6746

5000

3.2 LTFU

In [19]:

```
ch_baseline <- ch %>% #6733 at ch
        filter(!is.na(MMSE_b))%>%
        filter(age_b > 55)%>%#>55 years old at baseline (n=0)
        filter(MMSE_b > 23)%>%
        filter(futime_F1 > 3 |futime_F2 > 3)%>%#baseline dietary assessment at lea
        filter((sex == 1 & totalcal_b > 799 & totalcal_b < 4201)|(sex == 0 & total
        filter(!is.na(F2MME))

baseline_b <- unique(c(ch_baseline$pt))
length(baseline_b) #1334

excluded <- setdiff(included, baseline_b)
length(excluded) #432

ch_sc <- ch_sc %>% mutate(ltfu = ifelse(pt %in% baseline_b, 1,
                                  ifelse(pt %in% excluded, 0, NA)))
```

1334

412

4. Data descriptives

4.1 Main outcome at each time point

Categorical cognitive measures

In [20]:
```
#CDR
table(ch_sc$CDR_b) #we cannot include participants with MCI at baseline in the surv
table(ch_sc$F2CDR)
table(ch_sc$F3CDR)

#SCD
ch_sc$SCD_b = ch_sc$F1_QPC_A+ch_sc$F1_QPC_B+ch_sc$F1_QPC_1+ch_sc$F1_QPC_2+ch_sc$F1_

ch_sc$F2SCD = ch_sc$F2_QPC_A+ch_sc$F2_QPC_B+ch_sc$F2_QPC_1+ch_sc$F2_QPC_2+ch_sc$F2_

ch_sc$F3SCD = ch_sc$F3_QPC_A+ch_sc$F3_QPC_B+ch_sc$F3_QPC_1+ch_sc$F3_QPC_2+ch_sc$F3_

ch_sc <- mutate(ch_sc, SCD_b_yn = ifelse(SCD_b >= 3 |F1_QPC_5==1|(F1_QPC_A+F1_QPC_
ch_sc <- mutate(ch_sc, F2SCD_yn = ifelse(F2SCD >= 3 |F2_QPC_5==1|(F2_QPC_A+F2_QPC_
ch_sc <- mutate(ch_sc, F3SCD_yn = ifelse(F3SCD >= 3 |F3_QPC_5==1|(F3_QPC_A+F3_QPC_

table(ch_sc$SCD_b_yn)
table(ch_sc$F2SCD_yn)
table(ch_sc$F3SCD_yn)
```

```
0.0 0.5 1.0
509 465   1
0.0 0.5
487 426
0.0 0.5
247 181
  0   1
789 177
  0   1
793 179
  0   1
383  76
```

In [21]:
```
quantile(na.omit(ch_sc$F2verbal), 0.75)
```

75%: 36

Continuous cognitive measures

In [22]:
```
hist(ch_sc$F2memory,breaks=48) #max 48
hist(ch_sc$F2verbal,breaks=58) #max 58
t.test(ch_sc$F2verbal)
hist(ch_sc$F2stroop,breaks=24)#max 24
hist(ch_sc$F2do40,breaks=40)  #max 40
hist(ch_sc$F2CERAD,breaks=11)  #max 11
```

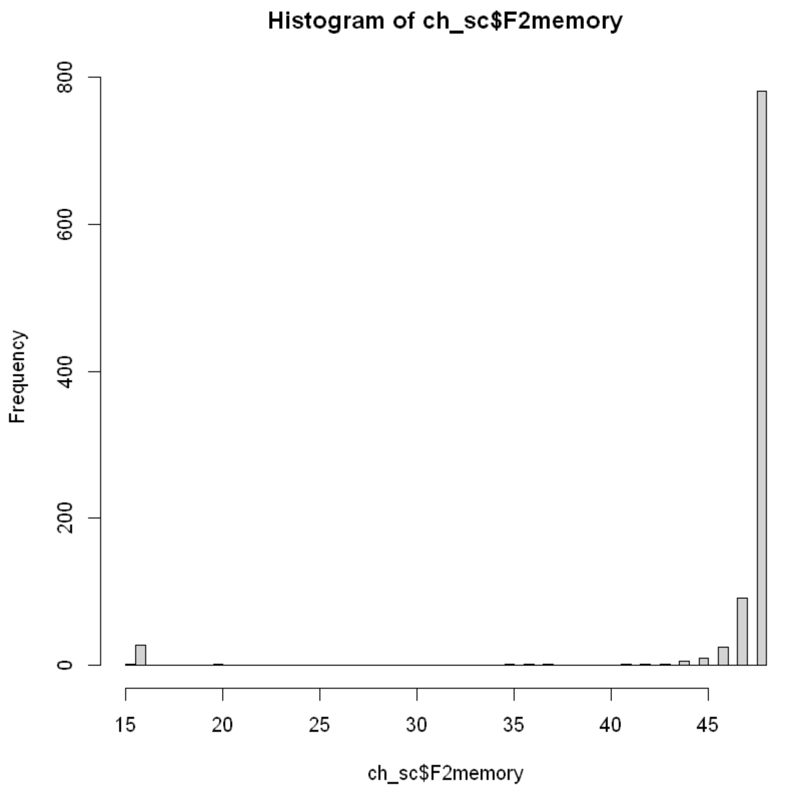


```
        One Sample t-test

data:  ch_sc$F2verbal
t = 108.75, df = 953, p-value < 2.2e-16
alternative hypothesis: true mean is not equal to 0
95 percent confidence interval:
 29.35363 30.43254
sample estimates:
mean of x
 29.89308
```

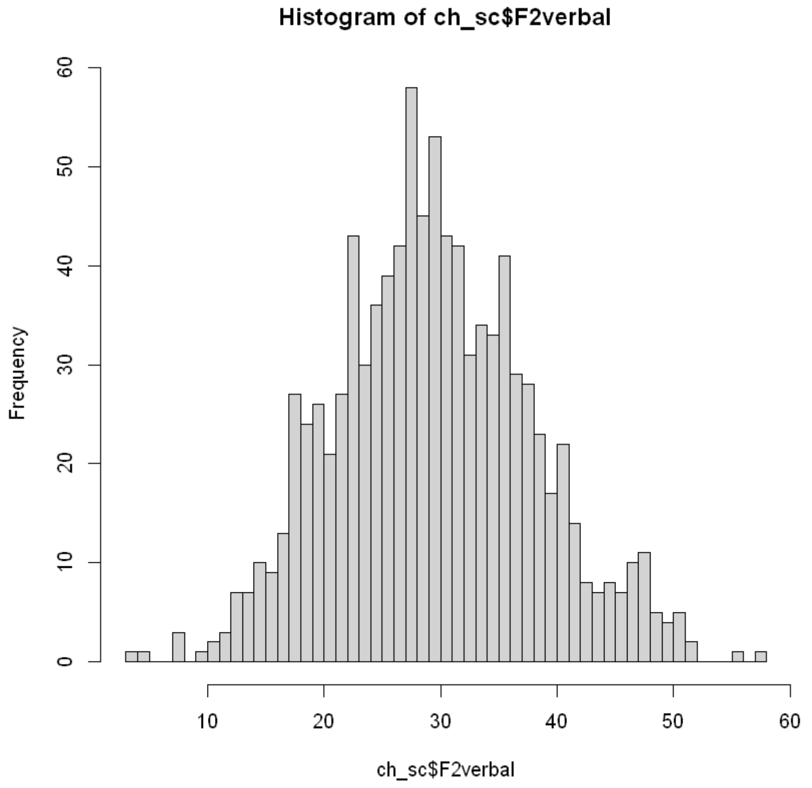

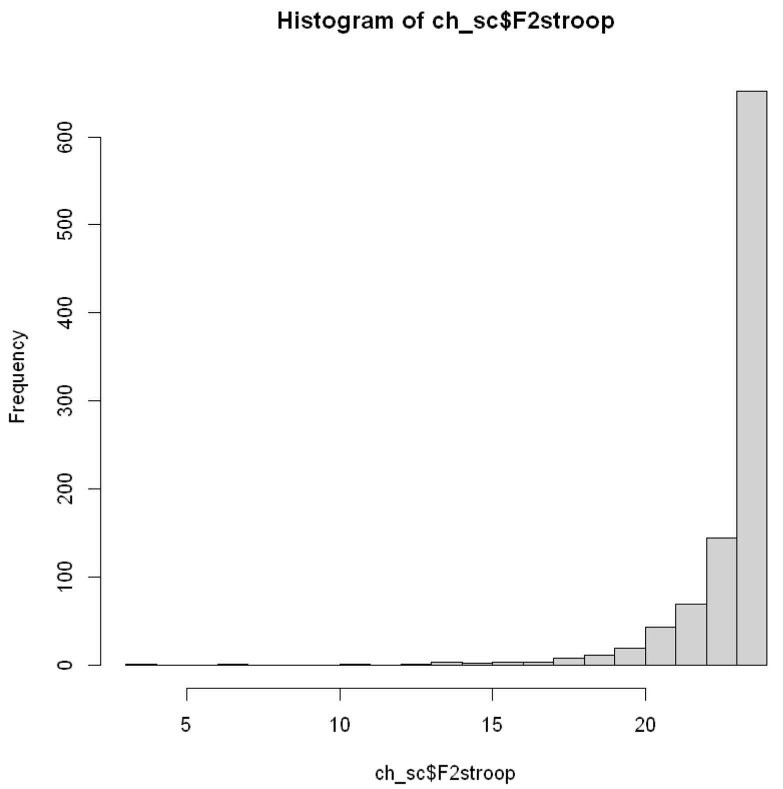
Histogram of ch_sc$F2stroop
Frequency
0
100
200
300
400
500
600
5
10
15
20
ch_sc$F2stroop

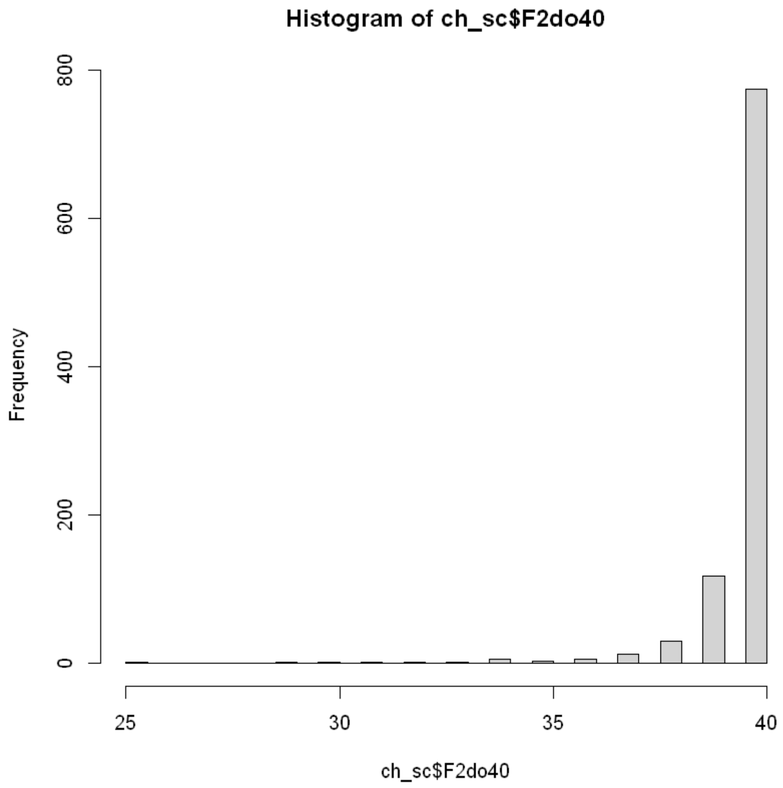
Histogram of ch_sc$F2do40
Frequency
0
200
400
600
800
25
30
35
40
ch_sc$F2do40

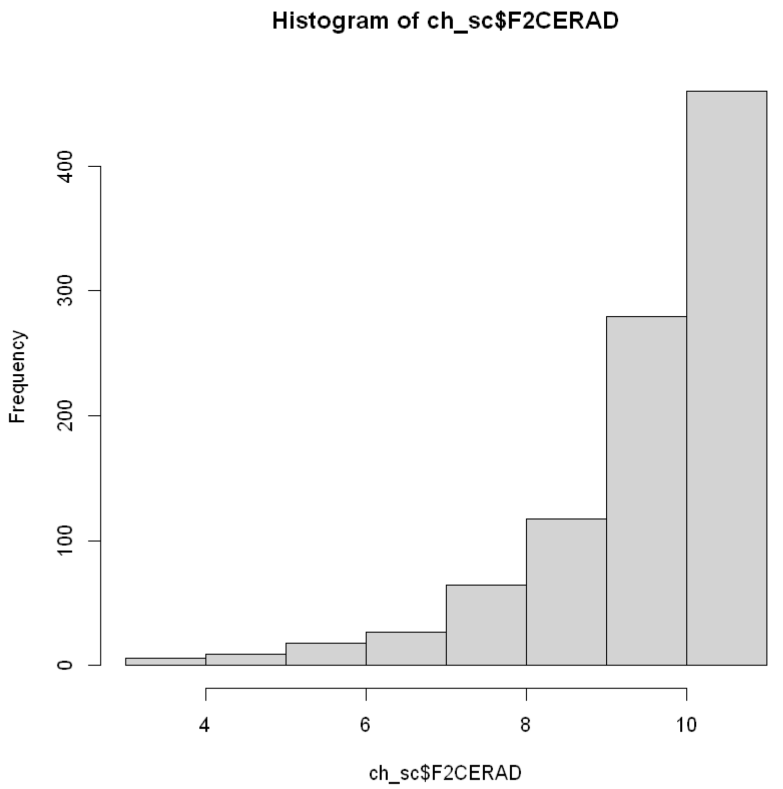

Dicotomization of continuous outcomes

In [23]:
```
quantile(na.omit(ch_sc$F2verbal), 0.75)

ch_sc <- mutate(ch_sc,
                memory_b_cat = ifelse(memory_b<48,1,0),
                verbal_b_cat =ifelse(verbal_b<30,1,0) ,
                stroop_b_cat = ifelse(stroop_b<24,1,0),
                do40_b_cat =ifelse(do40_b<40,1,0),
                CERAD_b_cat =ifelse(CERAD_b<11,1,0),
                F2memory_cat = ifelse(F2memory<48,1,0),
                F2verbal_cat =ifelse(F2verbal<36,1,0) ,
                F2stroop_cat = ifelse(F2stroop<24,1,0),
                F2do40_cat =ifelse(F2do40<40,1,0),
                F2CERAD_cat =ifelse(F2CERAD<11,1,0),
                F3memory_cat = ifelse(F3memory<48,1,0),
                F3verbal_cat =ifelse(F3verbal<36,1,0) ,
                F3stroop_cat = ifelse(F3stroop<24,1,0),
                F3do40_cat =ifelse(F3do40<40,1,0),
                F3CERAD_cat =ifelse(F3CERAD<11,1,0))

table(ch_sc$F2CDR)
table(ch_sc$F2SCD_yn)
table(ch_sc$F2verbal_cat)
table(ch_sc$F2memory_cat)
table(ch_sc$F2do40_cat)
table(ch_sc$F2stroop_cat)
table(ch_sc$F2CERAD_cat)
```

75%: 36

```
0.0 0.5
487 426
  0   1
793 179
  0   1
243 711
```

```
  0   1
781 171
  0   1
774 181
  0   1
652 313
  0   1
460 520
```

4.2 Main exposure at each time point

DAIRY

Continuous dairy intake and subtypes of dairy

In [24]:
```
#grams
ch_sc <- ch_sc %>%
        mutate(total_dairy_b = F1FFQ1amount+F1FFQ2amount+F1FFQ3amount+F1FFQ4amount
                    F1FFQ8amount+F1FFQ52amount+F1FFQ53amount+F1FFQ71amount+F1FFQ68
                    F1FFQ84amount+F1FFQ85amount+F1FFQ86amount+F1FFQ63amount,
                ferm_dairy_b = F1FFQ1amount+F1FFQ2amount+F1FFQ3amount+F1FFQ4amount
                    F1FFQ8amount,
                nonferm_dairy_b = F1FFQ52amount+F1FFQ53amount+F1FFQ71amount+F1FFQ6
                    F1FFQ84amount+F1FFQ85amount+F1FFQ86amount+F1FFQ63amount,
                fullfat_dairy_b = F1FFQ1amount+F1FFQ3amount+F1FFQ5amount+F1FFQ6amo
                    F1FFQ53amount+F1FFQ71amount+F1FFQ68amount+F1FFQ83amount+F1FFQ8
                nonfat_dairy_b = F1FFQ2amount+F1FFQ4amount+F1FFQ82amount+F1FFQ85am
                sugar_dairy_b = F1FFQ3amount+F1FFQ68amount+F1FFQ63amount,
               nonsug_dairy_b = F1FFQ1amount+F1FFQ2amount+F1FFQ4amount+F1FFQ5amoun
                    F1FFQ8amount+F1FFQ52amount+F1FFQ53amount+F1FFQ71amount+F1FFQ82
                    F1FFQ84amount+F1FFQ85amount+F1FFQ86amount,
               F2nonsug_dairy = F2FFQ1amount+F2FFQ2amount+F2FFQ4amount+F2FFQ5amoun
                    F2FFQ8amount+F2FFQ52amount+F2FFQ53amount+F2FFQ71amount+F2FFQ82
                    F2FFQ84amount+F2FFQ85amount+F2FFQ86amount,
                F2total_dairy = F2FFQ1amount+F2FFQ2amount+F2FFQ3amount+F2FFQ4amoun
                    F2FFQ8amount+F2FFQ52amount+F2FFQ53amount+F2FFQ71amount+F2FFQ68
                    F2FFQ84amount+F2FFQ85amount+F2FFQ86amount+F2FFQ63amount,
                F2ferm_dairy = F2FFQ1amount+F2FFQ2amount+F2FFQ3amount+F2FFQ4amount
                    F2FFQ8amount,
                F2nonferm_dairy = F2FFQ52amount+F2FFQ53amount+F2FFQ71amount+F2FFQ6
                    F2FFQ84amount+F2FFQ85amount+F2FFQ86amount+F2FFQ63amount,
                F2fullfat_dairy = F2FFQ1amount+F2FFQ3amount+F2FFQ5amount+F2FFQ6amo
                    F2FFQ53amount+F2FFQ71amount+F2FFQ68amount+F2FFQ83amount+F2FFQ8
                F2nonfat_dairy = F2FFQ2amount+F2FFQ4amount+F2FFQ82amount+F2FFQ85am
                F2sugar_dairy = F2FFQ3amount + F2FFQ68amount+F2FFQ63amount,
                F3total_dairy = F3FFQ1amount+F3FFQ2amount+F3FFQ3amount+F3FFQ4amoun
                    F3FFQ8amount+F3FFQ52amount+F3FFQ53amount+F3FFQ71amount+F3FFQ68
                    F3FFQ84amount+F3FFQ85amount+F3FFQ86amount+F3FFQ63amount,
                F3ferm_dairy = F3FFQ1amount+F3FFQ2amount+F3FFQ3amount+F3FFQ4amount
                    F3FFQ8amount,
                F3nonferm_dairy = F3FFQ52amount+F3FFQ53amount+F3FFQ71amount+F3FFQ6
                    F3FFQ84amount+F3FFQ85amount+F3FFQ86amount+F3FFQ63amount,
                F3fullfat_dairy = F3FFQ1amount+F3FFQ3amount+F3FFQ5amount+F3FFQ6amo
                    F3FFQ53amount+F3FFQ71amount+F3FFQ68amount+F3FFQ83amount+F3FFQ8
                F3nonfat_dairy = F3FFQ2amount+F3FFQ4amount+F3FFQ82amount+F3FFQ85am
                F3sugar_dairy = F3FFQ3amount + F3FFQ68amount+F3FFQ63amount)
```

In [25]:
```
hist(ch_sc$total_dairy_b)
hist(ch_sc$ferm_dairy_b)
hist(ch_sc$nonferm_dairy_b)
hist(ch_sc$fullfat_dairy_b)
```

```
hist(ch_sc$nonfat_dairy_b)
hist(ch_sc$sugar_dairy_b)
```

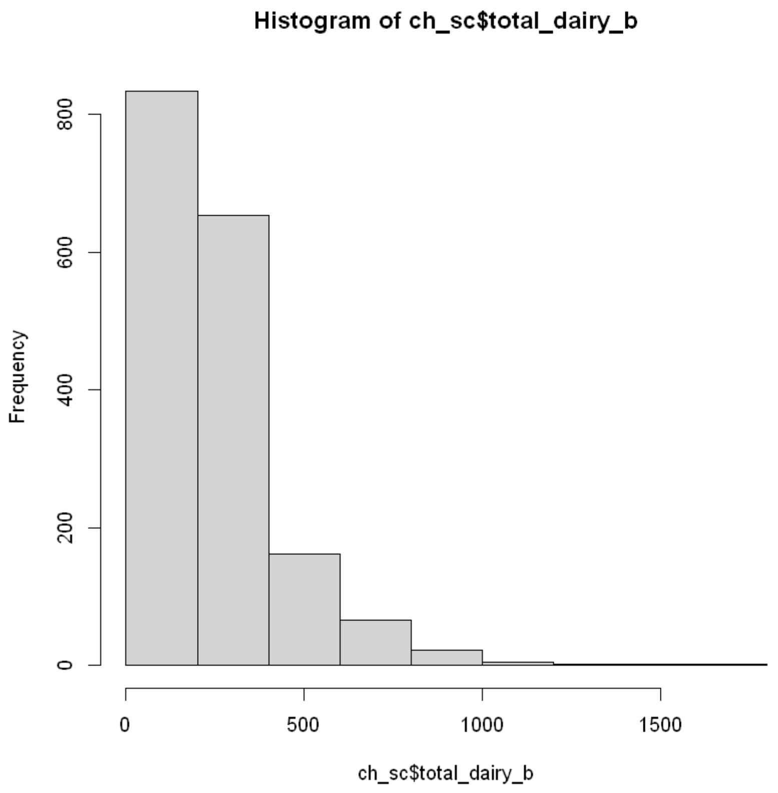

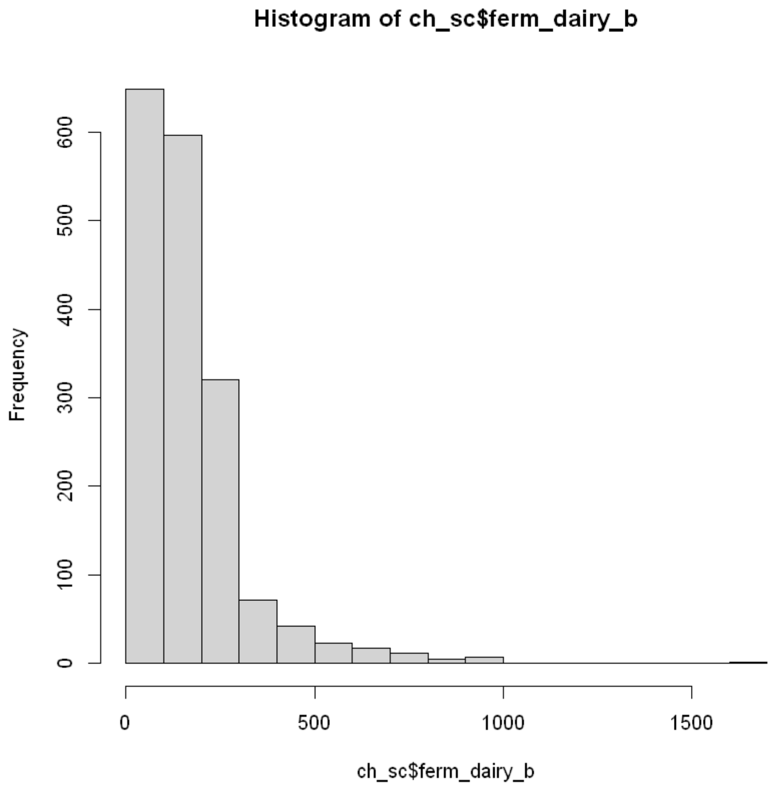

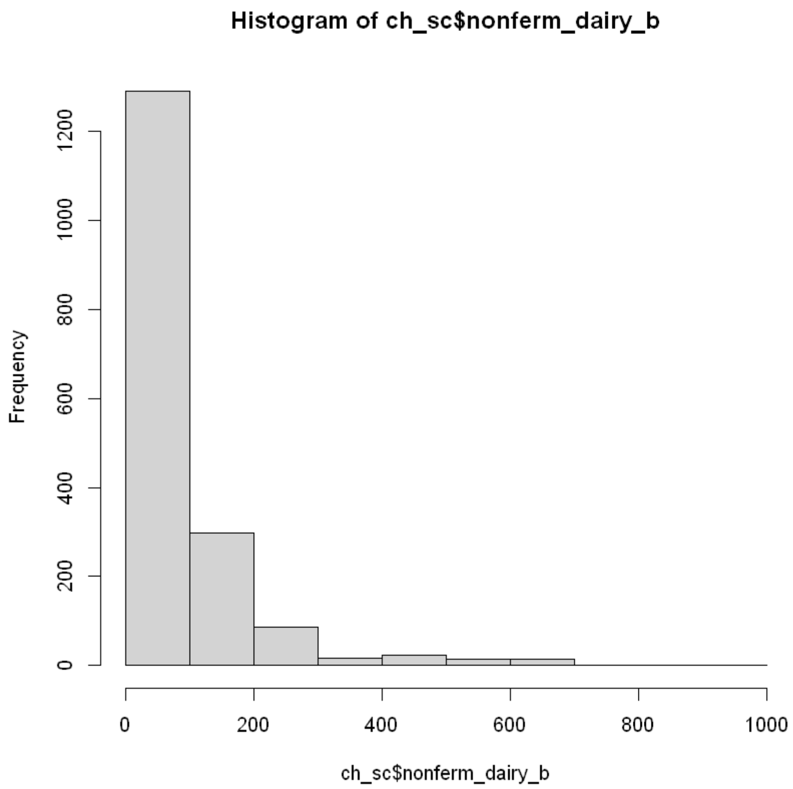
Histogram of ch_sc$nonferm_dairy_b
Frequency
0
200
400
600
800
1000
1200
0
200
400
600
800
1000
ch_sc$nonferm_dairy_b

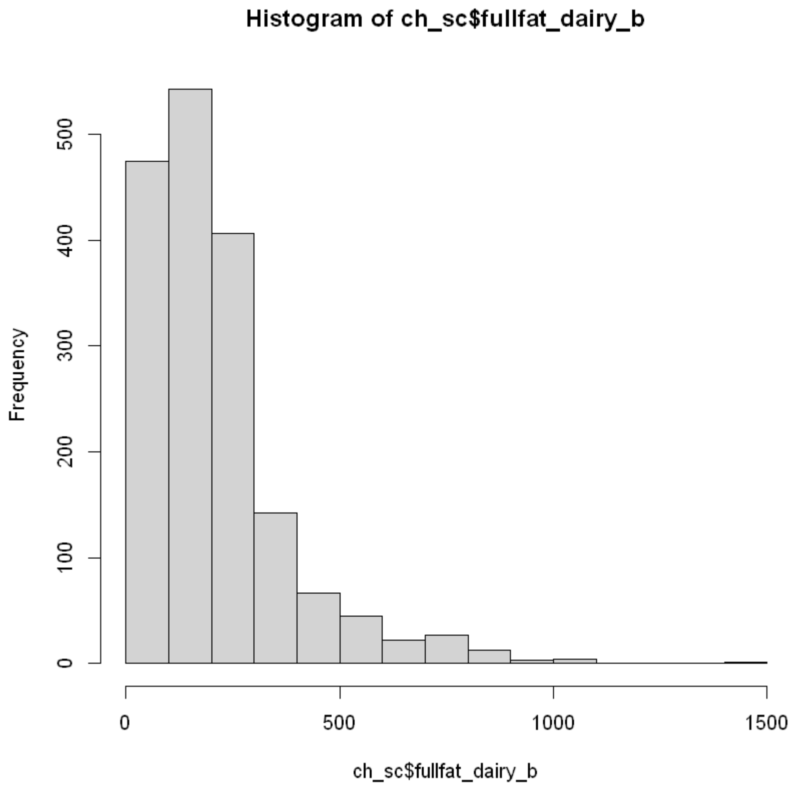
Histogram of ch_sc$fullfat_dairy_b
Frequency
0
100
200
300
400
500
0
500
1000
1500
ch_sc$fullfat_dairy_b

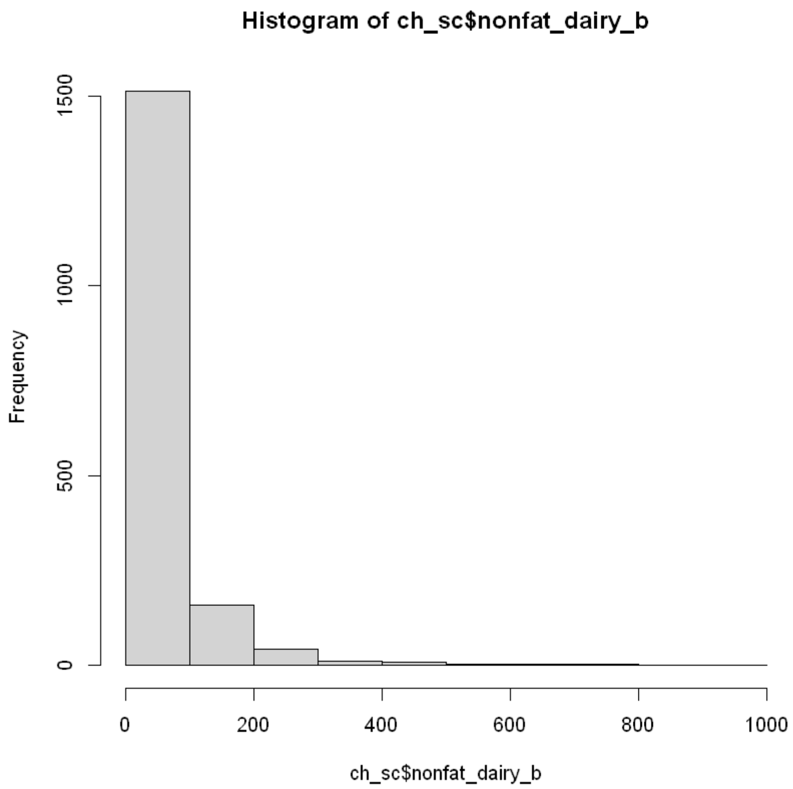

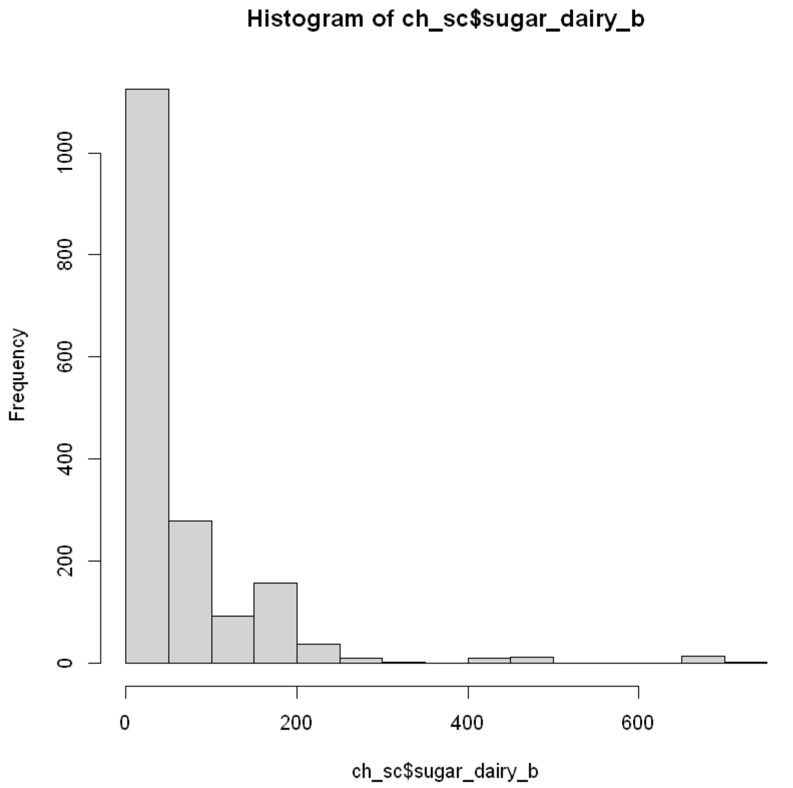

In [26]:

```
ch_sc <- mutate(ch_sc,nonfat_dairy_b_b= ifelse(is.na(nonfat_dairy_b), NA,
                                               ifelse(nonfat_dairy_b == 0, 0, 1)),
                    F2nonfat_dairy_b= ifelse(is.na(F2nonfat_dairy), NA,
                                               ifelse(F2nonfat_dairy == 0, 0, 1)),
                    F3nonfat_dairy_b= ifelse(is.na(F3nonfat_dairy), NA,
                                               ifelse(F3nonfat_dairy == 0, 0, 1)),
                    total_dairy_avg = (total_dairy_b+F2total_dairy)/2,
                    ferm_dairy_avg = (ferm_dairy_b+F2ferm_dairy)/2,
                    nonferm_dairy_avg=(nonferm_dairy_b+F2nonferm_dairy)/2,
                    fullfat_dairy_avg = (fullfat_dairy_b+F2fullfat_dairy)/2,
                    nonfat_dairy_avg = (nonfat_dairy_b+F2nonfat_dairy)/2,
```

```
                        sugar_dairy_avg=(sugar_dairy_b+F2sugar_dairy)/2)

ch_sc <- ch_sc %>%
        mutate(total_dairy_freq_b = F1freqFFQ1+F1freqFFQ2+F1freqFFQ3+F1freqFFQ4+F1
                    F1freqFFQ8+F1freqFFQ52+F1freqFFQ53+F1freqFFQ71+F1freqFFQ68+F1f
                    F1freqFFQ84+F1freqFFQ85+F1freqFFQ86+F1freqFFQ63,
                ferm_dairy_freq_b = F1freqFFQ1+F1freqFFQ2+F1freqFFQ3+F1freqFFQ4+F1
                    F1freqFFQ8,
                nonferm_dairy_freq_b = F1freqFFQ52+F1freqFFQ53+F1freqFFQ71+F1freqF
                    F1freqFFQ84+F1freqFFQ85+F1freqFFQ86+F1freqFFQ63,
                fullfat_dairy_freq_b = F1freqFFQ1+F1freqFFQ3+F1freqFFQ5+F1freqFFQ6
                    F1freqFFQ53+F1freqFFQ71+F1freqFFQ68+F1freqFFQ83+F1freqFFQ84+F1
                nonfat_dairy_freq_b = F1freqFFQ2+F1freqFFQ4+F1freqFFQ82+F1freqFFQ8
                sugar_dairy_freq_b = F1freqFFQ3+F1freqFFQ68+F1freqFFQ63,
                F2total_dairy_freq = F2freqFFQ1+F2freqFFQ2+F2freqFFQ3+F2freqFFQ4+F
                    F2freqFFQ8+F2freqFFQ52+F2freqFFQ53+F2freqFFQ71+F2freqFFQ68+F2f
                    F2freqFFQ84+F2freqFFQ85+F2freqFFQ86+F2freqFFQ63,
                F2ferm_dairy_freq = F2freqFFQ1+F2freqFFQ2+F2freqFFQ3+F2freqFFQ4+F2
                    F2freqFFQ8,
                F2nonferm_dairy_freq = F2freqFFQ52+F2freqFFQ53+F2freqFFQ71+F2freqF
                    F2freqFFQ84+F2freqFFQ85+F2freqFFQ86+F2freqFFQ63,
                F2fullfat_dairy_freq = F2freqFFQ1+F2freqFFQ3+F2freqFFQ5+F2freqFFQ6
                    F2freqFFQ53+F2freqFFQ71+F2freqFFQ68+F2freqFFQ83+F2freqFFQ84+F2
                F2nonfat_dairy_freq = F2freqFFQ2+F2freqFFQ4+F2freqFFQ82+F2freqFFQ8
                F2sugar_dairy_freq = F2freqFFQ3 + F2freqFFQ68+F2freqFFQ63,
                F3total_dairy_freq = F3freqFFQ1+F3freqFFQ2+F3freqFFQ3+F3freqFFQ4+F
                    F3freqFFQ8+F3freqFFQ52+F3freqFFQ53+F3freqFFQ71+F3freqFFQ68+F3f
                    F3freqFFQ84+F3freqFFQ85+F3freqFFQ86+F3freqFFQ63,
                F3ferm_dairy_freq = F3freqFFQ1+F3freqFFQ2+F3freqFFQ3+F3freqFFQ4+F3
                    F3freqFFQ8,
                F3nonferm_dairy_freq = F3freqFFQ52+F3freqFFQ53+F3freqFFQ71+F3freqF
                    F3freqFFQ84+F3freqFFQ85+F3freqFFQ86+F3freqFFQ63,
                F3fullfat_dairy_freq = F3freqFFQ1+F3freqFFQ3+F3freqFFQ5+F3freqFFQ6
                    F3freqFFQ53+F3freqFFQ71+F3freqFFQ68+F3freqFFQ83+F3freqFFQ84+F3
                F3nonfat_dairy_freq = F3freqFFQ2+F3freqFFQ4+F3freqFFQ82+F3freqFFQ8
                F3sugar_dairy_freq = F3freqFFQ3 + F3freqFFQ68+F3freqFFQ63)
```

In [27]:

```
#Calculate number serving/day

ch_sc <- ch_sc %>% mutate(total_dairy_freq_b_serv3 = ifelse(total_dairy_freq_b <=1
                                             ifelse(total_dairy_freq_b < 3, 2,
                                                     ifelse(total_dairy_freq_b >=
                         ferm_dairy_freq_b_serv3 = ifelse(ferm_dairy_freq_b <=1, 1
                                                       ifelse(ferm_dairy_freq_b <
                                                           ifelse(ferm_dairy_freq
                         nonferm_dairy_freq_b_serv3 = ifelse(nonferm_dairy_freq_b
                                             ifelse(nonferm_dairy_freq_b< 3, 2,
                                                    ifelse(nonferm_dairy_freq_b>=
                         fullfat_dairy_freq_b_serv3 = ifelse(fullfat_dairy_freq_b
                                             ifelse(fullfat_dairy_freq_b< 3, 2,
                                                    ifelse(fullfat_dairy_freq_b>=
                         nonfat_dairy_freq_b_serv3 = ifelse(nonfat_dairy_freq_b <=
                                             ifelse(nonfat_dairy_freq_b < 3, 2,
                                                    ifelse(nonfat_dairy_freq_b >=
                         sugar_dairy_freq_b_serv3 = ifelse(sugar_dairy_freq_b <=1,
                                             ifelse(sugar_dairy_freq_b < 3, 2,
                                                    ifelse(sugar_dairy_freq_b >= 3

ch_sc$total_dairy_freq_b_serv3 <- factor(ch_sc$total_dairy_freq_b_serv3, levels =
                               labels = c("Once or less than once per day","More
ch_sc$ferm_dairy_freq_b_serv3 <- factor(ch_sc$ferm_dairy_freq_b_serv3, levels = c(
                               labels = c("Once or less than once per day","More
ch_sc$nonferm_dairy_freq_b_serv3 <- factor(ch_sc$nonferm_dairy_freq_b_serv3, level
```

```
                                labels = c("Once or less than once per day","More 
ch_sc$fullfat_dairy_freq_b_serv3 <- factor(ch_sc$fullfat_dairy_freq_b_serv3, level
                                labels = c("Once or less than once per day","More 
ch_sc$nonfat_dairy_freq_b_serv3 <- factor(ch_sc$nonfat_dairy_freq_b_serv3, levels 
                                labels = c("Once or less than once per day","More 
ch_sc$sugar_dairy_freq_b_serv3 <- factor(ch_sc$sugar_dairy_freq_b_serv3, levels = 
                                labels = c("Once or less than once per day","More 
```

In [28]:

```
# Mean and 95% CI of continuous exposures

mean(na.omit(ch_sc$total_dairy_b))
mean(na.omit(ch_sc$ferm_dairy_b))
mean(na.omit(ch_sc$nonferm_dairy_b))
mean(na.omit(ch_sc$fullfat_dairy_b))
mean(na.omit(ch_sc$nonfat_dairy_b))
mean(na.omit(ch_sc$sugar_dairy_b))

t.test(na.omit(ch_sc$total_dairy_b))$"conf.int"
t.test(na.omit(ch_sc$ferm_dairy_b))$"conf.int"
t.test(na.omit(ch_sc$nonferm_dairy_b))$"conf.int"
t.test(na.omit(ch_sc$fullfat_dairy_b))$"conf.int"
t.test(na.omit(ch_sc$nonfat_dairy_b))$"conf.int"
t.test(na.omit(ch_sc$sugar_dairy_b))$"conf.int"

mean(na.omit(ch_sc$F2total_dairy))
mean(na.omit(ch_sc$F2ferm_dairy))
mean(na.omit(ch_sc$F2nonferm_dairy))
mean(na.omit(ch_sc$F2fullfat_dairy))
mean(na.omit(ch_sc$F2nonfat_dairy))
mean(na.omit(ch_sc$F2sugar_dairy))

mean(na.omit(ch_sc$F3total_dairy))
mean(na.omit(ch_sc$F3ferm_dairy))
mean(na.omit(ch_sc$F3nonferm_dairy))
mean(na.omit(ch_sc$F3fullfat_dairy))
mean(na.omit(ch_sc$F3nonfat_dairy))
mean(na.omit(ch_sc$F3sugar_dairy))
```

246.680168176174

164.546004036082

82.1341641400916

208.087312976375

38.5928551997995

66.1026227605957

237.864060937652 · 255.496275414696

157.629082849351 · 171.462925222814

77.0330948751982 · 87.2352334049851

200.247959416622 · 215.926666536127

34.5315720231644 · 42.6541383764347

61.3821897371313 · 70.82305578406

250.445090966103

165.665648220086

83.1498799634399

214.294216653429

36.6110048020156

68.004752135794

236.845940091824

156.753681395471

78.8512517174936

210.093601199806

27.2583699257982

65.0383786424779

Compare if the means of total dairy consumption change over time

In [29]:
```
ch_sc_assumpt_test1 <- ch_sc %>% mutate(ind_diff = total_dairy_b - F2total_dairy)
ch_sc_assumpt_test2 <- ch_sc %>% mutate(ind_diff = total_dairy_b - F3total_dairy)
ch_sc_assumpt_test3 <- ch_sc %>% mutate(ind_diff = F2total_dairy - F3total_dairy)

mean(na.omit(ch_sc_assumpt_test1$ind_diff)) #Very small individual variation from o
mean(na.omit(ch_sc_assumpt_test2$ind_diff)) #Quite a lot of variation from one time
mean(na.omit(ch_sc_assumpt_test3$ind_diff)) #Very small individual variation from o
```

-1.94772073065388

13.2547127274515

6.56456055647929

OTHER FOOD GROUPS

In [30]:
```
ch_sc <- ch_sc %>%
        mutate(veg_b = F1FFQ30amount+F1FFQ31amount+F1FFQ32amount+F1FFQ33amount+F1FF
                   F1FFQ37amount+F1FFQ38amount+F1FFQ39amount+F1FFQ45amount+F1FFQ50
                fru_b = F1FFQ55amount+F1FFQ56amount+F1FFQ57amount+F1FFQ58amount+F1F
                sea_b = F1FFQ25amount+F1FFQ26amount+F1FFQ27amount+F1FFQ28amount+F1F
                meat_b = F1FFQ14amount+F1FFQ17amount+F1FFQ18amount+F1FFQ23amount+F1
                   F1FFQ19amount+F1FFQ20amount+F1FFQ21amount+F1FFQ22amount,
                eggs_b = F1FFQ49amount,
                grains_b = F1FFQ9amount+F1FFQ10amount+F1FFQ11amount+F1FFQ12amount+F
                   F1FFQ42amount+F1FFQ43amount+F1FFQ44amount+F1FFQ46amount,
                alcohol_b = F1FFQ94amount+F1FFQ95amount+F1FFQ96amount+F1FFQ97amount
                sugary_b = F1FFQ61amount+F1FFQ62amount+F1FFQ64amount+F1FFQ65amount+
                   F1FFQ90amount+F1FFQ91amount+F1FFQ92amount,
                fats_b = F1FFQ73amount+F1FFQ74amount+F1FFQ75amount+F1FFQ51amount+F1
                      F1FFQ47amount,
                F2veg = F2FFQ30amount+F2FFQ31amount+F2FFQ32amount+F2FFQ33amount+F2F
                   F2FFQ37amount+F2FFQ38amount+F2FFQ39amount+F2FFQ45amount+F2FFQ50
                F2fru = F2FFQ55amount+F2FFQ56amount+F2FFQ57amount+F2FFQ58amount+F2F
                F2sea = F2FFQ25amount+F2FFQ26amount+F2FFQ27amount+F2FFQ28amount+F2F
                F2meat = F2FFQ14amount+F2FFQ17amount+F2FFQ18amount+F2FFQ23amount+F2
                   F2FFQ19amount+F2FFQ20amount+F2FFQ21amount+F2FFQ22amount,
                F2eggs = F2FFQ49amount,
                F2grains = F2FFQ9amount+F2FFQ10amount+F2FFQ11amount+F2FFQ12amount+F
                   F2FFQ42amount+F2FFQ43amount+F2FFQ44amount+F2FFQ46amount,
                F2alcohol = F2FFQ94amount+F2FFQ95amount+F2FFQ96amount+F2FFQ97amount
                F2sugary = F2FFQ61amount+F2FFQ62amount+F2FFQ64amount+F2FFQ65amount+
                   F2FFQ90amount+F2FFQ91amount+F2FFQ92amount,
                F2fats = F2FFQ73amount+F2FFQ74amount+F2FFQ75amount+F2FFQ51amount+F2
                      F2FFQ47amount,
                F3veg = F3FFQ30amount+F3FFQ31amount+F3FFQ32amount+F3FFQ33amount+F3F
                   F3FFQ37amount+F3FFQ38amount+F3FFQ39amount+F3FFQ45amount+F3FFQ50
                F3fru = F3FFQ55amount+F3FFQ56amount+F3FFQ57amount+F3FFQ58amount+F3F
                F3sea = F3FFQ25amount+F3FFQ26amount+F3FFQ27amount+F3FFQ28amount+F3F
                F3meat = F3FFQ14amount+F3FFQ17amount+F3FFQ18amount+F3FFQ23amount+F3
                   F3FFQ19amount+F3FFQ20amount+F3FFQ21amount+F3FFQ22amount,
```

```
                    F3eggs = F3FFQ49amount,
                    F3grains = F3FFQ9amount+F3FFQ10amount+F3FFQ11amount+F3FFQ12amount+
                        F3FFQ42amount+F3FFQ43amount+F3FFQ44amount+F3FFQ46amount,
                    F3alcohol = F3FFQ94amount+F3FFQ95amount+F3FFQ96amount+F3FFQ97amoun
                    F3sugary = F3FFQ61amount+F3FFQ62amount+F3FFQ64amount+F3FFQ65amount
                        F3FFQ90amount+F3FFQ91amount+F3FFQ92amount,
                    F3fats = F3FFQ73amount+F3FFQ74amount+F3FFQ75amount+F3FFQ51amount+F
                           F3FFQ47amount)
```

4.3 Table 1 (in the 1st html file of the code for the addition effects)

5. Missingness (in the 1st html file of the code for the addition effects)

6. IPTW and MSMs

Covariate selection

Find DAG in the 1st html file of the code for the addition effects.

Here, we target the Average Relative Causal effect of substituting orher food groups with dairy (100 g) while maintaing total amount of food constant using **all components models**

Create IPTW for the different substitutions

In [31]:
```
#estimate weights with a linear model for the treatment dose
#and with kerney density estimation for obtaining the generalized propensity score
#see https://en.wikipedia.org/wiki/Kernel_density_estimation
#using the weightit package. Covariate balance tables for dairy are shown in total

#################
###TOTAL DAIRY###
#################
weight.model = weightit(formula("total_dairy_b ~ veg_b+fru_b+sea_b+meat_b+eggs_b+g
                 age_cat+edu+sm_b+HTA_b+depre_b+pa_b+cvevent_b+famincome_b+occ_b+br
                        method = "ps", use.kernel=T)

#check weights
summary(weight.model$weights)

#add weights to data
ch_sc$ipw_totdairy <- weight.model$weights

#################
###FERM DAIRY###
#################
weight.model = weightit(formula("ferm_dairy_b ~ nonferm_dairy_b+veg_b+fru_b+sea_b+
                 age_cat+edu+sm_b+HTA_b+depre_b+pa_b+cvevent_b+famincome_b+diab_b+
                        method = "ps", use.kernel=T)

#check weights
summary(weight.model$weights)

#add weights to data
ch_sc$ipw_fermdairy <- weight.model$weights
```

```
#################
##NONFERM DAIRY##
#################
weight.model = weightit(formula("nonferm_dairy_b ~ ferm_dairy_b+veg_b+fru_b+sea_b+r
                 age_cat+edu+sm_b+HTA_b+depre_b+pa_b+cvevent_b+famincome_b+diab_b+
                        method = "ps", use.kernel=T)

#check weights
summary(weight.model$weights)

#add weights to data
ch_sc$ipw_nonfermdairy <- weight.model$weights

#################
##FULLFAT DAIRY##
#################
weight.model = weightit(formula("fullfat_dairy_b ~ nonfat_dairy_b+veg_b+fru_b+sea_
                 age_cat+edu+sm_b+HTA_b+depre_b+pa_b+cvevent_b+famincome_b+diab_b+
                        method = "ps", use.kernel=T)

#check weights
summary(weight.model$weights)

#add weights to data
ch_sc$ipw_fullfatdairy <- weight.model$weights

#################
###NONFAT DAIRY##
#################
weight.model = weightit(formula("nonfat_dairy_b ~ fullfat_dairy_b+veg_b+fru_b+sea_
                 age_cat+edu+sm_b+HTA_b+depre_b+pa_b+cvevent_b+famincome_b+diab_b+
                        method = "ps", use.kernel=T)

#check weights
summary(weight.model$weights)

#add weights to data
ch_sc$ipw_nonfatdairy <- weight.model$weights

#################
###SUGARY DAIRY##
#################
weight.model = weightit(formula("sugar_dairy_b ~ nonsug_dairy_b+veg_b+fru_b+sea_b+r
                 age_cat+edu+sm_b+HTA_b+depre_b+pa_b+cvevent_b+famincome_b+diab_b+
                        method = "ps", use.kernel=T)

#check weights
summary(weight.model$weights)

#add weights to data
ch_sc$ipw_sugdairy <- weight.model$weights

#################
######MEAT#######
#################

weight.model = weightit(formula("meat_b ~ total_dairy_b+veg_b+fru_b+sea_b+eggs_b+gr
                 age_cat+edu+sm_b+HTA_b+depre_b+pa_b+cvevent_b+famincome_b+occ_b+br
                        method = "ps", use.kernel=T)

#check weights
summary(weight.model$weights)
```

```
#add weights to data
ch_sc$ipw_meat <- weight.model$weights

#covariate balance for weights obtained with linear model for treatment dose
#and kernel density estimation
balance.table.2 = bal.tab(weight.model, r.threshold=0.1)$Balance
balance.table.2

#################
#######FISH######
#################
weight.model = weightit(formula("sea_b ~ veg_b+fru_b+total_dairy_b+meat_b+eggs_b+gr
                 age_cat+edu+sm_b+HTA_b+depre_b+pa_b+cvevent_b+famincome_b+occ_b+br
                        method = "ps", use.kernel=T)

#check weights
summary(weight.model$weights)

#add weights to data
ch_sc$ipw_sea <- weight.model$weights

#covariate balance for weights obtained with linear model for treatment dose
#and kernel density estimation
balance.table.2 = bal.tab(weight.model, r.threshold=0.1)$Balance
balance.table.2

#################
#######EGGS######
#################
weight.model = weightit(formula("eggs_b ~ veg_b+fru_b+sea_b+meat_b+total_dairy_b+gr
                 age_cat+edu+sm_b+HTA_b+depre_b+pa_b+cvevent_b+famincome_b+occ_b+br
                        method = "ps", use.kernel=T)

#check weights
summary(weight.model$weights)

#add weights to data
ch_sc$ipw_eggs <- weight.model$weights

#covariate balance for weights obtained with linear model for treatment dose
#and kernel density estimation
balance.table.2 = bal.tab(weight.model, r.threshold=0.1)$Balance
balance.table.2

#################
####VEGETABLES###
#################
weight.model = weightit(formula("veg_b ~ total_dairy_b+fru_b+sea_b+meat_b+eggs_b+gr
                 age_cat+edu+sm_b+HTA_b+depre_b+pa_b+cvevent_b+famincome_b+occ_b+br
                        method = "ps", use.kernel=T)

#check weights
summary(weight.model$weights)

#add weights to data
ch_sc$ipw_veg <- weight.model$weights

#covariate balance for weights obtained with linear model for treatment dose
#and kernel density estimation
balance.table.2 = bal.tab(weight.model, r.threshold=0.1)$Balance
balance.table.2

#################
#####FRUITS######
```

```
#################
weight.model = weightit(formula("fru_b ~ veg_b+total_dairy_b+sea_b+meat_b+eggs_b+gr
                age_cat+edu+sm_b+HTA_b+depre_b+pa_b+cvevent_b+famincome_b+occ_b+br
                        method = "ps", use.kernel=T)

#check weights
summary(weight.model$weights)

#add weights to data
ch_sc$ipw_fru <- weight.model$weights

#covariate balance for weights obtained with linear model for treatment dose
#and kernel density estimation
balance.table.2 = bal.tab(weight.model, r.threshold=0.1)$Balance
balance.table.2

threshold_99 <- quantile(ch_sc$ipw_totdairy, 0.995)
ch_sc$ipw_totdairy[ch_sc$ipw_totdairy > threshold_99] <- threshold_99

threshold_99 <- quantile(ch_sc$ipw_fermdairy, 0.995)
ch_sc$ipw_fermdairy[ch_sc$ipw_fermdairy > threshold_99] <- threshold_99

threshold_99 <- quantile(ch_sc$ipw_nonfermdairy, 0.995)
ch_sc$ipw_nonfermdairy[ch_sc$ipw_nonfermdairy > threshold_99] <- threshold_99

threshold_99 <- quantile(ch_sc$ipw_fullfatdairy, 0.995)
ch_sc$ipw_fullfatdairy[ch_sc$ipw_fullfatdairy > threshold_99] <- threshold_99

threshold_99 <- quantile(ch_sc$ipw_nonfatdairy, 0.995)
ch_sc$ipw_nonfatdairy[ch_sc$ipw_nonfatdairy > threshold_99] <- threshold_99

threshold_99 <- quantile(ch_sc$ipw_sugdairy, 0.995)
ch_sc$ipw_sugdairy[ch_sc$ipw_sugdairy > threshold_99] <- threshold_99

threshold_99 <- quantile(ch_sc$ipw_meat, 0.995)
ch_sc$ipw_meat[ch_sc$ipw_meat > threshold_99] <- threshold_99

threshold_99 <- quantile(ch_sc$ipw_sea, 0.995)
ch_sc$ipw_sea[ch_sc$ipw_sea > threshold_99] <- threshold_99

threshold_99 <- quantile(ch_sc$ipw_veg, 0.995)
ch_sc$ipw_veg[ch_sc$ipw_veg > threshold_99] <- threshold_99

threshold_99 <- quantile(ch_sc$ipw_fru, 0.995)
ch_sc$ipw_fru[ch_sc$ipw_fru > threshold_99] <- threshold_99

threshold_99 <- quantile(ch_sc$ipw_eggs, 0.995)
ch_sc$ipw_eggs[ch_sc$ipw_eggs > threshold_99] <- threshold_99
```

```
   Min. 1st Qu.  Median    Mean 3rd Qu.    Max. 
 0.2582  0.9084  0.9715  1.1118  1.0996 24.4390 
   Min. 1st Qu.  Median    Mean 3rd Qu.    Max. 
 0.2027  0.8864  0.9794  1.2044  1.1434 47.4543 
   Min. 1st Qu.  Median    Mean 3rd Qu.    Max. 
 0.3445  0.9256  1.1089  1.4266  1.2847 93.1436 
   Min. 1st Qu.  Median    Mean 3rd Qu.    Max. 
 0.2598  0.8952  0.9931  1.1050  1.0799 10.1937 
    Min.  1st Qu.   Median     Mean  3rd Qu.     Max. 
  0.0656   1.1726   2.7203   4.9051   3.4712 942.8417 
     Min.   1st Qu.    Median      Mean   3rd Qu.      Max. 
  0.09371   1.04618   1.26717   1.59129   1.55894 103.97194 
   Min. 1st Qu.  Median    Mean 3rd Qu.    Max. 
 0.1532  0.7162  0.9009  1.1822  1.1251 69.3263 
```

A data.frame: 39 × 4

	Type	Corr.Un	Corr.Adj	R.Threshold
	<chr>	<dbl>	<dbl>	<chr>
**total_dairy_b**	Contin.	NA	-0.0645979476	Balanced, <0.1
**veg_b**	Contin.	NA	-0.0217577756	Balanced, <0.1
**fru_b**	Contin.	NA	0.0003770678	Balanced, <0.1
**sea_b**	Contin.	NA	-0.0217161744	Balanced, <0.1
**eggs_b**	Contin.	NA	-0.2224258393	Not Balanced, >0.1
**grains_b**	Contin.	NA	-0.1238149702	Not Balanced, >0.1
**alcohol_b**	Contin.	NA	0.0085741225	Balanced, <0.1
**sugary_b**	Contin.	NA	-0.0315916144	Balanced, <0.1
**fats_b**	Contin.	NA	-0.0151034729	Balanced, <0.1
**sex**	Binary	NA	0.0151972662	Balanced, <0.1
**age_cat**	Contin.	NA	-0.0712952148	Balanced, <0.1
**edu_Elementary**	Binary	NA	-0.0123139717	Balanced, <0.1
**edu_High school**	Binary	NA	0.0274147535	Balanced, <0.1
**edu_Superior**	Binary	NA	-0.0117355643	Balanced, <0.1
**sm_b_0**	Binary	NA	-0.0060590013	Balanced, <0.1
**sm_b_1**	Binary	NA	-0.0076145726	Balanced, <0.1
**sm_b_2**	Binary	NA	0.0190976949	Balanced, <0.1
**sm_b:<NA>**	Binary	NA	0.0185965250	Balanced, <0.1
**HTA_b**	Binary	NA	-0.0008509772	Balanced, <0.1
**HTA_b:<NA>**	Binary	NA	-0.0029502275	Balanced, <0.1
**depre_b**	Binary	NA	-0.0105021464	Balanced, <0.1
**depre_b:<NA>**	Binary	NA	-0.1032849094	Not Balanced, >0.1
**pa_b_High**	Binary	NA	0.0374320844	Balanced, <0.1
**pa_b_Low**	Binary	NA	-0.0512272105	Balanced, <0.1
**pa_b_Medium**	Binary	NA	0.0225527981	Balanced, <0.1
**pa_b:<NA>**	Binary	NA	-0.0345680872	Balanced, <0.1
**cvevent_b**	Binary	NA	0.0109120492	Balanced, <0.1
**cvevent_b:<NA>**	Binary	NA	-0.0103758220	Balanced, <0.1
**famincome_b_1**	Binary	NA	0.0147919091	Balanced, <0.1
**famincome_b_2**	Binary	NA	-0.0319941761	Balanced, <0.1
**famincome_b_3**	Binary	NA	0.0250629230	Balanced, <0.1
**famincome_b:<NA>**	Binary	NA	-0.0409948384	Balanced, <0.1
**occ_b_1**	Binary	NA	0.0233043661	Balanced, <0.1
**occ_b_2**	Binary	NA	0.0081899081	Balanced, <0.1

	Type	Corr.Un	Corr.Adj	R.Threshold
	<chr>	<dbl>	<dbl>	<chr>
**occ_b_3**	Binary	NA	-0.0483653700	Balanced, <0.1
**occ_b_9**	Binary	NA	0.0180982586	Balanced, <0.1
**occ_b:<NA>**	Binary	NA	0.0200700612	Balanced, <0.1
**bmi_cat**	Contin.	NA	-0.0310173410	Balanced, <0.1
**bmi_cat:<NA>**	Binary	NA	0.0058419697	Balanced, <0.1

```
  Min.  1st Qu.   Median     Mean  3rd Qu.     Max.
0.1032   0.8029   0.9120   1.2869   1.1025 183.0050
```

A data.frame: 39 × 4

	Type	Corr.Un	Corr.Adj	R.Threshold
	<chr>	<dbl>	<dbl>	<chr>
**veg_b**	Contin.	NA	-0.074230603	Balanced, <0.1
**fru_b**	Contin.	NA	-0.087771314	Balanced, <0.1
**total_dairy_b**	Contin.	NA	0.032597115	Balanced, <0.1
**meat_b**	Contin.	NA	-0.873740950	Not Balanced, >0.1
**eggs_b**	Contin.	NA	-0.114632041	Not Balanced, >0.1
**grains_b**	Contin.	NA	0.019466458	Balanced, <0.1
**alcohol_b**	Contin.	NA	0.059832761	Balanced, <0.1
**sugary_b**	Contin.	NA	0.043073493	Balanced, <0.1
**fats_b**	Contin.	NA	0.035311752	Balanced, <0.1
**sex**	Binary	NA	0.075541565	Balanced, <0.1
**age_cat**	Contin.	NA	0.055729702	Balanced, <0.1
**edu_Elementary**	Binary	NA	0.042811291	Balanced, <0.1
**edu_High school**	Binary	NA	0.050123502	Balanced, <0.1
**edu_Superior**	Binary	NA	-0.104634455	Not Balanced, >0.1
**sm_b_0**	Binary	NA	0.079115346	Balanced, <0.1
**sm_b_1**	Binary	NA	0.034485351	Balanced, <0.1
**sm_b_2**	Binary	NA	-0.158491077	Not Balanced, >0.1
**sm_b:<NA>**	Binary	NA	0.009750775	Balanced, <0.1
**HTA_b**	Binary	NA	0.082686836	Balanced, <0.1
**HTA_b:<NA>**	Binary	NA	0.011648921	Balanced, <0.1
**depre_b**	Binary	NA	-0.092746613	Balanced, <0.1
**depre_b:<NA>**	Binary	NA	0.034379918	Balanced, <0.1
**pa_b_High**	Binary	NA	0.052469233	Balanced, <0.1
**pa_b_Low**	Binary	NA	-0.111545598	Not Balanced, >0.1
**pa_b_Medium**	Binary	NA	0.073044876	Balanced, <0.1
**pa_b:<NA>**	Binary	NA	0.033309257	Balanced, <0.1
**cvevent_b**	Binary	NA	0.065445155	Balanced, <0.1
**cvevent_b:<NA>**	Binary	NA	-0.005507576	Balanced, <0.1
**famincome_b_1**	Binary	NA	0.022476006	Balanced, <0.1
**famincome_b_2**	Binary	NA	-0.078319238	Balanced, <0.1
**famincome_b_3**	Binary	NA	0.080625142	Balanced, <0.1
**famincome_b:<NA>**	Binary	NA	0.006146685	Balanced, <0.1
**occ_b_1**	Binary	NA	0.025861624	Balanced, <0.1
**occ_b_2**	Binary	NA	0.025405871	Balanced, <0.1

	Type	Corr.Un	Corr.Adj	R.Threshold
	<chr>	<dbl>	<dbl>	<chr>
**occ_b_3**	Binary	NA	0.012856259	Balanced, <0.1
**occ_b_9**	Binary	NA	-0.039583305	Balanced, <0.1
**occ_b:<NA>**	Binary	NA	0.022442648	Balanced, <0.1
**bmi_cat**	Contin.	NA	-0.046470611	Balanced, <0.1
**bmi_cat:<NA>**	Binary	NA	-0.038746126	Balanced, <0.1

```
   Min. 1st Qu.  Median    Mean 3rd Qu.    Max. 
 0.0921  0.9804  1.2438  1.6000  1.5785 86.4767 
```

A data.frame: 39 × 4

	Type	Corr.Un	Corr.Adj	R.Threshold
	<chr>	<dbl>	<dbl>	<chr>
**veg_b**	Contin.	NA	-0.016605012	Balanced, <0.1
**fru_b**	Contin.	NA	-0.070548129	Balanced, <0.1
**sea_b**	Contin.	NA	-0.021353858	Balanced, <0.1
**meat_b**	Contin.	NA	-0.396953650	Not Balanced, >0.1
**total_dairy_b**	Contin.	NA	0.065245730	Balanced, <0.1
**grains_b**	Contin.	NA	0.040062793	Balanced, <0.1
**alcohol_b**	Contin.	NA	0.007384689	Balanced, <0.1
**sugary_b**	Contin.	NA	0.040420454	Balanced, <0.1
**fats_b**	Contin.	NA	-0.060874655	Balanced, <0.1
**sex**	Binary	NA	0.035500205	Balanced, <0.1
**age_cat**	Contin.	NA	0.004168584	Balanced, <0.1
**edu_Elementary**	Binary	NA	0.032850180	Balanced, <0.1
**edu_High school**	Binary	NA	0.009572757	Balanced, <0.1
**edu_Superior**	Binary	NA	-0.051400381	Balanced, <0.1
**sm_b_0**	Binary	NA	0.024713391	Balanced, <0.1
**sm_b_1**	Binary	NA	0.018874390	Balanced, <0.1
**sm_b_2**	Binary	NA	-0.060845916	Balanced, <0.1
**sm_b:<NA>**	Binary	NA	0.018509714	Balanced, <0.1
**HTA_b**	Binary	NA	0.064012326	Balanced, <0.1
**HTA_b:<NA>**	Binary	NA	-0.006006178	Balanced, <0.1
**depre_b**	Binary	NA	0.127362486	Not Balanced, >0.1
**depre_b:<NA>**	Binary	NA	0.046322900	Balanced, <0.1
**pa_b_High**	Binary	NA	-0.002266606	Balanced, <0.1
**pa_b_Low**	Binary	NA	-0.064491006	Balanced, <0.1
**pa_b_Medium**	Binary	NA	0.069106469	Balanced, <0.1
**pa_b:<NA>**	Binary	NA	-0.031124733	Balanced, <0.1
**cvevent_b**	Binary	NA	0.082593717	Balanced, <0.1
**cvevent_b:<NA>**	Binary	NA	-0.024721083	Balanced, <0.1
**famincome_b_1**	Binary	NA	0.058804705	Balanced, <0.1
**famincome_b_2**	Binary	NA	-0.072609666	Balanced, <0.1
**famincome_b_3**	Binary	NA	0.021465436	Balanced, <0.1
**famincome_b:<NA>**	Binary	NA	0.007833164	Balanced, <0.1
**occ_b_1**	Binary	NA	0.040718208	Balanced, <0.1
**occ_b_2**	Binary	NA	0.001821063	Balanced, <0.1

	Type	Corr.Un	Corr.Adj	R.Threshold
	<chr>	<dbl>	<dbl>	<chr>
**occ_b_3**	Binary	NA	0.007599743	Balanced, <0.1
**occ_b_9**	Binary	NA	-0.026416907	Balanced, <0.1
**occ_b:<NA>**	Binary	NA	-0.002498949	Balanced, <0.1
**bmi_cat**	Contin.	NA	-0.069094005	Balanced, <0.1
**bmi_cat:<NA>**	Binary	NA	0.007753757	Balanced, <0.1

```
    Min.   1st Qu.    Median      Mean   3rd Qu.      Max.
 0.09365   0.74920   0.91683   1.55869   1.13384 254.12074
```

A data.frame: 39 × 4

	Type	Corr.Un	Corr.Adj	R.Threshold
	<chr>	<dbl>	<dbl>	<chr>
**total_dairy_b**	Contin.	NA	-0.2420720295	Not Balanced, >0.1
**fru_b**	Contin.	NA	-0.1189337118	Not Balanced, >0.1
**sea_b**	Contin.	NA	-0.3828003667	Not Balanced, >0.1
**meat_b**	Contin.	NA	-0.0046455994	Balanced, <0.1
**eggs_b**	Contin.	NA	-0.2826397588	Not Balanced, >0.1
**grains_b**	Contin.	NA	-0.1735364128	Not Balanced, >0.1
**alcohol_b**	Contin.	NA	0.0274489165	Balanced, <0.1
**sugary_b**	Contin.	NA	-0.2356860344	Not Balanced, >0.1
**fats_b**	Contin.	NA	-0.0815733337	Balanced, <0.1
**sex**	Binary	NA	-0.0534075453	Balanced, <0.1
**age_cat**	Contin.	NA	-0.0312530172	Balanced, <0.1
**edu_Elementary**	Binary	NA	-0.0348856366	Balanced, <0.1
**edu_High school**	Binary	NA	0.0581956522	Balanced, <0.1
**edu_Superior**	Binary	NA	-0.0137763121	Balanced, <0.1
**sm_b_0**	Binary	NA	-0.0351832332	Balanced, <0.1
**sm_b_1**	Binary	NA	0.0156721257	Balanced, <0.1
**sm_b_2**	Binary	NA	0.0270909241	Balanced, <0.1
**sm_b:<NA>**	Binary	NA	-0.0005258657	Balanced, <0.1
**HTA_b**	Binary	NA	-0.1060355009	Not Balanced, >0.1
**HTA_b:<NA>**	Binary	NA	-0.0019832878	Balanced, <0.1
**depre_b**	Binary	NA	-0.2592824604	Not Balanced, >0.1
**depre_b:<NA>**	Binary	NA	0.0426645832	Balanced, <0.1
**pa_b_High**	Binary	NA	0.0569618318	Balanced, <0.1
**pa_b_Low**	Binary	NA	-0.0403891069	Balanced, <0.1
**pa_b_Medium**	Binary	NA	-0.0048459538	Balanced, <0.1
**pa_b:<NA>**	Binary	NA	0.0493674724	Balanced, <0.1
**cvevent_b**	Binary	NA	-0.0728127072	Balanced, <0.1
**cvevent_b:<NA>**	Binary	NA	-0.0122260326	Balanced, <0.1
**famincome_b_1**	Binary	NA	0.0243931791	Balanced, <0.1
**famincome_b_2**	Binary	NA	-0.0346076033	Balanced, <0.1
**famincome_b_3**	Binary	NA	0.0153316973	Balanced, <0.1
**famincome_b:<NA>**	Binary	NA	-0.1516217107	Not Balanced, >0.1
**occ_b_1**	Binary	NA	0.0300034860	Balanced, <0.1
**occ_b_2**	Binary	NA	0.0047880361	Balanced, <0.1

	Type	Corr.Un	Corr.Adj	R.Threshold
	<chr>	<dbl>	<dbl>	<chr>
**occ_b_3**	Binary	NA	-0.0613143360	Balanced, <0.1
**occ_b_9**	Binary	NA	0.0266402153	Balanced, <0.1
**occ_b:<NA>**	Binary	NA	0.0234584294	Balanced, <0.1
**bmi_cat**	Contin.	NA	0.0510379141	Balanced, <0.1
**bmi_cat:<NA>**	Binary	NA	0.0200793174	Balanced, <0.1

```
    Min.  1st Qu.   Median     Mean  3rd Qu.     Max. 
  0.1203   0.7885   0.9145   1.2946   1.1513 168.9782 
```

A data.frame: 39 × 4

	Type	Corr.Un	Corr.Adj	R.Threshold
	<chr>	<dbl>	<dbl>	<chr>
**veg_b**	Contin.	NA	-0.311937774	Not Balanced, >0.1
**total_dairy_b**	Contin.	NA	-0.153014622	Not Balanced, >0.1
**sea_b**	Contin.	NA	-0.237355913	Not Balanced, >0.1
**meat_b**	Contin.	NA	-0.051951963	Balanced, <0.1
**eggs_b**	Contin.	NA	-0.037023296	Balanced, <0.1
**grains_b**	Contin.	NA	-0.022047334	Balanced, <0.1
**alcohol_b**	Contin.	NA	0.001066092	Balanced, <0.1
**sugary_b**	Contin.	NA	-0.024115652	Balanced, <0.1
**fats_b**	Contin.	NA	0.045287914	Balanced, <0.1
**sex**	Binary	NA	0.021907203	Balanced, <0.1
**age_cat**	Contin.	NA	0.028734739	Balanced, <0.1
**edu_Elementary**	Binary	NA	-0.017223273	Balanced, <0.1
**edu_High school**	Binary	NA	-0.002581729	Balanced, <0.1
**edu_Superior**	Binary	NA	0.024511856	Balanced, <0.1
**sm_b_0**	Binary	NA	-0.053204619	Balanced, <0.1
**sm_b_1**	Binary	NA	0.005325663	Balanced, <0.1
**sm_b_2**	Binary	NA	0.066679052	Balanced, <0.1
**sm_b:<NA>**	Binary	NA	-0.003581435	Balanced, <0.1
**HTA_b**	Binary	NA	0.053009326	Balanced, <0.1
**HTA_b:<NA>**	Binary	NA	-0.014528011	Balanced, <0.1
**depre_b**	Binary	NA	-0.189322727	Not Balanced, >0.1
**depre_b:<NA>**	Binary	NA	0.017085089	Balanced, <0.1
**pa_b_High**	Binary	NA	0.045072445	Balanced, <0.1
**pa_b_Low**	Binary	NA	-0.104230162	Not Balanced, >0.1
**pa_b_Medium**	Binary	NA	0.071515244	Balanced, <0.1
**pa_b:<NA>**	Binary	NA	0.017007737	Balanced, <0.1
**cvevent_b**	Binary	NA	0.047370404	Balanced, <0.1
**cvevent_b:<NA>**	Binary	NA	-0.015515487	Balanced, <0.1
**famincome_b_1**	Binary	NA	0.046457394	Balanced, <0.1
**famincome_b_2**	Binary	NA	-0.012416965	Balanced, <0.1
**famincome_b_3**	Binary	NA	-0.047413532	Balanced, <0.1
**famincome_b:<NA>**	Binary	NA	-0.146206362	Not Balanced, >0.1
**occ_b_1**	Binary	NA	0.015721293	Balanced, <0.1
**occ_b_2**	Binary	NA	0.008272074	Balanced, <0.1

	Type	Corr.Un	Corr.Adj	R.Threshold
	<chr>	<dbl>	<dbl>	<chr>
**occ_b_3**	Binary	NA	-0.024207716	Balanced, <0.1
**occ_b_9**	Binary	NA	0.004180053	Balanced, <0.1
**occ_b:<NA>**	Binary	NA	0.005159787	Balanced, <0.1
**bmi_cat**	Contin.	NA	-0.060836032	Balanced, <0.1
**bmi_cat:<NA>**	Binary	NA	0.016287878	Balanced, <0.1

IPFollow-up Weights

In [68]:

```
weight.model = weightit(formula("ltfu ~ age_cat + sex +occ_b +bmi_cat + sm_b+ cveve
                        method = "ps", use.kernel=T)

#check weights
summary(weight.model$weights)

#add weights to data
ch_sc$ipcw <- weight.model$weights

#covariate balance for weights obtained with linear model for treatment dose
#and kernel density estimation
balance.table.2 = bal.tab(weight.model, r.threshold=0.1)$Balance
balance.table.2
```

```
   Min. 1st Qu.  Median    Mean 3rd Qu.    Max. 
  1.000   1.186   1.323   2.002   1.837  10.215 
```

A data.frame: 19 × 3

	Type	Diff.Un	Diff.Adj
	<chr>	<dbl>	<dbl>
**prop.score**	Distance	NA	-0.0016961501
**age_cat**	Contin.	NA	0.0105005591
**sex**	Binary	NA	0.0060064694
**occ_b_1**	Binary	NA	0.0015017208
**occ_b_2**	Binary	NA	-0.0012827358
**occ_b_3**	Binary	NA	0.0022397483
**occ_b_9**	Binary	NA	-0.0024587333
**occ_b:<NA>**	Binary	NA	0.0003200799
**bmi_cat**	Contin.	NA	-0.0027748853
**bmi_cat:<NA>**	Binary	NA	0.0013921209
**sm_b_0**	Binary	NA	0.0018245267
**sm_b_1**	Binary	NA	0.0036786160
**sm_b_2**	Binary	NA	-0.0055031427
**sm_b:<NA>**	Binary	NA	-0.0001238209
**cvevent_b**	Binary	NA	0.0085639565
**cvevent_b:<NA>**	Binary	NA	0.0001081469
**HTA_b**	Binary	NA	-0.0107818136
**HTA_b:<NA>**	Binary	NA	0.0011462133
**diab_b**	Binary	NA	0.0030457235

Products of the weights

In [69]:
```
ch_sc <- dplyr::mutate(ch_sc,
                   ipw_totaldairy = ipw_totdairy * ipcw,
                   ipw_fermdairy = ipw_fermdairy * ipcw,
                   ipw_nonfermdairy = ipw_nonfermdairy * ipcw,
                   ipw_fullfatdairy= ipw_fullfatdairy * ipcw,
                   ipw_nonfatdairy= ipw_nonfatdairy * ipcw,
                   ipw_sugdairy= ipw_sugdairy * ipcw,
                   ipw_meat = ipw_meat * ipcw,
                   ipw_sea= ipw_sea * ipcw,
                   ipw_veg= ipw_veg * ipcw,
                   ipw_fru= ipw_fru * ipcw,
                   ipw_eggs= ipw_eggs * ipcw)
```

Extracting ATEs

CDR

In [70]:
```
ch_sc$F2CDR <- as.numeric(ifelse(ch_sc$F2CDR == "0.0", 0,
                     ifelse(ch_sc$F2CDR == "0.5", 1,NA)))
```

```
cdr <- ch_sc %>% filter(!is.na(F2CDR))
```

MSM for CDR

In [71]:
```
msm.totdairy <- geeglm(F2CDR ~ total_dairy_b, data=cdr, weights=ipw_totdairy,id=pt
                       corstr="independence")
SEtotal <- coef(summary(msm.totdairy))[,2]

msm.fermdairy <- geeglm(F2CDR ~ ferm_dairy_b, data=cdr, weights=ipw_fermdairy, id=
                 corstr="independence")
SEferm <- coef(summary(msm.fermdairy))[,2]

msm.nonfermdairy <- geeglm(F2CDR ~ nonferm_dairy_b, data=cdr, weights=ipw_nonfermd
                 corstr="independence")
SEnonferm <- coef(summary(msm.nonfermdairy))[,2]

msm.ffdairy <- geeglm(F2CDR ~ fullfat_dairy_b, data=cdr, weights=ipw_fullfatdairy,
                 corstr="independence")
SEff <- coef(summary(msm.ffdairy))[,2]

msm.nfdairy <- geeglm(F2CDR ~ nonfat_dairy_b, data=cdr, weights=ipw_nonfatdairy, i
                 corstr="independence")
SEnf <- coef(summary(msm.nfdairy))[,2]

msm.sugdairy <- geeglm(F2CDR ~ sugar_dairy_b, data=cdr, weights=ipw_sugdairy, id=p
                 corstr="independence")
SEsug <- coef(summary(msm.sugdairy))[,2]

msm.meat <- geeglm(F2CDR ~ meat_b, data=cdr, weights=ipw_meat, id=pt,
                 corstr="independence")
SEmeat <- coef(summary(msm.meat))[,2]

msm.sea <- geeglm(F2CDR ~ sea_b, data=cdr, weights=ipw_sea, id=pt,
                 corstr="independence")
SEsea <- coef(summary(msm.sea))[,2]


msm.veg <- geeglm(F2CDR ~ veg_b, data=cdr, weights=ipw_veg, id=pt,
                 corstr="independence")
SEveg <- coef(summary(msm.veg))[,2]


msm.fru <- geeglm(F2CDR ~ fru_b, data=cdr, weights=ipw_fru, id=pt,
                 corstr="independence")
SEfru <- coef(summary(msm.fru))[,2]


msm.eggs <- geeglm(F2CDR ~ eggs_b, data=cdr, weights=ipw_eggs, id=pt,
                 corstr="independence")
SEeggs <- coef(summary(msm.eggs))[,2]
```

In [72]:
```
a <- ((coef(msm.totdairy) - coef(msm.meat))*100)[2]
b <- ((coef(msm.totdairy) - coef(msm.meat) - qnorm(0.975) * sqrt(SEtotal^2 + SEmea
c <- ((coef(msm.totdairy) - coef(msm.meat) + qnorm(0.975) * sqrt(SEtotal^2 + SEmea

d <- ((coef(msm.totdairy) - coef(msm.sea))*100)[2]
e <- ((coef(msm.totdairy) - coef(msm.sea) - qnorm(0.975) * sqrt(SEtotal^2 + SEsea^
f <- ((coef(msm.totdairy) - coef(msm.sea) + qnorm(0.975) * sqrt(SEtotal^2 + SEsea^

g <- ((coef(msm.totdairy) - coef(msm.eggs))*100)[2]
h <- ((coef(msm.totdairy) - coef(msm.eggs) - qnorm(0.975) * sqrt(SEtotal^2 + SEegg
i <- ((coef(msm.totdairy) - coef(msm.eggs) + qnorm(0.975) * sqrt(SEtotal^2 + SEegg
```

```
j <- ((coef(msm.totdairy) - coef(msm.veg))*100)[2]
k <- ((coef(msm.totdairy) - coef(msm.veg) - qnorm(0.975) * sqrt(SEtotal^2 + SEveg^
l <- ((coef(msm.totdairy) - coef(msm.veg) + qnorm(0.975) * sqrt(SEtotal^2 + SEveg^

m <- ((coef(msm.totdairy) - coef(msm.fru))*100)[2]
n <- ((coef(msm.totdairy) - coef(msm.fru) - qnorm(0.975) * sqrt(SEtotal^2 + SEfru^
o <- ((coef(msm.totdairy) - coef(msm.fru) + qnorm(0.975) * sqrt(SEtotal^2 + SEfru^

cdrtotal <- c(paste(round(a,3)*100, "% (", round(b,3)*100,"% to ", round(c,3)*100,
              paste(round(d,3)*100, "% (", round(e,3)*100,"% to ", round(f,3)*100,
              paste(round(g,3)*100, "% (", round(h,3)*100,"% to ", round(i,3)*100,
              paste(round(j,3)*100, "% (", round(k,3)*100,"% to ", round(l,3)*100,
              paste(round(m,3)*100, "% (", round(n,3)*100,"% to ", round(o,3)*100,

a <- ((coef(msm.fermdairy) - coef(msm.meat))*100)[2]
b <- ((coef(msm.fermdairy) - coef(msm.meat) - qnorm(0.975) * sqrt(SEferm^2 + SEmea
c <- ((coef(msm.fermdairy) - coef(msm.meat) + qnorm(0.975) * sqrt(SEferm^2 + SEmea

d <- ((coef(msm.fermdairy) - coef(msm.sea))*100)[2]
e <- ((coef(msm.fermdairy) - coef(msm.sea) - qnorm(0.975) * sqrt(SEferm^2 + SEsea^
f <- ((coef(msm.fermdairy) - coef(msm.sea) + qnorm(0.975) * sqrt(SEferm^2 + SEsea^

g <- ((coef(msm.fermdairy) - coef(msm.eggs))*100)[2]
h <- ((coef(msm.fermdairy) - coef(msm.eggs) - qnorm(0.975) * sqrt(SEferm^2 + SEegg
i <- ((coef(msm.fermdairy) - coef(msm.eggs) + qnorm(0.975) * sqrt(SEferm^2 + SEegg

j <- ((coef(msm.fermdairy) - coef(msm.veg))*100)[2]
k <- ((coef(msm.fermdairy) - coef(msm.veg) - qnorm(0.975) * sqrt(SEferm^2 + SEveg^
l <- ((coef(msm.fermdairy) - coef(msm.veg) + qnorm(0.975) * sqrt(SEferm^2 + SEveg^

m <- ((coef(msm.fermdairy) - coef(msm.fru))*100)[2]
n <- ((coef(msm.fermdairy) - coef(msm.fru) - qnorm(0.975) * sqrt(SEferm^2 + SEfru^
o <- ((coef(msm.fermdairy) - coef(msm.fru) + qnorm(0.975) * sqrt(SEferm^2 + SEfru^

cdrferm <- c(paste(round(a,3)*100, "% (", round(b,3)*100,"% to ", round(c,3)*100,"
              paste(round(d,3)*100, "% (", round(e,3)*100,"% to ", round(f,3)*100,
              paste(round(g,3)*100, "% (", round(h,3)*100,"% to ", round(i,3)*100,
              paste(round(j,3)*100, "% (", round(k,3)*100,"% to ", round(l,3)*100,
              paste(round(m,3)*100, "% (", round(n,3)*100,"% to ", round(o,3)*100,

a <- ((coef(msm.nonfermdairy) - coef(msm.meat))*100)[2]
b <- ((coef(msm.nonfermdairy) - coef(msm.meat) - qnorm(0.975) * sqrt(SEnonferm^2 +
c <- ((coef(msm.nonfermdairy) - coef(msm.meat) + qnorm(0.975) * sqrt(SEnonferm^2 +

d <- ((coef(msm.nonfermdairy) - coef(msm.sea))*100)[2]
e <- ((coef(msm.nonfermdairy) - coef(msm.sea) - qnorm(0.975) * sqrt(SEnonferm^2 +
f <- ((coef(msm.nonfermdairy) - coef(msm.sea) + qnorm(0.975) * sqrt(SEnonferm^2 +

g <- ((coef(msm.nonfermdairy) - coef(msm.eggs))*100)[2]
h <- ((coef(msm.nonfermdairy) - coef(msm.eggs) - qnorm(0.975) * sqrt(SEnonferm^2 +
i <- ((coef(msm.nonfermdairy) - coef(msm.eggs) + qnorm(0.975) * sqrt(SEnonferm^2 +

j <- ((coef(msm.nonfermdairy) - coef(msm.veg))*100)[2]
k <- ((coef(msm.nonfermdairy) - coef(msm.veg) - qnorm(0.975) * sqrt(SEnonferm^2 +
l <- ((coef(msm.nonfermdairy) - coef(msm.veg) + qnorm(0.975) * sqrt(SEnonferm^2 +

m <- ((coef(msm.nonfermdairy) - coef(msm.fru))*100)[2]
n <- ((coef(msm.nonfermdairy) - coef(msm.fru) - qnorm(0.975) * sqrt(SEnonferm^2 +
o <- ((coef(msm.nonfermdairy) - coef(msm.fru) + qnorm(0.975) * sqrt(SEnonferm^2 +

cdrnonferm <- c(paste(round(a,3)*100, "% (", round(b,3)*100,"% to ", round(c,3)*10
              paste(round(d,3)*100, "% (", round(e,3)*100,"% to ", round(f,3)*100,
              paste(round(g,3)*100, "% (", round(h,3)*100,"% to ", round(i,3)*100,
```

```
              paste(round(j,3)*100, "% (", round(k,3)*100,"% to ", round(l,3)*100,
              paste(round(m,3)*100, "% (", round(n,3)*100,"% to ", round(o,3)*100,


a <- ((coef(msm.ffdairy) - coef(msm.meat))*100)[2]
b <- ((coef(msm.ffdairy) - coef(msm.meat) - qnorm(0.975) * sqrt(SEff^2 + SEmeat^2)
c <- ((coef(msm.ffdairy) - coef(msm.meat) + qnorm(0.975) * sqrt(SEff^2 + SEmeat^2)

d <- ((coef(msm.ffdairy) - coef(msm.sea))*100)[2]
e <- ((coef(msm.ffdairy) - coef(msm.sea) - qnorm(0.975) * sqrt(SEff^2 + SEsea^2))*
f <- ((coef(msm.ffdairy) - coef(msm.sea) + qnorm(0.975) * sqrt(SEff^2 + SEsea^2))*

g <- ((coef(msm.ffdairy) - coef(msm.eggs))*100)[2]
h <- ((coef(msm.ffdairy) - coef(msm.eggs) - qnorm(0.975) * sqrt(SEff^2 + SEeggs^2)
i <- ((coef(msm.ffdairy) - coef(msm.eggs) + qnorm(0.975) * sqrt(SEff^2 + SEeggs^2)

j <- ((coef(msm.ffdairy) - coef(msm.veg))*100)[2]
k <- ((coef(msm.ffdairy) - coef(msm.veg) - qnorm(0.975) * sqrt(SEff^2 + SEveg^2))*
l <- ((coef(msm.ffdairy) - coef(msm.veg) + qnorm(0.975) * sqrt(SEff^2 + SEveg^2))*

m <- ((coef(msm.ffdairy) - coef(msm.fru))*100)[2]
n <- ((coef(msm.ffdairy) - coef(msm.fru) - qnorm(0.975) * sqrt(SEff^2 + SEfru^2))*
o <- ((coef(msm.ffdairy) - coef(msm.fru) + qnorm(0.975) * sqrt(SEff^2 + SEfru^2))*

cdrfullfat <- c(paste(round(a,3)*100, "% (", round(b,3)*100,"% to ", round(c,3)*10
              paste(round(d,3)*100, "% (", round(e,3)*100,"% to ", round(f,3)*100,
              paste(round(g,3)*100, "% (", round(h,3)*100,"% to ", round(i,3)*100,
              paste(round(j,3)*100, "% (", round(k,3)*100,"% to ", round(l,3)*100,
              paste(round(m,3)*100, "% (", round(n,3)*100,"% to ", round(o,3)*100,


a <- ((coef(msm.nfdairy) - coef(msm.meat))*100)[2]
b <- ((coef(msm.nfdairy) - coef(msm.meat) - qnorm(0.975) * sqrt(SEnf^2 + SEmeat^2)
c <- ((coef(msm.nfdairy) - coef(msm.meat) + qnorm(0.975) * sqrt(SEnf^2 + SEmeat^2)

d <- ((coef(msm.nfdairy) - coef(msm.sea))*100)[2]
e <- ((coef(msm.nfdairy) - coef(msm.sea) - qnorm(0.975) * sqrt(SEnf^2 + SEsea^2))*
f <- ((coef(msm.nfdairy) - coef(msm.sea) + qnorm(0.975) * sqrt(SEnf^2 + SEsea^2))*

g <- ((coef(msm.nfdairy) - coef(msm.eggs))*100)[2]
h <- ((coef(msm.nfdairy) - coef(msm.eggs) - qnorm(0.975) * sqrt(SEnf^2 + SEeggs^2)
i <- ((coef(msm.nfdairy) - coef(msm.eggs) + qnorm(0.975) * sqrt(SEnf^2 + SEeggs^2)

j <- ((coef(msm.nfdairy) - coef(msm.veg))*100)[2]
k <- ((coef(msm.nfdairy) - coef(msm.veg) - qnorm(0.975) * sqrt(SEnf^2 + SEveg^2))*
l <- ((coef(msm.nfdairy) - coef(msm.veg) + qnorm(0.975) * sqrt(SEnf^2 + SEveg^2))*

m <- ((coef(msm.nfdairy) - coef(msm.fru))*100)[2]
n <- ((coef(msm.nfdairy) - coef(msm.fru) - qnorm(0.975) * sqrt(SEnf^2 + SEfru^2))*
o <- ((coef(msm.nfdairy) - coef(msm.fru) + qnorm(0.975) * sqrt(SEnf^2 + SEfru^2))*

cdrnonfat <- c(paste(round(a,3)*100, "% (", round(b,3)*100,"% to ", round(c,3)*100
              paste(round(d,3)*100, "% (", round(e,3)*100,"% to ", round(f,3)*100,
              paste(round(g,3)*100, "% (", round(h,3)*100,"% to ", round(i,3)*100,
              paste(round(j,3)*100, "% (", round(k,3)*100,"% to ", round(l,3)*100,
              paste(round(m,3)*100, "% (", round(n,3)*100,"% to ", round(o,3)*100,


a <- ((coef(msm.sugdairy) - coef(msm.meat))*100)[2]
b <- ((coef(msm.sugdairy) - coef(msm.meat) - qnorm(0.975) * sqrt(SEsug^2 + SEmeat^
c <- ((coef(msm.sugdairy) - coef(msm.meat) + qnorm(0.975) * sqrt(SEsug^2 + SEmeat^

d <- ((coef(msm.sugdairy) - coef(msm.sea))*100)[2]
e <- ((coef(msm.sugdairy) - coef(msm.sea) - qnorm(0.975) * sqrt(SEsug^2 + SEsea^2)
```

```
f <- ((coef(msm.sugdairy) - coef(msm.sea) + qnorm(0.975) * sqrt(SEsug^2 + SEsea^2)

g <- ((coef(msm.sugdairy) - coef(msm.eggs))*100)[2]
h <- ((coef(msm.sugdairy) - coef(msm.eggs) - qnorm(0.975) * sqrt(SEsug^2 + SEeggs^
i <- ((coef(msm.sugdairy) - coef(msm.eggs) + qnorm(0.975) * sqrt(SEsug^2 + SEeggs^

j <- ((coef(msm.sugdairy) - coef(msm.veg))*100)[2]
k <- ((coef(msm.sugdairy) - coef(msm.veg) - qnorm(0.975) * sqrt(SEsug^2 + SEveg^2)
l <- ((coef(msm.sugdairy) - coef(msm.veg) + qnorm(0.975) * sqrt(SEsug^2 + SEveg^2)

m <- ((coef(msm.sugdairy) - coef(msm.fru))*100)[2]
n <- ((coef(msm.sugdairy) - coef(msm.fru) - qnorm(0.975) * sqrt(SEsug^2 + SEfru^2)
o <- ((coef(msm.sugdairy) - coef(msm.fru) + qnorm(0.975) * sqrt(SEsug^2 + SEfru^2)

cdrsug <- c(paste(round(a,3)*100, "% (", round(b,3)*100,"% to ", round(c,3)*100,"%
              paste(round(d,3)*100, "% (", round(e,3)*100,"% to ", round(f,3)*100,
              paste(round(g,3)*100, "% (", round(h,3)*100,"% to ", round(i,3)*100,
              paste(round(j,3)*100, "% (", round(k,3)*100,"% to ", round(l,3)*100,
              paste(round(m,3)*100, "% (", round(n,3)*100,"% to ", round(o,3)*100,

CDR <- data.frame(cdrtotal,cdrferm, cdrnonferm, cdrfullfat, cdrnonfat, cdrsug)
CDR
```

A data.frame: 5 × 6

cdrtotal	cdrferm	cdrnonferm	cdrfullfat	cdrnonfat	cdrsug
<chr>	<chr>	<chr>	<chr>	<chr>	<chr>
-1.3 % (-9.9 % to 7.3 %)	-1.6 % (-10.4 % to 7.2 %)	-1.7 % (-10.7 % to 7.3 %)	-0.3 % (-8.9 % to 8.4 %)	-8.7 % (-19.5 % to 2.1 %)	2.2 % (-6.8 % to 11.3 %)
0.5 % (-16 % to 17.1 %)	0.2 % (-16.4 % to 16.9 %)	0.1 % (-16.7 % to 16.9 %)	1.6 % (-15 % to 18.2 %)	-6.8 % (-24.6 % to 11 %)	4.1 % (-12.7 % to 20.9 %)
10.3 % (-17.9 % to 38.5 %)	10 % (-18.2 % to 38.3 %)	9.9 % (-18.4 % to 38.2 %)	11.4 % (-16.8 % to 39.6 %)	3 % (-26 % to 31.9 %)	13.9 % (-14.4 % to 42.2 %)
3.4 % (-0.5 % to 7.3 %)	3.1 % (-1.3 % to 7.4 %)	3 % (-1.8 % to 7.7 %)	4.4 % (0.4 % to 8.4 %)	-4 % (-11.6 % to 3.6 %)	6.9 % (2.1 % to 11.8 %)
0.4 % (-2.4 % to 3.3 %)	0.2 % (-3.3 % to 3.6 %)	0 % (-3.9 % to 4 %)	1.5 % (-1.5 % to 4.5 %)	-6.9 % (-14 % to 0.2 %)	4 % (0 % to 8 %)

SCD

In [73]:

```
ch_sc$F2SCD_yn <- as.numeric(ch_sc$F2SCD_yn)

scd <- ch_sc %>% filter(!is.na(F2SCD_yn))

msm.totdairy <- geeglm(F2SCD_yn ~ total_dairy_b, data=scd, weights=ipw_totdairy,id
                       corstr="independence")
SEtotal <- coef(summary(msm.totdairy))[,2]

msm.fermdairy <- geeglm(F2SCD_yn ~ ferm_dairy_b, data=scd, weights=ipw_fermdairy, 
                corstr="independence")
SEferm <- coef(summary(msm.fermdairy))[,2]

msm.nonfermdairy <- geeglm(F2SCD_yn ~ nonferm_dairy_b, data=scd, weights=ipw_nonfe
                corstr="independence")
SEnonferm <- coef(summary(msm.nonfermdairy))[,2]

msm.ffdairy <- geeglm(F2SCD_yn ~ fullfat_dairy_b, data=scd, weights=ipw_fullfatdai
                corstr="independence")
```

```
SEff <- coef(summary(msm.ffdairy))[,2]

msm.nfdairy <- geeglm(F2SCD_yn ~ nonfat_dairy_b, data=scd, weights=ipw_nonfatdairy
                corstr="independence")
SEnf <- coef(summary(msm.nfdairy))[,2]

msm.sugdairy <- geeglm(F2SCD_yn ~ sugar_dairy_b, data=scd, weights=ipw_sugdairy, i
                corstr="independence")
SEsug <- coef(summary(msm.sugdairy))[,2]

msm.meat <- geeglm(F2SCD_yn ~ meat_b, data=scd, weights=ipw_meat, id=pt,
                corstr="independence")
SEmeat <- coef(summary(msm.meat))[,2]

msm.sea <- geeglm(F2SCD_yn ~ sea_b, data=scd, weights=ipw_sea, id=pt,
                corstr="independence")
SEsea <- coef(summary(msm.sea))[,2]


msm.veg <- geeglm(F2SCD_yn ~ veg_b, data=scd, weights=ipw_veg, id=pt,
                corstr="independence")
SEveg <- coef(summary(msm.veg))[,2]

msm.fru <- geeglm(F2SCD_yn ~ fru_b, data=scd, weights=ipw_fru, id=pt,
                corstr="independence")
SEfru <- coef(summary(msm.fru))[,2]


msm.eggs <- geeglm(F2SCD_yn ~ eggs_b, data=scd, weights=ipw_eggs, id=pt,
                corstr="independence")
SEeggs <- coef(summary(msm.eggs))[,2]
```

In [74]:

```
a <- ((coef(msm.totdairy) - coef(msm.meat))*100)[2]
b <- ((coef(msm.totdairy) - coef(msm.meat) - qnorm(0.975) * sqrt(SEtotal^2 + SEmea
c <- ((coef(msm.totdairy) - coef(msm.meat) + qnorm(0.975) * sqrt(SEtotal^2 + SEmea

d <- ((coef(msm.totdairy) - coef(msm.sea))*100)[2]
e <- ((coef(msm.totdairy) - coef(msm.sea) - qnorm(0.975) * sqrt(SEtotal^2 + SEsea^
f <- ((coef(msm.totdairy) - coef(msm.sea) + qnorm(0.975) * sqrt(SEtotal^2 + SEsea^

g <- ((coef(msm.totdairy) - coef(msm.eggs))*100)[2]
h <- ((coef(msm.totdairy) - coef(msm.eggs) - qnorm(0.975) * sqrt(SEtotal^2 + SEegg
i <- ((coef(msm.totdairy) - coef(msm.eggs) + qnorm(0.975) * sqrt(SEtotal^2 + SEegg

j <- ((coef(msm.totdairy) - coef(msm.veg))*100)[2]
k <- ((coef(msm.totdairy) - coef(msm.veg) - qnorm(0.975) * sqrt(SEtotal^2 + SEveg^
l <- ((coef(msm.totdairy) - coef(msm.veg) + qnorm(0.975) * sqrt(SEtotal^2 + SEveg^

m <- ((coef(msm.totdairy) - coef(msm.fru))*100)[2]
n <- ((coef(msm.totdairy) - coef(msm.fru) - qnorm(0.975) * sqrt(SEtotal^2 + SEfru^
o <- ((coef(msm.totdairy) - coef(msm.fru) + qnorm(0.975) * sqrt(SEtotal^2 + SEfru^

scdtotal <- c(paste(round(a,3)*100, "% (", round(b,3)*100,"% to ", round(c,3)*100,
              paste(round(d,3)*100, "% (", round(e,3)*100,"% to ", round(f,3)*100,
              paste(round(g,3)*100, "% (", round(h,3)*100,"% to ", round(i,3)*100,
              paste(round(j,3)*100, "% (", round(k,3)*100,"% to ", round(l,3)*100,
              paste(round(m,3)*100, "% (", round(n,3)*100,"% to ", round(o,3)*100,

a <- ((coef(msm.fermdairy) - coef(msm.meat))*100)[2]
b <- ((coef(msm.fermdairy) - coef(msm.meat) - qnorm(0.975) * sqrt(SEferm^2 + SEmea
c <- ((coef(msm.fermdairy) - coef(msm.meat) + qnorm(0.975) * sqrt(SEferm^2 + SEmea

d <- ((coef(msm.fermdairy) - coef(msm.sea))*100)[2]
```

```
e <- ((coef(msm.fermdairy) - coef(msm.sea) - qnorm(0.975) * sqrt(SEferm^2 + SEsea^
f <- ((coef(msm.fermdairy) - coef(msm.sea) + qnorm(0.975) * sqrt(SEferm^2 + SEsea^

g <- ((coef(msm.fermdairy) - coef(msm.eggs))*100)[2]
h <- ((coef(msm.fermdairy) - coef(msm.eggs) - qnorm(0.975) * sqrt(SEferm^2 + SEegg
i <- ((coef(msm.fermdairy) - coef(msm.eggs) + qnorm(0.975) * sqrt(SEferm^2 + SEegg

j <- ((coef(msm.fermdairy) - coef(msm.veg))*100)[2]
k <- ((coef(msm.fermdairy) - coef(msm.veg) - qnorm(0.975) * sqrt(SEferm^2 + SEveg^
l <- ((coef(msm.fermdairy) - coef(msm.veg) + qnorm(0.975) * sqrt(SEferm^2 + SEveg^

m <- ((coef(msm.fermdairy) - coef(msm.fru))*100)[2]
n <- ((coef(msm.fermdairy) - coef(msm.fru) - qnorm(0.975) * sqrt(SEferm^2 + SEfru^
o <- ((coef(msm.fermdairy) - coef(msm.fru) + qnorm(0.975) * sqrt(SEferm^2 + SEfru^

scdferm <- c(paste(round(a,3)*100, "% (", round(b,3)*100,"% to ", round(c,3)*100,"
              paste(round(d,3)*100, "% (", round(e,3)*100,"% to ", round(f,3)*100,
              paste(round(g,3)*100, "% (", round(h,3)*100,"% to ", round(i,3)*100,
              paste(round(j,3)*100, "% (", round(k,3)*100,"% to ", round(l,3)*100,
              paste(round(m,3)*100, "% (", round(n,3)*100,"% to ", round(o,3)*100,

a <- ((coef(msm.nonfermdairy) - coef(msm.meat))*100)[2]
b <- ((coef(msm.nonfermdairy) - coef(msm.meat) - qnorm(0.975) * sqrt(SEnonferm^2 +
c <- ((coef(msm.nonfermdairy) - coef(msm.meat) + qnorm(0.975) * sqrt(SEnonferm^2 +

d <- ((coef(msm.nonfermdairy) - coef(msm.sea))*100)[2]
e <- ((coef(msm.nonfermdairy) - coef(msm.sea) - qnorm(0.975) * sqrt(SEnonferm^2 +
f <- ((coef(msm.nonfermdairy) - coef(msm.sea) + qnorm(0.975) * sqrt(SEnonferm^2 +

g <- ((coef(msm.nonfermdairy) - coef(msm.eggs))*100)[2]
h <- ((coef(msm.nonfermdairy) - coef(msm.eggs) - qnorm(0.975) * sqrt(SEnonferm^2 +
i <- ((coef(msm.nonfermdairy) - coef(msm.eggs) + qnorm(0.975) * sqrt(SEnonferm^2 +

j <- ((coef(msm.nonfermdairy) - coef(msm.veg))*100)[2]
k <- ((coef(msm.nonfermdairy) - coef(msm.veg) - qnorm(0.975) * sqrt(SEnonferm^2 +
l <- ((coef(msm.nonfermdairy) - coef(msm.veg) + qnorm(0.975) * sqrt(SEnonferm^2 +

m <- ((coef(msm.nonfermdairy) - coef(msm.fru))*100)[2]
n <- ((coef(msm.nonfermdairy) - coef(msm.fru) - qnorm(0.975) * sqrt(SEnonferm^2 +
o <- ((coef(msm.nonfermdairy) - coef(msm.fru) + qnorm(0.975) * sqrt(SEnonferm^2 +

scdnonferm <- c(paste(round(a,3)*100, "% (", round(b,3)*100,"% to ", round(c,3)*10
              paste(round(d,3)*100, "% (", round(e,3)*100,"% to ", round(f,3)*100,
              paste(round(g,3)*100, "% (", round(h,3)*100,"% to ", round(i,3)*100,
              paste(round(j,3)*100, "% (", round(k,3)*100,"% to ", round(l,3)*100,
              paste(round(m,3)*100, "% (", round(n,3)*100,"% to ", round(o,3)*100,


a <- ((coef(msm.ffdairy) - coef(msm.meat))*100)[2]
b <- ((coef(msm.ffdairy) - coef(msm.meat) - qnorm(0.975) * sqrt(SEff^2 + SEmeat^2)
c <- ((coef(msm.ffdairy) - coef(msm.meat) + qnorm(0.975) * sqrt(SEff^2 + SEmeat^2)

d <- ((coef(msm.ffdairy) - coef(msm.sea))*100)[2]
e <- ((coef(msm.ffdairy) - coef(msm.sea) - qnorm(0.975) * sqrt(SEff^2 + SEsea^2))*
f <- ((coef(msm.ffdairy) - coef(msm.sea) + qnorm(0.975) * sqrt(SEff^2 + SEsea^2))*

g <- ((coef(msm.ffdairy) - coef(msm.eggs))*100)[2]
h <- ((coef(msm.ffdairy) - coef(msm.eggs) - qnorm(0.975) * sqrt(SEff^2 + SEeggs^2)
i <- ((coef(msm.ffdairy) - coef(msm.eggs) + qnorm(0.975) * sqrt(SEff^2 + SEeggs^2)

j <- ((coef(msm.ffdairy) - coef(msm.veg))*100)[2]
k <- ((coef(msm.ffdairy) - coef(msm.veg) - qnorm(0.975) * sqrt(SEff^2 + SEveg^2))*
l <- ((coef(msm.ffdairy) - coef(msm.veg) + qnorm(0.975) * sqrt(SEff^2 + SEveg^2))*
```

```
m <- ((coef(msm.ffdairy) - coef(msm.fru))*100)[2]
n <- ((coef(msm.ffdairy) - coef(msm.fru) - qnorm(0.975) * sqrt(SEff^2 + SEfru^2))*
o <- ((coef(msm.ffdairy) - coef(msm.fru) + qnorm(0.975) * sqrt(SEff^2 + SEfru^2))*

scdfullfat <- c(paste(round(a,3)*100, "% (", round(b,3)*100,"% to ", round(c,3)*10
              paste(round(d,3)*100, "% (", round(e,3)*100,"% to ", round(f,3)*100,
              paste(round(g,3)*100, "% (", round(h,3)*100,"% to ", round(i,3)*100,
              paste(round(j,3)*100, "% (", round(k,3)*100,"% to ", round(l,3)*100,
              paste(round(m,3)*100, "% (", round(n,3)*100,"% to ", round(o,3)*100,


a <- ((coef(msm.nfdairy) - coef(msm.meat))*100)[2]
b <- ((coef(msm.nfdairy) - coef(msm.meat) - qnorm(0.975) * sqrt(SEnf^2 + SEmeat^2)
c <- ((coef(msm.nfdairy) - coef(msm.meat) + qnorm(0.975) * sqrt(SEnf^2 + SEmeat^2)

d <- ((coef(msm.nfdairy) - coef(msm.sea))*100)[2]
e <- ((coef(msm.nfdairy) - coef(msm.sea) - qnorm(0.975) * sqrt(SEnf^2 + SEsea^2))*
f <- ((coef(msm.nfdairy) - coef(msm.sea) + qnorm(0.975) * sqrt(SEnf^2 + SEsea^2))*

g <- ((coef(msm.nfdairy) - coef(msm.eggs))*100)[2]
h <- ((coef(msm.nfdairy) - coef(msm.eggs) - qnorm(0.975) * sqrt(SEnf^2 + SEeggs^2)
i <- ((coef(msm.nfdairy) - coef(msm.eggs) + qnorm(0.975) * sqrt(SEnf^2 + SEeggs^2)

j <- ((coef(msm.nfdairy) - coef(msm.veg))*100)[2]
k <- ((coef(msm.nfdairy) - coef(msm.veg) - qnorm(0.975) * sqrt(SEnf^2 + SEveg^2))*
l <- ((coef(msm.nfdairy) - coef(msm.veg) + qnorm(0.975) * sqrt(SEnf^2 + SEveg^2))*

m <- ((coef(msm.nfdairy) - coef(msm.fru))*100)[2]
n <- ((coef(msm.nfdairy) - coef(msm.fru) - qnorm(0.975) * sqrt(SEnf^2 + SEfru^2))*
o <- ((coef(msm.nfdairy) - coef(msm.fru) + qnorm(0.975) * sqrt(SEnf^2 + SEfru^2))*

scdnonfat <- c(paste(round(a,3)*100, "% (", round(b,3)*100,"% to ", round(c,3)*100
              paste(round(d,3)*100, "% (", round(e,3)*100,"% to ", round(f,3)*100,
              paste(round(g,3)*100, "% (", round(h,3)*100,"% to ", round(i,3)*100,
              paste(round(j,3)*100, "% (", round(k,3)*100,"% to ", round(l,3)*100,
              paste(round(m,3)*100, "% (", round(n,3)*100,"% to ", round(o,3)*100,


a <- ((coef(msm.sugdairy) - coef(msm.meat))*100)[2]
b <- ((coef(msm.sugdairy) - coef(msm.meat) - qnorm(0.975) * sqrt(SEsug^2 + SEmeat^
c <- ((coef(msm.sugdairy) - coef(msm.meat) + qnorm(0.975) * sqrt(SEsug^2 + SEmeat^

d <- ((coef(msm.sugdairy) - coef(msm.sea))*100)[2]
e <- ((coef(msm.sugdairy) - coef(msm.sea) - qnorm(0.975) * sqrt(SEsug^2 + SEsea^2)
f <- ((coef(msm.sugdairy) - coef(msm.sea) + qnorm(0.975) * sqrt(SEsug^2 + SEsea^2)

g <- ((coef(msm.sugdairy) - coef(msm.eggs))*100)[2]
h <- ((coef(msm.sugdairy) - coef(msm.eggs) - qnorm(0.975) * sqrt(SEsug^2 + SEeggs^
i <- ((coef(msm.sugdairy) - coef(msm.eggs) + qnorm(0.975) * sqrt(SEsug^2 + SEeggs^

j <- ((coef(msm.sugdairy) - coef(msm.veg))*100)[2]
k <- ((coef(msm.sugdairy) - coef(msm.veg) - qnorm(0.975) * sqrt(SEsug^2 + SEveg^2)
l <- ((coef(msm.sugdairy) - coef(msm.veg) + qnorm(0.975) * sqrt(SEsug^2 + SEveg^2)

m <- ((coef(msm.sugdairy) - coef(msm.fru))*100)[2]
n <- ((coef(msm.sugdairy) - coef(msm.fru) - qnorm(0.975) * sqrt(SEsug^2 + SEfru^2)
o <- ((coef(msm.sugdairy) - coef(msm.fru) + qnorm(0.975) * sqrt(SEsug^2 + SEfru^2)

scdsug <- c(paste(round(a,3)*100, "% (", round(b,3)*100,"% to ", round(c,3)*100,"%
              paste(round(d,3)*100, "% (", round(e,3)*100,"% to ", round(f,3)*100,
              paste(round(g,3)*100, "% (", round(h,3)*100,"% to ", round(i,3)*100,
              paste(round(j,3)*100, "% (", round(k,3)*100,"% to ", round(l,3)*100,
              paste(round(m,3)*100, "% (", round(n,3)*100,"% to ", round(o,3)*100,
```

```
SCD <- data.frame(scdtotal,scdferm, scdnonferm, scdfullfat, scdnonfat, scdsug)
SCD
```

A data.frame: 5 × 6

scdtotal	scdferm	scdnonferm	scdfullfat	scdnonfat	scdsug
<chr>	<chr>	<chr>	<chr>	<chr>	<chr>
-0.8 % (-10.3 % to 8.6 %)	-2 % (-11.7 % to 7.7 %)	0.1 % (-9.6 % to 9.8 %)	-0.6 % (-10.1 % to 8.9 %)	-2.6 % (-13.2 % to 8 %)	0.2 % (-9.8 % to 10.2 %)
4.5 % (-7.3 % to 16.2 %)	3.3 % (-8.7 % to 15.3 %)	5.4 % (-6.6 % to 17.4 %)	4.7 % (-7.2 % to 16.5 %)	2.7 % (-10.1 % to 15.4 %)	5.5 % (-6.8 % to 17.7 %)
-1.7 % (-28.2 % to 24.8 %)	-2.9 % (-29.5 % to 23.7 %)	-0.8 % (-27.4 % to 25.8 %)	-1.5 % (-28 % to 25 %)	-3.5 % (-30.4 % to 23.4 %)	-0.7 % (-27.4 % to 26 %)
1 % (-1.9 % to 3.8 %)	-0.2 % (-3.9 % to 3.5 %)	1.9 % (-1.7 % to 5.5 %)	1.2 % (-1.9 % to 4.3 %)	-0.8 % (-6.4 % to 4.8 %)	2 % (-2.4 % to 6.4 %)
2.5 % (0.5 % to 4.5 %)	1.4 % (-1.7 % to 4.4 %)	3.4 % (0.5 % to 6.4 %)	2.7 % (0.4 % to 5.1 %)	0.7 % (-4.5 % to 6 %)	3.5 % (-0.4 % to 7.4 %)

Memory (binary)

In [77]:
```
ch_sc$F2memory_cat <- as.numeric(ch_sc$F2memory_cat)

mem <- ch_sc %>% filter(!is.na(F2memory_cat))

msm.totdairy <- geeglm(F2memory_cat ~ total_dairy_b, data=mem, weights=ipw_totdairy
                       corstr="independence")
SEtotal <- coef(summary(msm.totdairy))[,2]

msm.fermdairy <- geeglm(F2memory_cat ~ ferm_dairy_b, data=mem, weights=ipw_fermdai
                corstr="independence")
SEferm <- coef(summary(msm.fermdairy))[,2]

msm.nonfermdairy <- geeglm(F2memory_cat ~ nonferm_dairy_b, data=mem, weights=ipw_n
                corstr="independence")
SEnonferm <- coef(summary(msm.nonfermdairy))[,2]

msm.ffdairy <- geeglm(F2memory_cat ~ fullfat_dairy_b, data=mem, weights=ipw_fullfa
                corstr="independence")
SEff <- coef(summary(msm.ffdairy))[,2]

msm.nfdairy <- geeglm(F2memory_cat ~ nonfat_dairy_b, data=mem, weights=ipw_nonfatd
                corstr="independence")
SEnf <- coef(summary(msm.nfdairy))[,2]

msm.sugdairy <- geeglm(F2memory_cat ~ sugar_dairy_b, data=mem, weights=ipw_sugdair
                corstr="independence")
SEsug <- coef(summary(msm.sugdairy))[,2]

msm.meat <- geeglm(F2memory_cat ~ meat_b, data=mem, weights=ipw_meat, id=pt,
                corstr="independence")
SEmeat <- coef(summary(msm.meat))[,2]

msm.sea <- geeglm(F2memory_cat ~ sea_b, data=mem, weights=ipw_sea, id=pt,
                corstr="independence")
SEsea <- coef(summary(msm.sea))[,2]

msm.veg <- geeglm(F2memory_cat ~ veg_b, data=mem, weights=ipw_veg, id=pt,
                corstr="independence")
```

```
SEveg <- coef(summary(msm.veg))[,2]


msm.fru <- geeglm(F2memory_cat ~ fru_b, data=mem, weights=ipw_fru, id=pt,
                corstr="independence")
SEfru <- coef(summary(msm.fru))[,2]


msm.eggs <- geeglm(F2memory_cat ~ eggs_b, data=mem, weights=ipw_eggs, id=pt,
                corstr="independence")
SEeggs <- coef(summary(msm.eggs))[,2]

a <- ((coef(msm.totdairy) - coef(msm.meat))*100)[2]
b <- ((coef(msm.totdairy) - coef(msm.meat) - qnorm(0.975) * sqrt(SEtotal^2 + SEmea
c <- ((coef(msm.totdairy) - coef(msm.meat) + qnorm(0.975) * sqrt(SEtotal^2 + SEmea

d <- ((coef(msm.totdairy) - coef(msm.sea))*100)[2]
e <- ((coef(msm.totdairy) - coef(msm.sea) - qnorm(0.975) * sqrt(SEtotal^2 + SEsea^
f <- ((coef(msm.totdairy) - coef(msm.sea) + qnorm(0.975) * sqrt(SEtotal^2 + SEsea^

g <- ((coef(msm.totdairy) - coef(msm.eggs))*100)[2]
h <- ((coef(msm.totdairy) - coef(msm.eggs) - qnorm(0.975) * sqrt(SEtotal^2 + SEegg
i <- ((coef(msm.totdairy) - coef(msm.eggs) + qnorm(0.975) * sqrt(SEtotal^2 + SEegg

j <- ((coef(msm.totdairy) - coef(msm.veg))*100)[2]
k <- ((coef(msm.totdairy) - coef(msm.veg) - qnorm(0.975) * sqrt(SEtotal^2 + SEveg^
l <- ((coef(msm.totdairy) - coef(msm.veg) + qnorm(0.975) * sqrt(SEtotal^2 + SEveg^

m <- ((coef(msm.totdairy) - coef(msm.fru))*100)[2]
n <- ((coef(msm.totdairy) - coef(msm.fru) - qnorm(0.975) * sqrt(SEtotal^2 + SEfru^
o <- ((coef(msm.totdairy) - coef(msm.fru) + qnorm(0.975) * sqrt(SEtotal^2 + SEfru^

memorytotal <- c(paste(round(a,3)*100, "% (", round(b,3)*100,"% to ", round(c,3)*1
              paste(round(d,3)*100, "% (", round(e,3)*100,"% to ", round(f,3)*100,
              paste(round(g,3)*100, "% (", round(h,3)*100,"% to ", round(i,3)*100,
              paste(round(j,3)*100, "% (", round(k,3)*100,"% to ", round(l,3)*100,
              paste(round(m,3)*100, "% (", round(n,3)*100,"% to ", round(o,3)*100,

a <- ((coef(msm.fermdairy) - coef(msm.meat))*100)[2]
b <- ((coef(msm.fermdairy) - coef(msm.meat) - qnorm(0.975) * sqrt(SEferm^2 + SEmea
c <- ((coef(msm.fermdairy) - coef(msm.meat) + qnorm(0.975) * sqrt(SEferm^2 + SEmea

d <- ((coef(msm.fermdairy) - coef(msm.sea))*100)[2]
e <- ((coef(msm.fermdairy) - coef(msm.sea) - qnorm(0.975) * sqrt(SEferm^2 + SEsea^
f <- ((coef(msm.fermdairy) - coef(msm.sea) + qnorm(0.975) * sqrt(SEferm^2 + SEsea^

g <- ((coef(msm.fermdairy) - coef(msm.eggs))*100)[2]
h <- ((coef(msm.fermdairy) - coef(msm.eggs) - qnorm(0.975) * sqrt(SEferm^2 + SEegg
i <- ((coef(msm.fermdairy) - coef(msm.eggs) + qnorm(0.975) * sqrt(SEferm^2 + SEegg

j <- ((coef(msm.fermdairy) - coef(msm.veg))*100)[2]
k <- ((coef(msm.fermdairy) - coef(msm.veg) - qnorm(0.975) * sqrt(SEferm^2 + SEveg^
l <- ((coef(msm.fermdairy) - coef(msm.veg) + qnorm(0.975) * sqrt(SEferm^2 + SEveg^

m <- ((coef(msm.fermdairy) - coef(msm.fru))*100)[2]
n <- ((coef(msm.fermdairy) - coef(msm.fru) - qnorm(0.975) * sqrt(SEferm^2 + SEfru^
o <- ((coef(msm.fermdairy) - coef(msm.fru) + qnorm(0.975) * sqrt(SEferm^2 + SEfru^

memoryferm <- c(paste(round(a,3)*100, "% (", round(b,3)*100,"% to ", round(c,3)*10
              paste(round(d,3)*100, "% (", round(e,3)*100,"% to ", round(f,3)*100,
              paste(round(g,3)*100, "% (", round(h,3)*100,"% to ", round(i,3)*100,
              paste(round(j,3)*100, "% (", round(k,3)*100,"% to ", round(l,3)*100,
              paste(round(m,3)*100, "% (", round(n,3)*100,"% to ", round(o,3)*100,
```

```
a <- ((coef(msm.nonfermdairy) - coef(msm.meat))*100)[2]
b <- ((coef(msm.nonfermdairy) - coef(msm.meat) - qnorm(0.975) * sqrt(SEnonferm^2 +
c <- ((coef(msm.nonfermdairy) - coef(msm.meat) + qnorm(0.975) * sqrt(SEnonferm^2 +

d <- ((coef(msm.nonfermdairy) - coef(msm.sea))*100)[2]
e <- ((coef(msm.nonfermdairy) - coef(msm.sea) - qnorm(0.975) * sqrt(SEnonferm^2 + :
f <- ((coef(msm.nonfermdairy) - coef(msm.sea) + qnorm(0.975) * sqrt(SEnonferm^2 + :

g <- ((coef(msm.nonfermdairy) - coef(msm.eggs))*100)[2]
h <- ((coef(msm.nonfermdairy) - coef(msm.eggs) - qnorm(0.975) * sqrt(SEnonferm^2 +
i <- ((coef(msm.nonfermdairy) - coef(msm.eggs) + qnorm(0.975) * sqrt(SEnonferm^2 +

j <- ((coef(msm.nonfermdairy) - coef(msm.veg))*100)[2]
k <- ((coef(msm.nonfermdairy) - coef(msm.veg) - qnorm(0.975) * sqrt(SEnonferm^2 + :
l <- ((coef(msm.nonfermdairy) - coef(msm.veg) + qnorm(0.975) * sqrt(SEnonferm^2 + :

m <- ((coef(msm.nonfermdairy) - coef(msm.fru))*100)[2]
n <- ((coef(msm.nonfermdairy) - coef(msm.fru) - qnorm(0.975) * sqrt(SEnonferm^2 + :
o <- ((coef(msm.nonfermdairy) - coef(msm.fru) + qnorm(0.975) * sqrt(SEnonferm^2 + :

memorynonferm <- c(paste(round(a,3)*100, "% (", round(b,3)*100,"% to ", round(c,3)
                   paste(round(d,3)*100, "% (", round(e,3)*100,"% to ", round(f,3)*100,
                   paste(round(g,3)*100, "% (", round(h,3)*100,"% to ", round(i,3)*100,
                   paste(round(j,3)*100, "% (", round(k,3)*100,"% to ", round(l,3)*100,
                   paste(round(m,3)*100, "% (", round(n,3)*100,"% to ", round(o,3)*100,


a <- ((coef(msm.ffdairy) - coef(msm.meat))*100)[2]
b <- ((coef(msm.ffdairy) - coef(msm.meat) - qnorm(0.975) * sqrt(SEff^2 + SEmeat^2)
c <- ((coef(msm.ffdairy) - coef(msm.meat) + qnorm(0.975) * sqrt(SEff^2 + SEmeat^2)

d <- ((coef(msm.ffdairy) - coef(msm.sea))*100)[2]
e <- ((coef(msm.ffdairy) - coef(msm.sea) - qnorm(0.975) * sqrt(SEff^2 + SEsea^2))*
f <- ((coef(msm.ffdairy) - coef(msm.sea) + qnorm(0.975) * sqrt(SEff^2 + SEsea^2))*

g <- ((coef(msm.ffdairy) - coef(msm.eggs))*100)[2]
h <- ((coef(msm.ffdairy) - coef(msm.eggs) - qnorm(0.975) * sqrt(SEff^2 + SEeggs^2)
i <- ((coef(msm.ffdairy) - coef(msm.eggs) + qnorm(0.975) * sqrt(SEff^2 + SEeggs^2)

j <- ((coef(msm.ffdairy) - coef(msm.veg))*100)[2]
k <- ((coef(msm.ffdairy) - coef(msm.veg) - qnorm(0.975) * sqrt(SEff^2 + SEveg^2))*
l <- ((coef(msm.ffdairy) - coef(msm.veg) + qnorm(0.975) * sqrt(SEff^2 + SEveg^2))*

m <- ((coef(msm.ffdairy) - coef(msm.fru))*100)[2]
n <- ((coef(msm.ffdairy) - coef(msm.fru) - qnorm(0.975) * sqrt(SEff^2 + SEfru^2))*
o <- ((coef(msm.ffdairy) - coef(msm.fru) + qnorm(0.975) * sqrt(SEff^2 + SEfru^2))*

memoryfullfat <- c(paste(round(a,3)*100, "% (", round(b,3)*100,"% to ", round(c,3)
                   paste(round(d,3)*100, "% (", round(e,3)*100,"% to ", round(f,3)*100,
                   paste(round(g,3)*100, "% (", round(h,3)*100,"% to ", round(i,3)*100,
                   paste(round(j,3)*100, "% (", round(k,3)*100,"% to ", round(l,3)*100,
                   paste(round(m,3)*100, "% (", round(n,3)*100,"% to ", round(o,3)*100,


a <- ((coef(msm.nfdairy) - coef(msm.meat))*100)[2]
b <- ((coef(msm.nfdairy) - coef(msm.meat) - qnorm(0.975) * sqrt(SEnf^2 + SEmeat^2)
c <- ((coef(msm.nfdairy) - coef(msm.meat) + qnorm(0.975) * sqrt(SEnf^2 + SEmeat^2)

d <- ((coef(msm.nfdairy) - coef(msm.sea))*100)[2]
e <- ((coef(msm.nfdairy) - coef(msm.sea) - qnorm(0.975) * sqrt(SEnf^2 + SEsea^2))*
f <- ((coef(msm.nfdairy) - coef(msm.sea) + qnorm(0.975) * sqrt(SEnf^2 + SEsea^2))*

g <- ((coef(msm.nfdairy) - coef(msm.eggs))*100)[2]
h <- ((coef(msm.nfdairy) - coef(msm.eggs) - qnorm(0.975) * sqrt(SEnf^2 + SEeggs^2)
```

```
i <- ((coef(msm.nfdairy) - coef(msm.eggs) + qnorm(0.975) * sqrt(SEnf^2 + SEeggs^2)

j <- ((coef(msm.nfdairy) - coef(msm.veg))*100)[2]
k <- ((coef(msm.nfdairy) - coef(msm.veg) - qnorm(0.975) * sqrt(SEnf^2 + SEveg^2))*
l <- ((coef(msm.nfdairy) - coef(msm.veg) + qnorm(0.975) * sqrt(SEnf^2 + SEveg^2))*

m <- ((coef(msm.nfdairy) - coef(msm.fru))*100)[2]
n <- ((coef(msm.nfdairy) - coef(msm.fru) - qnorm(0.975) * sqrt(SEnf^2 + SEfru^2))*
o <- ((coef(msm.nfdairy) - coef(msm.fru) + qnorm(0.975) * sqrt(SEnf^2 + SEfru^2))*

memorynonfat <- c(paste(round(a,3)*100, "% (", round(b,3)*100,"% to ", round(c,3)*
              paste(round(d,3)*100, "% (", round(e,3)*100,"% to ", round(f,3)*100,
              paste(round(g,3)*100, "% (", round(h,3)*100,"% to ", round(i,3)*100,
              paste(round(j,3)*100, "% (", round(k,3)*100,"% to ", round(l,3)*100,
              paste(round(m,3)*100, "% (", round(n,3)*100,"% to ", round(o,3)*100,


a <- ((coef(msm.sugdairy) - coef(msm.meat))*100)[2]
b <- ((coef(msm.sugdairy) - coef(msm.meat) - qnorm(0.975) * sqrt(SEsug^2 + SEmeat^
c <- ((coef(msm.sugdairy) - coef(msm.meat) + qnorm(0.975) * sqrt(SEsug^2 + SEmeat^

d <- ((coef(msm.sugdairy) - coef(msm.sea))*100)[2]
e <- ((coef(msm.sugdairy) - coef(msm.sea) - qnorm(0.975) * sqrt(SEsug^2 + SEsea^2)
f <- ((coef(msm.sugdairy) - coef(msm.sea) + qnorm(0.975) * sqrt(SEsug^2 + SEsea^2)

g <- ((coef(msm.sugdairy) - coef(msm.eggs))*100)[2]
h <- ((coef(msm.sugdairy) - coef(msm.eggs) - qnorm(0.975) * sqrt(SEsug^2 + SEeggs^
i <- ((coef(msm.sugdairy) - coef(msm.eggs) + qnorm(0.975) * sqrt(SEsug^2 + SEeggs^

j <- ((coef(msm.sugdairy) - coef(msm.veg))*100)[2]
k <- ((coef(msm.sugdairy) - coef(msm.veg) - qnorm(0.975) * sqrt(SEsug^2 + SEveg^2)
l <- ((coef(msm.sugdairy) - coef(msm.veg) + qnorm(0.975) * sqrt(SEsug^2 + SEveg^2)

m <- ((coef(msm.sugdairy) - coef(msm.fru))*100)[2]
n <- ((coef(msm.sugdairy) - coef(msm.fru) - qnorm(0.975) * sqrt(SEsug^2 + SEfru^2)
o <- ((coef(msm.sugdairy) - coef(msm.fru) + qnorm(0.975) * sqrt(SEsug^2 + SEfru^2)

memorysug <- c(paste(round(a,3)*100, "% (", round(b,3)*100,"% to ", round(c,3)*100
              paste(round(d,3)*100, "% (", round(e,3)*100,"% to ", round(f,3)*100,
              paste(round(g,3)*100, "% (", round(h,3)*100,"% to ", round(i,3)*100,
              paste(round(j,3)*100, "% (", round(k,3)*100,"% to ", round(l,3)*100,
              paste(round(m,3)*100, "% (", round(n,3)*100,"% to ", round(o,3)*100,

MEMORY <- data.frame(memorytotal,memoryferm, memorynonferm, memoryfullfat, memoryn
MEMORY
```

A data.frame: 5 × 6

memorytotal	memoryferm	memorynonferm	memoryfullfat	memorynonfat	memorysug
<chr>	<chr>	<chr>	<chr>	<chr>	<chr>
-1.1 % (-9.9 % to 7.7 %)	0.3 % (-9.2 % to 9.8 %)	-0.5 % (-9.6 % to 8.5 %)	-0.3 % (-9.2 % to 8.7 %)	-1.4 % (-11.3 % to 8.5 %)	-1.7 % (-11.1 % to 7.7 %)
-3.3 % (-17.6 % to 11 %)	-2 % (-16.7 % to 12.7 %)	-2.8 % (-17.3 % to 11.7 %)	-2.5 % (-16.9 % to 11.9 %)	-3.6 % (-18.7 % to 11.4 %)	-4 % (-18.6 % to 10.7 %)
15 % (-1.9 % to 31.9 %)	16.4 % (-0.9 % to 33.6 %)	15.6 % (-1.5 % to 32.6 %)	15.8 % (-1.1 % to 32.8 %)	14.7 % (-2.8 % to 32.2 %)	14.4 % (-2.8 % to 31.6 %)
-0.4 % (-5.1 % to 4.3 %)	0.9 % (-4.9 % to 6.8 %)	0.1 % (-5.1 % to 5.3 %)	0.4 % (-4.5 % to 5.3 %)	-0.7 % (-7.3 % to 5.8 %)	-1.1 % (-6.8 % to 4.6 %)
-0.4 % (-3 % to 2.3 %)	1 % (-3.4 % to 5.4 %)	0.2 % (-3.3 % to 3.6 %)	0.5 % (-2.6 % to 3.5 %)	-0.7 % (-5.9 % to 4.6 %)	-1 % (-5.2 % to 3.2 %)

Veral (Binary)

In [78]:
```
ch_sc$F2verbal_cat <- as.numeric(ch_sc$F2verbal_cat)

ver <- ch_sc %>% filter(!is.na(F2verbal_cat))

msm.totdairy <- geeglm(F2verbal_cat ~ total_dairy_b, data=ver, weights=ipw_totdairy
                       corstr="independence")

SEtotal <- coef(summary(msm.totdairy))[,2]

msm.fermdairy <- geeglm(F2verbal_cat ~ ferm_dairy_b, data=ver, weights=ipw_fermdai
                        corstr="independence")

SEferm <- coef(summary(msm.fermdairy))[,2]

msm.nonfermdairy <- geeglm(F2verbal_cat ~ nonferm_dairy_b, data=ver, weights=ipw_no
                        corstr="independence")

SEnonferm <- coef(summary(msm.nonfermdairy))[,2]

msm.ffdairy <- geeglm(F2verbal_cat ~ fullfat_dairy_b, data=ver, weights=ipw_fullfat
                        corstr="independence")

SEff <- coef(summary(msm.ffdairy))[,2]

msm.nfdairy <- geeglm(F2verbal_cat ~ nonfat_dairy_b, data=ver, weights=ipw_nonfatda
                        corstr="independence")

SEnf <- coef(summary(msm.nfdairy))[,2]

msm.sugdairy <- geeglm(F2verbal_cat ~ sugar_dairy_b, data=ver, weights=ipw_sugdairy
                        corstr="independence")
SEsug <- coef(summary(msm.sugdairy))[,2]

msm.meat <- geeglm(F2verbal_cat ~ meat_b, data=ver, weights=ipw_meat, id=pt,
                        corstr="independence")
SEmeat <- coef(summary(msm.meat))[,2]

msm.sea <- geeglm(F2verbal_cat ~ sea_b, data=ver, weights=ipw_sea, id=pt,
                        corstr="independence")
SEsea <- coef(summary(msm.sea))[,2]
```

```
msm.veg <- geeglm(F2verbal_cat ~ veg_b, data=ver, weights=ipw_veg, id=pt,
                corstr="independence")
SEveg <- coef(summary(msm.veg))[,2]

msm.fru <- geeglm(F2verbal_cat ~ fru_b, data=ver, weights=ipw_fru, id=pt,
                corstr="independence")
SEfru <- coef(summary(msm.fru))[,2]


msm.eggs <- geeglm(F2verbal_cat ~ eggs_b, data=ver, weights=ipw_eggs, id=pt,
                corstr="independence")
SEeggs <- coef(summary(msm.eggs))[,2]


a <- ((coef(msm.totdairy) - coef(msm.meat))*100)[2]
b <- ((coef(msm.totdairy) - coef(msm.meat) - qnorm(0.975) * sqrt(SEtotal^2 + SEmea
c <- ((coef(msm.totdairy) - coef(msm.meat) + qnorm(0.975) * sqrt(SEtotal^2 + SEmea

d <- ((coef(msm.totdairy) - coef(msm.sea))*100)[2]
e <- ((coef(msm.totdairy) - coef(msm.sea) - qnorm(0.975) * sqrt(SEtotal^2 + SEsea^
f <- ((coef(msm.totdairy) - coef(msm.sea) + qnorm(0.975) * sqrt(SEtotal^2 + SEsea^

g <- ((coef(msm.totdairy) - coef(msm.eggs))*100)[2]
h <- ((coef(msm.totdairy) - coef(msm.eggs) - qnorm(0.975) * sqrt(SEtotal^2 + SEegg
i <- ((coef(msm.totdairy) - coef(msm.eggs) + qnorm(0.975) * sqrt(SEtotal^2 + SEegg

j <- ((coef(msm.totdairy) - coef(msm.veg))*100)[2]
k <- ((coef(msm.totdairy) - coef(msm.veg) - qnorm(0.975) * sqrt(SEtotal^2 + SEveg^
l <- ((coef(msm.totdairy) - coef(msm.veg) + qnorm(0.975) * sqrt(SEtotal^2 + SEveg^

m <- ((coef(msm.totdairy) - coef(msm.fru))*100)[2]
n <- ((coef(msm.totdairy) - coef(msm.fru) - qnorm(0.975) * sqrt(SEtotal^2 + SEfru^
o <- ((coef(msm.totdairy) - coef(msm.fru) + qnorm(0.975) * sqrt(SEtotal^2 + SEfru^

verbaltotal <- c(paste(round(a,3)*100, "% (", round(b,3)*100,"% to ", round(c,3)*1
              paste(round(d,3)*100, "% (", round(e,3)*100,"% to ", round(f,3)*100,
              paste(round(g,3)*100, "% (", round(h,3)*100,"% to ", round(i,3)*100,
              paste(round(j,3)*100, "% (", round(k,3)*100,"% to ", round(l,3)*100,
              paste(round(m,3)*100, "% (", round(n,3)*100,"% to ", round(o,3)*100,

a <- ((coef(msm.fermdairy) - coef(msm.meat))*100)[2]
b <- ((coef(msm.fermdairy) - coef(msm.meat) - qnorm(0.975) * sqrt(SEferm^2 + SEmea
c <- ((coef(msm.fermdairy) - coef(msm.meat) + qnorm(0.975) * sqrt(SEferm^2 + SEmea

d <- ((coef(msm.fermdairy) - coef(msm.sea))*100)[2]
e <- ((coef(msm.fermdairy) - coef(msm.sea) - qnorm(0.975) * sqrt(SEferm^2 + SEsea^
f <- ((coef(msm.fermdairy) - coef(msm.sea) + qnorm(0.975) * sqrt(SEferm^2 + SEsea^

g <- ((coef(msm.fermdairy) - coef(msm.eggs))*100)[2]
h <- ((coef(msm.fermdairy) - coef(msm.eggs) - qnorm(0.975) * sqrt(SEferm^2 + SEegg
i <- ((coef(msm.fermdairy) - coef(msm.eggs) + qnorm(0.975) * sqrt(SEferm^2 + SEegg

j <- ((coef(msm.fermdairy) - coef(msm.veg))*100)[2]
k <- ((coef(msm.fermdairy) - coef(msm.veg) - qnorm(0.975) * sqrt(SEferm^2 + SEveg^
l <- ((coef(msm.fermdairy) - coef(msm.veg) + qnorm(0.975) * sqrt(SEferm^2 + SEveg^

m <- ((coef(msm.fermdairy) - coef(msm.fru))*100)[2]
n <- ((coef(msm.fermdairy) - coef(msm.fru) - qnorm(0.975) * sqrt(SEferm^2 + SEfru^
o <- ((coef(msm.fermdairy) - coef(msm.fru) + qnorm(0.975) * sqrt(SEferm^2 + SEfru^

verbalferm <- c(paste(round(a,3)*100, "% (", round(b,3)*100,"% to ", round(c,3)*10
              paste(round(d,3)*100, "% (", round(e,3)*100,"% to ", round(f,3)*100,
              paste(round(g,3)*100, "% (", round(h,3)*100,"% to ", round(i,3)*100,
```

```
              paste(round(j,3)*100, "% (", round(k,3)*100,"% to ", round(l,3)*100,
              paste(round(m,3)*100, "% (", round(n,3)*100,"% to ", round(o,3)*100,

a <- ((coef(msm.nonfermdairy) - coef(msm.meat))*100)[2]
b <- ((coef(msm.nonfermdairy) - coef(msm.meat) - qnorm(0.975) * sqrt(SEnonferm^2 +
c <- ((coef(msm.nonfermdairy) - coef(msm.meat) + qnorm(0.975) * sqrt(SEnonferm^2 +

d <- ((coef(msm.nonfermdairy) - coef(msm.sea))*100)[2]
e <- ((coef(msm.nonfermdairy) - coef(msm.sea) - qnorm(0.975) * sqrt(SEnonferm^2 +
f <- ((coef(msm.nonfermdairy) - coef(msm.sea) + qnorm(0.975) * sqrt(SEnonferm^2 +

g <- ((coef(msm.nonfermdairy) - coef(msm.eggs))*100)[2]
h <- ((coef(msm.nonfermdairy) - coef(msm.eggs) - qnorm(0.975) * sqrt(SEnonferm^2 +
i <- ((coef(msm.nonfermdairy) - coef(msm.eggs) + qnorm(0.975) * sqrt(SEnonferm^2 +

j <- ((coef(msm.nonfermdairy) - coef(msm.veg))*100)[2]
k <- ((coef(msm.nonfermdairy) - coef(msm.veg) - qnorm(0.975) * sqrt(SEnonferm^2 +
l <- ((coef(msm.nonfermdairy) - coef(msm.veg) + qnorm(0.975) * sqrt(SEnonferm^2 +

m <- ((coef(msm.nonfermdairy) - coef(msm.fru))*100)[2]
n <- ((coef(msm.nonfermdairy) - coef(msm.fru) - qnorm(0.975) * sqrt(SEnonferm^2 +
o <- ((coef(msm.nonfermdairy) - coef(msm.fru) + qnorm(0.975) * sqrt(SEnonferm^2 +

verbalnonferm <- c(paste(round(a,3)*100, "% (", round(b,3)*100,"% to ", round(c,3)
              paste(round(d,3)*100, "% (", round(e,3)*100,"% to ", round(f,3)*100,
              paste(round(g,3)*100, "% (", round(h,3)*100,"% to ", round(i,3)*100,
              paste(round(j,3)*100, "% (", round(k,3)*100,"% to ", round(l,3)*100,
              paste(round(m,3)*100, "% (", round(n,3)*100,"% to ", round(o,3)*100,


a <- ((coef(msm.ffdairy) - coef(msm.meat))*100)[2]
b <- ((coef(msm.ffdairy) - coef(msm.meat) - qnorm(0.975) * sqrt(SEff^2 + SEmeat^2)
c <- ((coef(msm.ffdairy) - coef(msm.meat) + qnorm(0.975) * sqrt(SEff^2 + SEmeat^2)

d <- ((coef(msm.ffdairy) - coef(msm.sea))*100)[2]
e <- ((coef(msm.ffdairy) - coef(msm.sea) - qnorm(0.975) * sqrt(SEff^2 + SEsea^2))*
f <- ((coef(msm.ffdairy) - coef(msm.sea) + qnorm(0.975) * sqrt(SEff^2 + SEsea^2))*

g <- ((coef(msm.ffdairy) - coef(msm.eggs))*100)[2]
h <- ((coef(msm.ffdairy) - coef(msm.eggs) - qnorm(0.975) * sqrt(SEff^2 + SEeggs^2)
i <- ((coef(msm.ffdairy) - coef(msm.eggs) + qnorm(0.975) * sqrt(SEff^2 + SEeggs^2)

j <- ((coef(msm.ffdairy) - coef(msm.veg))*100)[2]
k <- ((coef(msm.ffdairy) - coef(msm.veg) - qnorm(0.975) * sqrt(SEff^2 + SEveg^2))*
l <- ((coef(msm.ffdairy) - coef(msm.veg) + qnorm(0.975) * sqrt(SEff^2 + SEveg^2))*

m <- ((coef(msm.ffdairy) - coef(msm.fru))*100)[2]
n <- ((coef(msm.ffdairy) - coef(msm.fru) - qnorm(0.975) * sqrt(SEff^2 + SEfru^2))*
o <- ((coef(msm.ffdairy) - coef(msm.fru) + qnorm(0.975) * sqrt(SEff^2 + SEfru^2))*

verbalfullfat <- c(paste(round(a,3)*100, "% (", round(b,3)*100,"% to ", round(c,3)
              paste(round(d,3)*100, "% (", round(e,3)*100,"% to ", round(f,3)*100,
              paste(round(g,3)*100, "% (", round(h,3)*100,"% to ", round(i,3)*100,
              paste(round(j,3)*100, "% (", round(k,3)*100,"% to ", round(l,3)*100,
              paste(round(m,3)*100, "% (", round(n,3)*100,"% to ", round(o,3)*100,


a <- ((coef(msm.nfdairy) - coef(msm.meat))*100)[2]
b <- ((coef(msm.nfdairy) - coef(msm.meat) - qnorm(0.975) * sqrt(SEnf^2 + SEmeat^2)
c <- ((coef(msm.nfdairy) - coef(msm.meat) + qnorm(0.975) * sqrt(SEnf^2 + SEmeat^2)

d <- ((coef(msm.nfdairy) - coef(msm.sea))*100)[2]
e <- ((coef(msm.nfdairy) - coef(msm.sea) - qnorm(0.975) * sqrt(SEnf^2 + SEsea^2))*
f <- ((coef(msm.nfdairy) - coef(msm.sea) + qnorm(0.975) * sqrt(SEnf^2 + SEsea^2))*
```

```
g <- ((coef(msm.nfdairy) - coef(msm.eggs))*100)[2]
h <- ((coef(msm.nfdairy) - coef(msm.eggs) - qnorm(0.975) * sqrt(SEnf^2 + SEeggs^2)
i <- ((coef(msm.nfdairy) - coef(msm.eggs) + qnorm(0.975) * sqrt(SEnf^2 + SEeggs^2)

j <- ((coef(msm.nfdairy) - coef(msm.veg))*100)[2]
k <- ((coef(msm.nfdairy) - coef(msm.veg) - qnorm(0.975) * sqrt(SEnf^2 + SEveg^2))*
l <- ((coef(msm.nfdairy) - coef(msm.veg) + qnorm(0.975) * sqrt(SEnf^2 + SEveg^2))*

m <- ((coef(msm.nfdairy) - coef(msm.fru))*100)[2]
n <- ((coef(msm.nfdairy) - coef(msm.fru) - qnorm(0.975) * sqrt(SEnf^2 + SEfru^2))*
o <- ((coef(msm.nfdairy) - coef(msm.fru) + qnorm(0.975) * sqrt(SEnf^2 + SEfru^2))*

verbalnonfat <- c(paste(round(a,3)*100, "% (", round(b,3)*100,"% to ", round(c,3)*
              paste(round(d,3)*100, "% (", round(e,3)*100,"% to ", round(f,3)*100,
              paste(round(g,3)*100, "% (", round(h,3)*100,"% to ", round(i,3)*100,
              paste(round(j,3)*100, "% (", round(k,3)*100,"% to ", round(l,3)*100,
              paste(round(m,3)*100, "% (", round(n,3)*100,"% to ", round(o,3)*100,


a <- ((coef(msm.sugdairy) - coef(msm.meat))*100)[2]
b <- ((coef(msm.sugdairy) - coef(msm.meat) - qnorm(0.975) * sqrt(SEsug^2 + SEmeat^
c <- ((coef(msm.sugdairy) - coef(msm.meat) + qnorm(0.975) * sqrt(SEsug^2 + SEmeat^

d <- ((coef(msm.sugdairy) - coef(msm.sea))*100)[2]
e <- ((coef(msm.sugdairy) - coef(msm.sea) - qnorm(0.975) * sqrt(SEsug^2 + SEsea^2)
f <- ((coef(msm.sugdairy) - coef(msm.sea) + qnorm(0.975) * sqrt(SEsug^2 + SEsea^2)

g <- ((coef(msm.sugdairy) - coef(msm.eggs))*100)[2]
h <- ((coef(msm.sugdairy) - coef(msm.eggs) - qnorm(0.975) * sqrt(SEsug^2 + SEeggs^
i <- ((coef(msm.sugdairy) - coef(msm.eggs) + qnorm(0.975) * sqrt(SEsug^2 + SEeggs^

j <- ((coef(msm.sugdairy) - coef(msm.veg))*100)[2]
k <- ((coef(msm.sugdairy) - coef(msm.veg) - qnorm(0.975) * sqrt(SEsug^2 + SEveg^2)
l <- ((coef(msm.sugdairy) - coef(msm.veg) + qnorm(0.975) * sqrt(SEsug^2 + SEveg^2)

m <- ((coef(msm.sugdairy) - coef(msm.fru))*100)[2]
n <- ((coef(msm.sugdairy) - coef(msm.fru) - qnorm(0.975) * sqrt(SEsug^2 + SEfru^2)
o <- ((coef(msm.sugdairy) - coef(msm.fru) + qnorm(0.975) * sqrt(SEsug^2 + SEfru^2)

verbalsug <- c(paste(round(a,3)*100, "% (", round(b,3)*100,"% to ", round(c,3)*100
              paste(round(d,3)*100, "% (", round(e,3)*100,"% to ", round(f,3)*100,
              paste(round(g,3)*100, "% (", round(h,3)*100,"% to ", round(i,3)*100,
              paste(round(j,3)*100, "% (", round(k,3)*100,"% to ", round(l,3)*100,
              paste(round(m,3)*100, "% (", round(n,3)*100,"% to ", round(o,3)*100,

VERBAL <- data.frame(verbaltotal,verbalferm, verbalnonferm, verbalfullfat, verbaln
VERBAL
```

A data.frame: 5 × 6

verbaltotal	verbalferm	verbalnonferm	verbalfullfat	verbalnonfat	verbalsug
<chr>	<chr>	<chr>	<chr>	<chr>	<chr>
5.3 % (-3 % to 13.6 %)	4.3 % (-4.3 % to 12.8 %)	4.5 % (-4.3 % to 13.2 %)	4.5 % (-3.9 % to 12.9 %)	7.3 % (-2.4 % to 17.1 %)	7.1 % (-1.6 % to 15.8 %)
10.2 % (-3.7 % to 24 %)	9.1 % (-4.8 % to 23.1 %)	9.3 % (-4.8 % to 23.4 %)	9.4 % (-4.5 % to 23.3 %)	12.2 % (-2.5 % to 26.9 %)	12 % (-2.1 % to 26.1 %)
-5 % (-27 % to 17.1 %)	-6 % (-28.2 % to 16.1 %)	-5.8 % (-28 % to 16.4 %)	-5.8 % (-27.8 % to 16.3 %)	-2.9 % (-25.6 % to 19.7 %)	-3.2 % (-25.4 % to 19.1 %)
-0.9 % (-4.1 % to 2.3 %)	-1.9 % (-5.7 % to 1.9 %)	-1.7 % (-6 % to 2.6 %)	-1.6 % (-5.1 % to 1.8 %)	1.2 % (-4.9 % to 7.2 %)	1 % (-3.2 % to 5.1 %)
0.1 % (-2.2 % to 2.5 %)	-0.9 % (-3.9 % to 2.2 %)	-0.7 % (-4.4 % to 3 %)	-0.6 % (-3.3 % to 2.1 %)	2.2 % (-3.5 % to 7.8 %)	2 % (-1.5 % to 5.5 %)

Stroop (binary)

In [79]:
```
ch_sc$F2stroop_cat <- as.numeric(ch_sc$F2stroop_cat)

stroop <- ch_sc %>% filter(!is.na(F2stroop_cat))

msm.totdairy <- geeglm(F2stroop_cat ~ total_dairy_b, data=stroop, weights=ipw_totda
                       corstr="independence")
SEtotal <- coef(summary(msm.totdairy))[,2]

msm.fermdairy <- geeglm(F2stroop_cat ~ ferm_dairy_b, data=stroop, weights=ipw_ferm
                corstr="independence")
SEferm <- coef(summary(msm.fermdairy))[,2]

msm.nonfermdairy <- geeglm(F2stroop_cat ~ nonferm_dairy_b, data=stroop, weights=ipw
                corstr="independence")
SEnonferm <- coef(summary(msm.nonfermdairy))[,2]

msm.ffdairy <- geeglm(F2stroop_cat ~ fullfat_dairy_b, data=stroop, weights=ipw_ful
                corstr="independence")
SEff <- coef(summary(msm.ffdairy))[,2]

msm.nfdairy <- geeglm(F2stroop_cat ~ nonfat_dairy_b, data=stroop, weights=ipw_nonf
                corstr="independence")
SEnf <- coef(summary(msm.nfdairy))[,2]

msm.sugdairy <- geeglm(F2stroop_cat ~ sugar_dairy_b, data=stroop, weights=ipw_sugd
                corstr="independence")
SEsug <- coef(summary(msm.sugdairy))[,2]

msm.meat <- geeglm(F2stroop_cat ~ meat_b, data=stroop, weights=ipw_meat, id=pt,
                corstr="independence")
SEmeat <- coef(summary(msm.meat))[,2]

msm.sea <- geeglm(F2stroop_cat ~ sea_b, data=stroop, weights=ipw_sea, id=pt,
                corstr="independence")
SEsea <- coef(summary(msm.sea))[,2]


msm.veg <- geeglm(F2stroop_cat ~ veg_b, data=stroop, weights=ipw_veg, id=pt,
                corstr="independence")
SEveg <- coef(summary(msm.veg))[,2]
```

```
msm.fru <- geeglm(F2stroop_cat ~ fru_b, data=stroop, weights=ipw_fru, id=pt,
                corstr="independence")
SEfru <- coef(summary(msm.fru))[,2]


msm.eggs <- geeglm(F2stroop_cat ~ eggs_b, data=stroop, weights=ipw_eggs, id=pt,
                corstr="independence")
SEeggs <- coef(summary(msm.eggs))[,2]


a <- ((coef(msm.totdairy) - coef(msm.meat))*100)[2]
b <- ((coef(msm.totdairy) - coef(msm.meat) - qnorm(0.975) * sqrt(SEtotal^2 + SEmea
c <- ((coef(msm.totdairy) - coef(msm.meat) + qnorm(0.975) * sqrt(SEtotal^2 + SEmea

d <- ((coef(msm.totdairy) - coef(msm.sea))*100)[2]
e <- ((coef(msm.totdairy) - coef(msm.sea) - qnorm(0.975) * sqrt(SEtotal^2 + SEsea^
f <- ((coef(msm.totdairy) - coef(msm.sea) + qnorm(0.975) * sqrt(SEtotal^2 + SEsea^

g <- ((coef(msm.totdairy) - coef(msm.eggs))*100)[2]
h <- ((coef(msm.totdairy) - coef(msm.eggs) - qnorm(0.975) * sqrt(SEtotal^2 + SEegg
i <- ((coef(msm.totdairy) - coef(msm.eggs) + qnorm(0.975) * sqrt(SEtotal^2 + SEegg

j <- ((coef(msm.totdairy) - coef(msm.veg))*100)[2]
k <- ((coef(msm.totdairy) - coef(msm.veg) - qnorm(0.975) * sqrt(SEtotal^2 + SEveg^
l <- ((coef(msm.totdairy) - coef(msm.veg) + qnorm(0.975) * sqrt(SEtotal^2 + SEveg^

m <- ((coef(msm.totdairy) - coef(msm.fru))*100)[2]
n <- ((coef(msm.totdairy) - coef(msm.fru) - qnorm(0.975) * sqrt(SEtotal^2 + SEfru^
o <- ((coef(msm.totdairy) - coef(msm.fru) + qnorm(0.975) * sqrt(SEtotal^2 + SEfru^

strooptotal <- c(paste(round(a,3)*100, "% (", round(b,3)*100,"% to", round(c,3)*10
              paste(round(d,3)*100, "% (", round(e,3)*100,"% to", round(f,3)*100,"
              paste(round(g,3)*100, "% (", round(h,3)*100,"% to", round(i,3)*100,"
              paste(round(j,3)*100, "% (", round(k,3)*100,"% to", round(l,3)*100,"
              paste(round(m,3)*100, "% (", round(n,3)*100,"% to", round(o,3)*100,"

a <- ((coef(msm.fermdairy) - coef(msm.meat))*100)[2]
b <- ((coef(msm.fermdairy) - coef(msm.meat) - qnorm(0.975) * sqrt(SEferm^2 + SEmea
c <- ((coef(msm.fermdairy) - coef(msm.meat) + qnorm(0.975) * sqrt(SEferm^2 + SEmea

d <- ((coef(msm.fermdairy) - coef(msm.sea))*100)[2]
e <- ((coef(msm.fermdairy) - coef(msm.sea) - qnorm(0.975) * sqrt(SEferm^2 + SEsea^
f <- ((coef(msm.fermdairy) - coef(msm.sea) + qnorm(0.975) * sqrt(SEferm^2 + SEsea^

g <- ((coef(msm.fermdairy) - coef(msm.eggs))*100)[2]
h <- ((coef(msm.fermdairy) - coef(msm.eggs) - qnorm(0.975) * sqrt(SEferm^2 + SEegg
i <- ((coef(msm.fermdairy) - coef(msm.eggs) + qnorm(0.975) * sqrt(SEferm^2 + SEegg

j <- ((coef(msm.fermdairy) - coef(msm.veg))*100)[2]
k <- ((coef(msm.fermdairy) - coef(msm.veg) - qnorm(0.975) * sqrt(SEferm^2 + SEveg^
l <- ((coef(msm.fermdairy) - coef(msm.veg) + qnorm(0.975) * sqrt(SEferm^2 + SEveg^

m <- ((coef(msm.fermdairy) - coef(msm.fru))*100)[2]
n <- ((coef(msm.fermdairy) - coef(msm.fru) - qnorm(0.975) * sqrt(SEferm^2 + SEfru^
o <- ((coef(msm.fermdairy) - coef(msm.fru) + qnorm(0.975) * sqrt(SEferm^2 + SEfru^

stroopferm <- c(paste(round(a,3)*100, "% (", round(b,3)*100,"% to", round(c,3)*100
              paste(round(d,3)*100, "% (", round(e,3)*100,"% to", round(f,3)*100,"
              paste(round(g,3)*100, "% (", round(h,3)*100,"% to", round(i,3)*100,"
              paste(round(j,3)*100, "% (", round(k,3)*100,"% to", round(l,3)*100,"
              paste(round(m,3)*100, "% (", round(n,3)*100,"% to", round(o,3)*100,"

a <- ((coef(msm.nonfermdairy) - coef(msm.meat))*100)[2]
```

```
b <- ((coef(msm.nonfermdairy) - coef(msm.meat) - qnorm(0.975) * sqrt(SEnonferm^2 +
c <- ((coef(msm.nonfermdairy) - coef(msm.meat) + qnorm(0.975) * sqrt(SEnonferm^2 +

d <- ((coef(msm.nonfermdairy) - coef(msm.sea))*100)[2]
e <- ((coef(msm.nonfermdairy) - coef(msm.sea) - qnorm(0.975) * sqrt(SEnonferm^2 +
f <- ((coef(msm.nonfermdairy) - coef(msm.sea) + qnorm(0.975) * sqrt(SEnonferm^2 +

g <- ((coef(msm.nonfermdairy) - coef(msm.eggs))*100)[2]
h <- ((coef(msm.nonfermdairy) - coef(msm.eggs) - qnorm(0.975) * sqrt(SEnonferm^2 +
i <- ((coef(msm.nonfermdairy) - coef(msm.eggs) + qnorm(0.975) * sqrt(SEnonferm^2 +

j <- ((coef(msm.nonfermdairy) - coef(msm.veg))*100)[2]
k <- ((coef(msm.nonfermdairy) - coef(msm.veg) - qnorm(0.975) * sqrt(SEnonferm^2 +
l <- ((coef(msm.nonfermdairy) - coef(msm.veg) + qnorm(0.975) * sqrt(SEnonferm^2 +

m <- ((coef(msm.nonfermdairy) - coef(msm.fru))*100)[2]
n <- ((coef(msm.nonfermdairy) - coef(msm.fru) - qnorm(0.975) * sqrt(SEnonferm^2 +
o <- ((coef(msm.nonfermdairy) - coef(msm.fru) + qnorm(0.975) * sqrt(SEnonferm^2 +

stroopnonferm <- c(paste(round(a,3)*100, "% (", round(b,3)*100,"% to", round(c,3)*
              paste(round(d,3)*100, "% (", round(e,3)*100,"% to", round(f,3)*100,"
              paste(round(g,3)*100, "% (", round(h,3)*100,"% to", round(i,3)*100,"
              paste(round(j,3)*100, "% (", round(k,3)*100,"% to", round(l,3)*100,"
              paste(round(m,3)*100, "% (", round(n,3)*100,"% to", round(o,3)*100,"


a <- ((coef(msm.ffdairy) - coef(msm.meat))*100)[2]
b <- ((coef(msm.ffdairy) - coef(msm.meat) - qnorm(0.975) * sqrt(SEff^2 + SEmeat^2)
c <- ((coef(msm.ffdairy) - coef(msm.meat) + qnorm(0.975) * sqrt(SEff^2 + SEmeat^2)

d <- ((coef(msm.ffdairy) - coef(msm.sea))*100)[2]
e <- ((coef(msm.ffdairy) - coef(msm.sea) - qnorm(0.975) * sqrt(SEff^2 + SEsea^2))*
f <- ((coef(msm.ffdairy) - coef(msm.sea) + qnorm(0.975) * sqrt(SEff^2 + SEsea^2))*

g <- ((coef(msm.ffdairy) - coef(msm.eggs))*100)[2]
h <- ((coef(msm.ffdairy) - coef(msm.eggs) - qnorm(0.975) * sqrt(SEff^2 + SEeggs^2)
i <- ((coef(msm.ffdairy) - coef(msm.eggs) + qnorm(0.975) * sqrt(SEff^2 + SEeggs^2)

j <- ((coef(msm.ffdairy) - coef(msm.veg))*100)[2]
k <- ((coef(msm.ffdairy) - coef(msm.veg) - qnorm(0.975) * sqrt(SEff^2 + SEveg^2))*
l <- ((coef(msm.ffdairy) - coef(msm.veg) + qnorm(0.975) * sqrt(SEff^2 + SEveg^2))*

m <- ((coef(msm.ffdairy) - coef(msm.fru))*100)[2]
n <- ((coef(msm.ffdairy) - coef(msm.fru) - qnorm(0.975) * sqrt(SEff^2 + SEfru^2))*
o <- ((coef(msm.ffdairy) - coef(msm.fru) + qnorm(0.975) * sqrt(SEff^2 + SEfru^2))*

stroopfullfat <- c(paste(round(a,3)*100, "% (", round(b,3)*100,"% to", round(c,3)*
              paste(round(d,3)*100, "% (", round(e,3)*100,"% to", round(f,3)*100,"
              paste(round(g,3)*100, "% (", round(h,3)*100,"% to", round(i,3)*100,"
              paste(round(j,3)*100, "% (", round(k,3)*100,"% to", round(l,3)*100,"
              paste(round(m,3)*100, "% (", round(n,3)*100,"% to", round(o,3)*100,"


a <- ((coef(msm.nfdairy) - coef(msm.meat))*100)[2]
b <- ((coef(msm.nfdairy) - coef(msm.meat) - qnorm(0.975) * sqrt(SEnf^2 + SEmeat^2)
c <- ((coef(msm.nfdairy) - coef(msm.meat) + qnorm(0.975) * sqrt(SEnf^2 + SEmeat^2)

d <- ((coef(msm.nfdairy) - coef(msm.sea))*100)[2]
e <- ((coef(msm.nfdairy) - coef(msm.sea) - qnorm(0.975) * sqrt(SEnf^2 + SEsea^2))*
f <- ((coef(msm.nfdairy) - coef(msm.sea) + qnorm(0.975) * sqrt(SEnf^2 + SEsea^2))*

g <- ((coef(msm.nfdairy) - coef(msm.eggs))*100)[2]
h <- ((coef(msm.nfdairy) - coef(msm.eggs) - qnorm(0.975) * sqrt(SEnf^2 + SEeggs^2)
i <- ((coef(msm.nfdairy) - coef(msm.eggs) + qnorm(0.975) * sqrt(SEnf^2 + SEeggs^2)
```

```
j <- ((coef(msm.nfdairy) - coef(msm.veg))*100)[2]
k <- ((coef(msm.nfdairy) - coef(msm.veg) - qnorm(0.975) * sqrt(SEnf^2 + SEveg^2))*
l <- ((coef(msm.nfdairy) - coef(msm.veg) + qnorm(0.975) * sqrt(SEnf^2 + SEveg^2))*

m <- ((coef(msm.nfdairy) - coef(msm.fru))*100)[2]
n <- ((coef(msm.nfdairy) - coef(msm.fru) - qnorm(0.975) * sqrt(SEnf^2 + SEfru^2))*
o <- ((coef(msm.nfdairy) - coef(msm.fru) + qnorm(0.975) * sqrt(SEnf^2 + SEfru^2))*

stroopnonfat <- c(paste(round(a,3)*100, "% (", round(b,3)*100,"% to", round(c,3)*1
              paste(round(d,3)*100, "% (", round(e,3)*100,"% to", round(f,3)*100,"
              paste(round(g,3)*100, "% (", round(h,3)*100,"% to", round(i,3)*100,"
              paste(round(j,3)*100, "% (", round(k,3)*100,"% to", round(l,3)*100,"
              paste(round(m,3)*100, "% (", round(n,3)*100,"% to", round(o,3)*100,"


a <- ((coef(msm.sugdairy) - coef(msm.meat))*100)[2]
b <- ((coef(msm.sugdairy) - coef(msm.meat) - qnorm(0.975) * sqrt(SEsug^2 + SEmeat^
c <- ((coef(msm.sugdairy) - coef(msm.meat) + qnorm(0.975) * sqrt(SEsug^2 + SEmeat^

d <- ((coef(msm.sugdairy) - coef(msm.sea))*100)[2]
e <- ((coef(msm.sugdairy) - coef(msm.sea) - qnorm(0.975) * sqrt(SEsug^2 + SEsea^2)
f <- ((coef(msm.sugdairy) - coef(msm.sea) + qnorm(0.975) * sqrt(SEsug^2 + SEsea^2)

g <- ((coef(msm.sugdairy) - coef(msm.eggs))*100)[2]
h <- ((coef(msm.sugdairy) - coef(msm.eggs) - qnorm(0.975) * sqrt(SEsug^2 + SEeggs^
i <- ((coef(msm.sugdairy) - coef(msm.eggs) + qnorm(0.975) * sqrt(SEsug^2 + SEeggs^

j <- ((coef(msm.sugdairy) - coef(msm.veg))*100)[2]
k <- ((coef(msm.sugdairy) - coef(msm.veg) - qnorm(0.975) * sqrt(SEsug^2 + SEveg^2)
l <- ((coef(msm.sugdairy) - coef(msm.veg) + qnorm(0.975) * sqrt(SEsug^2 + SEveg^2)

m <- ((coef(msm.sugdairy) - coef(msm.fru))*100)[2]
n <- ((coef(msm.sugdairy) - coef(msm.fru) - qnorm(0.975) * sqrt(SEsug^2 + SEfru^2)
o <- ((coef(msm.sugdairy) - coef(msm.fru) + qnorm(0.975) * sqrt(SEsug^2 + SEfru^2)

stroopsug <- c(paste(round(a,3)*100, "% (", round(b,3)*100,"% to", round(c,3)*100,
              paste(round(d,3)*100, "% (", round(e,3)*100,"% to", round(f,3)*100,"
              paste(round(g,3)*100, "% (", round(h,3)*100,"% to", round(i,3)*100,"
              paste(round(j,3)*100, "% (", round(k,3)*100,"% to", round(l,3)*100,"
              paste(round(m,3)*100, "% (", round(n,3)*100,"% to", round(o,3)*100,"

STROOP <- data.frame(strooptotal,stroopferm, stroopnonferm, stroopfullfat, stroopn

STROOP
```

A data.frame: 5 × 6

strooptotal	stroopferm	stroopnonferm	stroopfullfat	stroopnonfat	stroopsug
<chr>	<chr>	<chr>	<chr>	<chr>	<chr>
6.8 % (-7.9 % to 21.5 %)	10 % (-5 % to 25 %)	5.5 % (-9.6 % to 20.5 %)	8.4 % (-6.5 % to 23.2 %)	4.7 % (-11.7 % to 21.1 %)	9.1 % (-6.1 % to 24.3 %)
-9.1 % (-25.2 % to 7 %)	-5.9 % (-22.2 % to 10.4 %)	-10.4 % (-26.8 % to 6 %)	-7.5 % (-23.7 % to 8.7 %)	-11.2 % (-28.8 % to 6.5 %)	-6.8 % (-23.3 % to 9.8 %)
2.8 % (-26.1 % to 31.7 %)	6 % (-23 % to 35 %)	1.5 % (-27.6 % to 30.6 %)	4.4 % (-24.6 % to 33.4 %)	0.8 % (-29 % to 30.5 %)	5.2 % (-24 % to 34.3 %)
-1.4 % (-5.9 % to 3.1 %)	1.8 % (-3.5 % to 7.1 %)	-2.7 % (-8.2 % to 2.8 %)	0.2 % (-4.7 % to 5.1 %)	-3.5 % (-12 % to 5 %)	0.9 % (-4.9 % to 6.8 %)
-0.5 % (-2.8 % to 1.9 %)	2.7 % (-1 % to 6.4 %)	-1.8 % (-5.8 % to 2.1 %)	1.1 % (-2 % to 4.2 %)	-2.6 % (-10.2 % to 5 %)	1.8 % (-2.6 % to 6.3 %)

DO40 (binary)

In [80]:
```
ch_sc$F2do40_cat <- as.numeric(ch_sc$F2do40_cat)

do <- ch_sc %>% filter(!is.na(F2do40_cat))

msm.totdairy <- geeglm(F2do40_cat ~ total_dairy_b, data=do, weights=ipw_totdairy,i
                       corstr="independence")
SEtotal <- coef(summary(msm.totdairy))[,2]

msm.fermdairy <- geeglm(F2do40_cat ~ ferm_dairy_b, data=do, weights=ipw_fermdairy,
                corstr="independence")
SEferm <- coef(summary(msm.fermdairy))[,2]

msm.nonfermdairy <- geeglm(F2do40_cat ~ nonferm_dairy_b, data=do, weights=ipw_nonfe
                corstr="independence")
SEnonferm <- coef(summary(msm.nonfermdairy))[,2]

msm.ffdairy <- geeglm(F2do40_cat ~ fullfat_dairy_b, data=do, weights=ipw_fullfatda
                corstr="independence")
SEff <- coef(summary(msm.ffdairy))[,2]

msm.nfdairy <- geeglm(F2do40_cat ~ nonfat_dairy_b, data=do, weights=ipw_nonfatdair
                corstr="independence")
SEnf <- coef(summary(msm.nfdairy))[,2]

msm.sugdairy <- geeglm(F2do40_cat ~ sugar_dairy_b, data=do, weights=ipw_sugdairy, 
                corstr="independence")
SEsug <- coef(summary(msm.sugdairy))[,2]

msm.meat <- geeglm(F2do40_cat ~ meat_b, data=do, weights=ipw_meat, id=pt,
                corstr="independence")
SEmeat <- coef(summary(msm.meat))[,2]

msm.sea <- geeglm(F2do40_cat ~ sea_b, data=do, weights=ipw_sea, id=pt,
                corstr="independence")
SEsea <- coef(summary(msm.sea))[,2]


msm.veg <- geeglm(F2do40_cat ~ veg_b, data=do, weights=ipw_veg, id=pt,
                corstr="independence")
SEveg <- coef(summary(msm.veg))[,2]
```

```
msm.fru <- geeglm(F2do40_cat ~ fru_b, data=do, weights=ipw_fru, id=pt,
                corstr="independence")
SEfru <- coef(summary(msm.fru))[,2]

msm.eggs <- geeglm(F2do40_cat ~ eggs_b, data=do, weights=ipw_eggs, id=pt,
                corstr="independence")
SEeggs <- coef(summary(msm.eggs))[,2]

a <- ((coef(msm.totdairy) - coef(msm.meat))*100)[2]
b <- ((coef(msm.totdairy) - coef(msm.meat) - qnorm(0.975) * sqrt(SEtotal^2 + SEmea
c <- ((coef(msm.totdairy) - coef(msm.meat) + qnorm(0.975) * sqrt(SEtotal^2 + SEmea

d <- ((coef(msm.totdairy) - coef(msm.sea))*100)[2]
e <- ((coef(msm.totdairy) - coef(msm.sea) - qnorm(0.975) * sqrt(SEtotal^2 + SEsea^
f <- ((coef(msm.totdairy) - coef(msm.sea) + qnorm(0.975) * sqrt(SEtotal^2 + SEsea^

g <- ((coef(msm.totdairy) - coef(msm.eggs))*100)[2]
h <- ((coef(msm.totdairy) - coef(msm.eggs) - qnorm(0.975) * sqrt(SEtotal^2 + SEegg
i <- ((coef(msm.totdairy) - coef(msm.eggs) + qnorm(0.975) * sqrt(SEtotal^2 + SEegg

j <- ((coef(msm.totdairy) - coef(msm.veg))*100)[2]
k <- ((coef(msm.totdairy) - coef(msm.veg) - qnorm(0.975) * sqrt(SEtotal^2 + SEveg^
l <- ((coef(msm.totdairy) - coef(msm.veg) + qnorm(0.975) * sqrt(SEtotal^2 + SEveg^

m <- ((coef(msm.totdairy) - coef(msm.fru))*100)[2]
n <- ((coef(msm.totdairy) - coef(msm.fru) - qnorm(0.975) * sqrt(SEtotal^2 + SEfru^
o <- ((coef(msm.totdairy) - coef(msm.fru) + qnorm(0.975) * sqrt(SEtotal^2 + SEfru^

do40total <- c(paste(round(a,3)*100, "% (", round(b,3)*100,"% to ", round(c,3)*100
              paste(round(d,3)*100, "% (", round(e,3)*100,"% to ", round(f,3)*100,
              paste(round(g,3)*100, "% (", round(h,3)*100,"% to ", round(i,3)*100,
              paste(round(j,3)*100, "% (", round(k,3)*100,"% to ", round(l,3)*100,
              paste(round(m,3)*100, "% (", round(n,3)*100,"% to ", round(o,3)*100,

a <- ((coef(msm.fermdairy) - coef(msm.meat))*100)[2]
b <- ((coef(msm.fermdairy) - coef(msm.meat) - qnorm(0.975) * sqrt(SEferm^2 + SEmea
c <- ((coef(msm.fermdairy) - coef(msm.meat) + qnorm(0.975) * sqrt(SEferm^2 + SEmea

d <- ((coef(msm.fermdairy) - coef(msm.sea))*100)[2]
e <- ((coef(msm.fermdairy) - coef(msm.sea) - qnorm(0.975) * sqrt(SEferm^2 + SEsea^
f <- ((coef(msm.fermdairy) - coef(msm.sea) + qnorm(0.975) * sqrt(SEferm^2 + SEsea^

g <- ((coef(msm.fermdairy) - coef(msm.eggs))*100)[2]
h <- ((coef(msm.fermdairy) - coef(msm.eggs) - qnorm(0.975) * sqrt(SEferm^2 + SEegg
i <- ((coef(msm.fermdairy) - coef(msm.eggs) + qnorm(0.975) * sqrt(SEferm^2 + SEegg

j <- ((coef(msm.fermdairy) - coef(msm.veg))*100)[2]
k <- ((coef(msm.fermdairy) - coef(msm.veg) - qnorm(0.975) * sqrt(SEferm^2 + SEveg^
l <- ((coef(msm.fermdairy) - coef(msm.veg) + qnorm(0.975) * sqrt(SEferm^2 + SEveg^

m <- ((coef(msm.fermdairy) - coef(msm.fru))*100)[2]
n <- ((coef(msm.fermdairy) - coef(msm.fru) - qnorm(0.975) * sqrt(SEferm^2 + SEfru^
o <- ((coef(msm.fermdairy) - coef(msm.fru) + qnorm(0.975) * sqrt(SEferm^2 + SEfru^

do40ferm <- c(paste(round(a,3)*100, "% (", round(b,3)*100,"% to ", round(c,3)*100,
              paste(round(d,3)*100, "% (", round(e,3)*100,"% to ", round(f,3)*100,
              paste(round(g,3)*100, "% (", round(h,3)*100,"% to ", round(i,3)*100,
              paste(round(j,3)*100, "% (", round(k,3)*100,"% to ", round(l,3)*100,
              paste(round(m,4), "% (", round(n,3)*100,"% to ", round(o,3)*100,"%)"

a <- ((coef(msm.nonfermdairy) - coef(msm.meat))*100)[2]
b <- ((coef(msm.nonfermdairy) - coef(msm.meat) - qnorm(0.975) * sqrt(SEnonferm^2 +
c <- ((coef(msm.nonfermdairy) - coef(msm.meat) + qnorm(0.975) * sqrt(SEnonferm^2 +
```

```
d <- ((coef(msm.nonfermdairy) - coef(msm.sea))*100)[2]
e <- ((coef(msm.nonfermdairy) - coef(msm.sea) - qnorm(0.975) * sqrt(SEnonferm^2 + S
f <- ((coef(msm.nonfermdairy) - coef(msm.sea) + qnorm(0.975) * sqrt(SEnonferm^2 + S

g <- ((coef(msm.nonfermdairy) - coef(msm.eggs))*100)[2]
h <- ((coef(msm.nonfermdairy) - coef(msm.eggs) - qnorm(0.975) * sqrt(SEnonferm^2 +
i <- ((coef(msm.nonfermdairy) - coef(msm.eggs) + qnorm(0.975) * sqrt(SEnonferm^2 +

j <- ((coef(msm.nonfermdairy) - coef(msm.veg))*100)[2]
k <- ((coef(msm.nonfermdairy) - coef(msm.veg) - qnorm(0.975) * sqrt(SEnonferm^2 + S
l <- ((coef(msm.nonfermdairy) - coef(msm.veg) + qnorm(0.975) * sqrt(SEnonferm^2 + S

m <- ((coef(msm.nonfermdairy) - coef(msm.fru))*100)[2]
n <- ((coef(msm.nonfermdairy) - coef(msm.fru) - qnorm(0.975) * sqrt(SEnonferm^2 + S
o <- ((coef(msm.nonfermdairy) - coef(msm.fru) + qnorm(0.975) * sqrt(SEnonferm^2 + S

do40nonferm <- c(paste(round(a,3)*100, "% (", round(b,3)*100,"% to ", round(c,3)*10
              paste(round(d,3)*100, "% (", round(e,3)*100,"% to ", round(f,3)*100,
              paste(round(g,3)*100, "% (", round(h,3)*100,"% to ", round(i,3)*100,
              paste(round(j,3)*100, "% (", round(k,3)*100,"% to ", round(l,3)*100,
              paste(round(m,4), "% (", round(n,3)*100,"% to ", round(o,3)*100,"%)"


a <- ((coef(msm.ffdairy) - coef(msm.meat))*100)[2]
b <- ((coef(msm.ffdairy) - coef(msm.meat) - qnorm(0.975) * sqrt(SEff^2 + SEmeat^2)
c <- ((coef(msm.ffdairy) - coef(msm.meat) + qnorm(0.975) * sqrt(SEff^2 + SEmeat^2)

d <- ((coef(msm.ffdairy) - coef(msm.sea))*100)[2]
e <- ((coef(msm.ffdairy) - coef(msm.sea) - qnorm(0.975) * sqrt(SEff^2 + SEsea^2))*1
f <- ((coef(msm.ffdairy) - coef(msm.sea) + qnorm(0.975) * sqrt(SEff^2 + SEsea^2))*1

g <- ((coef(msm.ffdairy) - coef(msm.eggs ))*100)[2]
h <- ((coef(msm.ffdairy) - coef(msm.eggs) - qnorm(0.975) * sqrt(SEff^2 + SEeggs^2)
i <- ((coef(msm.ffdairy) - coef(msm.eggs) + qnorm(0.975) * sqrt(SEff^2 + SEeggs^2)

j <- ((coef(msm.ffdairy) - coef(msm.veg))*100)[2]
k <- ((coef(msm.ffdairy) - coef(msm.veg) - qnorm(0.975) * sqrt(SEff^2 + SEveg^2))*1
l <- ((coef(msm.ffdairy) - coef(msm.veg) + qnorm(0.975) * sqrt(SEff^2 + SEveg^2))*1

m <- ((coef(msm.ffdairy) - coef(msm.fru))*100)[2]
n <- ((coef(msm.ffdairy) - coef(msm.fru) - qnorm(0.975) * sqrt(SEff^2 + SEfru^2))*1
o <- ((coef(msm.ffdairy) - coef(msm.fru) + qnorm(0.975) * sqrt(SEff^2 + SEfru^2))*1

do40fullfat <- c(paste(round(a,3)*100, "% (", round(b,3)*100,"% to ", round(c,3)*10
              paste(round(d,3)*100, "% (", round(e,3)*100,"% to ", round(f,3)*100,
              paste(round(g,3)*100, "% (", round(h,3)*100,"% to ", round(i,3)*100,
              paste(round(j,3)*100, "% (", round(k,3)*100,"% to ", round(l,3)*100,
              paste(round(m,3)*100, "% (", round(n,3)*100,"% to ", round(o,3)*100,


a <- ((coef(msm.nfdairy) - coef(msm.meat))*100)[2]
b <- ((coef(msm.nfdairy) - coef(msm.meat) - qnorm(0.975) * sqrt(SEnf^2 + SEmeat^2)
c <- ((coef(msm.nfdairy) - coef(msm.meat) + qnorm(0.975) * sqrt(SEnf^2 + SEmeat^2)

d <- ((coef(msm.nfdairy) - coef(msm.sea))*100)[2]
e <- ((coef(msm.nfdairy) - coef(msm.sea) - qnorm(0.975) * sqrt(SEnf^2 + SEsea^2))*1
f <- ((coef(msm.nfdairy) - coef(msm.sea) + qnorm(0.975) * sqrt(SEnf^2 + SEsea^2))*1

g <- ((coef(msm.nfdairy) - coef(msm.eggs))*100)[2]
h <- ((coef(msm.nfdairy) - coef(msm.eggs) - qnorm(0.975) * sqrt(SEnf^2 + SEeggs^2)
i <- ((coef(msm.nfdairy) - coef(msm.eggs) + qnorm(0.975) * sqrt(SEnf^2 + SEeggs^2)

j <- ((coef(msm.nfdairy) - coef(msm.veg))*100)[2]
```

```
k <- ((coef(msm.nfdairy) - coef(msm.veg) - qnorm(0.975) * sqrt(SEnf^2 + SEveg^2))*
l <- ((coef(msm.nfdairy) - coef(msm.veg) + qnorm(0.975) * sqrt(SEnf^2 + SEveg^2))*

m <- ((coef(msm.nfdairy) - coef(msm.fru))*100)[2]
n <- ((coef(msm.nfdairy) - coef(msm.fru) - qnorm(0.975) * sqrt(SEnf^2 + SEfru^2))*
o <- ((coef(msm.nfdairy) - coef(msm.fru) + qnorm(0.975) * sqrt(SEnf^2 + SEfru^2))*

do40nonfat <- c(paste(round(a,3)*100, "% (", round(b,3)*100,"% to ", round(c,3)*10
              paste(round(d,3)*100, "% (", round(e,3)*100,"% to ", round(f,3)*100,
              paste(round(g,3)*100, "% (", round(h,3)*100,"% to ", round(i,3)*100,
              paste(round(j,3)*100, "% (", round(k,3)*100,"% to ", round(l,3)*100,
              paste(round(m,3)*100, "% (", round(n,3)*100,"% to ", round(o,3)*100,


a <- ((coef(msm.sugdairy) - coef(msm.meat))*100)[2]
b <- ((coef(msm.sugdairy) - coef(msm.meat) - qnorm(0.975) * sqrt(SEsug^2 + SEmeat^
c <- ((coef(msm.sugdairy) - coef(msm.meat) + qnorm(0.975) * sqrt(SEsug^2 + SEmeat^

d <- ((coef(msm.sugdairy) - coef(msm.sea))*100)[2]
e <- ((coef(msm.sugdairy) - coef(msm.sea) - qnorm(0.975) * sqrt(SEsug^2 + SEsea^2)
f <- ((coef(msm.sugdairy) - coef(msm.sea) + qnorm(0.975) * sqrt(SEsug^2 + SEsea^2)

g <- ((coef(msm.sugdairy) - coef(msm.eggs))*100)[2]
h <- ((coef(msm.sugdairy) - coef(msm.eggs) - qnorm(0.975) * sqrt(SEsug^2 + SEeggs^
i <- ((coef(msm.sugdairy) - coef(msm.eggs) + qnorm(0.975) * sqrt(SEsug^2 + SEeggs^

j <- ((coef(msm.sugdairy) - coef(msm.veg))*100)[2]
k <- ((coef(msm.sugdairy) - coef(msm.veg) - qnorm(0.975) * sqrt(SEsug^2 + SEveg^2)
l <- ((coef(msm.sugdairy) - coef(msm.veg) + qnorm(0.975) * sqrt(SEsug^2 + SEveg^2)

do40sug <- c(meat = paste(round(a,3)*100, "% (", round(b,3)*100,"% to ", round(c,3
              fish = paste(round(d,3)*100, "% (", round(e,3)*100,"% to ", round(f,
              eggs = paste(round(g,3)*100, "% (", round(h,3)*100,"% to ", round(i,
              veg = paste(round(j,3)*100, "% (", round(k,3)*100,"% to ", round(l,3
              fru = paste(round(((coef(msm.sugdairy) - coef(msm.fru))*100)[2],3)*10
                    round(((coef(msm.sugdairy) - coef(msm.fru) - qnorm(0.975) * sq
                    round(((coef(msm.sugdairy) - coef(msm.fru) + qnorm(0.975) * sq

DO40 <- data.frame(do40total,do40ferm, do40nonferm, do40fullfat, do40nonfat, do40su
DO40
```

A data.frame: 5 × 6

	do40total<br><chr>	do40ferm<br><chr>	do40nonferm<br><chr>	do40fullfat<br><chr>	do40nonfat<br><chr>	do40sug<br><chr>
**meat**	0 % (-9.1 % to 9 %)	-1.7 % (-11 % to 7.5 %)	2.4 % (-7.3 % to 12.1 %)	-1.5 % (-10.8 % to 7.8 %)	2.5 % (-9.5 % to 14.4 %)	-0.6 % (-10.3 % to 9 %)
**fish**	11.7 % (-3 % to 26.5 %)	10 % (-4.8 % to 24.9 %)	14.2 % (-1 % to 29.3 %)	10.3 % (-4.6 % to 25.2 %)	14.3 % (-2.4 % to 30.9 %)	11.1 % (-4 % to 26.3 %)
**eggs**	18 % (2.3 % to 33.7 %)	16.3 % (0.5 % to 32.1 %)	20.4 % (4.4 % to 36.5 %)	16.5 % (0.7 % to 32.3 %)	20.5 % (3 % to 38 %)	17.4 % (1.3 % to 33.4 %)
**veg**	0 % (-3.7 % to 3.7 %)	-1.7 % (-5.8 % to 2.5 %)	2.5 % (-2.6 % to 7.5 %)	-1.4 % (-5.6 % to 2.7 %)	2.5 % (-6.1 % to 11.2 %)	-0.6 % (-5.6 % to 4.4 %)
**fru**	-1.7 % (-4.6 % to 1.1 %)	-0.0342 % (-6.9 % to 0 %)	0.0073 % (-3.8 % to 5.2 %)	-3.2 % (-6.6 % to 0.3 %)	0.8 % (-7.5 % to 9.1 %)	-2.3 % (-6.8 % to 2.1 %)

CERAD (binary)

In [81]:

```
ch_sc$F2cerad_cat <- as.numeric(ch_sc$F2CERAD_cat)

cer <- ch_sc %>% filter(!is.na(F2CERAD_cat))

msm.totdairy <- geeglm(F2CERAD_cat ~ total_dairy_b, data=cer, weights=ipw_totdairy
                    corstr="independence")
SEtotal <- coef(summary(msm.totdairy))[,2]

msm.fermdairy <- geeglm(F2CERAD_cat ~ ferm_dairy_b, data=cer, weights=ipw_fermdair
                corstr="independence")
SEferm <- coef(summary(msm.fermdairy))[,2]

msm.nonfermdairy <- geeglm(F2CERAD_cat ~ nonferm_dairy_b, data=cer, weights=ipw_no
                corstr="independence")
SEnonferm <- coef(summary(msm.nonfermdairy))[,2]

msm.ffdairy <- geeglm(F2CERAD_cat ~ fullfat_dairy_b, data=cer, weights=ipw_fullfat
                corstr="independence")
SEff <- coef(summary(msm.ffdairy))[,2]

msm.nfdairy <- geeglm(F2CERAD_cat ~ nonfat_dairy_b, data=cer, weights=ipw_nonfatda
                corstr="independence")
SEnf <- coef(summary(msm.nfdairy))[,2]

msm.sugdairy <- geeglm(F2CERAD_cat ~ sugar_dairy_b, data=cer, weights=ipw_sugdairy
                corstr="independence")
SEsug <- coef(summary(msm.sugdairy))[,2]

msm.meat <- geeglm(F2CERAD_cat ~ meat_b, data=cer, weights=ipw_meat, id=pt,
                corstr="independence")
SEmeat <- coef(summary(msm.meat))[,2]

msm.sea <- geeglm(F2CERAD_cat ~ sea_b, data=cer, weights=ipw_sea, id=pt,
                corstr="independence")
SEsea <- coef(summary(msm.sea))[,2]


msm.veg <- geeglm(F2CERAD_cat ~ veg_b, data=cer, weights=ipw_veg, id=pt,
                corstr="independence")
SEveg <- coef(summary(msm.veg))[,2]


msm.fru <- geeglm(F2CERAD_cat ~ fru_b, data=cer, weights=ipw_fru, id=pt,
                corstr="independence")
SEfru <- coef(summary(msm.fru))[,2]


msm.eggs <- geeglm(F2CERAD_cat ~ eggs_b, data=cer, weights=ipw_eggs, id=pt,
                corstr="independence")
SEeggs <- coef(summary(msm.eggs))[,2]


a <- ((coef(msm.totdairy) - coef(msm.meat))*100)[2]
b <- ((coef(msm.totdairy) - coef(msm.meat) - qnorm(0.975) * sqrt(SEtotal^2 + SEmea
c <- ((coef(msm.totdairy) - coef(msm.meat) + qnorm(0.975) * sqrt(SEtotal^2 + SEmea

d <- ((coef(msm.totdairy) - coef(msm.sea))*100)[2]
e <- ((coef(msm.totdairy) - coef(msm.sea) - qnorm(0.975) * sqrt(SEtotal^2 + SEsea^
f <- ((coef(msm.totdairy) - coef(msm.sea) + qnorm(0.975) * sqrt(SEtotal^2 + SEsea^
```

```
g <- ((coef(msm.totdairy) - coef(msm.eggs))*100)[2]
h <- ((coef(msm.totdairy) - coef(msm.eggs) - qnorm(0.975) * sqrt(SEtotal^2 + SEegg
i <- ((coef(msm.totdairy) - coef(msm.eggs) + qnorm(0.975) * sqrt(SEtotal^2 + SEegg

j <- ((coef(msm.totdairy) - coef(msm.veg))*100)[2]
k <- ((coef(msm.totdairy) - coef(msm.veg) - qnorm(0.975) * sqrt(SEtotal^2 + SEveg^
l <- ((coef(msm.totdairy) - coef(msm.veg) + qnorm(0.975) * sqrt(SEtotal^2 + SEveg^

m <- ((coef(msm.totdairy) - coef(msm.fru))*100)[2]
n <- ((coef(msm.totdairy) - coef(msm.fru) - qnorm(0.975) * sqrt(SEtotal^2 + SEfru^
o <- ((coef(msm.totdairy) - coef(msm.fru) + qnorm(0.975) * sqrt(SEtotal^2 + SEfru^

ceradtotal <- c(paste(round(a,3)*100, "% (", round(b,3)*100,"% to ", round(c,3)*10
              paste(round(d,3)*100, "% (", round(e,3)*100,"% to ", round(f,3)*100,
              paste(round(g,3)*100, "% (", round(h,3)*100,"% to ", round(i,3)*100,
              paste(round(j,3)*100, "% (", round(k,3)*100,"% to ", round(l,3)*100,
              paste(round(m,3)*100, "% (", round(n,3)*100,"% to ", round(o,3)*100,

a <- ((coef(msm.fermdairy) - coef(msm.meat))*100)[2]
b <- ((coef(msm.fermdairy) - coef(msm.meat) - qnorm(0.975) * sqrt(SEferm^2 + SEmea
c <- ((coef(msm.fermdairy) - coef(msm.meat) + qnorm(0.975) * sqrt(SEferm^2 + SEmea

d <- ((coef(msm.fermdairy) - coef(msm.sea))*100)[2]
e <- ((coef(msm.fermdairy) - coef(msm.sea) - qnorm(0.975) * sqrt(SEferm^2 + SEsea^
f <- ((coef(msm.fermdairy) - coef(msm.sea) + qnorm(0.975) * sqrt(SEferm^2 + SEsea^

g <- ((coef(msm.fermdairy) - coef(msm.eggs))*100)[2]
h <- ((coef(msm.fermdairy) - coef(msm.eggs) - qnorm(0.975) * sqrt(SEferm^2 + SEegg
i <- ((coef(msm.fermdairy) - coef(msm.eggs) + qnorm(0.975) * sqrt(SEferm^2 + SEegg

j <- ((coef(msm.fermdairy) - coef(msm.veg))*100)[2]
k <- ((coef(msm.fermdairy) - coef(msm.veg) - qnorm(0.975) * sqrt(SEferm^2 + SEveg^
l <- ((coef(msm.fermdairy) - coef(msm.veg) + qnorm(0.975) * sqrt(SEferm^2 + SEveg^

m <- ((coef(msm.fermdairy) - coef(msm.fru))*100)[2]
n <- ((coef(msm.fermdairy) - coef(msm.fru) - qnorm(0.975) * sqrt(SEferm^2 + SEfru^
o <- ((coef(msm.fermdairy) - coef(msm.fru) + qnorm(0.975) * sqrt(SEferm^2 + SEfru^

ceradferm <- c(paste(round(a,3)*100, "% (", round(b,3)*100,"% to ", round(c,3)*100
              paste(round(d,3)*100, "% (", round(e,3)*100,"% to ", round(f,3)*100,
              paste(round(g,3)*100, "% (", round(h,3)*100,"% to ", round(i,3)*100,
              paste(round(j,3)*100, "% (", round(k,3)*100,"% to ", round(l,3)*100,
              paste(round(m,3)*100, "% (", round(n,3)*100,"% to ", round(o,3)*100,

a <- ((coef(msm.nonfermdairy) - coef(msm.meat))*100)[2]
b <- ((coef(msm.nonfermdairy) - coef(msm.meat) - qnorm(0.975) * sqrt(SEnonferm^2 +
c <- ((coef(msm.nonfermdairy) - coef(msm.meat) + qnorm(0.975) * sqrt(SEnonferm^2 +

d <- ((coef(msm.nonfermdairy) - coef(msm.sea))*100)[2]
e <- ((coef(msm.nonfermdairy) - coef(msm.sea) - qnorm(0.975) * sqrt(SEnonferm^2 +
f <- ((coef(msm.nonfermdairy) - coef(msm.sea) + qnorm(0.975) * sqrt(SEnonferm^2 +

g <- ((coef(msm.nonfermdairy) - coef(msm.eggs))*100)[2]
h <- ((coef(msm.nonfermdairy) - coef(msm.eggs) - qnorm(0.975) * sqrt(SEnonferm^2 +
i <- ((coef(msm.nonfermdairy) - coef(msm.eggs) + qnorm(0.975) * sqrt(SEnonferm^2 +

j <- ((coef(msm.nonfermdairy) - coef(msm.veg))*100)[2]
k <- ((coef(msm.nonfermdairy) - coef(msm.veg) - qnorm(0.975) * sqrt(SEnonferm^2 +
l <- ((coef(msm.nonfermdairy) - coef(msm.veg) + qnorm(0.975) * sqrt(SEnonferm^2 +

m <- ((coef(msm.nonfermdairy) - coef(msm.fru))*100)[2]
n <- ((coef(msm.nonfermdairy) - coef(msm.fru) - qnorm(0.975) * sqrt(SEnonferm^2 +
o <- ((coef(msm.nonfermdairy) - coef(msm.fru) + qnorm(0.975) * sqrt(SEnonferm^2 +
```

```
ceradnonferm <- c(paste(round(a,3)*100, "% (", round(b,3)*100,"% to ", round(c,3)*
              paste(round(d,3)*100, "% (", round(e,3)*100,"% to ", round(f,3)*100,
              paste(round(g,3)*100, "% (", round(h,3)*100,"% to ", round(i,3)*100,
              paste(round(j,3)*100, "% (", round(k,3)*100,"% to ", round(l,3)*100,
              paste(round(m,3)*100, "% (", round(n,3)*100,"% to ", round(o,3)*100,


a <- ((coef(msm.ffdairy) - coef(msm.meat))*100)[2]
b <- ((coef(msm.ffdairy) - coef(msm.meat) - qnorm(0.975) * sqrt(SEff^2 + SEmeat^2)
c <- ((coef(msm.ffdairy) - coef(msm.meat) + qnorm(0.975) * sqrt(SEff^2 + SEmeat^2)

d <- ((coef(msm.ffdairy) - coef(msm.sea))*100)[2]
e <- ((coef(msm.ffdairy) - coef(msm.sea) - qnorm(0.975) * sqrt(SEff^2 + SEsea^2))*
f <- ((coef(msm.ffdairy) - coef(msm.sea) + qnorm(0.975) * sqrt(SEff^2 + SEsea^2))*

g <- ((coef(msm.ffdairy) - coef(msm.eggs))*100)[2]
h <- ((coef(msm.ffdairy) - coef(msm.eggs) - qnorm(0.975) * sqrt(SEff^2 + SEeggs^2)
i <- ((coef(msm.ffdairy) - coef(msm.eggs) + qnorm(0.975) * sqrt(SEff^2 + SEeggs^2)

j <- ((coef(msm.ffdairy) - coef(msm.veg))*100)[2]
k <- ((coef(msm.ffdairy) - coef(msm.veg) - qnorm(0.975) * sqrt(SEff^2 + SEveg^2))*
l <- ((coef(msm.ffdairy) - coef(msm.veg) + qnorm(0.975) * sqrt(SEff^2 + SEveg^2))*

m <- ((coef(msm.ffdairy) - coef(msm.fru))*100)[2]
n <- ((coef(msm.ffdairy) - coef(msm.fru) - qnorm(0.975) * sqrt(SEff^2 + SEfru^2))*
o <- ((coef(msm.ffdairy) - coef(msm.fru) + qnorm(0.975) * sqrt(SEff^2 + SEfru^2))*

ceradfullfat <- c(paste(round(a,3)*100, "% (", round(b,3)*100,"% to ", round(c,3)*
              paste(round(d,3)*100, "% (", round(e,3)*100,"% to ", round(f,3)*100,
              paste(round(g,3)*100, "% (", round(h,3)*100,"% to ", round(i,3)*100,
              paste(round(j,3)*100, "% (", round(k,3)*100,"% to ", round(l,3)*100,
              paste(round(m,3)*100, "% (", round(n,3)*100,"% to ", round(o,3)*100,


a <- ((coef(msm.nfdairy) - coef(msm.meat))*100)[2]
b <- ((coef(msm.nfdairy) - coef(msm.meat) - qnorm(0.975) * sqrt(SEnf^2 + SEmeat^2)
c <- ((coef(msm.nfdairy) - coef(msm.meat) + qnorm(0.975) * sqrt(SEnf^2 + SEmeat^2)

d <- ((coef(msm.nfdairy) - coef(msm.sea))*100)[2]
e <- ((coef(msm.nfdairy) - coef(msm.sea) - qnorm(0.975) * sqrt(SEnf^2 + SEsea^2))*
f <- ((coef(msm.nfdairy) - coef(msm.sea) + qnorm(0.975) * sqrt(SEnf^2 + SEsea^2))*

g <- ((coef(msm.nfdairy) - coef(msm.eggs))*100)[2]
h <- ((coef(msm.nfdairy) - coef(msm.eggs) - qnorm(0.975) * sqrt(SEnf^2 + SEeggs^2)
i <- ((coef(msm.nfdairy) - coef(msm.eggs) + qnorm(0.975) * sqrt(SEnf^2 + SEeggs^2)

j <- ((coef(msm.nfdairy) - coef(msm.veg))*100)[2]
k <- ((coef(msm.nfdairy) - coef(msm.veg) - qnorm(0.975) * sqrt(SEnf^2 + SEveg^2))*
l <- ((coef(msm.nfdairy) - coef(msm.veg) + qnorm(0.975) * sqrt(SEnf^2 + SEveg^2))*

m <- ((coef(msm.nfdairy) - coef(msm.fru))*100)[2]
n <- ((coef(msm.nfdairy) - coef(msm.fru) - qnorm(0.975) * sqrt(SEnf^2 + SEfru^2))*
o <- ((coef(msm.nfdairy) - coef(msm.fru) + qnorm(0.975) * sqrt(SEnf^2 + SEfru^2))*

ceradnonfat <- c(paste(round(a,3)*100, "% (", round(b,3)*100,"% to ", round(c,3)*1
              paste(round(d,3)*100, "% (", round(e,3)*100,"% to ", round(f,3)*100,
              paste(round(g,3)*100, "% (", round(h,3)*100,"% to ", round(i,3)*100,
              paste(round(j,3)*100, "% (", round(k,3)*100,"% to ", round(l,3)*100,
              paste(round(m,3)*100, "% (", round(n,3)*100,"% to ", round(o,3)*100,


a <- ((coef(msm.sugdairy) - coef(msm.meat))*100)[2]
b <- ((coef(msm.sugdairy) - coef(msm.meat) - qnorm(0.975) * sqrt(SEsug^2 + SEmeat^
c <- ((coef(msm.sugdairy) - coef(msm.meat) + qnorm(0.975) * sqrt(SEsug^2 + SEmeat^
```

```
d <- ((coef(msm.sugdairy) - coef(msm.sea))*100)[2]
e <- ((coef(msm.sugdairy) - coef(msm.sea) - qnorm(0.975) * sqrt(SEsug^2 + SEsea^2)
f <- ((coef(msm.sugdairy) - coef(msm.sea) + qnorm(0.975) * sqrt(SEsug^2 + SEsea^2)

g <- ((coef(msm.sugdairy) - coef(msm.eggs))*100)[2]
h <- ((coef(msm.sugdairy) - coef(msm.eggs) - qnorm(0.975) * sqrt(SEsug^2 + SEeggs^
i <- ((coef(msm.sugdairy) - coef(msm.eggs) + qnorm(0.975) * sqrt(SEsug^2 + SEeggs^

j <- ((coef(msm.sugdairy) - coef(msm.veg))*100)[2]
k <- ((coef(msm.sugdairy) - coef(msm.veg) - qnorm(0.975) * sqrt(SEsug^2 + SEveg^2)
l <- ((coef(msm.sugdairy) - coef(msm.veg) + qnorm(0.975) * sqrt(SEsug^2 + SEveg^2)

m <- ((coef(msm.sugdairy) - coef(msm.fru))*100)[2]
n <- ((coef(msm.sugdairy) - coef(msm.fru) - qnorm(0.975) * sqrt(SEsug^2 + SEfru^2)
o <- ((coef(msm.sugdairy) - coef(msm.fru) + qnorm(0.975) * sqrt(SEsug^2 + SEfru^2)

ceradsug <- c(paste(round(a,3)*100, "% (", round(b,3)*100,"% to ", round(c,3)*100,
              paste(round(d,3)*100, "% (", round(e,3)*100,"% to ", round(f,3)*100,
              paste(round(g,3)*100, "% (", round(h,3)*100,"% to ", round(i,3)*100,
              paste(round(j,3)*100, "% (", round(k,3)*100,"% to ", round(l,3)*100,
              paste(round(m,3)*100, "% (", round(n,3)*100,"% to ", round(o,3)*100,

CERAD <- data.frame(ceradtotal,ceradferm, ceradnonferm, ceradfullfat, ceradnonfat,
CERAD
```

A data.frame: 5 × 6

ceradtotal	ceradferm	ceradnonferm	ceradfullfat	ceradnonfat	ceradsug
<chr>	<chr>	<chr>	<chr>	<chr>	<chr>
4.9 % (-7.2 % to 17 %)	4.8 % (-7.7 % to 17.2 %)	4.9 % (-7.7 % to 17.4 %)	3.2 % (-9.1 % to 15.5 %)	-1.7 % (-15.7 % to 12.3 %)	3.5 % (-9.1 % to 16.1 %)
0.7 % (-16.1 % to 17.5 %)	0.5 % (-16.5 % to 17.6 %)	0.6 % (-16.5 % to 17.8 %)	-1 % (-18 % to 15.9 %)	-5.9 % (-24.1 % to 12.3 %)	-0.7 % (-17.9 % to 16.4 %)
-0.7 % (-33.7 % to 32.3 %)	-0.9 % (-34 % to 32.3 %)	-0.8 % (-34 % to 32.4 %)	-2.4 % (-35.5 % to 30.7 %)	-7.3 % (-41.1 % to 26.4 %)	-2.1 % (-35.3 % to 31.1 %)
-1.5 % (-5.2 % to 2.2 %)	-1.7 % (-6.4 % to 3 %)	-1.6 % (-6.5 % to 3.4 %)	-3.2 % (-7.5 % to 1.1 %)	-8.1 % (-16 % to -0.3 %)	-2.9 % (-8 % to 2.2 %)
0.5 % (-2.2 % to 3.1 %)	0.3 % (-3.6 % to 4.3 %)	0.4 % (-3.7 % to 4.6 %)	-1.2 % (-4.6 % to 2.2 %)	-6.2 % (-13.6 % to 1.3 %)	-0.9 % (-5.3 % to 3.4 %)

In [82]:
```
pro <- data.frame(CDR, SCD, MEMORY, VERBAL, STROOP,DO40, CERAD)
write.csv(pro, "substitutioneffects.csv")
```

7. Sensitivity analysis for substitution effects

Subset of participants and exposure calculation

In [83]:
```
ch_sensi <- ch_sc %>%
        filter(futime_F2 > 3)%>%#baseline dietary assessment at least 3 years befo
        mutate(totalcal_avg = (totalcal_b + F2totalcal)/2,
                veg_avg = (F2veg+veg_b)/2,
                fru_avg  = (F2fru +fru_b )/2,
                sea_avg  = (F2sea +sea_b )/2,
                meat_avg  = (F2meat +meat_b )/2,
                eggs_avg  = (F2eggs +eggs_b)/2,
                grains_avg  = (F2grains +grains_b )/2,
```

```
                alcohol_avg = (F2alcohol+alcohol_b)/2,
                sugary_avg  = (F2sugary +sugary_b )/2,
                fats_avg = (F2fats+fats_b)/2,
                total_dairy_avg  = (F2total_dairy +total_dairy_b) /2,
                ferm_dairy_avg  = (F2ferm_dairy +ferm_dairy_b) /2,
                nonferm_dairy_avg  = (F2nonferm_dairy +nonferm_dairy_b) /2,
                fullfat_dairy_avg  = (F2fullfat_dairy +fullfat_dairy_b) /2,
                nonfat_dairy_avg = (F2nonfat_dairy+nonfat_dairy_b)/2,
                sugar_dairy_avg = (F2sugar_dairy+sugar_dairy_b)/2,
              nonsug_dairy_avg = (F2nonsug_dairy+nonsug_dairy_b)/2)%>%
        filter(!is.na(total_dairy_avg)& !is.na(meat_avg) & !is.na(sea_avg)& !is.na
               & !is.na(fru_avg) & totalcal_avg > 500 & totalcal_avg < 4200)

ch_sensi #n= 884
```

A

pt	sex	datbirth	datexam	bthpl_dem	ethori_self	edtyp	edlv	mrtsts	sclhlp	⋯	veg_
<int>	<int>	<date>	<date>	<chr>	<chr>	<int>	<int>	<int>	<int>	⋯	<d
6	0	1943-01-30	2003-07-08	SZ	W	1	12	3	0	⋯	202.4
7	0	1933-03-26	2003-07-04	EY	W	0	8	0	1	⋯	186.6
20	0	1943-05-26	2003-07-16	SZ	W	3	14	2	0	⋯	150.7
24	1	1936-09-16	2003-07-14	PD	W	3	50	2	1	⋯	119.7
25	0	1943-12-28	2003-08-28	SZ	W	1	12	3	0	⋯	180.3
31	0	1948-03-10	2003-07-23	EG	W	2	24	2	0	⋯	260.8
46	0	1942-11-07	2003-07-31	SZ	W	0	10	0	0	⋯	138.5
48	1	1947-05-05	2003-08-13	SZ	W	2	16	2	0	⋯	127.3
51	0	1948-10-16	2003-09-01	SZ	W	1	14	NA	0	⋯	222.3
56	1	1939-01-01	2003-08-04	SZ	W	3	12	0	1	⋯	140.1
68	0	1940-07-10	2003-08-05	SZ	W	0	10	3	0	⋯	121.5
72	0	1941-06-09	2003-08-08	RM	W	2	18	0	0	⋯	170.5
87	1	1943-04-30	2003-08-12	SZ	W	3	15	0	0	⋯	223.0
103	0	1946-02-04	2003-08-19	SZ	W	3	15	0	0	⋯	295.8
112	1	1929-11-24	2003-09-02	SZ	W	0	10	0	1	⋯	128.0
121	1	1945-07-17	2003-08-25	FR	W	1	13	0	0	⋯	161.8
130	1	1943-09-06	2003-08-26	CT	X	1	12	2	1	⋯	218.1
144	0	1948-09-25	2003-09-19	SZ	W	3	16	0	0	⋯	371.3
157	0	1943-05-06	2003-09-12	SZ	W	1	10	2	1	⋯	117.4
167	1	1944-02-25	2003-09-17	SZ	W	2	18	0	0	⋯	162.2
170	1	1940-06-23	2003-09-04	SZ	W	2	19	0	0	⋯	194.4
175	0	1949-03-03	2003-10-08	SZ	W	1	12	2	0	⋯	486.6

pt	sex	datbirth	datexam	bthpl_dem	ethori_self	edtyp	edlv	mrtsts	sclhlp	⋯	veg_
<int>	<int>	<date>	<date>	<chr>	<chr>	<int>	<int>	<int>	<int>	⋯	<d
182	0	1943-07-12	2003-09-02	FR	W	2	14	0	0	⋯	182.2
186	0	1942-05-02	2003-09-01	SZ	W	1	12	0	0	⋯	193.3
191	0	1945-02-03	2003-09-19	HL	W	3	15	0	0	⋯	193.7
203	1	1945-03-31	2003-09-08	SZ	W	3	14	2	0	⋯	587.5
206	0	1943-09-30	2003-09-19	SZ	W	0	9	0	0	⋯	187.0
208	0	1941-05-04	2003-09-23	SZ	W	0	9	0	0	⋯	249.6
213	0	1940-10-01	2003-10-29	SZ	W	2	16	0	0	⋯	252.9
223	1	1938-09-21	2003-10-07	SZ	W	3	15	0	1	⋯	106.5
⋮	⋮	⋮	⋮	⋮	⋮	⋮	⋮	⋮	⋮	⋱	
6607	0	1945-12-02	2006-03-24	SZ	W	1	12	0	0	⋯	143.52
6611	0	1941-06-10	2006-03-16	IT	W	0	9	0	1	⋯	282.67
6631	1	1944-09-04	2006-04-04	SZ	W	1	11	2	0	⋯	93.17
6632	0	1932-11-07	2006-04-03	SZ	W	2	15	0	0	⋯	173.04
6650	1	1950-04-12	2006-04-28	IT	W	4	17	0	0	⋯	195.13
6661	0	1946-08-27	2006-04-07	FR	W	2	14	NA	0	⋯	230.93
6662	1	1950-11-05	2006-04-06	SZ	W	1	13	NA	0	⋯	164.24
6686	1	1949-12-07	2006-04-19	IT	W	1	12	0	0	⋯	140.35
6746	0	1943-12-13	2006-04-26	SZ	W	1	12	NA	0	⋯	199.10
9029	0	1943-12-31	2003-10-18	PR	O	4	14	NA	0	⋯	156.87
9056	0	1949-05-18	2003-12-03	TL	A	4	14	2	0	⋯	145.66
9094	1	1948-12-04	2004-02-03	BV	O	4	18	0	0	⋯	388.03
9179	0	1948-07-07	2004-07-15	CI	O	0	13	2	0	⋯	89.82

pt	sex	datbirth	datexam	bthpl_dem	ethori_self	edtyp	edlv	mrtsts	sclhlp	⋯	veg_
<int>	<int>	<date>	<date>	<chr>	<chr>	<int>	<int>	<int>	<int>	⋯	<d
9208	1	1940-07-25	2004-08-23	CI	O	3	15	0	0	⋯	427.85
9237	0	1938-05-02	2004-09-16	TY	O	1	12	0	0	⋯	89.10
9269	1	1944-03-22	2004-10-29	EY	B	4	21	0	0	⋯	366.96
9292	0	1939-07-17	2005-02-02	CO	O	0	12	0	1	⋯	274.86
9366	1	1946-08-20	2005-03-31	CI	O	4	25	0	0	⋯	298.98
9397	0	1949-11-11	2005-05-04	MY	A	3	17	0	0	⋯	401.60
9413	0	1947-01-13	2005-05-18	X	O	0	8	0	0	⋯	415.08
9429	1	1947-11-02	2005-06-06	X	O	3	18	0	0	⋯	127.27
9448	0	1941-04-05	2005-07-25	X	O	2	3	3	0	⋯	287.18
9490	1	1941-05-01	2005-09-13	TY	A	4	12	0	0	⋯	601.78
9494	0	1950-09-29	2005-09-12	PS	A	4	17	0	0	⋯	123.39
9520	0	1947-09-01	2005-11-01	TY	A	3	14	0	1	⋯	407.58
9549	0	1948-06-21	2005-12-06	HT	O	2	18	NA	0	⋯	220.17
9551	1	1940-10-23	2005-12-06	TY	A	4	17	0	1	⋯	744.10
9565	0	1941-07-09	2006-01-12	IR	A	4	17	0	1	⋯	243.43
9569	0	1944-06-18	2006-02-16	TU	B	0	9	2	1	⋯	51.65
9592	0	1943-[illegible]	2006-01-[illegible]	IR	A	1	12	2	1	⋯	272.85

In [84]:

```
#######################################
#########TOTAL DAIRY#################
#######################################

weight.model = weightit(formula("total_dairy_avg ~ veg_avg+fru_avg+sea_avg+meat_av
               grains_avg+alcohol_avg+sugary_avg+fats_avg+sex+
                 age_cat+edu+sm_b+HTA_b+depre_b+pa_b+cvevent_b+famincome_b+diab_b+
                        method = "ps", use.kernel=T)

#check weights
summary(weight.model$weights)

#add weights to data
```

```
ch_sensi$ipw_totdairy <- weight.model$weights

#####################################
##########FERMENTED DAIRY############
#####################################
weight.model = weightit(formula("ferm_dairy_avg ~ nonferm_dairy_avg+veg_avg+fru_av
               grains_avg+alcohol_avg+sugary_avg+fats_avg+sex+
                 age_cat+edu+sm_b+HTA_b+depre_b+pa_b+cvevent_b+famincome_b+diab_b+
                        method = "ps", use.kernel=T)

#check weights
summary(weight.model$weights)

#add weights to data
ch_sensi$ipw_fermdairy <- weight.model$weights

#####################################
##########NON FERMENTED DAIRY########
#####################################
weight.model = weightit(formula("nonferm_dairy_avg ~ ferm_dairy_avg+veg_avg+fru_av
               grains_avg+alcohol_avg+sugary_avg+fats_avg+sex+
                 age_cat+edu+sm_b+HTA_b+depre_b+pa_b+cvevent_b+famincome_b+diab_b+
                        method = "ps", use.kernel=T)

#check weights
summary(weight.model$weights)

#add weights to data
ch_sensi$ipw_nonfermdairy <- weight.model$weights

#####################################
##########FULL FAT DAIRY#############
#####################################
weight.model = weightit(formula("fullfat_dairy_avg ~ nonfat_dairy_avg+veg_avg+fru_
               grains_avg+alcohol_avg+sugary_avg+fats_avg+sex+
                 age_cat+edu+sm_b+HTA_b+depre_b+pa_b+cvevent_b+famincome_b+diab_b+
                        method = "ps", use.kernel=T)

#check weights
summary(weight.model$weights)

#add weights to data
ch_sensi$ipw_fullfatdairy <- weight.model$weights

#####################################
###########NON FAT DAIRY#############
#####################################

weight.model = weightit(formula("nonferm_dairy_avg ~ ferm_dairy_avg+veg_avg+fru_av
               grains_avg+alcohol_avg+sugary_avg+fats_avg+sex+
                 age_cat+edu+sm_b+HTA_b+depre_b+pa_b+cvevent_b+famincome_b+diab_b+
                        method = "ps", use.kernel=T)

#check weights
summary(weight.model$weights)

#add weights to data
ch_sensi$ipw_nonfatdairy <- weight.model$weights

#####################################
############SUGARY DAIRY#############
#####################################
weight.model = weightit(formula("sugar_dairy_avg ~ nonsug_dairy_avg+veg_avg+fru_av
               grains_avg+alcohol_avg+sugary_avg+fats_avg+sex+
```

```
                 age_cat+edu+sm_b+HTA_b+depre_b+pa_b+cvevent_b+famincome_b+diab_b+c
                        method = "ps", use.kernel=T)

#check weights
summary(weight.model$weights)

#add weights to data
ch_sensi$ipw_sugdairy <- weight.model$weights

#####################################
################ MEAT ###############
#####################################
weight.model = weightit(formula("meat_avg ~ veg_avg+fru_avg+sea_avg+total_dairy_avg
               grains_avg+alcohol_avg+sugary_avg+fats_avg+sex+
                 age_cat+edu+sm_b+HTA_b+depre_b+pa_b+cvevent_b+famincome_b+diab_b+c
                        method = "ps", use.kernel=T)

#check weights
summary(weight.model$weights)

#add weights to data
ch_sensi$ipw_meat <- weight.model$weights
#####################################
############### FISH ################
#####################################
weight.model = weightit(formula("sea_avg ~ veg_avg+fru_avg+meat_avg+total_dairy_avg
               grains_avg+alcohol_avg+sugary_avg+fats_avg+sex+
                 age_cat+edu+sm_b+HTA_b+depre_b+pa_b+cvevent_b+famincome_b+diab_b+c
                        method = "ps", use.kernel=T)

#check weights
summary(weight.model$weights)

#add weights to data
ch_sensi$ipw_sea <- weight.model$weights

#####################################
################EGGS#################
#####################################
weight.model = weightit(formula("eggs_avg ~ veg_avg+fru_avg+sea_avg+total_dairy_avg
               grains_avg+alcohol_avg+sugary_avg+fats_avg+sex+
                 age_cat+edu+sm_b+HTA_b+depre_b+pa_b+cvevent_b+famincome_b+diab_b+c
                        method = "ps", use.kernel=T)

#check weights
summary(weight.model$weights)

#add weights to data
ch_sensi$ipw_eggs <- weight.model$weights

#####################################
############VEGETABLES##############
#####################################
weight.model = weightit(formula(" veg_avg ~ meat_avg+fru_avg+sea_avg+total_dairy_av
               grains_avg+alcohol_avg+sugary_avg+fats_avg+sex+
                 age_cat+edu+sm_b+HTA_b+depre_b+pa_b+cvevent_b+famincome_b+diab_b+c
                        method = "ps", use.kernel=T)

#check weights
summary(weight.model$weights)

#add weights to data
ch_sensi$ipw_veg <- weight.model$weights
```

```
########################################
##############FRUITS###############
########################################
weight.model = weightit(formula(" sea_avg ~ veg_avg+fru_avg+total_dairy_avg+meat_av
               grains_avg+alcohol_avg+sugary_avg+fats_avg+sex+
                 age_cat+edu+sm_b+HTA_b+depre_b+pa_b+cvevent_b+famincome_b+diab_b+c
                        method = "ps", use.kernel=T)

#check weights
summary(weight.model$weights)

#add weights to data
ch_sensi$ipw_fru <- weight.model$weights
```

```
   Min. 1st Qu.  Median    Mean 3rd Qu.    Max. 
 0.2096  0.8252  0.9635  1.1411  1.1101 42.5159 
   Min. 1st Qu.  Median    Mean 3rd Qu.    Max. 
 0.2010  0.8518  0.9524  1.1232  1.0955 13.3998 
    Min.   1st Qu.    Median      Mean   3rd Qu.      Max. 
  0.3145    0.8869    1.1035    1.5025    1.2939  142.6740 
   Min. 1st Qu.  Median    Mean 3rd Qu.    Max. 
 0.2459  0.8164  0.9652  1.1443  1.1084 28.1483 
    Min.   1st Qu.    Median      Mean   3rd Qu.      Max. 
  0.3145    0.8869    1.1035    1.5025    1.2939  142.6740 
   Min. 1st Qu.  Median    Mean 3rd Qu.    Max. 
 0.3751  0.8871  1.0728  1.4403  1.2614 98.2858 
    Min.  1st Qu.   Median     Mean  3rd Qu.     Max. 
 0.05556  0.68264  0.86526  1.09065  1.06288 30.05315 
    Min.  1st Qu.   Median     Mean  3rd Qu.     Max. 
 0.07549  0.78625  0.90937  1.29610  1.12666 37.66408 
    Min.  1st Qu.   Median     Mean  3rd Qu.     Max. 
 0.06202  0.89198  0.99136  1.21857  1.16450 32.11392 
    Min.  1st Qu.   Median     Mean  3rd Qu.     Max. 
 0.06972  0.68283  0.86476  1.18106  1.10264 49.27692 
    Min.  1st Qu.   Median     Mean  3rd Qu.     Max. 
 0.07549  0.78625  0.90937  1.29610  1.12666 37.66408 
```

In [85]:

```
threshold_99a <- quantile(ch_sensi$ipw_totdairy, 0.995)
ch_sensi$ipw_totdairy[ch_sensi$ipw_totdairy > threshold_99a] <- threshold_99a

threshold_99b <- quantile(ch_sensi$ipw_fermdairy, 0.995)
ch_sensi$ipw_fermdairy[ch_sensi$ipw_fermdairy > threshold_99b] <- threshold_99b

threshold_99c <- quantile(ch_sensi$ipw_nonfermdairy, 0.995)
ch_sensi$ipw_nonfermdairy[ch_sensi$ipw_nonfermdairy > threshold_99c] <- threshold_9

threshold_99d <- quantile(ch_sensi$ipw_fullfatdairy, 0.995)
ch_sensi$ipw_fullfatdairy[ch_sensi$ipw_fullfatdairy > threshold_99d] <- threshold_9

threshold_99e <- quantile(ch_sensi$ipw_nonfatdairy, 0.995)
ch_sensi$ipw_nonfatdairy[ch_sensi$ipw_nonfatdairy > threshold_99e] <- threshold_99

threshold_99f <- quantile(ch_sensi$ipw_sugdairy, 0.995)
ch_sensi$ipw_sugdairy[ch_sensi$ipw_sugdairy > threshold_99f] <- threshold_99f

threshold_99g <- quantile(ch_sensi$ipw_meat, 0.995)
ch_sensi$ipw_meat[ch_sensi$ipw_meat > threshold_99g] <- threshold_99g

threshold_99m <- quantile(ch_sensi$ipw_sea, 0.995)
ch_sensi$ipw_sea[ch_sensi$ipw_sea > threshold_99m] <- threshold_99m

threshold_99n <- quantile(ch_sensi$ipw_veg, 0.995)
ch_sensi$ipw_veg[ch_sensi$ipw_veg > threshold_99n] <- threshold_99n
```

```
threshold_99o <- quantile(ch_sensi$ipw_fru, 0.995)
ch_sensi$ipw_fru[ch_sensi$ipw_fru > threshold_99o] <- threshold_99o

threshold_99p <- quantile(ch_sensi$ipw_eggs, 0.995)
ch_sensi$ipw_eggs[ch_sensi$ipw_eggs > threshold_99p] <- threshold_99p
```

Computing ATEs for relative effects

CDR

In [86]:

```
ch_sensi$F3CDR <- as.numeric(ifelse(ch_sensi$F3CDR == "0.0", 0,
                         ifelse(ch_sensi$F3CDR == "0.5", 1, NA)))

cdr <- ch_sensi %>% filter(!is.na(F3CDR))

msm.totdairy <- geeglm(F3CDR ~ total_dairy_avg, data=cdr, weights=ipw_totdairy,id=
                       corstr="independence")
SEtotal <- coef(summary(msm.totdairy))[,2]

msm.fermdairy <- geeglm(F3CDR ~ ferm_dairy_avg, data=cdr, weights=ipw_fermdairy, i
                 corstr="independence")
SEferm <- coef(summary(msm.fermdairy))[,2]

msm.nonfermdairy <- geeglm(F3CDR ~ nonferm_dairy_avg, data=cdr, weights=ipw_nonfer
                 corstr="independence")
SEnonferm <- coef(summary(msm.nonfermdairy))[,2]

msm.ffdairy <- geeglm(F3CDR ~ fullfat_dairy_avg, data=cdr, weights=ipw_fullfatdair
                 corstr="independence")
SEff <- coef(summary(msm.ffdairy))[,2]

msm.nfdairy <- geeglm(F3CDR ~ nonfat_dairy_avg, data=cdr, weights=ipw_nonfatdairy,
                 corstr="independence")
SEnf <- coef(summary(msm.nfdairy))[,2]

msm.sugdairy <- geeglm(F3CDR ~ sugar_dairy_avg, data=cdr, weights=ipw_sugdairy, id
                 corstr="independence")
SEsug <- coef(summary(msm.sugdairy))[,2]

msm.meat <- geeglm(F3CDR ~ meat_avg, data=cdr, weights=ipw_meat, id=pt,
                 corstr="independence")
SEmeat <- coef(summary(msm.meat))[,2]

msm.sea <- geeglm(F3CDR ~ sea_avg, data=cdr, weights=ipw_sea, id=pt,
                 corstr="independence")
SEsea <- coef(summary(msm.sea))[,2]


msm.veg <- geeglm(F3CDR ~ veg_avg, data=cdr, weights=ipw_veg, id=pt,
                 corstr="independence")
SEveg <- coef(summary(msm.veg))[,2]

msm.fru <- geeglm(F3CDR ~ fru_avg, data=cdr, weights=ipw_fru, id=pt,
                 corstr="independence")
SEfru <- coef(summary(msm.fru))[,2]


msm.eggs <- geeglm(F3CDR ~ eggs_avg, data=cdr, weights=ipw_eggs, id=pt,
                 corstr="independence")
SEeggs <- coef(summary(msm.eggs))[,2]
```

```
a <- ((coef(msm.totdairy) - coef(msm.meat))*100)[2]
b <- ((coef(msm.totdairy) - coef(msm.meat) - qnorm(0.975) * sqrt(SEtotal^2 + SEmea
c <- ((coef(msm.totdairy) - coef(msm.meat) + qnorm(0.975) * sqrt(SEtotal^2 + SEmea

d <- ((coef(msm.totdairy) - coef(msm.sea))*100)[2]
e <- ((coef(msm.totdairy) - coef(msm.sea) - qnorm(0.975) * sqrt(SEtotal^2 + SEsea^
f <- ((coef(msm.totdairy) - coef(msm.sea) + qnorm(0.975) * sqrt(SEtotal^2 + SEsea^

g <- ((coef(msm.totdairy) - coef(msm.eggs))*100)[2]
h <- ((coef(msm.totdairy) - coef(msm.eggs) - qnorm(0.975) * sqrt(SEtotal^2 + SEegg
i <- ((coef(msm.totdairy) - coef(msm.eggs) + qnorm(0.975) * sqrt(SEtotal^2 + SEegg

j <- ((coef(msm.totdairy) - coef(msm.veg))*100)[2]
k <- ((coef(msm.totdairy) - coef(msm.veg) - qnorm(0.975) * sqrt(SEtotal^2 + SEveg^
l <- ((coef(msm.totdairy) - coef(msm.veg) + qnorm(0.975) * sqrt(SEtotal^2 + SEveg^

m <- ((coef(msm.totdairy) - coef(msm.fru))*100)[2]
n <- ((coef(msm.totdairy) - coef(msm.fru) - qnorm(0.975) * sqrt(SEtotal^2 + SEfru^
o <- ((coef(msm.totdairy) - coef(msm.fru) + qnorm(0.975) * sqrt(SEtotal^2 + SEfru^

cdrtotal <- c(paste(round(a,3)*100, "% (", round(b,3)*100,"% to ", round(c,3)*100,
              paste(round(d,3)*100, "% (", round(e,3)*100,"% to ", round(f,3)*100,
              paste(round(g,3)*100, "% (", round(h,3)*100,"% to ", round(i,3)*100,
              paste(round(j,3)*100, "% (", round(k,3)*100,"% to ", round(l,3)*100,
              paste(round(m,3)*100, "% (", round(n,3)*100,"% to ", round(o,3)*100,

a <- ((coef(msm.fermdairy) - coef(msm.meat))*100)[2]
b <- ((coef(msm.fermdairy) - coef(msm.meat) - qnorm(0.975) * sqrt(SEferm^2 + SEmea
c <- ((coef(msm.fermdairy) - coef(msm.meat) + qnorm(0.975) * sqrt(SEferm^2 + SEmea

d <- ((coef(msm.fermdairy) - coef(msm.sea))*100)[2]
e <- ((coef(msm.fermdairy) - coef(msm.sea) - qnorm(0.975) * sqrt(SEferm^2 + SEsea^
f <- ((coef(msm.fermdairy) - coef(msm.sea) + qnorm(0.975) * sqrt(SEferm^2 + SEsea^

g <- ((coef(msm.fermdairy) - coef(msm.eggs))*100)[2]
h <- ((coef(msm.fermdairy) - coef(msm.eggs) - qnorm(0.975) * sqrt(SEferm^2 + SEegg
i <- ((coef(msm.fermdairy) - coef(msm.eggs) + qnorm(0.975) * sqrt(SEferm^2 + SEegg

j <- ((coef(msm.fermdairy) - coef(msm.veg))*100)[2]
k <- ((coef(msm.fermdairy) - coef(msm.veg) - qnorm(0.975) * sqrt(SEferm^2 + SEveg^
l <- ((coef(msm.fermdairy) - coef(msm.veg) + qnorm(0.975) * sqrt(SEferm^2 + SEveg^

m <- ((coef(msm.fermdairy) - coef(msm.fru))*100)[2]
n <- ((coef(msm.fermdairy) - coef(msm.fru) - qnorm(0.975) * sqrt(SEferm^2 + SEfru^
o <- ((coef(msm.fermdairy) - coef(msm.fru) + qnorm(0.975) * sqrt(SEferm^2 + SEfru^

cdrferm <- c(paste(round(a,3)*100, "% (", round(b,3)*100,"% to ", round(c,3)*100,"
              paste(round(d,3)*100, "% (", round(e,3)*100,"% to ", round(f,3)*100,
              paste(round(g,3)*100, "% (", round(h,3)*100,"% to ", round(i,3)*100,
              paste(round(j,3)*100, "% (", round(k,3)*100,"% to ", round(l,3)*100,
              paste(round(m,3)*100, "% (", round(n,3)*100,"% to ", round(o,3)*100,

a <- ((coef(msm.nonfermdairy) - coef(msm.meat))*100)[2]
b <- ((coef(msm.nonfermdairy) - coef(msm.meat) - qnorm(0.975) * sqrt(SEnonferm^2 +
c <- ((coef(msm.nonfermdairy) - coef(msm.meat) + qnorm(0.975) * sqrt(SEnonferm^2 +

d <- ((coef(msm.nonfermdairy) - coef(msm.sea))*100)[2]
e <- ((coef(msm.nonfermdairy) - coef(msm.sea) - qnorm(0.975) * sqrt(SEnonferm^2 +
f <- ((coef(msm.nonfermdairy) - coef(msm.sea) + qnorm(0.975) * sqrt(SEnonferm^2 +

g <- ((coef(msm.nonfermdairy) - coef(msm.eggs))*100)[2]
h <- ((coef(msm.nonfermdairy) - coef(msm.eggs) - qnorm(0.975) * sqrt(SEnonferm^2 +
i <- ((coef(msm.nonfermdairy) - coef(msm.eggs) + qnorm(0.975) * sqrt(SEnonferm^2 +
```

```
j <- ((coef(msm.nonfermdairy) - coef(msm.veg))*100)[2]
k <- ((coef(msm.nonfermdairy) - coef(msm.veg) - qnorm(0.975) * sqrt(SEnonferm^2 + S
l <- ((coef(msm.nonfermdairy) - coef(msm.veg) + qnorm(0.975) * sqrt(SEnonferm^2 + S

m <- ((coef(msm.nonfermdairy) - coef(msm.fru))*100)[2]
n <- ((coef(msm.nonfermdairy) - coef(msm.fru) - qnorm(0.975) * sqrt(SEnonferm^2 + S
o <- ((coef(msm.nonfermdairy) - coef(msm.fru) + qnorm(0.975) * sqrt(SEnonferm^2 + S

cdrnonferm <- c(paste(round(a,4), "% (", round(b,3)*100,"% to ", round(c,3)*100,"%
              paste(round(d,3)*100, "% (", round(e,3)*100,"% to ", round(f,3)*100,
              paste(round(g,3)*100, "% (", round(h,3)*100,"% to ", round(i,3)*100,
              paste(round(j,3)*100, "% (", round(k,3)*100,"% to ", round(l,3)*100,
              paste(round(m,3)*100, "% (", round(n,3)*100,"% to ", round(o,3)*100,


a <- ((coef(msm.ffdairy) - coef(msm.meat))*100)[2]
b <- ((coef(msm.ffdairy) - coef(msm.meat) - qnorm(0.975) * sqrt(SEff^2 + SEmeat^2)
c <- ((coef(msm.ffdairy) - coef(msm.meat) + qnorm(0.975) * sqrt(SEff^2 + SEmeat^2)

d <- ((coef(msm.ffdairy) - coef(msm.sea))*100)[2]
e <- ((coef(msm.ffdairy) - coef(msm.sea) - qnorm(0.975) * sqrt(SEff^2 + SEsea^2))*
f <- ((coef(msm.ffdairy) - coef(msm.sea) + qnorm(0.975) * sqrt(SEff^2 + SEsea^2))*

g <- ((coef(msm.ffdairy) - coef(msm.eggs))*100)[2]
h <- ((coef(msm.ffdairy) - coef(msm.eggs) - qnorm(0.975) * sqrt(SEff^2 + SEeggs^2)
i <- ((coef(msm.ffdairy) - coef(msm.eggs) + qnorm(0.975) * sqrt(SEff^2 + SEeggs^2)

j <- ((coef(msm.ffdairy) - coef(msm.veg))*100)[2]
k <- ((coef(msm.ffdairy) - coef(msm.veg) - qnorm(0.975) * sqrt(SEff^2 + SEveg^2))*
l <- ((coef(msm.ffdairy) - coef(msm.veg) + qnorm(0.975) * sqrt(SEff^2 + SEveg^2))*

m <- ((coef(msm.ffdairy) - coef(msm.fru))*100)[2]
n <- ((coef(msm.ffdairy) - coef(msm.fru) - qnorm(0.975) * sqrt(SEff^2 + SEfru^2))*
o <- ((coef(msm.ffdairy) - coef(msm.fru) + qnorm(0.975) * sqrt(SEff^2 + SEfru^2))*

cdrfullfat <- c(paste(round(a,3)*100, "% (", round(b,3)*100,"% to ", round(c,3)*10
              paste(round(d,3)*100, "% (", round(e,3)*100,"% to ", round(f,3)*100,
              paste(round(g,3)*100, "% (", round(h,3)*100,"% to ", round(i,3)*100,
              paste(round(j,3)*100, "% (", round(k,3)*100,"% to ", round(l,3)*100,
              paste(round(m,3)*100, "% (", round(n,3)*100,"% to ", round(o,3)*100,


a <- ((coef(msm.nfdairy) - coef(msm.meat))*100)[2]
b <- ((coef(msm.nfdairy) - coef(msm.meat) - qnorm(0.975) * sqrt(SEnf^2 + SEmeat^2)
c <- ((coef(msm.nfdairy) - coef(msm.meat) + qnorm(0.975) * sqrt(SEnf^2 + SEmeat^2)

d <- ((coef(msm.nfdairy) - coef(msm.sea))*100)[2]
e <- ((coef(msm.nfdairy) - coef(msm.sea) - qnorm(0.975) * sqrt(SEnf^2 + SEsea^2))*
f <- ((coef(msm.nfdairy) - coef(msm.sea) + qnorm(0.975) * sqrt(SEnf^2 + SEsea^2))*

g <- ((coef(msm.nfdairy) - coef(msm.eggs))*100)[2]
h <- ((coef(msm.nfdairy) - coef(msm.eggs) - qnorm(0.975) * sqrt(SEnf^2 + SEeggs^2)
i <- ((coef(msm.nfdairy) - coef(msm.eggs) + qnorm(0.975) * sqrt(SEnf^2 + SEeggs^2)

j <- ((coef(msm.nfdairy) - coef(msm.veg))*100)[2]
k <- ((coef(msm.nfdairy) - coef(msm.veg) - qnorm(0.975) * sqrt(SEnf^2 + SEveg^2))*
l <- ((coef(msm.nfdairy) - coef(msm.veg) + qnorm(0.975) * sqrt(SEnf^2 + SEveg^2))*

m <- ((coef(msm.nfdairy) - coef(msm.fru))*100)[2]
n <- ((coef(msm.nfdairy) - coef(msm.fru) - qnorm(0.975) * sqrt(SEnf^2 + SEfru^2))*
o <- ((coef(msm.nfdairy) - coef(msm.fru) + qnorm(0.975) * sqrt(SEnf^2 + SEfru^2))*

cdrnonfat <- c(paste(round(a,3)*100, "% (", round(b,3)*100,"% to ", round(c,3)*100
```

```
              paste(round(d,3)*100, "% (", round(e,3)*100,"% to ", round(f,3)*100,
              paste(round(g,3)*100, "% (", round(h,3)*100,"% to ", round(i,3)*100,
              paste(round(j,3)*100, "% (", round(k,3)*100,"% to ", round(l,3)*100,
              paste(round(m,3)*100, "% (", round(n,3)*100,"% to ", round(o,3)*100,


a <- ((coef(msm.sugdairy) - coef(msm.meat))*100)[2]
b <- ((coef(msm.sugdairy) - coef(msm.meat) - qnorm(0.975) * sqrt(SEsug^2 + SEmeat^
c <- ((coef(msm.sugdairy) - coef(msm.meat) + qnorm(0.975) * sqrt(SEsug^2 + SEmeat^

d <- ((coef(msm.sugdairy) - coef(msm.sea))*100)[2]
e <- ((coef(msm.sugdairy) - coef(msm.sea) - qnorm(0.975) * sqrt(SEsug^2 + SEsea^2)
f <- ((coef(msm.sugdairy) - coef(msm.sea) + qnorm(0.975) * sqrt(SEsug^2 + SEsea^2)

g <- ((coef(msm.sugdairy) - coef(msm.eggs))*100)[2]
h <- ((coef(msm.sugdairy) - coef(msm.eggs) - qnorm(0.975) * sqrt(SEsug^2 + SEeggs^
i <- ((coef(msm.sugdairy) - coef(msm.eggs) + qnorm(0.975) * sqrt(SEsug^2 + SEeggs^

j <- ((coef(msm.sugdairy) - coef(msm.veg))*100)[2]
k <- ((coef(msm.sugdairy) - coef(msm.veg) - qnorm(0.975) * sqrt(SEsug^2 + SEveg^2)
l <- ((coef(msm.sugdairy) - coef(msm.veg) + qnorm(0.975) * sqrt(SEsug^2 + SEveg^2)

m <- ((coef(msm.sugdairy) - coef(msm.fru))*100)[2]
n <- ((coef(msm.sugdairy) - coef(msm.fru) - qnorm(0.975) * sqrt(SEsug^2 + SEfru^2)
o <- ((coef(msm.sugdairy) - coef(msm.fru) + qnorm(0.975) * sqrt(SEsug^2 + SEfru^2)

cdrsug <- c(paste(round(a,3)*100, "% (", round(b,3)*100,"% to ", round(c,3)*100,"%
              paste(round(d,3)*100, "% (", round(e,3)*100,"% to ", round(f,3)*100,
              paste(round(g,3)*100, "% (", round(h,3)*100,"% to ", round(i,3)*100,
              paste(round(j,3)*100, "% (", round(k,3)*100,"% to ", round(l,3)*100,
              paste(round(m,3)*100, "% (", round(n,3)*100,"% to ", round(o,3)*100,

CDR <- data.frame(cdrtotal,cdrferm, cdrnonferm, cdrfullfat, cdrnonfat, cdrsug)
CDR
```

A data.frame: 5 × 6

cdrtotal	cdrferm	cdrnonferm	cdrfullfat	cdrnonfat	cdrsug
<chr>	<chr>	<chr>	<chr>	<chr>	<chr>
0.5 % (-14.8 % to 15.8 %)	-0.3 % (-15.9 % to 15.4 %)	-0.0071 % (-16.7 % to 15.3 %)	-1.7 % (-17.2 % to 13.8 %)	7.3 % (-9.1 % to 23.7 %)	-1 % (-18.2 % to 16.2 %)
11.4 % (-20 % to 42.7 %)	10.6 % (-20.9 % to 42.2 %)	10.2 % (-21.5 % to 41.9 %)	9.3 % (-22.2 % to 40.7 %)	18.2 % (-13.7 % to 50.2 %)	9.9 % (-22.4 % to 42.2 %)
-9.2 % (-63.7 % to 45.3 %)	-10 % (-64.6 % to 44.6 %)	-10.4 % (-65.1 % to 44.3 %)	-11.4 % (-65.9 % to 43.2 %)	-2.4 % (-57.2 % to 52.5 %)	-10.7 % (-65.8 % to 44.4 %)
9.1 % (3.2 % to 14.9 %)	8.3 % (1.6 % to 15 %)	7.9 % (0.4 % to 15.4 %)	6.9 % (0.6 % to 13.3 %)	15.9 % (7.6 % to 24.3 %)	7.6 % (-2.3 % to 17.4 %)
1.2 % (-4.2 % to 6.5 %)	0.4 % (-5.9 % to 6.7 %)	0 % (-7.1 % to 7.1 %)	-0.9 % (-6.9 % to 5 %)	8 % (0 % to 16 %)	-0.3 % (-9.8 % to 9.3 %)

SCD

In [87]:

```
ch_sensi$F3SCD_yn <- as.numeric(ch_sensi$F3SCD_yn)

mem <- ch_sensi %>% filter(!is.na(F3SCD_yn))

msm.totdairy <- geeglm(F3SCD_yn ~ total_dairy_avg, data=mem, weights=ipw_totdairy,
                       corstr="independence")
```

```
SEtotal <- coef(summary(msm.totdairy))[,2]

msm.fermdairy <- geeglm(F3SCD_yn ~ ferm_dairy_avg, data=mem, weights=ipw_fermdairy
                corstr="independence")
SEferm <- coef(summary(msm.fermdairy))[,2]

msm.nonfermdairy <- geeglm(F3SCD_yn ~ nonferm_dairy_avg, data=mem, weights=ipw_non
                corstr="independence")
SEnonferm <- coef(summary(msm.nonfermdairy))[,2]

msm.ffdairy <- geeglm(F3SCD_yn ~ fullfat_dairy_avg, data=mem, weights=ipw_fullfatd
                corstr="independence")
SEff <- coef(summary(msm.ffdairy))[,2]

msm.nfdairy <- geeglm(F3SCD_yn ~ nonfat_dairy_avg, data=mem, weights=ipw_nonfatdai
                corstr="independence")
SEnf <- coef(summary(msm.nfdairy))[,2]

msm.sugdairy <- geeglm(F3SCD_yn ~ sugar_dairy_avg, data=mem, weights=ipw_sugdairy,
                corstr="independence")
SEsug <- coef(summary(msm.sugdairy))[,2]

msm.meat <- geeglm(F3SCD_yn ~ meat_avg, data=mem, weights=ipw_meat, id=pt,
                corstr="independence")
SEmeat <- coef(summary(msm.meat))[,2]

msm.sea <- geeglm(F3SCD_yn ~ sea_avg, data=mem, weights=ipw_sea, id=pt,
                corstr="independence")
SEsea <- coef(summary(msm.sea))[,2]


msm.veg <- geeglm(F3SCD_yn ~ veg_avg, data=mem, weights=ipw_veg, id=pt,
                corstr="independence")
SEveg <- coef(summary(msm.veg))[,2]


msm.fru <- geeglm(F3SCD_yn ~ fru_avg, data=mem, weights=ipw_fru, id=pt,
                corstr="independence")
SEfru <- coef(summary(msm.fru))[,2]


msm.eggs <- geeglm(F3SCD_yn ~ eggs_avg, data=mem, weights=ipw_eggs, id=pt,
                corstr="independence")
SEeggs <- coef(summary(msm.eggs))[,2]

a <- ((coef(msm.totdairy) - coef(msm.meat))*100)[2]
b <- ((coef(msm.totdairy) - coef(msm.meat) - qnorm(0.975) * sqrt(SEtotal^2 + SEmea
c <- ((coef(msm.totdairy) - coef(msm.meat) + qnorm(0.975) * sqrt(SEtotal^2 + SEmea

d <- ((coef(msm.totdairy) - coef(msm.sea))*100)[2]
e <- ((coef(msm.totdairy) - coef(msm.sea) - qnorm(0.975) * sqrt(SEtotal^2 + SEsea^
f <- ((coef(msm.totdairy) - coef(msm.sea) + qnorm(0.975) * sqrt(SEtotal^2 + SEsea^

g <- ((coef(msm.totdairy) - coef(msm.eggs))*100)[2]
h <- ((coef(msm.totdairy) - coef(msm.eggs) - qnorm(0.975) * sqrt(SEtotal^2 + SEegg
i <- ((coef(msm.totdairy) - coef(msm.eggs) + qnorm(0.975) * sqrt(SEtotal^2 + SEegg

j <- ((coef(msm.totdairy) - coef(msm.veg))*100)[2]
k <- ((coef(msm.totdairy) - coef(msm.veg) - qnorm(0.975) * sqrt(SEtotal^2 + SEveg^
l <- ((coef(msm.totdairy) - coef(msm.veg) + qnorm(0.975) * sqrt(SEtotal^2 + SEveg^

m <- ((coef(msm.totdairy) - coef(msm.fru))*100)[2]
n <- ((coef(msm.totdairy) - coef(msm.fru) - qnorm(0.975) * sqrt(SEtotal^2 + SEfru^
o <- ((coef(msm.totdairy) - coef(msm.fru) + qnorm(0.975) * sqrt(SEtotal^2 + SEfru^
```

```
scdtotal <- c(paste(round(a,3)*100, "% (", round(b,3)*100,"% to ", round(c,3)*100,
              paste(round(d,3)*100, "% (", round(e,3)*100,"% to ", round(f,3)*100,
              paste(round(g,3)*100, "% (", round(h,3)*100,"% to ", round(i,3)*100,
              paste(round(j,3)*100, "% (", round(k,3)*100,"% to ", round(l,3)*100,
              paste(round(m,3)*100, "% (", round(n,3)*100,"% to ", round(o,3)*100,

a <- ((coef(msm.fermdairy) - coef(msm.meat))*100)[2]
b <- ((coef(msm.fermdairy) - coef(msm.meat) - qnorm(0.975) * sqrt(SEferm^2 + SEmea
c <- ((coef(msm.fermdairy) - coef(msm.meat) + qnorm(0.975) * sqrt(SEferm^2 + SEmea

d <- ((coef(msm.fermdairy) - coef(msm.sea))*100)[2]
e <- ((coef(msm.fermdairy) - coef(msm.sea) - qnorm(0.975) * sqrt(SEferm^2 + SEsea^
f <- ((coef(msm.fermdairy) - coef(msm.sea) + qnorm(0.975) * sqrt(SEferm^2 + SEsea^

g <- ((coef(msm.fermdairy) - coef(msm.eggs))*100)[2]
h <- ((coef(msm.fermdairy) - coef(msm.eggs) - qnorm(0.975) * sqrt(SEferm^2 + SEegg
i <- ((coef(msm.fermdairy) - coef(msm.eggs) + qnorm(0.975) * sqrt(SEferm^2 + SEegg

j <- ((coef(msm.fermdairy) - coef(msm.veg))*100)[2]
k <- ((coef(msm.fermdairy) - coef(msm.veg) - qnorm(0.975) * sqrt(SEferm^2 + SEveg^
l <- ((coef(msm.fermdairy) - coef(msm.veg) + qnorm(0.975) * sqrt(SEferm^2 + SEveg^

m <- ((coef(msm.fermdairy) - coef(msm.fru))*100)[2]
n <- ((coef(msm.fermdairy) - coef(msm.fru) - qnorm(0.975) * sqrt(SEferm^2 + SEfru^
o <- ((coef(msm.fermdairy) - coef(msm.fru) + qnorm(0.975) * sqrt(SEferm^2 + SEfru^

scdferm <- c(paste(round(a,3)*100, "% (", round(b,3)*100,"% to ", round(c,3)*100,"
              paste(round(d,3)*100, "% (", round(e,3)*100,"% to ", round(f,3)*100,
              paste(round(g,3)*100, "% (", round(h,3)*100,"% to ", round(i,3)*100,
              paste(round(j,3)*100, "% (", round(k,3)*100,"% to ", round(l,3)*100,
              paste(round(m,3)*100, "% (", round(n,3)*100,"% to ", round(o,3)*100,

a <- ((coef(msm.nonfermdairy) - coef(msm.meat))*100)[2]
b <- ((coef(msm.nonfermdairy) - coef(msm.meat) - qnorm(0.975) * sqrt(SEnonferm^2 +
c <- ((coef(msm.nonfermdairy) - coef(msm.meat) + qnorm(0.975) * sqrt(SEnonferm^2 +

d <- ((coef(msm.nonfermdairy) - coef(msm.sea))*100)[2]
e <- ((coef(msm.nonfermdairy) - coef(msm.sea) - qnorm(0.975) * sqrt(SEnonferm^2 +
f <- ((coef(msm.nonfermdairy) - coef(msm.sea) + qnorm(0.975) * sqrt(SEnonferm^2 +

g <- ((coef(msm.nonfermdairy) - coef(msm.eggs))*100)[2]
h <- ((coef(msm.nonfermdairy) - coef(msm.eggs) - qnorm(0.975) * sqrt(SEnonferm^2 +
i <- ((coef(msm.nonfermdairy) - coef(msm.eggs) + qnorm(0.975) * sqrt(SEnonferm^2 +

j <- ((coef(msm.nonfermdairy) - coef(msm.veg))*100)[2]
k <- ((coef(msm.nonfermdairy) - coef(msm.veg) - qnorm(0.975) * sqrt(SEnonferm^2 +
l <- ((coef(msm.nonfermdairy) - coef(msm.veg) + qnorm(0.975) * sqrt(SEnonferm^2 +

m <- ((coef(msm.nonfermdairy) - coef(msm.fru))*100)[2]
n <- ((coef(msm.nonfermdairy) - coef(msm.fru) - qnorm(0.975) * sqrt(SEnonferm^2 +
o <- ((coef(msm.nonfermdairy) - coef(msm.fru) + qnorm(0.975) * sqrt(SEnonferm^2 +

scdnonferm <- c(paste(round(a,3)*100, "% (", round(b,3)*100,"% to ", round(c,3)*10
              paste(round(d,3)*100, "% (", round(e,3)*100,"% to ", round(f,3)*100,
              paste(round(g,3)*100, "% (", round(h,3)*100,"% to ", round(i,3)*100,
              paste(round(j,3)*100, "% (", round(k,3)*100,"% to ", round(l,3)*100,
              paste(round(m,3)*100, "% (", round(n,3)*100,"% to ", round(o,3)*100,


a <- ((coef(msm.ffdairy) - coef(msm.meat))*100)[2]
b <- ((coef(msm.ffdairy) - coef(msm.meat) - qnorm(0.975) * sqrt(SEff^2 + SEmeat^2)
c <- ((coef(msm.ffdairy) - coef(msm.meat) + qnorm(0.975) * sqrt(SEff^2 + SEmeat^2)
```

```
d <- ((coef(msm.ffdairy) - coef(msm.sea))*100)[2]
e <- ((coef(msm.ffdairy) - coef(msm.sea) - qnorm(0.975) * sqrt(SEff^2 + SEsea^2))*
f <- ((coef(msm.ffdairy) - coef(msm.sea) + qnorm(0.975) * sqrt(SEff^2 + SEsea^2))*

g <- ((coef(msm.ffdairy) - coef(msm.eggs))*100)[2]
h <- ((coef(msm.ffdairy) - coef(msm.eggs) - qnorm(0.975) * sqrt(SEff^2 + SEeggs^2)
i <- ((coef(msm.ffdairy) - coef(msm.eggs) + qnorm(0.975) * sqrt(SEff^2 + SEeggs^2)

j <- ((coef(msm.ffdairy) - coef(msm.veg))*100)[2]
k <- ((coef(msm.ffdairy) - coef(msm.veg) - qnorm(0.975) * sqrt(SEff^2 + SEveg^2))*
l <- ((coef(msm.ffdairy) - coef(msm.veg) + qnorm(0.975) * sqrt(SEff^2 + SEveg^2))*

m <- ((coef(msm.ffdairy) - coef(msm.fru))*100)[2]
n <- ((coef(msm.ffdairy) - coef(msm.fru) - qnorm(0.975) * sqrt(SEff^2 + SEfru^2))*
o <- ((coef(msm.ffdairy) - coef(msm.fru) + qnorm(0.975) * sqrt(SEff^2 + SEfru^2))*

scdfullfat <- c(paste(round(a,3)*100, "% (", round(b,3)*100,"% to ", round(c,3)*10
              paste(round(d,3)*100, "% (", round(e,3)*100,"% to ", round(f,3)*100,
              paste(round(g,3)*100, "% (", round(h,3)*100,"% to ", round(i,3)*100,
              paste(round(j,3)*100, "% (", round(k,3)*100,"% to ", round(l,3)*100,
              paste(round(m,3)*100, "% (", round(n,3)*100,"% to ", round(o,3)*100,


a <- ((coef(msm.nfdairy) - coef(msm.meat))*100)[2]
b <- ((coef(msm.nfdairy) - coef(msm.meat) - qnorm(0.975) * sqrt(SEnf^2 + SEmeat^2)
c <- ((coef(msm.nfdairy) - coef(msm.meat) + qnorm(0.975) * sqrt(SEnf^2 + SEmeat^2)

d <- ((coef(msm.nfdairy) - coef(msm.sea))*100)[2]
e <- ((coef(msm.nfdairy) - coef(msm.sea) - qnorm(0.975) * sqrt(SEnf^2 + SEsea^2))*
f <- ((coef(msm.nfdairy) - coef(msm.sea) + qnorm(0.975) * sqrt(SEnf^2 + SEsea^2))*

g <- ((coef(msm.nfdairy) - coef(msm.eggs))*100)[2]
h <- ((coef(msm.nfdairy) - coef(msm.eggs) - qnorm(0.975) * sqrt(SEnf^2 + SEeggs^2)
i <- ((coef(msm.nfdairy) - coef(msm.eggs) + qnorm(0.975) * sqrt(SEnf^2 + SEeggs^2)

j <- ((coef(msm.nfdairy) - coef(msm.veg))*100)[2]
k <- ((coef(msm.nfdairy) - coef(msm.veg) - qnorm(0.975) * sqrt(SEnf^2 + SEveg^2))*
l <- ((coef(msm.nfdairy) - coef(msm.veg) + qnorm(0.975) * sqrt(SEnf^2 + SEveg^2))*

m <- ((coef(msm.nfdairy) - coef(msm.fru))*100)[2]
n <- ((coef(msm.nfdairy) - coef(msm.fru) - qnorm(0.975) * sqrt(SEnf^2 + SEfru^2))*
o <- ((coef(msm.nfdairy) - coef(msm.fru) + qnorm(0.975) * sqrt(SEnf^2 + SEfru^2))*

scdnonfat <- c(paste(round(a,3)*100, "% (", round(b,3)*100,"% to ", round(c,3)*100
              paste(round(d,3)*100, "% (", round(e,3)*100,"% to ", round(f,3)*100,
              paste(round(g,3)*100, "% (", round(h,3)*100,"% to ", round(i,3)*100,
              paste(round(j,3)*100, "% (", round(k,3)*100,"% to ", round(l,3)*100,
              paste(round(m,3)*100, "% (", round(n,3)*100,"% to ", round(o,3)*100,


a <- ((coef(msm.sugdairy) - coef(msm.meat))*100)[2]
b <- ((coef(msm.sugdairy) - coef(msm.meat) - qnorm(0.975) * sqrt(SEsug^2 + SEmeat^
c <- ((coef(msm.sugdairy) - coef(msm.meat) + qnorm(0.975) * sqrt(SEsug^2 + SEmeat^

d <- ((coef(msm.sugdairy) - coef(msm.sea))*100)[2]
e <- ((coef(msm.sugdairy) - coef(msm.sea) - qnorm(0.975) * sqrt(SEsug^2 + SEsea^2)
f <- ((coef(msm.sugdairy) - coef(msm.sea) + qnorm(0.975) * sqrt(SEsug^2 + SEsea^2)

g <- ((coef(msm.sugdairy) - coef(msm.eggs))*100)[2]
h <- ((coef(msm.sugdairy) - coef(msm.eggs) - qnorm(0.975) * sqrt(SEsug^2 + SEeggs^
i <- ((coef(msm.sugdairy) - coef(msm.eggs) + qnorm(0.975) * sqrt(SEsug^2 + SEeggs^

j <- ((coef(msm.sugdairy) - coef(msm.veg))*100)[2]
k <- ((coef(msm.sugdairy) - coef(msm.veg) - qnorm(0.975) * sqrt(SEsug^2 + SEveg^2)
```

```
l <- ((coef(msm.sugdairy) - coef(msm.veg) + qnorm(0.975) * sqrt(SEsug^2 + SEveg^2)

m <- ((coef(msm.sugdairy) - coef(msm.fru))*100)[2]
n <- ((coef(msm.sugdairy) - coef(msm.fru) - qnorm(0.975) * sqrt(SEsug^2 + SEfru^2)
o <- ((coef(msm.sugdairy) - coef(msm.fru) + qnorm(0.975) * sqrt(SEsug^2 + SEfru^2)

scdsug <- c(paste(round(a,3)*100, "% (", round(b,3)*100,"% to ", round(c,3)*100,"%
              paste(round(d,3)*100, "% (", round(e,3)*100,"% to ", round(f,3)*100,
              paste(round(g,3)*100, "% (", round(h,3)*100,"% to ", round(i,3)*100,
              paste(round(j,3)*100, "% (", round(k,3)*100,"% to ", round(l,3)*100,
              paste(round(m,3)*100, "% (", round(n,3)*100,"% to ", round(o,3)*100,

SCD <- data.frame(scdtotal,scdferm, scdnonferm, scdfullfat, scdnonfat, scdsug)
SCD
```

A data.frame: 5 × 6

scdtotal	scdferm	scdnonferm	scdfullfat	scdnonfat	scdsug
<chr>	<chr>	<chr>	<chr>	<chr>	<chr>
-0.7 % (-11.4 % to 10.1 %)	-0.2 % (-11.2 % to 10.8 %)	-1.8 % (-13.2 % to 9.6 %)	-0.2 % (-11 % to 10.6 %)	-1.9 % (-13.9 % to 10.2 %)	-0.6 % (-12.1 % to 11 %)
-8.8 % (-29.1 % to 11.4 %)	-8.4 % (-28.8 % to 12 %)	-9.9 % (-30.5 % to 10.6 %)	-8.3 % (-28.6 % to 11.9 %)	-10 % (-31 % to 10.9 %)	-8.7 % (-29.4 % to 11.9 %)
-8 % (-41.3 % to 25.4 %)	-7.5 % (-41 % to 25.9 %)	-9.1 % (-42.7 % to 24.5 %)	-7.5 % (-40.9 % to 25.9 %)	-9.2 % (-43 % to 24.6 %)	-7.9 % (-41.5 % to 25.8 %)
1 % (-3.9 % to 5.9 %)	1.4 % (-4 % to 6.9 %)	-0.1 % (-6.3 % to 6 %)	1.4 % (-3.6 % to 6.5 %)	-0.2 % (-7.6 % to 7.1 %)	1.1 % (-5.4 % to 7.5 %)
3.4 % (1 % to 5.8 %)	3.8 % (0.5 % to 7.2 %)	2.3 % (-2.2 % to 6.7 %)	3.9 % (1.2 % to 6.5 %)	2.2 % (-3.8 % to 8.2 %)	3.5 % (-1.3 % to 8.3 %)

Memory

In [88]:

```
ch_sensi$F3memory_cat <- as.numeric(ch_sensi$F3memory_cat)

mem <- ch_sensi %>% filter(!is.na(F3memory_cat))

msm.totdairy <- geeglm(F3memory_cat ~ total_dairy_avg, data=mem, weights=ipw_totda
                       corstr="independence")
SEtotal <- coef(summary(msm.totdairy))[,2]

msm.fermdairy <- geeglm(F3memory_cat ~ ferm_dairy_avg, data=mem, weights=ipw_fermd
                corstr="independence")
SEferm <- coef(summary(msm.fermdairy))[,2]

msm.nonfermdairy <- geeglm(F3memory_cat ~ nonferm_dairy_avg, data=mem, weights=ipw_
                corstr="independence")
SEnonferm <- coef(summary(msm.nonfermdairy))[,2]

msm.ffdairy <- geeglm(F3memory_cat ~ fullfat_dairy_avg, data=mem, weights=ipw_full
                corstr="independence")
SEff <- coef(summary(msm.ffdairy))[,2]

msm.nfdairy <- geeglm(F3memory_cat ~ nonfat_dairy_avg, data=mem, weights=ipw_nonfa
                corstr="independence")
SEnf <- coef(summary(msm.nfdairy))[,2]

msm.sugdairy <- geeglm(F3memory_cat ~ sugar_dairy_avg, data=mem, weights=ipw_sugda
                corstr="independence")
```

```
SEsug <- coef(summary(msm.sugdairy))[,2]

msm.meat <- geeglm(F3memory_cat ~ meat_avg, data=mem, weights=ipw_meat, id=pt,
                corstr="independence")
SEmeat <- coef(summary(msm.meat))[,2]

msm.sea <- geeglm(F3memory_cat ~ sea_avg, data=mem, weights=ipw_sea, id=pt,
                corstr="independence")
SEsea <- coef(summary(msm.sea))[,2]

msm.veg <- geeglm(F3memory_cat ~ veg_avg, data=mem, weights=ipw_veg, id=pt,
                corstr="independence")
SEveg <- coef(summary(msm.veg))[,2]


msm.fru <- geeglm(F3memory_cat ~ fru_avg, data=mem, weights=ipw_fru, id=pt,
                corstr="independence")
SEfru <- coef(summary(msm.fru))[,2]


msm.eggs <- geeglm(F3memory_cat ~ eggs_avg, data=mem, weights=ipw_eggs, id=pt,
                corstr="independence")
SEeggs <- coef(summary(msm.eggs))[,2]


a <- ((coef(msm.totdairy) - coef(msm.meat))*100)[2]
b <- ((coef(msm.totdairy) - coef(msm.meat) - qnorm(0.975) * sqrt(SEtotal^2 + SEmea
c <- ((coef(msm.totdairy) - coef(msm.meat) + qnorm(0.975) * sqrt(SEtotal^2 + SEmea

d <- ((coef(msm.totdairy) - coef(msm.sea))*100)[2]
e <- ((coef(msm.totdairy) - coef(msm.sea) - qnorm(0.975) * sqrt(SEtotal^2 + SEsea^
f <- ((coef(msm.totdairy) - coef(msm.sea) + qnorm(0.975) * sqrt(SEtotal^2 + SEsea^

g <- ((coef(msm.totdairy) - coef(msm.eggs))*100)[2]
h <- ((coef(msm.totdairy) - coef(msm.eggs) - qnorm(0.975) * sqrt(SEtotal^2 + SEegg
i <- ((coef(msm.totdairy) - coef(msm.eggs) + qnorm(0.975) * sqrt(SEtotal^2 + SEegg

j <- ((coef(msm.totdairy) - coef(msm.veg))*100)[2]
k <- ((coef(msm.totdairy) - coef(msm.veg) - qnorm(0.975) * sqrt(SEtotal^2 + SEveg^
l <- ((coef(msm.totdairy) - coef(msm.veg) + qnorm(0.975) * sqrt(SEtotal^2 + SEveg^

m <- ((coef(msm.totdairy) - coef(msm.fru))*100)[2]
n <- ((coef(msm.totdairy) - coef(msm.fru) - qnorm(0.975) * sqrt(SEtotal^2 + SEfru^
o <- ((coef(msm.totdairy) - coef(msm.fru) + qnorm(0.975) * sqrt(SEtotal^2 + SEfru^

memorytotal <- c(paste(round(a,3)*100, "% (", round(b,3)*100,"% to ", round(c,3)*1
              paste(round(d,3)*100, "% (", round(e,3)*100,"% to ", round(f,3)*100,
              paste(round(g,3)*100, "% (", round(h,3)*100,"% to ", round(i,3)*100,
              paste(round(j,3)*100, "% (", round(k,3)*100,"% to ", round(l,3)*100,
              paste(round(m,3)*100, "% (", round(n,3)*100,"% to ", round(o,3)*100,

a <- ((coef(msm.fermdairy) - coef(msm.meat))*100)[2]
b <- ((coef(msm.fermdairy) - coef(msm.meat) - qnorm(0.975) * sqrt(SEferm^2 + SEmea
c <- ((coef(msm.fermdairy) - coef(msm.meat) + qnorm(0.975) * sqrt(SEferm^2 + SEmea

d <- ((coef(msm.fermdairy) - coef(msm.sea))*100)[2]
e <- ((coef(msm.fermdairy) - coef(msm.sea) - qnorm(0.975) * sqrt(SEferm^2 + SEsea^
f <- ((coef(msm.fermdairy) - coef(msm.sea) + qnorm(0.975) * sqrt(SEferm^2 + SEsea^

g <- ((coef(msm.fermdairy) - coef(msm.eggs))*100)[2]
h <- ((coef(msm.fermdairy) - coef(msm.eggs) - qnorm(0.975) * sqrt(SEferm^2 + SEegg
i <- ((coef(msm.fermdairy) - coef(msm.eggs) + qnorm(0.975) * sqrt(SEferm^2 + SEegg
```

```
j <- ((coef(msm.fermdairy) - coef(msm.veg))*100)[2]
k <- ((coef(msm.fermdairy) - coef(msm.veg) - qnorm(0.975) * sqrt(SEferm^2 + SEveg^
l <- ((coef(msm.fermdairy) - coef(msm.veg) + qnorm(0.975) * sqrt(SEferm^2 + SEveg^

m <- ((coef(msm.fermdairy) - coef(msm.fru))*100)[2]
n <- ((coef(msm.fermdairy) - coef(msm.fru) - qnorm(0.975) * sqrt(SEferm^2 + SEfru^
o <- ((coef(msm.fermdairy) - coef(msm.fru) + qnorm(0.975) * sqrt(SEferm^2 + SEfru^

memoryferm <- c(paste(round(a,3)*100, "% (", round(b,3)*100,"% to ", round(c,3)*10
              paste(round(d,3)*100, "% (", round(e,3)*100,"% to ", round(f,3)*100,
              paste(round(g,3)*100, "% (", round(h,3)*100,"% to ", round(i,3)*100,
              paste(round(j,3)*100, "% (", round(k,3)*100,"% to ", round(l,3)*100,
              paste(round(m,3)*100, "% (", round(n,3)*100,"% to ", round(o,3)*100,

a <- ((coef(msm.nonfermdairy) - coef(msm.meat))*100)[2]
b <- ((coef(msm.nonfermdairy) - coef(msm.meat) - qnorm(0.975) * sqrt(SEnonferm^2 +
c <- ((coef(msm.nonfermdairy) - coef(msm.meat) + qnorm(0.975) * sqrt(SEnonferm^2 +

d <- ((coef(msm.nonfermdairy) - coef(msm.sea))*100)[2]
e <- ((coef(msm.nonfermdairy) - coef(msm.sea) - qnorm(0.975) * sqrt(SEnonferm^2 +
f <- ((coef(msm.nonfermdairy) - coef(msm.sea) + qnorm(0.975) * sqrt(SEnonferm^2 +

g <- ((coef(msm.nonfermdairy) - coef(msm.eggs))*100)[2]
h <- ((coef(msm.nonfermdairy) - coef(msm.eggs) - qnorm(0.975) * sqrt(SEnonferm^2 +
i <- ((coef(msm.nonfermdairy) - coef(msm.eggs) + qnorm(0.975) * sqrt(SEnonferm^2 +

j <- ((coef(msm.nonfermdairy) - coef(msm.veg))*100)[2]
k <- ((coef(msm.nonfermdairy) - coef(msm.veg) - qnorm(0.975) * sqrt(SEnonferm^2 +
l <- ((coef(msm.nonfermdairy) - coef(msm.veg) + qnorm(0.975) * sqrt(SEnonferm^2 +

m <- ((coef(msm.nonfermdairy) - coef(msm.fru))*100)[2]
n <- ((coef(msm.nonfermdairy) - coef(msm.fru) - qnorm(0.975) * sqrt(SEnonferm^2 +
o <- ((coef(msm.nonfermdairy) - coef(msm.fru) + qnorm(0.975) * sqrt(SEnonferm^2 +

memorynonferm <- c(paste(round(a,3)*100, "% (", round(b,3)*100,"% to ", round(c,3)
              paste(round(d,3)*100, "% (", round(e,3)*100,"% to ", round(f,3)*100,
              paste(round(g,3)*100, "% (", round(h,3)*100,"% to ", round(i,3)*100,
              paste(round(j,3)*100, "% (", round(k,3)*100,"% to ", round(l,3)*100,
              paste(round(m,3)*100, "% (", round(n,3)*100,"% to ", round(o,3)*100,


a <- ((coef(msm.ffdairy) - coef(msm.meat))*100)[2]
b <- ((coef(msm.ffdairy) - coef(msm.meat) - qnorm(0.975) * sqrt(SEff^2 + SEmeat^2)
c <- ((coef(msm.ffdairy) - coef(msm.meat) + qnorm(0.975) * sqrt(SEff^2 + SEmeat^2)

d <- ((coef(msm.ffdairy) - coef(msm.sea))*100)[2]
e <- ((coef(msm.ffdairy) - coef(msm.sea) - qnorm(0.975) * sqrt(SEff^2 + SEsea^2))*
f <- ((coef(msm.ffdairy) - coef(msm.sea) + qnorm(0.975) * sqrt(SEff^2 + SEsea^2))*

g <- ((coef(msm.ffdairy) - coef(msm.eggs))*100)[2]
h <- ((coef(msm.ffdairy) - coef(msm.eggs) - qnorm(0.975) * sqrt(SEff^2 + SEeggs^2)
i <- ((coef(msm.ffdairy) - coef(msm.eggs) + qnorm(0.975) * sqrt(SEff^2 + SEeggs^2)

j <- ((coef(msm.ffdairy) - coef(msm.veg))*100)[2]
k <- ((coef(msm.ffdairy) - coef(msm.veg) - qnorm(0.975) * sqrt(SEff^2 + SEveg^2))*
l <- ((coef(msm.ffdairy) - coef(msm.veg) + qnorm(0.975) * sqrt(SEff^2 + SEveg^2))*

m <- ((coef(msm.ffdairy) - coef(msm.fru))*100)[2]
n <- ((coef(msm.ffdairy) - coef(msm.fru) - qnorm(0.975) * sqrt(SEff^2 + SEfru^2))*
o <- ((coef(msm.ffdairy) - coef(msm.fru) + qnorm(0.975) * sqrt(SEff^2 + SEfru^2))*

memoryfullfat <- c(paste(round(a,3)*100, "% (", round(b,3)*100,"% to ", round(c,3)
              paste(round(d,3)*100, "% (", round(e,3)*100,"% to ", round(f,3)*100,
              paste(round(g,3)*100, "% (", round(h,3)*100,"% to ", round(i,3)*100,
```

```
                paste(round(j,3)*100, "% (", round(k,3)*100,"% to ", round(l,3)*100,
                paste(round(m,3)*100, "% (", round(n,3)*100,"% to ", round(o,3)*100,


a <- ((coef(msm.nfdairy) - coef(msm.meat))*100)[2]
b <- ((coef(msm.nfdairy) - coef(msm.meat) - qnorm(0.975) * sqrt(SEnf^2 + SEmeat^2)
c <- ((coef(msm.nfdairy) - coef(msm.meat) + qnorm(0.975) * sqrt(SEnf^2 + SEmeat^2)

d <- ((coef(msm.nfdairy) - coef(msm.sea))*100)[2]
e <- ((coef(msm.nfdairy) - coef(msm.sea) - qnorm(0.975) * sqrt(SEnf^2 + SEsea^2))*
f <- ((coef(msm.nfdairy) - coef(msm.sea) + qnorm(0.975) * sqrt(SEnf^2 + SEsea^2))*

g <- ((coef(msm.nfdairy) - coef(msm.eggs))*100)[2]
h <- ((coef(msm.nfdairy) - coef(msm.eggs) - qnorm(0.975) * sqrt(SEnf^2 + SEeggs^2)
i <- ((coef(msm.nfdairy) - coef(msm.eggs) + qnorm(0.975) * sqrt(SEnf^2 + SEeggs^2)

j <- ((coef(msm.nfdairy) - coef(msm.veg))*100)[2]
k <- ((coef(msm.nfdairy) - coef(msm.veg) - qnorm(0.975) * sqrt(SEnf^2 + SEveg^2))*
l <- ((coef(msm.nfdairy) - coef(msm.veg) + qnorm(0.975) * sqrt(SEnf^2 + SEveg^2))*

m <- ((coef(msm.nfdairy) - coef(msm.fru))*100)[2]
n <- ((coef(msm.nfdairy) - coef(msm.fru) - qnorm(0.975) * sqrt(SEnf^2 + SEfru^2))*
o <- ((coef(msm.nfdairy) - coef(msm.fru) + qnorm(0.975) * sqrt(SEnf^2 + SEfru^2))*

memorynonfat <- c(paste(round(a,3)*100, "% (", round(b,3)*100,"% to ", round(c,3)*
                paste(round(d,3)*100, "% (", round(e,3)*100,"% to ", round(f,3)*100,
                paste(round(g,3)*100, "% (", round(h,3)*100,"% to ", round(i,3)*100,
                paste(round(j,3)*100, "% (", round(k,3)*100,"% to ", round(l,3)*100,
                paste(round(m,3)*100, "% (", round(n,3)*100,"% to ", round(o,3)*100,


a <- ((coef(msm.sugdairy) - coef(msm.meat))*100)[2]
b <- ((coef(msm.sugdairy) - coef(msm.meat) - qnorm(0.975) * sqrt(SEsug^2 + SEmeat^
c <- ((coef(msm.sugdairy) - coef(msm.meat) + qnorm(0.975) * sqrt(SEsug^2 + SEmeat^

d <- ((coef(msm.sugdairy) - coef(msm.sea))*100)[2]
e <- ((coef(msm.sugdairy) - coef(msm.sea) - qnorm(0.975) * sqrt(SEsug^2 + SEsea^2)
f <- ((coef(msm.sugdairy) - coef(msm.sea) + qnorm(0.975) * sqrt(SEsug^2 + SEsea^2)

g <- ((coef(msm.sugdairy) - coef(msm.eggs))*100)[2]
h <- ((coef(msm.sugdairy) - coef(msm.eggs) - qnorm(0.975) * sqrt(SEsug^2 + SEeggs^
i <- ((coef(msm.sugdairy) - coef(msm.eggs) + qnorm(0.975) * sqrt(SEsug^2 + SEeggs^

j <- ((coef(msm.sugdairy) - coef(msm.veg))*100)[2]
k <- ((coef(msm.sugdairy) - coef(msm.veg) - qnorm(0.975) * sqrt(SEsug^2 + SEveg^2)
l <- ((coef(msm.sugdairy) - coef(msm.veg) + qnorm(0.975) * sqrt(SEsug^2 + SEveg^2)

m <- ((coef(msm.sugdairy) - coef(msm.fru))*100)[2]
n <- ((coef(msm.sugdairy) - coef(msm.fru) - qnorm(0.975) * sqrt(SEsug^2 + SEfru^2)
o <- ((coef(msm.sugdairy) - coef(msm.fru) + qnorm(0.975) * sqrt(SEsug^2 + SEfru^2)

memorysug <- c(paste(round(a,3)*100, "% (", round(b,3)*100,"% to ", round(c,3)*100
                paste(round(d,3)*100, "% (", round(e,3)*100,"% to ", round(f,3)*100,
                paste(round(g,3)*100, "% (", round(h,3)*100,"% to ", round(i,3)*100,
                paste(round(j,3)*100, "% (", round(k,3)*100,"% to ", round(l,3)*100,
                paste(round(m,3)*100, "% (", round(n,3)*100,"% to ", round(o,3)*100,

MEMORY <- data.frame(memorytotal,memoryferm, memorynonferm, memoryfullfat, memoryn
MEMORY
```

A data.frame: 5 × 6

memorytotal	memoryferm	memorynonferm	memoryfullfat	memorynonfat	memorysug
<chr>	<chr>	<chr>	<chr>	<chr>	<chr>
-2.5 % (-12.2 % to 7.2 %)	-1.7 % (-11.6 % to 8.2 %)	-5.9 % (-16.1 % to 4.4 %)	-3 % (-12.9 % to 6.9 %)	-6.6 % (-17.3 % to 4.1 %)	1.7 % (-9.7 % to 13.2 %)
3 % (-14.4 % to 20.4 %)	3.8 % (-13.7 % to 21.4 %)	-0.3 % (-18.1 % to 17.4 %)	2.5 % (-15 % to 20.1 %)	-1.1 % (-19 % to 16.9 %)	7.2 % (-11.2 % to 25.7 %)
18.4 % (-18.1 % to 54.8 %)	19.2 % (-17.3 % to 55.7 %)	15 % (-21.6 % to 51.6 %)	17.9 % (-18.6 % to 54.4 %)	14.3 % (-22.4 % to 51 %)	22.6 % (-14.4 % to 59.5 %)
4 % (-0.3 % to 8.3 %)	4.8 % (0.1 % to 9.5 %)	0.6 % (-4.8 % to 6.1 %)	3.5 % (-1.2 % to 8.2 %)	-0.1 % (-6.2 % to 6.1 %)	8.2 % (0.8 % to 15.6 %)
3.1 % (0.4 % to 5.8 %)	3.9 % (0.6 % to 7.3 %)	-0.2 % (-4.5 % to 4 %)	2.6 % (-0.7 % to 5.9 %)	-1 % (-6.2 % to 4.2 %)	7.3 % (0.7 % to 13.9 %)

Verbal

In [89]:

```
ch_sensi$F3verbal_cat <- as.numeric(ch_sensi$F3verbal_cat)

mem <- ch_sensi %>% filter(!is.na(F3verbal_cat))

msm.totdairy <- geeglm(F3verbal_cat ~ total_dairy_avg, data=mem, weights=ipw_totda
                       corstr="independence")
SEtotal <- coef(summary(msm.totdairy))[,2]

msm.fermdairy <- geeglm(F3verbal_cat ~ ferm_dairy_avg, data=mem, weights=ipw_fermd
                to corstr="independence")
SEferm <- coef(summary(msm.fermdairy))[,2]

msm.nonfermdairy <- geeglm(F3verbal_cat ~ nonferm_dairy_avg, data=mem, weights=ipw_
                corstr="independence")
SEnonferm <- coef(summary(msm.nonfermdairy))[,2]

msm.ffdairy <- geeglm(F3verbal_cat ~ fullfat_dairy_avg, data=mem, weights=ipw_full
                corstr="independence")
SEff <- coef(summary(msm.ffdairy))[,2]

msm.nfdairy <- geeglm(F3verbal_cat ~ nonfat_dairy_avg, data=mem, weights=ipw_nonfa
                corstr="independence")
SEnf <- coef(summary(msm.nfdairy))[,2]

msm.sugdairy <- geeglm(F3verbal_cat ~ sugar_dairy_avg, data=mem, weights=ipw_sugda
                corstr="independence")
SEsug <- coef(summary(msm.sugdairy))[,2]

msm.meat <- geeglm(F3verbal_cat ~ meat_avg, data=mem, weights=ipw_meat, id=pt,
                corstr="independence")
SEmeat <- coef(summary(msm.meat))[,2]

msm.sea <- geeglm(F3verbal_cat ~ sea_avg, data=mem, weights=ipw_sea, id=pt,
                corstr="independence")
SEsea <- coef(summary(msm.sea))[,2]


msm.veg <- geeglm(F3verbal_cat ~ veg_avg, data=mem, weights=ipw_veg, id=pt,
                corstr="independence")
SEveg <- coef(summary(msm.veg))[,2]
```

```
msm.fru <- geeglm(F3verbal_cat ~ fru_avg, data=mem, weights=ipw_fru, id=pt,
                corstr="independence")
SEfru <- coef(summary(msm.fru))[,2]


msm.eggs <- geeglm(F3verbal_cat ~ eggs_avg, data=mem, weights=ipw_eggs, id=pt,
                corstr="independence")
SEeggs <- coef(summary(msm.eggs))[,2]


a <- ((coef(msm.totdairy) - coef(msm.meat))*100)[2]
b <- ((coef(msm.totdairy) - coef(msm.meat) - qnorm(0.975) * sqrt(SEtotal^2 + SEmea
c <- ((coef(msm.totdairy) - coef(msm.meat) + qnorm(0.975) * sqrt(SEtotal^2 + SEmea

d <- ((coef(msm.totdairy) - coef(msm.sea))*100)[2]
e <- ((coef(msm.totdairy) - coef(msm.sea) - qnorm(0.975) * sqrt(SEtotal^2 + SEsea^
f <- ((coef(msm.totdairy) - coef(msm.sea) + qnorm(0.975) * sqrt(SEtotal^2 + SEsea^

g <- ((coef(msm.totdairy) - coef(msm.eggs))*100)[2]
h <- ((coef(msm.totdairy) - coef(msm.eggs) - qnorm(0.975) * sqrt(SEtotal^2 + SEegg
i <- ((coef(msm.totdairy) - coef(msm.eggs) + qnorm(0.975) * sqrt(SEtotal^2 + SEegg

j <- ((coef(msm.totdairy) - coef(msm.veg))*100)[2]
k <- ((coef(msm.totdairy) - coef(msm.veg) - qnorm(0.975) * sqrt(SEtotal^2 + SEveg^
l <- ((coef(msm.totdairy) - coef(msm.veg) + qnorm(0.975) * sqrt(SEtotal^2 + SEveg^

m <- ((coef(msm.totdairy) - coef(msm.fru))*100)[2]
n <- ((coef(msm.totdairy) - coef(msm.fru) - qnorm(0.975) * sqrt(SEtotal^2 + SEfru^
o <- ((coef(msm.totdairy) - coef(msm.fru) + qnorm(0.975) * sqrt(SEtotal^2 + SEfru^

verbaltotal <- c(paste(round(a,3)*100, "% (", round(b,3)*100,"% to ", round(c,3)*1
              paste(round(d,3)*100, "% (", round(e,3)*100,"% to ", round(f,3)*100,
              paste(round(g,3)*100, "% (", round(h,3)*100,"% to ", round(i,3)*100,
              paste(round(j,3)*100, "% (", round(k,3)*100,"% to ", round(l,3)*100,
              paste(round(m,3)*100, "% (", round(n,3)*100,"% to ", round(o,3)*100,

a <- ((coef(msm.fermdairy) - coef(msm.meat))*100)[2]
b <- ((coef(msm.fermdairy) - coef(msm.meat) - qnorm(0.975) * sqrt(SEferm^2 + SEmea
c <- ((coef(msm.fermdairy) - coef(msm.meat) + qnorm(0.975) * sqrt(SEferm^2 + SEmea

d <- ((coef(msm.fermdairy) - coef(msm.sea))*100)[2]
e <- ((coef(msm.fermdairy) - coef(msm.sea) - qnorm(0.975) * sqrt(SEferm^2 + SEsea^
f <- ((coef(msm.fermdairy) - coef(msm.sea) + qnorm(0.975) * sqrt(SEferm^2 + SEsea^

g <- ((coef(msm.fermdairy) - coef(msm.eggs))*100)[2]
h <- ((coef(msm.fermdairy) - coef(msm.eggs) - qnorm(0.975) * sqrt(SEferm^2 + SEegg
i <- ((coef(msm.fermdairy) - coef(msm.eggs) + qnorm(0.975) * sqrt(SEferm^2 + SEegg

j <- ((coef(msm.fermdairy) - coef(msm.veg))*100)[2]
k <- ((coef(msm.fermdairy) - coef(msm.veg) - qnorm(0.975) * sqrt(SEferm^2 + SEveg^
l <- ((coef(msm.fermdairy) - coef(msm.veg) + qnorm(0.975) * sqrt(SEferm^2 + SEveg^

m <- ((coef(msm.fermdairy) - coef(msm.fru))*100)[2]
n <- ((coef(msm.fermdairy) - coef(msm.fru) - qnorm(0.975) * sqrt(SEferm^2 + SEfru^
o <- ((coef(msm.fermdairy) - coef(msm.fru) + qnorm(0.975) * sqrt(SEferm^2 + SEfru^

verbalferm <- c(paste(round(a,3)*100, "% (", round(b,3)*100,"% to ", round(c,3)*10
              paste(round(d,3)*100, "% (", round(e,3)*100,"% to ", round(f,3)*100,
              paste(round(g,3)*100, "% (", round(h,3)*100,"% to ", round(i,3)*100,
              paste(round(j,3)*100, "% (", round(k,3)*100,"% to ", round(l,3)*100,
              paste(round(m,3)*100, "% (", round(n,3)*100,"% to ", round(o,3)*100,

a <- ((coef(msm.nonfermdairy) - coef(msm.meat))*100)[2]
```

```
b <- ((coef(msm.nonfermdairy) - coef(msm.meat) - qnorm(0.975) * sqrt(SEnonferm^2 +
c <- ((coef(msm.nonfermdairy) - coef(msm.meat) + qnorm(0.975) * sqrt(SEnonferm^2 +

d <- ((coef(msm.nonfermdairy) - coef(msm.sea))*100)[2]
e <- ((coef(msm.nonfermdairy) - coef(msm.sea) - qnorm(0.975) * sqrt(SEnonferm^2 + 
f <- ((coef(msm.nonfermdairy) - coef(msm.sea) + qnorm(0.975) * sqrt(SEnonferm^2 + 

g <- ((coef(msm.nonfermdairy) - coef(msm.eggs))*100)[2]
h <- ((coef(msm.nonfermdairy) - coef(msm.eggs) - qnorm(0.975) * sqrt(SEnonferm^2 +
i <- ((coef(msm.nonfermdairy) - coef(msm.eggs) + qnorm(0.975) * sqrt(SEnonferm^2 +

j <- ((coef(msm.nonfermdairy) - coef(msm.veg))*100)[2]
k <- ((coef(msm.nonfermdairy) - coef(msm.veg) - qnorm(0.975) * sqrt(SEnonferm^2 + 
l <- ((coef(msm.nonfermdairy) - coef(msm.veg) + qnorm(0.975) * sqrt(SEnonferm^2 + 

m <- ((coef(msm.nonfermdairy) - coef(msm.fru))*100)[2]
n <- ((coef(msm.nonfermdairy) - coef(msm.fru) - qnorm(0.975) * sqrt(SEnonferm^2 + 
o <- ((coef(msm.nonfermdairy) - coef(msm.fru) + qnorm(0.975) * sqrt(SEnonferm^2 + 

verbalnonferm <- c(paste(round(a,3)*100, "% (", round(b,3)*100,"% to ", round(c,3)
              paste(round(d,3)*100, "% (", round(e,3)*100,"% to ", round(f,3)*100,
              paste(round(g,3)*100, "% (", round(h,3)*100,"% to ", round(i,3)*100,
              paste(round(j,3)*100, "% (", round(k,3)*100,"% to ", round(l,3)*100,
              paste(round(m,3)*100, "% (", round(n,3)*100,"% to ", round(o,3)*100,


a <- ((coef(msm.ffdairy) - coef(msm.meat))*100)[2]
b <- ((coef(msm.ffdairy) - coef(msm.meat) - qnorm(0.975) * sqrt(SEff^2 + SEmeat^2)
c <- ((coef(msm.ffdairy) - coef(msm.meat) + qnorm(0.975) * sqrt(SEff^2 + SEmeat^2)

d <- ((coef(msm.ffdairy) - coef(msm.sea))*100)[2]
e <- ((coef(msm.ffdairy) - coef(msm.sea) - qnorm(0.975) * sqrt(SEff^2 + SEsea^2))*
f <- ((coef(msm.ffdairy) - coef(msm.sea) + qnorm(0.975) * sqrt(SEff^2 + SEsea^2))*

g <- ((coef(msm.ffdairy) - coef(msm.eggs))*100)[2]
h <- ((coef(msm.ffdairy) - coef(msm.eggs) - qnorm(0.975) * sqrt(SEff^2 + SEeggs^2)
i <- ((coef(msm.ffdairy) - coef(msm.eggs) + qnorm(0.975) * sqrt(SEff^2 + SEeggs^2)

j <- ((coef(msm.ffdairy) - coef(msm.veg))*100)[2]
k <- ((coef(msm.ffdairy) - coef(msm.veg) - qnorm(0.975) * sqrt(SEff^2 + SEveg^2))*
l <- ((coef(msm.ffdairy) - coef(msm.veg) + qnorm(0.975) * sqrt(SEff^2 + SEveg^2))*

m <- ((coef(msm.ffdairy) - coef(msm.fru))*100)[2]
n <- ((coef(msm.ffdairy) - coef(msm.fru) - qnorm(0.975) * sqrt(SEff^2 + SEfru^2))*
o <- ((coef(msm.ffdairy) - coef(msm.fru) + qnorm(0.975) * sqrt(SEff^2 + SEfru^2))*

verbalfullfat <- c(paste(round(a,3)*100, "% (", round(b,3)*100,"% to ", round(c,3)
              paste(round(d,3)*100, "% (", round(e,3)*100,"% to ", round(f,3)*100,
              paste(round(g,3)*100, "% (", round(h,3)*100,"% to ", round(i,3)*100,
              paste(round(j,3)*100, "% (", round(k,3)*100,"% to ", round(l,3)*100,
              paste(round(m,3)*100, "% (", round(n,3)*100,"% to ", round(o,3)*100,


a <- ((coef(msm.nfdairy) - coef(msm.meat))*100)[2]
b <- ((coef(msm.nfdairy) - coef(msm.meat) - qnorm(0.975) * sqrt(SEnf^2 + SEmeat^2)
c <- ((coef(msm.nfdairy) - coef(msm.meat) + qnorm(0.975) * sqrt(SEnf^2 + SEmeat^2)

d <- ((coef(msm.nfdairy) - coef(msm.sea))*100)[2]
e <- ((coef(msm.nfdairy) - coef(msm.sea) - qnorm(0.975) * sqrt(SEnf^2 + SEsea^2))*
f <- ((coef(msm.nfdairy) - coef(msm.sea) + qnorm(0.975) * sqrt(SEnf^2 + SEsea^2))*

g <- ((coef(msm.nfdairy) - coef(msm.eggs))*100)[2]
h <- ((coef(msm.nfdairy) - coef(msm.eggs) - qnorm(0.975) * sqrt(SEnf^2 + SEeggs^2)
i <- ((coef(msm.nfdairy) - coef(msm.eggs) + qnorm(0.975) * sqrt(SEnf^2 + SEeggs^2)
```

```
j <- ((coef(msm.nfdairy) - coef(msm.veg))*100)[2]
k <- ((coef(msm.nfdairy) - coef(msm.veg) - qnorm(0.975) * sqrt(SEnf^2 + SEveg^2))*
l <- ((coef(msm.nfdairy) - coef(msm.veg) + qnorm(0.975) * sqrt(SEnf^2 + SEveg^2))*

m <- ((coef(msm.nfdairy) - coef(msm.fru))*100)[2]
n <- ((coef(msm.nfdairy) - coef(msm.fru) - qnorm(0.975) * sqrt(SEnf^2 + SEfru^2))*
o <- ((coef(msm.nfdairy) - coef(msm.fru) + qnorm(0.975) * sqrt(SEnf^2 + SEfru^2))*

verbalnonfat <- c(paste(round(a,3)*100, "% (", round(b,3)*100,"% to ", round(c,3)*
              paste(round(d,3)*100, "% (", round(e,3)*100,"% to ", round(f,3)*100,
              paste(round(g,3)*100, "% (", round(h,3)*100,"% to ", round(i,3)*100,
              paste(round(j,3)*100, "% (", round(k,3)*100,"% to ", round(l,3)*100,
              paste(round(m,3)*100, "% (", round(n,3)*100,"% to ", round(o,3)*100,


a <- ((coef(msm.sugdairy) - coef(msm.meat))*100)[2]
b <- ((coef(msm.sugdairy) - coef(msm.meat) - qnorm(0.975) * sqrt(SEsug^2 + SEmeat^
c <- ((coef(msm.sugdairy) - coef(msm.meat) + qnorm(0.975) * sqrt(SEsug^2 + SEmeat^

d <- ((coef(msm.sugdairy) - coef(msm.sea))*100)[2]
e <- ((coef(msm.sugdairy) - coef(msm.sea) - qnorm(0.975) * sqrt(SEsug^2 + SEsea^2)
f <- ((coef(msm.sugdairy) - coef(msm.sea) + qnorm(0.975) * sqrt(SEsug^2 + SEsea^2)

g <- ((coef(msm.sugdairy) - coef(msm.eggs))*100)[2]
h <- ((coef(msm.sugdairy) - coef(msm.eggs) - qnorm(0.975) * sqrt(SEsug^2 + SEeggs^
i <- ((coef(msm.sugdairy) - coef(msm.eggs) + qnorm(0.975) * sqrt(SEsug^2 + SEeggs^

j <- ((coef(msm.sugdairy) - coef(msm.veg))*100)[2]
k <- ((coef(msm.sugdairy) - coef(msm.veg) - qnorm(0.975) * sqrt(SEsug^2 + SEveg^2)
l <- ((coef(msm.sugdairy) - coef(msm.veg) + qnorm(0.975) * sqrt(SEsug^2 + SEveg^2)

m <- ((coef(msm.sugdairy) - coef(msm.fru))*100)[2]
n <- ((coef(msm.sugdairy) - coef(msm.fru) - qnorm(0.975) * sqrt(SEsug^2 + SEfru^2)
o <- ((coef(msm.sugdairy) - coef(msm.fru) + qnorm(0.975) * sqrt(SEsug^2 + SEfru^2)

verbalsug <- c(paste(round(a,3)*100, "% (", round(b,3)*100,"% to ", round(c,3)*100
              paste(round(d,3)*100, "% (", round(e,3)*100,"% to ", round(f,3)*100,
              paste(round(g,3)*100, "% (", round(h,3)*100,"% to ", round(i,3)*100,
              paste(round(j,3)*100, "% (", round(k,3)*100,"% to ", round(l,3)*100,
              paste(round(m,3)*100, "% (", round(n,3)*100,"% to ", round(o,3)*100,

VERBAL <- data.frame(verbaltotal,verbalferm, verbalnonferm, verbalfullfat, verbaln
VERBAL
```

A data.frame: 5 × 6

verbaltotal	verbalferm	verbalnonferm	verbalfullfat	verbalnonfat	verbalsug
<chr>	<chr>	<chr>	<chr>	<chr>	<chr>
0.7 % (-13.9 % to 15.2 %)	-1.5 % (-16.3 % to 13.4 %)	2.9 % (-12.3 % to 18.1 %)	-0.9 % (-15.6 % to 13.8 %)	1.9 % (-15 % to 18.7 %)	-0.8 % (-17.3 % to 15.7 %)
0.6 % (-30.7 % to 31.8 %)	-1.6 % (-33 % to 29.9 %)	2.7 % (-28.8 % to 34.3 %)	-1 % (-32.3 % to 30.3 %)	1.8 % (-30.7 % to 34.2 %)	-0.9 % (-33.2 % to 31.3 %)
25.3 % (-24.7 % to 75.3 %)	23.2 % (-26.9 % to 73.3 %)	27.5 % (-22.7 % to 77.7 %)	23.8 % (-26.3 % to 73.8 %)	26.5 % (-24.2 % to 77.3 %)	23.8 % (-26.8 % to 74.5 %)
-2.9 % (-9 % to 3.2 %)	-5.1 % (-12 % to 1.8 %)	-0.8 % (-8.3 % to 6.8 %)	-4.5 % (-10.9 % to 1.9 %)	-1.7 % (-12.2 % to 8.7 %)	-4.4 % (-14.4 % to 5.5 %)
2.8 % (-2.2 % to 7.8 %)	0.7 % (-5.2 % to 6.6 %)	5 % (-1.6 % to 11.7 %)	1.3 % (-4.1 % to 6.6 %)	4 % (-5.8 % to 13.9 %)	1.3 % (-8 % to 10.7 %)

Stroop

In [90]:
```
ch_sensi$F3stroop_cat <- as.numeric(ch_sensi$F3stroop_cat)

mem <- ch_sensi %>% filter(!is.na(F3stroop_cat))

msm.totdairy <- geeglm(F3stroop_cat ~ total_dairy_avg, data=mem, weights=ipw_totda
                       corstr="independence")
SEtotal <- coef(summary(msm.totdairy))[,2]

msm.fermdairy <- geeglm(F3stroop_cat ~ ferm_dairy_avg, data=mem, weights=ipw_fermd
                 corstr="independence")
SEferm <- coef(summary(msm.fermdairy))[,2]

msm.nonfermdairy <- geeglm(F3stroop_cat ~ nonferm_dairy_avg, data=mem, weights=ipw_
                 corstr="independence")
SEnonferm <- coef(summary(msm.nonfermdairy))[,2]

msm.ffdairy <- geeglm(F3stroop_cat ~ fullfat_dairy_avg, data=mem, weights=ipw_full
                 corstr="independence")
SEff <- coef(summary(msm.ffdairy))[,2]

msm.nfdairy <- geeglm(F3stroop_cat ~ nonfat_dairy_avg, data=mem, weights=ipw_nonfa
                 corstr="independence")
SEnf <- coef(summary(msm.nfdairy))[,2]

msm.sugdairy <- geeglm(F3stroop_cat ~ sugar_dairy_avg, data=mem, weights=ipw_sugda
                 corstr="independence")
SEsug <- coef(summary(msm.sugdairy))[,2]

msm.meat <- geeglm(F3stroop_cat ~ meat_avg, data=mem, weights=ipw_meat, id=pt,
                 corstr="independence")
SEmeat <- coef(summary(msm.meat))[,2]

msm.sea <- geeglm(F3stroop_cat ~ sea_avg, data=mem, weights=ipw_sea, id=pt,
                 corstr="independence")
SEsea <- coef(summary(msm.sea))[,2]


msm.veg <- geeglm(F3stroop_cat ~ veg_avg, data=mem, weights=ipw_veg, id=pt,
                 corstr="independence")
SEveg <- coef(summary(msm.veg))[,2]


msm.fru <- geeglm(F3stroop_cat ~ fru_avg, data=mem, weights=ipw_fru, id=pt,
                 corstr="independence")
SEfru <- coef(summary(msm.fru))[,2]


msm.eggs <- geeglm(F3stroop_cat ~ eggs_avg, data=mem, weights=ipw_eggs, id=pt,
                 corstr="independence")
SEeggs <- coef(summary(msm.eggs))[,2]

a <- ((coef(msm.totdairy) - coef(msm.meat))*100)[2]
b <- ((coef(msm.totdairy) - coef(msm.meat) - qnorm(0.975) * sqrt(SEtotal^2 + SEmea
c <- ((coef(msm.totdairy) - coef(msm.meat) + qnorm(0.975) * sqrt(SEtotal^2 + SEmea

d <- ((coef(msm.totdairy) - coef(msm.sea))*100)[2]
e <- ((coef(msm.totdairy) - coef(msm.sea) - qnorm(0.975) * sqrt(SEtotal^2 + SEsea^
f <- ((coef(msm.totdairy) - coef(msm.sea) + qnorm(0.975) * sqrt(SEtotal^2 + SEsea^

g <- ((coef(msm.totdairy) - coef(msm.eggs))*100)[2]
```

```
h <- ((coef(msm.totdairy) - coef(msm.eggs) - qnorm(0.975) * sqrt(SEtotal^2 + SEegg
i <- ((coef(msm.totdairy) - coef(msm.eggs) + qnorm(0.975) * sqrt(SEtotal^2 + SEegg

j <- ((coef(msm.totdairy) - coef(msm.veg))*100)[2]
k <- ((coef(msm.totdairy) - coef(msm.veg) - qnorm(0.975) * sqrt(SEtotal^2 + SEveg^
l <- ((coef(msm.totdairy) - coef(msm.veg) + qnorm(0.975) * sqrt(SEtotal^2 + SEveg^

m <- ((coef(msm.totdairy) - coef(msm.fru))*100)[2]
n <- ((coef(msm.totdairy) - coef(msm.fru) - qnorm(0.975) * sqrt(SEtotal^2 + SEfru^
o <- ((coef(msm.totdairy) - coef(msm.fru) + qnorm(0.975) * sqrt(SEtotal^2 + SEfru^

strooptotal <- c(paste(round(a,3)*100, "% (", round(b,3)*100,"% to ", round(c,3)*1
              paste(round(d,3)*100, "% (", round(e,3)*100,"% to ", round(f,3)*100,
              paste(round(g,3)*100, "% (", round(h,3)*100,"% to ", round(i,3)*100,
              paste(round(j,3)*100, "% (", round(k,3)*100,"% to ", round(l,3)*100,
              paste(round(m,3)*100, "% (", round(n,3)*100,"% to ", round(o,3)*100,

a <- ((coef(msm.fermdairy) - coef(msm.meat))*100)[2]
b <- ((coef(msm.fermdairy) - coef(msm.meat) - qnorm(0.975) * sqrt(SEferm^2 + SEmea
c <- ((coef(msm.fermdairy) - coef(msm.meat) + qnorm(0.975) * sqrt(SEferm^2 + SEmea

d <- ((coef(msm.fermdairy) - coef(msm.sea))*100)[2]
e <- ((coef(msm.fermdairy) - coef(msm.sea) - qnorm(0.975) * sqrt(SEferm^2 + SEsea^
f <- ((coef(msm.fermdairy) - coef(msm.sea) + qnorm(0.975) * sqrt(SEferm^2 + SEsea^

g <- ((coef(msm.fermdairy) - coef(msm.eggs))*100)[2]
h <- ((coef(msm.fermdairy) - coef(msm.eggs) - qnorm(0.975) * sqrt(SEferm^2 + SEegg
i <- ((coef(msm.fermdairy) - coef(msm.eggs) + qnorm(0.975) * sqrt(SEferm^2 + SEegg

j <- ((coef(msm.fermdairy) - coef(msm.veg))*100)[2]
k <- ((coef(msm.fermdairy) - coef(msm.veg) - qnorm(0.975) * sqrt(SEferm^2 + SEveg^
l <- ((coef(msm.fermdairy) - coef(msm.veg) + qnorm(0.975) * sqrt(SEferm^2 + SEveg^

m <- ((coef(msm.fermdairy) - coef(msm.fru))*100)[2]
n <- ((coef(msm.fermdairy) - coef(msm.fru) - qnorm(0.975) * sqrt(SEferm^2 + SEfru^
o <- ((coef(msm.fermdairy) - coef(msm.fru) + qnorm(0.975) * sqrt(SEferm^2 + SEfru^

stroopferm <- c(paste(round(a,3)*100, "% (", round(b,3)*100,"% to ", round(c,3)*10
              paste(round(d,3)*100, "% (", round(e,3)*100,"% to ", round(f,3)*100,
              paste(round(g,3)*100, "% (", round(h,3)*100,"% to ", round(i,3)*100,
              paste(round(j,3)*100, "% (", round(k,3)*100,"% to ", round(l,3)*100,
              paste(round(m,3)*100, "% (", round(n,3)*100,"% to ", round(o,3)*100,

a <- ((coef(msm.nonfermdairy) - coef(msm.meat))*100)[2]
b <- ((coef(msm.nonfermdairy) - coef(msm.meat) - qnorm(0.975) * sqrt(SEnonferm^2 +
c <- ((coef(msm.nonfermdairy) - coef(msm.meat) + qnorm(0.975) * sqrt(SEnonferm^2 +

d <- ((coef(msm.nonfermdairy) - coef(msm.sea))*100)[2]
e <- ((coef(msm.nonfermdairy) - coef(msm.sea) - qnorm(0.975) * sqrt(SEnonferm^2 +
f <- ((coef(msm.nonfermdairy) - coef(msm.sea) + qnorm(0.975) * sqrt(SEnonferm^2 +

g <- ((coef(msm.nonfermdairy) - coef(msm.eggs))*100)[2]
h <- ((coef(msm.nonfermdairy) - coef(msm.eggs) - qnorm(0.975) * sqrt(SEnonferm^2 +
i <- ((coef(msm.nonfermdairy) - coef(msm.eggs) + qnorm(0.975) * sqrt(SEnonferm^2 +

j <- ((coef(msm.nonfermdairy) - coef(msm.veg))*100)[2]
k <- ((coef(msm.nonfermdairy) - coef(msm.veg) - qnorm(0.975) * sqrt(SEnonferm^2 +
l <- ((coef(msm.nonfermdairy) - coef(msm.veg) + qnorm(0.975) * sqrt(SEnonferm^2 +

m <- ((coef(msm.nonfermdairy) - coef(msm.fru))*100)[2]
n <- ((coef(msm.nonfermdairy) - coef(msm.fru) - qnorm(0.975) * sqrt(SEnonferm^2 +
o <- ((coef(msm.nonfermdairy) - coef(msm.fru) + qnorm(0.975) * sqrt(SEnonferm^2 +

stroopnonferm <- c(paste(round(a,3)*100, "% (", round(b,3)*100,"% to ", round(c,3)
```

```
              paste(round(d,3)*100, "% (", round(e,3)*100,"% to ", round(f,3)*100,
              paste(round(g,3)*100, "% (", round(h,3)*100,"% to ", round(i,3)*100,
              paste(round(j,3)*100, "% (", round(k,3)*100,"% to ", round(l,3)*100,
              paste(round(m,3)*100, "% (", round(n,3)*100,"% to ", round(o,3)*100,


a <- ((coef(msm.ffdairy) - coef(msm.meat))*100)[2]
b <- ((coef(msm.ffdairy) - coef(msm.meat) - qnorm(0.975) * sqrt(SEff^2 + SEmeat^2)
c <- ((coef(msm.ffdairy) - coef(msm.meat) + qnorm(0.975) * sqrt(SEff^2 + SEmeat^2)

d <- ((coef(msm.ffdairy) - coef(msm.sea))*100)[2]
e <- ((coef(msm.ffdairy) - coef(msm.sea) - qnorm(0.975) * sqrt(SEff^2 + SEsea^2))*
f <- ((coef(msm.ffdairy) - coef(msm.sea) + qnorm(0.975) * sqrt(SEff^2 + SEsea^2))*

g <- ((coef(msm.ffdairy) - coef(msm.eggs))*100)[2]
h <- ((coef(msm.ffdairy) - coef(msm.eggs) - qnorm(0.975) * sqrt(SEff^2 + SEeggs^2)
i <- ((coef(msm.ffdairy) - coef(msm.eggs) + qnorm(0.975) * sqrt(SEff^2 + SEeggs^2)

j <- ((coef(msm.ffdairy) - coef(msm.veg))*100)[2]
k <- ((coef(msm.ffdairy) - coef(msm.veg) - qnorm(0.975) * sqrt(SEff^2 + SEveg^2))*
l <- ((coef(msm.ffdairy) - coef(msm.veg) + qnorm(0.975) * sqrt(SEff^2 + SEveg^2))*

m <- ((coef(msm.ffdairy) - coef(msm.fru))*100)[2]
n <- ((coef(msm.ffdairy) - coef(msm.fru) - qnorm(0.975) * sqrt(SEff^2 + SEfru^2))*
o <- ((coef(msm.ffdairy) - coef(msm.fru) + qnorm(0.975) * sqrt(SEff^2 + SEfru^2))*

stroopfullfat <- c(paste(round(a,3)*100, "% (", round(b,3)*100,"% to ", round(c,3)
              paste(round(d,3)*100, "% (", round(e,3)*100,"% to ", round(f,3)*100,
              paste(round(g,3)*100, "% (", round(h,3)*100,"% to ", round(i,3)*100,
              paste(round(j,3)*100, "% (", round(k,3)*100,"% to ", round(l,3)*100,
              paste(round(m,3)*100, "% (", round(n,3)*100,"% to ", round(o,3)*100,


a <- ((coef(msm.nfdairy) - coef(msm.meat))*100)[2]
b <- ((coef(msm.nfdairy) - coef(msm.meat) - qnorm(0.975) * sqrt(SEnf^2 + SEmeat^2)
c <- ((coef(msm.nfdairy) - coef(msm.meat) + qnorm(0.975) * sqrt(SEnf^2 + SEmeat^2)

d <- ((coef(msm.nfdairy) - coef(msm.sea))*100)[2]
e <- ((coef(msm.nfdairy) - coef(msm.sea) - qnorm(0.975) * sqrt(SEnf^2 + SEsea^2))*
f <- ((coef(msm.nfdairy) - coef(msm.sea) + qnorm(0.975) * sqrt(SEnf^2 + SEsea^2))*

g <- ((coef(msm.nfdairy) - coef(msm.eggs))*100)[2]
h <- ((coef(msm.nfdairy) - coef(msm.eggs) - qnorm(0.975) * sqrt(SEnf^2 + SEeggs^2)
i <- ((coef(msm.nfdairy) - coef(msm.eggs) + qnorm(0.975) * sqrt(SEnf^2 + SEeggs^2)

j <- ((coef(msm.nfdairy) - coef(msm.veg))*100)[2]
k <- ((coef(msm.nfdairy) - coef(msm.veg) - qnorm(0.975) * sqrt(SEnf^2 + SEveg^2))*
l <- ((coef(msm.nfdairy) - coef(msm.veg) + qnorm(0.975) * sqrt(SEnf^2 + SEveg^2))*

m <- ((coef(msm.nfdairy) - coef(msm.fru))*100)[2]
n <- ((coef(msm.nfdairy) - coef(msm.fru) - qnorm(0.975) * sqrt(SEnf^2 + SEfru^2))*
o <- ((coef(msm.nfdairy) - coef(msm.fru) + qnorm(0.975) * sqrt(SEnf^2 + SEfru^2))*

stroopnonfat <- c(paste(round(a,3)*100, "% (", round(b,3)*100,"% to ", round(c,3)*
              paste(round(d,3)*100, "% (", round(e,3)*100,"% to ", round(f,3)*100,
              paste(round(g,3)*100, "% (", round(h,3)*100,"% to ", round(i,3)*100,
              paste(round(j,3)*100, "% (", round(k,3)*100,"% to ", round(l,3)*100,
              paste(round(m,3)*100, "% (", round(n,3)*100,"% to ", round(o,3)*100,


a <- ((coef(msm.sugdairy) - coef(msm.meat))*100)[2]
b <- ((coef(msm.sugdairy) - coef(msm.meat) - qnorm(0.975) * sqrt(SEsug^2 + SEmeat^
c <- ((coef(msm.sugdairy) - coef(msm.meat) + qnorm(0.975) * sqrt(SEsug^2 + SEmeat^
```

```
d <- ((coef(msm.sugdairy) - coef(msm.sea))*100)[2]
e <- ((coef(msm.sugdairy) - coef(msm.sea) - qnorm(0.975) * sqrt(SEsug^2 + SEsea^2)
f <- ((coef(msm.sugdairy) - coef(msm.sea) + qnorm(0.975) * sqrt(SEsug^2 + SEsea^2)

g <- ((coef(msm.sugdairy) - coef(msm.eggs))*100)[2]
h <- ((coef(msm.sugdairy) - coef(msm.eggs) - qnorm(0.975) * sqrt(SEsug^2 + SEeggs^
i <- ((coef(msm.sugdairy) - coef(msm.eggs) + qnorm(0.975) * sqrt(SEsug^2 + SEeggs^

j <- ((coef(msm.sugdairy) - coef(msm.veg))*100)[2]
k <- ((coef(msm.sugdairy) - coef(msm.veg) - qnorm(0.975) * sqrt(SEsug^2 + SEveg^2)
l <- ((coef(msm.sugdairy) - coef(msm.veg) + qnorm(0.975) * sqrt(SEsug^2 + SEveg^2)

m <- ((coef(msm.sugdairy) - coef(msm.fru))*100)[2]
n <- ((coef(msm.sugdairy) - coef(msm.fru) - qnorm(0.975) * sqrt(SEsug^2 + SEfru^2)
o <- ((coef(msm.sugdairy) - coef(msm.fru) + qnorm(0.975) * sqrt(SEsug^2 + SEfru^2)

stroopsug <- c(paste(round(a,3)*100, "% (", round(b,3)*100,"% to ", round(c,3)*100
               paste(round(d,3)*100, "% (", round(e,3)*100,"% to ", round(f,3)*100,
               paste(round(g,3)*100, "% (", round(h,3)*100,"% to ", round(i,3)*100,
               paste(round(j,3)*100, "% (", round(k,3)*100,"% to ", round(l,3)*100,
               paste(round(m,3)*100, "% (", round(n,3)*100,"% to ", round(o,3)*100,

STROOP <- data.frame(strooptotal,stroopferm, stroopnonferm, stroopfullfat, stroopn

STROOP
```

A data.frame: 5 × 6

strooptotal	stroopferm	stroopnonferm	stroopfullfat	stroopnonfat	stroopsug
<chr>	<chr>	<chr>	<chr>	<chr>	<chr>
-0.5 % (-15.3 % to 14.3 %)	-1.7 % (-16.7 % to 13.4 %)	-2.7 % (-18 % to 12.6 %)	-0.8 % (-15.7 % to 14.1 %)	0.6 % (-15.7 % to 16.9 %)	-2.3 % (-18.2 % to 13.7 %)
2.9 % (-24.9 % to 30.6 %)	1.7 % (-26.2 % to 29.6 %)	0.7 % (-27.3 % to 28.7 %)	2.6 % (-25.2 % to 30.4 %)	4 % (-24.6 % to 32.6 %)	1.1 % (-27.3 % to 29.5 %)
-50.9 % (-100.9 % to -1 %)	-52.1 % (-102.1 % to -2.1 %)	-53.1 % (-103.2 % to -3 %)	-51.2 % (-101.2 % to -1.3 %)	-49.8 % (-100.2 % to 0.6 %)	-52.7 % (-103 % to -2.4 %)
-0.2 % (-5.7 % to 5.3 %)	-1.4 % (-7.5 % to 4.7 %)	-2.4 % (-9.1 % to 4.3 %)	-0.5 % (-6.2 % to 5.2 %)	0.9 % (-7.8 % to 9.7 %)	-2 % (-10.1 % to 6.1 %)
0 % (-4.1 % to 4.2 %)	-1.1 % (-6.1 % to 3.8 %)	-2.1 % (-7.8 % to 3.6 %)	-0.3 % (-4.7 % to 4.2 %)	1.2 % (-6.8 % to 9.2 %)	-1.7 % (-9 % to 5.5 %)

DO40

In [91]:

```
ch_sensi$F3do40_cat <- as.numeric(ch_sensi$F3do40_cat)

mem <- ch_sensi %>% filter(!is.na(F3do40_cat))

msm.totdairy <- geeglm(F3do40_cat ~ total_dairy_avg, data=mem, weights=ipw_totdair
                       corstr="independence")
SEtotal <- coef(summary(msm.totdairy))[,2]

msm.fermdairy <- geeglm(F3do40_cat ~ ferm_dairy_avg, data=mem, weights=ipw_fermdai
                        corstr="independence")
SEferm <- coef(summary(msm.fermdairy))[,2]

msm.nonfermdairy <- geeglm(F3do40_cat ~ nonferm_dairy_avg, data=mem, weights=ipw_n
                           corstr="independence")
SEnonferm <- coef(summary(msm.nonfermdairy))[,2]
```

```
msm.ffdairy <- geeglm(F3do40_cat ~ fullfat_dairy_avg, data=mem, weights=ipw_fullfat
                corstr="independence")
SEff <- coef(summary(msm.ffdairy))[,2]

msm.nfdairy <- geeglm(F3do40_cat ~ nonfat_dairy_avg, data=mem, weights=ipw_nonfatd
                corstr="independence")
SEnf <- coef(summary(msm.nfdairy))[,2]

msm.sugdairy <- geeglm(F3do40_cat ~ sugar_dairy_avg, data=mem, weights=ipw_sugdair
                corstr="independence")
SEsug <- coef(summary(msm.sugdairy))[,2]

msm.meat <- geeglm(F3do40_cat ~ meat_avg, data=mem, weights=ipw_meat, id=pt,
                corstr="independence")
SEmeat <- coef(summary(msm.meat))[,2]

msm.sea <- geeglm(F3do40_cat ~ sea_avg, data=mem, weights=ipw_sea, id=pt,
                corstr="independence")
SEsea <- coef(summary(msm.sea))[,2]


msm.veg <- geeglm(F3do40_cat ~ veg_avg, data=mem, weights=ipw_veg, id=pt,
                corstr="independence")
SEveg <- coef(summary(msm.veg))[,2]


msm.fru <- geeglm(F3do40_cat ~ fru_avg, data=mem, weights=ipw_fru, id=pt,
                corstr="independence")
SEfru <- coef(summary(msm.fru))[,2]


msm.eggs <- geeglm(F3do40_cat ~ eggs_avg, data=mem, weights=ipw_eggs, id=pt,
                corstr="independence")
SEeggs <- coef(summary(msm.eggs))[,2]


a <- ((coef(msm.totdairy) - coef(msm.meat))*100)[2]
b <- ((coef(msm.totdairy) - coef(msm.meat) - qnorm(0.975) * sqrt(SEtotal^2 + SEmea
c <- ((coef(msm.totdairy) - coef(msm.meat) + qnorm(0.975) * sqrt(SEtotal^2 + SEmea

d <- ((coef(msm.totdairy) - coef(msm.sea))*100)[2]
e <- ((coef(msm.totdairy) - coef(msm.sea) - qnorm(0.975) * sqrt(SEtotal^2 + SEsea^
f <- ((coef(msm.totdairy) - coef(msm.sea) + qnorm(0.975) * sqrt(SEtotal^2 + SEsea^

g <- ((coef(msm.totdairy) - coef(msm.eggs))*100)[2]
h <- ((coef(msm.totdairy) - coef(msm.eggs) - qnorm(0.975) * sqrt(SEtotal^2 + SEegg
i <- ((coef(msm.totdairy) - coef(msm.eggs) + qnorm(0.975) * sqrt(SEtotal^2 + SEegg

j <- ((coef(msm.totdairy) - coef(msm.veg))*100)[2]
k <- ((coef(msm.totdairy) - coef(msm.veg) - qnorm(0.975) * sqrt(SEtotal^2 + SEveg^
l <- ((coef(msm.totdairy) - coef(msm.veg) + qnorm(0.975) * sqrt(SEtotal^2 + SEveg^

m <- ((coef(msm.totdairy) - coef(msm.fru))*100)[2]
n <- ((coef(msm.totdairy) - coef(msm.fru) - qnorm(0.975) * sqrt(SEtotal^2 + SEfru^
o <- ((coef(msm.totdairy) - coef(msm.fru) + qnorm(0.975) * sqrt(SEtotal^2 + SEfru^

do40total <- c(paste(round(a,3)*100, "% (", round(b,3)*100,"% to ", round(c,3)*100
              paste(round(d,3)*100, "% (", round(e,3)*100,"% to ", round(f,3)*100,
              paste(round(g,3)*100, "% (", round(h,3)*100,"% to ", round(i,3)*100,
              paste(round(j,3)*100, "% (", round(k,3)*100,"% to ", round(l,3)*100,
              paste(round(m,3)*100, "% (", round(n,3)*100,"% to ", round(o,3)*100,

a <- ((coef(msm.fermdairy) - coef(msm.meat))*100)[2]
```

```
b <- ((coef(msm.fermdairy) - coef(msm.meat) - qnorm(0.975) * sqrt(SEferm^2 + SEmea
c <- ((coef(msm.fermdairy) - coef(msm.meat) + qnorm(0.975) * sqrt(SEferm^2 + SEmea

d <- ((coef(msm.fermdairy) - coef(msm.sea))*100)[2]
e <- ((coef(msm.fermdairy) - coef(msm.sea) - qnorm(0.975) * sqrt(SEferm^2 + SEsea^
f <- ((coef(msm.fermdairy) - coef(msm.sea) + qnorm(0.975) * sqrt(SEferm^2 + SEsea^

g <- ((coef(msm.fermdairy) - coef(msm.eggs))*100)[2]
h <- ((coef(msm.fermdairy) - coef(msm.eggs) - qnorm(0.975) * sqrt(SEferm^2 + SEegg
i <- ((coef(msm.fermdairy) - coef(msm.eggs) + qnorm(0.975) * sqrt(SEferm^2 + SEegg

j <- ((coef(msm.fermdairy) - coef(msm.veg))*100)[2]
k <- ((coef(msm.fermdairy) - coef(msm.veg) - qnorm(0.975) * sqrt(SEferm^2 + SEveg^
l <- ((coef(msm.fermdairy) - coef(msm.veg) + qnorm(0.975) * sqrt(SEferm^2 + SEveg^

m <- ((coef(msm.fermdairy) - coef(msm.fru))*100)[2]
n <- ((coef(msm.fermdairy) - coef(msm.fru) - qnorm(0.975) * sqrt(SEferm^2 + SEfru^
o <- ((coef(msm.fermdairy) - coef(msm.fru) + qnorm(0.975) * sqrt(SEferm^2 + SEfru^

do40ferm <- c(paste(round(a,3)*100, "% (", round(b,3)*100,"% to ", round(c,3)*100,
              paste(round(d,3)*100, "% (", round(e,3)*100,"% to ", round(f,3)*100,
              paste(round(g,3)*100, "% (", round(h,3)*100,"% to ", round(i,3)*100,
              paste(round(j,3)*100, "% (", round(k,3)*100,"% to ", round(l,3)*100,
              paste(round(m,3)*100, "% (", round(n,3)*100,"% to ", round(o,3)*100,

a <- ((coef(msm.nonfermdairy) - coef(msm.meat))*100)[2]
b <- ((coef(msm.nonfermdairy) - coef(msm.meat) - qnorm(0.975) * sqrt(SEnonferm^2 +
c <- ((coef(msm.nonfermdairy) - coef(msm.meat) + qnorm(0.975) * sqrt(SEnonferm^2 +

d <- ((coef(msm.nonfermdairy) - coef(msm.sea))*100)[2]
e <- ((coef(msm.nonfermdairy) - coef(msm.sea) - qnorm(0.975) * sqrt(SEnonferm^2 +
f <- ((coef(msm.nonfermdairy) - coef(msm.sea) + qnorm(0.975) * sqrt(SEnonferm^2 +

g <- ((coef(msm.nonfermdairy) - coef(msm.eggs))*100)[2]
h <- ((coef(msm.nonfermdairy) - coef(msm.eggs) - qnorm(0.975) * sqrt(SEnonferm^2 +
i <- ((coef(msm.nonfermdairy) - coef(msm.eggs) + qnorm(0.975) * sqrt(SEnonferm^2 +

j <- ((coef(msm.nonfermdairy) - coef(msm.veg))*100)[2]
k <- ((coef(msm.nonfermdairy) - coef(msm.veg) - qnorm(0.975) * sqrt(SEnonferm^2 +
l <- ((coef(msm.nonfermdairy) - coef(msm.veg) + qnorm(0.975) * sqrt(SEnonferm^2 +

m <- ((coef(msm.nonfermdairy) - coef(msm.fru))*100)[2]
n <- ((coef(msm.nonfermdairy) - coef(msm.fru) - qnorm(0.975) * sqrt(SEnonferm^2 +
o <- ((coef(msm.nonfermdairy) - coef(msm.fru) + qnorm(0.975) * sqrt(SEnonferm^2 +

do40nonferm <- c(paste(round(a,3)*100, "% (", round(b,3)*100,"% to ", round(c,3)*1
              paste(round(d,3)*100, "% (", round(e,3)*100,"% to ", round(f,3)*100,
              paste(round(g,3)*100, "% (", round(h,3)*100,"% to ", round(i,3)*100,
              paste(round(j,3)*100, "% (", round(k,3)*100,"% to ", round(l,3)*100,
              paste(round(m,3)*100, "% (", round(n,3)*100,"% to ", round(o,3)*100,


a <- ((coef(msm.ffdairy) - coef(msm.meat))*100)[2]
b <- ((coef(msm.ffdairy) - coef(msm.meat) - qnorm(0.975) * sqrt(SEff^2 + SEmeat^2)
c <- ((coef(msm.ffdairy) - coef(msm.meat) + qnorm(0.975) * sqrt(SEff^2 + SEmeat^2)

d <- ((coef(msm.ffdairy) - coef(msm.sea))*100)[2]
e <- ((coef(msm.ffdairy) - coef(msm.sea) - qnorm(0.975) * sqrt(SEff^2 + SEsea^2))*
f <- ((coef(msm.ffdairy) - coef(msm.sea) + qnorm(0.975) * sqrt(SEff^2 + SEsea^2))*

g <- ((coef(msm.ffdairy) - coef(msm.eggs))*100)[2]
h <- ((coef(msm.ffdairy) - coef(msm.eggs) - qnorm(0.975) * sqrt(SEff^2 + SEeggs^2)
i <- ((coef(msm.ffdairy) - coef(msm.eggs) + qnorm(0.975) * sqrt(SEff^2 + SEeggs^2)
```

```
j <- ((coef(msm.ffdairy) - coef(msm.veg))*100)[2]
k <- ((coef(msm.ffdairy) - coef(msm.veg) - qnorm(0.975) * sqrt(SEff^2 + SEveg^2))*
l <- ((coef(msm.ffdairy) - coef(msm.veg) + qnorm(0.975) * sqrt(SEff^2 + SEveg^2))*

m <- ((coef(msm.ffdairy) - coef(msm.fru))*100)[2]
n <- ((coef(msm.ffdairy) - coef(msm.fru) - qnorm(0.975) * sqrt(SEff^2 + SEfru^2))*
o <- ((coef(msm.ffdairy) - coef(msm.fru) + qnorm(0.975) * sqrt(SEff^2 + SEfru^2))*

do40fullfat <- c(paste(round(a,3)*100, "% (", round(b,3)*100,"% to ", round(c,3)*1
              paste(round(d,3)*100, "% (", round(e,3)*100,"% to ", round(f,3)*100,
              paste(round(g,3)*100, "% (", round(h,3)*100,"% to ", round(i,3)*100,
              paste(round(j,3)*100, "% (", round(k,3)*100,"% to ", round(l,3)*100,
              paste(round(m,4), "% (", round(n,3)*100,"% to ", round(o,3)*100,"%)"


a <- ((coef(msm.nfdairy) - coef(msm.meat))*100)[2]
b <- ((coef(msm.nfdairy) - coef(msm.meat) - qnorm(0.975) * sqrt(SEnf^2 + SEmeat^2)
c <- ((coef(msm.nfdairy) - coef(msm.meat) + qnorm(0.975) * sqrt(SEnf^2 + SEmeat^2)

d <- ((coef(msm.nfdairy) - coef(msm.sea))*100)[2]
e <- ((coef(msm.nfdairy) - coef(msm.sea) - qnorm(0.975) * sqrt(SEnf^2 + SEsea^2))*
f <- ((coef(msm.nfdairy) - coef(msm.sea) + qnorm(0.975) * sqrt(SEnf^2 + SEsea^2))*

g <- ((coef(msm.nfdairy) - coef(msm.eggs))*100)[2]
h <- ((coef(msm.nfdairy) - coef(msm.eggs) - qnorm(0.975) * sqrt(SEnf^2 + SEeggs^2)
i <- ((coef(msm.nfdairy) - coef(msm.eggs) + qnorm(0.975) * sqrt(SEnf^2 + SEeggs^2)

j <- ((coef(msm.nfdairy) - coef(msm.veg))*100)[2]
k <- ((coef(msm.nfdairy) - coef(msm.veg) - qnorm(0.975) * sqrt(SEnf^2 + SEveg^2))*
l <- ((coef(msm.nfdairy) - coef(msm.veg) + qnorm(0.975) * sqrt(SEnf^2 + SEveg^2))*

m <- ((coef(msm.nfdairy) - coef(msm.fru))*100)[2]
n <- ((coef(msm.nfdairy) - coef(msm.fru) - qnorm(0.975) * sqrt(SEnf^2 + SEfru^2))*
o <- ((coef(msm.nfdairy) - coef(msm.fru) + qnorm(0.975) * sqrt(SEnf^2 + SEfru^2))*

do40nonfat <- c(paste(round(a,3)*100, "% (", round(b,3)*100,"% to ", round(c,3)*10
              paste(round(d,3)*100, "% (", round(e,3)*100,"% to ", round(f,3)*100,
              paste(round(g,3)*100, "% (", round(h,3)*100,"% to ", round(i,3)*100,
              paste(round(j,3)*100, "% (", round(k,3)*100,"% to ", round(l,3)*100,
              paste(round(m,3)*100, "% (", round(n,3)*100,"% to ", round(o,3)*100,


a <- ((coef(msm.sugdairy) - coef(msm.meat))*100)[2]
b <- ((coef(msm.sugdairy) - coef(msm.meat) - qnorm(0.975) * sqrt(SEsug^2 + SEmeat^
c <- ((coef(msm.sugdairy) - coef(msm.meat) + qnorm(0.975) * sqrt(SEsug^2 + SEmeat^

d <- ((coef(msm.sugdairy) - coef(msm.sea))*100)[2]
e <- ((coef(msm.sugdairy) - coef(msm.sea) - qnorm(0.975) * sqrt(SEsug^2 + SEsea^2)
f <- ((coef(msm.sugdairy) - coef(msm.sea) + qnorm(0.975) * sqrt(SEsug^2 + SEsea^2)

g <- ((coef(msm.sugdairy) - coef(msm.eggs))*100)[2]
h <- ((coef(msm.sugdairy) - coef(msm.eggs) - qnorm(0.975) * sqrt(SEsug^2 + SEeggs^
i <- ((coef(msm.sugdairy) - coef(msm.eggs) + qnorm(0.975) * sqrt(SEsug^2 + SEeggs^

j <- ((coef(msm.sugdairy) - coef(msm.veg))*100)[2]
k <- ((coef(msm.sugdairy) - coef(msm.veg) - qnorm(0.975) * sqrt(SEsug^2 + SEveg^2)
l <- ((coef(msm.sugdairy) - coef(msm.veg) + qnorm(0.975) * sqrt(SEsug^2 + SEveg^2)

m <- ((coef(msm.sugdairy) - coef(msm.fru))*100)[2]
n <- ((coef(msm.sugdairy) - coef(msm.fru) - qnorm(0.975) * sqrt(SEsug^2 + SEfru^2)
o <- ((coef(msm.sugdairy) - coef(msm.fru) + qnorm(0.975) * sqrt(SEsug^2 + SEfru^2)

do40sug <- c(paste(round(a,3)*100, "% (", round(b,3)*100,"% to ", round(c,3)*100,"
              paste(round(d,3)*100, "% (", round(e,3)*100,"% to ", round(f,3)*100,
```

```
                    paste(round(g,3)*100, "% (", round(h,3)*100,"% to ", round(i,3)*100,
                    paste(round(j,3)*100, "% (", round(k,3)*100,"% to ", round(l,3)*100,
                    paste(round(m,3)*100, "% (", round(n,3)*100,"% to ", round(o,3)*100,

DO40 <- data.frame(do40total,do40ferm, do40nonferm, do40fullfat, do40nonfat, do40s
DO40
```

A data.frame: 5 × 6

do40total	do40ferm	do40nonferm	do40fullfat	do40nonfat	do40sug
<chr>	<chr>	<chr>	<chr>	<chr>	<chr>
12.3 % (-1.7 % to 26.3 %)	11.7 % (-2.5 % to 25.9 %)	12.9 % (-1.5 % to 27.2 %)	11.4 % (-2.9 % to 25.6 %)	12 % (-2.7 % to 26.6 %)	11.9 % (-3.5 % to 27.3 %)
-17.3 % (-34.2 % to -0.3 %)	-17.9 % (-35 % to -0.7 %)	-16.7 % (-33.9 % to 0.5 %)	-18.2 % (-35.3 % to -1.1 %)	-17.6 % (-35 % to -0.2 %)	-17.6 % (-35.8 % to 0.5 %)
25.6 % (-12.1 % to 63.4 %)	25 % (-12.8 % to 62.8 %)	26.2 % (-11.7 % to 64.1 %)	24.7 % (-13.2 % to 62.5 %)	25.3 % (-12.7 % to 63.2 %)	25.2 % (-13.1 % to 63.5 %)
0.6 % (-3.6 % to 4.8 %)	0 % (-4.8 % to 4.9 %)	1.2 % (-4 % to 6.4 %)	-0.3 % (-5.1 % to 4.5 %)	0.3 % (-5.7 % to 6.2 %)	0.3 % (-7.5 % to 8 %)
1.4 % (-1.1 % to 3.9 %)	0.9 % (-2.6 % to 4.4 %)	2 % (-1.9 % to 6 %)	0.005 % (-2.9 % to 3.9 %)	1.1 % (-3.8 % to 6 %)	1.1 % (-5.8 % to 8 %)

CERAD

In [92]:
```
ch_sensi$F3CERAD_cat <- as.numeric(ch_sensi$F3CERAD_cat)

mem <- ch_sensi %>% filter(!is.na(F3CERAD_cat))

msm.totdairy <- geeglm(F3CERAD_cat ~ total_dairy_avg, data=mem, weights=ipw_totdai
                       corstr="independence")
SEtotal <- coef(summary(msm.totdairy))[,2]

msm.fermdairy <- geeglm(F3CERAD_cat ~ ferm_dairy_avg, data=mem, weights=ipw_fermda
                construct="independence")
SEferm <- coef(summary(msm.fermdairy))[,2]

msm.nonfermdairy <- geeglm(F3CERAD_cat ~ nonferm_dairy_avg, data=mem, weights=ipw_
                corstr="independence")
SEnonferm <- coef(summary(msm.nonfermdairy))[,2]

msm.ffdairy <- geeglm(F3CERAD_cat ~ fullfat_dairy_avg, data=mem, weights=ipw_fullf
                corstr="independence")
SEff <- coef(summary(msm.ffdairy))[,2]

msm.nfdairy <- geeglm(F3CERAD_cat ~ nonfat_dairy_avg, data=mem, weights=ipw_nonfat
                corstr="independence")
SEnf <- coef(summary(msm.nfdairy))[,2]

msm.sugdairy <- geeglm(F3CERAD_cat ~ sugar_dairy_avg, data=mem, weights=ipw_sugdai
                corstr="independence")
SEsug <- coef(summary(msm.sugdairy))[,2]

msm.meat <- geeglm(F3CERAD_cat ~ meat_avg, data=mem, weights=ipw_meat, id=pt,
                corstr="independence")
SEmeat <- coef(summary(msm.meat))[,2]

msm.sea <- geeglm(F3CERAD_cat ~ sea_avg, data=mem, weights=ipw_sea, id=pt,
                corstr="independence")
```

```
SEsea <- coef(summary(msm.sea))[,2]

msm.veg <- geeglm(F3CERAD_cat ~ veg_avg, data=mem, weights=ipw_veg, id=pt,
                corstr="independence")
SEveg <- coef(summary(msm.veg))[,2]

msm.fru <- geeglm(F3CERAD_cat ~ fru_avg, data=mem, weights=ipw_fru, id=pt,
                corstr="independence")
SEfru <- coef(summary(msm.fru))[,2]

msm.eggs <- geeglm(F3CERAD_cat ~ eggs_avg, data=mem, weights=ipw_eggs, id=pt,
                corstr="independence")
SEeggs <- coef(summary(msm.eggs))[,2]

a <- ((coef(msm.totdairy) - coef(msm.meat))*100)[2]
b <- ((coef(msm.totdairy) - coef(msm.meat) - qnorm(0.975) * sqrt(SEtotal^2 + SEmea
c <- ((coef(msm.totdairy) - coef(msm.meat) + qnorm(0.975) * sqrt(SEtotal^2 + SEmea

d <- ((coef(msm.totdairy) - coef(msm.sea))*100)[2]
e <- ((coef(msm.totdairy) - coef(msm.sea) - qnorm(0.975) * sqrt(SEtotal^2 + SEsea^
f <- ((coef(msm.totdairy) - coef(msm.sea) + qnorm(0.975) * sqrt(SEtotal^2 + SEsea^

g <- ((coef(msm.totdairy) - coef(msm.eggs))*100)[2]
h <- ((coef(msm.totdairy) - coef(msm.eggs) - qnorm(0.975) * sqrt(SEtotal^2 + SEegg
i <- ((coef(msm.totdairy) - coef(msm.eggs) + qnorm(0.975) * sqrt(SEtotal^2 + SEegg

j <- ((coef(msm.totdairy) - coef(msm.veg))*100)[2]
k <- ((coef(msm.totdairy) - coef(msm.veg) - qnorm(0.975) * sqrt(SEtotal^2 + SEveg^
l <- ((coef(msm.totdairy) - coef(msm.veg) + qnorm(0.975) * sqrt(SEtotal^2 + SEveg^

m <- ((coef(msm.totdairy) - coef(msm.fru))*100)[2]
n <- ((coef(msm.totdairy) - coef(msm.fru) - qnorm(0.975) * sqrt(SEtotal^2 + SEfru^
o <- ((coef(msm.totdairy) - coef(msm.fru) + qnorm(0.975) * sqrt(SEtotal^2 + SEfru^

ceradtotal <- c(paste(round(a,3)*100, "% (", round(b,3)*100,"% to ", round(c,3)*10
              paste(round(d,3)*100, "% (", round(e,3)*100,"% to ", round(f,3)*100,
              paste(round(g,3)*100, "% (", round(h,3)*100,"% to ", round(i,3)*100,
              paste(round(j,3)*100, "% (", round(k,3)*100,"% to ", round(l,3)*100,
              paste(round(m,3)*100, "% (", round(n,3)*100,"% to ", round(o,3)*100,

a <- ((coef(msm.fermdairy) - coef(msm.meat))*100)[2]
b <- ((coef(msm.fermdairy) - coef(msm.meat) - qnorm(0.975) * sqrt(SEferm^2 + SEmea
c <- ((coef(msm.fermdairy) - coef(msm.meat) + qnorm(0.975) * sqrt(SEferm^2 + SEmea

d <- ((coef(msm.fermdairy) - coef(msm.sea))*100)[2]
e <- ((coef(msm.fermdairy) - coef(msm.sea) - qnorm(0.975) * sqrt(SEferm^2 + SEsea^
f <- ((coef(msm.fermdairy) - coef(msm.sea) + qnorm(0.975) * sqrt(SEferm^2 + SEsea^

g <- ((coef(msm.fermdairy) - coef(msm.eggs))*100)[2]
h <- ((coef(msm.fermdairy) - coef(msm.eggs) - qnorm(0.975) * sqrt(SEferm^2 + SEegg
i <- ((coef(msm.fermdairy) - coef(msm.eggs) + qnorm(0.975) * sqrt(SEferm^2 + SEegg

j <- ((coef(msm.fermdairy) - coef(msm.veg))*100)[2]
k <- ((coef(msm.fermdairy) - coef(msm.veg) - qnorm(0.975) * sqrt(SEferm^2 + SEveg^
l <- ((coef(msm.fermdairy) - coef(msm.veg) + qnorm(0.975) * sqrt(SEferm^2 + SEveg^

m <- ((coef(msm.fermdairy) - coef(msm.fru))*100)[2]
n <- ((coef(msm.fermdairy) - coef(msm.fru) - qnorm(0.975) * sqrt(SEferm^2 + SEfru^
o <- ((coef(msm.fermdairy) - coef(msm.fru) + qnorm(0.975) * sqrt(SEferm^2 + SEfru^

ceradferm <- c(paste(round(a,3)*100, "% (", round(b,3)*100,"% to ", round(c,3)*100
              paste(round(d,3)*100, "% (", round(e,3)*100,"% to ", round(f,3)*100,
              paste(round(g,3)*100, "% (", round(h,3)*100,"% to ", round(i,3)*100,
              paste(round(j,3)*100, "% (", round(k,3)*100,"% to ", round(l,3)*100,
```

```
             paste(round(m,3)*100, "% (", round(n,3)*100,"% to ", round(o,3)*100,

a <- ((coef(msm.nonfermdairy) - coef(msm.meat))*100)[2]
b <- ((coef(msm.nonfermdairy) - coef(msm.meat) - qnorm(0.975) * sqrt(SEnonferm^2 +
c <- ((coef(msm.nonfermdairy) - coef(msm.meat) + qnorm(0.975) * sqrt(SEnonferm^2 +

d <- ((coef(msm.nonfermdairy) - coef(msm.sea))*100)[2]
e <- ((coef(msm.nonfermdairy) - coef(msm.sea) - qnorm(0.975) * sqrt(SEnonferm^2 +
f <- ((coef(msm.nonfermdairy) - coef(msm.sea) + qnorm(0.975) * sqrt(SEnonferm^2 +

g <- ((coef(msm.nonfermdairy) - coef(msm.eggs))*100)[2]
h <- ((coef(msm.nonfermdairy) - coef(msm.eggs) - qnorm(0.975) * sqrt(SEnonferm^2 +
i <- ((coef(msm.nonfermdairy) - coef(msm.eggs) + qnorm(0.975) * sqrt(SEnonferm^2 +

j <- ((coef(msm.nonfermdairy) - coef(msm.veg))*100)[2]
k <- ((coef(msm.nonfermdairy) - coef(msm.veg) - qnorm(0.975) * sqrt(SEnonferm^2 +
l <- ((coef(msm.nonfermdairy) - coef(msm.veg) + qnorm(0.975) * sqrt(SEnonferm^2 +

m <- ((coef(msm.nonfermdairy) - coef(msm.fru))*100)[2]
n <- ((coef(msm.nonfermdairy) - coef(msm.fru) - qnorm(0.975) * sqrt(SEnonferm^2 +
o <- ((coef(msm.nonfermdairy) - coef(msm.fru) + qnorm(0.975) * sqrt(SEnonferm^2 +

ceradnonferm <- c(paste(round(a,3)*100, "% (", round(b,3)*100,"% to ", round(c,3)*
             paste(round(d,3)*100, "% (", round(e,3)*100,"% to ", round(f,3)*100,
             paste(round(g,3)*100, "% (", round(h,3)*100,"% to ", round(i,3)*100,
             paste(round(j,3)*100, "% (", round(k,3)*100,"% to ", round(l,3)*100,
             paste(round(m,3)*100, "% (", round(n,3)*100,"% to ", round(o,3)*100,


a <- ((coef(msm.ffdairy) - coef(msm.meat))*100)[2]
b <- ((coef(msm.ffdairy) - coef(msm.meat) - qnorm(0.975) * sqrt(SEff^2 + SEmeat^2)
c <- ((coef(msm.ffdairy) - coef(msm.meat) + qnorm(0.975) * sqrt(SEff^2 + SEmeat^2)

d <- ((coef(msm.ffdairy) - coef(msm.sea))*100)[2]
e <- ((coef(msm.ffdairy) - coef(msm.sea) - qnorm(0.975) * sqrt(SEff^2 + SEsea^2))*
f <- ((coef(msm.ffdairy) - coef(msm.sea) + qnorm(0.975) * sqrt(SEff^2 + SEsea^2))*

g <- ((coef(msm.ffdairy) - coef(msm.eggs))*100)[2]
h <- ((coef(msm.ffdairy) - coef(msm.eggs) - qnorm(0.975) * sqrt(SEff^2 + SEeggs^2)
i <- ((coef(msm.ffdairy) - coef(msm.eggs) + qnorm(0.975) * sqrt(SEff^2 + SEeggs^2)

j <- ((coef(msm.ffdairy) - coef(msm.veg))*100)[2]
k <- ((coef(msm.ffdairy) - coef(msm.veg) - qnorm(0.975) * sqrt(SEff^2 + SEveg^2))*
l <- ((coef(msm.ffdairy) - coef(msm.veg) + qnorm(0.975) * sqrt(SEff^2 + SEveg^2))*

m <- ((coef(msm.ffdairy) - coef(msm.fru))*100)[2]
n <- ((coef(msm.ffdairy) - coef(msm.fru) - qnorm(0.975) * sqrt(SEff^2 + SEfru^2))*
o <- ((coef(msm.ffdairy) - coef(msm.fru) + qnorm(0.975) * sqrt(SEff^2 + SEfru^2))*

ceradfullfat <- c(paste(round(a,3)*100, "% (", round(b,3)*100,"% to ", round(c,3)*
             paste(round(d,3)*100, "% (", round(e,3)*100,"% to ", round(f,3)*100,
             paste(round(g,3)*100, "% (", round(h,3)*100,"% to ", round(i,3)*100,
             paste(round(j,3)*100, "% (", round(k,3)*100,"% to ", round(l,3)*100,
             paste(round(m,3)*100, "% (", round(n,3)*100,"% to ", round(o,3)*100,


a <- ((coef(msm.nfdairy) - coef(msm.meat))*100)[2]
b <- ((coef(msm.nfdairy) - coef(msm.meat) - qnorm(0.975) * sqrt(SEnf^2 + SEmeat^2)
c <- ((coef(msm.nfdairy) - coef(msm.meat) + qnorm(0.975) * sqrt(SEnf^2 + SEmeat^2)

d <- ((coef(msm.nfdairy) - coef(msm.sea))*100)[2]
e <- ((coef(msm.nfdairy) - coef(msm.sea) - qnorm(0.975) * sqrt(SEnf^2 + SEsea^2))*
f <- ((coef(msm.nfdairy) - coef(msm.sea) + qnorm(0.975) * sqrt(SEnf^2 + SEsea^2))*
```

```
g <- ((coef(msm.nfdairy) - coef(msm.eggs))*100)[2]
h <- ((coef(msm.nfdairy) - coef(msm.eggs) - qnorm(0.975) * sqrt(SEnf^2 + SEeggs^2)
i <- ((coef(msm.nfdairy) - coef(msm.eggs) + qnorm(0.975) * sqrt(SEnf^2 + SEeggs^2)

j <- ((coef(msm.nfdairy) - coef(msm.veg))*100)[2]
k <- ((coef(msm.nfdairy) - coef(msm.veg) - qnorm(0.975) * sqrt(SEnf^2 + SEveg^2))*
l <- ((coef(msm.nfdairy) - coef(msm.veg) + qnorm(0.975) * sqrt(SEnf^2 + SEveg^2))*

m <- ((coef(msm.nfdairy) - coef(msm.fru))*100)[2]
n <- ((coef(msm.nfdairy) - coef(msm.fru) - qnorm(0.975) * sqrt(SEnf^2 + SEfru^2))*
o <- ((coef(msm.nfdairy) - coef(msm.fru) + qnorm(0.975) * sqrt(SEnf^2 + SEfru^2))*

ceradnonfat <- c(paste(round(a,3)*100, "% (", round(b,3)*100,"% to ", round(c,3)*1
              paste(round(d,3)*100, "% (", round(e,3)*100,"% to ", round(f,3)*100,
              paste(round(g,3)*100, "% (", round(h,3)*100,"% to ", round(i,3)*100,
              paste(round(j,3)*100, "% (", round(k,3)*100,"% to ", round(l,3)*100,
              paste(round(m,3)*100, "% (", round(n,3)*100,"% to ", round(o,3)*100,


a <- ((coef(msm.sugdairy) - coef(msm.meat))*100)[2]
b <- ((coef(msm.sugdairy) - coef(msm.meat) - qnorm(0.975) * sqrt(SEsug^2 + SEmeat^
c <- ((coef(msm.sugdairy) - coef(msm.meat) + qnorm(0.975) * sqrt(SEsug^2 + SEmeat^

d <- ((coef(msm.sugdairy) - coef(msm.sea))*100)[2]
e <- ((coef(msm.sugdairy) - coef(msm.sea) - qnorm(0.975) * sqrt(SEsug^2 + SEsea^2)
f <- ((coef(msm.sugdairy) - coef(msm.sea) + qnorm(0.975) * sqrt(SEsug^2 + SEsea^2)

g <- ((coef(msm.sugdairy) - coef(msm.eggs))*100)[2]
h <- ((coef(msm.sugdairy) - coef(msm.eggs) - qnorm(0.975) * sqrt(SEsug^2 + SEeggs^
i <- ((coef(msm.sugdairy) - coef(msm.eggs) + qnorm(0.975) * sqrt(SEsug^2 + SEeggs^

j <- ((coef(msm.sugdairy) - coef(msm.veg))*100)[2]
k <- ((coef(msm.sugdairy) - coef(msm.veg) - qnorm(0.975) * sqrt(SEsug^2 + SEveg^2)
l <- ((coef(msm.sugdairy) - coef(msm.veg) + qnorm(0.975) * sqrt(SEsug^2 + SEveg^2)

m <- ((coef(msm.sugdairy) - coef(msm.fru))*100)[2]
n <- ((coef(msm.sugdairy) - coef(msm.fru) - qnorm(0.975) * sqrt(SEsug^2 + SEfru^2)
o <- ((coef(msm.sugdairy) - coef(msm.fru) + qnorm(0.975) * sqrt(SEsug^2 + SEfru^2)

ceradsug <- c(paste(round(a,3)*100, "% (", round(b,3)*100,"% to ", round(c,3)*100,
              paste(round(d,3)*100, "% (", round(e,3)*100,"% to ", round(f,3)*100,
              paste(round(g,3)*100, "% (", round(h,3)*100,"% to ", round(i,3)*100,
              paste(round(j,3)*100, "% (", round(k,3)*100,"% to ", round(l,3)*100,
              paste(round(m,3)*100, "% (", round(n,3)*100,"% to ", round(o,3)*100,

CERAD <- data.frame(ceradtotal,ceradferm, ceradnonferm, ceradfullfat, ceradnonfat,
CERAD
```

A data.frame: 5 × 6

ceradtotal	ceradferm	ceradnonferm	ceradfullfat	ceradnonfat	ceradsug
<chr>	<chr>	<chr>	<chr>	<chr>	<chr>
-0.4 % (-15.6 % to 14.8 %)	-0.8 % (-16.3 % to 14.7 %)	-1.7 % (-17.4 % to 14.1 %)	-1.1 % (-16.4 % to 14.3 %)	6.7 % (-9.7 % to 23.1 %)	7.6 % (-8.9 % to 24.2 %)
26.6 % (-5.2 % to 58.4 %)	26.2 % (-5.8 % to 58.2 %)	25.3 % (-6.8 % to 57.4 %)	25.9 % (-6 % to 57.8 %)	33.7 % (1.3 % to 66.1 %)	34.6 % (2.1 % to 67.1 %)
-21.6 % (-74 % to 30.9 %)	-22 % (-74.5 % to 30.6 %)	-22.9 % (-75.5 % to 29.8 %)	-22.3 % (-74.7 % to 30.2 %)	-14.5 % (-67.3 % to 38.3 %)	-13.6 % (-66.4 % to 39.3 %)
-1.3 % (-8.3 % to 5.7 %)	-1.7 % (-9.5 % to 6 %)	-2.6 % (-10.8 % to 5.7 %)	-2 % (-9.3 % to 5.4 %)	5.8 % (-3.6 % to 15.2 %)	6.7 % (-3 % to 16.4 %)
0.1 % (-5 % to 5.1 %)	-0.3 % (-6.4 % to 5.7 %)	-1.2 % (-7.8 % to 5.4 %)	-0.6 % (-6.1 % to 4.9 %)	7.2 % (-0.8 % to 15.2 %)	8.1 % (-0.2 % to 16.4 %)

In [93]:
```
pro <- data.frame(CDR, SCD, MEMORY, VERBAL, STROOP,DO40, CERAD)
write.csv(pro, "substitution_sensi.csv")
```
